# Supplementary material for: Characterization of extensive diversity in immunoglobulin light chain variable germline genes across biomedically important mouse strains
Source: Immunohorizons. 2025 Jul 18;9(8):vlaf031. doi: 10.1093/immhor/vlaf031 (PMC12274646; doi:10.1093/immhor/vlaf031)
Supplement: vlaf031_Supplementary_Data [file vlaf031_supplementary_data.pdf]

Supplemental Figure 1. **Count of inferred IGKV genes of each subgroup by strain.** Bar plots showing the count of genes within each subgroup identified for all studied strains (strain indicated by bar color, as per legend). Subgroups are based on IMGT subgroup definitions.

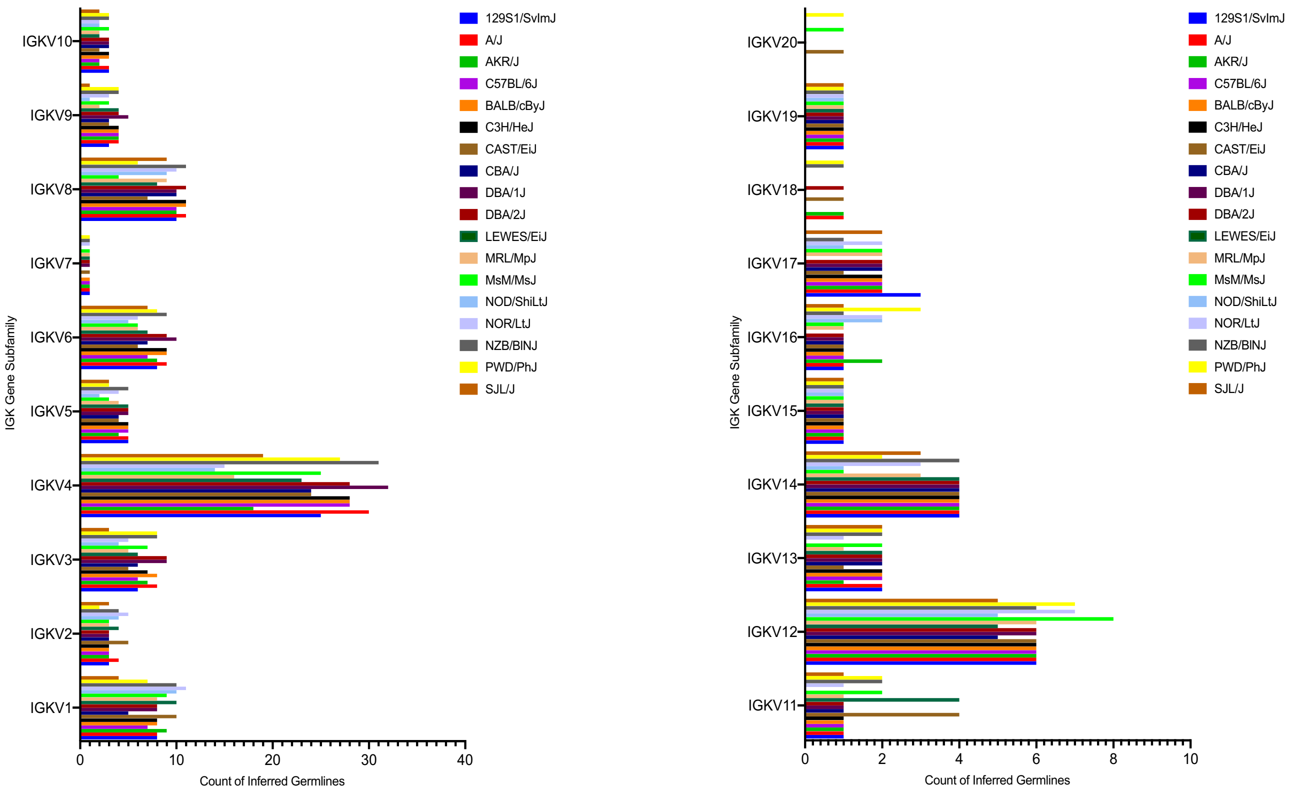

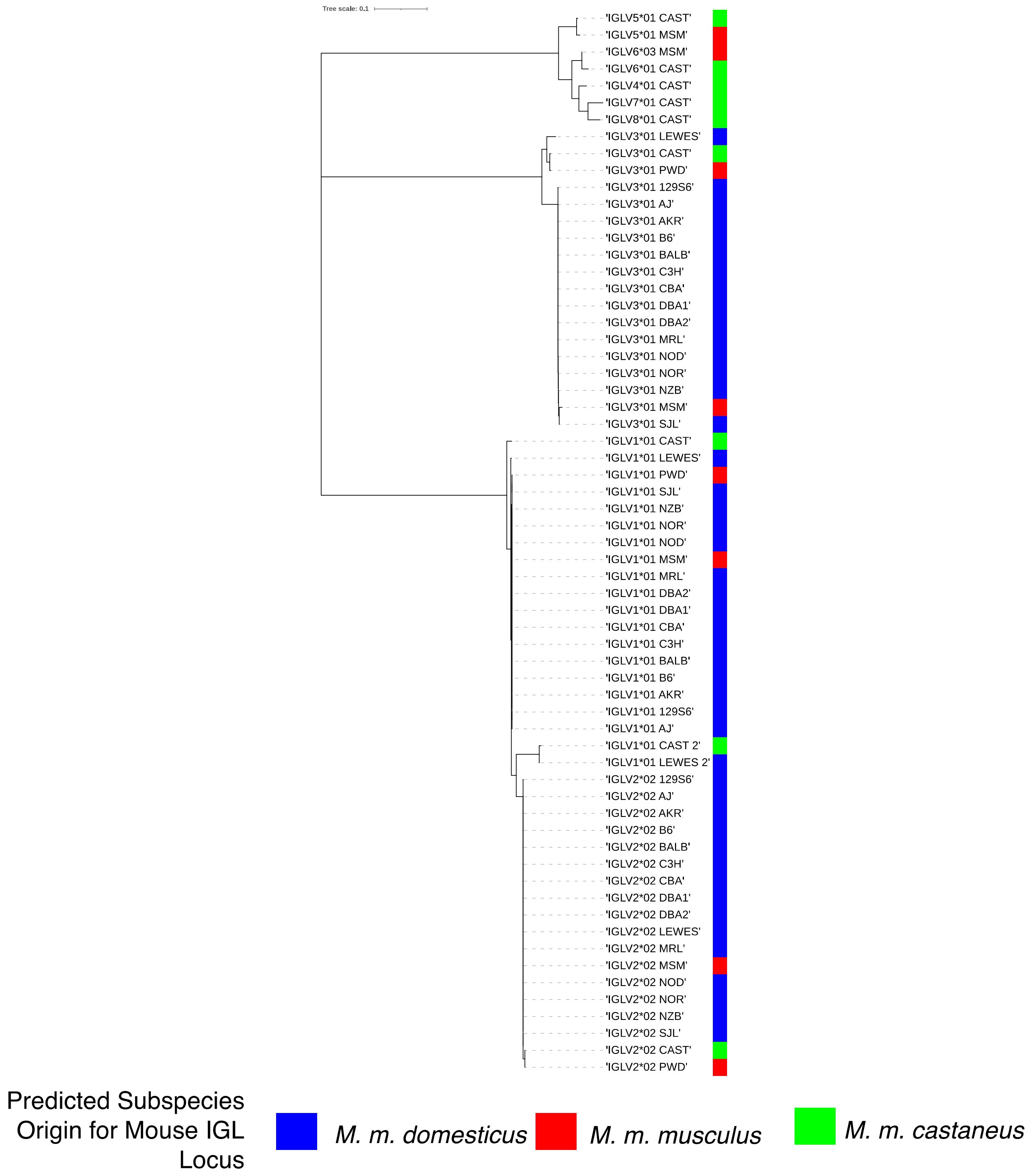

Supplemental Figure 2. Phylogenetic tree of inferred IGLV germline sequences. Neighbor-joining tree based on multiple sequence alignment of all characterized IGLV sequences across strains. Tips of tree are annotated based on the predicted subspecies origin of each strain. Tree scale is indicated above phylogeny.

Supplemental Table 1.

SEQUENCE NAME	STRAIN ID	SEGMENT TYPE	CLOSEST IMGT ALLELE	PERCENT IDENTITY TO IMGT	SEQUENCE
IGKV1-110*01_129	129S1/SVIMJ	IGKV	MUSMUS IGKV1-110*01 F	100	GATGTTGTGATGACCCAAACTCCACTCTCCCTGCCTGTCAGTCTTGGAGATCAAGCCTCCATCTCTTGCAGATCTAGTCAGAGCCTTGTACACAGTAATGGAAACACCTATTTACATTGGTACCTGCAGAAGCCAGGCCAGTCTCCAAAGCTCCTGATCTACAAAGTTTCCAACCGATTTTCTGGGGTCCCAGACAGGTTCAGTGGCAGTGGATCAGGGACAGATTTCACACTCAAGATCAGCAGAGTGGAGGCTGAGGATCTGGGAGTTTATTTCTGCTCTCAAAGTACACATGTTCCTCC
IGKV1-110*01_AJ	A/J	IGKV	MUSMUS IGKV1-110*01 F	100	GATGTTGTGATGACCCAAACTCCACTCTCCCTGCCTGTCAGTCTTGGAGATCAAGCCTCCATCTCTTGCAGATCTAGTCAGAGCCTTGTACACAGTAATGGAAACACCTATTTACATTGGTACCTGCAGAAGCCAGGCCAGTCTCCAAAGCTCCTGATCTACAAAGTTTCCAACCGATTTTCTGGGGTCCCAGACAGGTTCAGTGGCAGTGGATCAGGGACAGATTTCACACTCAAGATCAGCAGAGTGGAGGCTGAGGATCTGGGAGTTTATTTCTGCTCTCAAAGTACACATGTTCCTCC
IGKV1-110*01_AKR	AKR/J	IGKV	MUSMUS IGKV1-110*01 F	100	GATGTTGTGATGACCCAAACTCCACTCTCCCTGCCTGTCAGTCTTGGAGATCAAGCCTCCATCTCTTGCAGATCTAGTCAGAGCCTTGTACACAGTAATGGAAACACCTATTTACATTGGTACCTGCAGAAGCCAGGCCAGTCTCCAAAGCTCCTGATCTACAAAGTTTCCAACCGATTTTCTGGGGTCCCAGACAGGTTCAGTGGCAGTGGATCAGGGACAGATTTCACACTCAAGATCAGCAGAGTGGAGGCTGAGGATCTGGGAGTTTATTTCTGCTCTCAAAGTACACATGTTCCTCC

IGKV1-110*01_B6	C57BL/6J	IGKV	MUSMUS IGKV1-110*01 F	100	GATGTTGTGATGACCCAAACTC<br>CACTCTCCCTGCCTGTCAGTCT<br>TGGAGATCAAGCCTCCATCTCTT<br>GCAGATCTAGTCAGAGCCTTGT<br>ACACAGTAATGGAAACACCTATT<br>TACATTGGTACCTGCAGAAGCC<br>AGGCCAGTCTCCAAAGCTCCTG<br>ATCTACAAAGTTTCCAACCGATT<br>TTCTGGGGTCCCAGACAGGTTC<br>AGTGGCAGTGGATCAGGGACA<br>GATTTCACACTCAAGATCAGCA<br>GAGTGGAGGCTGAGGATCTGG<br>GAGTTTATTTCTGCTCTCAAAGTA<br>CACATGTTCCTCC
IGKV1-110*01_BALB	BALB/CBYJ	IGKV	MUSMUS IGKV1-110*01 F	100	GATGTTGTGATGACCCAAACTC<br>CACTCTCCCTGCCTGTCAGTCT<br>TGGAGATCAAGCCTCCATCTCTT<br>GCAGATCTAGTCAGAGCCTTGT<br>ACACAGTAATGGAAACACCTATT<br>TACATTGGTACCTGCAGAAGCC<br>AGGCCAGTCTCCAAAGCTCCTG<br>ATCTACAAAGTTTCCAACCGATT<br>TTCTGGGGTCCCAGACAGGTTC<br>AGTGGCAGTGGATCAGGGACA<br>GATTTCACACTCAAGATCAGCA<br>GAGTGGAGGCTGAGGATCTGG<br>GAGTTTATTTCTGCTCTCAAAGTA<br>CACATGTTCCTCC
IGKV1-110*01_C3H	C3H/HEJ	IGKV	MUSMUS IGKV1-110*01 F	100	GATGTTGTGATGACCCAAACTC<br>CACTCTCCCTGCCTGTCAGTCT<br>TGGAGATCAAGCCTCCATCTCTT<br>GCAGATCTAGTCAGAGCCTTGT<br>ACACAGTAATGGAAACACCTATT<br>TACATTGGTACCTGCAGAAGCC<br>AGGCCAGTCTCCAAAGCTCCTG<br>ATCTACAAAGTTTCCAACCGATT<br>TTCTGGGGTCCCAGACAGGTTC<br>AGTGGCAGTGGATCAGGGACA<br>GATTTCACACTCAAGATCAGCA<br>GAGTGGAGGCTGAGGATCTGG<br>GAGTTTATTTCTGCTCTCAAAGTA<br>CACATGTTCCTCC
IGKV1-110*01_CBA	CBA/J	IGKV	MUSMUS IGKV1-110*01 F	100	GATGTTGTGATGACCCAAACTC<br>CACTCTCCCTGCCTGTCAGTCT<br>TGGAGATCAAGCCTCCATCTCTT<br>GCAGATCTAGTCAGAGCCTTGT<br>ACACAGTAATGGAAACACCTATT<br>TACATTGGTACCTGCAGAAGCC<br>AGGCCAGTCTCCAAAGCTCCTG<br>ATCTACAAAGTTTCCAACCGATT<br>TTCTGGGGTCCCAGACAGGTTC<br>AGTGGCAGTGGATCAGGGACA<br>GATTTCACACTCAAGATCAGCA<br>GAGTGGAGGCTGAGGATCTGG<br>GAGTTTATTTCTGCTCTCAAAGTA<br>CACATGTTCCTCC

IGKV1-110*01_DBA1	DBA/1J	IGKV	MUSMUS IGKV1-110*01 F	100	GATGTTGTGATGACCCAAACTC<br>CACTCTCCCTGCCTGTCAGTCT<br>TGGAGATCAAGCCTCCATCTCTT<br>GCAGATCTAGTCAGAGCCTTGT<br>ACACAGTAATGGAAACACCTATT<br>TACATTGGTACCTGCAGAAGCC<br>AGGCCAGTCTCCAAAGCTCCTG<br>ATCTACAAAGTTTCCAACCGATT<br>TTCTGGGGTCCCAGACAGGTTC<br>AGTGGCAGTGGATCAGGGACA<br>GATTTCACACTCAAGATCAGCA<br>GAGTGGAGGCTGAGGATCTGG<br>GAGTTTATTTCTGCTCTCAAAGTA<br>CACATGTTCCTCC
IGKV1-110*01_DBA2	DBA/2J	IGKV	MUSMUS IGKV1-110*01 F	100	GATGTTGTGATGACCCAAACTC<br>CACTCTCCCTGCCTGTCAGTCT<br>TGGAGATCAAGCCTCCATCTCTT<br>GCAGATCTAGTCAGAGCCTTGT<br>ACACAGTAATGGAAACACCTATT<br>TACATTGGTACCTGCAGAAGCC<br>AGGCCAGTCTCCAAAGCTCCTG<br>ATCTACAAAGTTTCCAACCGATT<br>TTCTGGGGTCCCAGACAGGTTC<br>AGTGGCAGTGGATCAGGGACA<br>GATTTCACACTCAAGATCAGCA<br>GAGTGGAGGCTGAGGATCTGG<br>GAGTTTATTTCTGCTCTCAAAGTA<br>CACATGTTCCTCC
IGKV1-110*01_MRL	MRL/MPJ	IGKV	MUSMUS IGKV1-110*01 F	100	GATGTTGTGATGACCCAAACTC<br>CACTCTCCCTGCCTGTCAGTCT<br>TGGAGATCAAGCCTCCATCTCTT<br>GCAGATCTAGTCAGAGCCTTGT<br>ACACAGTAATGGAAACACCTATT<br>TACATTGGTACCTGCAGAAGCC<br>AGGCCAGTCTCCAAAGCTCCTG<br>ATCTACAAAGTTTCCAACCGATT<br>TTCTGGGGTCCCAGACAGGTTC<br>AGTGGCAGTGGATCAGGGACA<br>GATTTCACACTCAAGATCAGCA<br>GAGTGGAGGCTGAGGATCTGG<br>GAGTTTATTTCTGCTCTCAAAGTA<br>CACATGTTCCTCC
IGKV1-110*01_S3077_LEWES	LEWES/EIJ	IGKV	MUSMUS IGKV1-110*01 F, OR MUSMUS IGKV1-117*01 F	97.62	GATGTTATGATGACCCAAACTCC<br>ACTCTCCCTGCCTGTCAGTCTT<br>GGAGATCAAGCCTCCATCTCTT<br>GCAGATCTAGTCAGAGCCTTGT<br>ACACAGTAATGGAAACACCTATT<br>TATATTGGTTCCTGCAGAAACCA<br>GGCCAGTCTCCAAAGCTCCTGA<br>TCTACAAAGTTTCCAACCGATTT<br>TCTGGGGTCCCAGACAGGTTCA<br>GTGGCAGTGGATCAGGGACAG<br>ATTTCACACTCAAGATCAGCAGA<br>GTGGAGGCTGAGGATCTGGGA<br>GTTTATTACTGCTTTCAAGGTACA<br>CATGTTCCTC

IGKV1-110*01_S5250_MSM	MSM/MSJ	IGKV	MUSMUS IGKV1-110*01 F	97.96	GATGTTTTGATGACCCAAACTCCACTCTCCCTGCCTGTCAGTCTTGGAGATCAAGCCTCCATCTCTTGCAGATCTAGTCAGAGCCTTGTACACAGTAATGGAAACACCTATTTATATTGGTACCTGCAGAAGCCAGGCCAGTCTCCAAAGCTCCTGATCTACAAAGTTTCCAACCGATTTTCTGGGGTCCCAGACAGGTTCAGTGGCAGTGGATCAGGGACAGATTTCACACTCAAGATCAGCAGAGTGGAGGCTGAGGATATGGGAGTTTATTACTGCTTTCAAGGTACACATGTTCCTCC
IGKV1-110*01_S5755_PWD	PWD/PHJ	IGKV	MUSMUS IGKV1-110*01 F	97.96	GATGTTTTGATGACCCAAACTCCACTCTCCCTGCCTGTCAGTCTTGGAGATCAAGCCTCCATCTCTTGCAGATCTAGTCAGAGCCTTGTACACAGTAATGGAAACACCTATTTATATTGGTACCTGCAGAAGCCAGGCCAGTCTCCAAAGCTCCTGATCTACAAAGTTTCCAACCGATTTTCTGGGGTCCCAGACAGGTTCAGTGGCAGTGGATCAGGGACAGATTTCACACTCAAGATCAGCAGAGTGGAGGCTGAGGATATGGGAGTTTATTACTGCTTTCAAGGTACACATGTTCCTC
IGKV1-110*01_S6010_CAST	CAST/EIJ	IGKV	MUSMUS IGKV1-110*01 F	98.3	GATGTTGTGATGACCCAAACTCCACTCTCCCTGCCTGTCAGTCTTGGAGATCAAGCCTCCATCTCTTGCAGATCTAGTCAGAGCCTTGTACACAGTAATGGAAACACCTATTTACATTGGTACCTGCAGAAACCAGGCCAGTCTCCAAAGCTCCTGATCTACAGGGTTTCCAACCGATTTTCTGGGGTCCCAGACAGGTTCAGTGGCAGTGGATCAGGGACAGATTTCACACTCAAGATCAGCAGAGTGGAGGCTGAGGATCTGGGAGTTTATTACTGCTTTCAAAGTACACATGTTCCTC
IGKV1-110*01_SJL	SJL/J	IGKV	MUSMUS IGKV1-110*01 F	100	GATGTTGTGATGACCCAAACTCCACTCTCCCTGCCTGTCAGTCTTGGAGATCAAGCCTCCATCTCTTGCAGATCTAGTCAGAGCCTTGTACACAGTAATGGAAACACCTATTTACATTGGTACCTGCAGAAGCCAGGCCAGTCTCCAAAGCTCCTGATCTACAAAGTTTCCAACCGATTTTCTGGGGTCCCAGACAGGTTCAGTGGCAGTGGATCAGGGACAGATTTCACACTCAAGATCAGCAGAGTGGAGGCTGAGGATCTGGGAGTTTATTTCTGCTCTCAAAGTACACATGTTCCTCC

IGKV1-110*02_NOD	NOD/SHILTJ	IGKV	MUSMUS IGKV1-110*02 F	100	GATGTTGTGATGACCCAAACTCCACTCTCCCTGCCTGTCAGTCTTGGAGATCAAGCTTCCATCTCTTGCAGATCTAGTCAGAGCCTTGTACACAGCAATGGAAACACCTATTTATATTGGTACCTGCAGAAGCCAGGCCAGTCTCCAAAGCTCCTGATCTACAGGGTTTCCAACCGATTTTCTGGGGTCCCAGACAGGTTCAGTGGCAGTGGATCAGGGACAGATTTCACACTCAAGATCAGCAGAGTGGAGGCTGAGGATCTGGGAGTTTATTTCTGCTTTCAAGGTACACATGTTCCTCA
IGKV1-110*02_NOR	NOR/LTJ	IGKV	MUSMUS IGKV1-110*02 F	100	GATGTTGTGATGACCCAAACTCCACTCTCCCTGCCTGTCAGTCTTGGAGATCAAGCTTCCATCTCTTGCAGATCTAGTCAGAGCCTTGTACACAGCAATGGAAACACCTATTTATATTGGTACCTGCAGAAGCCAGGCCAGTCTCCAAAGCTCCTGATCTACAGGGTTTCCAACCGATTTTCTGGGGTCCCAGACAGGTTCAGTGGCAGTGGATCAGGGACAGATTTCACACTCAAGATCAGCAGAGTGGAGGCTGAGGATCTGGGAGTTTATTTCTGCTTTCAAGGTACACATGTTCCTCA
IGKV1-110*02_NZB	NZB/BLNJ	IGKV	MUSMUS IGKV1-110*02 F	100	GATGTTGTGATGACCCAAACTCCACTCTCCCTGCCTGTCAGTCTTGGAGATCAAGCTTCCATCTCTTGCAGATCTAGTCAGAGCCTTGTACACAGCAATGGAAACACCTATTTATATTGGTACCTGCAGAAGCCAGGCCAGTCTCCAAAGCTCCTGATCTACAGGGTTTCCAACCGATTTTCTGGGGTCCCAGACAGGTTCAGTGGCAGTGGATCAGGGACAGATTTCACACTCAAGATCAGCAGAGTGGAGGCTGAGGATCTGGGAGTTTATTTCTGCTTTCAAGGTACACATGTTCCTCA
IGKV1-110*02_S1372_LEWES	LEWES/EIJ	IGKV	MUSMUS IGKV1-110*02 F, OR MUSMUS IGKV1-117*01 F	95.92	GATGTTGTGATGACCCAAACTCCACTCTCCCTGCCTGTCAGTCTTGGAGATCAAGCCTCCATCTCTTGCAGATCTAGTCAGAGCATTGTACACAGTAATAGATACACCTATTTAGAATGGTACCTGCAGAAACCAGGCCAGTCTCTAAAGCTCCTGATATACGGGGTTTCCAACCGATTTTCTGGGGTCCCAGACAGGTTCAGTGGCAGTGGATCAAGGACAGATTTCACACTCAAGATCAGCAGAGTGGAGGCTGAGGATCTGGGAGTTTATTTCTGCTTTCAAGGTACACATGTTCCTCA

IGKV1-110*02_S1372_PWD	PWD/PHJ	IGKV	MUSMUS IGKV1-110*02 F, OR MUSMUS IGKV1-117*01 F	95.92	GATGTTGTGATGACCCAAACTCCACTCTCCCTGCCTGTCAGTCTTGGAGATCAAGCCTCCATCTCTTGCAGATCTAGTCAGAGCATTGTACACAGTAATAGATACACCTATTTAGAATGGTACCTGCAGAAACCAGGCCAGTCTCTAAAGCTCCTGATATACGGGGTTTCCAACCGATTTTCTGGGGTCCCAGACAGGTTCAGTGGCAGTGGATCAAGGACAGATTTCACACTCAAGATCAGCAGAGTGGAGGCTGAGGATCTGGGAGTTTATTTCTGCTTTCAAGGTACACATGTTCCTCA
IGKV1-110*02_S9753_CAST	CAST/EIJ	IGKV	MUSMUS IGKV1-110*02 F	97.28	GATGTTGTGATGACCCAAACTCCACTCTCCCTGCCTGTCAGTCTTGGAGATCAAGCCTCCATCTCTTGCAGATCTAGTCAGAGCATTGTAGCCAGTAATGGAAACACCTATTTATATTGGTACCTGCAGAAGCCAGGCCAGTCTCCAAAGCTCCTGATCTACAGGGTTTCCAACAGATTTTCTGGGGTCCCAGACAGGTTCAGTGGCAGTGGATCAGGGACAGATTTCACTCTCAAGATCAGCAGAGTGGAGGCTGAGGATCTGGGAGTTTATTACTGCTTTCAAGGTACACATGTTCCTCA
IGKV1-115*01_S3051_NZB	NZB/BLNJ	IGKV	MUSMUS IGKV1-115*01 P	95.58	GATGTTCTGTTGACCCAAACTCCACTTTTCCTGCATGTCAGCCTTGGAGATCAAGCCTCTATCTCTTGCACATCTAGTCAGAGTCTTGTACACAGTAATGGAAATTCCTATTTGGATTGGCACCTGCAGAAGCCAGACCAGTCTCTACAACTCCTGATCTATGAGGTTTCCAAACGAAATTCTGGGGTTCCAGACAGGTTCAGTGGCAGTGGATCAGGAACAGATTTCACACTTAAGATCAGCAGAGTAGAGCCTGAGGATTTGGGAGTTTATTACTGCTTCCAACGTACACATTTACCTC
IGKV1-115*01_S3088_CAST	CAST/EIJ	IGKV	MUSMUS IGKV1-115*01 P	95.24	GATGTTCTGTTGACCCAAACTCCACTTTTCCTGCATGTCAGCCTTGGAGATCAAGCCTCTATCTCTTGCACATCTAGTCAGAGTCTTGTACACAGTAATGGAAATTCCTATTTGGATTGGCACCTGCAGAAGCCAGACCAGTCTCTACAACTCCTGATCTATGAGGTTTCTAAACGAAATTCTGGGGTTCCAGACAGGTTCAGTGGCAGTGGATCAGGAACAGATTTCACACTTAAGATCAGCAGAGTAGAGCCTGAGGATTTGGGAGTTTATTACTGCTTCCAACGTACACATTTACCTCC

IGKV1-115*01_S9289_NOR	NOR/LTJ	IGKV	MUSMUS IGKV1-115*01 P	95.58	GATGTTCTGTTGACCCAAACTCCACTTTTCCTGCATGTCAGCCTTGGAGATCAAGCCTCTATCTCTTGCACATCTAGTCAGAGTCTTGTACACAGTAATGGAAATTCCTATTTGGATTGGCACCTGCAGAAGCCAGACCAGTCTCTACAACTCCTGATCTATGAGGTTTCCAAACGAAATTCTGGGGTTCCAGACAGGTTCAGTGGCAGTGGATCAGGAACAGATTTCACACTTAAGATCAGCAGAGTAGAGCCTGAGGATTTGGGAGTTTATTACTGCTTCCAACGTACACATTTACCTCC
IGKV1-117*01_129	129S1/SVIMJ	IGKV	MUSMUS IGKV1-117*01 F	100	GATGTTTTGATGACCCAAACTCCACTCTCCCTGCCTGTCAGTCTTGGAGATCAAGCCTCCATCTCTTGCAGATCTAGTCAGAGCATTGTACATAGTAATGGAAACACCTATTTAGAATGGTACCTGCAGAAACCAGGCCAGTCTCCAAAGCTCCTGATCTACAAAGTTTCCAACCGATTTTCTGGGGTCCCAGACAGGTTCAGTGGCAGTGGATCAGGGACAGATTTCACACTCAAGATCAGCAGAGTGGAGGCTGAGGATCTGGGAGTTTATTACTGCTTTCAAGGTTCACATGTTCCTCC
IGKV1-117*01_AJ	A/J	IGKV	MUSMUS IGKV1-117*01 F	100	GATGTTTTGATGACCCAAACTCCACTCTCCCTGCCTGTCAGTCTTGGAGATCAAGCCTCCATCTCTTGCAGATCTAGTCAGAGCATTGTACATAGTAATGGAAACACCTATTTAGAATGGTACCTGCAGAAACCAGGCCAGTCTCCAAAGCTCCTGATCTACAAAGTTTCCAACCGATTTTCTGGGGTCCCAGACAGGTTCAGTGGCAGTGGATCAGGGACAGATTTCACACTCAAGATCAGCAGAGTGGAGGCTGAGGATCTGGGAGTTTATTACTGCTTTCAAGGTTCACATGTTCCTCC
IGKV1-117*01_AKR	AKR/J	IGKV	MUSMUS IGKV1-117*01 F	100	GATGTTTTGATGACCCAAACTCCACTCTCCCTGCCTGTCAGTCTTGGAGATCAAGCCTCCATCTCTTGCAGATCTAGTCAGAGCATTGTACATAGTAATGGAAACACCTATTTAGAATGGTACCTGCAGAAACCAGGCCAGTCTCCAAAGCTCCTGATCTACAAAGTTTCCAACCGATTTTCTGGGGTCCCAGACAGGTTCAGTGGCAGTGGATCAGGGACAGATTTCACACTCAAGATCAGCAGAGTGGAGGCTGAGGATCTGGGAGTTTATTACTGCTTTCAAGGTTCACATGTTCCTCC

IGKV1-117*01_B6	C57BL/6J	IGKV	MUSMUS IGKV1-117*01 F	100	GATGTTTTGATGACCCAAACTCCACTCTCCCTGCCTGTCAGTCTTGGAGATCAAGCCTCCATCTCTTGCAGATCTAGTCAGAGCATTGTACATAGTAATGGAAACACCTATTTAGAATGGTACCTGCAGAAACCAGGCCAGTCTCCAAAGCTCCTGATCTACAAAGTTTCCAACCGATTTTCTGGGGTCCCAGACAGGTTCAGTGGCAGTGGATCAGGGACAGATTTCACACTCAAGATCAGCAGAGTGGAGGCTGAGGATCTGGGAGTTTATTACTGCTTTCAAGGTTCACATGTTCCTCC
IGKV1-117*01_BALB	BALB/CBYJ	IGKV	MUSMUS IGKV1-117*01 F	100	GATGTTTTGATGACCCAAACTCCACTCTCCCTGCCTGTCAGTCTTGGAGATCAAGCCTCCATCTCTTGCAGATCTAGTCAGAGCATTGTACATAGTAATGGAAACACCTATTTAGAATGGTACCTGCAGAAACCAGGCCAGTCTCCAAAGCTCCTGATCTACAAAGTTTCCAACCGATTTTCTGGGGTCCCAGACAGGTTCAGTGGCAGTGGATCAGGGACAGATTTCACACTCAAGATCAGCAGAGTGGAGGCTGAGGATCTGGGAGTTTATTACTGCTTTCAAGGTTCACATGTTCCTCC
IGKV1-117*01_C3H	C3H/HEJ	IGKV	MUSMUS IGKV1-117*01 F	100	GATGTTTTGATGACCCAAACTCCACTCTCCCTGCCTGTCAGTCTTGGAGATCAAGCCTCCATCTCTTGCAGATCTAGTCAGAGCATTGTACATAGTAATGGAAACACCTATTTAGAATGGTACCTGCAGAAACCAGGCCAGTCTCCAAAGCTCCTGATCTACAAAGTTTCCAACCGATTTTCTGGGGTCCCAGACAGGTTCAGTGGCAGTGGATCAGGGACAGATTTCACACTCAAGATCAGCAGAGTGGAGGCTGAGGATCTGGGAGTTTATTACTGCTTTCAAGGTTCACATGTTCCTCC
IGKV1-117*01_CBA	CBA/J	IGKV	MUSMUS IGKV1-117*01 F	100	GATGTTTTGATGACCCAAACTCCACTCTCCCTGCCTGTCAGTCTTGGAGATCAAGCCTCCATCTCTTGCAGATCTAGTCAGAGCATTGTACATAGTAATGGAAACACCTATTTAGAATGGTACCTGCAGAAACCAGGCCAGTCTCCAAAGCTCCTGATCTACAAAGTTTCCAACCGATTTTCTGGGGTCCCAGACAGGTTCAGTGGCAGTGGATCAGGGACAGATTTCACACTCAAGATCAGCAGAGTGGAGGCTGAGGATCTGGGAGTTTATTACTGCTTTCAAGGTTCACATGTTCCTCC

IGKV1-117*01_DBA1	DBA/1J	IGKV	MUSMUS IGKV1-117*01 F	100	GATGTTTTGATGACCCAAACTCCACTCTCCCTGCCTGTCAGTCTTGGAGATCAAGCCTCCATCTCTTGCAGATCTAGTCAGAGCATTGTACATAGTAATGGAAACACCTATTTAGAATGGTACCTGCAGAAACCAGGCCAGTCTCCAAAGCTCCTGATCTACAAAGTTTCCAACCGATTTTCTGGGGTCCCAGACAGGTTCAGTGGCAGTGGATCAGGGACAGATTTCACACTCAAGATCAGCAGAGTGGAGGCTGAGGATCTGGGAGTTTATTACTGCTTTCAAGGTTCACATGTTCCTCC
IGKV1-117*01_DBA2	DBA/2J	IGKV	MUSMUS IGKV1-117*01 F	100	GATGTTTTGATGACCCAAACTCCACTCTCCCTGCCTGTCAGTCTTGGAGATCAAGCCTCCATCTCTTGCAGATCTAGTCAGAGCATTGTACATAGTAATGGAAACACCTATTTAGAATGGTACCTGCAGAAACCAGGCCAGTCTCCAAAGCTCCTGATCTACAAAGTTTCCAACCGATTTTCTGGGGTCCCAGACAGGTTCAGTGGCAGTGGATCAGGGACAGATTTCACACTCAAGATCAGCAGAGTGGAGGCTGAGGATCTGGGAGTTTATTACTGCTTTCAAGGTTCACATGTTCCTCC
IGKV1-117*01_MRL	MRL/MPJ	IGKV	MUSMUS IGKV1-117*01 F	100	GATGTTTTGATGACCCAAACTCCACTCTCCCTGCCTGTCAGTCTTGGAGATCAAGCCTCCATCTCTTGCAGATCTAGTCAGAGCATTGTACATAGTAATGGAAACACCTATTTAGAATGGTACCTGCAGAAACCAGGCCAGTCTCCAAAGCTCCTGATCTACAAAGTTTCCAACCGATTTTCTGGGGTCCCAGACAGGTTCAGTGGCAGTGGATCAGGGACAGATTTCACACTCAAGATCAGCAGAGTGGAGGCTGAGGATCTGGGAGTTTATTACTGCTTTCAAGGTTCACATGTTCCTCC
IGKV1-117*01_S1348_CAST	CAST/EIJ	IGKV	MUSMUS IGKV1-117*01 F	99.32	GATGTTGTGATGACCCAAACTCCACTCTCCCTGCCTGTCAGTCTTGGAGATCAAGCCTCCATCTCTTGCAGATCTAGTCAGAGCATTGTACATAGTAATGGAAACACCTATTTAGAATGGTACCTGCAGAAGCCAGGCCAGTCTCCAAAGCTCCTGATCTACAAAGTTTCCAACCGATTTTCTGGGGTCCCAGACAGGTTCAGTGGCAGTGGATCAGGGACAGATTTCACACTCAAGATCAGCAGAGTGGAGGCTGAGGATCTGGGAGTTTATTACTGCTTTCAAGGTTCACATGTTCCTC

IGKV1-117*01_S1348_NOD	NOD/SHILTJ	IGKV	MUSMUS IGKV1-117*01 F	99.32	GATGTTGTGATGACCCAAACTCCACTCTCCCTGCCTGTCAGTCTTGGAGATCAAGCCTCCATCTCTTGCAGATCTAGTCAGAGCATTGTACATAGTAATGGAAACACCTATTTAGAATGGTACCTGCAGAAGCCAGGCCAGTCTCCAAAGCTCCTGATCTACAAAGTTTCCAACCGATTTTCTGGGGTCCCAGACAGGTTCAGTGGCAGTGGATCAGGGACAGATTTCACACTCAAGATCAGCAGAGTGGAGGCTGAGGATCTGGGAGTTTATTACTGCTTTCAAGGTTCACATGTTCCTC
IGKV1-117*01_S1348_NOR	NOR/LTJ	IGKV	MUSMUS IGKV1-117*01 F	99.32	GATGTTGTGATGACCCAAACTCCACTCTCCCTGCCTGTCAGTCTTGGAGATCAAGCCTCCATCTCTTGCAGATCTAGTCAGAGCATTGTACATAGTAATGGAAACACCTATTTAGAATGGTACCTGCAGAAGCCAGGCCAGTCTCCAAAGCTCCTGATCTACAAAGTTTCCAACCGATTTTCTGGGGTCCCAGACAGGTTCAGTGGCAGTGGATCAGGGACAGATTTCACACTCAAGATCAGCAGAGTGGAGGCTGAGGATCTGGGAGTTTATTACTGCTTTCAAGGTTCACATGTTCCTC
IGKV1-117*01_S1348_NZB	NZB/BLNJ	IGKV	MUSMUS IGKV1-117*01 F	99.32	GATGTTGTGATGACCCAAACTCCACTCTCCCTGCCTGTCAGTCTTGGAGATCAAGCCTCCATCTCTTGCAGATCTAGTCAGAGCATTGTACATAGTAATGGAAACACCTATTTAGAATGGTACCTGCAGAAGCCAGGCCAGTCTCCAAAGCTCCTGATCTACAAAGTTTCCAACCGATTTTCTGGGGTCCCAGACAGGTTCAGTGGCAGTGGATCAGGGACAGATTTCACACTCAAGATCAGCAGAGTGGAGGCTGAGGATCTGGGAGTTTATTACTGCTTTCAAGGTTCACATGTTCCTC
IGKV1-117*01_S7519_LEWES	LEWES/EIJ	IGKV	MUSMUS IGKV1-117*01 F	96.94	GATGTTTTGATGACCCAAACTCCACTCTCCCTGCCTGTCAGTCTTGGAGATCAAGCCTCCATCTCTTGCAGATCTAGTCAGAGCCTTGTACACAGTAATGGAAACACCTATTTATATTGGTTACTGCAGAAGTCAGGCCAGTCTCCAAAGCTCCTGATCTACAAAGTTTCCAACCGATTTTCTGGGGTCCCAGACAGGTTCAGTGGCAATGGATCAGGGACAGATTTCACACTCAAGATCAGCAGAGTGGAGGCTGAGGATCTGGGAGTTTATTACTGCTTTCAAGGTTCACATGTTCCTCC

IGKV1-117*01_SJL	SJL/J	IGKV	MUSMUS IGKV1-117*01 F	100	GATGTTTTGATGACCCAAACTCCACTCTCCCTGCCTGTCAGTCTTGGAGATCAAGCCTCCATCTCTTGCAGATCTAGTCAGAGCATTGTACATAGTAATGGAAACACCTATTTAGAATGGTACCTGCAGAAACCAGGCCAGTCTCCAAAGCTCCTGATCTACAAAGTTTCCAACCGATTTTCTGGGGTCCCAGACAGGTTCAGTGGCAGTGGATCAGGGACAGATTTCACACTCAAGATCAGCAGAGTGGAGGCTGAGGATCTGGGAGTTTATTACTGCTTTCAAGGTTCACATGTTCCTCC
IGKV1-117*02_S1601_MSM	MSM/MSJ	IGKV	MUSMUS IGKV1-117*01 F, OR MUSMUS IGKV1-117*02 F	99.32	GATGTTGTGATGACCCAAACTCCACTCTCCCTGCCTGTCAGTCTTGGAGATCAAGCCTCCATCTCTTGCAGATCTAGTCAGAGCATTGTACATAGTAATGGAAACACCTATTTAGAATGGTACCTGCAGAAACCAGGCCAGTCTCCAAAGCTCCTGATCTACAAAGTTTCCAACCGATTTTCTGGGGTCCCAGACAGGTTCAGTGGCAGTGGATCAGGGACAGACTTCACACTCAAGATCAGCAGAGTGGAGGCTGAGGATCTGGGAGTTTATTACTGCTTTCAAGGTTCACATGTTCCTCC
IGKV1-117*02_S1951_PWD	PWD/PHJ	IGKV	MUSMUS IGKV1-117*02 F	99.66	GATGTTGTGATGACCCAAACTCCACTCTCCCTGCCTGTCAGTCTTGGAGATCAAGCCTCCATCTCTTGCAGATCTAGTCAGAGCATTGTACATAGTAATGGAAACACCTATTTAGAATGGTACCTGCAGAAACCAGGCCAGTCTCCAAAGCTCCTGATCTACAAAGTTTCCAACCGATTGTCTGGGGTCCCAGACAGGTTCAGTGGCAGTGGATCAGGGACAGACTTCACACTCAAGATCAGCAGAGTGGAGGCTGAGGATCTGGGAGTTTATTACTGCTTTCAAGGTTCACATGTTCCTCC
IGKV1-117*02_S5705_LEWES	LEWES/EIJ	IGKV	MUSMUS IGKV1-117*02 F	99.66	GATGTTGTGATGACCCAAACTCCACTCTCCCTGCCTGTCAGTCTTGGAGATCAAGCCTCCATCTCTTGCAGATCTAGTCAGAGCATTGTACATAGTAATGGAAACACCTATTTAGAATGGTACCTGCAGAAACCAGGCCAGTCTCCAAAGCTCCTGATCTACAAAGTTTCCAACCGATTGTCTGGGGTCCCAGACAGGTTCAGTGGCAGTGGATCAGGGACAGACTTCACACTCAAGATCAGCAGAGTGGAGGCTGAGGATCTGGGAGTTTATTACTGCTTTCAAGGTTCACATGTTCCTC

IGKV1-122*01_129	129S1/SVIMJ	IGKV	MUSMUS IGKV1-122*01 F	100	GATGCTGTGATGACCCAAACTCCACTCTCCCTGCCTGTCAGTCTTGGAGATCAAGCCTCCATCTCTTGCAGGTCTAGTCAGAGCCTTGAAAACAGTAATGGAAACACCTATTTGAACTGGTACCTCCAGAAACCAGGCCAGTCTCCACAGCTCCTGATCTACAGGGTTTCCAACCGATTTTCTGGGGTCCTAGACAGGTTCAGTGGTAGTGGATCAGGGACAGATTTCACACTGAAAATCAGCAGAGTGGAGGCTGAGGATTTGGGAGTTTATTTCTGCCTCCAAGTTACACATGTCCCTCC
IGKV1-122*01_AJ	A/J	IGKV	MUSMUS IGKV1-122*01 F	100	GATGCTGTGATGACCCAAACTCCACTCTCCCTGCCTGTCAGTCTTGGAGATCAAGCCTCCATCTCTTGCAGGTCTAGTCAGAGCCTTGAAAACAGTAATGGAAACACCTATTTGAACTGGTACCTCCAGAAACCAGGCCAGTCTCCACAGCTCCTGATCTACAGGGTTTCCAACCGATTTTCTGGGGTCCTAGACAGGTTCAGTGGTAGTGGATCAGGGACAGATTTCACACTGAAAATCAGCAGAGTGGAGGCTGAGGATTTGGGAGTTTATTTCTGCCTCCAAGTTACACATGTCCCTCC
IGKV1-122*01_AKR	AKR/J	IGKV	MUSMUS IGKV1-122*01 F	100	GATGCTGTGATGACCCAAACTCCACTCTCCCTGCCTGTCAGTCTTGGAGATCAAGCCTCCATCTCTTGCAGGTCTAGTCAGAGCCTTGAAAACAGTAATGGAAACACCTATTTGAACTGGTACCTCCAGAAACCAGGCCAGTCTCCACAGCTCCTGATCTACAGGGTTTCCAACCGATTTTCTGGGGTCCTAGACAGGTTCAGTGGTAGTGGATCAGGGACAGATTTCACACTGAAAATCAGCAGAGTGGAGGCTGAGGATTTGGGAGTTTATTTCTGCCTCCAAGTTACACATGTCCCTCC
IGKV1-122*01_B6	C57BL/6J	IGKV	MUSMUS IGKV1-122*01 F	100	GATGCTGTGATGACCCAAACTCCACTCTCCCTGCCTGTCAGTCTTGGAGATCAAGCCTCCATCTCTTGCAGGTCTAGTCAGAGCCTTGAAAACAGTAATGGAAACACCTATTTGAACTGGTACCTCCAGAAACCAGGCCAGTCTCCACAGCTCCTGATCTACAGGGTTTCCAACCGATTTTCTGGGGTCCTAGACAGGTTCAGTGGTAGTGGATCAGGGACAGATTTCACACTGAAAATCAGCAGAGTGGAGGCTGAGGATTTGGGAGTTTATTTCTGCCTCCAAGTTACACATGTCCCTCC

IGKV1-122*01_BALB	BALB/CBYJ	IGKV	MUSMUS IGKV1-122*01 F	100	GATGCTGTGATGACCCAAACTC<br>CACTCTCCCTGCCTGTCAGTCT<br>TGGAGATCAAGCCTCCATCTCTT<br>GCAGGTCTAGTCAGAGCCTTGA<br>AAACAGTAATGGAAACACCTATT<br>TGAACTGGTACCTCCAGAAACC<br>AGGCCAGTCTCCACAGCTCCT<br>GATCTACAGGGTTTCCAACCGA<br>TTTTCTGGGGTCCTAGACAGGTT<br>CAGTGGTAGTGGATCAGGGACA<br>GATTTCACACTGAAAATCAGCAG<br>AGTGGAGGCTGAGGATTTGGGA<br>GTTTATTTCTGCCTCCAAGTTAC<br>ACATGTCCCTCC
IGKV1-122*01_C3H	C3H/HEJ	IGKV	MUSMUS IGKV1-122*01 F	100	GATGCTGTGATGACCCAAACTC<br>CACTCTCCCTGCCTGTCAGTCT<br>TGGAGATCAAGCCTCCATCTCTT<br>GCAGGTCTAGTCAGAGCCTTGA<br>AAACAGTAATGGAAACACCTATT<br>TGAACTGGTACCTCCAGAAACC<br>AGGCCAGTCTCCACAGCTCCT<br>GATCTACAGGGTTTCCAACCGA<br>TTTTCTGGGGTCCTAGACAGGTT<br>CAGTGGTAGTGGATCAGGGACA<br>GATTTCACACTGAAAATCAGCAG<br>AGTGGAGGCTGAGGATTTGGGA<br>GTTTATTTCTGCCTCCAAGTTAC<br>ACATGTCCCTCC
IGKV1-122*01_CBA	CBA/J	IGKV	MUSMUS IGKV1-122*01 F	100	GATGCTGTGATGACCCAAACTC<br>CACTCTCCCTGCCTGTCAGTCT<br>TGGAGATCAAGCCTCCATCTCTT<br>GCAGGTCTAGTCAGAGCCTTGA<br>AAACAGTAATGGAAACACCTATT<br>TGAACTGGTACCTCCAGAAACC<br>AGGCCAGTCTCCACAGCTCCT<br>GATCTACAGGGTTTCCAACCGA<br>TTTTCTGGGGTCCTAGACAGGTT<br>CAGTGGTAGTGGATCAGGGACA<br>GATTTCACACTGAAAATCAGCAG<br>AGTGGAGGCTGAGGATTTGGGA<br>GTTTATTTCTGCCTCCAAGTTAC<br>ACATGTCCCTCC
IGKV1-122*01_DBA1	DBA/1J	IGKV	MUSMUS IGKV1-122*01 F	100	GATGCTGTGATGACCCAAACTC<br>CACTCTCCCTGCCTGTCAGTCT<br>TGGAGATCAAGCCTCCATCTCTT<br>GCAGGTCTAGTCAGAGCCTTGA<br>AAACAGTAATGGAAACACCTATT<br>TGAACTGGTACCTCCAGAAACC<br>AGGCCAGTCTCCACAGCTCCT<br>GATCTACAGGGTTTCCAACCGA<br>TTTTCTGGGGTCCTAGACAGGTT<br>CAGTGGTAGTGGATCAGGGACA<br>GATTTCACACTGAAAATCAGCAG<br>AGTGGAGGCTGAGGATTTGGGA<br>GTTTATTTCTGCCTCCAAGTTAC<br>ACATGTCCCTCC

IGKV1-122*01_DBA2	DBA/2J	IGKV	MUSMUS IGKV1-122*01 F	100	GATGCTGTGATGACCCAAACTCCACTCTCCCTGCCTGTCAGTCTTGGAGATCAAGCCTCCATCTCTTGCAGGTCTAGTCAGAGCCTTGAAAACAGTAATGGAAACACCTATTTGAACTGGTACCTCCAGAAACCAGGCCAGTCTCCACAGCTCCTGATCTACAGGGTTTCCAACCGATTTTCTGGGGTCCTAGACAGGTTCAGTGGTAGTGGATCAGGGACAGATTTCACACTGAAAATCAGCAGAGTGGAGGCTGAGGATTTGGGAGTTTATTTCTGCCTCCAAGTTACACATGTCCCTCC
IGKV1-122*01_MRL	MRL/MPJ	IGKV	MUSMUS IGKV1-122*01 F	100	GATGCTGTGATGACCCAAACTCCACTCTCCCTGCCTGTCAGTCTTGGAGATCAAGCCTCCATCTCTTGCAGGTCTAGTCAGAGCCTTGAAAACAGTAATGGAAACACCTATTTGAACTGGTACCTCCAGAAACCAGGCCAGTCTCCACAGCTCCTGATCTACAGGGTTTCCAACCGATTTTCTGGGGTCCTAGACAGGTTCAGTGGTAGTGGATCAGGGACAGATTTCACACTGAAAATCAGCAGAGTGGAGGCTGAGGATTTGGGAGTTTATTTCTGCCTCCAAGTTACACATGTCCCTCC
IGKV1-122*01_S2138_NOD	NOD/SHILTJ	IGKV	MUSMUS IGKV1-122*01 F	98.64	GATGCTGTGATGACCCAAACTCCACTCTCCCTGCCTGTCAGTCTTGGAGATCAAGCCTCCATCTCTTGCAGGTCTAGTCAGAGCCTTGAAAACAGTAATGGAAACACCTATTTGAACTGGTACCTCCAGAAACCAGGCCAGTCTCCACAGCTCCTGATCTACAGGGTTTCCAACCGATTTTCTGGGGTCCTAGACAGGTTCAGTGGCAGTGGTTCAGGGACAGATTTCACACTCAAGATCAGCAGAGTGGAGGCTGAGGATTTGGGAGTTTATTTCTGCCTCCAAGTTACACATGTCCC
IGKV1-122*01_S2905_MSM	MSM/MSJ	IGKV	MUSMUS IGKV1-122*01 F	97.28	GATGCTGTGATGACCCAAACTCCACTCTCCCTGCCTGTCAGTCTTGGAGATCAAGCCTCCATCTCTTGCAGATCTAGTCAGAGCCTTGTACACAGTAATGGAAACACCTATTTGAACTGGTACCTCCAGAAACCAGGCCAGTCTCCACAGCTCTTGATCTACAGGGTTTCCAACCGATTTTCTGGGGTCCTAGACAGGTTCAGTGGCAGTGGTTCAGGGACAGATTTCACACTCAAGATCAGCAGAGTGGAGGCTGAGGATTTGGGAGTTTATTTCTGCCTCCAAGTTACACATGTCCCTCC

IGKV1-122*01_S5263_LEWES	LEWES/EIJ	IGKV	MUSMUS IGKV1-122*01 F	97.28	GATGCTGTGATGACCCAAACTCCACTCTCCCTGCCTGTCAGTCTTGGAGATCAAGCCTCCATCTCTTGCAGATCTAGTCAGAGCCTTGTACACAGTAATGGAAACACCTATTTGAACTGGTACCTCCAGAAACCAGGCCAGTCTCCACAGCTCTTGATCTACAGGGTTTCCAACCGATTTTCTGGGGTCCTAGACAGGTTCAGTGGCAGTGGTTCAGGGACAGATTTCACACTCAAGATCAGCAGAGTGGAGGCTGAGGATTTGGGAGTTTATTTCTGCCTCCAAGTTACACATATCCCTCC
IGKV1-122*01_S7970_CAST	CAST/EIJ	IGKV	MUSMUS IGKV1-122*01 F	98.64	GATGCTGTGATGACCCAAACTCCACTCTCCCTGCCTGTCAGTCTTGGAGATCAAGCCTCCATCTCTTGCAGGTCTAGTCAGAGCCTTGAAAACAGTAATGGAAACACCTATTTGAACTGGTACCTCCAGAAACCAGGCCAGTCTCCACAGCTCCTGATCTACAGGGTTTCCAACCGATTTTCTGGGGTCCTAGACAGGTTCAGTGGCAGTGGTTCAGGGACAGATTTCACACTCAAGATCAGCAGAGTGGAGGCTGAGGATTTGGGAGTTTATTTCTGCCTCCAAGTTACACATGTCCCTCC
IGKV1-122*01_S7970_NOR	NOR/LTJ	IGKV	MUSMUS IGKV1-122*01 F	98.64	GATGCTGTGATGACCCAAACTCCACTCTCCCTGCCTGTCAGTCTTGGAGATCAAGCCTCCATCTCTTGCAGGTCTAGTCAGAGCCTTGAAAACAGTAATGGAAACACCTATTTGAACTGGTACCTCCAGAAACCAGGCCAGTCTCCACAGCTCCTGATCTACAGGGTTTCCAACCGATTTTCTGGGGTCCTAGACAGGTTCAGTGGCAGTGGTTCAGGGACAGATTTCACACTCAAGATCAGCAGAGTGGAGGCTGAGGATTTGGGAGTTTATTTCTGCCTCCAAGTTACACATGTCCCTCC
IGKV1-122*01_S7970_NZB	NZB/BLNJ	IGKV	MUSMUS IGKV1-122*01 F	98.64	GATGCTGTGATGACCCAAACTCCACTCTCCCTGCCTGTCAGTCTTGGAGATCAAGCCTCCATCTCTTGCAGGTCTAGTCAGAGCCTTGAAAACAGTAATGGAAACACCTATTTGAACTGGTACCTCCAGAAACCAGGCCAGTCTCCACAGCTCCTGATCTACAGGGTTTCCAACCGATTTTCTGGGGTCCTAGACAGGTTCAGTGGCAGTGGTTCAGGGACAGATTTCACACTCAAGATCAGCAGAGTGGAGGCTGAGGATTTGGGAGTTTATTTCTGCCTCCAAGTTACACATGTCCCTCC

IGKV1-122*01_S7970_PWD	PWD/PHJ	IGKV	MUSMUS IGKV1-122*01 F	98.64	GATGCTGTGATGACCCAAACTCCACTCTCCCTGCCTGTCAGTCTTGGAGATCAAGCCTCCATCTCTTGCAGGTCTAGTCAGAGCCTTGAAAACAGTAATGGAAACACCTATTTGAACTGGTACCTCCAGAAACCAGGCCAGTCTCCACAGCTCCTGATCTACAGGGTTTCCAACCGATTTTCTGGGGTCCTAGACAGGTTCAGTGGCAGTGGTTCAGGGACAGATTTCACACTCAAGATCAGCAGAGTGGAGGCTGAGGATTTGGGAGTTTATTTCTGCCTCCAAGTTACACATGTCCCTCC
IGKV1-132*01_129	129S1/SVIMJ	IGKV	MUSMUS IGKV1-132*01 F	100	GATGTTGTGATGACCCAGACTCCACTGTCTTTGTCGGTTACCATTGGACAACCAGCCTCTATCTCTTGCAAGTCAAGTCAGAGCCTCTTATATAGTAATGGAAAGACATATTTGAATTGGTTACAACAGAGGCCTGGCCAGGCTCCAAAGCACCTAATGTATCAGGTGTCCAAACTGGACCCTGGCATCCCTGACAGGTTCAGTGGCAGTGGATCAGAAACAGATTTTACACTTAAAATCAGCAGAGTGGAGGCTGAAGATTTGGGAGTTTATTACTGCTTGCAAGGTACATATTATCCTCA
IGKV1-132*01_AJ	A/J	IGKV	MUSMUS IGKV1-132*01 F	100	GATGTTGTGATGACCCAGACTCCACTGTCTTTGTCGGTTACCATTGGACAACCAGCCTCTATCTCTTGCAAGTCAAGTCAGAGCCTCTTATATAGTAATGGAAAGACATATTTGAATTGGTTACAACAGAGGCCTGGCCAGGCTCCAAAGCACCTAATGTATCAGGTGTCCAAACTGGACCCTGGCATCCCTGACAGGTTCAGTGGCAGTGGATCAGAAACAGATTTTACACTTAAAATCAGCAGAGTGGAGGCTGAAGATTTGGGAGTTTATTACTGCTTGCAAGGTACATATTATCCTCA
IGKV1-132*01_AKR	AKR/J	IGKV	MUSMUS IGKV1-132*01 F	100	GATGTTGTGATGACCCAGACTCCACTGTCTTTGTCGGTTACCATTGGACAACCAGCCTCTATCTCTTGCAAGTCAAGTCAGAGCCTCTTATATAGTAATGGAAAGACATATTTGAATTGGTTACAACAGAGGCCTGGCCAGGCTCCAAAGCACCTAATGTATCAGGTGTCCAAACTGGACCCTGGCATCCCTGACAGGTTCAGTGGCAGTGGATCAGAAACAGATTTTACACTTAAAATCAGCAGAGTGGAGGCTGAAGATTTGGGAGTTTATTACTGCTTGCAAGGTACATATTATCCTCA

IGKV1-132*01_BALB	BALB/CBY J	IGKV	MUSMUS IGKV1-132*01 F	100	GATGTTGTGATGACCCAGACTCCACTGTCTTTGTCGGTTACCATTGGACAACCAGCCTCTATCTCTTGCAAGTCAAGTCAGAGCCTCTTATATAGTAATGGAAAGACATATTTGAATTGGTTACAACAGAGGCCTGGCCAGGCTCCAAAGCACCTAATGTATCAGGTGTCCAAACTGGACCCTGGCATCCCTGACAGGTTCAGTGGCAGTGGATCAGAAACAGATTTTACACTTAAAATCAGCAGAGTGGAGGCTGAAGATTTGGGAGTTTATTACTGCTTGCAAGGTACATATTATCCTCA
IGKV1-132*01_C3H	C3H/HEJ	IGKV	MUSMUS IGKV1-132*01 F	100	GATGTTGTGATGACCCAGACTCCACTGTCTTTGTCGGTTACCATTGGACAACCAGCCTCTATCTCTTGCAAGTCAAGTCAGAGCCTCTTATATAGTAATGGAAAGACATATTTGAATTGGTTACAACAGAGGCCTGGCCAGGCTCCAAAGCACCTAATGTATCAGGTGTCCAAACTGGACCCTGGCATCCCTGACAGGTTCAGTGGCAGTGGATCAGAAACAGATTTTACACTTAAAATCAGCAGAGTGGAGGCTGAAGATTTGGGAGTTTATTACTGCTTGCAAGGTACATATTATCCTCA
IGKV1-132*01_DBA1	DBA/1J	IGKV	MUSMUS IGKV1-132*01 F	100	GATGTTGTGATGACCCAGACTCCACTGTCTTTGTCGGTTACCATTGGACAACCAGCCTCTATCTCTTGCAAGTCAAGTCAGAGCCTCTTATATAGTAATGGAAAGACATATTTGAATTGGTTACAACAGAGGCCTGGCCAGGCTCCAAAGCACCTAATGTATCAGGTGTCCAAACTGGACCCTGGCATCCCTGACAGGTTCAGTGGCAGTGGATCAGAAACAGATTTTACACTTAAAATCAGCAGAGTGGAGGCTGAAGATTTGGGAGTTTATTACTGCTTGCAAGGTACATATTATCCTCA
IGKV1-132*01_DBA2	DBA/2J	IGKV	MUSMUS IGKV1-132*01 F	100	GATGTTGTGATGACCCAGACTCCACTGTCTTTGTCGGTTACCATTGGACAACCAGCCTCTATCTCTTGCAAGTCAAGTCAGAGCCTCTTATATAGTAATGGAAAGACATATTTGAATTGGTTACAACAGAGGCCTGGCCAGGCTCCAAAGCACCTAATGTATCAGGTGTCCAAACTGGACCCTGGCATCCCTGACAGGTTCAGTGGCAGTGGATCAGAAACAGATTTTACACTTAAAATCAGCAGAGTGGAGGCTGAAGATTTGGGAGTTTATTACTGCTTGCAAGGTACATATTATCCTCA

IGKV1-132*01_MRL	MRL/MPJ	IGKV	MUSMUS IGKV1-132*01 F	100	GATGTTGTGATGACCCAGACTCCACTGTCTTTGTCGGTTACCATTGGACAACCAGCCTCTATCTCTTGCAAGTCAAGTCAGAGCCTCTTATATAGTAATGGAAAGACATATTTGAATTGGTTACAACAGAGGCCTGGCCAGGCTCCAAAGCACCTAATGTATCAGGTGTCCAAACTGGACCCTGGCATCCCTGACAGGTTCAGTGGCAGTGGATCAGAAACAGATTTTACACTTAAAATCAGCAGAGTGGAGGCTGAAGATTTGGGAGTTTATTACTGCTTGCAAGGTACATATTATCCTCA
IGKV1-132*01_S5178_NOD	NOD/SHILTJ	IGKV	MUSMUS IGKV1-132*01 F	97.96	GATGTTGTGATGACCCAGACTCCACTGTCTTTGTCGGTTACCATTGGACAACCAGCCTCTATCTCTTGCAAGTCAAGTCAGAGCCTCTTATATAGTGATGGAAAGACATATTTGAATTGGTTACAACAGAGGCCAGGCCAGTCTCCAAAGCGCCTAATGTATCAGGTGTCCAAACTGGACCCTGGCATCCCTGACAGGTTCAGTGGCAGTGGATCAGAGACAGATTTTACACTTAAAATCAGCAGAGTGGAGGCTGAGGATTTGGGAGTTTATTACTGCTTGCAAGGTACATATTATCCTCA
IGKV1-132*01_S5178_NOR	NOR/LTJ	IGKV	MUSMUS IGKV1-132*01 F	97.96	GATGTTGTGATGACCCAGACTCCACTGTCTTTGTCGGTTACCATTGGACAACCAGCCTCTATCTCTTGCAAGTCAAGTCAGAGCCTCTTATATAGTGATGGAAAGACATATTTGAATTGGTTACAACAGAGGCCAGGCCAGTCTCCAAAGCGCCTAATGTATCAGGTGTCCAAACTGGACCCTGGCATCCCTGACAGGTTCAGTGGCAGTGGATCAGAGACAGATTTTACACTTAAAATCAGCAGAGTGGAGGCTGAGGATTTGGGAGTTTATTACTGCTTGCAAGGTACATATTATCCTCA
IGKV1-132*01_S5178_NZB	NZB/BLNJ	IGKV	MUSMUS IGKV1-132*01 F	97.96	GATGTTGTGATGACCCAGACTCCACTGTCTTTGTCGGTTACCATTGGACAACCAGCCTCTATCTCTTGCAAGTCAAGTCAGAGCCTCTTATATAGTGATGGAAAGACATATTTGAATTGGTTACAACAGAGGCCAGGCCAGTCTCCAAAGCGCCTAATGTATCAGGTGTCCAAACTGGACCCTGGCATCCCTGACAGGTTCAGTGGCAGTGGATCAGAGACAGATTTTACACTTAAAATCAGCAGAGTGGAGGCTGAGGATTTGGGAGTTTATTACTGCTTGCAAGGTACATATTATCCTCA

IGKV1-<br>133*01_129	129S1/SVI<br>MJ	IGKV	MUSMUS IGKV1-<br>133*01 F	100	GATGTTGTGATGACCCAGACTC<br>CACTCACTTTGTCGGTTACCATT<br>GGACAACCAGCCTCTATCTCTT<br>GCAAGTCAAGTCAGAGCCTCTT<br>ATATAGTAATGGAAAAACCTATTT<br>GAATTGGTTATTACAGAGGCCA<br>GGCCAGTCTCCAAAGCGCCTA<br>ATCTATCTGGTGTCTAAACTGGA<br>CTCTGGAGTCCCTGACAGGTTC<br>ACTGGCAGTGGATCAGGAACAG<br>ATTTTACACTGAAAATCAGCAGA<br>GTGGAGGCTGAGGATTTGGGAG<br>TTTATTACTGCGTGCAAGGTACA<br>CATTTTCCTCA
IGKV1-<br>133*01_AJ	A/J	IGKV	MUSMUS IGKV1-<br>133*01 F	100	GATGTTGTGATGACCCAGACTC<br>CACTCACTTTGTCGGTTACCATT<br>GGACAACCAGCCTCTATCTCTT<br>GCAAGTCAAGTCAGAGCCTCTT<br>ATATAGTAATGGAAAAACCTATTT<br>GAATTGGTTATTACAGAGGCCA<br>GGCCAGTCTCCAAAGCGCCTA<br>ATCTATCTGGTGTCTAAACTGGA<br>CTCTGGAGTCCCTGACAGGTTC<br>ACTGGCAGTGGATCAGGAACAG<br>ATTTTACACTGAAAATCAGCAGA<br>GTGGAGGCTGAGGATTTGGGAG<br>TTTATTACTGCGTGCAAGGTACA<br>CATTTTCCTCA
IGKV1-<br>133*01_AKR	AKR/J	IGKV	MUSMUS IGKV1-<br>133*01 F	100	GATGTTGTGATGACCCAGACTC<br>CACTCACTTTGTCGGTTACCATT<br>GGACAACCAGCCTCTATCTCTT<br>GCAAGTCAAGTCAGAGCCTCTT<br>ATATAGTAATGGAAAAACCTATTT<br>GAATTGGTTATTACAGAGGCCA<br>GGCCAGTCTCCAAAGCGCCTA<br>ATCTATCTGGTGTCTAAACTGGA<br>CTCTGGAGTCCCTGACAGGTTC<br>ACTGGCAGTGGATCAGGAACAG<br>ATTTTACACTGAAAATCAGCAGA<br>GTGGAGGCTGAGGATTTGGGAG<br>TTTATTACTGCGTGCAAGGTACA<br>CATTTTCCTCA
IGKV1-<br>133*01_B6	C57BL/6J	IGKV	MUSMUS IGKV1-<br>133*01 F	100	GATGTTGTGATGACCCAGACTC<br>CACTCACTTTGTCGGTTACCATT<br>GGACAACCAGCCTCTATCTCTT<br>GCAAGTCAAGTCAGAGCCTCTT<br>ATATAGTAATGGAAAAACCTATTT<br>GAATTGGTTATTACAGAGGCCA<br>GGCCAGTCTCCAAAGCGCCTA<br>ATCTATCTGGTGTCTAAACTGGA<br>CTCTGGAGTCCCTGACAGGTTC<br>ACTGGCAGTGGATCAGGAACAG<br>ATTTTACACTGAAAATCAGCAGA<br>GTGGAGGCTGAGGATTTGGGAG<br>TTTATTACTGCGTGCAAGGTACA<br>CATTTTCCTCA

IGKV1-133*01_BALB	BALB/CBYJ	IGKV	MUSMUS IGKV1-133*01 F	100	GATGTTGTGATGACCCAGACTCCACTCACTTTGTCGGTTACCATTGGACAACCAGCCTCTATCTCTTGCAAGTCAAGTCAGAGCCTCTTATATAGTAATGGAAAAACCTATTTGAATTGGTTATTACAGAGGCCAGGCCAGTCTCCAAAGCGCCTAATCTATCTGGTGTCTAAACTGGACTCTGGAGTCCCTGACAGGTTCACTGGCAGTGGATCAGGAACAGATTTTACACTGAAAATCAGCAGAGTGGAGGCTGAGGATTTGGGAGTTTATTACTGCGTGCAAGGTACACATTTTCCTCA
IGKV1-133*01_C3H	C3H/HEJ	IGKV	MUSMUS IGKV1-133*01 F	100	GATGTTGTGATGACCCAGACTCCACTCACTTTGTCGGTTACCATTGGACAACCAGCCTCTATCTCTTGCAAGTCAAGTCAGAGCCTCTTATATAGTAATGGAAAAACCTATTTGAATTGGTTATTACAGAGGCCAGGCCAGTCTCCAAAGCGCCTAATCTATCTGGTGTCTAAACTGGACTCTGGAGTCCCTGACAGGTTCACTGGCAGTGGATCAGGAACAGATTTTACACTGAAAATCAGCAGAGTGGAGGCTGAGGATTTGGGAGTTTATTACTGCGTGCAAGGTACACATTTTCCTCA
IGKV1-133*01_CBA	CBA/J	IGKV	MUSMUS IGKV1-133*01 F	100	GATGTTGTGATGACCCAGACTCCACTCACTTTGTCGGTTACCATTGGACAACCAGCCTCTATCTCTTGCAAGTCAAGTCAGAGCCTCTTATATAGTAATGGAAAAACCTATTTGAATTGGTTATTACAGAGGCCAGGCCAGTCTCCAAAGCGCCTAATCTATCTGGTGTCTAAACTGGACTCTGGAGTCCCTGACAGGTTCACTGGCAGTGGATCAGGAACAGATTTTACACTGAAAATCAGCAGAGTGGAGGCTGAGGATTTGGGAGTTTATTACTGCGTGCAAGGTACACATTTTCCTCA
IGKV1-133*01_DBA1	DBA/1J	IGKV	MUSMUS IGKV1-133*01 F	100	GATGTTGTGATGACCCAGACTCCACTCACTTTGTCGGTTACCATTGGACAACCAGCCTCTATCTCTTGCAAGTCAAGTCAGAGCCTCTTATATAGTAATGGAAAAACCTATTTGAATTGGTTATTACAGAGGCCAGGCCAGTCTCCAAAGCGCCTAATCTATCTGGTGTCTAAACTGGACTCTGGAGTCCCTGACAGGTTCACTGGCAGTGGATCAGGAACAGATTTTACACTGAAAATCAGCAGAGTGGAGGCTGAGGATTTGGGAGTTTATTACTGCGTGCAAGGTACACATTTTCCTCA

IGKV1-133*01_DBA2	DBA/2J	IGKV	MUSMUS IGKV1-133*01 F	100	GATGTTGTGATGACCCAGACTCCACTCACTTTGTCGGTTACCATTGGACAACCAGCCTCTATCTCTTGCAAGTCAAGTCAGAGCCTCTTATATAGTAATGGAAAAACCTATTTGAATTGGTTATTACAGAGGCCAGGCCAGTCTCCAAAGCGCCTAATCTATCTGGTGTCTAAACTGGACTCTGGAGTCCCTGACAGGTTCACTGGCAGTGGATCAGGAACAGATTTTACACTGAAAATCAGCAGAGTGGAGGCTGAGGATTTGGGAGTTTATTACTGCGTGCAAGGTACACATTTTCCTCA
IGKV1-133*01_MRL	MRL/MPJ	IGKV	MUSMUS IGKV1-133*01 F	100	GATGTTGTGATGACCCAGACTCCACTCACTTTGTCGGTTACCATTGGACAACCAGCCTCTATCTCTTGCAAGTCAAGTCAGAGCCTCTTATATAGTAATGGAAAAACCTATTTGAATTGGTTATTACAGAGGCCAGGCCAGTCTCCAAAGCGCCTAATCTATCTGGTGTCTAAACTGGACTCTGGAGTCCCTGACAGGTTCACTGGCAGTGGATCAGGAACAGATTTTACACTGAAAATCAGCAGAGTGGAGGCTGAGGATTTGGGAGTTTATTACTGCGTGCAAGGTACACATTTTCCTCA
IGKV1-133*01_S0950_CAST	CAST/EIJ	IGKV	MUSMUS IGKV1-133*01 F	99.32	GATGTTGTGATGACCCAGACTCCACTCACTTTGTTGGTTACCATTGGACAACCAGCCTCTATCTCTTGCAAGTCAAGTCAGAGCCTCTTATATAGTAATGGAAAAACCTATTTGAATTGGTTATTACAGAGGCCAGGCCAGTCTCCAAAGCGCCTAATCTATCTGGTGTCTAAACTGGACTCTGGAGTCCCTGACAGGTTCACTGGCAGTGGATCAGGAACAGATTTTACACTGAAAATCAGCAGAGTGGAGGCTGAGGATTTGGGAGTTTATTACTGCTTGCAAGGTACACATTTTCCTCA
IGKV1-133*01_S2477_MSM	MSM/MSJ	IGKV	MUSMUS IGKV1-133*01 F	95.58	GATGTTGTGATGACTCAGACCCCACTCACTTTGTCGGTTACCATTGGACAACCAGCCTCCATCTCTTGCAAGTCAAGTCAGAGCCTCTTATATAGTGATGGAAAAACATATTTGGATTGGTTATTACAGAGGCCAGGTCAGTCTCCAAAGAGCCTAATCTATCTGGTGTCTAAACTGGAATCTGGAGTCCCTGACAGGTTCAGTGGCAGTGGATCAGGGACAGATTTCATACTGAAAATCAGCAGAGTGGAGGCTGAGGATTTGGGAGTTTATTACTGCGTGCAAGGTACACATTTTCCTCA

IGKV1-133*01_S2808_MSM	MSM/MSJ	IGKV	MUSMUS IGKV1-133*01 F	99.66	GATGTTGTGATGACCCAGACTC<br>CACTCACTTTGTTGGTTACCATT<br>GGACAACCAGCCTCTATCTCTT<br>GCAAGTCAAGTCAGAGCCTCTT<br>ATATAGTAATGGAAAAACCTATTT<br>GAATTGGTTATTACAGAGGCCA<br>GGCCAGTCTCCAAAGCGCCTA<br>ATCTATCTGGTGTCTAAACTGGA<br>CTCTGGAGTCCCTGACAGGTTC<br>ACTGGCAGTGGATCAGGAACAG<br>ATTTTACACTGAAAATCAGCAGA<br>GTGGAGGCTGAGGATTTGGGAG<br>TTTATTACTGCGTGCAAGGTACA<br>CATTTTCCTCA
IGKV1-133*01_S3070_NOD	NOD/SHILTJ	IGKV	MUSMUS IGKV1-133*01 F	95.92	GATGTTGTGATGACCCAGACTC<br>CACTCACTTTGTCTGTTACCATT<br>GGACAGCCAGCTTCCATTTCTT<br>GCAAGTCAAGTCAGAGCCTCTT<br>ATATAGTGATGGAAAAACCTATTT<br>GAATTGGTTATTACAGAGTCCAG<br>GCCAGTCTCCAAAGCTCCTAAT<br>CTATCTGGTGTCTAAACTGGAAT<br>CTGGAGTCCCTGACAGATTCAG<br>TGGCAGTGGATCAGGGACAGAT<br>TTTACACTGAAAATCAGCAGAGT<br>GGAGGCTGAGGATTTGGGAGTT<br>TATTACTGCGTGCAAGGTACACA<br>TTTCCCTCA
IGKV1-133*01_S3070_NOR	NOR/LTJ	IGKV	MUSMUS IGKV1-133*01 F	95.92	GATGTTGTGATGACCCAGACTC<br>CACTCACTTTGTCTGTTACCATT<br>GGACAGCCAGCTTCCATTTCTT<br>GCAAGTCAAGTCAGAGCCTCTT<br>ATATAGTGATGGAAAAACCTATTT<br>GAATTGGTTATTACAGAGTCCAG<br>GCCAGTCTCCAAAGCTCCTAAT<br>CTATCTGGTGTCTAAACTGGAAT<br>CTGGAGTCCCTGACAGATTCAG<br>TGGCAGTGGATCAGGGACAGAT<br>TTTACACTGAAAATCAGCAGAGT<br>GGAGGCTGAGGATTTGGGAGTT<br>TATTACTGCGTGCAAGGTACACA<br>TTTCCCTCA
IGKV1-133*01_S5139_NOD	NOD/SHILTJ	IGKV	MUSMUS IGKV1-133*01 F	99.32	GATGTTGTGATGACCCAGACTC<br>CACTCACTTTGTCAGTTACCATT<br>GGACAACCAGCCTCTATCTCTT<br>GCAAGTCAAGTCAGAGCCTCTT<br>ATATAGTAATGGAAAAACCTATTT<br>GAACTGGTTATTACAGAGGCCA<br>GGCCAGTCTCCAAAGCGCCTA<br>ATCTATCTGGTGTCTAAACTGGA<br>CTCTGGAGTCCCTGACAGGTTC<br>ACTGGCAGTGGATCAGGAACAG<br>ATTTTACACTGAAAATCAGCAGA<br>GTGGAGGCTGAGGATTTGGGAG<br>TTTATTACTGCGTGCAAGGTACA<br>CATTTTCCTCA

IGKV1-133*01_S5139_NOR	NOR/LTJ	IGKV	MUSMUS IGKV1-133*01 F	99.32	GATGTTGTGATGACCCAGACTC<br>CACTCACTTTGTCAGTTACCATT<br>GGACAACCAGCCTCTATCTCTT<br>GCAAGTCAAGTCAGAGCCTCTT<br>ATATAGTAATGGAAAAACCTATTT<br>GAACTGGTTATTACAGAGGCCA<br>GGCCAGTCTCCAAAGCGCCTA<br>ATCTATCTGGTGTCTAAACTGGA<br>CTCTGGAGTCCCTGACAGGTTC<br>ACTGGCAGTGGATCAGGAACAG<br>ATTTTACACTGAAAATCAGCAGA<br>GTGGAGGCTGAGGATTTGGGAG<br>TTTATTACTGCGTGCAAGGTACA<br>CATTTTCCTCA
IGKV1-133*01_S5139_NZB	NZB/BLNJ	IGKV	MUSMUS IGKV1-133*01 F	99.32	GATGTTGTGATGACCCAGACTC<br>CACTCACTTTGTCAGTTACCATT<br>GGACAACCAGCCTCTATCTCTT<br>GCAAGTCAAGTCAGAGCCTCTT<br>ATATAGTAATGGAAAAACCTATTT<br>GAACTGGTTATTACAGAGGCCA<br>GGCCAGTCTCCAAAGCGCCTA<br>ATCTATCTGGTGTCTAAACTGGA<br>CTCTGGAGTCCCTGACAGGTTC<br>ACTGGCAGTGGATCAGGAACAG<br>ATTTTACACTGAAAATCAGCAGA<br>GTGGAGGCTGAGGATTTGGGAG<br>TTTATTACTGCGTGCAAGGTACA<br>CATTTTCCTCA
IGKV1-133*01_S9974_LEWES	LEWES/EIJ	IGKV	MUSMUS IGKV1-133*01 F	96.26	GATGTTGTGATGACCCAGACTC<br>CACTCACTTTGTCTGTTACCATT<br>GGACAGCCAGCTTCCATCTCTT<br>GCAAGTCAAGTCAGAGCCTCTT<br>ATATAGTGATGGAAAAACCTATTT<br>GAATTGGTTATTACAGAGTCCAG<br>GCCAGTCTCCAAAGCTCCTAAT<br>CTATCTGGTGTCTAAACTGGAAT<br>CTGGAGTCCCTGACAGATTCAG<br>TGGCAGTGGATCAGGGACAGAT<br>TTTACACTGAAAATCAGCAGAGT<br>GGAGGCTGAGGATTTGGGAGTT<br>TATTACTGCGTGCAAGGTACACA<br>TTTCCCTCA
IGKV1-133*01_S9974_MSM	MSM/MSJ	IGKV	MUSMUS IGKV1-133*01 F	96.26	GATGTTGTGATGACCCAGACTC<br>CACTCACTTTGTCTGTTACCATT<br>GGACAGCCAGCTTCCATCTCTT<br>GCAAGTCAAGTCAGAGCCTCTT<br>ATATAGTGATGGAAAAACCTATTT<br>GAATTGGTTATTACAGAGTCCAG<br>GCCAGTCTCCAAAGCTCCTAAT<br>CTATCTGGTGTCTAAACTGGAAT<br>CTGGAGTCCCTGACAGATTCAG<br>TGGCAGTGGATCAGGGACAGAT<br>TTTACACTGAAAATCAGCAGAGT<br>GGAGGCTGAGGATTTGGGAGTT<br>TATTACTGCGTGCAAGGTACACA<br>TTTCCCTCA

IGKV1-133*01_S9974_PWD	PWD/PHJ	IGKV	MUSMUS IGKV1-133*01 F	96.26	GATGTTGTGATGACCCAGACTC<br>CACTCACTTTGTCTGTTACCATT<br>GGACAGCCAGCTTCCATCTCTT<br>GCAAGTCAAGTCAGAGCCTCTT<br>ATATAGTGATGGAAAAACCTATTT<br>GAATTGGTTATTACAGAGTCCAG<br>GCCAGTCTCCAAAGCTCCTAAT<br>CTATCTGGTGTCTAAACTGGAAT<br>CTGGAGTCCCTGACAGATTCAG<br>TGGCAGTGGATCAGGGACAGAT<br>TTTACACTGAAAATCAGCAGAGT<br>GGAGGCTGAGGATTTGGGAGTT<br>TATTACTGCGTGCAAGGTACACA<br>TTTCCCTCA
IGKV1-133*01_SJL	SJL/J	IGKV	MUSMUS IGKV1-133*01 F	100	GATGTTGTGATGACCCAGACTC<br>CACTCACTTTGTCGGTTACCATT<br>GGACAACCAGCCTCTATCTCTT<br>GCAAGTCAAGTCAGAGCCTCTT<br>ATATAGTAATGGAAAAACCTATTT<br>GAATTGGTTATTACAGAGGCCA<br>GGCCAGTCTCCAAAGCGCCTA<br>ATCTATCTGGTGTCTAAACTGGA<br>CTCTGGAGTCCCTGACAGGTTC<br>ACTGGCAGTGGATCAGGAACAG<br>ATTTTACACTGAAAATCAGCAGA<br>GTGGAGGCTGAGGATTTGGGAG<br>TTTATTACTGCGTGCAAGGTACA<br>CATTTTCCTCA
IGKV1-135*01_129	129S1/SVIMJ	IGKV	MUSMUS IGKV1-135*01 F	100	GATGTTGTGATGACCCAGACTC<br>CACTCACTTTGTCGGTTACCATT<br>GGACAACCAGCCTCCATCTCTT<br>GCAAGTCAAGTCAGAGCCTCTT<br>AGATAGTGATGGAAAGACATATT<br>TGAATTGGTTGTTACAGAGGCCA<br>GGCCAGTCTCCAAAGCGCCTA<br>ATCTATCTGGTGTCTAAACTGGA<br>CTCTGGAGTCCCTGACAGGTTC<br>ACTGGCAGTGGATCAGGGACA<br>GATTTCACACTGAAAATCAGCAG<br>AGTGGAGGCTGAGGATTTGGGA<br>GTTTATTATTGCTGGCAAGGTAC<br>ACATTTTCCTCA
IGKV1-135*01_AJ	A/J	IGKV	MUSMUS IGKV1-135*01 F	100	GATGTTGTGATGACCCAGACTC<br>CACTCACTTTGTCGGTTACCATT<br>GGACAACCAGCCTCCATCTCTT<br>GCAAGTCAAGTCAGAGCCTCTT<br>AGATAGTGATGGAAAGACATATT<br>TGAATTGGTTGTTACAGAGGCCA<br>GGCCAGTCTCCAAAGCGCCTA<br>ATCTATCTGGTGTCTAAACTGGA<br>CTCTGGAGTCCCTGACAGGTTC<br>ACTGGCAGTGGATCAGGGACA<br>GATTTCACACTGAAAATCAGCAG<br>AGTGGAGGCTGAGGATTTGGGA<br>GTTTATTATTGCTGGCAAGGTAC<br>ACATTTTCCTCA

IGKV1-135*01_AKR	AKR/J	IGKV	MUSMUS IGKV1-135*01 F	100	GATGTTGTGATGACCCAGACTCCACTCACTTTGTCGGTTACCATTGGACAACCAGCCTCCATCTCTTGCAAGTCAAGTCAGAGCCTCTTAGATAGTGATGGAAAGACATATTTGAATTGGTTGTTACAGAGGCCAGGCCAGTCTCCAAAGCGCCTAATCTATCTGGTGTCTAAACTGGACTCTGGAGTCCCTGACAGGTTCACTGGCAGTGGATCAGGGACAGATTTCACACTGAAAATCAGCAGAGTGGAGGCTGAGGATTTGGGAGTTTATTATTGCTGGCAAGGTACACATTTTCCTCA
IGKV1-135*01_B6	C57BL/6J	IGKV	MUSMUS IGKV1-135*01 F	100	GATGTTGTGATGACCCAGACTCCACTCACTTTGTCGGTTACCATTGGACAACCAGCCTCCATCTCTTGCAAGTCAAGTCAGAGCCTCTTAGATAGTGATGGAAAGACATATTTGAATTGGTTGTTACAGAGGCCAGGCCAGTCTCCAAAGCGCCTAATCTATCTGGTGTCTAAACTGGACTCTGGAGTCCCTGACAGGTTCACTGGCAGTGGATCAGGGACAGATTTCACACTGAAAATCAGCAGAGTGGAGGCTGAGGATTTGGGAGTTTATTATTGCTGGCAAGGTACACATTTTCCTCA
IGKV1-135*01_BALB	BALB/CBYJ	IGKV	MUSMUS IGKV1-135*01 F	100	GATGTTGTGATGACCCAGACTCCACTCACTTTGTCGGTTACCATTGGACAACCAGCCTCCATCTCTTGCAAGTCAAGTCAGAGCCTCTTAGATAGTGATGGAAAGACATATTTGAATTGGTTGTTACAGAGGCCAGGCCAGTCTCCAAAGCGCCTAATCTATCTGGTGTCTAAACTGGACTCTGGAGTCCCTGACAGGTTCACTGGCAGTGGATCAGGGACAGATTTCACACTGAAAATCAGCAGAGTGGAGGCTGAGGATTTGGGAGTTTATTATTGCTGGCAAGGTACACATTTTCCTCA
IGKV1-135*01_C3H	C3H/HEJ	IGKV	MUSMUS IGKV1-135*01 F	100	GATGTTGTGATGACCCAGACTCCACTCACTTTGTCGGTTACCATTGGACAACCAGCCTCCATCTCTTGCAAGTCAAGTCAGAGCCTCTTAGATAGTGATGGAAAGACATATTTGAATTGGTTGTTACAGAGGCCAGGCCAGTCTCCAAAGCGCCTAATCTATCTGGTGTCTAAACTGGACTCTGGAGTCCCTGACAGGTTCACTGGCAGTGGATCAGGGACAGATTTCACACTGAAAATCAGCAGAGTGGAGGCTGAGGATTTGGGAGTTTATTATTGCTGGCAAGGTACACATTTTCCTCA

IGKV1-135*01_CBA	CBA/J	IGKV	MUSMUS IGKV1-135*01 F	100	GATGTTGTGATGACCCAGACTCCACTCACTTTGTCGGTTACCATTGGACAACCAGCCTCCATCTCTTGCAAGTCAAGTCAGAGCCTCTTAGATAGTGATGGAAAGACATATTTGAATTGGTTGTTACAGAGGCCAGGCCAGTCTCCAAAGCGCCTAATCTATCTGGTGTCTAAACTGGACTCTGGAGTCCCTGACAGGTTCACTGGCAGTGGATCAGGGACAGATTTCACACTGAAAATCAGCAGAGTGGAGGCTGAGGATTTGGGAGTTTATTATTGCTGGCAAGGTACACATTTTCCTCA
IGKV1-135*01_DBA1	DBA/1J	IGKV	MUSMUS IGKV1-135*01 F	100	GATGTTGTGATGACCCAGACTCCACTCACTTTGTCGGTTACCATTGGACAACCAGCCTCCATCTCTTGCAAGTCAAGTCAGAGCCTCTTAGATAGTGATGGAAAGACATATTTGAATTGGTTGTTACAGAGGCCAGGCCAGTCTCCAAAGCGCCTAATCTATCTGGTGTCTAAACTGGACTCTGGAGTCCCTGACAGGTTCACTGGCAGTGGATCAGGGACAGATTTCACACTGAAAATCAGCAGAGTGGAGGCTGAGGATTTGGGAGTTTATTATTGCTGGCAAGGTACACATTTTCCTCA
IGKV1-135*01_DBA2	DBA/2J	IGKV	MUSMUS IGKV1-135*01 F	100	GATGTTGTGATGACCCAGACTCCACTCACTTTGTCGGTTACCATTGGACAACCAGCCTCCATCTCTTGCAAGTCAAGTCAGAGCCTCTTAGATAGTGATGGAAAGACATATTTGAATTGGTTGTTACAGAGGCCAGGCCAGTCTCCAAAGCGCCTAATCTATCTGGTGTCTAAACTGGACTCTGGAGTCCCTGACAGGTTCACTGGCAGTGGATCAGGGACAGATTTCACACTGAAAATCAGCAGAGTGGAGGCTGAGGATTTGGGAGTTTATTATTGCTGGCAAGGTACACATTTTCCTCA
IGKV1-135*01_LEWES	LEWES/EIJ	IGKV	MUSMUS IGKV1-135*01 F	100	GATGTTGTGATGACCCAGACTCCACTCACTTTGTCGGTTACCATTGGACAACCAGCCTCCATCTCTTGCAAGTCAAGTCAGAGCCTCTTAGATAGTGATGGAAAGACATATTTGAATTGGTTGTTACAGAGGCCAGGCCAGTCTCCAAAGCGCCTAATCTATCTGGTGTCTAAACTGGACTCTGGAGTCCCTGACAGGTTCACTGGCAGTGGATCAGGGACAGATTTCACACTGAAAATCAGCAGAGTGGAGGCTGAGGATTTGGGAGTTTATTATTGCTGGCAAGGTACACATTTTCCTCA

IGKV1-135*01_MRL	MRL/MPJ	IGKV	MUSMUS IGKV1-135*01 F	100	GATGTTGTGATGACCCAGACTCCACTCACTTTGTCGGTTACCATTGGACAACCAGCCTCCATCTCTTGCAAGTCAAGTCAGAGCCTCTTAGATAGTGATGGAAAGACATATTTGAATTGGTTGTTACAGAGGCCAGGCCAGTCTCCAAAGCGCCTAATCTATCTGGTGTCTAAACTGGACTCTGGAGTCCCTGACAGGTTCACTGGCAGTGGATCAGGGACAGATTTCACACTGAAAATCAGCAGAGTGGAGGCTGAGGATTTGGGAGTTTATTATTGCTGGCAAGGTACACATTTTCCTCA
IGKV1-135*01_S0376_PWD	PWD/PHJ	IGKV	MUSMUS IGKV1-135*01 F	95.92	GATGTTGTGATGACTCAGACCCCACTCACTTTGTCGGTTACCATTGGACAACCAGCTTCCATCTCTTGCAAGTCAAGTCAGAGCCTCTTACATAGTAATGGAAAGACATATTTGAATTGGTTATTACAGAGGCCAGGCCAGTCTCCAAAGCTCCTAATCTATCTGGTGTCTAAACTGGAATCTGGAGTCCCTGACAGGTTCAGTGGCAGTGGATCAGGGACAGATTTCACACTGAAAATCAGCAGAGTGGAGGCTGAGGATTTGGGAGTTTATTACTGCTTGCAAGCTACACATTTTCCTCA
IGKV1-135*01_S1357_LEWES	LEWES/EIJ	IGKV	MUSMUS IGKV1-135*01 F	95.92	GATGTTGTGATGACTCAGACCCCACTCACTTTGTCGGTTACCATTGGACAACCAGCCTCCATCTCTTGCAAGTCAAGTCAGAGCCTCTTATATAGTGATGGAAAGACATATTTGGATTGGTTATTACAGAGGCCAGGTCAGTCTCCAAAGAGCCTAATCTATCTGGTGTCTAAACTGGAATCTGGAGTCCCTGACAGGTTCAGTGGCAGTGGATCAGGGACAGATTTCACACTGAAAATCAGCAGAGTGGAGGCTGAGGATTTGGGAGTTTATTACTGCTTGCAAGCTACACATTTTCCTCA
IGKV1-135*01_S3001_NOD	NOD/SHILTJ	IGKV	MUSMUS IGKV1-135*01 F	95.92	GATGTTGTGATGACTCAGACCCCACTCACTTTGTCGGTTACCATTGGACAACCAGCCTCCATCTCTTGCAAATCAAGTCAGAGCCTCTTACATAGTAATGGAAAGACATATTTGAATTGGTTATTACAGAGGCCAGGCCAGTCTCCAAAGCTCCTAATCTATCTGGTGTCTAAACTGGAATCTGGAGTCCCTGACAGGTTCAGTGGCAGTGGATCAGGGACAGATTTCACACTGAAAATCAGCAGAGTGGAGGCTGAGGATTTGGGAGTTTATTACTGCTTGCAAGCTACACATTTTCCTCA

IGKV1-135*01_S3001_NOR	NOR/LTJ	IGKV	MUSMUS IGKV1-135*01 F	95.92	GATGTTGTGATGACTCAGACCCCACTCACTTTGTCGGTTACCATTGGACAACCAGCCTCCATCTCTTGCAAATCAAGTCAGAGCCTCTTACATAGTAATGGAAAGACATATTTGAATTGGTTATTACAGAGGCCAGGCCAGTCTCCAAAGCTCCTAATCTATCTGGTGTCTAAACTGGAATCTGGAGTCCCTGACAGGTTCAGTGGCAGTGGATCAGGGACAGATTTCACACTGAAAATCAGCAGAGTGGAGGCTGAGGATTTGGGAGTTTATTACTGCTTGCAAGCTACACATTTTCCTCA
IGKV1-135*01_S3001A_NZB	NZB/BLNJ	IGKV	MUSMUS IGKV1-135*01 F	95.92	GATGTTGTGATGACTCAGACCCCACTCACTTTGTCGGTTACCATTGGACAACCAGCCTCCATCTCTTGCAAATCAAGTCAGAGCCTCTTACATAGTAATGGAAAGACATATTTGAATTGGTTATTACAGAGGCCAGGCCAGTCTCCAAAGCTCCTAATCTATCTGGTGTCTAAACTGGAATCTGGAGTCCCTGACAGGTTCAGTGGCAGTGGATCAGGGACAGATTTCACACTGAAAATCAGCAGAGTGGAGGCTGAGGATTTGGGAGTTTATTACTGCTTGCAAGCTACACATTTTCCTCA
IGKV1-135*01_S6159_LEWES	LEWES/EIJ	IGKV	MUSMUS IGKV1-135*01 F	95.58	GATGTTGTGATGACTCAGACCCCACTCACTTTGTCGGTTACCATTGGACAACCAGCCTCCATCTCTTGCAAGTCAAGTCAGAGCCTCTTATATAGTGATGGAAAGACATATTTGGATTGGTTATTACAGAGGCCAGGTCAGTCTCCAAAGAGCCTAATCTATCTGGTGTCTAAACTGGAATCTGGAGTCCCTGACAGGTTCAGTGGCAGTGGATCAGGGACAGATTTCATACTGAAAATCAGCAGAGTGGAGGCTGAGGATTTGGGAGTTTATTACTGCGTGCAAGGTACACATTTTCCTC
IGKV1-135*01_S6159_PWD	PWD/PHJ	IGKV	MUSMUS IGKV1-135*01 F	95.58	GATGTTGTGATGACTCAGACCCCACTCACTTTGTCGGTTACCATTGGACAACCAGCCTCCATCTCTTGCAAGTCAAGTCAGAGCCTCTTATATAGTGATGGAAAGACATATTTGGATTGGTTATTACAGAGGCCAGGTCAGTCTCCAAAGAGCCTAATCTATCTGGTGTCTAAACTGGAATCTGGAGTCCCTGACAGGTTCAGTGGCAGTGGATCAGGGACAGATTTCATACTGAAAATCAGCAGAGTGGAGGCTGAGGATTTGGGAGTTTATTACTGCGTGCAAGGTACACATTTTCCTC

IGKV1-135*01_S7405_NOD	NOD/SHILTJ	IGKV	MUSMUS IGKV1-135*01 F	95.24	GATGTTGTGATGACCCAGACTCCACTGTCTTTGTCGGTTACCATTGGACAACCAGCCTCCATCTCTTGCAAGTCAAGTCAGAGCCTCTTACATAGTGATGGAAAGACATATTTGAATTGGTTATTACAGAGGCCAGGCCAGTCTCCAAAGCTCCTAATCTATCTGGTGTCTAAACTGGAATCTGGCATCCCTGACAGGTTCAGTGGCAGTGGATCAGGGACAGATTTCACACTGAAAATCAGCAGAGTGGAGGTTGAGGATTTGGGAGTTTATTACTGCTTGCAACATACACATTTTCCTCA
IGKV1-135*01_S7405_NOR	NOR/LTJ	IGKV	MUSMUS IGKV1-135*01 F	95.24	GATGTTGTGATGACCCAGACTCCACTGTCTTTGTCGGTTACCATTGGACAACCAGCCTCCATCTCTTGCAAGTCAAGTCAGAGCCTCTTACATAGTGATGGAAAGACATATTTGAATTGGTTATTACAGAGGCCAGGCCAGTCTCCAAAGCTCCTAATCTATCTGGTGTCTAAACTGGAATCTGGCATCCCTGACAGGTTCAGTGGCAGTGGATCAGGGACAGATTTCACACTGAAAATCAGCAGAGTGGAGGTTGAGGATTTGGGAGTTTATTACTGCTTGCAACATACACATTTTCCTCA
IGKV1-135*01_S7405_NZB	NZB/BLNJ	IGKV	MUSMUS IGKV1-135*01 F	95.24	GATGTTGTGATGACCCAGACTCCACTGTCTTTGTCGGTTACCATTGGACAACCAGCCTCCATCTCTTGCAAGTCAAGTCAGAGCCTCTTACATAGTGATGGAAAGACATATTTGAATTGGTTATTACAGAGGCCAGGCCAGTCTCCAAAGCTCCTAATCTATCTGGTGTCTAAACTGGAATCTGGCATCCCTGACAGGTTCAGTGGCAGTGGATCAGGGACAGATTTCACACTGAAAATCAGCAGAGTGGAGGTTGAGGATTTGGGAGTTTATTACTGCTTGCAACATACACATTTTCCTCA
IGKV1-135*01_S8190_MSM	MSM/MSJ	IGKV	MUSMUS IGKV1-135*01 F	96.26	GATGTTGTGATGACTCAGACCCCACTCACTTTGTCGGTTACCATTGGACAACCAGCCTCCATCTCTTGCAAGTCAAGTCAGAGCCTCTTACATAGTAATGGAAAGACATATTTGAATTGGTTATTACAGAGGCCAGGCCAGTCTCCAAAGCTCCTAATCTATCTGGTGTCTAAACTGGAATCTGGAGTCCCTGACAGGTTCAGTGGCAGTGGATCAGGGACAGATTTCACACTGAAAATCAGCAGAGTGGAGGCTGAGGATTTGGGAGTTTATTACTGCTTGCAAGCTACACATTTTCCTCA

IGKV1-135*01_S9254_CAST	CAST/EIJ	IGKV	MUSMUS IGKV1-135*01 F	95.58	GATGTTGTGATGACTCAGACCCCAATCACTATGTCGGTTACCATTGGACAACCAGCCTCCATCTCTTGCAAGTCAAGTCAGAGCCTCTTACATAGTAATGGAAAGACATATTTGAATTGGTTATTACAGAGGCCAGGCCAGTCTCCAAAGCTCCTAATCTATCTGGTGTCTAAACTGGAATCTGGAGTCCCTGACAGGTTCAGTGGCAGTGGATCAGGGACAGATTTCACACTGAAAATCAGCAGAGTGGAGGCTGAGGATTTGGGAGTTTATTACTGCTTGCAAGGTACATATTATCCTCA
IGKV1-135*01_SJL	SJL/J	IGKV	MUSMUS IGKV1-135*01 F	100	GATGTTGTGATGACCCAGACTCCACTCACTTTGTCGGTTACCATTGGACAACCAGCCTCCATCTCTTGCAAGTCAAGTCAGAGCCTCTTAGATAGTGATGGAAAGACATATTTGAATTGGTTGTTACAGAGGCCAGGCCAGTCTCCAAAGCGCCTAATCTATCTGGTGTCTAAACTGGACTCTGGAGTCCCTGACAGGTTCACTGGCAGTGGATCAGGGACAGATTTCACACTGAAAATCAGCAGAGTGGAGGCTGAGGATTTGGGAGTTTATTATTGCTGGCAAGGTACACATTTTCCTCA
IGKV1-35*01_S0416_AKR	AKR/J	IGKV	MUSMUS IGKV1-35*01 ORF	97.28	GACATTGTGATGACCCAGACTCCACTCACTTTATCAGCTACCATTGGACAATCAGCCTCCATCTCTTGCAGGTCAAGTCAGAGTCTCTTACATAGTAATGGAAACACATACTTGAATTGGTTTCTACAGAGGCCAGGCCAATCTCCACAGCTTCTGATTTATGGGGTGTTTGAACGGGAATCTGGGGTTCCTGACAGGTTCAGTGGCAGTGGGTCAGGAACAGATTTCACACTCAAGATCAGCAGAGTGGAGGCTGAGGATTTGGGAGTTTATTACTGCATGCAAGCTACCTATGAACCTC
IGKV1-35*01_S0416_MSM	MSM/MSJ	IGKV	MUSMUS IGKV1-35*01 ORF	97.28	GACATTGTGATGACCCAGACTCCACTCACTTTATCAGCTACCATTGGACAATCAGCCTCCATCTCTTGCAGGTCAAGTCAGAGTCTCTTACATAGTAATGGAAACACATACTTGAATTGGTTTCTACAGAGGCCAGGCCAATCTCCACAGCTTCTGATTTATGGGGTGTTTGAACGGGAATCTGGGGTTCCTGACAGGTTCAGTGGCAGTGGGTCAGGAACAGATTTCACACTCAAGATCAGCAGAGTGGAGGCTGAGGATTTGGGAGTTTATTACTGCATGCAAGCTACCTATGAACCTC

IGKV1-35*01_S2930_CAST	CAST/EIJ	IGKV	MUSMUS IGKV1-35*01 ORF	96.94	GACATTGTGATGACCCAGACTCCACTCACTTTATCAGCTACCATTGGACAATCAGCCTCCATCTCTTGCAGGTCAAGTCAGAGTCTCTTACATAGTAATGGAAACACATACTTGAATTGGTTTCTACAGAGGCCAGGCCAATCTCCACAGCTTCTGATTTATGGGGTGTTTGAACGGGAATCTGGGGTTCCTGACAGGTTCAGTGGCAGTGGGTCAGGAACAGATTTCACACTCAAGATCAGTAGAGTGGAGGCTGAGGATTTGGGAGTTTATTACTGCATGCAAGCTACCTATGAACCTCC
IGKV1-88*01_129	129S1/SVIMJ	IGKV	MUSMUS IGKV1-88*01 F	100	GATGTTGTGGTGACTCAAACTCCACTCTCCCTGCCTGTCAGCTTTGGAGATCAAGTTTCTATCTCTTGCAGGTCTAGTCAGAGTCTTGCAAACAGTTATGGGAACACCTATTTGTCTTGGTACCTGCACAAGCCTGGCCAGTCTCCACAGCTCCTCATCTATGGGATTTCCAACAGATTTTCTGGGGTGCCAGACAGGTTCAGTGGCAGTGGTTCAGGGACAGATTTCACACTCAAGATCAGCACAATAAAGCCTGAGGACTTGGGAATGTATTACTGCTTACAAGGTACACATCAGCCTCC
IGKV1-88*01_AJ	A/J	IGKV	MUSMUS IGKV1-88*01 F	100	GATGTTGTGGTGACTCAAACTCCACTCTCCCTGCCTGTCAGCTTTGGAGATCAAGTTTCTATCTCTTGCAGGTCTAGTCAGAGTCTTGCAAACAGTTATGGGAACACCTATTTGTCTTGGTACCTGCACAAGCCTGGCCAGTCTCCACAGCTCCTCATCTATGGGATTTCCAACAGATTTTCTGGGGTGCCAGACAGGTTCAGTGGCAGTGGTTCAGGGACAGATTTCACACTCAAGATCAGCACAATAAAGCCTGAGGACTTGGGAATGTATTACTGCTTACAAGGTACACATCAGCCTCC
IGKV1-88*01_B6	C57BL/6J	IGKV	MUSMUS IGKV1-88*01 F	100	GATGTTGTGGTGACTCAAACTCCACTCTCCCTGCCTGTCAGCTTTGGAGATCAAGTTTCTATCTCTTGCAGGTCTAGTCAGAGTCTTGCAAACAGTTATGGGAACACCTATTTGTCTTGGTACCTGCACAAGCCTGGCCAGTCTCCACAGCTCCTCATCTATGGGATTTCCAACAGATTTTCTGGGGTGCCAGACAGGTTCAGTGGCAGTGGTTCAGGGACAGATTTCACACTCAAGATCAGCACAATAAAGCCTGAGGACTTGGGAATGTATTACTGCTTACAAGGTACACATCAGCCTCC

IGKV1-88*01_BALB	BALB/CBYJ	IGKV	MUSMUS IGKV1-88*01 F	100	GATGTTGTGGTGACTCAAACTC<br>CACTCTCCCTGCCTGTCAGCTT<br>TGGAGATCAAGTTTCTATCTCTT<br>GCAGGTCTAGTCAGAGTCTTGC<br>AAACAGTTATGGGAACACCTATT<br>TGTCTTGGTACCTGCACAAGCC<br>TGGCCAGTCTCCACAGCTCCTC<br>ATCTATGGGATTTCCAACAGATT<br>TTCTGGGGTGCCAGACAGGTTC<br>AGTGGCAGTGGTTCAGGGACA<br>GATTTCACACTCAAGATCAGCA<br>CAATAAAGCCTGAGGACTTGGG<br>AATGTATTACTGCTTACAAGGTA<br>CACATCAGCCTCC
IGKV1-88*01_C3H	C3H/HEJ	IGKV	MUSMUS IGKV1-88*01 F	100	GATGTTGTGGTGACTCAAACTC<br>CACTCTCCCTGCCTGTCAGCTT<br>TGGAGATCAAGTTTCTATCTCTT<br>GCAGGTCTAGTCAGAGTCTTGC<br>AAACAGTTATGGGAACACCTATT<br>TGTCTTGGTACCTGCACAAGCC<br>TGGCCAGTCTCCACAGCTCCTC<br>ATCTATGGGATTTCCAACAGATT<br>TTCTGGGGTGCCAGACAGGTTC<br>AGTGGCAGTGGTTCAGGGACA<br>GATTTCACACTCAAGATCAGCA<br>CAATAAAGCCTGAGGACTTGGG<br>AATGTATTACTGCTTACAAGGTA<br>CACATCAGCCTCC
IGKV1-88*01_DBA1	DBA/1J	IGKV	MUSMUS IGKV1-88*01 F	100	GATGTTGTGGTGACTCAAACTC<br>CACTCTCCCTGCCTGTCAGCTT<br>TGGAGATCAAGTTTCTATCTCTT<br>GCAGGTCTAGTCAGAGTCTTGC<br>AAACAGTTATGGGAACACCTATT<br>TGTCTTGGTACCTGCACAAGCC<br>TGGCCAGTCTCCACAGCTCCTC<br>ATCTATGGGATTTCCAACAGATT<br>TTCTGGGGTGCCAGACAGGTTC<br>AGTGGCAGTGGTTCAGGGACA<br>GATTTCACACTCAAGATCAGCA<br>CAATAAAGCCTGAGGACTTGGG<br>AATGTATTACTGCTTACAAGGTA<br>CACATCAGCCTCC
IGKV1-88*01_DBA2	DBA/2J	IGKV	MUSMUS IGKV1-88*01 F	100	GATGTTGTGGTGACTCAAACTC<br>CACTCTCCCTGCCTGTCAGCTT<br>TGGAGATCAAGTTTCTATCTCTT<br>GCAGGTCTAGTCAGAGTCTTGC<br>AAACAGTTATGGGAACACCTATT<br>TGTCTTGGTACCTGCACAAGCC<br>TGGCCAGTCTCCACAGCTCCTC<br>ATCTATGGGATTTCCAACAGATT<br>TTCTGGGGTGCCAGACAGGTTC<br>AGTGGCAGTGGTTCAGGGACA<br>GATTTCACACTCAAGATCAGCA<br>CAATAAAGCCTGAGGACTTGGG<br>AATGTATTACTGCTTACAAGGTA<br>CACATCAGCCTCC

IGKV1-88*01_LEWES	LEWES/EIJ	IGKV	MUSMUS IGKV1-88*01 F	100	GATGTTGTGGTGACTCAAACTC CACTCTCCCTGCCTGTCAGCTT TGGAGATCAAGTTTCTATCTCTT GCAGGTCTAGTCAGAGTCTTGC AAACAGTTATGGGAACACCTATT TGTCTTGGTACCTGCACAAGCC TGGCCAGTCTCCACAGCTCCTC ATCTATGGGATTTCCAACAGATT TTCTGGGGTGCCAGACAGGTTC AGTGGCAGTGGTTCAGGGACA GATTTCACACTCAAGATCAGCA CAATAAAGCCTGAGGACTTGGG AATGTATTACTGCTTACAAGGTA CACATCAGCCTCC
IGKV1-88*01_NZB	NZB/BLNJ	IGKV	MUSMUS IGKV1-88*01 F	100	GATGTTGTGGTGACTCAAACTC CACTCTCCCTGCCTGTCAGCTT TGGAGATCAAGTTTCTATCTCTT GCAGGTCTAGTCAGAGTCTTGC AAACAGTTATGGGAACACCTATT TGTCTTGGTACCTGCACAAGCC TGGCCAGTCTCCACAGCTCCTC ATCTATGGGATTTCCAACAGATT TTCTGGGGTGCCAGACAGGTTC AGTGGCAGTGGTTCAGGGACA GATTTCACACTCAAGATCAGCA CAATAAAGCCTGAGGACTTGGG AATGTATTACTGCTTACAAGGTA CACATCAGCCTCC
IGKV1-88*01_S0716_AKR	AKR/J	IGKV	MUSMUS IGKV1-88*01 F	99.66	GATGTTGTGGTGACTCAAACTC CACTCTCCCTGCCTGTCAGCTT TGGAGATCAAGTTTCTATCTCTT GCAGGTCTAGTCAGAGTCTTGC AAACAGTTATGGGAACACCTATT TGTCTTGGTACCTGCACAAGCC TGGCCAGTCTCCACAGCTCCTC ATCTATGGGATTTCCAACAGATT TTCCGGGGTGCCAGACAGGTTC AGTGGCAGTGGTTCAGGGACA GATTTCACACTCAAGATCAGCA CAATAAAGCCTGAGGACTTGGG AATGTATTACTGCTTACAAGGTA CACATCAGCCTCC
IGKV1-88*01_S0716_MRL	MRL/MPJ	IGKV	MUSMUS IGKV1-88*01 F	99.66	GATGTTGTGGTGACTCAAACTC CACTCTCCCTGCCTGTCAGCTT TGGAGATCAAGTTTCTATCTCTT GCAGGTCTAGTCAGAGTCTTGC AAACAGTTATGGGAACACCTATT TGTCTTGGTACCTGCACAAGCC TGGCCAGTCTCCACAGCTCCTC ATCTATGGGATTTCCAACAGATT TTCCGGGGTGCCAGACAGGTTC AGTGGCAGTGGTTCAGGGACA GATTTCACACTCAAGATCAGCA CAATAAAGCCTGAGGACTTGGG AATGTATTACTGCTTACAAGGTA CACATCAGCCTCC

IGKV1-88*01_S0716_NOD	NOD/SHILTJ	IGKV	MUSMUS IGKV1-88*01 F	99.66	GATGTTGTGGTGACTCAAACTCCACTCTCCCTGCCTGTCAGCTTTGGAGATCAAGTTTCTATCTCTTGCAGGTCTAGTCAGAGTCTTGCAAACAGTTATGGGAACACCTATTTGTCTTGGTACCTGCACAAGCCTGGCCAGTCTCCACAGCTCCTCATCTATGGGATTTCCAACAGATTTTCCGGGGTGCCAGACAGGTTCAGTGGCAGTGGTTCAGGGACAGATTTCACACTCAAGATCAGCACAATAAAGCCTGAGGACTTGGGAATGTATTACTGCTTACAAGGTACACATCAGCCTCC
IGKV1-88*01_S1851_CAST	CAST/EIJ	IGKV	MUSMUS IGKV1-88*01 F	98.3	GATGTTGTGGTGACCCAAACTCCACTCTCCCTGCCTGTCAGCTTTGGAGATCAGGTTTCTATCTCTTGCAGGTCTAGTCAGAGTCTTGCAAACAGTTATGGGAACACCTATTTGTCTTGGTACCTGCACAAGCCTGGCCAGTCTCCTCAGCTCCTCATCTATGGGATTTTCAACAGATTTTCCGGGGTGCCAGACAGGTTCAGTGGCAGTGGTTCAGGGACAGATTTCACACTCAAGATCAGCACAATAAAGCCTGAGGACTTGGGAATGTATTACTGCTTACAAGGTACACATCAGCC
IGKV1-99*01_129	129S1/SVIMJ	IGKV	MUSMUS IGKV1-99*01 F	100	GATGTTGTTCTGACCCAAACTCCACTCTCTCTGCCTGTCAATATTGGAGATCAAGCCTCTATCTCTTGCAAGTCTACTAAGAGTCTTCTGAATAGTGATGGATTCACTTATTTGGACTGGTACCTGCAGAAGCCAGGCCAGTCTCCACAGCTCCTAATATATTTGGTTTCTAATCGATTTTCTGGAGTTCCAGACAGGTTCAGTGGCAGTGGGTCAGGAACAGATTTCACACTCAAGATCAGCAGAGTGGAGGCTGAGGATTTGGGAGTTTATTATTGCTTCCAGAGTAACTATCTTCCTCT
IGKV1-99*01_AJ	A/J	IGKV	MUSMUS IGKV1-99*01 F	100	GATGTTGTTCTGACCCAAACTCCACTCTCTCTGCCTGTCAATATTGGAGATCAAGCCTCTATCTCTTGCAAGTCTACTAAGAGTCTTCTGAATAGTGATGGATTCACTTATTTGGACTGGTACCTGCAGAAGCCAGGCCAGTCTCCACAGCTCCTAATATATTTGGTTTCTAATCGATTTTCTGGAGTTCCAGACAGGTTCAGTGGCAGTGGGTCAGGAACAGATTTCACACTCAAGATCAGCAGAGTGGAGGCTGAGGATTTGGGAGTTTATTATTGCTTCCAGAGTAACTATCTTCCTCT

IGKV1-99*01_B6	C57BL/6J	IGKV	MUSMUS IGKV1-99*01 F	100	GATGTTGTTCTGACCCAAACTCCACTCTCTCTGCCTGTCAATATTGGAGATCAAGCCTCTATCTCTTGCAAGTCTACTAAGAGTCTTCTGAATAGTGATGGATTCACTTATTTGGACTGGTACCTGCAGAAGCCAGGCCAGTCTCCACAGCTCCTAATATATTTGGTTTCTAATCGATTTTCTGGAGTTCCAGACAGGTTCAGTGGCAGTGGGTCAGGAACAGATTTCACACTCAAGATCAGCAGAGTGGAGGCTGAGGATTTGGGAGTTTATTATTGCTTCCAGAGTAACTATCTTCCTCT
IGKV1-99*01_BALB	BALB/CBYJ	IGKV	MUSMUS IGKV1-99*01 F	100	GATGTTGTTCTGACCCAAACTCCACTCTCTCTGCCTGTCAATATTGGAGATCAAGCCTCTATCTCTTGCAAGTCTACTAAGAGTCTTCTGAATAGTGATGGATTCACTTATTTGGACTGGTACCTGCAGAAGCCAGGCCAGTCTCCACAGCTCCTAATATATTTGGTTTCTAATCGATTTTCTGGAGTTCCAGACAGGTTCAGTGGCAGTGGGTCAGGAACAGATTTCACACTCAAGATCAGCAGAGTGGAGGCTGAGGATTTGGGAGTTTATTATTGCTTCCAGAGTAACTATCTTCCTCT
IGKV1-99*01_C3H	C3H/HEJ	IGKV	MUSMUS IGKV1-99*01 F	100	GATGTTGTTCTGACCCAAACTCCACTCTCTCTGCCTGTCAATATTGGAGATCAAGCCTCTATCTCTTGCAAGTCTACTAAGAGTCTTCTGAATAGTGATGGATTCACTTATTTGGACTGGTACCTGCAGAAGCCAGGCCAGTCTCCACAGCTCCTAATATATTTGGTTTCTAATCGATTTTCTGGAGTTCCAGACAGGTTCAGTGGCAGTGGGTCAGGAACAGATTTCACACTCAAGATCAGCAGAGTGGAGGCTGAGGATTTGGGAGTTTATTATTGCTTCCAGAGTAACTATCTTCCTCT
IGKV1-99*01_DBA1	DBA/1J	IGKV	MUSMUS IGKV1-99*01 F	100	GATGTTGTTCTGACCCAAACTCCACTCTCTCTGCCTGTCAATATTGGAGATCAAGCCTCTATCTCTTGCAAGTCTACTAAGAGTCTTCTGAATAGTGATGGATTCACTTATTTGGACTGGTACCTGCAGAAGCCAGGCCAGTCTCCACAGCTCCTAATATATTTGGTTTCTAATCGATTTTCTGGAGTTCCAGACAGGTTCAGTGGCAGTGGGTCAGGAACAGATTTCACACTCAAGATCAGCAGAGTGGAGGCTGAGGATTTGGGAGTTTATTATTGCTTCCAGAGTAACTATCTTCCTCT

IGKV1-99*01_NZB	NZB/BLNJ	IGKV	MUSMUS IGKV1-99*01 F	100	GATGTTGTTCTGACCCAAACTCCACTCTCTCTGCCTGTCAATATTGGAGATCAAGCCTCTATCTCTTGCAAGTCTACTAAGAGTCTTCTGAATAGTGATGGATTCACTTATTTGGACTGGTACCTGCAGAAGCCAGGCCAGTCTCCACAGCTCCTAATATATTTGGTTTCTAATCGATTTTCTGGAGTTCCAGACAGGTTCAGTGGCAGTGGGTCAGGAACAGATTTCACACTCAAGATCAGCAGAGTGGAGGCTGAGGATTTGGGAGTTTATTATTGCTTCCAGAGTAACTATCTTCCTCT
IGKV1-99*01_S1751_DBA2	DBA/2J	IGKV	MUSMUS IGKV1-99*01 F	100	GATGTTGTTCTGACCCAAACTCCACTCTCTCTGCCTGTCAATATTGGAGATCAAGCCTCTATCTCTTGCAAGTCTACTAAGAGTCTTCTGAATAGTGATGGATTCACTTATTTGGACTGGTACCTGCAGAAGCCAGGCCAGTCTCCACAGCTCCTAATATATTTGGTTTCTAATCGATTTTCTGGAGTTCCAGACAGGTTCAGTGGCAGTGGGTCAGGAACAGATTTCACACTCAAGATCAGCAGAGTGGAGGCTGAGGATTTGGGAGTTTATTATTGCTTCCAGAGTAACTATCTTCCGCT
IGKV1-99*01_S3206_AKR	AKR/J	IGKV	MUSMUS IGKV1-99*01 F	98.3	GATGTTGTTCTGACCCAAACTCCACTCTCTCTGCCTGTCAATATTGGAGATCAAGCCTCTATCTCTTGCAAGTCTACTAAGAGCCTTCTGAATAGTGATGGATTCACTTATTTGGGCTGGTACCTGCAGAAGCCAGGCCAGTCTCCACAGCTCCTAATATATTTGGTTTCTAATCGATTTTCTGGAGTTCCAGACAGGTTCAGTGGTAGTGGGTCAGGGACAGATTTCACCCTCAAGATCAGCAGAGTGGAGGCTGAGGATTTGGGAGTTTATTATTGCTTCCAGAGTAACTATCTTCCTCT
IGKV1-99*01_S3206_CAST	CAST/EIJ	IGKV	MUSMUS IGKV1-99*01 F	98.3	GATGTTGTTCTGACCCAAACTCCACTCTCTCTGCCTGTCAATATTGGAGATCAAGCCTCTATCTCTTGCAAGTCTACTAAGAGCCTTCTGAATAGTGATGGATTCACTTATTTGGGCTGGTACCTGCAGAAGCCAGGCCAGTCTCCACAGCTCCTAATATATTTGGTTTCTAATCGATTTTCTGGAGTTCCAGACAGGTTCAGTGGTAGTGGGTCAGGGACAGATTTCACCCTCAAGATCAGCAGAGTGGAGGCTGAGGATTTGGGAGTTTATTATTGCTTCCAGAGTAACTATCTTCCTCT

IGKV1-99*01_S3206_MRL	MRL/MPJ	IGKV	MUSMUS IGKV1-99*01 F	98.3	GATGTTGTTCTGACCCAAACTCCACTCTCTCTGCCTGTCAATATTGGAGATCAAGCCTCTATCTCTTGCAAGTCTACTAAGAGCCTTCTGAATAGTGATGGATTCACTTATTTGGGCTGGTACCTGCAGAAGCCAGGCCAGTCTCCACAGCTCCTAATATATTTGGTTTCTAATCGATTTTCTGGAGTTCCAGACAGGTTCAGTGGTAGTGGGTCAGGGACAGATTTCACCCTCAAGATCAGCAGAGTGGAGGCTGAGGATTTGGGAGTTTATTATTGCTTCCAGAGTAACTATCTTCCTCT
IGKV1-99*01_S3206_NOD	NOD/SHILTJ	IGKV	MUSMUS IGKV1-99*01 F	98.3	GATGTTGTTCTGACCCAAACTCCACTCTCTCTGCCTGTCAATATTGGAGATCAAGCCTCTATCTCTTGCAAGTCTACTAAGAGCCTTCTGAATAGTGATGGATTCACTTATTTGGGCTGGTACCTGCAGAAGCCAGGCCAGTCTCCACAGCTCCTAATATATTTGGTTTCTAATCGATTTTCTGGAGTTCCAGACAGGTTCAGTGGTAGTGGGTCAGGGACAGATTTCACCCTCAAGATCAGCAGAGTGGAGGCTGAGGATTTGGGAGTTTATTATTGCTTCCAGAGTAACTATCTTCCTCT
IGKV1-99*01_S3206_NOR	NOR/LTJ	IGKV	MUSMUS IGKV1-99*01 F	98.3	GATGTTGTTCTGACCCAAACTCCACTCTCTCTGCCTGTCAATATTGGAGATCAAGCCTCTATCTCTTGCAAGTCTACTAAGAGCCTTCTGAATAGTGATGGATTCACTTATTTGGGCTGGTACCTGCAGAAGCCAGGCCAGTCTCCACAGCTCCTAATATATTTGGTTTCTAATCGATTTTCTGGAGTTCCAGACAGGTTCAGTGGTAGTGGGTCAGGGACAGATTTCACCCTCAAGATCAGCAGAGTGGAGGCTGAGGATTTGGGAGTTTATTATTGCTTCCAGAGTAACTATCTTCCTCT
IGKV1-99*01_S6030_MSM	MSM/MSJ	IGKV	MUSMUS IGKV1-99*01 F	97.96	GATGTTGTTCTGACCCAAACTCCACTCTCTCTGCCTGTCAATATTGGAGATCAAGCCTCTATCTCTTGCAAGTCTACTAAGACCCTTCTGAATAGTGATGGATTCACTTATTTGGGCTGGTACCTGCAGAAGCCAGGCCAGTCTCCACAGCTCCTAATATATTTGGTTTCTAATCGATTTTCTGGAGTTCCAGACAGGTTCAGTGGTAGTGGGTCAGGGACAGATTTCACCCTCAAGATCAGCAGAGTGGAGGCTGAGGATTTGGGAGTTTATTATTGCTTCCAGAGTAACTATCTTCCTCT

IGKV1-99*01_S7463_NOR	NOR/LTJ	IGKV	MUSMUS IGKV1-99*01 F	98.3	GATGTTGTTCTGACCCAAACTCCACTCTCTCTGCCTGTCAATATTGGAGATCAAGCCTCTATCTCTTGCAAGTCTACTAAGAGCCTTCTGAATAGTGATGGATTCACTTATTTGGGCTGGTACCTGCAGAAGCCAGGCCAGTCTCCACAGCTCCTAATATATTTGGTTTCTAATCGATTTTCTGGAGTTCCAGACAGGTTCAGTGGTAGTGGGTCAGGGACAGATTTCACCCTCAAGATCAGCAGAGTGGAGGCTGAGGATTTGGGAGTTTATTATTGCTTCCAGAGTAACTATCTTCCGCT
IGKV10-94*01_129	129S1/SVIMJ	IGKV	MUSMUS IGKV10-94*01 F	100	GATATCCAGATGACACAGACTACATCCTCCCTGTCTGCCTCTCTGGGAGACAGAGTCACCATCAGTTGCAGTGCAAGTCAGGGCATTAGCAATTATTTAAACTGGTATCAGCAGAAACCAGATGGAACTGTTAAACTCCTGATCTATTACACATCAAGTTTACACTCAGGAGTCCCATCAAGGTTCAGTGGCAGTGGGTCTGGGACAGATTATTCTCTCACCATCAGCAACCTGGAACCTGAAGATATTGCCACTTACTATTGTCAGCAGTATAGTAAGCTTCCTCC
IGKV10-94*01_AJ	A/J	IGKV	MUSMUS IGKV10-94*01 F	100	GATATCCAGATGACACAGACTACATCCTCCCTGTCTGCCTCTCTGGGAGACAGAGTCACCATCAGTTGCAGTGCAAGTCAGGGCATTAGCAATTATTTAAACTGGTATCAGCAGAAACCAGATGGAACTGTTAAACTCCTGATCTATTACACATCAAGTTTACACTCAGGAGTCCCATCAAGGTTCAGTGGCAGTGGGTCTGGGACAGATTATTCTCTCACCATCAGCAACCTGGAACCTGAAGATATTGCCACTTACTATTGTCAGCAGTATAGTAAGCTTCCTCC
IGKV10-94*01_B6	C57BL/6J	IGKV	MUSMUS IGKV10-94*01 F	100	GATATCCAGATGACACAGACTACATCCTCCCTGTCTGCCTCTCTGGGAGACAGAGTCACCATCAGTTGCAGTGCAAGTCAGGGCATTAGCAATTATTTAAACTGGTATCAGCAGAAACCAGATGGAACTGTTAAACTCCTGATCTATTACACATCAAGTTTACACTCAGGAGTCCCATCAAGGTTCAGTGGCAGTGGGTCTGGGACAGATTATTCTCTCACCATCAGCAACCTGGAACCTGAAGATATTGCCACTTACTATTGTCAGCAGTATAGTAAGCTTCCTCC

IGKV10-94*01_BALB	BALB/CBYJ	IGKV	MUSMUS IGKV10-94*01 F	100	GATATCCAGATGACACAGACTACATCCTCCCTGTCTGCCTCTCTGGGAGACAGAGTCACCATCAGTTGCAGTGCAAGTCAGGGCATTAGCAATTATTTAAACTGGTATCAGCAGAAACCAGATGGAACTGTTAAACTCCTGATCTATTACACATCAAGTTTACACTCAGGAGTCCCATCAAGGTTCAGTGGCAGTGGGTCTGGGACAGATTATTCTCTCACCATCAGCAACCTGGAACCTGAAGATATTGCCACTTACTATTGTCAGCAGTATAGTAAGCTTCCTCC
IGKV10-94*01_C3H	C3H/HEJ	IGKV	MUSMUS IGKV10-94*01 F	100	GATATCCAGATGACACAGACTACATCCTCCCTGTCTGCCTCTCTGGGAGACAGAGTCACCATCAGTTGCAGTGCAAGTCAGGGCATTAGCAATTATTTAAACTGGTATCAGCAGAAACCAGATGGAACTGTTAAACTCCTGATCTATTACACATCAAGTTTACACTCAGGAGTCCCATCAAGGTTCAGTGGCAGTGGGTCTGGGACAGATTATTCTCTCACCATCAGCAACCTGGAACCTGAAGATATTGCCACTTACTATTGTCAGCAGTATAGTAAGCTTCCTCC
IGKV10-94*01_CBA	CBA/J	IGKV	MUSMUS IGKV10-94*01 F	100	GATATCCAGATGACACAGACTACATCCTCCCTGTCTGCCTCTCTGGGAGACAGAGTCACCATCAGTTGCAGTGCAAGTCAGGGCATTAGCAATTATTTAAACTGGTATCAGCAGAAACCAGATGGAACTGTTAAACTCCTGATCTATTACACATCAAGTTTACACTCAGGAGTCCCATCAAGGTTCAGTGGCAGTGGGTCTGGGACAGATTATTCTCTCACCATCAGCAACCTGGAACCTGAAGATATTGCCACTTACTATTGTCAGCAGTATAGTAAGCTTCCTCC
IGKV10-94*01_DBA1	DBA/1J	IGKV	MUSMUS IGKV10-94*01 F	100	GATATCCAGATGACACAGACTACATCCTCCCTGTCTGCCTCTCTGGGAGACAGAGTCACCATCAGTTGCAGTGCAAGTCAGGGCATTAGCAATTATTTAAACTGGTATCAGCAGAAACCAGATGGAACTGTTAAACTCCTGATCTATTACACATCAAGTTTACACTCAGGAGTCCCATCAAGGTTCAGTGGCAGTGGGTCTGGGACAGATTATTCTCTCACCATCAGCAACCTGGAACCTGAAGATATTGCCACTTACTATTGTCAGCAGTATAGTAAGCTTCCTCC

IGKV10-94*01_DBA2	DBA/2J	IGKV	MUSMUS IGKV10-94*01 F	100	GATATCCAGATGACACAGACTACATCCTCCCTGTCTGCCTCTCTGGGAGACAGAGTCACCATCAGTTGCAGTGCAAGTCAGGGCATTAGCAATTATTTAAACTGGTATCAGCAGAAACCAGATGGAACTGTTAAACTCCTGATCTATTACACATCAAGTTTACACTCAGGAGTCCCATCAAGGTTCAGTGGCAGTGGGTCTGGGACAGATTATTCTCTCACCATCAGCAACCTGGAACCTGAAGATATTGCCACTTACTATTGTCAGCAGTATAGTAAGCTTCCTCC
IGKV10-94*01_LEWES	LEWES/EIJ	IGKV	MUSMUS IGKV10-94*01 F	100	GATATCCAGATGACACAGACTACATCCTCCCTGTCTGCCTCTCTGGGAGACAGAGTCACCATCAGTTGCAGTGCAAGTCAGGGCATTAGCAATTATTTAAACTGGTATCAGCAGAAACCAGATGGAACTGTTAAACTCCTGATCTATTACACATCAAGTTTACACTCAGGAGTCCCATCAAGGTTCAGTGGCAGTGGGTCTGGGACAGATTATTCTCTCACCATCAGCAACCTGGAACCTGAAGATATTGCCACTTACTATTGTCAGCAGTATAGTAAGCTTCCTCC
IGKV10-94*01_MUS_SPRETUS_S4111_MSM	MSM/MSJ	IGKV	MUSSPR IGKV10-94*01 F	98.21	GATATCCAGATGACACAGACTACATCCTCCCTGTCTGCCTCTCTGGGAGACAGAGTCACCATCAGTTGCAGGGCAAGTCAGGATATTAGCAATTATTTAAACTGGTATCAGCAGAAACCAGATGGAACTGTTAAACCCCTGATCTATTATGCGTCAAGTTTACACTCAGGAGTCCCATCAAGGTTCAGTGGCAGTGGGTCTGGGACAGATTATTCTCTCACCATCAGCAACCTGGAACCTGAAGATATTGCCACTTACTATTGTCAGCAGTATAGTAAGCTTCCTCC
IGKV10-94*01_MUS_SPRETUS_S4447_PWD	PWD/PHJ	IGKV	MUSSPR IGKV10-94*01 F	97.49	GATATCCAGATGACACAGACTACATCCTCCCTGTCTGCCTCTCTAGGAGACAGAGTCACCATCAGTTGCAGGGCAAGTCAGGATATTAGCAATTATTTAAACTGGTATCAGCAGAAACCAGATGGAACTGTTAAACCCCTGATCTATTATGCGTCAAGTTTACACTCAGGAGTCCCATCAAGGTTCAGTGGCAGTGGGTCTGGGACAGATTATTCTCTCACCATCAGCAACCTGGAACCTGAAGATATTGCCACTTATTATTGTCAGCAGTATAGTAAGCTTCCTCC

IGKV10-94*01_NZB	NZB/BLNJ	IGKV	MUSMUS IGKV10-94*01 F	100	GATATCCAGATGACACAGACTACATCCTCCCTGTCTGCCTCTCTGGGAGACAGAGTCACCATCAGTTGCAGTGCAAGTCAGGGCATTAGCAATTATTTAAACTGGTATCAGCAGAAACCAGATGGAACTGTTAAACTCCTGATCTATTACACATCAAGTTTACACTCAGGAGTCCCATCAAGGTTCAGTGGCAGTGGGTCTGGGACAGATTATTCTCTCACCATCAGCAACCTGGAACCTGAAGATATTGCCACTTACTATTGTCAGCAGTATAGTAAGCTTCCTCC
IGKV10-94*01_S9482_MSM	MSM/MSJ	IGKV	MUSMUS IGKV10-94*01 F	98.21	GATATCCAGATGACACAGAGTACATCCACCCTGTCTGCCTCTCTGGGAGACAGAGTCACCATCAGTTGCAGTGCAAGTCAGGGCATTAGCAATTATTTAAACTGGTATCAGCAGAAACCAGATGGAACTGTTAAACTCCTGATCTATTATACATCAAGTTTACACTCAGGAGTCCCATCAAGGTTCAGTGGCAGTGGGTCTGGAACAGATTATTCTCTCACCATCAGCAACCTGGAACCTGAAGATATTGCCACTTACTATTGTCAGCAGTATAGTAATTTTCCTCC
IGKV10-94*01_S9482_PWD	PWD/PHJ	IGKV	MUSMUS IGKV10-94*01 F	98.21	GATATCCAGATGACACAGAGTACATCCACCCTGTCTGCCTCTCTGGGAGACAGAGTCACCATCAGTTGCAGTGCAAGTCAGGGCATTAGCAATTATTTAAACTGGTATCAGCAGAAACCAGATGGAACTGTTAAACTCCTGATCTATTATACATCAAGTTTACACTCAGGAGTCCCATCAAGGTTCAGTGGCAGTGGGTCTGGAACAGATTATTCTCTCACCATCAGCAACCTGGAACCTGAAGATATTGCCACTTACTATTGTCAGCAGTATAGTAATTTTCCTCC
IGKV10-94*01_SJL	SJL/J	IGKV	MUSMUS IGKV10-94*01 F	100	GATATCCAGATGACACAGACTACATCCTCCCTGTCTGCCTCTCTGGGAGACAGAGTCACCATCAGTTGCAGTGCAAGTCAGGGCATTAGCAATTATTTAAACTGGTATCAGCAGAAACCAGATGGAACTGTTAAACTCCTGATCTATTACACATCAAGTTTACACTCAGGAGTCCCATCAAGGTTCAGTGGCAGTGGGTCTGGGACAGATTATTCTCTCACCATCAGCAACCTGGAACCTGAAGATATTGCCACTTACTATTGTCAGCAGTATAGTAAGCTTCCTCC

IGKV10-94*03_AKR	AKR/J	IGKV	MUSMUS IGKV10-94*03 F, OR MUSMUS IGKV10-94*08 F	100	GATATCCAGATGACACAGACTACATCCTCCCTGTCTGCCTCTCTGGGAGACAGAGTCACCATCAGTTGCAGGGCAAGTCAGGATATTAGCAATTATTTAAACTGGTATCAGCAGAAACCAGATGGAACTGTTAAACTCCTGATCTACTACACATCAAGATTACACTCAGGAGTCCCATCAAGGTTCAGTGGCAGTGGGTCTGGGACAGATTATTCTCTCACCATCAGCAACCTGGAACCTGAAGATATTGCCACTTACTATTGTCAGCAGTATAGTAAGCTTCCTCC
IGKV10-94*03_CAST	CAST/EIJ	IGKV	MUSMUS IGKV10-94*03 F, OR MUSMUS IGKV10-94*08 F	100	GATATCCAGATGACACAGACTACATCCTCCCTGTCTGCCTCTCTGGGAGACAGAGTCACCATCAGTTGCAGGGCAAGTCAGGATATTAGCAATTATTTAAACTGGTATCAGCAGAAACCAGATGGAACTGTTAAACTCCTGATCTACTACACATCAAGATTACACTCAGGAGTCCCATCAAGGTTCAGTGGCAGTGGGTCTGGGACAGATTATTCTCTCACCATCAGCAACCTGGAACCTGAAGATATTGCCACTTACTATTGTCAGCAGTATAGTAAGCTTCCTCC
IGKV10-94*03_MRL	MRL/MPJ	IGKV	MUSMUS IGKV10-94*03 F, OR MUSMUS IGKV10-94*08 F	100	GATATCCAGATGACACAGACTACATCCTCCCTGTCTGCCTCTCTGGGAGACAGAGTCACCATCAGTTGCAGGGCAAGTCAGGATATTAGCAATTATTTAAACTGGTATCAGCAGAAACCAGATGGAACTGTTAAACTCCTGATCTACTACACATCAAGATTACACTCAGGAGTCCCATCAAGGTTCAGTGGCAGTGGGTCTGGGACAGATTATTCTCTCACCATCAGCAACCTGGAACCTGAAGATATTGCCACTTACTATTGTCAGCAGTATAGTAAGCTTCCTCC
IGKV10-94*03_NOD	NOD/SHILTJ	IGKV	MUSMUS IGKV10-94*03 F, OR MUSMUS IGKV10-94*08 F	100	GATATCCAGATGACACAGACTACATCCTCCCTGTCTGCCTCTCTGGGAGACAGAGTCACCATCAGTTGCAGGGCAAGTCAGGATATTAGCAATTATTTAAACTGGTATCAGCAGAAACCAGATGGAACTGTTAAACTCCTGATCTACTACACATCAAGATTACACTCAGGAGTCCCATCAAGGTTCAGTGGCAGTGGGTCTGGGACAGATTATTCTCTCACCATCAGCAACCTGGAACCTGAAGATATTGCCACTTACTATTGTCAGCAGTATAGTAAGCTTCCTCC

IGKV10-94*03_NOR	NOR/LTJ	IGKV	MUSMUS IGKV10-94*03 F, OR MUSMUS IGKV10-94*08 F	100	GATATCCAGATGACACAGACTACATCCTCCCTGTCTGCCTCTCTGGGAGACAGAGTCACCATCAGTTGCAGGGCAAGTCAGGATATTAGCAATTATTTAAACTGGTATCAGCAGAAACCAGATGGAACTGTTAAACTCCTGATCTACTACACATCAAGATTACACTCAGGAGTCCCATCAAGGTTCAGTGGCAGTGGGTCTGGGACAGATTATTCTCTCACCATCAGCAACCTGGAACCTGAAGATATTGCCACTTACTATTGTCAGCAGTATAGTAAGCTTCCTCC
IGKV10-95*01_129	129S1/SVIMJ	IGKV	MUSMUS IGKV10-95*01 F	100	GATATCCAGATGACACAGACTACTTCCTCCCTGTCTGCCTCTCTGGGAGACAGAGTCACCATCAGTTGCAGGGCAAGTGAGGACATTAGCACTTATTTAAACTGGTATCAGCAGAAACCAGATGGAACTGTTAAACTCCTGATCTATTACACATCAGGATTACACTCAGGAGTCCCATCAAGGTTCAGTGGCAGTGGGTCTGGGGCAGATTATTCTCTCACCATCAGCAACCTGGAACCTGAAGATATTGCCACTTACTATTGTCAGCAGTATAGTAAGCTTCCTCC
IGKV10-95*01_AJ	A/J	IGKV	MUSMUS IGKV10-95*01 F	100	GATATCCAGATGACACAGACTACTTCCTCCCTGTCTGCCTCTCTGGGAGACAGAGTCACCATCAGTTGCAGGGCAAGTGAGGACATTAGCACTTATTTAAACTGGTATCAGCAGAAACCAGATGGAACTGTTAAACTCCTGATCTATTACACATCAGGATTACACTCAGGAGTCCCATCAAGGTTCAGTGGCAGTGGGTCTGGGGCAGATTATTCTCTCACCATCAGCAACCTGGAACCTGAAGATATTGCCACTTACTATTGTCAGCAGTATAGTAAGCTTCCTCC
IGKV10-95*01_BALB	BALB/CBYJ	IGKV	MUSMUS IGKV10-95*01 F	100	GATATCCAGATGACACAGACTACTTCCTCCCTGTCTGCCTCTCTGGGAGACAGAGTCACCATCAGTTGCAGGGCAAGTGAGGACATTAGCACTTATTTAAACTGGTATCAGCAGAAACCAGATGGAACTGTTAAACTCCTGATCTATTACACATCAGGATTACACTCAGGAGTCCCATCAAGGTTCAGTGGCAGTGGGTCTGGGGCAGATTATTCTCTCACCATCAGCAACCTGGAACCTGAAGATATTGCCACTTACTATTGTCAGCAGTATAGTAAGCTTCCTCC

IGKV10-95*01_C3H	C3H/HEJ	IGKV	MUSMUS IGKV10-95*01 F	100	GATATCCAGATGACACAGACTACTTCCTCCCTGTCTGCCTCTCTGGGAGACAGAGTCACCATCAGTTGCAGGGCAAGTGAGGACATTAGCACTTATTTAAACTGGTATCAGCAGAAACCAGATGGAACTGTTAAACTCCTGATCTATTACACATCAGGATTACACTCAGGAGTCCCATCAAGGTTCAGTGGCAGTGGGTCTGGGGCAGATTATTCTCTCACCATCAGCAACCTGGAACCTGAAGATATTGCCACTTACTATTGTCAGCAGTATAGTAAGCTTCCTCC
IGKV10-95*01_CBA	CBA/J	IGKV	MUSMUS IGKV10-95*01 F	100	GATATCCAGATGACACAGACTACTTCCTCCCTGTCTGCCTCTCTGGGAGACAGAGTCACCATCAGTTGCAGGGCAAGTGAGGACATTAGCACTTATTTAAACTGGTATCAGCAGAAACCAGATGGAACTGTTAAACTCCTGATCTATTACACATCAGGATTACACTCAGGAGTCCCATCAAGGTTCAGTGGCAGTGGGTCTGGGGCAGATTATTCTCTCACCATCAGCAACCTGGAACCTGAAGATATTGCCACTTACTATTGTCAGCAGTATAGTAAGCTTCCTCC
IGKV10-95*01_DBA1	DBA/1J	IGKV	MUSMUS IGKV10-95*01 F	100	GATATCCAGATGACACAGACTACTTCCTCCCTGTCTGCCTCTCTGGGAGACAGAGTCACCATCAGTTGCAGGGCAAGTGAGGACATTAGCACTTATTTAAACTGGTATCAGCAGAAACCAGATGGAACTGTTAAACTCCTGATCTATTACACATCAGGATTACACTCAGGAGTCCCATCAAGGTTCAGTGGCAGTGGGTCTGGGGCAGATTATTCTCTCACCATCAGCAACCTGGAACCTGAAGATATTGCCACTTACTATTGTCAGCAGTATAGTAAGCTTCCTCC
IGKV10-95*01_DBA2	DBA/2J	IGKV	MUSMUS IGKV10-95*01 F	100	GATATCCAGATGACACAGACTACTTCCTCCCTGTCTGCCTCTCTGGGAGACAGAGTCACCATCAGTTGCAGGGCAAGTGAGGACATTAGCACTTATTTAAACTGGTATCAGCAGAAACCAGATGGAACTGTTAAACTCCTGATCTATTACACATCAGGATTACACTCAGGAGTCCCATCAAGGTTCAGTGGCAGTGGGTCTGGGGCAGATTATTCTCTCACCATCAGCAACCTGGAACCTGAAGATATTGCCACTTACTATTGTCAGCAGTATAGTAAGCTTCCTCC

IGKV10-95*01_NZB	NZB/BLNJ	IGKV	MUSMUS IGKV10-95*01 F	100	GATATCCAGATGACACAGACTACTTCCTCCCTGTCTGCCTCTCTGGGAGACAGAGTCACCATCAGTTGCAGGGCAAGTGAGGACATTAGCACTTATTTAAACTGGTATCAGCAGAAACCAGATGGAACTGTTAAACTCCTGATCTATTACACATCAGGATTACACTCAGGAGTCCCATCAAGGTTCAGTGGCAGTGGGTCTGGGGCAGATTATTCTCTCACCATCAGCAACCTGGAACCTGAAGATATTGCCACTTACTATTGTCAGCAGTATAGTAAGCTTCCTCC
IGKV10-96*01_129	129S1/SVIMJ	IGKV	MUSMUS IGKV10-96*01 F	100	GATATCCAGATGACACAGACTACATCCTCCCTGTCTGCCTCTCTGGGAGACAGAGTCACCATCAGTTGCAGGGCAAGTCAGGACATTAGCAATTATTTAAACTGGTATCAGCAGAAACCAGATGGAACTGTTAAACTCCTGATCTACTACACATCAAGATTACACTCAGGAGTCCCATCAAGGTTCAGTGGCAGTGGGTCTGGAACAGATTATTCTCTCACCATTAGCAACCTGGAGCAAGAAGATATTGCCACTTACTTTTGCCAACAGGGTAATACGCTTCCTC
IGKV10-96*01_AJ	A/J	IGKV	MUSMUS IGKV10-96*01 F	100	GATATCCAGATGACACAGACTACATCCTCCCTGTCTGCCTCTCTGGGAGACAGAGTCACCATCAGTTGCAGGGCAAGTCAGGACATTAGCAATTATTTAAACTGGTATCAGCAGAAACCAGATGGAACTGTTAAACTCCTGATCTACTACACATCAAGATTACACTCAGGAGTCCCATCAAGGTTCAGTGGCAGTGGGTCTGGAACAGATTATTCTCTCACCATTAGCAACCTGGAGCAAGAAGATATTGCCACTTACTTTTGCCAACAGGGTAATACGCTTCCTC
IGKV10-96*01_B6	C57BL/6J	IGKV	MUSMUS IGKV10-96*01 F	100	GATATCCAGATGACACAGACTACATCCTCCCTGTCTGCCTCTCTGGGAGACAGAGTCACCATCAGTTGCAGGGCAAGTCAGGACATTAGCAATTATTTAAACTGGTATCAGCAGAAACCAGATGGAACTGTTAAACTCCTGATCTACTACACATCAAGATTACACTCAGGAGTCCCATCAAGGTTCAGTGGCAGTGGGTCTGGAACAGATTATTCTCTCACCATTAGCAACCTGGAGCAAGAAGATATTGCCACTTACTTTTGCCAACAGGGTAATACGCTTCCTC

IGKV10-96*01_BALB	BALB/CBYJ	IGKV	MUSMUS IGKV10-96*01 F	100	GATATCCAGATGACACAGACTACATCCTCCCTGTCTGCCTCTCTGGGAGACAGAGTCACCATCAGTTGCAGGGCAAGTCAGGACATTAGCAATTATTTAAACTGGTATCAGCAGAAACCAGATGGAACTGTTAAACTCCTGATCTACTACACATCAAGATTACACTCAGGAGTCCCATCAAGGTTCAGTGGCAGTGGGTCTGGAACAGATTATTCTCTCACCATTAGCAACCTGGAGCAAGAAGATATTGCCACTTACTTTTGCCAACAGGGTAATACGCTTCCTC
IGKV10-96*01_C3H	C3H/HEJ	IGKV	MUSMUS IGKV10-96*01 F	100	GATATCCAGATGACACAGACTACATCCTCCCTGTCTGCCTCTCTGGGAGACAGAGTCACCATCAGTTGCAGGGCAAGTCAGGACATTAGCAATTATTTAAACTGGTATCAGCAGAAACCAGATGGAACTGTTAAACTCCTGATCTACTACACATCAAGATTACACTCAGGAGTCCCATCAAGGTTCAGTGGCAGTGGGTCTGGAACAGATTATTCTCTCACCATTAGCAACCTGGAGCAAGAAGATATTGCCACTTACTTTTGCCAACAGGGTAATACGCTTCCTC
IGKV10-96*01_CBA	CBA/J	IGKV	MUSMUS IGKV10-96*01 F	100	GATATCCAGATGACACAGACTACATCCTCCCTGTCTGCCTCTCTGGGAGACAGAGTCACCATCAGTTGCAGGGCAAGTCAGGACATTAGCAATTATTTAAACTGGTATCAGCAGAAACCAGATGGAACTGTTAAACTCCTGATCTACTACACATCAAGATTACACTCAGGAGTCCCATCAAGGTTCAGTGGCAGTGGGTCTGGAACAGATTATTCTCTCACCATTAGCAACCTGGAGCAAGAAGATATTGCCACTTACTTTTGCCAACAGGGTAATACGCTTCCTC
IGKV10-96*01_DBA1	DBA/1J	IGKV	MUSMUS IGKV10-96*01 F	100	GATATCCAGATGACACAGACTACATCCTCCCTGTCTGCCTCTCTGGGAGACAGAGTCACCATCAGTTGCAGGGCAAGTCAGGACATTAGCAATTATTTAAACTGGTATCAGCAGAAACCAGATGGAACTGTTAAACTCCTGATCTACTACACATCAAGATTACACTCAGGAGTCCCATCAAGGTTCAGTGGCAGTGGGTCTGGAACAGATTATTCTCTCACCATTAGCAACCTGGAGCAAGAAGATATTGCCACTTACTTTTGCCAACAGGGTAATACGCTTCCTC

IGKV10-96*01_DBA2	DBA/2J	IGKV	MUSMUS IGKV10-96*01 F	100	GATATCCAGATGACACAGACTACATCCTCCCTGTCTGCCTCTCTGGGAGACAGAGTCACCATCAGTTGCAGGGCAAGTCAGGACATTAGCAATTATTTAAACTGGTATCAGCAGAAACCAGATGGAACTGTTAAACTCCTGATCTACTACACATCAAGATTACACTCAGGAGTCCCATCAAGGTTCAGTGGCAGTGGGTCTGGAACAGATTATTCTCTCACCATTAGCAACCTGGAGCAAGAAGATATTGCCACTTACTTTTGCCAACAGGGTAATACGCTTCCTC
IGKV10-96*01_LEWES	LEWES/EIJ	IGKV	MUSMUS IGKV10-96*01 F	100	GATATCCAGATGACACAGACTACATCCTCCCTGTCTGCCTCTCTGGGAGACAGAGTCACCATCAGTTGCAGGGCAAGTCAGGACATTAGCAATTATTTAAACTGGTATCAGCAGAAACCAGATGGAACTGTTAAACTCCTGATCTACTACACATCAAGATTACACTCAGGAGTCCCATCAAGGTTCAGTGGCAGTGGGTCTGGAACAGATTATTCTCTCACCATTAGCAACCTGGAGCAAGAAGATATTGCCACTTACTTTTGCCAACAGGGTAATACGCTTCCTC
IGKV10-96*01_NZB	NZB/BLNJ	IGKV	MUSMUS IGKV10-96*01 F	100	GATATCCAGATGACACAGACTACATCCTCCCTGTCTGCCTCTCTGGGAGACAGAGTCACCATCAGTTGCAGGGCAAGTCAGGACATTAGCAATTATTTAAACTGGTATCAGCAGAAACCAGATGGAACTGTTAAACTCCTGATCTACTACACATCAAGATTACACTCAGGAGTCCCATCAAGGTTCAGTGGCAGTGGGTCTGGAACAGATTATTCTCTCACCATTAGCAACCTGGAGCAAGAAGATATTGCCACTTACTTTTGCCAACAGGGTAATACGCTTCCTC
IGKV10-96*01_SJL	SJL/J	IGKV	MUSMUS IGKV10-96*01 F	100	GATATCCAGATGACACAGACTACATCCTCCCTGTCTGCCTCTCTGGGAGACAGAGTCACCATCAGTTGCAGGGCAAGTCAGGACATTAGCAATTATTTAAACTGGTATCAGCAGAAACCAGATGGAACTGTTAAACTCCTGATCTACTACACATCAAGATTACACTCAGGAGTCCCATCAAGGTTCAGTGGCAGTGGGTCTGGAACAGATTATTCTCTCACCATTAGCAACCTGGAGCAAGAAGATATTGCCACTTACTTTTGCCAACAGGGTAATACGCTTCCTC

IGKV10-96*03_MRL	MRL/MPJ	IGKV	MUSMUS IGKV10-96*03 F	100	GATATCCAGATGACACAGACTACATCCTCCCTGTCTGCCTCTCTGGGAGACAGAGTCACCATCAGTTGCAGGGCAAGTCAGGACATTAGCAATTATTTAAACTGGTATCAGCAGAAACCAGATGGAACTGTTAAACTCCTGATCTACTACACATCAAGATTACACTCAGGAGTCCCATCAAGGTTCAGTGGCAGTGGGTCTGGGACAGATTATTCTCTCACTATTAGCAACCTGGAACAAGAAGATATTGCCACTTACTTTTGCCAACAGGATAGTAAGCATCCTCC
IGKV10-96*03_NOD	NOD/SHILTJ	IGKV	MUSMUS IGKV10-96*03 F	100	GATATCCAGATGACACAGACTACATCCTCCCTGTCTGCCTCTCTGGGAGACAGAGTCACCATCAGTTGCAGGGCAAGTCAGGACATTAGCAATTATTTAAACTGGTATCAGCAGAAACCAGATGGAACTGTTAAACTCCTGATCTACTACACATCAAGATTACACTCAGGAGTCCCATCAAGGTTCAGTGGCAGTGGGTCTGGGACAGATTATTCTCTCACTATTAGCAACCTGGAACAAGAAGATATTGCCACTTACTTTTGCCAACAGGATAGTAAGCATCCTCC
IGKV10-96*03_NOR	NOR/LTJ	IGKV	MUSMUS IGKV10-96*03 F	100	GATATCCAGATGACACAGACTACATCCTCCCTGTCTGCCTCTCTGGGAGACAGAGTCACCATCAGTTGCAGGGCAAGTCAGGACATTAGCAATTATTTAAACTGGTATCAGCAGAAACCAGATGGAACTGTTAAACTCCTGATCTACTACACATCAAGATTACACTCAGGAGTCCCATCAAGGTTCAGTGGCAGTGGGTCTGGGACAGATTATTCTCTCACTATTAGCAACCTGGAACAAGAAGATATTGCCACTTACTTTTGCCAACAGGATAGTAAGCATCCTCC
IGKV10-96*03_S5544_AKR	AKR/J	IGKV	MUSMUS IGKV10-96*03 F	99.64	GATATCCAGATGACACAGACTACATCCTCCCTGTCTGCCTCTCTGGGAGACAGAGTCACCATCAGTTGCAGGGCAAGTCAGGACATTAGCAATTATTTAAACTGGTATCAGCAGAAACCAGATGGAACTGTTAAACTCCTGATCTACTACACATCAAGATTACACTCAGGAGTCCCATCAAGGTTCAGTGGCAGTGGGTCTGGGACAGATTATTCTCTCACTATCAGCAACCTGGAACAAGAAGATATTGCCACTTACTTTTGCCAACAGGATAGTAAGCATCCTCC

IGKV10-96*03_S5777_PWD	PWD/PHJ	IGKV	MUSMUS IGKV10-96*03 F	99.28	GATATCCAGATGACACAGACTACATCCTCCCTGTCTGCCTCTCTGGGAGACAGAGTCACCATCAGTTGCAGGGCAAGTCAGGACATTAGCAATTATTTAAACTGGTATCAGCAGAAACCAGATGGAACTGTTAAACTCCTGATCTACTACACATCAAGATTACACTCAGGAGTCCCATCAAGGTTCAGTGGCAGTGGGTCTGGGACAGATTATTCTCTCATCATTAGCAACCTGGAACAAGAAGATATTGCCACTTACTTTTGCCAACAGGATAGTAAGCGTCCTCC
IGKV10-96*04_MSM	MSM/MSJ	IGKV	MUSMUS IGKV10-96*04 F	100	GATATCCAGATGACACAGACTACATCCTCCCTGTCTGCCTCTCTGGGAGACAGAGTCACCATCAGTTGCAGGGCAAGTCAGGACATTAGCAATTATTTAAACTGGTATCAGCAGAAACCAGATGGAACTGTTAAACTACTGATCTACTACACATCAAGATTACACTCAGGAGTCCCATCAAGGTTCAGTGGCAGTGGGTCTGGGACAGATTATTCTCTCACTATTAGCAACCTGGAACAAGAAGATATTGCCACTTACTTTTGCCAACAGGATAGTAAGCATCCTCC
IGKV10-96*06_CAST	CAST/EIJ	IGKV	MUSMUS IGKV10-96*06 ORF	100	GATATCCAGATGACACAGACTACATCCTCCCTGTCTGCCTCTCTGGGAGACAGAGTCACCATCAGTTGCAGGGCAAGTCAGGACATTAGCAATTATTTAAACGGGTATCAGCAGAAACCAGATGGAACTGTTAAACTCCTGATCTACTACACATCAAGATTACACTCAGGAGTCCCATCAAGGTTCAGTGGCAGTGGGTCTGGGACAGATTATTCTCTCACTATTAGCAACCTGGAACAAGAAGATATTGCCACTTACTTTTGCCAACAGGATAGTAAGCATCCTCC
IGKV11-125*01_129	129S1/SVIMJ	IGKV	MUSMUS IGKV11-125*01 F	100	GATGTCCAGATGATTCAGTCTCCATCCTCCCTGTCTGCATCTTTGGGAGACATAGTCACCATGACTTGCCAGGCAAGTCAGGGCACTAGCATTAATTTAAACTGGTTTCAGCAAAAACCAGGGAAAGCTCCTAAGCTCCTGATCTATGGTGCAAGCAACTTGGAAGATGGGGTCCCATCAAGGTTCAGTGGCAGTAGATATGGGACAGATTTCACTCTCACCATCAGCAGCCTGGAGGATGAAGATATGGCAACTTATTTCTGTCTACAGCATAGTTATCTCCCTCC

IGKV11-125*01_AJ	A/J	IGKV	MUSMUS IGKV11-125*01 F	100	GATGTCCAGATGATTCAGTCTCCATCCTCCCTGTCTGCATCTTTGGGAGACATAGTCACCATGACTTGCCAGGCAAGTCAGGGCACTAGCATTAATTTAAACTGGTTTCAGCAAAAACCAGGGAAAGCTCCTAAGCTCCTGATCTATGGTGCAAGCAACTTGGAAGATGGGGTCCCATCAAGGTTCAGTGGCAGTAGATATGGGACAGATTTCACTCTCACCATCAGCAGCCTGGAGGATGAAGATATGGCAACTTATTTCTGTCTACAGCATAGTTATCTCCCTCC
IGKV11-125*01_AKR	AKR/J	IGKV	MUSMUS IGKV11-125*01 F	100	GATGTCCAGATGATTCAGTCTCCATCCTCCCTGTCTGCATCTTTGGGAGACATAGTCACCATGACTTGCCAGGCAAGTCAGGGCACTAGCATTAATTTAAACTGGTTTCAGCAAAAACCAGGGAAAGCTCCTAAGCTCCTGATCTATGGTGCAAGCAACTTGGAAGATGGGGTCCCATCAAGGTTCAGTGGCAGTAGATATGGGACAGATTTCACTCTCACCATCAGCAGCCTGGAGGATGAAGATATGGCAACTTATTTCTGTCTACAGCATAGTTATCTCCCTCC
IGKV11-125*01_B6	C57BL/6J	IGKV	MUSMUS IGKV11-125*01 F	100	GATGTCCAGATGATTCAGTCTCCATCCTCCCTGTCTGCATCTTTGGGAGACATAGTCACCATGACTTGCCAGGCAAGTCAGGGCACTAGCATTAATTTAAACTGGTTTCAGCAAAAACCAGGGAAAGCTCCTAAGCTCCTGATCTATGGTGCAAGCAACTTGGAAGATGGGGTCCCATCAAGGTTCAGTGGCAGTAGATATGGGACAGATTTCACTCTCACCATCAGCAGCCTGGAGGATGAAGATATGGCAACTTATTTCTGTCTACAGCATAGTTATCTCCCTCC
IGKV11-125*01_BALB	BALB/CBYJ	IGKV	MUSMUS IGKV11-125*01 F	100	GATGTCCAGATGATTCAGTCTCCATCCTCCCTGTCTGCATCTTTGGGAGACATAGTCACCATGACTTGCCAGGCAAGTCAGGGCACTAGCATTAATTTAAACTGGTTTCAGCAAAAACCAGGGAAAGCTCCTAAGCTCCTGATCTATGGTGCAAGCAACTTGGAAGATGGGGTCCCATCAAGGTTCAGTGGCAGTAGATATGGGACAGATTTCACTCTCACCATCAGCAGCCTGGAGGATGAAGATATGGCAACTTATTTCTGTCTACAGCATAGTTATCTCCCTCC

IGKV11-125*01_C3H	C3H/HEJ	IGKV	MUSMUS IGKV11-125*01 F	100	GATGTCCAGATGATTCAGTCTCCATCCTCCCTGTCTGCATCTTTGGGAGACATAGTCACCATGACTTGCCAGGCAAGTCAGGGCACTAGCATTAATTTAAACTGGTTTCAGCAAAAACCAGGGAAAGCTCCTAAGCTCCTGATCTATGGTGCAAGCAACTTGGAAGATGGGGTCCCATCAAGGTTCAGTGGCAGTAGATATGGGACAGATTTCACTCTCACCATCAGCAGCCTGGAGGATGAAGATATGGCAACTTATTTCTGTCTACAGCATAGTTATCTCCCTCC
IGKV11-125*01_CAST	CAST/EIJ	IGKV	MUSMUS IGKV11-125*01 F	100	GATGTCCAGATGATTCAGTCTCCATCCTCCCTGTCTGCATCTTTGGGAGACATAGTCACCATGACTTGCCAGGCAAGTCAGGGCACTAGCATTAATTTAAACTGGTTTCAGCAAAAACCAGGGAAAGCTCCTAAGCTCCTGATCTATGGTGCAAGCAACTTGGAAGATGGGGTCCCATCAAGGTTCAGTGGCAGTAGATATGGGACAGATTTCACTCTCACCATCAGCAGCCTGGAGGATGAAGATATGGCAACTTATTTCTGTCTACAGCATAGTTATCTCCCTCC
IGKV11-125*01_CBA	CBA/J	IGKV	MUSMUS IGKV11-125*01 F	100	GATGTCCAGATGATTCAGTCTCCATCCTCCCTGTCTGCATCTTTGGGAGACATAGTCACCATGACTTGCCAGGCAAGTCAGGGCACTAGCATTAATTTAAACTGGTTTCAGCAAAAACCAGGGAAAGCTCCTAAGCTCCTGATCTATGGTGCAAGCAACTTGGAAGATGGGGTCCCATCAAGGTTCAGTGGCAGTAGATATGGGACAGATTTCACTCTCACCATCAGCAGCCTGGAGGATGAAGATATGGCAACTTATTTCTGTCTACAGCATAGTTATCTCCCTCC
IGKV11-125*01_DBA1	DBA/1J	IGKV	MUSMUS IGKV11-125*01 F	100	GATGTCCAGATGATTCAGTCTCCATCCTCCCTGTCTGCATCTTTGGGAGACATAGTCACCATGACTTGCCAGGCAAGTCAGGGCACTAGCATTAATTTAAACTGGTTTCAGCAAAAACCAGGGAAAGCTCCTAAGCTCCTGATCTATGGTGCAAGCAACTTGGAAGATGGGGTCCCATCAAGGTTCAGTGGCAGTAGATATGGGACAGATTTCACTCTCACCATCAGCAGCCTGGAGGATGAAGATATGGCAACTTATTTCTGTCTACAGCATAGTTATCTCCCTCC

IGKV11-125*01_DBA2	DBA/2J	IGKV	MUSMUS IGKV11-125*01 F	100	GATGTCCAGATGATTCAGTCTCCATCCTCCCTGTCTGCATCTTTGGGAGACATAGTCACCATGACTTGCCAGGCAAGTCAGGGCACTAGCATTAATTTAAACTGGTTTCAGCAAAAACCAGGGAAAGCTCCTAAGCTCCTGATCTATGGTGCAAGCAACTTGGAAGATGGGGTCCCATCAAGGTTCAGTGGCAGTAGATATGGGACAGATTTCACTCTCACCATCAGCAGCCTGGAGGATGAAGATATGGCAACTTATTTCTGTCTACAGCATAGTTATCTCCCTCC
IGKV11-125*01_LEWES	LEWES/EIJ	IGKV	MUSMUS IGKV11-125*01 F	100	GATGTCCAGATGATTCAGTCTCCATCCTCCCTGTCTGCATCTTTGGGAGACATAGTCACCATGACTTGCCAGGCAAGTCAGGGCACTAGCATTAATTTAAACTGGTTTCAGCAAAAACCAGGGAAAGCTCCTAAGCTCCTGATCTATGGTGCAAGCAACTTGGAAGATGGGGTCCCATCAAGGTTCAGTGGCAGTAGATATGGGACAGATTTCACTCTCACCATCAGCAGCCTGGAGGATGAAGATATGGCAACTTATTTCTGTCTACAGCATAGTTATCTCCCTCC
IGKV11-125*01_MRL	MRL/MPJ	IGKV	MUSMUS IGKV11-125*01 F	100	GATGTCCAGATGATTCAGTCTCCATCCTCCCTGTCTGCATCTTTGGGAGACATAGTCACCATGACTTGCCAGGCAAGTCAGGGCACTAGCATTAATTTAAACTGGTTTCAGCAAAAACCAGGGAAAGCTCCTAAGCTCCTGATCTATGGTGCAAGCAACTTGGAAGATGGGGTCCCATCAAGGTTCAGTGGCAGTAGATATGGGACAGATTTCACTCTCACCATCAGCAGCCTGGAGGATGAAGATATGGCAACTTATTTCTGTCTACAGCATAGTTATCTCCCTCC
IGKV11-125*01_MSM	MSM/MSJ	IGKV	MUSMUS IGKV11-125*01 F	100	GATGTCCAGATGATTCAGTCTCCATCCTCCCTGTCTGCATCTTTGGGAGACATAGTCACCATGACTTGCCAGGCAAGTCAGGGCACTAGCATTAATTTAAACTGGTTTCAGCAAAAACCAGGGAAAGCTCCTAAGCTCCTGATCTATGGTGCAAGCAACTTGGAAGATGGGGTCCCATCAAGGTTCAGTGGCAGTAGATATGGGACAGATTTCACTCTCACCATCAGCAGCCTGGAGGATGAAGATATGGCAACTTATTTCTGTCTACAGCATAGTTATCTCCCTCC

IGKV11-125*01_NOR	NOR/LTJ	IGKV	MUSMUS IGKV11-125*01 F	100	GATGTCCAGATGATTCAGTCTCCATCCTCCCTGTCTGCATCTTTGGGAGACATAGTCACCATGACTTGCCAGGCAAGTCAGGGCACTAGCATTAATTTAAACTGGTTTCAGCAAAAACCAGGGAAAGCTCCTAAGCTCCTGATCTATGGTGCAAGCAACTTGGAAGATGGGGTCCCATCAAGGTTCAGTGGCAGTAGATATGGGACAGATTTCACTCTCACCATCAGCAGCCTGGAGGATGAAGATATGGCAACTTATTTCTGTCTACAGCATAGTTATCTCCCTCC
IGKV11-125*01_NZB	NZB/BLNJ	IGKV	MUSMUS IGKV11-125*01 F	100	GATGTCCAGATGATTCAGTCTCCATCCTCCCTGTCTGCATCTTTGGGAGACATAGTCACCATGACTTGCCAGGCAAGTCAGGGCACTAGCATTAATTTAAACTGGTTTCAGCAAAAACCAGGGAAAGCTCCTAAGCTCCTGATCTATGGTGCAAGCAACTTGGAAGATGGGGTCCCATCAAGGTTCAGTGGCAGTAGATATGGGACAGATTTCACTCTCACCATCAGCAGCCTGGAGGATGAAGATATGGCAACTTATTTCTGTCTACAGCATAGTTATCTCCCTCC
IGKV11-125*01_PWD	PWD/PHJ	IGKV	MUSMUS IGKV11-125*01 F	100	GATGTCCAGATGATTCAGTCTCCATCCTCCCTGTCTGCATCTTTGGGAGACATAGTCACCATGACTTGCCAGGCAAGTCAGGGCACTAGCATTAATTTAAACTGGTTTCAGCAAAAACCAGGGAAAGCTCCTAAGCTCCTGATCTATGGTGCAAGCAACTTGGAAGATGGGGTCCCATCAAGGTTCAGTGGCAGTAGATATGGGACAGATTTCACTCTCACCATCAGCAGCCTGGAGGATGAAGATATGGCAACTTATTTCTGTCTACAGCATAGTTATCTCCCTCC
IGKV11-125*01_S0249_CAST	CAST/EIJ	IGKV	MUSMUS IGKV11-106*02 F	96.77	GATGTTCAAATGACCCAGTCTCCATCCTCCCTGTCTGCATCTTTGGGAGAGAGAGTCTCCCTGACTTGCCAGGCAAGTCAGGGCATTAGCAATTATTTAAACTGGTATCAGCAAACACCAGGGAAAGCTCCTAGGCTCTTGATCTATGGTGCAAGCAACTTGGAAGATGGGGTCCCTTCAAGGTTCAGTGGCACTGGATATGGGACAGATTTCACTTTCACCATCAGCAGCCTGGAGGAAGAAGATGTGGCAACTTATTTCTGTCTACAGCATGGTTATCTCCCTCC

IGKV11-125*01_S0413_CAST	CAST/EIJ	IGKV	MUSMUS IGKV11-106*02 F	97.13	GATGTTCAAATGACCCAGTCTCCATCCTCCCTGTCTGCATCTTTGGGAGAGAGAGTCTCCCTGACTTGCCAGGCAAGTCAGAGCGTTAGCAATAATTTAAACTGGTATCAGCAAACACCAGGGAAAGCTCCTAGGCTCTTGATCTATGGTGCAAGCAAATTGGAAGATGGGGTCCCTTCAAGGTTCAGTGGCACTGGATATGGGACAGATTTCACTTTCACCATCAGCAGCCTGGAGGAAGAAGATGTGGCAACTTATTTCTGCCTACAGCATAGGTATCTCCCTCC
IGKV11-125*01_S1984_MSM	MSM/MSJ	IGKV	MUSMUS IGKV11-106*02 F	96.06	GATGTTCAAATGACCCAGTCTCCATCCTCCCTGTCTGCATCTTTGGGAGAGAGAGTCTCCCTGACTTGCCAGGCAAGTCAGAGCATTAGCAATTATTTAAACTGGTTTCAGCAAACACCAGGGAAAGCTCCTAGGCTCTTGATCTATGATGCAAACAACTTGGAAGATGGGGTCCCTTCAAGGTTCAGTGGCACTGGATATGGGACAGATTTCACTTTCACCATCAGCAGCCTGGAGGAAGAAGATGTCGCAACTTATTTCTGTCTACAGCATAAGTATCTCCCTC
IGKV11-125*01_S1984_PWD	PWD/PHJ	IGKV	MUSMUS IGKV11-106*02 F	96.06	GATGTTCAAATGACCCAGTCTCCATCCTCCCTGTCTGCATCTTTGGGAGAGAGAGTCTCCCTGACTTGCCAGGCAAGTCAGAGCATTAGCAATTATTTAAACTGGTTTCAGCAAACACCAGGGAAAGCTCCTAGGCTCTTGATCTATGATGCAAACAACTTGGAAGATGGGGTCCCTTCAAGGTTCAGTGGCACTGGATATGGGACAGATTTCACTTTCACCATCAGCAGCCTGGAGGAAGAAGATGTCGCAACTTATTTCTGTCTACAGCATAAGTATCTCCCTC
IGKV11-125*01_S3440_LEWES	LEWES/EIJ	IGKV	MUSMUS IGKV11-106*02 F	96.06	GATGTTCAAATGACCCAGTCTCCATCCTCCCTGTCTGCATCTTTGGGAGAGAGAGTCTCCCTGACTTGCCAGGCAAGTCAGAGCGTTAGCAATTATTTAAACTGGTATCAGCAAACACCAGGGAAAGCTCCTAGGCTCTTGATCTATGGTGCAAGCAAATTGGAAGATAGGGTCCCTTCAAGGTTCAGTGGAACTGGATATGGGACAGATTTCACTTTCACCATCAGAAGCCTGGAGGAAGAAGATGTGGCAACTTATTTCTGTCTACAGCATAGGTATCTCCCTCC

IGKV11-125*01_S3701_LEWES	LEWES/EIJ	IGKV	MUSMUS IGKV11-106*02 F	96.06	GATGTTCAAATGACCCAGTCTCCATCCTCCCCGTCTGCATCTTTGGGAGAGAGAGTCTCCCTGACTTGCCAGGCAAGTCAGGACATTAGCAATTATTTAAACTGGTATCAGCAAACACCAGGGAAAGCTCCTAGGCTCTTGATCTATGGTGCAAGCAACTTGGAAGATGGGGTCCCTTCAAGGTTCAGTGGCACTGGATATGGGACAGATTTCACTTTCACCATCAGCAGCCTGGAGGAAGAAGATGTGGCAACTTATTTCTGTCTACAGCATGGTTATCTCCCTCC
IGKV11-125*01_S3707_LEWES	LEWES/EIJ	IGKV	MUSMUS IGKV11-106*02 F	96.42	AATGTTCAAATGACCCAGTCTCCATCCTCCCTGTCTGCATCTTTGGGAGAGAGAGTCTCCCTGACTTGCCAGGCAAGACAGAGCGTTAGCAATAATTTAAACTGGTATCAGCAAACACCAGGGAAAGCTCCTAGGCTCTTGATCTATGGTGCAAGCAAATTGGAAGATGGGGTCCCTTCAAGGTTCAGTGGCACTGGATATGGGACAGATTTCACTTTCACCATCAGCAGCCTGGAGGAAGAAGATGTGGCAACTTATTTCTGTCTACAGCATAGTTATCTCCCTCC
IGKV11-125*01_S4527_CAST	CAST/EIJ	IGKV	MUSMUS IGKV11-106*02 F	96.06	AATGTTCAAATGACCCAGTCTCCATCCTCCCTGTCTGCATCTTTGGGAGAGAGAGTCTCCCTGACTTGCCAGGCAAGTCAGAGCGTTAGCAATAATTTAAACTGGTATCAGCAAACACCAGGGAAAGCTCCTAGGCTCTTGATCTATGGTGCAAGCAAATTGGAAGATGGGGTCCCTTCAAGGTTCAGTGGCACTGGATATGGGACAGATTTCACTTTCACCATCAGCAGCCTGGAGGAAGAAGATGTGGCAATTTATTTCTGTCTACAGGATAGTTATCTCCCTC
IGKV11-125*01_S8154_NZB	NZB/BLNJ	IGKV	MUSMUS IGKV11-106*02 F	95.34	GATGTTCAAATGACCCAGTCTCCATCCTCCCTGTCTGCATCTTTGGGAGAAAGAGTCTCCCTGACCTGCCAGGCAAGTCAGAGCATTAGCAATTATTTAAACTGGTATCAGCAAACACTAGGGAAAGCTGCTAGGCTCTTGATCTATGGTGCAAGCAAATTGGAAGATGGGGTCCCTTCAAGGTTCAGTGGAACTGGATATGGGACAGATTTCACTTTCACCATCAGCAGCCAGGAGGAAGAAGATGTGGCAACTTATTTCTGTCTACAGCATAGGTATCTCCCTCC

IGKV11-125*01_SJL	SJL/J	IGKV	MUSMUS IGKV11-125*01 F	100	GATGTCCAGATGATTCAGTCTCCATCCTCCCTGTCTGCATCTTTGGGAGACATAGTCACCATGACTTGCCAGGCAAGTCAGGGCACTAGCATTAATTTAAACTGGTTTCAGCAAAAACCAGGGAAAGCTCCTAAGCTCCTGATCTATGGTGCAAGCAACTTGGAAGATGGGGTCCCATCAAGGTTCAGTGGCAGTAGATATGGGACAGATTTCACTCTCACCATCAGCAGCCTGGAGGATGAAGATATGGCAACTTATTTCTGTCTACAGCATAGTTATCTCCCTCC
IGKV12-38*01_129	129S1/SVIMJ	IGKV	MUSMUS IGKV12-38*01 F	100	GACATCCAGATGACTCAGTCTCCAGCCTCCCTGGCTGCATCTGTGGGAGAAACTGTCACCATCACATGTCGAGCAAGTGAGAACATTTACTACAGTTTAGCATGGTATCAGCAGAAGCAAGGGAAATCTCCTCAGCTCCTGATCTATAATGCAAACAGCTTGGAAGATGGTGTCCCATCGAGGTTCAGTGGCAGTGGATCTGGGACACAGTATTCTATGAAGATCAACAGCATGCAGCCTGAAGATACCGCAACTTATTTCTGTAAACAGGCTTATGACGTTCCTCC
IGKV12-38*01_AJ	A/J	IGKV	MUSMUS IGKV12-38*01 F	100	GACATCCAGATGACTCAGTCTCCAGCCTCCCTGGCTGCATCTGTGGGAGAAACTGTCACCATCACATGTCGAGCAAGTGAGAACATTTACTACAGTTTAGCATGGTATCAGCAGAAGCAAGGGAAATCTCCTCAGCTCCTGATCTATAATGCAAACAGCTTGGAAGATGGTGTCCCATCGAGGTTCAGTGGCAGTGGATCTGGGACACAGTATTCTATGAAGATCAACAGCATGCAGCCTGAAGATACCGCAACTTATTTCTGTAAACAGGCTTATGACGTTCCTCC
IGKV12-38*01_B6	C57BL/6J	IGKV	MUSMUS IGKV12-38*01 F	100	GACATCCAGATGACTCAGTCTCCAGCCTCCCTGGCTGCATCTGTGGGAGAAACTGTCACCATCACATGTCGAGCAAGTGAGAACATTTACTACAGTTTAGCATGGTATCAGCAGAAGCAAGGGAAATCTCCTCAGCTCCTGATCTATAATGCAAACAGCTTGGAAGATGGTGTCCCATCGAGGTTCAGTGGCAGTGGATCTGGGACACAGTATTCTATGAAGATCAACAGCATGCAGCCTGAAGATACCGCAACTTATTTCTGTAAACAGGCTTATGACGTTCCTCC

IGKV12-38*01_BALB	BALB/CBYJ	IGKV	MUSMUS IGKV12-38*01 F	100	GACATCCAGATGACTCAGTCTC<br>CAGCCTCCCTGGCTGCATCTGT<br>GGGAGAAACTGTCACCATCACA<br>TGTCGAGCAAGTGAGAACATTTA<br>CTACAGTTTAGCATGGTATCAGC<br>AGAAGCAAGGGAAATCTCCTCA<br>GCTCCTGATCTATAATGCAAACA<br>GCTTGGAAGATGGTGTCCCATC<br>GAGGTTCAGTGGCAGTGGATCT<br>GGGACACAGTATTCTATGAAGAT<br>CAACAGCATGCAGCCTGAAGAT<br>ACCGCAACTTATTTCTGTAAACA<br>GGCTTATGACGTTCCTCC
IGKV12-38*01_C3H	C3H/HEJ	IGKV	MUSMUS IGKV12-38*01 F	100	GACATCCAGATGACTCAGTCTC<br>CAGCCTCCCTGGCTGCATCTGT<br>GGGAGAAACTGTCACCATCACA<br>TGTCGAGCAAGTGAGAACATTTA<br>CTACAGTTTAGCATGGTATCAGC<br>AGAAGCAAGGGAAATCTCCTCA<br>GCTCCTGATCTATAATGCAAACA<br>GCTTGGAAGATGGTGTCCCATC<br>GAGGTTCAGTGGCAGTGGATCT<br>GGGACACAGTATTCTATGAAGAT<br>CAACAGCATGCAGCCTGAAGAT<br>ACCGCAACTTATTTCTGTAAACA<br>GGCTTATGACGTTCCTCC
IGKV12-38*01_CBA	CBA/J	IGKV	MUSMUS IGKV12-38*01 F	100	GACATCCAGATGACTCAGTCTC<br>CAGCCTCCCTGGCTGCATCTGT<br>GGGAGAAACTGTCACCATCACA<br>TGTCGAGCAAGTGAGAACATTTA<br>CTACAGTTTAGCATGGTATCAGC<br>AGAAGCAAGGGAAATCTCCTCA<br>GCTCCTGATCTATAATGCAAACA<br>GCTTGGAAGATGGTGTCCCATC<br>GAGGTTCAGTGGCAGTGGATCT<br>GGGACACAGTATTCTATGAAGAT<br>CAACAGCATGCAGCCTGAAGAT<br>ACCGCAACTTATTTCTGTAAACA<br>GGCTTATGACGTTCCTCC
IGKV12-38*01_DBA1	DBA/1J	IGKV	MUSMUS IGKV12-38*01 F	100	GACATCCAGATGACTCAGTCTC<br>CAGCCTCCCTGGCTGCATCTGT<br>GGGAGAAACTGTCACCATCACA<br>TGTCGAGCAAGTGAGAACATTTA<br>CTACAGTTTAGCATGGTATCAGC<br>AGAAGCAAGGGAAATCTCCTCA<br>GCTCCTGATCTATAATGCAAACA<br>GCTTGGAAGATGGTGTCCCATC<br>GAGGTTCAGTGGCAGTGGATCT<br>GGGACACAGTATTCTATGAAGAT<br>CAACAGCATGCAGCCTGAAGAT<br>ACCGCAACTTATTTCTGTAAACA<br>GGCTTATGACGTTCCTCC

IGKV12-38*01_DBA2	DBA/2J	IGKV	MUSMUS IGKV12-38*01 F	100	GACATCCAGATGACTCAGTCTC<br>CAGCCTCCCTGGCTGCATCTGT<br>GGGAGAAACTGTCACCATCACA<br>TGTCGAGCAAGTGAGAACATTTA<br>CTACAGTTTAGCATGGTATCAGC<br>AGAAGCAAGGGAAATCTCCTCA<br>GCTCCTGATCTATAATGCAAACA<br>GCTTGGAAGATGGTGTCCCATC<br>GAGGTTCAGTGGCAGTGGATCT<br>GGGACACAGTATTCTATGAAGAT<br>CAACAGCATGCAGCCTGAAGAT<br>ACCGCAACTTATTTCTGTAAACA<br>GGCTTATGACGTTCCTCC
IGKV12-38*01_LEWES	LEWES/EIJ	IGKV	MUSMUS IGKV12-38*01 F	100	GACATCCAGATGACTCAGTCTC<br>CAGCCTCCCTGGCTGCATCTGT<br>GGGAGAAACTGTCACCATCACA<br>TGTCGAGCAAGTGAGAACATTTA<br>CTACAGTTTAGCATGGTATCAGC<br>AGAAGCAAGGGAAATCTCCTCA<br>GCTCCTGATCTATAATGCAAACA<br>GCTTGGAAGATGGTGTCCCATC<br>GAGGTTCAGTGGCAGTGGATCT<br>GGGACACAGTATTCTATGAAGAT<br>CAACAGCATGCAGCCTGAAGAT<br>ACCGCAACTTATTTCTGTAAACA<br>GGCTTATGACGTTCCTCC
IGKV12-38*01_NZB	NZB/BLNJ	IGKV	MUSMUS IGKV12-38*01 F	100	GACATCCAGATGACTCAGTCTC<br>CAGCCTCCCTGGCTGCATCTGT<br>GGGAGAAACTGTCACCATCACA<br>TGTCGAGCAAGTGAGAACATTTA<br>CTACAGTTTAGCATGGTATCAGC<br>AGAAGCAAGGGAAATCTCCTCA<br>GCTCCTGATCTATAATGCAAACA<br>GCTTGGAAGATGGTGTCCCATC<br>GAGGTTCAGTGGCAGTGGATCT<br>GGGACACAGTATTCTATGAAGAT<br>CAACAGCATGCAGCCTGAAGAT<br>ACCGCAACTTATTTCTGTAAACA<br>GGCTTATGACGTTCCTCC
IGKV12-38*01_S2611_AKR	AKR/J	IGKV	MUSMUS IGKV12-38*01 F	98.21	GACATCCAGATGACTCAGTCTC<br>CAGCCTCCCTGGCTGCATCTGT<br>GGGAGAAACCATCACCATCACA<br>TGTCAAGCAAGTGAGAACATTTA<br>CTTCAGTTTAGCATGGTATCAGC<br>AGAAGCAAGGGAAATCTCCTCA<br>GCTCCTGATCTATAATGCAAACA<br>GCTTGGAAGATGGTGTCCCATC<br>GAGGTTCAGTGGCAGTGGATCT<br>GGGACACAGTATTCTATGAAGAT<br>CAACAGCATGCAGCCTGAAGAT<br>ACTGCAACTTATTTCTGTAAACA<br>GGCTTATGACTTTCCTCC

IGKV12-38*01_S2611_CAST	CAST/EIJ	IGKV	MUSMUS IGKV12-38*01 F	98.21	GACATCCAGATGACTCAGTCTCCAGCCTCCCTGGCTGCATCTGTGGGAGAAACCATCACCATCACATGTCAAGCAAGTGAGAACATTTACTTCAGTTTAGCATGGTATCAGCAGAAGCAAGGGAAATCTCCTCAGCTCCTGATCTATAATGCAAACAGCTTGGAAGATGGTGTCCCATCGAGGTTCAGTGGCAGTGGATCTGGGACACAGTATTCTATGAAGATCAACAGCATGCAGCCTGAAGATACTGCAACTTATTTCTGTAAACAGGCTTATGACTTTCCTCC
IGKV12-38*01_S2611_MRL	MRL/MPJ	IGKV	MUSMUS IGKV12-38*01 F	98.21	GACATCCAGATGACTCAGTCTCCAGCCTCCCTGGCTGCATCTGTGGGAGAAACCATCACCATCACATGTCAAGCAAGTGAGAACATTTACTTCAGTTTAGCATGGTATCAGCAGAAGCAAGGGAAATCTCCTCAGCTCCTGATCTATAATGCAAACAGCTTGGAAGATGGTGTCCCATCGAGGTTCAGTGGCAGTGGATCTGGGACACAGTATTCTATGAAGATCAACAGCATGCAGCCTGAAGATACTGCAACTTATTTCTGTAAACAGGCTTATGACTTTCCTCC
IGKV12-38*01_S2611_NOR	NOR/LTJ	IGKV	MUSMUS IGKV12-38*01 F	98.21	GACATCCAGATGACTCAGTCTCCAGCCTCCCTGGCTGCATCTGTGGGAGAAACCATCACCATCACATGTCAAGCAAGTGAGAACATTTACTTCAGTTTAGCATGGTATCAGCAGAAGCAAGGGAAATCTCCTCAGCTCCTGATCTATAATGCAAACAGCTTGGAAGATGGTGTCCCATCGAGGTTCAGTGGCAGTGGATCTGGGACACAGTATTCTATGAAGATCAACAGCATGCAGCCTGAAGATACTGCAACTTATTTCTGTAAACAGGCTTATGACTTTCCTCC
IGKV12-38*01_S3762_MSM	MSM/MSJ	IGKV	MUSMUS IGKV12-38*01 F	98.57	GACATCCAGATGACTCAGTCTCCAGCCTCCCTGGCTGCATCTGTGGGAGAAACTATCACCATCACATGTCAAGCAAGTGAGAACATTTACTACAGTTTAGCATGGTATCAGCAGAAGCAAGGGAAATCTCCTCAGCTCCTGATCTATAATGCAAACAGCTTGGAAGATGGTGTCCCATCAAGGTTCAGTGGCAGTGGATCTGGGACACAGTATTCTATGAAGATCAACAGCATGCAGCCTGAAGATACTGCAACTTATTTCTGTAAACAGGCTTATGACTTTCCTCC

IGKV12-38*01_S6950_PWD	PWD/PHJ	IGKV	MUSMUS IGKV12-38*01 F	98.57	GACATCCAGATGACTCAGTCTC<br>CAGCCTCCCTGGCTGCATCTGT<br>GGGAGAAACCATCACCATCACA<br>TGTCAAGCAAGTGAGAACATTTA<br>CTACAGTTTAGCATGGTATCAGC<br>AGAAGCAAGGGAAATCTCCTCA<br>GCTCCTGATCTATAATGCAAACA<br>GCTTGGAAGATGGTGTCCCATC<br>GAGGTTCAGTGGCAGTGGATCT<br>GGGACACAGTATTCTATGAAGAT<br>CAACAGCATGCAGCCTGAAGAT<br>ACTGCAACTTATTTCTGTAAACA<br>GGCTTATGACTTTCCTCC
IGKV12-38*01_SJL	SJL/J	IGKV	MUSMUS IGKV12-38*01 F	100	GACATCCAGATGACTCAGTCTC<br>CAGCCTCCCTGGCTGCATCTGT<br>GGGAGAAACTGTCACCATCACA<br>TGTCGAGCAAGTGAGAACATTTA<br>CTACAGTTTAGCATGGTATCAGC<br>AGAAGCAAGGGAAATCTCCTCA<br>GCTCCTGATCTATAATGCAAACA<br>GCTTGGAAGATGGTGTCCCATC<br>GAGGTTCAGTGGCAGTGGATCT<br>GGGACACAGTATTCTATGAAGAT<br>CAACAGCATGCAGCCTGAAGAT<br>ACCGCAACTTATTTCTGTAAACA<br>GGCTTATGACGTTCCTCC
IGKV12-40*01_S1894_MSM	MSM/MSJ	IGKV	MUSMUS IGKV12-40*01 P	98.57	GACATCCAGATGACTCAGTCTC<br>CAGCCTCCCTATCTGCATCTGT<br>GGGAGAAACTGTCACCATCACA<br>TGTCAAGCAAGTGAGAATATTGC<br>CAGTGATTTAGCATGGTATCAGC<br>AGAAACAGGGAAAATCTCCTCA<br>GCTCCTGGTCTATGATGCGAGA<br>AACTTAGCAGATGGTGTGCCAT<br>CAAGGTTCAGTGGCAGTGGATC<br>AGGCACACAGTATTCTCTCAATA<br>TCAACAGCCTGCAGTCTGAAGA<br>TGTTGCGAGATATTACTGTCAAC<br>ATTATTATGGTACTCCTCC
IGKV12-40*01_S1894_PWD	PWD/PHJ	IGKV	MUSMUS IGKV12-40*01 P	98.57	GACATCCAGATGACTCAGTCTC<br>CAGCCTCCCTATCTGCATCTGT<br>GGGAGAAACTGTCACCATCACA<br>TGTCAAGCAAGTGAGAATATTGC<br>CAGTGATTTAGCATGGTATCAGC<br>AGAAACAGGGAAAATCTCCTCA<br>GCTCCTGGTCTATGATGCGAGA<br>AACTTAGCAGATGGTGTGCCAT<br>CAAGGTTCAGTGGCAGTGGATC<br>AGGCACACAGTATTCTCTCAATA<br>TCAACAGCCTGCAGTCTGAAGA<br>TGTTGCGAGATATTACTGTCAAC<br>ATTATTATGGTACTCCTCC

IGKV12-40*01_S2386_CAST	CAST/EIJ	IGKV	MUSMUS IGKV12-40*01 P	97.49	GACATCCAGATGACTCAGTCTCCAGCCTCCCTGTCTGCATCTGTGGGAGAAACTGTCACCATCACATGTCGAGCAAGTGAGAATATTGCCAGTAATTTAGCATGGTATCAGCAGAAACAGGGAAAATCTCCTCAGCTCCTGGTCTATGATGCGAGAAACTTAGCAGATGGTGTGCCATCAAGGTTCAGTGGCAGTGGATCAGGCACACAGTATTCTCTCAATATCAACAGCCTGCAGTCTGAAGATGTTGCGAGATATTACTGTCAACATTATTATGGTACTCCTCC
IGKV12-40*01_S9191_NOR	NOR/LTJ	IGKV	MUSMUS IGKV12-40*01 P, OR MUSMUS IGKV12-46*01 F	93.91	GACATCCAGATGACTCAGTCTCCAGCTTCCCTGTCTGCATCTGTGGGAGAAACTGTCACCATCACATGTCGAGCAAGTGAGAATATTGACAGTTATTTAGCATGGTATCAGCAGAAACAGGGAAAATCTCCTCAGCTCCTGGTCTATGTTGCAACACTCTTAGCAGATGGTGTGCCATCAAGGTTCAGTGGCAGTGGATCAGGCACACAGTATTCTCTCAAGATCAACAGCCTGCAGTCTGAAGATGTTGCGAGATATTACTGTCAGCATTATTATAGTACTCCTC
IGKV12-41*01_129	129S1/SVIMJ	IGKV	MUSMUS IGKV12-41*01 F	100	GACATCCAGATGACTCAGTCTCCAGCCTCCCTATCTGCATCTGTGGGAGAAACTGTCACCATCACATGTCGAGCAAGTGGGAATATTCACAATTATTTAGCATGGTATCAGCAGAAACAGGGAAAATCTCCTCAGCTCCTGGTCTATAATGCAAAAACCTTAGCAGATGGTGTGCCATCAAGGTTCAGTGGCAGTGGATCAGGAACACAATATTCTCTCAAGATCAACAGCCTGCAGCCTGAAGATTTTGGGAGTTATTACTGTCAACATTTTTGGAGTACTCCTCC
IGKV12-41*01_AJ	A/J	IGKV	MUSMUS IGKV12-41*01 F	100	GACATCCAGATGACTCAGTCTCCAGCCTCCCTATCTGCATCTGTGGGAGAAACTGTCACCATCACATGTCGAGCAAGTGGGAATATTCACAATTATTTAGCATGGTATCAGCAGAAACAGGGAAAATCTCCTCAGCTCCTGGTCTATAATGCAAAAACCTTAGCAGATGGTGTGCCATCAAGGTTCAGTGGCAGTGGATCAGGAACACAATATTCTCTCAAGATCAACAGCCTGCAGCCTGAAGATTTTGGGAGTTATTACTGTCAACATTTTTGGAGTACTCCTCC

IGKV12-41*01_B6	C57BL/6J	IGKV	MUSMUS IGKV12-41*01 F	100	GACATCCAGATGACTCAGTCTCCAGCCTCCCTATCTGCATCTGTGGGAGAAACTGTCACCATCACATGTCGAGCAAGTGGGAATATTCACAATTATTTAGCATGGTATCAGCAGAAACAGGGAAAATCTCCTCAGCTCCTGGTCTATAATGCAAAAACCTTAGCAGATGGTGTGCCATCAAGGTTCAGTGGCAGTGGATCAGGAACACAATATTCTCTCAAGATCAACAGCCTGCAGCCTGAAGATTTTGGGAGTTATTACTGTCAACATTTTTGGAGTACTCCTCC
IGKV12-41*01_BALB	BALB/CBYJ	IGKV	MUSMUS IGKV12-41*01 F	100	GACATCCAGATGACTCAGTCTCCAGCCTCCCTATCTGCATCTGTGGGAGAAACTGTCACCATCACATGTCGAGCAAGTGGGAATATTCACAATTATTTAGCATGGTATCAGCAGAAACAGGGAAAATCTCCTCAGCTCCTGGTCTATAATGCAAAAACCTTAGCAGATGGTGTGCCATCAAGGTTCAGTGGCAGTGGATCAGGAACACAATATTCTCTCAAGATCAACAGCCTGCAGCCTGAAGATTTTGGGAGTTATTACTGTCAACATTTTTGGAGTACTCCTCC
IGKV12-41*01_C3H	C3H/HEJ	IGKV	MUSMUS IGKV12-41*01 F	100	GACATCCAGATGACTCAGTCTCCAGCCTCCCTATCTGCATCTGTGGGAGAAACTGTCACCATCACATGTCGAGCAAGTGGGAATATTCACAATTATTTAGCATGGTATCAGCAGAAACAGGGAAAATCTCCTCAGCTCCTGGTCTATAATGCAAAAACCTTAGCAGATGGTGTGCCATCAAGGTTCAGTGGCAGTGGATCAGGAACACAATATTCTCTCAAGATCAACAGCCTGCAGCCTGAAGATTTTGGGAGTTATTACTGTCAACATTTTTGGAGTACTCCTCC
IGKV12-41*01_CBA	CBA/J	IGKV	MUSMUS IGKV12-41*01 F	100	GACATCCAGATGACTCAGTCTCCAGCCTCCCTATCTGCATCTGTGGGAGAAACTGTCACCATCACATGTCGAGCAAGTGGGAATATTCACAATTATTTAGCATGGTATCAGCAGAAACAGGGAAAATCTCCTCAGCTCCTGGTCTATAATGCAAAAACCTTAGCAGATGGTGTGCCATCAAGGTTCAGTGGCAGTGGATCAGGAACACAATATTCTCTCAAGATCAACAGCCTGCAGCCTGAAGATTTTGGGAGTTATTACTGTCAACATTTTTGGAGTACTCCTCC

IGKV12-41*01_DBA1	DBA/1J	IGKV	MUSMUS IGKV12-41*01 F	100	GACATCCAGATGACTCAGTCTCCAGCCTCCCTATCTGCATCTGTGGGAGAAACTGTCACCATCACATGTCGAGCAAGTGGGAATATTCACAATTATTTAGCATGGTATCAGCAGAAACAGGGAAAATCTCCTCAGCTCCTGGTCTATAATGCAAAAACCTTAGCAGATGGTGTGCCATCAAGGTTCAGTGGCAGTGGATCAGGAACACAATATTCTCTCAAGATCAACAGCCTGCAGCCTGAAGATTTTGGGAGTTATTACTGTCAACATTTTTGGAGTACTCCTCC
IGKV12-41*01_DBA2	DBA/2J	IGKV	MUSMUS IGKV12-41*01 F	100	GACATCCAGATGACTCAGTCTCCAGCCTCCCTATCTGCATCTGTGGGAGAAACTGTCACCATCACATGTCGAGCAAGTGGGAATATTCACAATTATTTAGCATGGTATCAGCAGAAACAGGGAAAATCTCCTCAGCTCCTGGTCTATAATGCAAAAACCTTAGCAGATGGTGTGCCATCAAGGTTCAGTGGCAGTGGATCAGGAACACAATATTCTCTCAAGATCAACAGCCTGCAGCCTGAAGATTTTGGGAGTTATTACTGTCAACATTTTTGGAGTACTCCTCC
IGKV12-41*01_LEWES	LEWES/EIJ	IGKV	MUSMUS IGKV12-41*01 F	100	GACATCCAGATGACTCAGTCTCCAGCCTCCCTATCTGCATCTGTGGGAGAAACTGTCACCATCACATGTCGAGCAAGTGGGAATATTCACAATTATTTAGCATGGTATCAGCAGAAACAGGGAAAATCTCCTCAGCTCCTGGTCTATAATGCAAAAACCTTAGCAGATGGTGTGCCATCAAGGTTCAGTGGCAGTGGATCAGGAACACAATATTCTCTCAAGATCAACAGCCTGCAGCCTGAAGATTTTGGGAGTTATTACTGTCAACATTTTTGGAGTACTCCTCC
IGKV12-41*01_NZB	NZB/BLNJ	IGKV	MUSMUS IGKV12-41*01 F	100	GACATCCAGATGACTCAGTCTCCAGCCTCCCTATCTGCATCTGTGGGAGAAACTGTCACCATCACATGTCGAGCAAGTGGGAATATTCACAATTATTTAGCATGGTATCAGCAGAAACAGGGAAAATCTCCTCAGCTCCTGGTCTATAATGCAAAAACCTTAGCAGATGGTGTGCCATCAAGGTTCAGTGGCAGTGGATCAGGAACACAATATTCTCTCAAGATCAACAGCCTGCAGCCTGAAGATTTTGGGAGTTATTACTGTCAACATTTTTGGAGTACTCCTCC

IGKV12-41*01_S5467_MSM	MSM/MSJ	IGKV	MUSMUS IGKV12-41*01 F	96.42	GACATCCAGATGACTCAGTCTCCAGCTTCCCTATCTGCATCTGTGGGAGAAACTGTCACCATCACATGTCGAGCAAGTGGGAATATTTACAGTAATTTAGCATGGTATCAGCAGAAACAGGGAAAATCTCCTCAGCTCCTGGTCTATAATGCAAAAACCTTAGCAGAAGGTGTGCCATCAAGGTTCAGTGGCAGTGGATCAGGAACACAATATTCTCTCAAGATCAACAGCCTACAGCCTGAAGATTTTGGGAGTTATTACTGTCAACATTATTATAATACTCC
IGKV12-41*01_S6800_AKR	AKR/J	IGKV	MUSMUS IGKV12-41*01 F	97.13	GATATCCAGATGACTCAGTCTCCAGCCTCCCTATCTGCATCTGTGGGAGAAACTGTCACCATCACATGTCGAGCAAGTGGGAATATTCACAATTATTTAGCATGGTATCAGCAGAAACAGGGAAAATCTCCTCAGCTCCTGGTCTATAATGCAAAAACCTTAGCGGAAGGTGTGCCATCAAGGTTCAGTGGCAGTGGATCAGGAACACAATATTCTCTCAAGATCAACAGCCTGCAGCCTGAGGATTTTGGGAGTTATTACTGTCATCATTATTATAGTACTCCTCC
IGKV12-41*01_S6800_MRL	MRL/MPJ	IGKV	MUSMUS IGKV12-41*01 F	97.13	GATATCCAGATGACTCAGTCTCCAGCCTCCCTATCTGCATCTGTGGGAGAAACTGTCACCATCACATGTCGAGCAAGTGGGAATATTCACAATTATTTAGCATGGTATCAGCAGAAACAGGGAAAATCTCCTCAGCTCCTGGTCTATAATGCAAAAACCTTAGCGGAAGGTGTGCCATCAAGGTTCAGTGGCAGTGGATCAGGAACACAATATTCTCTCAAGATCAACAGCCTGCAGCCTGAGGATTTTGGGAGTTATTACTGTCATCATTATTATAGTACTCCTCC
IGKV12-41*01_S6800_NOD	NOD/SHILTJ	IGKV	MUSMUS IGKV12-41*01 F	97.13	GATATCCAGATGACTCAGTCTCCAGCCTCCCTATCTGCATCTGTGGGAGAAACTGTCACCATCACATGTCGAGCAAGTGGGAATATTCACAATTATTTAGCATGGTATCAGCAGAAACAGGGAAAATCTCCTCAGCTCCTGGTCTATAATGCAAAAACCTTAGCGGAAGGTGTGCCATCAAGGTTCAGTGGCAGTGGATCAGGAACACAATATTCTCTCAAGATCAACAGCCTGCAGCCTGAGGATTTTGGGAGTTATTACTGTCATCATTATTATAGTACTCCTCC

IGKV12-41*01_S6800_NOR	NOR/LTJ	IGKV	MUSMUS IGKV12-41*01 F	97.13	GATATCCAGATGACTCAGTCTCCAGCCTCCCTATCTGCATCTGTGGGAGAAACTGTCACCATCACATGTCGAGCAAGTGGGAATATTCACAATTATTTAGCATGGTATCAGCAGAAACAGGGAAAATCTCCTCAGCTCCTGGTCTATAATGCAAAAACCTTAGCGGAAGGTGTGCCATCAAGGTTCAGTGGCAGTGGATCAGGAACACAATATTCTCTCAAGATCAACAGCCTGCAGCCTGAGGATTTTGGGAGTTATTACTGTCATCATTATTATAGTACTCCTCC
IGKV12-41*01_SJL	SJL/J	IGKV	MUSMUS IGKV12-41*01 F	100	GACATCCAGATGACTCAGTCTCCAGCCTCCCTATCTGCATCTGTGGGAGAAACTGTCACCATCACATGTCGAGCAAGTGGGAATATTCACAATTATTTAGCATGGTATCAGCAGAAACAGGGAAAATCTCCTCAGCTCCTGGTCTATAATGCAAAAACCTTAGCAGATGGTGTGCCATCAAGGTTCAGTGGCAGTGGATCAGGAACACAATATTCTCTCAAGATCAACAGCCTGCAGCCTGAAGATTTTGGGAGTTATTACTGTCAACATTTTTGGAGTACTCCTCC
IGKV12-44*01_129	129S1/SVIMJ	IGKV	MUSMUS IGKV12-44*01 F	100	GACATCCAGATGACTCAGTCTCCAGCCTCCCTATCTGCATCTGTGGGAGAAACTGTCACCATCACATGTCGAGCAAGTGAGAATATTTACAGTTATTTAGCATGGTATCAGCAGAAACAGGGAAAATCTCCTCAGCTCCTGGTCTATAATGCAAAAACCTTAGCAGAAGGTGTGCCATCAAGGTTCAGTGGCAGTGGATCAGGCACACAGTTTTCTCTGAAGATCAACAGCCTGCAGCCTGAAGATTTTGGGAGTTATTACTGTCAACATCATTATGGTACTCCTCC
IGKV12-44*01_AJ	A/J	IGKV	MUSMUS IGKV12-44*01 F	100	GACATCCAGATGACTCAGTCTCCAGCCTCCCTATCTGCATCTGTGGGAGAAACTGTCACCATCACATGTCGAGCAAGTGAGAATATTTACAGTTATTTAGCATGGTATCAGCAGAAACAGGGAAAATCTCCTCAGCTCCTGGTCTATAATGCAAAAACCTTAGCAGAAGGTGTGCCATCAAGGTTCAGTGGCAGTGGATCAGGCACACAGTTTTCTCTGAAGATCAACAGCCTGCAGCCTGAAGATTTTGGGAGTTATTACTGTCAACATCATTATGGTACTCCTCC

IGKV12-44*01_AKR	AKR/J	IGKV	MUSMUS IGKV12-44*01 F	100	GACATCCAGATGACTCAGTCTCCAGCCTCCCTATCTGCATCTGTGGGAGAAACTGTCACCATCACATGTCGAGCAAGTGAGAATATTTACAGTTATTTAGCATGGTATCAGCAGAAACAGGGAAAATCTCCTCAGCTCCTGGTCTATAATGCAAAAACCTTAGCAGAAGGTGTGCCATCAAGGTTCAGTGGCAGTGGATCAGGCACACAGTTTTCTCTGAAGATCAACAGCCTGCAGCCTGAAGATTTTGGGAGTTATTACTGTCAACATCATTATGGTACTCCTCC
IGKV12-44*01_B6	C57BL/6J	IGKV	MUSMUS IGKV12-44*01 F	100	GACATCCAGATGACTCAGTCTCCAGCCTCCCTATCTGCATCTGTGGGAGAAACTGTCACCATCACATGTCGAGCAAGTGAGAATATTTACAGTTATTTAGCATGGTATCAGCAGAAACAGGGAAAATCTCCTCAGCTCCTGGTCTATAATGCAAAAACCTTAGCAGAAGGTGTGCCATCAAGGTTCAGTGGCAGTGGATCAGGCACACAGTTTTCTCTGAAGATCAACAGCCTGCAGCCTGAAGATTTTGGGAGTTATTACTGTCAACATCATTATGGTACTCCTCC
IGKV12-44*01_BALB	BALB/CBYJ	IGKV	MUSMUS IGKV12-44*01 F	100	GACATCCAGATGACTCAGTCTCCAGCCTCCCTATCTGCATCTGTGGGAGAAACTGTCACCATCACATGTCGAGCAAGTGAGAATATTTACAGTTATTTAGCATGGTATCAGCAGAAACAGGGAAAATCTCCTCAGCTCCTGGTCTATAATGCAAAAACCTTAGCAGAAGGTGTGCCATCAAGGTTCAGTGGCAGTGGATCAGGCACACAGTTTTCTCTGAAGATCAACAGCCTGCAGCCTGAAGATTTTGGGAGTTATTACTGTCAACATCATTATGGTACTCCTCC
IGKV12-44*01_C3H	C3H/HEJ	IGKV	MUSMUS IGKV12-44*01 F	100	GACATCCAGATGACTCAGTCTCCAGCCTCCCTATCTGCATCTGTGGGAGAAACTGTCACCATCACATGTCGAGCAAGTGAGAATATTTACAGTTATTTAGCATGGTATCAGCAGAAACAGGGAAAATCTCCTCAGCTCCTGGTCTATAATGCAAAAACCTTAGCAGAAGGTGTGCCATCAAGGTTCAGTGGCAGTGGATCAGGCACACAGTTTTCTCTGAAGATCAACAGCCTGCAGCCTGAAGATTTTGGGAGTTATTACTGTCAACATCATTATGGTACTCCTCC

IGKV12-44*01_CBA	CBA/J	IGKV	MUSMUS IGKV12-44*01 F	100	GACATCCAGATGACTCAGTCTCCAGCCTCCCTATCTGCATCTGTGGGAGAAACTGTCACCATCACATGTCGAGCAAGTGAGAATATTTACAGTTATTTAGCATGGTATCAGCAGAAACAGGGAAAATCTCCTCAGCTCCTGGTCTATAATGCAAAAACCTTAGCAGAAGGTGTGCCATCAAGGTTCAGTGGCAGTGGATCAGGCACACAGTTTTCTCTGAAGATCAACAGCCTGCAGCCTGAAGATTTTGGGAGTTATTACTGTCAACATCATTATGGTACTCCTCC
IGKV12-44*01_DBA1	DBA/1J	IGKV	MUSMUS IGKV12-44*01 F	100	GACATCCAGATGACTCAGTCTCCAGCCTCCCTATCTGCATCTGTGGGAGAAACTGTCACCATCACATGTCGAGCAAGTGAGAATATTTACAGTTATTTAGCATGGTATCAGCAGAAACAGGGAAAATCTCCTCAGCTCCTGGTCTATAATGCAAAAACCTTAGCAGAAGGTGTGCCATCAAGGTTCAGTGGCAGTGGATCAGGCACACAGTTTTCTCTGAAGATCAACAGCCTGCAGCCTGAAGATTTTGGGAGTTATTACTGTCAACATCATTATGGTACTCCTCC
IGKV12-44*01_DBA2	DBA/2J	IGKV	MUSMUS IGKV12-44*01 F	100	GACATCCAGATGACTCAGTCTCCAGCCTCCCTATCTGCATCTGTGGGAGAAACTGTCACCATCACATGTCGAGCAAGTGAGAATATTTACAGTTATTTAGCATGGTATCAGCAGAAACAGGGAAAATCTCCTCAGCTCCTGGTCTATAATGCAAAAACCTTAGCAGAAGGTGTGCCATCAAGGTTCAGTGGCAGTGGATCAGGCACACAGTTTTCTCTGAAGATCAACAGCCTGCAGCCTGAAGATTTTGGGAGTTATTACTGTCAACATCATTATGGTACTCCTCC
IGKV12-44*01_LEWES	LEWES/EIJ	IGKV	MUSMUS IGKV12-44*01 F	100	GACATCCAGATGACTCAGTCTCCAGCCTCCCTATCTGCATCTGTGGGAGAAACTGTCACCATCACATGTCGAGCAAGTGAGAATATTTACAGTTATTTAGCATGGTATCAGCAGAAACAGGGAAAATCTCCTCAGCTCCTGGTCTATAATGCAAAAACCTTAGCAGAAGGTGTGCCATCAAGGTTCAGTGGCAGTGGATCAGGCACACAGTTTTCTCTGAAGATCAACAGCCTGCAGCCTGAAGATTTTGGGAGTTATTACTGTCAACATCATTATGGTACTCCTCC

IGKV12-44*01_MRL	MRL/MPJ	IGKV	MUSMUS IGKV12-44*01 F	100	GACATCCAGATGACTCAGTCTC<br>CAGCCTCCCTATCTGCATCTGT<br>GGGAGAAACTGTCACCATCACA<br>TGTCGAGCAAGTGAGAATATTTA<br>CAGTTATTTAGCATGGTATCAGC<br>AGAAACAGGGAAAATCTCCTCA<br>GCTCCTGGTCTATAATGCAAAAA<br>CCTTAGCAGAAGGTGTGCCATC<br>AAGGTTCAGTGGCAGTGGATCA<br>GGCACACAGTTTTCTCTGAAGAT<br>CAACAGCCTGCAGCCTGAAGAT<br>TTTGGGAGTTATTACTGTCAACAT<br>CATTATGGTACTCCTCC
IGKV12-44*01_MSM	MSM/MSJ	IGKV	MUSMUS IGKV12-44*01 F	100	GACATCCAGATGACTCAGTCTC<br>CAGCCTCCCTATCTGCATCTGT<br>GGGAGAAACTGTCACCATCACA<br>TGTCGAGCAAGTGAGAATATTTA<br>CAGTTATTTAGCATGGTATCAGC<br>AGAAACAGGGAAAATCTCCTCA<br>GCTCCTGGTCTATAATGCAAAAA<br>CCTTAGCAGAAGGTGTGCCATC<br>AAGGTTCAGTGGCAGTGGATCA<br>GGCACACAGTTTTCTCTGAAGAT<br>CAACAGCCTGCAGCCTGAAGAT<br>TTTGGGAGTTATTACTGTCAACAT<br>CATTATGGTACTCCTCC
IGKV12-44*01_NOD	NOD/SHILTJ	IGKV	MUSMUS IGKV12-44*01 F	100	GACATCCAGATGACTCAGTCTC<br>CAGCCTCCCTATCTGCATCTGT<br>GGGAGAAACTGTCACCATCACA<br>TGTCGAGCAAGTGAGAATATTTA<br>CAGTTATTTAGCATGGTATCAGC<br>AGAAACAGGGAAAATCTCCTCA<br>GCTCCTGGTCTATAATGCAAAAA<br>CCTTAGCAGAAGGTGTGCCATC<br>AAGGTTCAGTGGCAGTGGATCA<br>GGCACACAGTTTTCTCTGAAGAT<br>CAACAGCCTGCAGCCTGAAGAT<br>TTTGGGAGTTATTACTGTCAACAT<br>CATTATGGTACTCCTCC
IGKV12-44*01_NOR	NOR/LTJ	IGKV	MUSMUS IGKV12-44*01 F	100	GACATCCAGATGACTCAGTCTC<br>CAGCCTCCCTATCTGCATCTGT<br>GGGAGAAACTGTCACCATCACA<br>TGTCGAGCAAGTGAGAATATTTA<br>CAGTTATTTAGCATGGTATCAGC<br>AGAAACAGGGAAAATCTCCTCA<br>GCTCCTGGTCTATAATGCAAAAA<br>CCTTAGCAGAAGGTGTGCCATC<br>AAGGTTCAGTGGCAGTGGATCA<br>GGCACACAGTTTTCTCTGAAGAT<br>CAACAGCCTGCAGCCTGAAGAT<br>TTTGGGAGTTATTACTGTCAACAT<br>CATTATGGTACTCCTCC

IGKV12-44*01_NZB	NZB/BLNJ	IGKV	MUSMUS IGKV12-44*01 F	100	GACATCCAGATGACTCAGTCTCCAGCCTCCCTATCTGCATCTGTGGGAGAAACTGTCACCATCACATGTCGAGCAAGTGAGAATATTTACAGTTATTTAGCATGGTATCAGCAGAAACAGGGAAAATCTCCTCAGCTCCTGGTCTATAATGCAAAAACCTTAGCAGAAGGTGTGCCATCAAGGTTCAGTGGCAGTGGATCAGGCACACAGTTTTCTCTGAAGATCAACAGCCTGCAGCCTGAAGATTTTGGGAGTTATTACTGTCAACATCATTATGGTACTCCTCC
IGKV12-44*01_PWD	PWD/PHJ	IGKV	MUSMUS IGKV12-44*01 F	100	GACATCCAGATGACTCAGTCTCCAGCCTCCCTATCTGCATCTGTGGGAGAAACTGTCACCATCACATGTCGAGCAAGTGAGAATATTTACAGTTATTTAGCATGGTATCAGCAGAAACAGGGAAAATCTCCTCAGCTCCTGGTCTATAATGCAAAAACCTTAGCAGAAGGTGTGCCATCAAGGTTCAGTGGCAGTGGATCAGGCACACAGTTTTCTCTGAAGATCAACAGCCTGCAGCCTGAAGATTTTGGGAGTTATTACTGTCAACATCATTATGGTACTCCTCC
IGKV12-44*01_S1769_CAST	CAST/EIJ	IGKV	MUSMUS IGKV12-44*01 F	97.13	GACATCCAGATGACTCAGTCTCCAGCTTCCCTATCTGCATCTGTGGGAGAAACTGTCACCATCACATGTCGAGCAAGTGAGAATATTTACAGTAATTTAGCCTGGTATCAGCAGAAACAGGGAAAATCTCCTCAGCTCCTGGTCTATAATGCAAAAACCTTAGCAGAAGGTGTGCCATCAAGGTTCAGTGGCAGTGGATCAGGAACACAATATTCTCTCAAGATCAACAGCCTGCAGCCTGAAGATTTTGGGAGTTATTACTGTCAACATTATTATGGTACTCCTCC
IGKV12-44*01_S2900_PWD	PWD/PHJ	IGKV	MUSMUS IGKV12-44*01 F	96.77	GACATCCAGATGACTCAGTCTCCAGCTTCCCTATCTGCATCTGTGGGAGAAACTGTCACCATCACATGTCGAGCAACTGGGAATATTTACAGTAATTTAGCATGGTATCAGCAGAAACAGGGAAAATCTCCTCAGCTCCTGGTCTATAATGCAAAAACCTTAGCAGAAGGTGTGCCATCAAGGTTCAGTGGCAGTGGATCAGGAACACAATATTCTCTCAAGATCAACAGCCTGCAGCCTGAAGATTTTGGGAGTTATTACTGTCAACATTATTATGGTACTCCTCC

IGKV12-44*01_S4418_SJL	SJL/J	IGKV	MUSMUS IGKV12-44*01 F	100	GACATCCAGATGACTCAGTCTCCAGCCTCCCTATCTGCATCTGTGGGAGAAACTGTCACCATCACATGTCGAGCAAGTGAGAATATTTACAGTTATTTAGCATGGTATCAGCAGAAACAGGGAAAATCTCCTCAGCTCCTGGTCTATAATGCAAAAACCTTAGCAGAAGGTGTGCCATCAAGGTTCAGTGGCAGTGGATCAGGCACACAGTTTTCTCTGAAGATCAACAGCCTGCAGCCTGAAGATTTTGGGAGTTATTACTGTCAACATCATTATGGTACTCCTCCT
IGKV12-44*01_S9406_MSM	MSM/MSJ	IGKV	MUSMUS IGKV12-44*01 F	96.77	GACATCCAGATGACTCAGTCTCCAGCTTCCCTATCTGCATCTGTGGGAGAAACTGTCACCATCACATGTCGAGCAAGTGGGAATATTTACAGTAATTTAGCATGGTATCAGCAGAAACAGGGAAAATCTCCTCAGCTCCTGGTCTATAATGCAAAAACCTTAGCAGAAGGTGTGCCATCAAGGTTCAGTGGCAGTGGATCAGGAACACAATATTCTCTCAAGATCAACAGCCTACAGCCTGAAGATTTTGGGAGTTATTACTGTCAACATTATTATGGTACTCCTCC
IGKV12-46*01_129	129S1/SVIMJ	IGKV	MUSMUS IGKV12-46*01 F	100	GACATCCAGATGACTCAGTCTCCAGCCTCCCTATCTGTATCTGTGGGAGAAACTGTCACCATCACATGTCGAGCAAGTGAGAATATTTACAGTAATTTAGCATGGTATCAGCAGAAACAGGGAAAATCTCCTCAGCTCCTGGTCTATGCTGCAACAAACTTAGCAGATGGTGTGCCATCAAGGTTCAGTGGCAGTGGATCAGGCACACAGTATTCCCTCAAGATCAACAGCCTGCAGTCTGAAGATTTTGGGAGTTATTACTGTCAACATTTTTGGGGTACTCCTCC
IGKV12-46*01_AJ	A/J	IGKV	MUSMUS IGKV12-46*01 F	100	GACATCCAGATGACTCAGTCTCCAGCCTCCCTATCTGTATCTGTGGGAGAAACTGTCACCATCACATGTCGAGCAAGTGAGAATATTTACAGTAATTTAGCATGGTATCAGCAGAAACAGGGAAAATCTCCTCAGCTCCTGGTCTATGCTGCAACAAACTTAGCAGATGGTGTGCCATCAAGGTTCAGTGGCAGTGGATCAGGCACACAGTATTCCCTCAAGATCAACAGCCTGCAGTCTGAAGATTTTGGGAGTTATTACTGTCAACATTTTTGGGGTACTCCTCC

IGKV12-46*01_B6	C57BL/6J	IGKV	MUSMUS IGKV12-46*01 F	100	GACATCCAGATGACTCAGTCTCCAGCCTCCCTATCTGTATCTGTGGGAGAAACTGTCACCATCACATGTCGAGCAAGTGAGAATATTTACAGTAATTTAGCATGGTATCAGCAGAAACAGGGAAAATCTCCTCAGCTCCTGGTCTATGCTGCAACAAACTTAGCAGATGGTGTGCCATCAAGGTTCAGTGGCAGTGGATCAGGCACACAGTATTCCCTCAAGATCAACAGCCTGCAGTCTGAAGATTTTGGGAGTTATTACTGTCAACATTTTTGGGGTACTCCTCC
IGKV12-46*01_BALB	BALB/CBYJ	IGKV	MUSMUS IGKV12-46*01 F	100	GACATCCAGATGACTCAGTCTCCAGCCTCCCTATCTGTATCTGTGGGAGAAACTGTCACCATCACATGTCGAGCAAGTGAGAATATTTACAGTAATTTAGCATGGTATCAGCAGAAACAGGGAAAATCTCCTCAGCTCCTGGTCTATGCTGCAACAAACTTAGCAGATGGTGTGCCATCAAGGTTCAGTGGCAGTGGATCAGGCACACAGTATTCCCTCAAGATCAACAGCCTGCAGTCTGAAGATTTTGGGAGTTATTACTGTCAACATTTTTGGGGTACTCCTCC
IGKV12-46*01_C3H	C3H/HEJ	IGKV	MUSMUS IGKV12-46*01 F	100	GACATCCAGATGACTCAGTCTCCAGCCTCCCTATCTGTATCTGTGGGAGAAACTGTCACCATCACATGTCGAGCAAGTGAGAATATTTACAGTAATTTAGCATGGTATCAGCAGAAACAGGGAAAATCTCCTCAGCTCCTGGTCTATGCTGCAACAAACTTAGCAGATGGTGTGCCATCAAGGTTCAGTGGCAGTGGATCAGGCACACAGTATTCCCTCAAGATCAACAGCCTGCAGTCTGAAGATTTTGGGAGTTATTACTGTCAACATTTTTGGGGTACTCCTCC
IGKV12-46*01_CBA	CBA/J	IGKV	MUSMUS IGKV12-46*01 F	100	GACATCCAGATGACTCAGTCTCCAGCCTCCCTATCTGTATCTGTGGGAGAAACTGTCACCATCACATGTCGAGCAAGTGAGAATATTTACAGTAATTTAGCATGGTATCAGCAGAAACAGGGAAAATCTCCTCAGCTCCTGGTCTATGCTGCAACAAACTTAGCAGATGGTGTGCCATCAAGGTTCAGTGGCAGTGGATCAGGCACACAGTATTCCCTCAAGATCAACAGCCTGCAGTCTGAAGATTTTGGGAGTTATTACTGTCAACATTTTTGGGGTACTCCTCC

IGKV12-46*01_DBA1	DBA/1J	IGKV	MUSMUS IGKV12-46*01 F	100	GACATCCAGATGACTCAGTCTCCAGCCTCCCTATCTGTATCTGTGGGAGAAACTGTCACCATCACATGTCGAGCAAGTGAGAATATTTACAGTAATTTAGCATGGTATCAGCAGAAACAGGGAAAATCTCCTCAGCTCCTGGTCTATGCTGCAACAAACTTAGCAGATGGTGTGCCATCAAGGTTCAGTGGCAGTGGATCAGGCACACAGTATTCCCTCAAGATCAACAGCCTGCAGTCTGAAGATTTTGGGAGTTATTACTGTCAACATTTTTGGGGTACTCCTCC
IGKV12-46*01_DBA2	DBA/2J	IGKV	MUSMUS IGKV12-46*01 F	100	GACATCCAGATGACTCAGTCTCCAGCCTCCCTATCTGTATCTGTGGGAGAAACTGTCACCATCACATGTCGAGCAAGTGAGAATATTTACAGTAATTTAGCATGGTATCAGCAGAAACAGGGAAAATCTCCTCAGCTCCTGGTCTATGCTGCAACAAACTTAGCAGATGGTGTGCCATCAAGGTTCAGTGGCAGTGGATCAGGCACACAGTATTCCCTCAAGATCAACAGCCTGCAGTCTGAAGATTTTGGGAGTTATTACTGTCAACATTTTTGGGGTACTCCTCC
IGKV12-46*01_LEWES	LEWES/EIJ	IGKV	MUSMUS IGKV12-46*01 F	100	GACATCCAGATGACTCAGTCTCCAGCCTCCCTATCTGTATCTGTGGGAGAAACTGTCACCATCACATGTCGAGCAAGTGAGAATATTTACAGTAATTTAGCATGGTATCAGCAGAAACAGGGAAAATCTCCTCAGCTCCTGGTCTATGCTGCAACAAACTTAGCAGATGGTGTGCCATCAAGGTTCAGTGGCAGTGGATCAGGCACACAGTATTCCCTCAAGATCAACAGCCTGCAGTCTGAAGATTTTGGGAGTTATTACTGTCAACATTTTTGGGGTACTCCTCC
IGKV12-46*01_NZB	NZB/BLNJ	IGKV	MUSMUS IGKV12-46*01 F	100	GACATCCAGATGACTCAGTCTCCAGCCTCCCTATCTGTATCTGTGGGAGAAACTGTCACCATCACATGTCGAGCAAGTGAGAATATTTACAGTAATTTAGCATGGTATCAGCAGAAACAGGGAAAATCTCCTCAGCTCCTGGTCTATGCTGCAACAAACTTAGCAGATGGTGTGCCATCAAGGTTCAGTGGCAGTGGATCAGGCACACAGTATTCCCTCAAGATCAACAGCCTGCAGTCTGAAGATTTTGGGAGTTATTACTGTCAACATTTTTGGGGTACTCCTCC

IGKV12-46*01_S1254_AKR	AKR/J	IGKV	MUSMUS IGKV12-46*01 F	94.62	GACATCCAGATGACTCAGTCTCCAGCTTCCCTGTCTGCATCTGTGGGAGAAACTGTCACCATCACATGTCGAGCAAGTGAGAATATTGACAGTTATTTAGCATGGTATCAGCAGAAACAGGGAAAATCTCCTCAGCTCCTGGTCTATGCTGCAACACTCTTAGCAGATGGTGTGCCATCAAGGTTCAGTGGCAGTGGATCAGGCACACAGTATTCTCTCAAGATCAACAGCCTGCAGTCTGAAGATGTTGCGAGATATTACTGTCAACATTATTATAGTACTCCTCC
IGKV12-46*01_S1254_MRL	MRL/MPJ	IGKV	MUSMUS IGKV12-46*01 F	94.62	GACATCCAGATGACTCAGTCTCCAGCTTCCCTGTCTGCATCTGTGGGAGAAACTGTCACCATCACATGTCGAGCAAGTGAGAATATTGACAGTTATTTAGCATGGTATCAGCAGAAACAGGGAAAATCTCCTCAGCTCCTGGTCTATGCTGCAACACTCTTAGCAGATGGTGTGCCATCAAGGTTCAGTGGCAGTGGATCAGGCACACAGTATTCTCTCAAGATCAACAGCCTGCAGTCTGAAGATGTTGCGAGATATTACTGTCAACATTATTATAGTACTCCTCC
IGKV12-46*01_S1254_NOD	NOD/SHILTJ	IGKV	MUSMUS IGKV12-46*01 F	94.62	GACATCCAGATGACTCAGTCTCCAGCTTCCCTGTCTGCATCTGTGGGAGAAACTGTCACCATCACATGTCGAGCAAGTGAGAATATTGACAGTTATTTAGCATGGTATCAGCAGAAACAGGGAAAATCTCCTCAGCTCCTGGTCTATGCTGCAACACTCTTAGCAGATGGTGTGCCATCAAGGTTCAGTGGCAGTGGATCAGGCACACAGTATTCTCTCAAGATCAACAGCCTGCAGTCTGAAGATGTTGCGAGATATTACTGTCAACATTATTATAGTACTCCTCC
IGKV12-46*01_S1254_NOR	NOR/LTJ	IGKV	MUSMUS IGKV12-46*01 F	94.62	GACATCCAGATGACTCAGTCTCCAGCTTCCCTGTCTGCATCTGTGGGAGAAACTGTCACCATCACATGTCGAGCAAGTGAGAATATTGACAGTTATTTAGCATGGTATCAGCAGAAACAGGGAAAATCTCCTCAGCTCCTGGTCTATGCTGCAACACTCTTAGCAGATGGTGTGCCATCAAGGTTCAGTGGCAGTGGATCAGGCACACAGTATTCTCTCAAGATCAACAGCCTGCAGTCTGAAGATGTTGCGAGATATTACTGTCAACATTATTATAGTACTCCTCC

IGKV12-46*01_S2465_CAST	CAST/EIJ	IGKV	MUSMUS IGKV12-46*01 F	97.13	GACATCCAGATGACTCAGTCTC<br>CAGCCTCCCTATCTGCATCTGT<br>AGGAGAAACTGTCACCATCACA<br>TGTCGAGCAAGTGAGAATATTTA<br>CAGTTATTTAGCCTGGTATCAGC<br>AGAAACAGGGAAAATCTCCTCA<br>GCTCCTGGTCTATGCTGCAACA<br>AACTTAGCAGATGGTGTGCCAT<br>CAAGGTTCAGTGGCAGTGGATC<br>AGGCACACAGTATTCCCTCAAG<br>ATCAACAGCCTGCAGCCTGAAG<br>ATTTTGGGAGTTATTACTGTCAAC<br>ATTATTATGGTACTCCTCC
IGKV12-46*01_S2796_PWD	PWD/PHJ	IGKV	MUSMUS IGKV12-46*01 F	98.21	GACATCCAGATGACTCAGTCTC<br>CAGCCTCCCTATCTGCATCTGT<br>AGGAGAAACTGTCACCATCACA<br>TGTCGAGCAAGTGAGAATATTTA<br>CAGTTATTTAGCATGGTATCAGC<br>AGAAACAGGGAAAATCTCCTCA<br>GCTCCTGGTCTATGCTGCAACA<br>AACTTAGCAGATGGTGTGCCAT<br>CAAGGTTCAGTGGCAGTGGATC<br>AGGCACACAGTATTCCCTCAAG<br>ATCAACAGCCTGCAGCCTGAAG<br>ATTTTGGGAGTTATTACTGTCAAC<br>ATTTTTTGGGTACTCCTCC
IGKV12-46*01_S4026_AKR	AKR/J	IGKV	MUSMUS IGKV12-46*01 F	99.64	GACATCCAGATGACTCAGTCTC<br>CAGCCTCCCTATCTGTATCTGTG<br>GGAGAAACTGTCACCATCACAT<br>GTCGAGCAAGTGAGAATATTTAC<br>AGTAATTTAGCATGGTATCAGCA<br>GAAACAGGGAAAATCTCCTCAG<br>CTCCTGGTCTATGCTGCAACAA<br>ACTTAGCAGATGGTGTTCCATCA<br>AGGTTCAGTGGCAGTGGATCAG<br>GCACACAGTATTCCCTCAAGAT<br>CAACAGCCTGCAGTCTGAAGAT<br>TTTGGGAGTTATTACTGTCAACAT<br>TTTTGGGGTACTCCTCC
IGKV12-46*01_S4026_MRL	MRL/MPJ	IGKV	MUSMUS IGKV12-46*01 F	99.64	GACATCCAGATGACTCAGTCTC<br>CAGCCTCCCTATCTGTATCTGTG<br>GGAGAAACTGTCACCATCACAT<br>GTCGAGCAAGTGAGAATATTTAC<br>AGTAATTTAGCATGGTATCAGCA<br>GAAACAGGGAAAATCTCCTCAG<br>CTCCTGGTCTATGCTGCAACAA<br>ACTTAGCAGATGGTGTTCCATCA<br>AGGTTCAGTGGCAGTGGATCAG<br>GCACACAGTATTCCCTCAAGAT<br>CAACAGCCTGCAGTCTGAAGAT<br>TTTGGGAGTTATTACTGTCAACAT<br>TTTTGGGGTACTCCTCC

IGKV12-46*01_S4026_NOD	NOD/SHILTJ	IGKV	MUSMUS IGKV12-46*01 F	99.64	GACATCCAGATGACTCAGTCTCCAGCCTCCCTATCTGTATCTGTGGGAGAAACTGTCACCATCACATGTCGAGCAAGTGAGAATATTTACAGTAATTTAGCATGGTATCAGCAGAAACAGGGAAAATCTCCTCAGCTCCTGGTCTATGCTGCAACAAACTTAGCAGATGGTGTTCCATCAAGGTTCAGTGGCAGTGGATCAGGCACACAGTATTCCCTCAAGATCAACAGCCTGCAGTCTGAAGATTTTGGGAGTTATTACTGTCAACATTTTTGGGGTACTCCTCC
IGKV12-46*01_S4026_NOR	NOR/LTJ	IGKV	MUSMUS IGKV12-46*01 F	99.64	GACATCCAGATGACTCAGTCTCCAGCCTCCCTATCTGTATCTGTGGGAGAAACTGTCACCATCACATGTCGAGCAAGTGAGAATATTTACAGTAATTTAGCATGGTATCAGCAGAAACAGGGAAAATCTCCTCAGCTCCTGGTCTATGCTGCAACAAACTTAGCAGATGGTGTTCCATCAAGGTTCAGTGGCAGTGGATCAGGCACACAGTATTCCCTCAAGATCAACAGCCTGCAGTCTGAAGATTTTGGGAGTTATTACTGTCAACATTTTTGGGGTACTCCTCC
IGKV12-46*01_S4541_AKR	AKR/J	IGKV	MUSMUS IGKV12-46*01 F	97.13	GACATCCAGATGACTCAGTCTCCAGCCTCCCTATCTGCATCTGTGGGAGAAACTGTCACCATCACATGTCGAGCAAGTGAGAATATTTACAGTAATTTAGCATGGTATCAGCAGAAACAGGGAAAATCTCCTCAGCTCCTGGTCTATGCTGCAACAAATTTAGCAGATGGTGTGCCATCAAGGTTCAGTGGCAGTGGATCAGGCACACAGTTTTCTCTGAAGATCAACAGCCTGCAGCCTGAAGATTTTGGGAGTTATTACTGTCAACATTTTTATGGTACTCCTCC
IGKV12-46*01_S4541_MRL	MRL/MPJ	IGKV	MUSMUS IGKV12-46*01 F	97.13	GACATCCAGATGACTCAGTCTCCAGCCTCCCTATCTGCATCTGTGGGAGAAACTGTCACCATCACATGTCGAGCAAGTGAGAATATTTACAGTAATTTAGCATGGTATCAGCAGAAACAGGGAAAATCTCCTCAGCTCCTGGTCTATGCTGCAACAAATTTAGCAGATGGTGTGCCATCAAGGTTCAGTGGCAGTGGATCAGGCACACAGTTTTCTCTGAAGATCAACAGCCTGCAGCCTGAAGATTTTGGGAGTTATTACTGTCAACATTTTTATGGTACTCCTCC

IGKV12-46*01_S4541_NOD	NOD/SHILTJ	IGKV	MUSMUS IGKV12-46*01 F	97.13	GACATCCAGATGACTCAGTCTCCAGCCTCCCTATCTGCATCTGTGGGAGAAACTGTCACCATCACATGTCGAGCAAGTGAGAATATTTACAGTAATTTAGCATGGTATCAGCAGAAACAGGGAAAATCTCCTCAGCTCCTGGTCTATGCTGCAACAAATTTAGCAGATGGTGTGCCATCAAGGTTCAGTGGCAGTGGATCAGGCACACAGTTTTCTCTGAAGATCAACAGCCTGCAGCCTGAAGATTTTGGGAGTTATTACTGTCAACATTTTTATGGTACTCCTCC
IGKV12-46*01_S4541_NOR	NOR/LTJ	IGKV	MUSMUS IGKV12-46*01 F	97.13	GACATCCAGATGACTCAGTCTCCAGCCTCCCTATCTGCATCTGTGGGAGAAACTGTCACCATCACATGTCGAGCAAGTGAGAATATTTACAGTAATTTAGCATGGTATCAGCAGAAACAGGGAAAATCTCCTCAGCTCCTGGTCTATGCTGCAACAAATTTAGCAGATGGTGTGCCATCAAGGTTCAGTGGCAGTGGATCAGGCACACAGTTTTCTCTGAAGATCAACAGCCTGCAGCCTGAAGATTTTGGGAGTTATTACTGTCAACATTTTTATGGTACTCCTCC
IGKV12-46*01_S7378_MSM	MSM/MSJ	IGKV	MUSMUS IGKV12-46*01 F	99.64	GACATCCAGATGACTCAGTCTCCAGCCTCCCTATCTGTATCTGTGGGAGAAACTGTCACCATCACATGTCGAGCAAGTGAGAATATTTACAGTAATTTAGCATGGTATCAGCAGAAACAGGGAAAATCTCCTCAGCTCCTGGTCTATGCTGCAACAAACTTAGCAGACGGTGTGCCATCAAGGTTCAGTGGCAGTGGATCAGGCACACAGTATTCCCTCAAGATCAACAGCCTGCAGTCTGAAGATTTTGGGAGTTATTACTGTCAACATTTTTGGGGTACTCCTCC
IGKV12-46*01_SJL	SJL/J	IGKV	MUSMUS IGKV12-46*01 F	100	GACATCCAGATGACTCAGTCTCCAGCCTCCCTATCTGTATCTGTGGGAGAAACTGTCACCATCACATGTCGAGCAAGTGAGAATATTTACAGTAATTTAGCATGGTATCAGCAGAAACAGGGAAAATCTCCTCAGCTCCTGGTCTATGCTGCAACAAACTTAGCAGATGGTGTGCCATCAAGGTTCAGTGGCAGTGGATCAGGCACACAGTATTCCCTCAAGATCAACAGCCTGCAGTCTGAAGATTTTGGGAGTTATTACTGTCAACATTTTTGGGGTACTCCTCC

IGKV12-89*01_129	129S1/SVIMJ	IGKV	MUSMUS IGKV12-89*01 F	100	GACATCCAGATGACTCAGTCTCCAGCTTCACTGTCTGCATCTGTGGGAGAAACTGTCACCATCACATGTGGAGCAAGTGAGAATATTTACGGTGCTTTAAATTGGTATCAGCGGAAACAGGGAAAATCTCCTCAGCTCCTGATCTATGGTGCAACCAACTTGGCAGATGGCATGTCATCGAGGTTCAGTGGCAGTGGATCTGGTAGACAGTATTCTCTCAAGATCAGTAGCCTGCATCCTGACGATGTTGCAACGTATTACTGTCAAAATGTGTTAAGTACTCCTCC
IGKV12-89*01_AJ	A/J	IGKV	MUSMUS IGKV12-89*01 F	100	GACATCCAGATGACTCAGTCTCCAGCTTCACTGTCTGCATCTGTGGGAGAAACTGTCACCATCACATGTGGAGCAAGTGAGAATATTTACGGTGCTTTAAATTGGTATCAGCGGAAACAGGGAAAATCTCCTCAGCTCCTGATCTATGGTGCAACCAACTTGGCAGATGGCATGTCATCGAGGTTCAGTGGCAGTGGATCTGGTAGACAGTATTCTCTCAAGATCAGTAGCCTGCATCCTGACGATGTTGCAACGTATTACTGTCAAAATGTGTTAAGTACTCCTCC
IGKV12-89*01_B6	C57BL/6J	IGKV	MUSMUS IGKV12-89*01 F	100	GACATCCAGATGACTCAGTCTCCAGCTTCACTGTCTGCATCTGTGGGAGAAACTGTCACCATCACATGTGGAGCAAGTGAGAATATTTACGGTGCTTTAAATTGGTATCAGCGGAAACAGGGAAAATCTCCTCAGCTCCTGATCTATGGTGCAACCAACTTGGCAGATGGCATGTCATCGAGGTTCAGTGGCAGTGGATCTGGTAGACAGTATTCTCTCAAGATCAGTAGCCTGCATCCTGACGATGTTGCAACGTATTACTGTCAAAATGTGTTAAGTACTCCTCC
IGKV12-89*01_BALB	BALB/CBYJ	IGKV	MUSMUS IGKV12-89*01 F	100	GACATCCAGATGACTCAGTCTCCAGCTTCACTGTCTGCATCTGTGGGAGAAACTGTCACCATCACATGTGGAGCAAGTGAGAATATTTACGGTGCTTTAAATTGGTATCAGCGGAAACAGGGAAAATCTCCTCAGCTCCTGATCTATGGTGCAACCAACTTGGCAGATGGCATGTCATCGAGGTTCAGTGGCAGTGGATCTGGTAGACAGTATTCTCTCAAGATCAGTAGCCTGCATCCTGACGATGTTGCAACGTATTACTGTCAAAATGTGTTAAGTACTCCTCC

IGKV12-89*01_C3H	C3H/HEJ	IGKV	MUSMUS IGKV12-89*01 F	100	GACATCCAGATGACTCAGTCTCCAGCTTCACTGTCTGCATCTGTGGGAGAAACTGTCACCATCACATGTGGAGCAAGTGAGAATATTTACGGTGCTTTAAATTGGTATCAGCGGAAACAGGGAAAATCTCCTCAGCTCCTGATCTATGGTGCAACCAACTTGGCAGATGGCATGTCATCGAGGTTCAGTGGCAGTGGATCTGGTAGACAGTATTCTCTCAAGATCAGTAGCCTGCATCCTGACGATGTTGCAACGTATTACTGTCAAAATGTGTTAAGTACTCCTCC
IGKV12-89*01_CBA	CBA/J	IGKV	MUSMUS IGKV12-89*01 F	100	GACATCCAGATGACTCAGTCTCCAGCTTCACTGTCTGCATCTGTGGGAGAAACTGTCACCATCACATGTGGAGCAAGTGAGAATATTTACGGTGCTTTAAATTGGTATCAGCGGAAACAGGGAAAATCTCCTCAGCTCCTGATCTATGGTGCAACCAACTTGGCAGATGGCATGTCATCGAGGTTCAGTGGCAGTGGATCTGGTAGACAGTATTCTCTCAAGATCAGTAGCCTGCATCCTGACGATGTTGCAACGTATTACTGTCAAAATGTGTTAAGTACTCCTCC
IGKV12-89*01_DBA1	DBA/1J	IGKV	MUSMUS IGKV12-89*01 F	100	GACATCCAGATGACTCAGTCTCCAGCTTCACTGTCTGCATCTGTGGGAGAAACTGTCACCATCACATGTGGAGCAAGTGAGAATATTTACGGTGCTTTAAATTGGTATCAGCGGAAACAGGGAAAATCTCCTCAGCTCCTGATCTATGGTGCAACCAACTTGGCAGATGGCATGTCATCGAGGTTCAGTGGCAGTGGATCTGGTAGACAGTATTCTCTCAAGATCAGTAGCCTGCATCCTGACGATGTTGCAACGTATTACTGTCAAAATGTGTTAAGTACTCCTCC
IGKV12-89*01_DBA2	DBA/2J	IGKV	MUSMUS IGKV12-89*01 F	100	GACATCCAGATGACTCAGTCTCCAGCTTCACTGTCTGCATCTGTGGGAGAAACTGTCACCATCACATGTGGAGCAAGTGAGAATATTTACGGTGCTTTAAATTGGTATCAGCGGAAACAGGGAAAATCTCCTCAGCTCCTGATCTATGGTGCAACCAACTTGGCAGATGGCATGTCATCGAGGTTCAGTGGCAGTGGATCTGGTAGACAGTATTCTCTCAAGATCAGTAGCCTGCATCCTGACGATGTTGCAACGTATTACTGTCAAAATGTGTTAAGTACTCCTCC

IGKV12-89*01_NZB	NZB/BLNJ	IGKV	MUSMUS IGKV12-89*01 F	100	GACATCCAGATGACTCAGTCTCCAGCTTCACTGTCTGCATCTGTGGGAGAAACTGTCACCATCACATGTGGAGCAAGTGAGAATATTTACGGTGCTTTAAATTGGTATCAGCGGAAACAGGGAAAATCTCCTCAGCTCCTGATCTATGGTGCAACCAACTTGGCAGATGGCATGTCATCGAGGTTCAGTGGCAGTGGATCTGGTAGACAGTATTCTCTCAAGATCAGTAGCCTGCATCCTGACGATGTTGCAACGTATTACTGTCAAAATGTGTTAAGTACTCCTCC
IGKV12-89*01_S6231_PWD	PWD/PHJ	IGKV	MUSMUS IGKV12-89*01 F	99.28	GACATCCAGATGACTCAGTCTCCAGCTTCACTGTCTGCATCTGTGGGAGAAAGTGTCTCCATCACATGTGGAGCAAGTGAGAATATTTACGGTGCTTTAAATTGGTATCAGCGGAAACAGGGAAAATCTCCTCAGCTCCTGATCTATGGTGCAACCAACTTGGCAGATGGCATGTCATCGAGGTTCAGTGGCAGTGGATCTGGTAGACAGTATTCTCTCAAGATCAGTAGCCTGCATCCTGACGATGTTGCAACGTATTACTGTCAAAATGTGTTAAGTACCCCTCC
IGKV12-89*01_S8177_MSM	MSM/MSJ	IGKV	MUSMUS IGKV12-89*01 F	98.92	GACATCCAGATGACTCAGTCTCCAGCTTCACTGTCTGCATCTGTGGGAGAAACTGTCACCATCACATGTGGAGCAAGTGAGAATATTTACGGTGCTTTAAATTGGTATCAGCAGAAACAGGGAAAATCTCCTCAGCTCCTGATCTATGGTGCAACCAACTTGGCAGATGGAATGTCATCGAGGTTCAGTGGTAGTGGATCTGGTAGACAGTATTCTCTCAAGATCAGTAGCCTGCATCCTGACGATGTTGCAACGTATTACTGTCAAAATGTGTTAAGTACCCCTCC
IGKV12-89*01_S9010_CAST	CAST/EIJ	IGKV	MUSMUS IGKV12-89*01 F	99.28	GACATCCAGATGACTCAGTCTCCAGCTTCACTGTCTGCATCTGTGGGAGAAACTGTCACCATCACATGTGGAGCAAGTGAGAATATTTACGGTGCTTTAAATTGGTATCAGCAGAAACAGGGAAAATCTCCTCAGCTCCTGATCTATGGTGCAACCAACTTGGCAGATGGAATGTCATCGAGGTTCAGTGGCAGTGGATCTGGTAGACAGTATTCTCTCAAGATCAGTAGCCTGCATCCTGACGATGTTGCAACGTATTACTGTCAAAATGTGTTAAGTACCCCTCC

IGKV12-89*01_SJL	SJL/J	IGKV	MUSMUS IGKV12-89*01 F	100	GACATCCAGATGACTCAGTCTCCAGCTTCACTGTCTGCATCTGTGGGAGAAACTGTCACCATCACATGTGGAGCAAGTGAGAATATTTACGGTGCTTTAAATTGGTATCAGCGGAAACAGGGAAAATCTCCTCAGCTCCTGATCTATGGTGCAACCAACTTGGCAGATGGCATGTCATCGAGGTTCAGTGGCAGTGGATCTGGTAGACAGTATTCTCTCAAGATCAGTAGCCTGCATCCTGACGATGTTGCAACGTATTACTGTCAAAATGTGTTAAGTACTCCTCC
IGKV12-98*01_129	129S1/SVIMJ	IGKV	MUSMUS IGKV12-98*01 F	100	GACATTCAGATGACCCAGTCTCCTGCCTCCCAGTCTGCATCTCTGGGAGAAAGTGTCACCATCACATGCCTGGCAAGTCAGACCATTGGTACATGGTTAGCATGGTATCAGCAGAAACCAGGGAAATCTCCTCAGCTCCTGATTTATGCTGCAACCAGCTTGGCAGATGGGGTCCCATCAAGGTTCAGTGGTAGTGGATCTGGCACAAAATTTTCTTTCAAGATCAGCAGCCTACAGGCTGAAGATTTTGTAAGTTATTACTGTCAACAACTTTACAGTACTCCTCT
IGKV12-98*01_AJ	A/J	IGKV	MUSMUS IGKV12-98*01 F	100	GACATTCAGATGACCCAGTCTCCTGCCTCCCAGTCTGCATCTCTGGGAGAAAGTGTCACCATCACATGCCTGGCAAGTCAGACCATTGGTACATGGTTAGCATGGTATCAGCAGAAACCAGGGAAATCTCCTCAGCTCCTGATTTATGCTGCAACCAGCTTGGCAGATGGGGTCCCATCAAGGTTCAGTGGTAGTGGATCTGGCACAAAATTTTCTTTCAAGATCAGCAGCCTACAGGCTGAAGATTTTGTAAGTTATTACTGTCAACAACTTTACAGTACTCCTCT
IGKV12-98*01_B6	C57BL/6J	IGKV	MUSMUS IGKV12-98*01 F	100	GACATTCAGATGACCCAGTCTCCTGCCTCCCAGTCTGCATCTCTGGGAGAAAGTGTCACCATCACATGCCTGGCAAGTCAGACCATTGGTACATGGTTAGCATGGTATCAGCAGAAACCAGGGAAATCTCCTCAGCTCCTGATTTATGCTGCAACCAGCTTGGCAGATGGGGTCCCATCAAGGTTCAGTGGTAGTGGATCTGGCACAAAATTTTCTTTCAAGATCAGCAGCCTACAGGCTGAAGATTTTGTAAGTTATTACTGTCAACAACTTTACAGTACTCCTCT

IGKV12-98*01_BALB	BALB/CBYJ	IGKV	MUSMUS IGKV12-98*01 F	100	GACATTCAGATGACCCAGTCTCCTGCCTCCCAGTCTGCATCTCTGGGAGAAAGTGTCACCATCACATGCCTGGCAAGTCAGACCATTGGTACATGGTTAGCATGGTATCAGCAGAAACCAGGGAAATCTCCTCAGCTCCTGATTTATGCTGCAACCAGCTTGGCAGATGGGGTCCCATCAAGGTTCAGTGGTAGTGGATCTGGCACAAAATTTTCTTTCAAGATCAGCAGCCTACAGGCTGAAGATTTTGTAAGTTATTACTGTCAACAACTTTACAGTACTCCTCT
IGKV12-98*01_C3H	C3H/HEJ	IGKV	MUSMUS IGKV12-98*01 F	100	GACATTCAGATGACCCAGTCTCCTGCCTCCCAGTCTGCATCTCTGGGAGAAAGTGTCACCATCACATGCCTGGCAAGTCAGACCATTGGTACATGGTTAGCATGGTATCAGCAGAAACCAGGGAAATCTCCTCAGCTCCTGATTTATGCTGCAACCAGCTTGGCAGATGGGGTCCCATCAAGGTTCAGTGGTAGTGGATCTGGCACAAAATTTTCTTTCAAGATCAGCAGCCTACAGGCTGAAGATTTTGTAAGTTATTACTGTCAACAACTTTACAGTACTCCTCT
IGKV12-98*01_DBA1	DBA/1J	IGKV	MUSMUS IGKV12-98*01 F	100	GACATTCAGATGACCCAGTCTCCTGCCTCCCAGTCTGCATCTCTGGGAGAAAGTGTCACCATCACATGCCTGGCAAGTCAGACCATTGGTACATGGTTAGCATGGTATCAGCAGAAACCAGGGAAATCTCCTCAGCTCCTGATTTATGCTGCAACCAGCTTGGCAGATGGGGTCCCATCAAGGTTCAGTGGTAGTGGATCTGGCACAAAATTTTCTTTCAAGATCAGCAGCCTACAGGCTGAAGATTTTGTAAGTTATTACTGTCAACAACTTTACAGTACTCCTCT
IGKV12-98*01_DBA2	DBA/2J	IGKV	MUSMUS IGKV12-98*01 F	100	GACATTCAGATGACCCAGTCTCCTGCCTCCCAGTCTGCATCTCTGGGAGAAAGTGTCACCATCACATGCCTGGCAAGTCAGACCATTGGTACATGGTTAGCATGGTATCAGCAGAAACCAGGGAAATCTCCTCAGCTCCTGATTTATGCTGCAACCAGCTTGGCAGATGGGGTCCCATCAAGGTTCAGTGGTAGTGGATCTGGCACAAAATTTTCTTTCAAGATCAGCAGCCTACAGGCTGAAGATTTTGTAAGTTATTACTGTCAACAACTTTACAGTACTCCTCT

IGKV12-98*01_LEWES	LEWES/EIJ	IGKV	MUSMUS IGKV12-98*01 F	100	GACATTCAGATGACCCAGTCTC<br>CTGCCTCCCAGTCTGCATCTCT<br>GGGAGAAAGTGTCACCATCACA<br>TGCCTGGCAAGTCAGACCATTG<br>GTACATGGTTAGCATGGTATCAG<br>CAGAAACCAGGGAAATCTCCTC<br>AGCTCCTGATTTATGCTGCAACC<br>AGCTTGGCAGATGGGGTCCCAT<br>CAAGGTTCAGTGGTAGTGGATC<br>TGGCACAAAATTTTCTTTCAAGAT<br>CAGCAGCCTACAGGCTGAAGAT<br>TTTGTAAGTTATTACTGTCAACAA<br>CTTTACAGTACTCCTCT
IGKV12-98*01_NZB	NZB/BLNJ	IGKV	MUSMUS IGKV12-98*01 F	100	GACATTCAGATGACCCAGTCTC<br>CTGCCTCCCAGTCTGCATCTCT<br>GGGAGAAAGTGTCACCATCACA<br>TGCCTGGCAAGTCAGACCATTG<br>GTACATGGTTAGCATGGTATCAG<br>CAGAAACCAGGGAAATCTCCTC<br>AGCTCCTGATTTATGCTGCAACC<br>AGCTTGGCAGATGGGGTCCCAT<br>CAAGGTTCAGTGGTAGTGGATC<br>TGGCACAAAATTTTCTTTCAAGAT<br>CAGCAGCCTACAGGCTGAAGAT<br>TTTGTAAGTTATTACTGTCAACAA<br>CTTTACAGTACTCCTCT
IGKV12-98*01_S2656_CAST	CAST/EIJ	IGKV	MUSMUS IGKV12-98*01 F	98.92	GACATTCAGATGACCCAGTCTC<br>CTGCCTCCCAGTCTGCATCTCT<br>GGGAGAAAGTGTCACCATCACA<br>TGCCTGGCAAGTCAGACCATTG<br>GTACATGGTTAGCATGGTATCAG<br>CAGAAACCAGGGAAATCTCCTC<br>AGCTCCTGATTTATGCTGCAACC<br>AGCTTGGCAGATGGGGTCCCAT<br>CAAGGTTCAGTGGTAGTGGATC<br>TGGCACAAAGTTTTCTTTCAAGA<br>TCAGCAGCCTACAGGCTGAAGA<br>TTTTGCAAGTTATTACTGTCAACA<br>ACATTACAGTACTCCTCT
IGKV12-98*01_S2656_MSM	MSM/MSJ	IGKV	MUSMUS IGKV12-98*01 F	98.92	GACATTCAGATGACCCAGTCTC<br>CTGCCTCCCAGTCTGCATCTCT<br>GGGAGAAAGTGTCACCATCACA<br>TGCCTGGCAAGTCAGACCATTG<br>GTACATGGTTAGCATGGTATCAG<br>CAGAAACCAGGGAAATCTCCTC<br>AGCTCCTGATTTATGCTGCAACC<br>AGCTTGGCAGATGGGGTCCCAT<br>CAAGGTTCAGTGGTAGTGGATC<br>TGGCACAAAGTTTTCTTTCAAGA<br>TCAGCAGCCTACAGGCTGAAGA<br>TTTTGCAAGTTATTACTGTCAACA<br>ACATTACAGTACTCCTCT

IGKV12-98*01_S4885_PWD	PWD/PHJ	IGKV	MUSMUS IGKV12-98*01 F	98.21	GACATTCAGATGACCCAGTCTCCTGCCTCCCAGTCTGCATCTCTGGGAGAAAGTGTCACCATCACATGCCTGGCAAGTCAGCCCATTGGTGCATGGTTAGCATGGTATCAGCAGAAACCAGGGAAATCTCCTCAGCTCCTGATTTATGCTGCAACCAGCTTGGCAGATGGGGTCCCATCAAGGTTCAGTGGTAGTGGATCTGGCACAAAGTTTTCTTTCAAGATCAGCAGCCTACAGGCTGAAGATTTTGCAAGTTATTACTGTCAACAACGTTACAGTACTCCTCT
IGKV13-84*01_129	129S1/SVIMJ	IGKV	MUSMUS IGKV13-84*01 F	100	GACATCCAGATGACACAATCTTCATCCTCCTTTTCTGTATCTCTAGGAGACAGAGTCACCATTACTTGCAAGGCAAGTGAGGACATATATAATCGGTTAGCCTGGTATCAGCAGAAACCAGGAAATGCTCCTAGGCTCTTAATATCTGGTGCAACCAGTTTGGAAACTGGGGTTCCTTCAAGATTCAGTGGCAGTGGATCTGGAAAGGATTACACTCTCAGCATTACCAGTCTTCAGACTGAAGATGTTGCTACTTATTACTGTCAACAGTATTGGAGTACTCCTCC
IGKV13-84*01_AJ	A/J	IGKV	MUSMUS IGKV13-84*01 F	100	GACATCCAGATGACACAATCTTCATCCTCCTTTTCTGTATCTCTAGGAGACAGAGTCACCATTACTTGCAAGGCAAGTGAGGACATATATAATCGGTTAGCCTGGTATCAGCAGAAACCAGGAAATGCTCCTAGGCTCTTAATATCTGGTGCAACCAGTTTGGAAACTGGGGTTCCTTCAAGATTCAGTGGCAGTGGATCTGGAAAGGATTACACTCTCAGCATTACCAGTCTTCAGACTGAAGATGTTGCTACTTATTACTGTCAACAGTATTGGAGTACTCCTCC
IGKV13-84*01_B6	C57BL/6J	IGKV	MUSMUS IGKV13-84*01 F	100	GACATCCAGATGACACAATCTTCATCCTCCTTTTCTGTATCTCTAGGAGACAGAGTCACCATTACTTGCAAGGCAAGTGAGGACATATATAATCGGTTAGCCTGGTATCAGCAGAAACCAGGAAATGCTCCTAGGCTCTTAATATCTGGTGCAACCAGTTTGGAAACTGGGGTTCCTTCAAGATTCAGTGGCAGTGGATCTGGAAAGGATTACACTCTCAGCATTACCAGTCTTCAGACTGAAGATGTTGCTACTTATTACTGTCAACAGTATTGGAGTACTCCTCC

IGKV13-84*01_BALB	BALB/CBY J	IGKV	MUSMUS IGKV13-84*01 F	100	GACATCCAGATGACACAATCTTCATCCTCCTTTTCTGTATCTCTAGGAGACAGAGTCACCATTACTTGCAAGGCAAGTGAGGACATATATAATCGGTTAGCCTGGTATCAGCAGAAACCAGGAAATGCTCCTAGGCTCTTAATATCTGGTGCAACCAGTTTGGAAACTGGGGTTCCTTCAAGATTCAGTGGCAGTGGATCTGGAAAGGATTACACTCTCAGCATTACCAGTCTTCAGACTGAAGATGTTGCTACTTATTACTGTCAACAGTATTGGAGTACTCCTCC
IGKV13-84*01_C3H	C3H/HEJ	IGKV	MUSMUS IGKV13-84*01 F	100	GACATCCAGATGACACAATCTTCATCCTCCTTTTCTGTATCTCTAGGAGACAGAGTCACCATTACTTGCAAGGCAAGTGAGGACATATATAATCGGTTAGCCTGGTATCAGCAGAAACCAGGAAATGCTCCTAGGCTCTTAATATCTGGTGCAACCAGTTTGGAAACTGGGGTTCCTTCAAGATTCAGTGGCAGTGGATCTGGAAAGGATTACACTCTCAGCATTACCAGTCTTCAGACTGAAGATGTTGCTACTTATTACTGTCAACAGTATTGGAGTACTCCTCC
IGKV13-84*01_CBA	CBA/J	IGKV	MUSMUS IGKV13-84*01 F	100	GACATCCAGATGACACAATCTTCATCCTCCTTTTCTGTATCTCTAGGAGACAGAGTCACCATTACTTGCAAGGCAAGTGAGGACATATATAATCGGTTAGCCTGGTATCAGCAGAAACCAGGAAATGCTCCTAGGCTCTTAATATCTGGTGCAACCAGTTTGGAAACTGGGGTTCCTTCAAGATTCAGTGGCAGTGGATCTGGAAAGGATTACACTCTCAGCATTACCAGTCTTCAGACTGAAGATGTTGCTACTTATTACTGTCAACAGTATTGGAGTACTCCTCC
IGKV13-84*01_DBA1	DBA/1J	IGKV	MUSMUS IGKV13-84*01 F	100	GACATCCAGATGACACAATCTTCATCCTCCTTTTCTGTATCTCTAGGAGACAGAGTCACCATTACTTGCAAGGCAAGTGAGGACATATATAATCGGTTAGCCTGGTATCAGCAGAAACCAGGAAATGCTCCTAGGCTCTTAATATCTGGTGCAACCAGTTTGGAAACTGGGGTTCCTTCAAGATTCAGTGGCAGTGGATCTGGAAAGGATTACACTCTCAGCATTACCAGTCTTCAGACTGAAGATGTTGCTACTTATTACTGTCAACAGTATTGGAGTACTCCTCC

IGKV13-84*01_DBA2	DBA/2J	IGKV	MUSMUS IGKV13-84*01 F	100	GACATCCAGATGACACAATCTT<br>CATCCTCCTTTTCTGTATCTCTA<br>GGAGACAGAGTCACCATTACTT<br>GCAAGGCAAGTGAGGACATATA<br>TAATCGGTTAGCCTGGTATCAG<br>CAGAAACCAGGAAATGCTCCTA<br>GGCTCTTAATATCTGGTGCAACC<br>AGTTTGGAAACTGGGGTTCCTTC<br>AAGATTCAGTGGCAGTGGATCT<br>GGAAAGGATTACACTCTCAGCA<br>TTACCAGTCTTCAGACTGAAGAT<br>GTTGCTACTTATTACTGTCAACA<br>GTATTGGAGTACTCCTCC
IGKV13-84*01_LEWES	LEWES/EIJ	IGKV	MUSMUS IGKV13-84*01 F	100	GACATCCAGATGACACAATCTT<br>CATCCTCCTTTTCTGTATCTCTA<br>GGAGACAGAGTCACCATTACTT<br>GCAAGGCAAGTGAGGACATATA<br>TAATCGGTTAGCCTGGTATCAG<br>CAGAAACCAGGAAATGCTCCTA<br>GGCTCTTAATATCTGGTGCAACC<br>AGTTTGGAAACTGGGGTTCCTTC<br>AAGATTCAGTGGCAGTGGATCT<br>GGAAAGGATTACACTCTCAGCA<br>TTACCAGTCTTCAGACTGAAGAT<br>GTTGCTACTTATTACTGTCAACA<br>GTATTGGAGTACTCCTCC
IGKV13-84*01_NZB	NZB/BLNJ	IGKV	MUSMUS IGKV13-84*01 F	100	GACATCCAGATGACACAATCTT<br>CATCCTCCTTTTCTGTATCTCTA<br>GGAGACAGAGTCACCATTACTT<br>GCAAGGCAAGTGAGGACATATA<br>TAATCGGTTAGCCTGGTATCAG<br>CAGAAACCAGGAAATGCTCCTA<br>GGCTCTTAATATCTGGTGCAACC<br>AGTTTGGAAACTGGGGTTCCTTC<br>AAGATTCAGTGGCAGTGGATCT<br>GGAAAGGATTACACTCTCAGCA<br>TTACCAGTCTTCAGACTGAAGAT<br>GTTGCTACTTATTACTGTCAACA<br>GTATTGGAGTACTCCTCC
IGKV13-84*01_S4500_MSM	MSM/MSJ	IGKV	MUSMUS IGKV13-84*01 F	98.92	GACATCCAGATGACACAATCTT<br>CATCCTCCTTGTCTGTATCTCTA<br>GGAGACAGAGTTACCATTACTT<br>GCAAGGCAAGTGAGGACATATA<br>TAATCGGTTAGCCTCGTATCAGC<br>AGAAACCAGGAAATGCTCCTAG<br>GCTCTTAATATCTGGTGCAACCA<br>GTTTGGAAACTGGGGTTCCTTCA<br>AGATTCAGTGGCAGTGGATCTG<br>GAAAGGATTACACTCTCAGCATT<br>ACCAGTCTTCAGACTGAAGATGT<br>TGCTACTTATTACTGTCAACAGT<br>ATTGGAGTACTCCTCC

IGKV13-84*01_S4500_PWD	PWD/PHJ	IGKV	MUSMUS IGKV13-84*01 F	98.92	GACATCCAGATGACACAATCTTCATCCTCCTTGTCTGTATCTCTAGGAGACAGAGTTACCATTACTTGCAAGGCAAGTGAGGACATATATAATCGGTTAGCCTCGTATCAGCAGAAACCAGGAAATGCTCCTAGGCTCTTAATATCTGGTGCAACCAGTTTGGAAACTGGGGTTCCTTCAAGATTCAGTGGCAGTGGATCTGGAAAGGATTACACTCTCAGCATTACCAGTCTTCAGACTGAAGATGTTGCTACTTATTACTGTCAACAGTATTGGAGTACTCCTCC
IGKV13-84*01_SJL	SJL/J	IGKV	MUSMUS IGKV13-84*01 F	100	GACATCCAGATGACACAATCTTCATCCTCCTTTTCTGTATCTCTAGGAGACAGAGTCACCATTACTTGCAAGGCAAGTGAGGACATATATAATCGGTTAGCCTGGTATCAGCAGAAACCAGGAAATGCTCCTAGGCTCTTAATATCTGGTGCAACCAGTTTGGAAACTGGGGTTCCTTCAAGATTCAGTGGCAGTGGATCTGGAAAGGATTACACTCTCAGCATTACCAGTCTTCAGACTGAAGATGTTGCTACTTATTACTGTCAACAGTATTGGAGTACTCCTCC
IGKV13-85*01_129	129S1/SVIMJ	IGKV	MUSMUS IGKV13-85*01 F	100	GACATCCAGATGACACAATCTTCATCCTACTTGTCTGTATCTCTAGGAGGCAGAGTCACCATTACTTGCAAGGCAAGTGACCACATTAATAATTGGTTAGCCTGGTATCAGCAGAAACCAGGAAATGCTCCTAGGCTCTTAATATCTGGTGCAACCAGTTTGGAAACTGGGGTTCCTTCAAGATTCAGTGGCAGTGGATCTGGAAAGGATTACACTCTCAGCATTACCAGTCTTCAGACTGAAGATGTTGCTACTTATTACTGTCAACAGTATTGGAGTACTCCTCC
IGKV13-85*01_AJ	A/J	IGKV	MUSMUS IGKV13-85*01 F	100	GACATCCAGATGACACAATCTTCATCCTACTTGTCTGTATCTCTAGGAGGCAGAGTCACCATTACTTGCAAGGCAAGTGACCACATTAATAATTGGTTAGCCTGGTATCAGCAGAAACCAGGAAATGCTCCTAGGCTCTTAATATCTGGTGCAACCAGTTTGGAAACTGGGGTTCCTTCAAGATTCAGTGGCAGTGGATCTGGAAAGGATTACACTCTCAGCATTACCAGTCTTCAGACTGAAGATGTTGCTACTTATTACTGTCAACAGTATTGGAGTACTCCTCC

IGKV13-85*01_B6	C57BL/6J	IGKV	MUSMUS IGKV13-85*01 F	100	GACATCCAGATGACACAATCTTCATCCTACTTGTCTGTATCTCTAGGAGGCAGAGTCACCATTACTTGCAAGGCAAGTGACCACATTAATAATTGGTTAGCCTGGTATCAGCAGAAACCAGGAAATGCTCCTAGGCTCTTAATATCTGGTGCAACCAGTTTGGAAACTGGGGTTCCTTCAAGATTCAGTGGCAGTGGATCTGGAAAGGATTACACTCTCAGCATTACCAGTCTTCAGACTGAAGATGTTGCTACTTATTACTGTCAACAGTATTGGAGTACTCCTCC
IGKV13-85*01_BALB	BALB/CBYJ	IGKV	MUSMUS IGKV13-85*01 F	100	GACATCCAGATGACACAATCTTCATCCTACTTGTCTGTATCTCTAGGAGGCAGAGTCACCATTACTTGCAAGGCAAGTGACCACATTAATAATTGGTTAGCCTGGTATCAGCAGAAACCAGGAAATGCTCCTAGGCTCTTAATATCTGGTGCAACCAGTTTGGAAACTGGGGTTCCTTCAAGATTCAGTGGCAGTGGATCTGGAAAGGATTACACTCTCAGCATTACCAGTCTTCAGACTGAAGATGTTGCTACTTATTACTGTCAACAGTATTGGAGTACTCCTCC
IGKV13-85*01_C3H	C3H/HEJ	IGKV	MUSMUS IGKV13-85*01 F	100	GACATCCAGATGACACAATCTTCATCCTACTTGTCTGTATCTCTAGGAGGCAGAGTCACCATTACTTGCAAGGCAAGTGACCACATTAATAATTGGTTAGCCTGGTATCAGCAGAAACCAGGAAATGCTCCTAGGCTCTTAATATCTGGTGCAACCAGTTTGGAAACTGGGGTTCCTTCAAGATTCAGTGGCAGTGGATCTGGAAAGGATTACACTCTCAGCATTACCAGTCTTCAGACTGAAGATGTTGCTACTTATTACTGTCAACAGTATTGGAGTACTCCTCC
IGKV13-85*01_CBA	CBA/J	IGKV	MUSMUS IGKV13-85*01 F	100	GACATCCAGATGACACAATCTTCATCCTACTTGTCTGTATCTCTAGGAGGCAGAGTCACCATTACTTGCAAGGCAAGTGACCACATTAATAATTGGTTAGCCTGGTATCAGCAGAAACCAGGAAATGCTCCTAGGCTCTTAATATCTGGTGCAACCAGTTTGGAAACTGGGGTTCCTTCAAGATTCAGTGGCAGTGGATCTGGAAAGGATTACACTCTCAGCATTACCAGTCTTCAGACTGAAGATGTTGCTACTTATTACTGTCAACAGTATTGGAGTACTCCTCC

IGKV13-85*01_DBA1	DBA/1J	IGKV	MUSMUS IGKV13-85*01 F	100	GACATCCAGATGACACAATCTTCATCCTACTTGTCTGTATCTCTAGGAGGCAGAGTCACCATTACTTGCAAGGCAAGTGACCACATTAATAATTGGTTAGCCTGGTATCAGCAGAAACCAGGAAATGCTCCTAGGCTCTTAATATCTGGTGCAACCAGTTTGGAAACTGGGGTTCCTTCAAGATTCAGTGGCAGTGGATCTGGAAAGGATTACACTCTCAGCATTACCAGTCTTCAGACTGAAGATGTTGCTACTTATTACTGTCAACAGTATTGGAGTACTCCTCC
IGKV13-85*01_DBA2	DBA/2J	IGKV	MUSMUS IGKV13-85*01 F	100	GACATCCAGATGACACAATCTTCATCCTACTTGTCTGTATCTCTAGGAGGCAGAGTCACCATTACTTGCAAGGCAAGTGACCACATTAATAATTGGTTAGCCTGGTATCAGCAGAAACCAGGAAATGCTCCTAGGCTCTTAATATCTGGTGCAACCAGTTTGGAAACTGGGGTTCCTTCAAGATTCAGTGGCAGTGGATCTGGAAAGGATTACACTCTCAGCATTACCAGTCTTCAGACTGAAGATGTTGCTACTTATTACTGTCAACAGTATTGGAGTACTCCTCC
IGKV13-85*01_LEWES	LEWES/EIJ	IGKV	MUSMUS IGKV13-85*01 F	100	GACATCCAGATGACACAATCTTCATCCTACTTGTCTGTATCTCTAGGAGGCAGAGTCACCATTACTTGCAAGGCAAGTGACCACATTAATAATTGGTTAGCCTGGTATCAGCAGAAACCAGGAAATGCTCCTAGGCTCTTAATATCTGGTGCAACCAGTTTGGAAACTGGGGTTCCTTCAAGATTCAGTGGCAGTGGATCTGGAAAGGATTACACTCTCAGCATTACCAGTCTTCAGACTGAAGATGTTGCTACTTATTACTGTCAACAGTATTGGAGTACTCCTCC
IGKV13-85*01_NZB	NZB/BLNJ	IGKV	MUSMUS IGKV13-85*01 F	100	GACATCCAGATGACACAATCTTCATCCTACTTGTCTGTATCTCTAGGAGGCAGAGTCACCATTACTTGCAAGGCAAGTGACCACATTAATAATTGGTTAGCCTGGTATCAGCAGAAACCAGGAAATGCTCCTAGGCTCTTAATATCTGGTGCAACCAGTTTGGAAACTGGGGTTCCTTCAAGATTCAGTGGCAGTGGATCTGGAAAGGATTACACTCTCAGCATTACCAGTCTTCAGACTGAAGATGTTGCTACTTATTACTGTCAACAGTATTGGAGTACTCCTCC

IGKV13-85*01_S1266_AKR	AKR/J	IGKV	MUSMUS IGKV13-85*01 F	97.13	GACATCCAGATGACACAATCTTCATCCTCCTTGTCTGTATCTCTAGGAGACAGAGTCACCATTACTTGCAAGGCAAGTGAGCACATTAATAGTTGGTTAGCCTGGTATCAGCAAAAACCAGGAAATGCTCCTAGGCTCTTAATATCTGGTGCAACCAGTTTGGAAACTGGGGTTCCTTCAAGATTCAGTGGCAGTGCATCTGGAAAGGATTACACTCTCAGCATTACTAGTCTTCAGACTGAAGATGTTGCTACTTATTACTGTCAACAGTATTGGGGTACTCCTCC
IGKV13-85*01_S1266_CAST	CAST/EIJ	IGKV	MUSMUS IGKV13-85*01 F	97.13	GACATCCAGATGACACAATCTTCATCCTCCTTGTCTGTATCTCTAGGAGACAGAGTCACCATTACTTGCAAGGCAAGTGAGCACATTAATAGTTGGTTAGCCTGGTATCAGCAAAAACCAGGAAATGCTCCTAGGCTCTTAATATCTGGTGCAACCAGTTTGGAAACTGGGGTTCCTTCAAGATTCAGTGGCAGTGCATCTGGAAAGGATTACACTCTCAGCATTACTAGTCTTCAGACTGAAGATGTTGCTACTTATTACTGTCAACAGTATTGGGGTACTCCTCC
IGKV13-85*01_S1899_MSM	MSM/MSJ	IGKV	MUSMUS IGKV13-85*01 F	97.49	GACATCCAGATGACACAATCTTCATCCTCCTTGTCTGTATCTCTACGAGGCAGAGTCACCATTACGTGCAAGGCAAGTGACCACATTAATAATTGGTTAGCCTGGTATCAGCAGAAACCAGGAAATGCTTCTAGGCTCTTAATATCTAGTGCGACCAGTTTGGAAAATGGGGTTCCTTCAAGATTCAGTGGCAGTGGATCTGGAAAGGATTACACTCTCAGCATTACCAGTCTTCAGACTGAAGATGTTGCTACTTATTACTGTCAACAGTATTGGAGTACTCCTCC
IGKV13-85*01_S2277_MRL	MRL/MPJ	IGKV	MUSMUS IGKV13-85*01 F	97.13	GACATCCAGATGACACAATCTTCATCCTCCTTGTCTGTATCTCTAGGAGACAGAGTCACCATTACTTGCAAGGCAAGTGAGCACATTAATAGTTGGTTAGCCTGGTATCAGCAAAAACCAGGAAATGCTCCTAGGCTCTTAATATCTGGTGCAACCAGTTTGGAAACTGGGGTTCCTTCAAGATTCAGTGGCAGTGCATCTGGAAAGGATTACACTCTCAGCATTACTAGTCTTCAGACTGAAGATGTTGCTACTTATTACTGTCAACAGTATTGGGGTACTCCTC

IGKV13-85*01_S2277_NOR	NOR/LTJ	IGKV	MUSMUS IGKV13-85*01 F	97.13	GACATCCAGATGACACAATCTTCATCCTCCTTGTCTGTATCTCTAGGAGACAGAGTCACCATTACTTGCAAGGCAAGTGAGCACATTAATAGTTGGTTAGCCTGGTATCAGCAAAAACCAGGAAATGCTCCTAGGCTCTTAATATCTGGTGCAACCAGTTTGGAAACTGGGGTTCCTTCAAGATTCAGTGGCAGTGCATCTGGAAAGGATTACACTCTCAGCATTACTAGTCTTCAGACTGAAGATGTTGCTACTTATTACTGTCAACAGTATTGGGGTACTCCTC
IGKV13-85*01_S5594_PWD	PWD/PHJ	IGKV	MUSMUS IGKV13-85*01 F	97.85	GACATCCAGATGACACAATCTTCATCCTCCTTGTCTGTATCTCTACGAGGCAGAGTCACCATTACGTGCAAGGCAAGTGACCACATTAATAATTGGTTAGCCTGGTATCAGCAGAAACCAGGAAATGCTTCTAGGCTCTTAATATCTGGTGCGACCAGTTTGGAAAATGGGGTTCCTTCAAGATTCAGTGGCAGTGGATCTGGAAAGGATTACACTCTCAGCATTACCAGTCTTCAGACTGAAGATGTTGCTACTTATTACTGTCAACAGTATTGGAGTACTCCTCC
IGKV13-85*01_SJL	SJL/J	IGKV	MUSMUS IGKV13-85*01 F	100	GACATCCAGATGACACAATCTTCATCCTACTTGTCTGTATCTCTAGGAGGCAGAGTCACCATTACTTGCAAGGCAAGTGACCACATTAATAATTGGTTAGCCTGGTATCAGCAGAAACCAGGAAATGCTCCTAGGCTCTTAATATCTGGTGCAACCAGTTTGGAAACTGGGGTTCCTTCAAGATTCAGTGGCAGTGGATCTGGAAAGGATTACACTCTCAGCATTACCAGTCTTCAGACTGAAGATGTTGCTACTTATTACTGTCAACAGTATTGGAGTACTCCTCC
IGKV14-100*01_129	129S1/SVIMJ	IGKV	MUSMUS IGKV14-100*01 F	100	GACATCCTGATGACCCAATCTCCATCCTCCATGTCTGTATCTCTGGGAGACACAGTCAGCATCACTTGCCATGCAAGTCAGGGCATTAGCAGTAATATAGGGTGGTTGCAGCAGAAACCAGGGAAATCATTTAAGGGCCTGATCTATCATGGAACCAACTTGGAAGATGGAGTTCCATCAAGGTTCAGTGGCAGTGGATCTGGAGCAGATTATTCTCTCACCATCAGCAGCCTGGAATCTGAAGATTTTGCAGACTATTACTGTGTACAGTATGCTCAGTTTCCTCC

IGKV14-100*01_AJ	A/J	IGKV	MUSMUS IGKV14-100*01 F	100	GACATCCTGATGACCCAATCTCCATCCTCCATGTCTGTATCTCTGGGAGACACAGTCAGCATCACTTGCCATGCAAGTCAGGGCATTAGCAGTAATATAGGGTGGTTGCAGCAGAAACCAGGGAAATCATTTAAGGGCCTGATCTATCATGGAACCAACTTGGAAGATGGAGTTCCATCAAGGTTCAGTGGCAGTGGATCTGGAGCAGATTATTCTCTCACCATCAGCAGCCTGGAATCTGAAGATTTTGCAGACTATTACTGTGTACAGTATGCTCAGTTTCCTCC
IGKV14-100*01_B6	C57BL/6J	IGKV	MUSMUS IGKV14-100*01 F	100	GACATCCTGATGACCCAATCTCCATCCTCCATGTCTGTATCTCTGGGAGACACAGTCAGCATCACTTGCCATGCAAGTCAGGGCATTAGCAGTAATATAGGGTGGTTGCAGCAGAAACCAGGGAAATCATTTAAGGGCCTGATCTATCATGGAACCAACTTGGAAGATGGAGTTCCATCAAGGTTCAGTGGCAGTGGATCTGGAGCAGATTATTCTCTCACCATCAGCAGCCTGGAATCTGAAGATTTTGCAGACTATTACTGTGTACAGTATGCTCAGTTTCCTCC
IGKV14-100*01_BALB	BALB/CBYJ	IGKV	MUSMUS IGKV14-100*01 F	100	GACATCCTGATGACCCAATCTCCATCCTCCATGTCTGTATCTCTGGGAGACACAGTCAGCATCACTTGCCATGCAAGTCAGGGCATTAGCAGTAATATAGGGTGGTTGCAGCAGAAACCAGGGAAATCATTTAAGGGCCTGATCTATCATGGAACCAACTTGGAAGATGGAGTTCCATCAAGGTTCAGTGGCAGTGGATCTGGAGCAGATTATTCTCTCACCATCAGCAGCCTGGAATCTGAAGATTTTGCAGACTATTACTGTGTACAGTATGCTCAGTTTCCTCC
IGKV14-100*01_C3H	C3H/HEJ	IGKV	MUSMUS IGKV14-100*01 F	100	GACATCCTGATGACCCAATCTCCATCCTCCATGTCTGTATCTCTGGGAGACACAGTCAGCATCACTTGCCATGCAAGTCAGGGCATTAGCAGTAATATAGGGTGGTTGCAGCAGAAACCAGGGAAATCATTTAAGGGCCTGATCTATCATGGAACCAACTTGGAAGATGGAGTTCCATCAAGGTTCAGTGGCAGTGGATCTGGAGCAGATTATTCTCTCACCATCAGCAGCCTGGAATCTGAAGATTTTGCAGACTATTACTGTGTACAGTATGCTCAGTTTCCTCC

IGKV14-100*01_CBA	CBA/J	IGKV	MUSMUS IGKV14-100*01 F	100	GACATCCTGATGACCCAATCTC<br>CATCCTCCATGTCTGTATCTCTG<br>GGAGACACAGTCAGCATCACTT<br>GCCATGCAAGTCAGGGCATTAG<br>CAGTAATATAGGGTGGTTGCAG<br>CAGAAACCAGGGAAATCATTTAA<br>GGGCCTGATCTATCATGGAACC<br>AACTTGGAAGATGGAGTTCCAT<br>CAAGGTTCAGTGGCAGTGGATC<br>TGGAGCAGATTATTCTCTCACCA<br>TCAGCAGCCTGGAATCTGAAGA<br>TTTTGCAGACTATTACTGTGTACA<br>GTATGCTCAGTTTCCTCC
IGKV14-100*01_DBA1	DBA/1J	IGKV	MUSMUS IGKV14-100*01 F	100	GACATCCTGATGACCCAATCTC<br>CATCCTCCATGTCTGTATCTCTG<br>GGAGACACAGTCAGCATCACTT<br>GCCATGCAAGTCAGGGCATTAG<br>CAGTAATATAGGGTGGTTGCAG<br>CAGAAACCAGGGAAATCATTTAA<br>GGGCCTGATCTATCATGGAACC<br>AACTTGGAAGATGGAGTTCCAT<br>CAAGGTTCAGTGGCAGTGGATC<br>TGGAGCAGATTATTCTCTCACCA<br>TCAGCAGCCTGGAATCTGAAGA<br>TTTTGCAGACTATTACTGTGTACA<br>GTATGCTCAGTTTCCTCC
IGKV14-100*01_DBA2	DBA/2J	IGKV	MUSMUS IGKV14-100*01 F	100	GACATCCTGATGACCCAATCTC<br>CATCCTCCATGTCTGTATCTCTG<br>GGAGACACAGTCAGCATCACTT<br>GCCATGCAAGTCAGGGCATTAG<br>CAGTAATATAGGGTGGTTGCAG<br>CAGAAACCAGGGAAATCATTTAA<br>GGGCCTGATCTATCATGGAACC<br>AACTTGGAAGATGGAGTTCCAT<br>CAAGGTTCAGTGGCAGTGGATC<br>TGGAGCAGATTATTCTCTCACCA<br>TCAGCAGCCTGGAATCTGAAGA<br>TTTTGCAGACTATTACTGTGTACA<br>GTATGCTCAGTTTCCTCC
IGKV14-100*01_LEWES	LEWES/EIJ	IGKV	MUSMUS IGKV14-100*01 F	100	GACATCCTGATGACCCAATCTC<br>CATCCTCCATGTCTGTATCTCTG<br>GGAGACACAGTCAGCATCACTT<br>GCCATGCAAGTCAGGGCATTAG<br>CAGTAATATAGGGTGGTTGCAG<br>CAGAAACCAGGGAAATCATTTAA<br>GGGCCTGATCTATCATGGAACC<br>AACTTGGAAGATGGAGTTCCAT<br>CAAGGTTCAGTGGCAGTGGATC<br>TGGAGCAGATTATTCTCTCACCA<br>TCAGCAGCCTGGAATCTGAAGA<br>TTTTGCAGACTATTACTGTGTACA<br>GTATGCTCAGTTTCCTCC

IGKV14-100*01_NZB	NZB/BLNJ	IGKV	MUSMUS IGKV14-100*01 F	100	GACATCCTGATGACCCAATCTCCATCCTCCATGTCTGTATCTCTGGGAGACACAGTCAGCATCACTTGCCATGCAAGTCAGGGCATTAGCAGTAATATAGGGTGGTTGCAGCAGAAACCAGGGAAATCATTTAAGGGCCTGATCTATCATGGAACCAACTTGGAAGATGGAGTTCCATCAAGGTTCAGTGGCAGTGGATCTGGAGCAGATTATTCTCTCACCATCAGCAGCCTGGAATCTGAAGATTTTGCAGACTATTACTGTGTACAGTATGCTCAGTTTCCTCC
IGKV14-100*01_S4794_AKR	AKR/J	IGKV	MUSMUS IGKV14-100*01 F	99.64	GACATCCTGATGACCCAATCTCCATCCTCCATGTCTGTATCTCTGGGAGACACAGTCAGCATCACTTGCCATGCAAGTCAGGGCATTAGCAGTAATATAGGGTGGTTGCAGCAGAAACCAGGGAAATCATTTAAGGGCCTGATCTATCATGCAACCAACTTGGAAGATGGAGTTCCATCAAGGTTCAGTGGCAGTGGATCTGGAGCAGATTATTCTCTCACCATCAGCAGCCTGGAATCTGAAGATTTTGCAGACTATTACTGTGTACAGTATGCTCAGTTTCCTCC
IGKV14-100*01_S4794_MRL	MRL/MPJ	IGKV	MUSMUS IGKV14-100*01 F	99.64	GACATCCTGATGACCCAATCTCCATCCTCCATGTCTGTATCTCTGGGAGACACAGTCAGCATCACTTGCCATGCAAGTCAGGGCATTAGCAGTAATATAGGGTGGTTGCAGCAGAAACCAGGGAAATCATTTAAGGGCCTGATCTATCATGCAACCAACTTGGAAGATGGAGTTCCATCAAGGTTCAGTGGCAGTGGATCTGGAGCAGATTATTCTCTCACCATCAGCAGCCTGGAATCTGAAGATTTTGCAGACTATTACTGTGTACAGTATGCTCAGTTTCCTCC
IGKV14-100*01_S4794_NOR	NOR/LTJ	IGKV	MUSMUS IGKV14-100*01 F	99.64	GACATCCTGATGACCCAATCTCCATCCTCCATGTCTGTATCTCTGGGAGACACAGTCAGCATCACTTGCCATGCAAGTCAGGGCATTAGCAGTAATATAGGGTGGTTGCAGCAGAAACCAGGGAAATCATTTAAGGGCCTGATCTATCATGCAACCAACTTGGAAGATGGAGTTCCATCAAGGTTCAGTGGCAGTGGATCTGGAGCAGATTATTCTCTCACCATCAGCAGCCTGGAATCTGAAGATTTTGCAGACTATTACTGTGTACAGTATGCTCAGTTTCCTCC

IGKV14-100*01_S6933_CAST	CAST/EIJ	IGKV	MUSMUS IGKV14-100*01 F	99.64	GACATCCTGATGACCCAATCTC<br>CATCCTCCATGTCTGTATCTCTG<br>GGAGACACAGTCAGCATCACTT<br>GCCATGCAAGTCAGGGCATTAG<br>CAGTAATATAGGGTGGTTGCAG<br>CAGAAACCAGGGAAATCATTTAA<br>GGGCCTGATCTATCATGGAACC<br>AACTTGGAAGATGGAGTTCCAT<br>CAAGGTTCAGTGGCAGTGGATC<br>TGGAGCAGATTATTCTCTCACCA<br>TCAGCAGCCTGGAATCTGAGGA<br>TTTTGCAGACTATTACTGTGTACA<br>GTATGCTCAGTTTCCTCC
IGKV14-100*01_SJL	SJL/J	IGKV	MUSMUS IGKV14-100*01 F	100	GACATCCTGATGACCCAATCTC<br>CATCCTCCATGTCTGTATCTCTG<br>GGAGACACAGTCAGCATCACTT<br>GCCATGCAAGTCAGGGCATTAG<br>CAGTAATATAGGGTGGTTGCAG<br>CAGAAACCAGGGAAATCATTTAA<br>GGGCCTGATCTATCATGGAACC<br>AACTTGGAAGATGGAGTTCCAT<br>CAAGGTTCAGTGGCAGTGGATC<br>TGGAGCAGATTATTCTCTCACCA<br>TCAGCAGCCTGGAATCTGAAGA<br>TTTTGCAGACTATTACTGTGTACA<br>GTATGCTCAGTTTCCTCC
IGKV14-111*01_129	129S1/SVIMJ	IGKV	MUSMUS IGKV14-111*01 F	100	GACATCAAGATGACCCAGTCTC<br>CATCTTCCATGTATGCATCTCTA<br>GGAGAGAGAGTCACTATCACTT<br>GCAAGGCGAGTCAGGACATTAA<br>TAGCTATTTAAGCTGGTTCCAGC<br>AGAAACCAGGGAAATCTCCTAA<br>GACCCTGATCTATCGTGCAAAC<br>AGATTGGTAGATGGGGTCCCAT<br>CAAGGTTCAGTGGCAGTGGATC<br>TGGGCAAGATTATTCTCTCACCA<br>TCAGCAGCCTGGAGTATGAAGA<br>TATGGGAATTTATTATTGTCTACA<br>GTATGATGAGTTTCCTCC
IGKV14-111*01_AJ	A/J	IGKV	MUSMUS IGKV14-111*01 F	100	GACATCAAGATGACCCAGTCTC<br>CATCTTCCATGTATGCATCTCTA<br>GGAGAGAGAGTCACTATCACTT<br>GCAAGGCGAGTCAGGACATTAA<br>TAGCTATTTAAGCTGGTTCCAGC<br>AGAAACCAGGGAAATCTCCTAA<br>GACCCTGATCTATCGTGCAAAC<br>AGATTGGTAGATGGGGTCCCAT<br>CAAGGTTCAGTGGCAGTGGATC<br>TGGGCAAGATTATTCTCTCACCA<br>TCAGCAGCCTGGAGTATGAAGA<br>TATGGGAATTTATTATTGTCTACA<br>GTATGATGAGTTTCCTCC

IGKV14-111*01_AKR	AKR/J	IGKV	MUSMUS IGKV14-111*01 F	100	GACATCAAGATGACCCAGTCTCCATCTTCCATGTATGCATCTCTAGGAGAGAGAGTCACTATCACTTGCAAGGCGAGTCAGGACATTAATAGCTATTTAAGCTGGTTCCAGCAGAAACCAGGGAAATCTCCTAAGACCCTGATCTATCGTGCAAACAGATTGGTAGATGGGGTCCCATCAAGGTTCAGTGGCAGTGGATCTGGGCAAGATTATTCTCTCACCATCAGCAGCCTGGAGTATGAAGATATGGGAATTTATTATTGTCTACAGTATGATGAGTTTCCTCC
IGKV14-111*01_B6	C57BL/6J	IGKV	MUSMUS IGKV14-111*01 F	100	GACATCAAGATGACCCAGTCTCCATCTTCCATGTATGCATCTCTAGGAGAGAGAGTCACTATCACTTGCAAGGCGAGTCAGGACATTAATAGCTATTTAAGCTGGTTCCAGCAGAAACCAGGGAAATCTCCTAAGACCCTGATCTATCGTGCAAACAGATTGGTAGATGGGGTCCCATCAAGGTTCAGTGGCAGTGGATCTGGGCAAGATTATTCTCTCACCATCAGCAGCCTGGAGTATGAAGATATGGGAATTTATTATTGTCTACAGTATGATGAGTTTCCTCC
IGKV14-111*01_BALB	BALB/CBYJ	IGKV	MUSMUS IGKV14-111*01 F	100	GACATCAAGATGACCCAGTCTCCATCTTCCATGTATGCATCTCTAGGAGAGAGAGTCACTATCACTTGCAAGGCGAGTCAGGACATTAATAGCTATTTAAGCTGGTTCCAGCAGAAACCAGGGAAATCTCCTAAGACCCTGATCTATCGTGCAAACAGATTGGTAGATGGGGTCCCATCAAGGTTCAGTGGCAGTGGATCTGGGCAAGATTATTCTCTCACCATCAGCAGCCTGGAGTATGAAGATATGGGAATTTATTATTGTCTACAGTATGATGAGTTTCCTCC
IGKV14-111*01_C3H	C3H/HEJ	IGKV	MUSMUS IGKV14-111*01 F	100	GACATCAAGATGACCCAGTCTCCATCTTCCATGTATGCATCTCTAGGAGAGAGAGTCACTATCACTTGCAAGGCGAGTCAGGACATTAATAGCTATTTAAGCTGGTTCCAGCAGAAACCAGGGAAATCTCCTAAGACCCTGATCTATCGTGCAAACAGATTGGTAGATGGGGTCCCATCAAGGTTCAGTGGCAGTGGATCTGGGCAAGATTATTCTCTCACCATCAGCAGCCTGGAGTATGAAGATATGGGAATTTATTATTGTCTACAGTATGATGAGTTTCCTCC

IGKV14-111*01_CBA	CBA/J	IGKV	MUSMUS IGKV14-111*01 F	100	GACATCAAGATGACCCAGTCTCCATCTTCCATGTATGCATCTCTAGGAGAGAGAGTCACTATCACTTGCAAGGCGAGTCAGGACATTAATAGCTATTTAAGCTGGTTCCAGCAGAAACCAGGGAAATCTCCTAAGACCCTGATCTATCGTGCAAACAGATTGGTAGATGGGGTCCCATCAAGGTTCAGTGGCAGTGGATCTGGGCAAGATTATTCTCTCACCATCAGCAGCCTGGAGTATGAAGATATGGGAATTTATTATTGTCTACAGTATGATGAGTTTCCTCC
IGKV14-111*01_DBA1	DBA/1J	IGKV	MUSMUS IGKV14-111*01 F	100	GACATCAAGATGACCCAGTCTCCATCTTCCATGTATGCATCTCTAGGAGAGAGAGTCACTATCACTTGCAAGGCGAGTCAGGACATTAATAGCTATTTAAGCTGGTTCCAGCAGAAACCAGGGAAATCTCCTAAGACCCTGATCTATCGTGCAAACAGATTGGTAGATGGGGTCCCATCAAGGTTCAGTGGCAGTGGATCTGGGCAAGATTATTCTCTCACCATCAGCAGCCTGGAGTATGAAGATATGGGAATTTATTATTGTCTACAGTATGATGAGTTTCCTCC
IGKV14-111*01_DBA2	DBA/2J	IGKV	MUSMUS IGKV14-111*01 F	100	GACATCAAGATGACCCAGTCTCCATCTTCCATGTATGCATCTCTAGGAGAGAGAGTCACTATCACTTGCAAGGCGAGTCAGGACATTAATAGCTATTTAAGCTGGTTCCAGCAGAAACCAGGGAAATCTCCTAAGACCCTGATCTATCGTGCAAACAGATTGGTAGATGGGGTCCCATCAAGGTTCAGTGGCAGTGGATCTGGGCAAGATTATTCTCTCACCATCAGCAGCCTGGAGTATGAAGATATGGGAATTTATTATTGTCTACAGTATGATGAGTTTCCTCC
IGKV14-111*01_MRL	MRL/MPJ	IGKV	MUSMUS IGKV14-111*01 F	100	GACATCAAGATGACCCAGTCTCCATCTTCCATGTATGCATCTCTAGGAGAGAGAGTCACTATCACTTGCAAGGCGAGTCAGGACATTAATAGCTATTTAAGCTGGTTCCAGCAGAAACCAGGGAAATCTCCTAAGACCCTGATCTATCGTGCAAACAGATTGGTAGATGGGGTCCCATCAAGGTTCAGTGGCAGTGGATCTGGGCAAGATTATTCTCTCACCATCAGCAGCCTGGAGTATGAAGATATGGGAATTTATTATTGTCTACAGTATGATGAGTTTCCTCC

IGKV14-111*01_S1462_LEWES	LEWES/EIJ	IGKV	MUSMUS IGKV14-111*01 F	97.85	GACATCAAGATGACCCAGTCTCCATCTTCCATGTATGCATCTCTAGGAGAGAGAGTCACTATCACTTGCAAGGCGAGTCAGGACATTAATAGCTATTTAAGCTGGTACCAGCAGAAACCAGGGAAATCTCCTAAGACCCTGATCTATTATGCAAACAGCTTGGTAGATGGGGTCCCATCAAGGTTCAGTGGCAGTGGATCTGGGCAAGATTGTTCTCTCACCATCAGCAGCCTGGAGTATGAAGATACGGGAATTTATTATTGTCTACAGTATGATGAGTTTCCTC
IGKV14-111*01_S2608_NZB	NZB/BLNJ	IGKV	MUSMUS IGKV14-111*01 F	97.85	GACATCAAGATGACCCAGTCTCCATCTTCCATGTATGCATCTCTAGGAGAGAGAGTCACTATCACTTGCAAGGCGAGTCAGGACATTAATAGCTATTTACGCTGGTACCAGCAGAAACCAGGAAAATCTCCTAAGACCCTGATCTATGGTGCAAACAGCTTGGTAGATGGGGTCCCATCAAGGTTCAGTGGCAGTGGATCTGGGCAAGATTATTCTCTCACCATCAGCAGTCTGGAGTATGAAGATATGGGAATTTATTATTGTCTACAGTATGATGAGTTTCCTCC
IGKV14-111*01_S2944_PWD	PWD/PHJ	IGKV	MUSMUS IGKV14-111*01 F	98.21	GACATCAAGATGACCCAGTCTCCATCTTCCATGTATGCATCTCTAGGAGAGAGAGTCACTATCACTTGCAAGGCGAGTCAGGACATTAATAGCTATTTAAGCTGGTACCAGCAGAAACCAGGGAAATCTCCTAAGACCCTGATCTATTATGCAAACAGCTTGGTAGATGGGGTCCCATCAAGGTTCAGTGGCAGTGGATCTGGGCAAGATTATTCTCTCACCATCAGCAGCCTGGAGTATGAAGATACGGGAATTTATTATTGTCTACAGTATGATGAGTTTCCTCC
IGKV14-111*01_S5416_CAST	CAST/EIJ	IGKV	MUSMUS IGKV14-111*01 F	95.7	GACATCAAGATGACCCAGTCTGCATCCTCCATGTATGCATCGCTGGGAGAGAGAGTCACTATCACTTGCAAGGCGAGTCAGGACATTAAAAGCTATTTAAGCTGGTACCAGCAGAAACCAGGGAAATCTCCTAAGACCCTGATCTATCGTGCAAACAGCTTGGTAGATGGGGTCCCATCAAGGTTCAGTGGCAGTGGATCTGGGCAAGATTATTCTCTCACCATCAGCAGCCTGGAGTATGAAGATCCGGCAATTTATTTTTGTCTACAGTATAATGAGTTTCCTCC

IGKV14-111*01_SJL	SJL/J	IGKV	MUSMUS IGKV14-111*01 F	100	GACATCAAGATGACCCAGTCTCCATCTTCCATGTATGCATCTCTAGGAGAGAGAGTCACTATCACTTGCAAGGCGAGTCAGGACATTAATAGCTATTTAAGCTGGTTCCAGCAGAAACCAGGGAAATCTCCTAAGACCCTGATCTATCGTGCAAACAGATTGGTAGATGGGGTCCCATCAAGGTTCAGTGGCAGTGGATCTGGGCAAGATTATTCTCTCACCATCAGCAGCCTGGAGTATGAAGATATGGGAATTTATTATTGTCTACAGTATGATGAGTTTCCTCC
IGKV14-126*01_129	129S1/SVIMJ	IGKV	MUSMUS IGKV14-126*01 F	100	GACATCAAGATGACCCAGTCTCCATCCTCCATGTATGCATCGCTGGGAGAGAGAGTCACTATCACTTGCAAGGCGAGTCAGGACATTAAAAGCTATTTAAGCTGGTACCAGCAGAAACCATGGAAATCTCCTAAGACCCTGATCTATTATGCAACAAGCTTGGCAGATGGGGTCCCATCAAGATTCAGTGGCAGTGGATCTGGGCAAGATTATTCTCTAACCATCAGCAGCCTGGAGTCTGACGATACAGCAACTTATTACTGTCTACAGCATGGTGAGAGCCCTCC
IGKV14-126*01_AJ	A/J	IGKV	MUSMUS IGKV14-126*01 F	100	GACATCAAGATGACCCAGTCTCCATCCTCCATGTATGCATCGCTGGGAGAGAGAGTCACTATCACTTGCAAGGCGAGTCAGGACATTAAAAGCTATTTAAGCTGGTACCAGCAGAAACCATGGAAATCTCCTAAGACCCTGATCTATTATGCAACAAGCTTGGCAGATGGGGTCCCATCAAGATTCAGTGGCAGTGGATCTGGGCAAGATTATTCTCTAACCATCAGCAGCCTGGAGTCTGACGATACAGCAACTTATTACTGTCTACAGCATGGTGAGAGCCCTCC
IGKV14-126*01_AKR	AKR/J	IGKV	MUSMUS IGKV14-126*01 F	100	GACATCAAGATGACCCAGTCTCCATCCTCCATGTATGCATCGCTGGGAGAGAGAGTCACTATCACTTGCAAGGCGAGTCAGGACATTAAAAGCTATTTAAGCTGGTACCAGCAGAAACCATGGAAATCTCCTAAGACCCTGATCTATTATGCAACAAGCTTGGCAGATGGGGTCCCATCAAGATTCAGTGGCAGTGGATCTGGGCAAGATTATTCTCTAACCATCAGCAGCCTGGAGTCTGACGATACAGCAACTTATTACTGTCTACAGCATGGTGAGAGCCCTCC

IGKV14-126*01_B6	C57BL/6J	IGKV	MUSMUS IGKV14-126*01 F	100	GACATCAAGATGACCCAGTCTC<br>CATCCTCCATGTATGCATCGCT<br>GGGAGAGAGAGTCACTATCACT<br>TGCAAGGCGAGTCAGGACATTA<br>AAAGCTATTTAAGCTGGTACCAG<br>CAGAAACCATGGAAATCTCCTA<br>AGACCCTGATCTATTATGCAACA<br>AGCTTGGCAGATGGGGTCCCAT<br>CAAGATTCAGTGGCAGTGGATC<br>TGGGCAAGATTATTCTCTAACCA<br>TCAGCAGCCTGGAGTCTGACGA<br>TACAGCAACTTATTACTGTCTAC<br>AGCATGGTGAGAGCCCTCC
IGKV14-126*01_BALB	BALB/CBYJ	IGKV	MUSMUS IGKV14-126*01 F	100	GACATCAAGATGACCCAGTCTC<br>CATCCTCCATGTATGCATCGCT<br>GGGAGAGAGAGTCACTATCACT<br>TGCAAGGCGAGTCAGGACATTA<br>AAAGCTATTTAAGCTGGTACCAG<br>CAGAAACCATGGAAATCTCCTA<br>AGACCCTGATCTATTATGCAACA<br>AGCTTGGCAGATGGGGTCCCAT<br>CAAGATTCAGTGGCAGTGGATC<br>TGGGCAAGATTATTCTCTAACCA<br>TCAGCAGCCTGGAGTCTGACGA<br>TACAGCAACTTATTACTGTCTAC<br>AGCATGGTGAGAGCCCTCC
IGKV14-126*01_C3H	C3H/HEJ	IGKV	MUSMUS IGKV14-126*01 F	100	GACATCAAGATGACCCAGTCTC<br>CATCCTCCATGTATGCATCGCT<br>GGGAGAGAGAGTCACTATCACT<br>TGCAAGGCGAGTCAGGACATTA<br>AAAGCTATTTAAGCTGGTACCAG<br>CAGAAACCATGGAAATCTCCTA<br>AGACCCTGATCTATTATGCAACA<br>AGCTTGGCAGATGGGGTCCCAT<br>CAAGATTCAGTGGCAGTGGATC<br>TGGGCAAGATTATTCTCTAACCA<br>TCAGCAGCCTGGAGTCTGACGA<br>TACAGCAACTTATTACTGTCTAC<br>AGCATGGTGAGAGCCCTCC
IGKV14-126*01_CAST	CAST/EIJ	IGKV	MUSMUS IGKV14-126*01 F	100	GACATCAAGATGACCCAGTCTC<br>CATCCTCCATGTATGCATCGCT<br>GGGAGAGAGAGTCACTATCACT<br>TGCAAGGCGAGTCAGGACATTA<br>AAAGCTATTTAAGCTGGTACCAG<br>CAGAAACCATGGAAATCTCCTA<br>AGACCCTGATCTATTATGCAACA<br>AGCTTGGCAGATGGGGTCCCAT<br>CAAGATTCAGTGGCAGTGGATC<br>TGGGCAAGATTATTCTCTAACCA<br>TCAGCAGCCTGGAGTCTGACGA<br>TACAGCAACTTATTACTGTCTAC<br>AGCATGGTGAGAGCCCTCC

IGKV14-126*01_CBA	CBA/J	IGKV	MUSMUS IGKV14-126*01 F	100	GACATCAAGATGACCCAGTCTC<br>CATCCTCCATGTATGCATCGCT<br>GGGAGAGAGAGTCACTATCACT<br>TGCAAGGCGAGTCAGGACATTA<br>AAAGCTATTTAAGCTGGTACCAG<br>CAGAAACCATGGAAATCTCCTA<br>AGACCCTGATCTATTATGCAACA<br>AGCTTGGCAGATGGGGTCCCAT<br>CAAGATTCAGTGGCAGTGGATC<br>TGGGCAAGATTATTCTCTAACCA<br>TCAGCAGCCTGGAGTCTGACGA<br>TACAGCAACTTATTACTGTCTAC<br>AGCATGGTGAGAGCCCTCC
IGKV14-126*01_DBA1	DBA/1J	IGKV	MUSMUS IGKV14-126*01 F	100	GACATCAAGATGACCCAGTCTC<br>CATCCTCCATGTATGCATCGCT<br>GGGAGAGAGAGTCACTATCACT<br>TGCAAGGCGAGTCAGGACATTA<br>AAAGCTATTTAAGCTGGTACCAG<br>CAGAAACCATGGAAATCTCCTA<br>AGACCCTGATCTATTATGCAACA<br>AGCTTGGCAGATGGGGTCCCAT<br>CAAGATTCAGTGGCAGTGGATC<br>TGGGCAAGATTATTCTCTAACCA<br>TCAGCAGCCTGGAGTCTGACGA<br>TACAGCAACTTATTACTGTCTAC<br>AGCATGGTGAGAGCCCTCC
IGKV14-126*01_DBA2	DBA/2J	IGKV	MUSMUS IGKV14-126*01 F	100	GACATCAAGATGACCCAGTCTC<br>CATCCTCCATGTATGCATCGCT<br>GGGAGAGAGAGTCACTATCACT<br>TGCAAGGCGAGTCAGGACATTA<br>AAAGCTATTTAAGCTGGTACCAG<br>CAGAAACCATGGAAATCTCCTA<br>AGACCCTGATCTATTATGCAACA<br>AGCTTGGCAGATGGGGTCCCAT<br>CAAGATTCAGTGGCAGTGGATC<br>TGGGCAAGATTATTCTCTAACCA<br>TCAGCAGCCTGGAGTCTGACGA<br>TACAGCAACTTATTACTGTCTAC<br>AGCATGGTGAGAGCCCTCC
IGKV14-126*01_MRL	MRL/MPJ	IGKV	MUSMUS IGKV14-126*01 F	100	GACATCAAGATGACCCAGTCTC<br>CATCCTCCATGTATGCATCGCT<br>GGGAGAGAGAGTCACTATCACT<br>TGCAAGGCGAGTCAGGACATTA<br>AAAGCTATTTAAGCTGGTACCAG<br>CAGAAACCATGGAAATCTCCTA<br>AGACCCTGATCTATTATGCAACA<br>AGCTTGGCAGATGGGGTCCCAT<br>CAAGATTCAGTGGCAGTGGATC<br>TGGGCAAGATTATTCTCTAACCA<br>TCAGCAGCCTGGAGTCTGACGA<br>TACAGCAACTTATTACTGTCTAC<br>AGCATGGTGAGAGCCCTCC

IGKV14-126*01_NOD	NOD/SHILTJ	IGKV	MUSMUS IGKV14-126*01 F	100	GACATCAAGATGACCCAGTCTCCATCCTCCATGTATGCATCGCTGGGAGAGAGAGTCACTATCACTTGCAAGGCGAGTCAGGACATTAAAAGCTATTTAAGCTGGTACCAGCAGAAACCATGGAAATCTCCTAAGACCCTGATCTATTATGCAACAAGCTTGGCAGATGGGGTCCCATCAAGATTCAGTGGCAGTGGATCTGGGCAAGATTATTCTCTAACCATCAGCAGCCTGGAGTCTGACGATACAGCAACTTATTACTGTCTACAGCATGGTGAGAGCCCTCC
IGKV14-126*01_NOR	NOR/LTJ	IGKV	MUSMUS IGKV14-126*01 F	100	GACATCAAGATGACCCAGTCTCCATCCTCCATGTATGCATCGCTGGGAGAGAGAGTCACTATCACTTGCAAGGCGAGTCAGGACATTAAAAGCTATTTAAGCTGGTACCAGCAGAAACCATGGAAATCTCCTAAGACCCTGATCTATTATGCAACAAGCTTGGCAGATGGGGTCCCATCAAGATTCAGTGGCAGTGGATCTGGGCAAGATTATTCTCTAACCATCAGCAGCCTGGAGTCTGACGATACAGCAACTTATTACTGTCTACAGCATGGTGAGAGCCCTCC
IGKV14-126*01_NZB	NZB/BLNJ	IGKV	MUSMUS IGKV14-126*01 F	100	GACATCAAGATGACCCAGTCTCCATCCTCCATGTATGCATCGCTGGGAGAGAGAGTCACTATCACTTGCAAGGCGAGTCAGGACATTAAAAGCTATTTAAGCTGGTACCAGCAGAAACCATGGAAATCTCCTAAGACCCTGATCTATTATGCAACAAGCTTGGCAGATGGGGTCCCATCAAGATTCAGTGGCAGTGGATCTGGGCAAGATTATTCTCTAACCATCAGCAGCCTGGAGTCTGACGATACAGCAACTTATTACTGTCTACAGCATGGTGAGAGCCCTCC
IGKV14-126*01_PWD	PWD/PHJ	IGKV	MUSMUS IGKV14-126*01 F	100	GACATCAAGATGACCCAGTCTCCATCCTCCATGTATGCATCGCTGGGAGAGAGAGTCACTATCACTTGCAAGGCGAGTCAGGACATTAAAAGCTATTTAAGCTGGTACCAGCAGAAACCATGGAAATCTCCTAAGACCCTGATCTATTATGCAACAAGCTTGGCAGATGGGGTCCCATCAAGATTCAGTGGCAGTGGATCTGGGCAAGATTATTCTCTAACCATCAGCAGCCTGGAGTCTGACGATACAGCAACTTATTACTGTCTACAGCATGGTGAGAGCCCTCC

IGKV14-126*01_S4454_LEWES	LEWES/EIJ	IGKV	MUSMUS IGKV14-126*01 F	99.28	GACATCAAGATGACCCAGTCTC<br>CATCCTCCATGTATGCATCGCT<br>GGGAGAGAGAGTCACTATCACT<br>TGCAAGGCGAGTCAGGACATTA<br>AAAGCTATTTAAGCTGGTACCAG<br>CAGAAACCAGGGAAATCTCCTA<br>AGACCCTGATCTATTATGCAACA<br>AGCTTGGCAGATGGGGTCCCAT<br>CAAGATTCAGCGGCAGTGGATC<br>TGGGCAAGATTATTCTCTAACCA<br>TCAGCAGCCTGGAGTCTGACGA<br>TACAGCAACTTATTACTGTCTAC<br>AGCATGGTGAGAGCCCTCC
IGKV14-126*01_S6908_MSM	MSM/MSJ	IGKV	MUSMUS IGKV14-126*01 F	99.64	GACATCAAGATGACCCAGTCTC<br>CATCCTCCATGTATGCATCGCT<br>GGGAGAGAGAGTCACTATCACT<br>TGCAAGGCGAGTCAGGACATTA<br>AAAGCTATTTAAGCTGGTACCAG<br>CAGAAACCAGGGAAATCTCCTA<br>AGACCCTGATCTATTATGCAACA<br>AGCTTGGCAGATGGGGTCCCAT<br>CAAGATTCAGTGGCAGTGGATC<br>TGGGCAAGATTATTCTCTAACCA<br>TCAGCAGCCTGGAGTCTGACGA<br>TACAGCAACTTATTACTGTCTAC<br>AGCATGGTGAGAGCCCTC
IGKV14-126*01_SJL	SJL/J	IGKV	MUSMUS IGKV14-126*01 F	100	GACATCAAGATGACCCAGTCTC<br>CATCCTCCATGTATGCATCGCT<br>GGGAGAGAGAGTCACTATCACT<br>TGCAAGGCGAGTCAGGACATTA<br>AAAGCTATTTAAGCTGGTACCAG<br>CAGAAACCATGGAAATCTCCTA<br>AGACCCTGATCTATTATGCAACA<br>AGCTTGGCAGATGGGGTCCCAT<br>CAAGATTCAGTGGCAGTGGATC<br>TGGGCAAGATTATTCTCTAACCA<br>TCAGCAGCCTGGAGTCTGACGA<br>TACAGCAACTTATTACTGTCTAC<br>AGCATGGTGAGAGCCCTCC
IGKV14-130*01_129	129S1/SVIMJ	IGKV	MUSMUS IGKV14-130*01 F	100	GAAATCCAGATGACCCAGTCTC<br>CATCCTCTATGTCTGCATCTCTG<br>GGAGACAGAATAACCATCACTT<br>GCCAGGCAACTCAAGACATTGT<br>TAAGAATTTAAACTGGTATCAGC<br>AGAAACCAGGGAAACCCCCTT<br>CATTCCTGATCTATTATGCAACT<br>GAACTGGCAGAAGGGGTCCCA<br>TCAAGGTTCAGTGGCAGTGGGT<br>CTGGGTCAGACTATTCTCTGACA<br>ATCAGCAACCTGGAGTCTGAAG<br>ATTTTGCAGACTATTACTGTCTAC<br>AGTTTTATGAGTTTCCTCC

IGKV14-130*01_AJ	A/J	IGKV	MUSMUS IGKV14-130*01 F	100	GAAATCCAGATGACCCAGTCTCCATCCTCTATGTCTGCATCTCTGGGAGACAGAATAACCATCACTTGCCAGGCAACTCAAGACATTGTTAAGAATTTAAACTGGTATCAGCAGAAACCAGGGAAACCCCCTTCATTCCTGATCTATTATGCAACTGAACTGGCAGAAGGGGTCCCATCAAGGTTCAGTGGCAGTGGGTCTGGGTCAGACTATTCTCTGACAATCAGCAACCTGGAGTCTGAAGATTTTGCAGACTATTACTGTCTACAGTTTTATGAGTTTCCTCC
IGKV14-130*01_AKR	AKR/J	IGKV	MUSMUS IGKV14-130*01 F	100	GAAATCCAGATGACCCAGTCTCCATCCTCTATGTCTGCATCTCTGGGAGACAGAATAACCATCACTTGCCAGGCAACTCAAGACATTGTTAAGAATTTAAACTGGTATCAGCAGAAACCAGGGAAACCCCCTTCATTCCTGATCTATTATGCAACTGAACTGGCAGAAGGGGTCCCATCAAGGTTCAGTGGCAGTGGGTCTGGGTCAGACTATTCTCTGACAATCAGCAACCTGGAGTCTGAAGATTTTGCAGACTATTACTGTCTACAGTTTTATGAGTTTCCTCC
IGKV14-130*01_B6	C57BL/6J	IGKV	MUSMUS IGKV14-130*01 F	100	GAAATCCAGATGACCCAGTCTCCATCCTCTATGTCTGCATCTCTGGGAGACAGAATAACCATCACTTGCCAGGCAACTCAAGACATTGTTAAGAATTTAAACTGGTATCAGCAGAAACCAGGGAAACCCCCTTCATTCCTGATCTATTATGCAACTGAACTGGCAGAAGGGGTCCCATCAAGGTTCAGTGGCAGTGGGTCTGGGTCAGACTATTCTCTGACAATCAGCAACCTGGAGTCTGAAGATTTTGCAGACTATTACTGTCTACAGTTTTATGAGTTTCCTCC
IGKV14-130*01_BALB	BALB/CBYJ	IGKV	MUSMUS IGKV14-130*01 F	100	GAAATCCAGATGACCCAGTCTCCATCCTCTATGTCTGCATCTCTGGGAGACAGAATAACCATCACTTGCCAGGCAACTCAAGACATTGTTAAGAATTTAAACTGGTATCAGCAGAAACCAGGGAAACCCCCTTCATTCCTGATCTATTATGCAACTGAACTGGCAGAAGGGGTCCCATCAAGGTTCAGTGGCAGTGGGTCTGGGTCAGACTATTCTCTGACAATCAGCAACCTGGAGTCTGAAGATTTTGCAGACTATTACTGTCTACAGTTTTATGAGTTTCCTCC

IGKV14-130*01_C3H	C3H/HEJ	IGKV	MUSMUS IGKV14-130*01 F	100	GAAATCCAGATGACCCAGTCTCCATCCTCTATGTCTGCATCTCTGGGAGACAGAATAACCATCACTTGCCAGGCAACTCAAGACATTGTTAAGAATTTAAACTGGTATCAGCAGAAACCAGGGAAACCCCCTTCATTCCTGATCTATTATGCAACTGAACTGGCAGAAGGGGTCCCATCAAGGTTCAGTGGCAGTGGGTCTGGGTCAGACTATTCTCTGACAATCAGCAACCTGGAGTCTGAAGATTTTGCAGACTATTACTGTCTACAGTTTTATGAGTTTCCTCC
IGKV14-130*01_CAST	CAST/EIJ	IGKV	MUSMUS IGKV14-130*01 F	100	GAAATCCAGATGACCCAGTCTCCATCCTCTATGTCTGCATCTCTGGGAGACAGAATAACCATCACTTGCCAGGCAACTCAAGACATTGTTAAGAATTTAAACTGGTATCAGCAGAAACCAGGGAAACCCCCTTCATTCCTGATCTATTATGCAACTGAACTGGCAGAAGGGGTCCCATCAAGGTTCAGTGGCAGTGGGTCTGGGTCAGACTATTCTCTGACAATCAGCAACCTGGAGTCTGAAGATTTTGCAGACTATTACTGTCTACAGTTTTATGAGTTTCCTCC
IGKV14-130*01_CBA	CBA/J	IGKV	MUSMUS IGKV14-130*01 F	100	GAAATCCAGATGACCCAGTCTCCATCCTCTATGTCTGCATCTCTGGGAGACAGAATAACCATCACTTGCCAGGCAACTCAAGACATTGTTAAGAATTTAAACTGGTATCAGCAGAAACCAGGGAAACCCCCTTCATTCCTGATCTATTATGCAACTGAACTGGCAGAAGGGGTCCCATCAAGGTTCAGTGGCAGTGGGTCTGGGTCAGACTATTCTCTGACAATCAGCAACCTGGAGTCTGAAGATTTTGCAGACTATTACTGTCTACAGTTTTATGAGTTTCCTCC
IGKV14-130*01_DBA1	DBA/1J	IGKV	MUSMUS IGKV14-130*01 F	100	GAAATCCAGATGACCCAGTCTCCATCCTCTATGTCTGCATCTCTGGGAGACAGAATAACCATCACTTGCCAGGCAACTCAAGACATTGTTAAGAATTTAAACTGGTATCAGCAGAAACCAGGGAAACCCCCTTCATTCCTGATCTATTATGCAACTGAACTGGCAGAAGGGGTCCCATCAAGGTTCAGTGGCAGTGGGTCTGGGTCAGACTATTCTCTGACAATCAGCAACCTGGAGTCTGAAGATTTTGCAGACTATTACTGTCTACAGTTTTATGAGTTTCCTCC

IGKV14-130*01_DBA2	DBA/2J	IGKV	MUSMUS IGKV14-130*01 F	100	GAAATCCAGATGACCCAGTCTCCATCCTCTATGTCTGCATCTCTGGGAGACAGAATAACCATCACTTGCCAGGCAACTCAAGACATTGTTAAGAATTTAAACTGGTATCAGCAGAAACCAGGGAAACCCCCTTCATTCCTGATCTATTATGCAACTGAACTGGCAGAAGGGGTCCCATCAAGGTTCAGTGGCAGTGGGTCTGGGTCAGACTATTCTCTGACAATCAGCAACCTGGAGTCTGAAGATTTTGCAGACTATTACTGTCTACAGTTTTATGAGTTTCCTCC
IGKV14-130*01_LEWES	LEWES/EIJ	IGKV	MUSMUS IGKV14-130*01 F	100	GAAATCCAGATGACCCAGTCTCCATCCTCTATGTCTGCATCTCTGGGAGACAGAATAACCATCACTTGCCAGGCAACTCAAGACATTGTTAAGAATTTAAACTGGTATCAGCAGAAACCAGGGAAACCCCCTTCATTCCTGATCTATTATGCAACTGAACTGGCAGAAGGGGTCCCATCAAGGTTCAGTGGCAGTGGGTCTGGGTCAGACTATTCTCTGACAATCAGCAACCTGGAGTCTGAAGATTTTGCAGACTATTACTGTCTACAGTTTTATGAGTTTCCTCC
IGKV14-130*01_NOR	NOR/LTJ	IGKV	MUSMUS IGKV14-130*01 F	100	GAAATCCAGATGACCCAGTCTCCATCCTCTATGTCTGCATCTCTGGGAGACAGAATAACCATCACTTGCCAGGCAACTCAAGACATTGTTAAGAATTTAAACTGGTATCAGCAGAAACCAGGGAAACCCCCTTCATTCCTGATCTATTATGCAACTGAACTGGCAGAAGGGGTCCCATCAAGGTTCAGTGGCAGTGGGTCTGGGTCAGACTATTCTCTGACAATCAGCAACCTGGAGTCTGAAGATTTTGCAGACTATTACTGTCTACAGTTTTATGAGTTTCCTCC
IGKV14-130*01_NZB	NZB/BLNJ	IGKV	MUSMUS IGKV14-130*01 F	100	GAAATCCAGATGACCCAGTCTCCATCCTCTATGTCTGCATCTCTGGGAGACAGAATAACCATCACTTGCCAGGCAACTCAAGACATTGTTAAGAATTTAAACTGGTATCAGCAGAAACCAGGGAAACCCCCTTCATTCCTGATCTATTATGCAACTGAACTGGCAGAAGGGGTCCCATCAAGGTTCAGTGGCAGTGGGTCTGGGTCAGACTATTCTCTGACAATCAGCAACCTGGAGTCTGAAGATTTTGCAGACTATTACTGTCTACAGTTTTATGAGTTTCCTCC

IGKV15-103*01_129	129S1/SVIMJ	IGKV	MUSMUS IGKV15-103*01 ORF	100	GACATCCAGATGAACCAGTCTCCATCCAGTCTGTCTGCATCCCTTGGAGACACAATTACCATCACTTGCCATGCCAGTCAGAACATTAATGTTTGGTTAAGCTGGTACCAGCAGAAACCAGGAAATATTCCTAAACTATTGATCTATAAGGCTTCCAACTTGCACACAGGCGTCCCATCAAGGTTTAGTGGCAGTGGATCTGGAACAGGTTTCACATTAACCATCAGCAGCCTGCAGCCTGAAGACATTGCCACTTACTACTGTCAACAGGGTCAAAGTTATCCTCT
IGKV15-103*01_AJ	A/J	IGKV	MUSMUS IGKV15-103*01 ORF	100	GACATCCAGATGAACCAGTCTCCATCCAGTCTGTCTGCATCCCTTGGAGACACAATTACCATCACTTGCCATGCCAGTCAGAACATTAATGTTTGGTTAAGCTGGTACCAGCAGAAACCAGGAAATATTCCTAAACTATTGATCTATAAGGCTTCCAACTTGCACACAGGCGTCCCATCAAGGTTTAGTGGCAGTGGATCTGGAACAGGTTTCACATTAACCATCAGCAGCCTGCAGCCTGAAGACATTGCCACTTACTACTGTCAACAGGGTCAAAGTTATCCTCT
IGKV15-103*01_AKR	AKR/J	IGKV	MUSMUS IGKV15-103*01 ORF	100	GACATCCAGATGAACCAGTCTCCATCCAGTCTGTCTGCATCCCTTGGAGACACAATTACCATCACTTGCCATGCCAGTCAGAACATTAATGTTTGGTTAAGCTGGTACCAGCAGAAACCAGGAAATATTCCTAAACTATTGATCTATAAGGCTTCCAACTTGCACACAGGCGTCCCATCAAGGTTTAGTGGCAGTGGATCTGGAACAGGTTTCACATTAACCATCAGCAGCCTGCAGCCTGAAGACATTGCCACTTACTACTGTCAACAGGGTCAAAGTTATCCTCT
IGKV15-103*01_B6	C57BL/6J	IGKV	MUSMUS IGKV15-103*01 ORF	100	GACATCCAGATGAACCAGTCTCCATCCAGTCTGTCTGCATCCCTTGGAGACACAATTACCATCACTTGCCATGCCAGTCAGAACATTAATGTTTGGTTAAGCTGGTACCAGCAGAAACCAGGAAATATTCCTAAACTATTGATCTATAAGGCTTCCAACTTGCACACAGGCGTCCCATCAAGGTTTAGTGGCAGTGGATCTGGAACAGGTTTCACATTAACCATCAGCAGCCTGCAGCCTGAAGACATTGCCACTTACTACTGTCAACAGGGTCAAAGTTATCCTCT

IGKV15-103*01_BALB	BALB/CBYJ	IGKV	MUSMUS IGKV15-103*01 ORF	100	GACATCCAGATGAACCAGTCTCCATCCAGTCTGTCTGCATCCCTTGGAGACACAATTACCATCACTTGCCATGCCAGTCAGAACATTAATGTTTGGTTAAGCTGGTACCAGCAGAAACCAGGAAATATTCCTAAACTATTGATCTATAAGGCTTCCAACTTGCACACAGGCGTCCCATCAAGGTTTAGTGGCAGTGGATCTGGAACAGGTTTCACATTAACCATCAGCAGCCTGCAGCCTGAAGACATTGCCACTTACTACTGTCAACAGGGTCAAAGTTATCCTCT
IGKV15-103*01_C3H	C3H/HEJ	IGKV	MUSMUS IGKV15-103*01 ORF	100	GACATCCAGATGAACCAGTCTCCATCCAGTCTGTCTGCATCCCTTGGAGACACAATTACCATCACTTGCCATGCCAGTCAGAACATTAATGTTTGGTTAAGCTGGTACCAGCAGAAACCAGGAAATATTCCTAAACTATTGATCTATAAGGCTTCCAACTTGCACACAGGCGTCCCATCAAGGTTTAGTGGCAGTGGATCTGGAACAGGTTTCACATTAACCATCAGCAGCCTGCAGCCTGAAGACATTGCCACTTACTACTGTCAACAGGGTCAAAGTTATCCTCT
IGKV15-103*01_CBA	CBA/J	IGKV	MUSMUS IGKV15-103*01 ORF	100	GACATCCAGATGAACCAGTCTCCATCCAGTCTGTCTGCATCCCTTGGAGACACAATTACCATCACTTGCCATGCCAGTCAGAACATTAATGTTTGGTTAAGCTGGTACCAGCAGAAACCAGGAAATATTCCTAAACTATTGATCTATAAGGCTTCCAACTTGCACACAGGCGTCCCATCAAGGTTTAGTGGCAGTGGATCTGGAACAGGTTTCACATTAACCATCAGCAGCCTGCAGCCTGAAGACATTGCCACTTACTACTGTCAACAGGGTCAAAGTTATCCTCT
IGKV15-103*01_DBA1	DBA/1J	IGKV	MUSMUS IGKV15-103*01 ORF	100	GACATCCAGATGAACCAGTCTCCATCCAGTCTGTCTGCATCCCTTGGAGACACAATTACCATCACTTGCCATGCCAGTCAGAACATTAATGTTTGGTTAAGCTGGTACCAGCAGAAACCAGGAAATATTCCTAAACTATTGATCTATAAGGCTTCCAACTTGCACACAGGCGTCCCATCAAGGTTTAGTGGCAGTGGATCTGGAACAGGTTTCACATTAACCATCAGCAGCCTGCAGCCTGAAGACATTGCCACTTACTACTGTCAACAGGGTCAAAGTTATCCTCT

IGKV15-103*01_DBA2	DBA/2J	IGKV	MUSMUS IGKV15-103*01 ORF	100	GACATCCAGATGAACCAGTCTCCATCCAGTCTGTCTGCATCCCTTGGAGACACAATTACCATCACTTGCCATGCCAGTCAGAACATTAATGTTTGGTTAAGCTGGTACCAGCAGAAACCAGGAAATATTCCTAAACTATTGATCTATAAGGCTTCCAACTTGCACACAGGCGTCCCATCAAGGTTTAGTGGCAGTGGATCTGGAACAGGTTTCACATTAACCATCAGCAGCCTGCAGCCTGAAGACATTGCCACTTACTACTGTCAACAGGGTCAAAGTTATCCTCT
IGKV15-103*01_MRL	MRL/MPJ	IGKV	MUSMUS IGKV15-103*01 ORF	100	GACATCCAGATGAACCAGTCTCCATCCAGTCTGTCTGCATCCCTTGGAGACACAATTACCATCACTTGCCATGCCAGTCAGAACATTAATGTTTGGTTAAGCTGGTACCAGCAGAAACCAGGAAATATTCCTAAACTATTGATCTATAAGGCTTCCAACTTGCACACAGGCGTCCCATCAAGGTTTAGTGGCAGTGGATCTGGAACAGGTTTCACATTAACCATCAGCAGCCTGCAGCCTGAAGACATTGCCACTTACTACTGTCAACAGGGTCAAAGTTATCCTCT
IGKV15-103*01_S0817_NOD	NOD/SHILTJ	IGKV	MUSMUS IGKV15-103*01 ORF	97.85	GACATCCAGATGAACCAGTCTCCATCCAGTCTGTCTGCATCCCTCGGAGACACAATTACCATCACTTGCCGTGCCAGTCAGAACATTAATATTTGGTTAAGCTGGTACCAGCAGAAACCAGGAAATATTCCTAAACTATTGATCTATAAGGCTTCCAACTTGCACACAGGCGTCCCATCAAGGTTTAGTGGCAGTGGATCTGGAACAGATTTCACATTAACCATCAGCAGTCTGCAGCCTGAAGACATTGCCACTTACTACTGTCTACAGGGTCAAAGTTATCCTCT
IGKV15-103*01_S0817_NOR	NOR/LTJ	IGKV	MUSMUS IGKV15-103*01 ORF	97.85	GACATCCAGATGAACCAGTCTCCATCCAGTCTGTCTGCATCCCTCGGAGACACAATTACCATCACTTGCCGTGCCAGTCAGAACATTAATATTTGGTTAAGCTGGTACCAGCAGAAACCAGGAAATATTCCTAAACTATTGATCTATAAGGCTTCCAACTTGCACACAGGCGTCCCATCAAGGTTTAGTGGCAGTGGATCTGGAACAGATTTCACATTAACCATCAGCAGTCTGCAGCCTGAAGACATTGCCACTTACTACTGTCTACAGGGTCAAAGTTATCCTCT

IGKV15-103*01_S0817_NZB	NZB/BLNJ	IGKV	MUSMUS IGKV15-103*01 ORF	97.85	GACATCCAGATGAACCAGTCTCCATCCAGTCTGTCTGCATCCCTCGGAGACACAATTACCATCACTTGCCGTGCCAGTCAGAACATTAATATTTGGTTAAGCTGGTACCAGCAGAAACCAGGAAATATTCCTAAACTATTGATCTATAAGGCTTCCAACTTGCACACAGGCGTCCCATCAAGGTTTAGTGGCAGTGGATCTGGAACAGATTTCACATTAACCATCAGCAGTCTGCAGCCTGAAGACATTGCCACTTACTACTGTCTACAGGGTCAAAGTTATCCTCT
IGKV15-103*01_S2403_CAST	CAST/EIJ	IGKV	MUSMUS IGKV15-103*01 ORF	98.21	GACATCCAGATGAACCAGTCTCCATCCAGTCTGTCTGCATCCCTCGGAGACACAATTACCATCACTTGCCGTGCCAGTCAGAACATTAATATTTGGTTAAGCTGGTACCAGCAGAAACCAGGAAATATTCCTAAACTATTGATCTATAAGGCTTCCAACTTGCACACAGGCGTCCCATCAAGGTTTAGTGGCAGTGGATCTGGAACAGATTTCACATTAACCATCAGCAGCCTGCAGCCTGAAGACATTGCCACTTACTACTGTCTACAGGGTCAAAGTTATCCTCT
IGKV15-103*01_S4601_LEWES	LEWES/EIJ	IGKV	MUSMUS IGKV15-103*01 ORF	98.21	GACATCCAGATGAACCAGTCTCCATCCAGTCTGTCTGCATCCCTCGGAGACACAATTACCATCACTTGCCGTGCCAGTCAGAACATTAATATTTGGTTAAGCTGGTACCAGCAGAAACCAGGAAATATTCCTAAACTATTGATCTATAAGGCTTCCAACTTGCACACAGGCGTCCCATCAAGGTTTAGTGGCAGTGGATCTGGAACAGATTTCACATTAACCATCAGCAGCCTGCAGCCTGAAGACATTGCCACTTACTACTGTCTACAGGGTCAAAGTCATCCTCT
IGKV15-103*01_S4991_MSM	MSM/MSJ	IGKV	MUSMUS IGKV15-103*01 ORF	95.34	GACATCCAAGTGAACCAGTCTCCATCCAGTCTGTCTGCATCCCTCGGAGACACAATTACCATCACTTGCCGTGCCAGTCAGGACATTAGTATTTGGTTAAGCTGGTACCAGCAGAAACCAGGAAATATTCCTAAACTATTGATCTATAAGGCTTCCAACTTGCACACAGGCGTCCCACCAAGGTTTAGTGGCAGTGGATCTGGGACAGATTTCACATTAACCATCAGCAGCCTACAGCCTGAAGACATTGCCACTTACTACTGTCTACAGAGTCAAAGTTATCCTCT

IGKV15-103*01_S9355_PWD	PWD/PHJ	IGKV	MUSMUS IGKV15-103*01 ORF	94.62	GACATCCAAATGAACCAGTCTCCATCCAGTCTGTCTGCATCCCTCGGAGACACAATTACCATCACTTGCCGTGCCAGTCAGGGCATTAGTATTTGGTTAAGCTGGTACCAGCAGAAACCAGGAAATATTCCTAAACTATTGATCTATAAGGCTTCCAACTTGCACACAGGAGTCCCACCAAGGTTTAGTGGCAGTGGATCTGGTACAGATTTCACATTAACTATCAGCAGCCTACAGCCTGAAGACATTGCCACTTACTACTGTCTACAGAGTCAAAGTTATCCTCT
IGKV15-103*01_SJL	SJL/J	IGKV	MUSMUS IGKV15-103*01 ORF	100	GACATCCAGATGAACCAGTCTCCATCCAGTCTGTCTGCATCCCTTGGAGACACAATTACCATCACTTGCCATGCCAGTCAGAACATTAATGTTTGGTTAAGCTGGTACCAGCAGAAACCAGGAAATATTCCTAAACTATTGATCTATAAGGCTTCCAACTTGCACACAGGCGTCCCATCAAGGTTTAGTGGCAGTGGATCTGGAACAGGTTTCACATTAACCATCAGCAGCCTGCAGCCTGAAGACATTGCCACTTACTACTGTCAACAGGGTCAAAGTTATCCTCT
IGKV16-104*01_129	129S1/SVIMJ	IGKV	MUSMUS IGKV16-104*01 F	100	GATGTCCAGATAACCCAGTCTCCATCTTATCTTGCTGCATCTCCTGGAGAAACCATTACTATTAATTGCAGGGCAAGTAAGAGCATTAGCAAATATTTAGCCTGGTATCAAGAGAAACCTGGGAAAACTAATAAGCTTCTTATCTACTCTGGATCCACTTTGCAATCTGGAATTCCATCAAGGTTCAGTGGCAGTGGATCTGGTACAGATTTCACTCTCACCATCAGTAGCCTGGAGCCTGAAGATTTTGCAATGTATTACTGTCAACAGCATAATGAATACCCGTAC
IGKV16-104*01_AJ	A/J	IGKV	MUSMUS IGKV16-104*01 F	100	GATGTCCAGATAACCCAGTCTCCATCTTATCTTGCTGCATCTCCTGGAGAAACCATTACTATTAATTGCAGGGCAAGTAAGAGCATTAGCAAATATTTAGCCTGGTATCAAGAGAAACCTGGGAAAACTAATAAGCTTCTTATCTACTCTGGATCCACTTTGCAATCTGGAATTCCATCAAGGTTCAGTGGCAGTGGATCTGGTACAGATTTCACTCTCACCATCAGTAGCCTGGAGCCTGAAGATTTTGCAATGTATTACTGTCAACAGCATAATGAATACCCGTAC

IGKV16-104*01_AKR	AKR/J	IGKV	MUSMUS IGKV16-104*01 F	100	GATGTCCAGATAACCCAGTCTCCATCTTATCTTGCTGCATCTCCTGGAGAAACCATTACTATTAATTGCAGGGCAAGTAAGAGCATTAGCAAATATTTAGCCTGGTATCAAGAGAAACCTGGGAAAACTAATAAGCTTCTTATCTACTCTGGATCCACTTTGCAATCTGGAATTCCATCAAGGTTCAGTGGCAGTGGATCTGGTACAGATTTCACTCTCACCATCAGTAGCCTGGAGCCTGAAGATTTTGCAATGTATTACTGTCAACAGCATAATGAATACCCGTAC
IGKV16-104*01_B6	C57BL/6J	IGKV	MUSMUS IGKV16-104*01 F	100	GATGTCCAGATAACCCAGTCTCCATCTTATCTTGCTGCATCTCCTGGAGAAACCATTACTATTAATTGCAGGGCAAGTAAGAGCATTAGCAAATATTTAGCCTGGTATCAAGAGAAACCTGGGAAAACTAATAAGCTTCTTATCTACTCTGGATCCACTTTGCAATCTGGAATTCCATCAAGGTTCAGTGGCAGTGGATCTGGTACAGATTTCACTCTCACCATCAGTAGCCTGGAGCCTGAAGATTTTGCAATGTATTACTGTCAACAGCATAATGAATACCCGTAC
IGKV16-104*01_BALB	BALB/CBYJ	IGKV	MUSMUS IGKV16-104*01 F	100	GATGTCCAGATAACCCAGTCTCCATCTTATCTTGCTGCATCTCCTGGAGAAACCATTACTATTAATTGCAGGGCAAGTAAGAGCATTAGCAAATATTTAGCCTGGTATCAAGAGAAACCTGGGAAAACTAATAAGCTTCTTATCTACTCTGGATCCACTTTGCAATCTGGAATTCCATCAAGGTTCAGTGGCAGTGGATCTGGTACAGATTTCACTCTCACCATCAGTAGCCTGGAGCCTGAAGATTTTGCAATGTATTACTGTCAACAGCATAATGAATACCCGTAC
IGKV16-104*01_C3H	C3H/HEJ	IGKV	MUSMUS IGKV16-104*01 F	100	GATGTCCAGATAACCCAGTCTCCATCTTATCTTGCTGCATCTCCTGGAGAAACCATTACTATTAATTGCAGGGCAAGTAAGAGCATTAGCAAATATTTAGCCTGGTATCAAGAGAAACCTGGGAAAACTAATAAGCTTCTTATCTACTCTGGATCCACTTTGCAATCTGGAATTCCATCAAGGTTCAGTGGCAGTGGATCTGGTACAGATTTCACTCTCACCATCAGTAGCCTGGAGCCTGAAGATTTTGCAATGTATTACTGTCAACAGCATAATGAATACCCGTAC

IGKV16-104*01_CBA	CBA/J	IGKV	MUSMUS IGKV16-104*01 F	100	GATGTCCAGATAACCCAGTCTCCATCTTATCTTGCTGCATCTCCTGGAGAAACCATTACTATTAATTGCAGGGCAAGTAAGAGCATTAGCAAATATTTAGCCTGGTATCAAGAGAAACCTGGGAAAACTAATAAGCTTCTTATCTACTCTGGATCCACTTTGCAATCTGGAATTCCATCAAGGTTCAGTGGCAGTGGATCTGGTACAGATTTCACTCTCACCATCAGTAGCCTGGAGCCTGAAGATTTTGCAATGTATTACTGTCAACAGCATAATGAATACCCGTAC
IGKV16-104*01_DBA1	DBA/1J	IGKV	MUSMUS IGKV16-104*01 F	100	GATGTCCAGATAACCCAGTCTCCATCTTATCTTGCTGCATCTCCTGGAGAAACCATTACTATTAATTGCAGGGCAAGTAAGAGCATTAGCAAATATTTAGCCTGGTATCAAGAGAAACCTGGGAAAACTAATAAGCTTCTTATCTACTCTGGATCCACTTTGCAATCTGGAATTCCATCAAGGTTCAGTGGCAGTGGATCTGGTACAGATTTCACTCTCACCATCAGTAGCCTGGAGCCTGAAGATTTTGCAATGTATTACTGTCAACAGCATAATGAATACCCGTAC
IGKV16-104*01_DBA2	DBA/2J	IGKV	MUSMUS IGKV16-104*01 F	100	GATGTCCAGATAACCCAGTCTCCATCTTATCTTGCTGCATCTCCTGGAGAAACCATTACTATTAATTGCAGGGCAAGTAAGAGCATTAGCAAATATTTAGCCTGGTATCAAGAGAAACCTGGGAAAACTAATAAGCTTCTTATCTACTCTGGATCCACTTTGCAATCTGGAATTCCATCAAGGTTCAGTGGCAGTGGATCTGGTACAGATTTCACTCTCACCATCAGTAGCCTGGAGCCTGAAGATTTTGCAATGTATTACTGTCAACAGCATAATGAATACCCGTAC
IGKV16-104*01_MRL	MRL/MPJ	IGKV	MUSMUS IGKV16-104*01 F	100	GATGTCCAGATAACCCAGTCTCCATCTTATCTTGCTGCATCTCCTGGAGAAACCATTACTATTAATTGCAGGGCAAGTAAGAGCATTAGCAAATATTTAGCCTGGTATCAAGAGAAACCTGGGAAAACTAATAAGCTTCTTATCTACTCTGGATCCACTTTGCAATCTGGAATTCCATCAAGGTTCAGTGGCAGTGGATCTGGTACAGATTTCACTCTCACCATCAGTAGCCTGGAGCCTGAAGATTTTGCAATGTATTACTGTCAACAGCATAATGAATACCCGTAC

IGKV16-104*01_S2488_PWD	PWD/PHJ	IGKV	MUSMUS IGKV14-87-2*01 F	99.28	GACATCCAAATGACCCAGTCCTCATCATTTCTCTCTGCATCTCTAGGAGATCATCTTACAATCAACTGCAGGGCCAGTAAGGATATTAACAAGTATTTTGCTTGGGTTCAACAGAAGCCAGGGAAGGCTCCAAGGATGTTGATTCATTTTGCTTCCACCTTGCTACCTGGGGTTCCAGAAAAGTTCAGTGGGAGTGGATCTGGGACAGATTTTTCTCTCACTATCAGAAACATAGAGTCCGAAGATATTGCAATGTATTACTGTCTACAGTATTCTGAGCATCCGTAC
IGKV16-104*01_S2563_PWD	PWD/PHJ	IGKV	MUSMUS IGKV14-87-2*01 F	98.21	GACATCCAAATGACCCAGTCCTCATCATTTCTCTCTGCATCTCTAGGAGATCATCTTACAATCAACTGCAGGGCCAGTAAGGATATTAACAAGTATTTTGCTTGGGTTCAACAGAAGCCAGGGAAGGCTCCAAGGATGTTGATTCATTTTGCTTCCACCTTGCTACCTGGGGTTCCAGAAAAGTTCAGTGGGAGTGGATCTGGGACAGATTTTTCTCTCACTGTCAGAAACATAGAGTCCGAAGATATTGCAATGTATTACTGTCTACAGTTTTCTGAACATCCGTAC
IGKV16-104*01_S6011_CAST	CAST/EIJ	IGKV	MUSMUS IGKV16-104*01 F	100	GATGTCCAGATAACCCAGTCTCCATCTTATCTTGCTGCATCTCCTGGAGAAACCATTACTATTAATTGCAGGGCAAGTAAGAGCATTAGCAAATATTTAGCCTGGTATCAAGAGAAACCTGGGAAAACTAATAAGCTTCTTATCTACTCTGGATCCACTTTGCAATCTGGAATTCCATCAAGGTTCAGTGGCAGTGGATCTGGTACAGATTTCACTCTCACCATCAGTAGCCTGGAGCCTGAAGATTTTGCAATGTATTACTGTCAACAGCATAATGAATCCCCGTAC
IGKV16-104*01_S6011_NOD	NOD/SHILTJ	IGKV	MUSMUS IGKV16-104*01 F	100	GATGTCCAGATAACCCAGTCTCCATCTTATCTTGCTGCATCTCCTGGAGAAACCATTACTATTAATTGCAGGGCAAGTAAGAGCATTAGCAAATATTTAGCCTGGTATCAAGAGAAACCTGGGAAAACTAATAAGCTTCTTATCTACTCTGGATCCACTTTGCAATCTGGAATTCCATCAAGGTTCAGTGGCAGTGGATCTGGTACAGATTTCACTCTCACCATCAGTAGCCTGGAGCCTGAAGATTTTGCAATGTATTACTGTCAACAGCATAATGAATCCCCGTAC

IGKV16-104*01_S6011_NOR	NOR/LTJ	IGKV	MUSMUS IGKV16-104*01 F	100	GATGTCCAGATAACCCAGTCTCCATCTTATCTTGCTGCATCTCCTGGAGAAACCATTACTATTAATTGCAGGGCAAGTAAGAGCATTAGCAAATATTTAGCCTGGTATCAAGAGAAACCTGGGAAAACTAATAAGCTTCTTATCTACTCTGGATCCACTTTGCAATCTGGAATTCCATCAAGGTTCAGTGGCAGTGGATCTGGTACAGATTTCACTCTCACCATCAGTAGCCTGGAGCCTGAAGATTTTGCAATGTATTACTGTCAACAGCATAATGAATCCCCGTAC
IGKV16-104*01_S6011_NZB	NZB/BLNJ	IGKV	MUSMUS IGKV16-104*01 F	100	GATGTCCAGATAACCCAGTCTCCATCTTATCTTGCTGCATCTCCTGGAGAAACCATTACTATTAATTGCAGGGCAAGTAAGAGCATTAGCAAATATTTAGCCTGGTATCAAGAGAAACCTGGGAAAACTAATAAGCTTCTTATCTACTCTGGATCCACTTTGCAATCTGGAATTCCATCAAGGTTCAGTGGCAGTGGATCTGGTACAGATTTCACTCTCACCATCAGTAGCCTGGAGCCTGAAGATTTTGCAATGTATTACTGTCAACAGCATAATGAATCCCCGTAC
IGKV16-104*01_S7081_PWD	PWD/PHJ	IGKV	MUSMUS IGKV16-104*01 F	98.21	GATGTCCAGATAACCCAGTCTCCATCTTATCTTGCTGCATCTCCTGGAGAAACCATTACTATTAATTGCAGGGCAAGTAAGAGCATTAACAAATATTTAGCCTGGTATCAAGAGAAACCTGGGAAAACTAATAAGCTTCTTATCTACTCTGGATCCACCTTGCAATCTGGAATTCCATCAAGGTTCAGTGGCAGTGGATCTGGTACAGATTTTACTCTCACCATCAGTAGCTTGGAGCCTGAAGATTTTGCAATGTATCACTGTCAACAGCATAATGAATACCCGTAC
IGKV16-104*01_S7508_MSM	MSM/MSJ	IGKV	MUSMUS IGKV16-104*01 F	98.92	GATGTCCAGATAACCCAGTCTCCATCTTATCTTGCTGCATCTCCTGGAGAAACCATTACTATTAATTGCAGGGCAAGTAAGAGCATTAGGACATATTTAGCCTGGTATCAAGAGAAACCTGGGAAAACTAATAAGCTTCTTATCTACTCTGGATCCACTTTGCAATCTGGAATTCCATCAAGGTTCAGTGGCAGTGGATCTGGTACAGATTTTACTCTCACCATCAGTAGCCTGGAGCCTGAAGATTTTGCAATGTATTACTGTCAACAGCATAATGAATACCCGTAC

IGKV16-104*01_S8917_NOD	NOD/SHILTJ	IGKV	MUSMUS IGKV16-104*01 F	99.64	GATGTCCAGATAACCCAGTCTCCATCTTATCTTGCTGCATCTCCTGGAGAAACCATTACTATTAATTGCAGGGCAAGTAAGAGCATTAGCAAATATTTAGCCTGGTATCAAGAGAAACCTGGGAAAACTAATAAGCTTCTTATCTACTCTGGATCCACTTTGCAATCTGGAATTCCATCAAGGTTCAGTGGCAGTGGATCTGGTACAGATTTCACTCTCACCATCAGTAGCCTGGAGCCTGAAGATTTTGCAATGTATTACTGTCAACAACATAATGAATCCCCGTAC
IGKV16-104*01_S9985_AKR	AKR/J	IGKV	MUSMUS IGKV14-87-2*01 F	99.64	GACATCCAAATGACCCAGTCCTCATCATTTCTCTCTGCATCTCTAGGAGATCATCTTACAATCAACTGCAGGGCCAGTAAGGATATTAACAAGTATTTTGCTTGGGTTCAACAGAAGCCAGGGAAGGCTCCAAGGATGTTGATTCATTTTGCTTCCACCTTGCTACCTGGGGTTCCAGAAAAGTTCAGTGGGAGTGGATCTGGGACAGATTTTTCTCTCACTATCAGAAACATAGAGTCTGAAGATATTGCAATGTATTACTGTCTACAGTATTCTGAGCATCCGTA
IGKV16-104*01_S9985_NOR	NOR/LTJ	IGKV	MUSMUS IGKV14-87-2*01 F	99.64	GACATCCAAATGACCCAGTCCTCATCATTTCTCTCTGCATCTCTAGGAGATCATCTTACAATCAACTGCAGGGCCAGTAAGGATATTAACAAGTATTTTGCTTGGGTTCAACAGAAGCCAGGGAAGGCTCCAAGGATGTTGATTCATTTTGCTTCCACCTTGCTACCTGGGGTTCCAGAAAAGTTCAGTGGGAGTGGATCTGGGACAGATTTTTCTCTCACTATCAGAAACATAGAGTCTGAAGATATTGCAATGTATTACTGTCTACAGTATTCTGAGCATCCGTA
IGKV16-104*01_SJL	SJL/J	IGKV	MUSMUS IGKV16-104*01 F	100	GATGTCCAGATAACCCAGTCTCCATCTTATCTTGCTGCATCTCCTGGAGAAACCATTACTATTAATTGCAGGGCAAGTAAGAGCATTAGCAAATATTTAGCCTGGTATCAAGAGAAACCTGGGAAAACTAATAAGCTTCTTATCTACTCTGGATCCACTTTGCAATCTGGAATTCCATCAAGGTTCAGTGGCAGTGGATCTGGTACAGATTTCACTCTCACCATCAGTAGCCTGGAGCCTGAAGATTTTGCAATGTATTACTGTCAACAGCATAATGAATACCCGTAC

IGKV17-121*01_129	129S1/SVIMJ	IGKV	MUSMUS IGKV17-121*01 F	100	GAAACAACTGTGACCCAGTCTCCAGCATCCCTGTCCATGGCTATAGGAGAAAAAGTCACCATCAGATGCATAACCAGCACTGATATTGATGATGATATGAACTGGTACCAGCAGAAGCCAGGGGAACCTCCTAAGCTCCTTATTTCAGAAGGCAATACTCTTCGTCCTGGAGTCCCATCCCGATTCTCCAGCAGTGGCTATGGTACAGATTTTGTTTTTACAATTGAAAACATGCTCTCAGAAGATGTTGCAGATTACTACTGTTTGCAAAGTGATAACTTGCCTCT
IGKV17-121*01_AJ	A/J	IGKV	MUSMUS IGKV17-121*01 F	100	GAAACAACTGTGACCCAGTCTCCAGCATCCCTGTCCATGGCTATAGGAGAAAAAGTCACCATCAGATGCATAACCAGCACTGATATTGATGATGATATGAACTGGTACCAGCAGAAGCCAGGGGAACCTCCTAAGCTCCTTATTTCAGAAGGCAATACTCTTCGTCCTGGAGTCCCATCCCGATTCTCCAGCAGTGGCTATGGTACAGATTTTGTTTTTACAATTGAAAACATGCTCTCAGAAGATGTTGCAGATTACTACTGTTTGCAAAGTGATAACTTGCCTCT
IGKV17-121*01_AKR	AKR/J	IGKV	MUSMUS IGKV17-121*01 F	100	GAAACAACTGTGACCCAGTCTCCAGCATCCCTGTCCATGGCTATAGGAGAAAAAGTCACCATCAGATGCATAACCAGCACTGATATTGATGATGATATGAACTGGTACCAGCAGAAGCCAGGGGAACCTCCTAAGCTCCTTATTTCAGAAGGCAATACTCTTCGTCCTGGAGTCCCATCCCGATTCTCCAGCAGTGGCTATGGTACAGATTTTGTTTTTACAATTGAAAACATGCTCTCAGAAGATGTTGCAGATTACTACTGTTTGCAAAGTGATAACTTGCCTCT
IGKV17-121*01_B6	C57BL/6J	IGKV	MUSMUS IGKV17-121*01 F	100	GAAACAACTGTGACCCAGTCTCCAGCATCCCTGTCCATGGCTATAGGAGAAAAAGTCACCATCAGATGCATAACCAGCACTGATATTGATGATGATATGAACTGGTACCAGCAGAAGCCAGGGGAACCTCCTAAGCTCCTTATTTCAGAAGGCAATACTCTTCGTCCTGGAGTCCCATCCCGATTCTCCAGCAGTGGCTATGGTACAGATTTTGTTTTTACAATTGAAAACATGCTCTCAGAAGATGTTGCAGATTACTACTGTTTGCAAAGTGATAACTTGCCTCT

IGKV17-121*01_BALB	BALB/CBYJ	IGKV	MUSMUS IGKV17-121*01 F	100	GAAACAACTGTGACCCAGTCTCCAGCATCCCTGTCCATGGCTATAGGAGAAAAAGTCACCATCAGATGCATAACCAGCACTGATATTGATGATGATATGAACTGGTACCAGCAGAAGCCAGGGGAACCTCCTAAGCTCCTTATTTCAGAAGGCAATACTCTTCGTCCTGGAGTCCCATCCCGATTCTCCAGCAGTGGCTATGGTACAGATTTTGTTTTTACAATTGAAAACATGCTCTCAGAAGATGTTGCAGATTACTACTGTTTGCAAAGTGATAACTTGCCTCT
IGKV17-121*01_C3H	C3H/HEJ	IGKV	MUSMUS IGKV17-121*01 F	100	GAAACAACTGTGACCCAGTCTCCAGCATCCCTGTCCATGGCTATAGGAGAAAAAGTCACCATCAGATGCATAACCAGCACTGATATTGATGATGATATGAACTGGTACCAGCAGAAGCCAGGGGAACCTCCTAAGCTCCTTATTTCAGAAGGCAATACTCTTCGTCCTGGAGTCCCATCCCGATTCTCCAGCAGTGGCTATGGTACAGATTTTGTTTTTACAATTGAAAACATGCTCTCAGAAGATGTTGCAGATTACTACTGTTTGCAAAGTGATAACTTGCCTCT
IGKV17-121*01_CBA	CBA/J	IGKV	MUSMUS IGKV17-121*01 F	100	GAAACAACTGTGACCCAGTCTCCAGCATCCCTGTCCATGGCTATAGGAGAAAAAGTCACCATCAGATGCATAACCAGCACTGATATTGATGATGATATGAACTGGTACCAGCAGAAGCCAGGGGAACCTCCTAAGCTCCTTATTTCAGAAGGCAATACTCTTCGTCCTGGAGTCCCATCCCGATTCTCCAGCAGTGGCTATGGTACAGATTTTGTTTTTACAATTGAAAACATGCTCTCAGAAGATGTTGCAGATTACTACTGTTTGCAAAGTGATAACTTGCCTCT
IGKV17-121*01_DBA1	DBA/1J	IGKV	MUSMUS IGKV17-121*01 F	100	GAAACAACTGTGACCCAGTCTCCAGCATCCCTGTCCATGGCTATAGGAGAAAAAGTCACCATCAGATGCATAACCAGCACTGATATTGATGATGATATGAACTGGTACCAGCAGAAGCCAGGGGAACCTCCTAAGCTCCTTATTTCAGAAGGCAATACTCTTCGTCCTGGAGTCCCATCCCGATTCTCCAGCAGTGGCTATGGTACAGATTTTGTTTTTACAATTGAAAACATGCTCTCAGAAGATGTTGCAGATTACTACTGTTTGCAAAGTGATAACTTGCCTCT

IGKV17-121*01_DBA2	DBA/2J	IGKV	MUSMUS IGKV17-121*01 F	100	GAAACAACTGTGACCCAGTCTCCAGCATCCCTGTCCATGGCTATAGGAGAAAAAGTCACCATCAGATGCATAACCAGCACTGATATTGATGATGATATGAACTGGTACCAGCAGAAGCCAGGGGAACCTCCTAAGCTCCTTATTTCAGAAGGCAATACTCTTCGTCCTGGAGTCCCATCCCGATTCTCCAGCAGTGGCTATGGTACAGATTTTGTTTTTACAATTGAAAACATGCTCTCAGAAGATGTTGCAGATTACTACTGTTTGCAAAGTGATAACTTGCCTCT
IGKV17-121*01_MRL	MRL/MPJ	IGKV	MUSMUS IGKV17-121*01 F	100	GAAACAACTGTGACCCAGTCTCCAGCATCCCTGTCCATGGCTATAGGAGAAAAAGTCACCATCAGATGCATAACCAGCACTGATATTGATGATGATATGAACTGGTACCAGCAGAAGCCAGGGGAACCTCCTAAGCTCCTTATTTCAGAAGGCAATACTCTTCGTCCTGGAGTCCCATCCCGATTCTCCAGCAGTGGCTATGGTACAGATTTTGTTTTTACAATTGAAAACATGCTCTCAGAAGATGTTGCAGATTACTACTGTTTGCAAAGTGATAACTTGCCTCT
IGKV17-121*01_S5117_MSM	MSM/MSJ	IGKV	MUSMUS IGKV17-127*01 F	98.57	GAAACAACTGTGACCCAGTCTCCAGCATCCCTGTCCATGGCTACAGGAGAAAAAGTCACTATCAGATGCATAACCAGCACTGATATTGATGATGATATGAACTGGTACCAGCAGAAGCCAGGGGAACCTCCTAAGCTCCTTATTTCAGAAGGCAATACTCTTCGTCCTGAAGTCCCATCCCGATTCTCCAGCAGTGGCTATGGTACAGATTTTGTTTTTACAATTGAAAACACTCTCTCAGAAGATGTTGCAGATTACTACTGTTTGCAAAGTGATAACTTGCCTCT
IGKV17-121*01_SJL	SJL/J	IGKV	MUSMUS IGKV17-121*01 F	100	GAAACAACTGTGACCCAGTCTCCAGCATCCCTGTCCATGGCTATAGGAGAAAAAGTCACCATCAGATGCATAACCAGCACTGATATTGATGATGATATGAACTGGTACCAGCAGAAGCCAGGGGAACCTCCTAAGCTCCTTATTTCAGAAGGCAATACTCTTCGTCCTGGAGTCCCATCCCGATTCTCCAGCAGTGGCTATGGTACAGATTTTGTTTTTACAATTGAAAACATGCTCTCAGAAGATGTTGCAGATTACTACTGTTTGCAAAGTGATAACTTGCCTCT

IGKV17-127*01_129	129S1/SVIMJ	IGKV	MUSMUS IGKV17-127*01 F	100	GAAACAACTGTGACCCAGTCTC<br>CAGCATCCCTGTCCGTGGCTAC<br>AGGAGAAAAAGTCACTATCAGAT<br>GCATAACCAGCACTGATATTGAT<br>GATGATATGAACTGGTACCAGC<br>AGAAGCCAGGGGAACCTCCTA<br>AGCTCCTTATTTCAGAAGGCAAT<br>ACTCTTCGTCCTGGAGTCCCAT<br>CCCGATTCTCCAGCAGTGGCTA<br>TGGCACAGATTTTGTTTTTACAAT<br>TGAAAACACGCTCTCAGAAGAT<br>GTTGCAGATTACTACTGTTTGCA<br>AAGTGATAACATGCCTCT
IGKV17-127*01_AJ	A/J	IGKV	MUSMUS IGKV17-127*01 F	100	GAAACAACTGTGACCCAGTCTC<br>CAGCATCCCTGTCCGTGGCTAC<br>AGGAGAAAAAGTCACTATCAGAT<br>GCATAACCAGCACTGATATTGAT<br>GATGATATGAACTGGTACCAGC<br>AGAAGCCAGGGGAACCTCCTA<br>AGCTCCTTATTTCAGAAGGCAAT<br>ACTCTTCGTCCTGGAGTCCCAT<br>CCCGATTCTCCAGCAGTGGCTA<br>TGGCACAGATTTTGTTTTTACAAT<br>TGAAAACACGCTCTCAGAAGAT<br>GTTGCAGATTACTACTGTTTGCA<br>AAGTGATAACATGCCTCT
IGKV17-127*01_AKR	AKR/J	IGKV	MUSMUS IGKV17-127*01 F	100	GAAACAACTGTGACCCAGTCTC<br>CAGCATCCCTGTCCGTGGCTAC<br>AGGAGAAAAAGTCACTATCAGAT<br>GCATAACCAGCACTGATATTGAT<br>GATGATATGAACTGGTACCAGC<br>AGAAGCCAGGGGAACCTCCTA<br>AGCTCCTTATTTCAGAAGGCAAT<br>ACTCTTCGTCCTGGAGTCCCAT<br>CCCGATTCTCCAGCAGTGGCTA<br>TGGCACAGATTTTGTTTTTACAAT<br>TGAAAACACGCTCTCAGAAGAT<br>GTTGCAGATTACTACTGTTTGCA<br>AAGTGATAACATGCCTCT
IGKV17-127*01_B6	C57BL/6J	IGKV	MUSMUS IGKV17-127*01 F	100	GAAACAACTGTGACCCAGTCTC<br>CAGCATCCCTGTCCGTGGCTAC<br>AGGAGAAAAAGTCACTATCAGAT<br>GCATAACCAGCACTGATATTGAT<br>GATGATATGAACTGGTACCAGC<br>AGAAGCCAGGGGAACCTCCTA<br>AGCTCCTTATTTCAGAAGGCAAT<br>ACTCTTCGTCCTGGAGTCCCAT<br>CCCGATTCTCCAGCAGTGGCTA<br>TGGCACAGATTTTGTTTTTACAAT<br>TGAAAACACGCTCTCAGAAGAT<br>GTTGCAGATTACTACTGTTTGCA<br>AAGTGATAACATGCCTCT

IGKV17-127*01_BALB	BALB/CBY J	IGKV	MUSMUS IGKV17-127*01 F	100	GAAACAACTGTGACCCAGTCTCCAGCATCCCTGTCCGTGGCTACAGGAGAAAAAGTCACTATCAGATGCATAACCAGCACTGATATTGATGATGATATGAACTGGTACCAGCAGAAGCCAGGGGAACCTCCTAAGCTCCTTATTTCAGAAGGCAATACTCTTCGTCCTGGAGTCCCATCCCGATTCTCCAGCAGTGGCTATGGCACAGATTTTGTTTTTACAATTGAAAACACGCTCTCAGAAGATGTTGCAGATTACTACTGTTTGCAAAGTGATAACATGCCTCT
IGKV17-127*01_C3H	C3H/HEJ	IGKV	MUSMUS IGKV17-127*01 F	100	GAAACAACTGTGACCCAGTCTCCAGCATCCCTGTCCGTGGCTACAGGAGAAAAAGTCACTATCAGATGCATAACCAGCACTGATATTGATGATGATATGAACTGGTACCAGCAGAAGCCAGGGGAACCTCCTAAGCTCCTTATTTCAGAAGGCAATACTCTTCGTCCTGGAGTCCCATCCCGATTCTCCAGCAGTGGCTATGGCACAGATTTTGTTTTTACAATTGAAAACACGCTCTCAGAAGATGTTGCAGATTACTACTGTTTGCAAAGTGATAACATGCCTCT
IGKV17-127*01_CBA	CBA/J	IGKV	MUSMUS IGKV17-127*01 F	100	GAAACAACTGTGACCCAGTCTCCAGCATCCCTGTCCGTGGCTACAGGAGAAAAAGTCACTATCAGATGCATAACCAGCACTGATATTGATGATGATATGAACTGGTACCAGCAGAAGCCAGGGGAACCTCCTAAGCTCCTTATTTCAGAAGGCAATACTCTTCGTCCTGGAGTCCCATCCCGATTCTCCAGCAGTGGCTATGGCACAGATTTTGTTTTTACAATTGAAAACACGCTCTCAGAAGATGTTGCAGATTACTACTGTTTGCAAAGTGATAACATGCCTCT
IGKV17-127*01_DBA1	DBA/1J	IGKV	MUSMUS IGKV17-127*01 F	100	GAAACAACTGTGACCCAGTCTCCAGCATCCCTGTCCGTGGCTACAGGAGAAAAAGTCACTATCAGATGCATAACCAGCACTGATATTGATGATGATATGAACTGGTACCAGCAGAAGCCAGGGGAACCTCCTAAGCTCCTTATTTCAGAAGGCAATACTCTTCGTCCTGGAGTCCCATCCCGATTCTCCAGCAGTGGCTATGGCACAGATTTTGTTTTTACAATTGAAAACACGCTCTCAGAAGATGTTGCAGATTACTACTGTTTGCAAAGTGATAACATGCCTCT

IGKV17-127*01_DBA2	DBA/2J	IGKV	MUSMUS IGKV17-127*01 F	100	GAAACAACTGTGACCCAGTCTCCAGCATCCCTGTCCGTGGCTACAGGAGAAAAAGTCACTATCAGATGCATAACCAGCACTGATATTGATGATGATATGAACTGGTACCAGCAGAAGCCAGGGGAACCTCCTAAGCTCCTTATTTCAGAAGGCAATACTCTTCGTCCTGGAGTCCCATCCCGATTCTCCAGCAGTGGCTATGGCACAGATTTTGTTTTTACAATTGAAAACACGCTCTCAGAAGATGTTGCAGATTACTACTGTTTGCAAAGTGATAACATGCCTCT
IGKV17-127*01_MRL	MRL/MPJ	IGKV	MUSMUS IGKV17-127*01 F	100	GAAACAACTGTGACCCAGTCTCCAGCATCCCTGTCCGTGGCTACAGGAGAAAAAGTCACTATCAGATGCATAACCAGCACTGATATTGATGATGATATGAACTGGTACCAGCAGAAGCCAGGGGAACCTCCTAAGCTCCTTATTTCAGAAGGCAATACTCTTCGTCCTGGAGTCCCATCCCGATTCTCCAGCAGTGGCTATGGCACAGATTTTGTTTTTACAATTGAAAACACGCTCTCAGAAGATGTTGCAGATTACTACTGTTTGCAAAGTGATAACATGCCTCT
IGKV17-127*01_S0118_129	129S1/SVIMJ	IGKV	MUSMUS IGKV17-127*01 F	100	GAAACAACTGTGACCCAGTCTCCAGCATCCCTGTCCGTGGCTACAGGAGAAAAAGTCACTATCAGATGCATAACCAGCACTGATATTGATGATGATATGAACTGGTACCAGCAGAAGCCAGGGGAACCTCCTAAGCTCCTTATTTCAGAAGGCAATACTCTTCGTCCTGGAGTCCCATCCCGATTCTCCAGCAGTGGCTATGGCACAGATTTTGTTTTTACAATTGAAAACACGCTCTCAGAAGATGTTGCAGATTACTACTGTTTGCAAAGTGATAACATGCCGCT
IGKV17-127*01_S3263_NOR	NOR/LTJ	IGKV	MUSMUS IGKV17-127*01 F	99.64	GAAACAACTGTGACCCAGTCTCCAGCATCCCTGTCCGTGGCTACAGGAGAAAAAGTCACTATCAGATGCATAACCAACACTGATATTGATGATGATATGAACTGGTACCAGCAGAAGCCAGGGGAACCTCCTAAGCTCCTTATTTCAGAAGGCAATACTCTTCGTCCTGGAGTCCCATCCCGATTCTCCAGCAGTGGCTATGGCACAGATTTTGTTTTTACAATTGAAAACACGCTCTCAGAAGATGTTGCAGATTACTACTGTTTGCAAAGTGATAACATGCCGCT

IGKV17-127*01_S4378_CAST	CAST/EIJ	IGKV	MUSMUS IGKV17-127*01 F	99.64	GAAACAACTGTGACCCAGTCTCCAGCATCCCTGTCCGTGGCTACAGGAGAAAAAGTCACTATCAGATGCATAACCAACACTGATATTGATGATGATATGAACTGGTACCAGCAGAAGCCAGGGGAACCTCCTAAGCTCCTTATTTCAGAAGGCAATACTCTTCGTCCTGGAGTCCCATCCCGATTCTCCAGCAGTGGCTATGGCACAGATTTTGTTTTTACAATTGAAAACACGCTCTCAGAAGATGTTGCAGATTACTACTGTTTGCAAAGTGATAACATGCCTCT
IGKV17-127*01_S4378_NOD	NOD/SHILTJ	IGKV	MUSMUS IGKV17-127*01 F	99.64	GAAACAACTGTGACCCAGTCTCCAGCATCCCTGTCCGTGGCTACAGGAGAAAAAGTCACTATCAGATGCATAACCAACACTGATATTGATGATGATATGAACTGGTACCAGCAGAAGCCAGGGGAACCTCCTAAGCTCCTTATTTCAGAAGGCAATACTCTTCGTCCTGGAGTCCCATCCCGATTCTCCAGCAGTGGCTATGGCACAGATTTTGTTTTTACAATTGAAAACACGCTCTCAGAAGATGTTGCAGATTACTACTGTTTGCAAAGTGATAACATGCCTCT
IGKV17-127*01_S4378_NOR	NOR/LTJ	IGKV	MUSMUS IGKV17-127*01 F	99.64	GAAACAACTGTGACCCAGTCTCCAGCATCCCTGTCCGTGGCTACAGGAGAAAAAGTCACTATCAGATGCATAACCAACACTGATATTGATGATGATATGAACTGGTACCAGCAGAAGCCAGGGGAACCTCCTAAGCTCCTTATTTCAGAAGGCAATACTCTTCGTCCTGGAGTCCCATCCCGATTCTCCAGCAGTGGCTATGGCACAGATTTTGTTTTTACAATTGAAAACACGCTCTCAGAAGATGTTGCAGATTACTACTGTTTGCAAAGTGATAACATGCCTCT
IGKV17-127*01_S4378_NZB	NZB/BLNJ	IGKV	MUSMUS IGKV17-127*01 F	99.64	GAAACAACTGTGACCCAGTCTCCAGCATCCCTGTCCGTGGCTACAGGAGAAAAAGTCACTATCAGATGCATAACCAACACTGATATTGATGATGATATGAACTGGTACCAGCAGAAGCCAGGGGAACCTCCTAAGCTCCTTATTTCAGAAGGCAATACTCTTCGTCCTGGAGTCCCATCCCGATTCTCCAGCAGTGGCTATGGCACAGATTTTGTTTTTACAATTGAAAACACGCTCTCAGAAGATGTTGCAGATTACTACTGTTTGCAAAGTGATAACATGCCTCT

IGKV17-127*01_S9169_MSM	MSM/MSJ	IGKV	MUSMUS IGKV17-127*01 F	95.7	GAAACAACTGTGACCCAGTCTCCAGCATCCCTGTCCATGGCTACAGGAGAAAAAGTCACTATCAGTTGCATCACCAGCACTGATATTGATGATGATATGAACTGGTACCAGCAGAAGCCCGGAGAAGCTCCTAAGCTCCTTATTTCAGAAGGCAATACTCTTCGTCCTGGAGTCCCATCCCGGTTCTCCAGTAGTGGCTATGGCACAGATTTTGTTTTTACAATTGAAAACACTCTCTCAGAAGATGTTGCAGATTACTTCTGTCAGCAAAGTGATAACCTGCCGCT
IGKV17-127*01_SJL	SJL/J	IGKV	MUSMUS IGKV17-127*01 F	100	GAAACAACTGTGACCCAGTCTCCAGCATCCCTGTCCGTGGCTACAGGAGAAAAAGTCACTATCAGATGCATAACCAGCACTGATATTGATGATGATATGAACTGGTACCAGCAGAAGCCAGGGGAACCTCCTAAGCTCCTTATTTCAGAAGGCAATACTCTTCGTCCTGGAGTCCCATCCCGATTCTCCAGCAGTGGCTATGGCACAGATTTTGTTTTTACAATTGAAAACACGCTCTCAGAAGATGTTGCAGATTACTACTGTTTGCAAAGTGATAACATGCCTCT
IGKV18-36*01_AJ	A/J	IGKV	MUSMUS IGKV18-36*01 F	100	ACTGGAGAAACAACACAGGCTCCAGCTTCTCTGAGTTTTTCTCTTGGTGAAACAGCAACACTGTCATGCAGGTCCAGTGAGAGTGTTGGCAGCTACTTAGCCTGGTACCAGCAGAAAGCAGAGCAAGTTCCCCGGCTCCTTATCCATAGTGCCTCCACTAGGGCCGGTGGTGTCCCAGTCCGGTTCAGTGGCACTGGGTCTGGGACAGACTTCACTCTCACCATCAGCAGTCTAGAACCTGAAGATGCTGCAGTTTACTACTGTCAACCTTTCAAAAGTTGGTCATA
IGKV18-36*01_DBA2	DBA/2J	IGKV	MUSMUS IGKV18-36*01 F	100	ACTGGAGAAACAACACAGGCTCCAGCTTCTCTGAGTTTTTCTCTTGGTGAAACAGCAACACTGTCATGCAGGTCCAGTGAGAGTGTTGGCAGCTACTTAGCCTGGTACCAGCAGAAAGCAGAGCAAGTTCCCCGGCTCCTTATCCATAGTGCCTCCACTAGGGCCGGTGGTGTCCCAGTCCGGTTCAGTGGCACTGGGTCTGGGACAGACTTCACTCTCACCATCAGCAGTCTAGAACCTGAAGATGCTGCAGTTTACTACTGTCAACCTTTCAAAAGTTGGTCATA

IGKV18-36*01_NZB	NZB/BLNJ	IGKV	MUSMUS IGKV18-36*01 F	100	ACTGGAGAAACAACACAGGCTCCAGCTTCTCTGAGTTTTTCTCTTGGTGAAACAGCAACACTGTCATGCAGGTCCAGTGAGAGTGTTGGCAGCTACTTAGCCTGGTACCAGCAGAAAGCAGAGCAAGTTCCCCGGCTCCTTATCCATAGTGCCTCCACTAGGGCCGGTGGTGTCCCAGTCCGGTTCAGTGGCACTGGGTCTGGGACAGACTTCACTCTCACCATCAGCAGTCTAGAACCTGAAGATGCTGCAGTTTACTACTGTCAACCTTTCAAAAGTTGGTCATA
IGKV18-36*01_S0889_AKR	AKR/J	IGKV	MUSMUS IGKV18-36*01 F	99.28	ACTGGAGAAACAACACAGTCTCCAGCTTCTCTGAGTTTTTCTCTTGGTGAAACAGCAACACTGTCATGCAGGTCCAGTGAGAGTGTTGGCAGCTACTTAGCCTGGTACCAGCAGAAAGCAGAGCAAGTTCCCCGGCTCCTTATCCATAGTGCCTCCACTAGGGCCGGTGGTGTCCCAGTCCGATTCAGTGGCACTGGGTCTGGGACAGACTTCACTCTCACCATCAGCAGTCTAGAACCTGAAGATGCTGCAGTTTACTACTGTCAACCTTTCAAAAGTTGGTCATA
IGKV18-36*01_S3639_CAST	CAST/EIJ	IGKV	MUSMUS IGKV18-36*01 F	99.64	ACTGGAGAAACAACACAGGCTCCAGCTTCTCTGAGTTTTTCTCTTGGTGAAACAGCAACACTGTCATGCAGGTCCAGTGAGAGTGTTGGCAGCTACTTAGCCTGGTACCAGCAGAAAGCAGAGCAAGTTCCCCGGCTCCTTATCCATAGTGCCTCCACTAGGACCGGTGGTGTCCCAGTCCGGTTCAGTGGCACTGGGTCTGGGACAGACTTCACTCTCACCATCAGCAGTCTAGAACCTGAAGATGCTGCAGTTTACTACTGTCAACCTTTCAAAAGTTGGTCATA
IGKV18-36*01_S9375_PWD	PWD/PHJ	IGKV	MUSMUS IGKV18-36*01 F	98.21	ACTGGAGAAACAACACAGTCTCCAGCCTCTCTGAGTTTTTCTCTTGGTGAAACAGCAACCCTGACATGCAGGTCCAGTGAGAGTGTTGGCAGCTACTTAGCCTGGTACCAGCAGAAAGCAGAGCAAGTTCCCCGGCTCCTTATCCATAGTGCTTCCACTAGGGCCGGTGGTGTCCCAGTCCGGTTCAGTGGCACTGGGTCTGGGACAGACTTCACTCTCACCATCAGCAGTCTAGAACCTGAAGATGCTGCAGTTTACTACTGTCAACCTTTCAAAAGTTGGTCATA

IGKV19-93*01_129	129S1/SVIMJ	IGKV	MUSMUS IGKV19-93*01 F	100	GACATCCAGATGACACAGTCTCCATCCTCACTGTCTGCATCTCTGGGAGGCAAAGTCACCATCACTTGCAAGGCAAGCCAAGACATTAACAAGTATATAGCTTGGTACCAACACAAGCCTGGAAAAGGTCCTAGGCTGCTCATACATTACACATCTACATTACAGCCAGGCATCCCATCAAGGTTCAGTGGAAGTGGGTCTGGGAGAGATTATTCCTTCAGCATCAGCAACCTGGAGCCTGAAGATATTGCAACTTATTATTGTCTACAGTATGATAATCTTCTACC
IGKV19-93*01_AJ	A/J	IGKV	MUSMUS IGKV19-93*01 F	100	GACATCCAGATGACACAGTCTCCATCCTCACTGTCTGCATCTCTGGGAGGCAAAGTCACCATCACTTGCAAGGCAAGCCAAGACATTAACAAGTATATAGCTTGGTACCAACACAAGCCTGGAAAAGGTCCTAGGCTGCTCATACATTACACATCTACATTACAGCCAGGCATCCCATCAAGGTTCAGTGGAAGTGGGTCTGGGAGAGATTATTCCTTCAGCATCAGCAACCTGGAGCCTGAAGATATTGCAACTTATTATTGTCTACAGTATGATAATCTTCTACC
IGKV19-93*01_B6	C57BL/6J	IGKV	MUSMUS IGKV19-93*01 F	100	GACATCCAGATGACACAGTCTCCATCCTCACTGTCTGCATCTCTGGGAGGCAAAGTCACCATCACTTGCAAGGCAAGCCAAGACATTAACAAGTATATAGCTTGGTACCAACACAAGCCTGGAAAAGGTCCTAGGCTGCTCATACATTACACATCTACATTACAGCCAGGCATCCCATCAAGGTTCAGTGGAAGTGGGTCTGGGAGAGATTATTCCTTCAGCATCAGCAACCTGGAGCCTGAAGATATTGCAACTTATTATTGTCTACAGTATGATAATCTTCTACC
IGKV19-93*01_BALB	BALB/CBYJ	IGKV	MUSMUS IGKV19-93*01 F	100	GACATCCAGATGACACAGTCTCCATCCTCACTGTCTGCATCTCTGGGAGGCAAAGTCACCATCACTTGCAAGGCAAGCCAAGACATTAACAAGTATATAGCTTGGTACCAACACAAGCCTGGAAAAGGTCCTAGGCTGCTCATACATTACACATCTACATTACAGCCAGGCATCCCATCAAGGTTCAGTGGAAGTGGGTCTGGGAGAGATTATTCCTTCAGCATCAGCAACCTGGAGCCTGAAGATATTGCAACTTATTATTGTCTACAGTATGATAATCTTCTACC

IGKV19-93*01_C3H	C3H/HEJ	IGKV	MUSMUS IGKV19-93*01 F	100	GACATCCAGATGACACAGTCTCCATCCTCACTGTCTGCATCTCTGGGAGGCAAAGTCACCATCACTTGCAAGGCAAGCCAAGACATTAACAAGTATATAGCTTGGTACCAACACAAGCCTGGAAAAGGTCCTAGGCTGCTCATACATTACACATCTACATTACAGCCAGGCATCCCATCAAGGTTCAGTGGAAGTGGGTCTGGGAGAGATTATTCCTTCAGCATCAGCAACCTGGAGCCTGAAGATATTGCAACTTATTATTGTCTACAGTATGATAATCTTCTACC
IGKV19-93*01_CBA	CBA/J	IGKV	MUSMUS IGKV19-93*01 F	100	GACATCCAGATGACACAGTCTCCATCCTCACTGTCTGCATCTCTGGGAGGCAAAGTCACCATCACTTGCAAGGCAAGCCAAGACATTAACAAGTATATAGCTTGGTACCAACACAAGCCTGGAAAAGGTCCTAGGCTGCTCATACATTACACATCTACATTACAGCCAGGCATCCCATCAAGGTTCAGTGGAAGTGGGTCTGGGAGAGATTATTCCTTCAGCATCAGCAACCTGGAGCCTGAAGATATTGCAACTTATTATTGTCTACAGTATGATAATCTTCTACC
IGKV19-93*01_DBA1	DBA/1J	IGKV	MUSMUS IGKV19-93*01 F	100	GACATCCAGATGACACAGTCTCCATCCTCACTGTCTGCATCTCTGGGAGGCAAAGTCACCATCACTTGCAAGGCAAGCCAAGACATTAACAAGTATATAGCTTGGTACCAACACAAGCCTGGAAAAGGTCCTAGGCTGCTCATACATTACACATCTACATTACAGCCAGGCATCCCATCAAGGTTCAGTGGAAGTGGGTCTGGGAGAGATTATTCCTTCAGCATCAGCAACCTGGAGCCTGAAGATATTGCAACTTATTATTGTCTACAGTATGATAATCTTCTACC
IGKV19-93*01_DBA2	DBA/2J	IGKV	MUSMUS IGKV19-93*01 F	100	GACATCCAGATGACACAGTCTCCATCCTCACTGTCTGCATCTCTGGGAGGCAAAGTCACCATCACTTGCAAGGCAAGCCAAGACATTAACAAGTATATAGCTTGGTACCAACACAAGCCTGGAAAAGGTCCTAGGCTGCTCATACATTACACATCTACATTACAGCCAGGCATCCCATCAAGGTTCAGTGGAAGTGGGTCTGGGAGAGATTATTCCTTCAGCATCAGCAACCTGGAGCCTGAAGATATTGCAACTTATTATTGTCTACAGTATGATAATCTTCTACC

IGKV19-93*01_LEWES	LEWES/EIJ	IGKV	MUSMUS IGKV19-93*01 F	100	GACATCCAGATGACACAGTCTCCATCCTCACTGTCTGCATCTCTGGGAGGCAAAGTCACCATCACTTGCAAGGCAAGCCAAGACATTAACAAGTATATAGCTTGGTACCAACACAAGCCTGGAAAAGGTCCTAGGCTGCTCATACATTACACATCTACATTACAGCCAGGCATCCCATCAAGGTTCAGTGGAAGTGGGTCTGGGAGAGATTATTCCTTCAGCATCAGCAACCTGGAGCCTGAAGATATTGCAACTTATTATTGTCTACAGTATGATAATCTTCTACC
IGKV19-93*01_NZB	NZB/BLNJ	IGKV	MUSMUS IGKV19-93*01 F	100	GACATCCAGATGACACAGTCTCCATCCTCACTGTCTGCATCTCTGGGAGGCAAAGTCACCATCACTTGCAAGGCAAGCCAAGACATTAACAAGTATATAGCTTGGTACCAACACAAGCCTGGAAAAGGTCCTAGGCTGCTCATACATTACACATCTACATTACAGCCAGGCATCCCATCAAGGTTCAGTGGAAGTGGGTCTGGGAGAGATTATTCCTTCAGCATCAGCAACCTGGAGCCTGAAGATATTGCAACTTATTATTGTCTACAGTATGATAATCTTCTACC
IGKV19-93*01_S0826_MSM	MSM/MSJ	IGKV	MUSMUS IGKV19-93*01 F	96.77	GACATCCAGATGACACAGTCTCCATCCTCACTGTCTGCATCTCTGGGAGGCAAAGTCACCATCACTTGCAAGGCAAGCCAAGACATTAACAAGAATATAGCTTGGTACCAACACAAGCCTGGAAAAGGTCCTAGGCTGCTCATACGTTACACATCAATACTAGAGTCAGGCATCCCATCAAGGTTCAGTGGAAGTGGGTCTGGGAGAGATTATTCCTTCAGCATCAGCAACCTGGAGCCTGAAGATATTGCAACTTATTACTGTCTACAGTATTATAATCTTCCACC
IGKV19-93*01_S8964_AKR	AKR/J	IGKV	MUSMUS IGKV19-93*01 F	98.21	GACATCCAGATGACACAGTCTCCATCCTCACTGTCTGCATCTCTGGGAGGCAAAGTCACCATCACTTGCAAGGCAAGCCAAGACATTAACAAGAATATAGCTTGGTACCAACACAAGCCTGGAAAAGGTCCTAGGCTGCTCATATGGTACACATCTACATTACAGCCAGGCATCCCATCAAGGTTCAGTGGAAGTGGGTCTGGGAGAGATTATTCCTTCAGCATCAGCAACCTGGAGCCTGAAGATATTGCAACTTATTACTGTCTACAGTATGATAATCTTCCACC

IGKV19-93*01_S8964_CAST	CAST/EIJ	IGKV	MUSMUS IGKV19-93*01 F	98.21	GACATCCAGATGACACAGTCTCCATCCTCACTGTCTGCATCTCTGGGAGGCAAAGTCACCATCACTTGCAAGGCAAGCCAAGACATTAACAAGAATATAGCTTGGTACCAACACAAGCCTGGAAAAGGTCCTAGGCTGCTCATATGGTACACATCTACATTACAGCCAGGCATCCCATCAAGGTTCAGTGGAAGTGGGTCTGGGAGAGATTATTCCTTCAGCATCAGCAACCTGGAGCCTGAAGATATTGCAACTTATTACTGTCTACAGTATGATAATCTTCCACC
IGKV19-93*01_S8964_MRL	MRL/MPJ	IGKV	MUSMUS IGKV19-93*01 F	98.21	GACATCCAGATGACACAGTCTCCATCCTCACTGTCTGCATCTCTGGGAGGCAAAGTCACCATCACTTGCAAGGCAAGCCAAGACATTAACAAGAATATAGCTTGGTACCAACACAAGCCTGGAAAAGGTCCTAGGCTGCTCATATGGTACACATCTACATTACAGCCAGGCATCCCATCAAGGTTCAGTGGAAGTGGGTCTGGGAGAGATTATTCCTTCAGCATCAGCAACCTGGAGCCTGAAGATATTGCAACTTATTACTGTCTACAGTATGATAATCTTCCACC
IGKV19-93*01_S8964_NOD	NOD/SHILTJ	IGKV	MUSMUS IGKV19-93*01 F	98.21	GACATCCAGATGACACAGTCTCCATCCTCACTGTCTGCATCTCTGGGAGGCAAAGTCACCATCACTTGCAAGGCAAGCCAAGACATTAACAAGAATATAGCTTGGTACCAACACAAGCCTGGAAAAGGTCCTAGGCTGCTCATATGGTACACATCTACATTACAGCCAGGCATCCCATCAAGGTTCAGTGGAAGTGGGTCTGGGAGAGATTATTCCTTCAGCATCAGCAACCTGGAGCCTGAAGATATTGCAACTTATTACTGTCTACAGTATGATAATCTTCCACC
IGKV19-93*01_S8964_NOR	NOR/LTJ	IGKV	MUSMUS IGKV19-93*01 F	98.21	GACATCCAGATGACACAGTCTCCATCCTCACTGTCTGCATCTCTGGGAGGCAAAGTCACCATCACTTGCAAGGCAAGCCAAGACATTAACAAGAATATAGCTTGGTACCAACACAAGCCTGGAAAAGGTCCTAGGCTGCTCATATGGTACACATCTACATTACAGCCAGGCATCCCATCAAGGTTCAGTGGAAGTGGGTCTGGGAGAGATTATTCCTTCAGCATCAGCAACCTGGAGCCTGAAGATATTGCAACTTATTACTGTCTACAGTATGATAATCTTCCACC

IGKV19-93*01_S9010_PWD	PWD/PHJ	IGKV	MUSMUS IGKV19-93*01 F	96.42	GACATCCAGATGACACAGTCTCCATCCTCACTGTCTGCATCTCTGGGAGGCAAAGTCACCATCACTTGCAAGGCAAGCCAGGACATTAACAAGAATATAGCTTGGTACCAACACAAGCCTGGAAAAGGTCCTAGGCTGCTCATACGTTACACATCAATACTAGAGTCAGGCATCCCATCAAGGTTCAGTGGAAGTGGGTCTGGGAGAGATTATTCCTTCAGCATCAGCAACCTGGAGCCTGAAGATATTGCAACATATTACTGTCTACAGTATGATAATCTTCCACC
IGKV19-93*01_SJL	SJL/J	IGKV	MUSMUS IGKV19-93*01 F	100	GACATCCAGATGACACAGTCTCCATCCTCACTGTCTGCATCTCTGGGAGGCAAAGTCACCATCACTTGCAAGGCAAGCCAAGACATTAACAAGTATATAGCTTGGTACCAACACAAGCCTGGAAAAGGTCCTAGGCTGCTCATACATTACACATCTACATTACAGCCAGGCATCCCATCAAGGTTCAGTGGAAGTGGGTCTGGGAGAGATTATTCCTTCAGCATCAGCAACCTGGAGCCTGAAGATATTGCAACTTATTATTGTCTACAGTATGATAATCTTCTACC
IGKV2-109*01_129	129S1/SVIMJ	IGKV	MUSMUS IGKV2-109*01 F	100	GATATTGTGATGACGCAGGCTGCATTCTCCAATCCAGTCACTCTTGGAACATCAGCTTCCATCTCCTGCAGGTCTAGTAAGAGTCTCCTACATAGTAATGGCATCACTTATTTGTATTGGTATCTGCAGAAGCCAGGCCAGTCTCCTCAGCTCCTGATTTATCAGATGTCCAACCTTGCCTCAGGAGTCCCAGACAGGTTCAGTAGCAGTGGGTCAGGAACTGATTTCACACTGAGAATCAGCAGAGTGGAGGCTGAGGATGTGGGTGTTTATTACTGTGCTCAAAATCTAGAACTTCCTCC
IGKV2-109*01_AJ	A/J	IGKV	MUSMUS IGKV2-109*01 F	100	GATATTGTGATGACGCAGGCTGCATTCTCCAATCCAGTCACTCTTGGAACATCAGCTTCCATCTCCTGCAGGTCTAGTAAGAGTCTCCTACATAGTAATGGCATCACTTATTTGTATTGGTATCTGCAGAAGCCAGGCCAGTCTCCTCAGCTCCTGATTTATCAGATGTCCAACCTTGCCTCAGGAGTCCCAGACAGGTTCAGTAGCAGTGGGTCAGGAACTGATTTCACACTGAGAATCAGCAGAGTGGAGGCTGAGGATGTGGGTGTTTATTACTGTGCTCAAAATCTAGAACTTCCTCC

IGKV2-109*01_AKR	AKR/J	IGKV	MUSMUS IGKV2-109*01 F	100	GATATTGTGATGACGCAGGCTG<br>CATTCTCCAATCCAGTCACTCTT<br>GGAACATCAGCTTCCATCTCCT<br>GCAGGTCTAGTAAGAGTCTCCT<br>ACATAGTAATGGCATCACTTATTT<br>GTATTGGTATCTGCAGAAGCCA<br>GGCCAGTCTCCTCAGCTCCTGA<br>TTTATCAGATGTCCAACCTTGCC<br>TCAGGAGTCCCAGACAGGTTCA<br>GTAGCAGTGGGTCAGGAACTGA<br>TTTCACACTGAGAATCAGCAGA<br>GTGGAGGCTGAGGATGTGGGT<br>GTTTATTACTGTGCTCAAAATCTA<br>GAACTTCCTCC
IGKV2-109*01_B6	C57BL/6J	IGKV	MUSMUS IGKV2-109*01 F	100	GATATTGTGATGACGCAGGCTG<br>CATTCTCCAATCCAGTCACTCTT<br>GGAACATCAGCTTCCATCTCCT<br>GCAGGTCTAGTAAGAGTCTCCT<br>ACATAGTAATGGCATCACTTATTT<br>GTATTGGTATCTGCAGAAGCCA<br>GGCCAGTCTCCTCAGCTCCTGA<br>TTTATCAGATGTCCAACCTTGCC<br>TCAGGAGTCCCAGACAGGTTCA<br>GTAGCAGTGGGTCAGGAACTGA<br>TTTCACACTGAGAATCAGCAGA<br>GTGGAGGCTGAGGATGTGGGT<br>GTTTATTACTGTGCTCAAAATCTA<br>GAACTTCCTCC
IGKV2-109*01_BALB	BALB/CBYJ	IGKV	MUSMUS IGKV2-109*01 F	100	GATATTGTGATGACGCAGGCTG<br>CATTCTCCAATCCAGTCACTCTT<br>GGAACATCAGCTTCCATCTCCT<br>GCAGGTCTAGTAAGAGTCTCCT<br>ACATAGTAATGGCATCACTTATTT<br>GTATTGGTATCTGCAGAAGCCA<br>GGCCAGTCTCCTCAGCTCCTGA<br>TTTATCAGATGTCCAACCTTGCC<br>TCAGGAGTCCCAGACAGGTTCA<br>GTAGCAGTGGGTCAGGAACTGA<br>TTTCACACTGAGAATCAGCAGA<br>GTGGAGGCTGAGGATGTGGGT<br>GTTTATTACTGTGCTCAAAATCTA<br>GAACTTCCTCC
IGKV2-109*01_C3H	C3H/HEJ	IGKV	MUSMUS IGKV2-109*01 F	100	GATATTGTGATGACGCAGGCTG<br>CATTCTCCAATCCAGTCACTCTT<br>GGAACATCAGCTTCCATCTCCT<br>GCAGGTCTAGTAAGAGTCTCCT<br>ACATAGTAATGGCATCACTTATTT<br>GTATTGGTATCTGCAGAAGCCA<br>GGCCAGTCTCCTCAGCTCCTGA<br>TTTATCAGATGTCCAACCTTGCC<br>TCAGGAGTCCCAGACAGGTTCA<br>GTAGCAGTGGGTCAGGAACTGA<br>TTTCACACTGAGAATCAGCAGA<br>GTGGAGGCTGAGGATGTGGGT<br>GTTTATTACTGTGCTCAAAATCTA<br>GAACTTCCTCC

IGKV2-109*01_CBA	CBA/J	IGKV	MUSMUS IGKV2-109*01 F	100	GATATTGTGATGACGCAGGCTGCATTCTCCAATCCAGTCACTCTTGGAACATCAGCTTCCATCTCCTGCAGGTCTAGTAAGAGTCTCCTACATAGTAATGGCATCACTTATTTGTATTGGTATCTGCAGAAGCCAGGCCAGTCTCCTCAGCTCCTGATTTATCAGATGTCCAACCTTGCCTCAGGAGTCCCAGACAGGTTCAGTAGCAGTGGGTCAGGAACTGATTTCACACTGAGAATCAGCAGAGTGGAGGCTGAGGATGTGGGTGTTTATTACTGTGCTCAAAATCTAGAACTTCCTCC
IGKV2-109*01_DBA1	DBA/1J	IGKV	MUSMUS IGKV2-109*01 F	100	GATATTGTGATGACGCAGGCTGCATTCTCCAATCCAGTCACTCTTGGAACATCAGCTTCCATCTCCTGCAGGTCTAGTAAGAGTCTCCTACATAGTAATGGCATCACTTATTTGTATTGGTATCTGCAGAAGCCAGGCCAGTCTCCTCAGCTCCTGATTTATCAGATGTCCAACCTTGCCTCAGGAGTCCCAGACAGGTTCAGTAGCAGTGGGTCAGGAACTGATTTCACACTGAGAATCAGCAGAGTGGAGGCTGAGGATGTGGGTGTTTATTACTGTGCTCAAAATCTAGAACTTCCTCC
IGKV2-109*01_DBA2	DBA/2J	IGKV	MUSMUS IGKV2-109*01 F	100	GATATTGTGATGACGCAGGCTGCATTCTCCAATCCAGTCACTCTTGGAACATCAGCTTCCATCTCCTGCAGGTCTAGTAAGAGTCTCCTACATAGTAATGGCATCACTTATTTGTATTGGTATCTGCAGAAGCCAGGCCAGTCTCCTCAGCTCCTGATTTATCAGATGTCCAACCTTGCCTCAGGAGTCCCAGACAGGTTCAGTAGCAGTGGGTCAGGAACTGATTTCACACTGAGAATCAGCAGAGTGGAGGCTGAGGATGTGGGTGTTTATTACTGTGCTCAAAATCTAGAACTTCCTCC
IGKV2-109*01_MRL	MRL/MPJ	IGKV	MUSMUS IGKV2-109*01 F	100	GATATTGTGATGACGCAGGCTGCATTCTCCAATCCAGTCACTCTTGGAACATCAGCTTCCATCTCCTGCAGGTCTAGTAAGAGTCTCCTACATAGTAATGGCATCACTTATTTGTATTGGTATCTGCAGAAGCCAGGCCAGTCTCCTCAGCTCCTGATTTATCAGATGTCCAACCTTGCCTCAGGAGTCCCAGACAGGTTCAGTAGCAGTGGGTCAGGAACTGATTTCACACTGAGAATCAGCAGAGTGGAGGCTGAGGATGTGGGTGTTTATTACTGTGCTCAAAATCTAGAACTTCCTCC

IGKV2-109*01_SJL	SJL/J	IGKV	MUSMUS IGKV2-109*01 F	100	GATATTGTGATGACGCAGGCTGCATTCTCCAATCCAGTCACTCTTGGAACATCAGCTTCCATCTCCTGCAGGTCTAGTAAGAGTCTCCTACATAGTAATGGCATCACTTATTTGTATTGGTATCTGCAGAAGCCAGGCCAGTCTCCTCAGCTCCTGATTTATCAGATGTCCAACCTTGCCTCAGGAGTCCCAGACAGGTTCAGTAGCAGTGGGTCAGGAACTGATTTCACACTGAGAATCAGCAGAGTGGAGGCTGAGGATGTGGGTGTTTATTACTGTGCTCAAAATCTAGAACTTCCTCC
IGKV2-109*03_NOD	NOD/SHILTJ	IGKV	MUSMUS IGKV2-109*03 F	100	GATATTGTGATGACGCAGGCTGCATTCTCCAATCCAGTCACTCTTGGAACATCAGCTTCCATCTCTTGCAGTTCTAGTAAGAGTCTCCTACATAGTAATGGCATCACTTATTTGTATTGGTATCTGCAGAGGCCAGGCCAGTCTCCTCAGCTCCTGATATATCGGATGTCCAACCTTGCCTCAGGAGTCCCAGACAGGTTCAGTGGCAGTGGGTCAGGAACTGATTTCACACTGAGAATCAGCAGAGTGGAGGCTGAGGATGTGGGTGTTTATTACTGTGCTCAAATGCTAGAACGCCCTCC
IGKV2-109*03_NOR	NOR/LTJ	IGKV	MUSMUS IGKV2-109*03 F	100	GATATTGTGATGACGCAGGCTGCATTCTCCAATCCAGTCACTCTTGGAACATCAGCTTCCATCTCTTGCAGTTCTAGTAAGAGTCTCCTACATAGTAATGGCATCACTTATTTGTATTGGTATCTGCAGAGGCCAGGCCAGTCTCCTCAGCTCCTGATATATCGGATGTCCAACCTTGCCTCAGGAGTCCCAGACAGGTTCAGTGGCAGTGGGTCAGGAACTGATTTCACACTGAGAATCAGCAGAGTGGAGGCTGAGGATGTGGGTGTTTATTACTGTGCTCAAATGCTAGAACGCCCTCC
IGKV2-109*03_NZB	NZB/BLNJ	IGKV	MUSMUS IGKV2-109*03 F	100	GATATTGTGATGACGCAGGCTGCATTCTCCAATCCAGTCACTCTTGGAACATCAGCTTCCATCTCTTGCAGTTCTAGTAAGAGTCTCCTACATAGTAATGGCATCACTTATTTGTATTGGTATCTGCAGAGGCCAGGCCAGTCTCCTCAGCTCCTGATATATCGGATGTCCAACCTTGCCTCAGGAGTCCCAGACAGGTTCAGTGGCAGTGGGTCAGGAACTGATTTCACACTGAGAATCAGCAGAGTGGAGGCTGAGGATGTGGGTGTTTATTACTGTGCTCAAATGCTAGAACGCCCTCC

IGKV2-109*03_S0318_CAST	CAST/EIJ	IGKV	MUSMUS IGKV2-109*03 F	99.32	GATATTGTGATGACACAGGCTGCATTCTCCAATCCAGTCACTCTTGGAACATCAGCTTCCATCTCTTGCAGTTCTAGTAAGAGTCTCCTACATAGTAATGGCATCACTTATTTGTATTGGTATCTGCAGAGGCCAGGCCAGTCTCCTCAGCTCCTGATATATCGGATGTCCAACCTTGCCTCAGGAGTCCCAGACAGGTTCAGTGGCAGAGGGTCAGGAACTGATTTCACACTGAGAATCAGCAGAGTGGAGGCTGAGGATGTGGGTGTTTATTACTGTGCTCAAATGCTAGAACGCCCTCC
IGKV2-109*03_S1521_AJ	A/J	IGKV	MUSMUS IGKV2-116*01 F	100	GATATTGTGATGACGCAGGCTGCCTTCTCCAATCCAGTCACTCTTGGAACATCAGCTTCCATCTCCTGCAGGTCTAGTAAGAATCTCCTACATAGTAATGGCATCACTTATTTGTATTGGTATCTGCAGAGGCCAGGCCAGTCTCCTCAGCTCCTGATATATCGGGTGTCCAATCTGGCCTCAGGAGTCCCAAACAGGTTCAGTGGCAGTGAGTCAGGAACTGATTTCACACTGAGAATCAGCAGAGTGGAGGCTGAGGATGTGGGTGTTTATTACTGTGCTCAACTGCTAGAACTCCCTC
IGKV2-109*03_S3211_LEWES	LEWES/EIJ	IGKV	MUSMUS IGKV2-109*03 F, OR MUSMUS IGKV2-109*04 F	97.62	GATATTGTGATGACACAGGCTGCATTCTCCAATCCAGTCACTCTTGGAACATCAGCTTCCATCTCCTGCAGGTCTAGTAAGAGTCTCCTAAATAGTAATGGCATCACTTCTTTGTATTGGTATCTGCAGAGGCCAGGCCAGTCTCCTCAGCTCCTGATATATCGGATGTCCAACCTTGCCTCAGGAGTCCCAGACAGGTTCAGTAGCAGTGGGTCAGGAACTGATTTCACACTGAGAATCAGCAGAGTGGAGGCTGAGGATGTGGGTGTTTATTACTGTGCTCAAAGGCTAGAACTCCCTC
IGKV2-109*03_S4111_CAST	CAST/EIJ	IGKV	MUSMUS IGKV2-109*03 F, OR MUSMUS IGKV2-109*04 F	97.96	GATATTGTGATGACACAGGCTGCATTCTCCAATCCAGTCACTCTTGGAACATCAGCTTCCATCTCCTGCAGGTCTAGTAAGAGTCTCCTACATAGTAATGGCATCACTTATTTGTATTGGTATCTGCAGAGGCCAGGCCAGTCTCCTCAGCTCCTGATACATCGGATGTCCAACCTTGCCTCAGGAGTCCCAGACAGGTTCAGTAGCAGTGGGTCAGGAACTGATTTCACACTGAGAATCAGCAGAGTGGAGGCTGAGGATGTGGGTGTTTATTACTGTGCTCAAAGGCTAGAACTCCCTC

IGKV2-109*03_S4495_LEWES	LEWES/EIJ	IGKV	MUSMUS IGKV2-109*03 F, OR MUSMUS IGKV2-109*04 F	98.98	GATATTGTGATGACGCAGGCTGCATTCTCCAATCCAGTCACTCTTGGAACATCAGCTTCCATCTCCTGCAGGTCTAGTAAGAGTCTCCTACATAGTAATGGCATCACTTATTTGTATTGGTATCTGCAGAGGCCAGGCCAGTCTCCTCAGCTCCTGATATATCGGATGTCCAACCTTGCCTCAGGAGTCCCAGACAGGTTCAGTAGCAGTGGGTCAGGAACTGATTTCACACTGAGAATCAGCAGAGTGGAGGCTGAGGATGTGGGTGTTTATTACTGTGCTCAAATGCTAGAACTCCCTC
IGKV2-109*03_S5201_PWD	PWD/PHJ	IGKV	MUSMUS IGKV2-109*03 F, OR MUSMUS IGKV2-109*04 F	98.3	GATATTGTGATGACACAGGCTGCATTCTCCAATCCAGTCACTCTTGGAACATCAGCTTCCATCTCCTGCAGGTCTAGTAAGAGTCTCCTACATAGTAATGGCGTCACTTATTTGTATTGGTATCTGCAGAGGCCAGGCCAGTCTCCTCAGCTCCTGATATATCGGATGTCCAACCTTGCCTCAGGAGTCCCAGACAGGTTCAGTAGCAGTGGGTCAGGAACTGATTTCACACTGAGAATCAGCAGAGTGGAGGCTGAGGATGTGGGTGTTTATTACTGTGCTCAAATGCTAGAACTCCCTCC
IGKV2-109*04_NOD	NOD/SHILTJ	IGKV	MUSMUS IGKV2-109*04 F	100	GATATTGTGATGACGCAGGCTGCATTCTCCAATCCAGTCACTCTTGGAACGTCAGCTTCCATCTCCTGCAGGTCTAGTAAGAGTCTCCTACATAGTGATGGCATCACTTATTTGTATTGGTATCTGCAGAGGCCAGGCCAGTCTCCTCAGCTCCTGATATATCGGATGTCCAACCTTGCCTCAGGAGTCCCAGACAGGTTCAGTGGCAGTGGGTCAGGAACTGATTTCACACTGAGAATCAGCAGAGTGGAGGCTGAGGATGTGGGTGTTTATTACTGTGCTCAAATGCTAGAATTCCCTCC
IGKV2-109*04_NOR	NOR/LTJ	IGKV	MUSMUS IGKV2-109*04 F	100	GATATTGTGATGACGCAGGCTGCATTCTCCAATCCAGTCACTCTTGGAACGTCAGCTTCCATCTCCTGCAGGTCTAGTAAGAGTCTCCTACATAGTGATGGCATCACTTATTTGTATTGGTATCTGCAGAGGCCAGGCCAGTCTCCTCAGCTCCTGATATATCGGATGTCCAACCTTGCCTCAGGAGTCCCAGACAGGTTCAGTGGCAGTGGGTCAGGAACTGATTTCACACTGAGAATCAGCAGAGTGGAGGCTGAGGATGTGGGTGTTTATTACTGTGCTCAAATGCTAGAATTCCCTCC

IGKV2-109*04_NZB	NZB/BLNJ	IGKV	MUSMUS IGKV2-109*04 F	100	GATATTGTGATGACGCAGGCTG<br>CATTCTCCAATCCAGTCACTCTT<br>GGAACGTCAGCTTCCATCTCCT<br>GCAGGTCTAGTAAGAGTCTCCT<br>ACATAGTGATGGCATCACTTATT<br>TGTATTGGTATCTGCAGAGGCC<br>AGGCCAGTCTCCTCAGCTCCTG<br>ATATATCGGATGTCCAACCTTGC<br>CTCAGGAGTCCCAGACAGGTTC<br>AGTGGCAGTGGGTCAGGAACT<br>GATTTCACACTGAGAATCAGCA<br>GAGTGGAGGCTGAGGATGTGG<br>GTGTTTATTACTGTGCTCAAATG<br>CTAGAATTCCCTCC
IGKV2-112*01_129	129S1/SVIMJ	IGKV	MUSMUS IGKV2-112*01 F	100	GATATTGTGATAACCCAGGATGA<br>ACTCTCCAATCCTGTCACTTCTG<br>GAGAATCAGTTTCCATCTCCTGC<br>AGGTCTAGTAAGAGTCTCCTATA<br>TAAGGATGGGAAGACATACTTG<br>AATTGGTTTCTGCAGAGACCAG<br>GACAATCTCCTCAGCTCCTGAT<br>CTATTTGATGTCCACCCGTGCAT<br>CAGGAGTCTCAGACCGGTTTAG<br>TGGCAGTGGGTCAGGAACAGAT<br>TTCACCCTGGAAATCAGTAGAGT<br>GAAGGCTGAGGATGTGGGTGTG<br>TATTACTGTCAACAACTTGTAGA<br>GTATCCT
IGKV2-112*01_AJ	A/J	IGKV	MUSMUS IGKV2-112*01 F	100	GATATTGTGATAACCCAGGATGA<br>ACTCTCCAATCCTGTCACTTCTG<br>GAGAATCAGTTTCCATCTCCTGC<br>AGGTCTAGTAAGAGTCTCCTATA<br>TAAGGATGGGAAGACATACTTG<br>AATTGGTTTCTGCAGAGACCAG<br>GACAATCTCCTCAGCTCCTGAT<br>CTATTTGATGTCCACCCGTGCAT<br>CAGGAGTCTCAGACCGGTTTAG<br>TGGCAGTGGGTCAGGAACAGAT<br>TTCACCCTGGAAATCAGTAGAGT<br>GAAGGCTGAGGATGTGGGTGTG<br>TATTACTGTCAACAACTTGTAGA<br>GTATCCT
IGKV2-112*01_AKR	AKR/J	IGKV	MUSMUS IGKV2-112*01 F	100	GATATTGTGATAACCCAGGATGA<br>ACTCTCCAATCCTGTCACTTCTG<br>GAGAATCAGTTTCCATCTCCTGC<br>AGGTCTAGTAAGAGTCTCCTATA<br>TAAGGATGGGAAGACATACTTG<br>AATTGGTTTCTGCAGAGACCAG<br>GACAATCTCCTCAGCTCCTGAT<br>CTATTTGATGTCCACCCGTGCAT<br>CAGGAGTCTCAGACCGGTTTAG<br>TGGCAGTGGGTCAGGAACAGAT<br>TTCACCCTGGAAATCAGTAGAGT<br>GAAGGCTGAGGATGTGGGTGTG<br>TATTACTGTCAACAACTTGTAGA<br>GTATCCT

IGKV2-112*01_B6	C57BL/6J	IGKV	MUSMUS IGKV2-112*01 F	100	GATATTGTGATAACCCAGGATGAACTCTCCAATCCTGTCACTTCTGGAGAATCAGTTTCCATCTCCTGCAGGTCTAGTAAGAGTCTCCTATATAAGGATGGGAAGACATACTTGAATTGGTTTCTGCAGAGACCAGGACAATCTCCTCAGCTCCTGATCTATTTGATGTCCACCCGTGCATCAGGAGTCTCAGACCGGTTTAGTGGCAGTGGGTCAGGAACAGATTTCACCCTGGAAATCAGTAGAGTGAAGGCTGAGGATGTGGGTGTGTATTACTGTCAACAACTTGTAGAGTATCCT
IGKV2-112*01_BALB	BALB/CBYJ	IGKV	MUSMUS IGKV2-112*01 F	100	GATATTGTGATAACCCAGGATGAACTCTCCAATCCTGTCACTTCTGGAGAATCAGTTTCCATCTCCTGCAGGTCTAGTAAGAGTCTCCTATATAAGGATGGGAAGACATACTTGAATTGGTTTCTGCAGAGACCAGGACAATCTCCTCAGCTCCTGATCTATTTGATGTCCACCCGTGCATCAGGAGTCTCAGACCGGTTTAGTGGCAGTGGGTCAGGAACAGATTTCACCCTGGAAATCAGTAGAGTGAAGGCTGAGGATGTGGGTGTGTATTACTGTCAACAACTTGTAGAGTATCCT
IGKV2-112*01_C3H	C3H/HEJ	IGKV	MUSMUS IGKV2-112*01 F	100	GATATTGTGATAACCCAGGATGAACTCTCCAATCCTGTCACTTCTGGAGAATCAGTTTCCATCTCCTGCAGGTCTAGTAAGAGTCTCCTATATAAGGATGGGAAGACATACTTGAATTGGTTTCTGCAGAGACCAGGACAATCTCCTCAGCTCCTGATCTATTTGATGTCCACCCGTGCATCAGGAGTCTCAGACCGGTTTAGTGGCAGTGGGTCAGGAACAGATTTCACCCTGGAAATCAGTAGAGTGAAGGCTGAGGATGTGGGTGTGTATTACTGTCAACAACTTGTAGAGTATCCT
IGKV2-112*01_CBA	CBA/J	IGKV	MUSMUS IGKV2-112*01 F	100	GATATTGTGATAACCCAGGATGAACTCTCCAATCCTGTCACTTCTGGAGAATCAGTTTCCATCTCCTGCAGGTCTAGTAAGAGTCTCCTATATAAGGATGGGAAGACATACTTGAATTGGTTTCTGCAGAGACCAGGACAATCTCCTCAGCTCCTGATCTATTTGATGTCCACCCGTGCATCAGGAGTCTCAGACCGGTTTAGTGGCAGTGGGTCAGGAACAGATTTCACCCTGGAAATCAGTAGAGTGAAGGCTGAGGATGTGGGTGTGTATTACTGTCAACAACTTGTAGAGTATCCT

IGKV2-112*01_DBA1	DBA/1J	IGKV	MUSMUS IGKV2-112*01 F	100	GATATTGTGATAACCCAGGATGAACTCTCCAATCCTGTCACTTCTGGAGAATCAGTTTCCATCTCCTGCAGGTCTAGTAAGAGTCTCCTATATAAGGATGGGAAGACATACTTGAATTGGTTTCTGCAGAGACCAGGACAATCTCCTCAGCTCCTGATCTATTTGATGTCCACCCGTGCATCAGGAGTCTCAGACCGGTTTAGTGGCAGTGGGTCAGGAACAGATTTCACCCTGGAAATCAGTAGAGTGAAGGCTGAGGATGTGGGTGTGTATTACTGTCAACAACTTGTAGAGTATCCT
IGKV2-112*01_DBA2	DBA/2J	IGKV	MUSMUS IGKV2-112*01 F	100	GATATTGTGATAACCCAGGATGAACTCTCCAATCCTGTCACTTCTGGAGAATCAGTTTCCATCTCCTGCAGGTCTAGTAAGAGTCTCCTATATAAGGATGGGAAGACATACTTGAATTGGTTTCTGCAGAGACCAGGACAATCTCCTCAGCTCCTGATCTATTTGATGTCCACCCGTGCATCAGGAGTCTCAGACCGGTTTAGTGGCAGTGGGTCAGGAACAGATTTCACCCTGGAAATCAGTAGAGTGAAGGCTGAGGATGTGGGTGTGTATTACTGTCAACAACTTGTAGAGTATCCT
IGKV2-112*01_MRL	MRL/MPJ	IGKV	MUSMUS IGKV2-112*01 F	100	GATATTGTGATAACCCAGGATGAACTCTCCAATCCTGTCACTTCTGGAGAATCAGTTTCCATCTCCTGCAGGTCTAGTAAGAGTCTCCTATATAAGGATGGGAAGACATACTTGAATTGGTTTCTGCAGAGACCAGGACAATCTCCTCAGCTCCTGATCTATTTGATGTCCACCCGTGCATCAGGAGTCTCAGACCGGTTTAGTGGCAGTGGGTCAGGAACAGATTTCACCCTGGAAATCAGTAGAGTGAAGGCTGAGGATGTGGGTGTGTATTACTGTCAACAACTTGTAGAGTATCCT
IGKV2-112*01_SJL	SJL/J	IGKV	MUSMUS IGKV2-112*01 F	100	GATATTGTGATAACCCAGGATGAACTCTCCAATCCTGTCACTTCTGGAGAATCAGTTTCCATCTCCTGCAGGTCTAGTAAGAGTCTCCTATATAAGGATGGGAAGACATACTTGAATTGGTTTCTGCAGAGACCAGGACAATCTCCTCAGCTCCTGATCTATTTGATGTCCACCCGTGCATCAGGAGTCTCAGACCGGTTTAGTGGCAGTGGGTCAGGAACAGATTTCACCCTGGAAATCAGTAGAGTGAAGGCTGAGGATGTGGGTGTGTATTACTGTCAACAACTTGTAGAGTATCCT

IGKV2-112*02_NOR	NOR/LTJ	IGKV	MUSMUS IGKV2-112*02 F	100	GATATTGTGATAACCCAGGATGAACTCTCCAATCCTGTCACTTCTGGAGAATCAGTTTCCATCTCCTGCAGGTCTAGTAAGAGTCTCCTATATAAGGATGGGAAGACATACTTGAATTGGTTTCTGCAGAGGCCAGGACAGTCTCCTCAGCTCCTGGTCTATTGGATGTCCACCCGTGCATCAGGAGTCTCAGACCGGTTTAGTGGCAGTGGGTCAGGAACAGATTTCACACTGGAAATCAGTAGAGTGAAGGCTGAGGATGTCGGTGTGTATTACTGTCAACAAGTTGTAGAGTATCCTC
IGKV2-112*02_NZB	NZB/BLNJ	IGKV	MUSMUS IGKV2-112*02 F	100	GATATTGTGATAACCCAGGATGAACTCTCCAATCCTGTCACTTCTGGAGAATCAGTTTCCATCTCCTGCAGGTCTAGTAAGAGTCTCCTATATAAGGATGGGAAGACATACTTGAATTGGTTTCTGCAGAGGCCAGGACAGTCTCCTCAGCTCCTGGTCTATTGGATGTCCACCCGTGCATCAGGAGTCTCAGACCGGTTTAGTGGCAGTGGGTCAGGAACAGATTTCACACTGGAAATCAGTAGAGTGAAGGCTGAGGATGTCGGTGTGTATTACTGTCAACAAGTTGTAGAGTATCCTC
IGKV2-112*02_S2310_CAST	CAST/EIJ	IGKV	MUSMUS IGKV2-112*02 F	99.66	GATATTGTGATAACCCAGGATGAACTCTCCAATCCTGTCACTTCTGGAGAATCAGTTTCCATCTCCTGCAGGTCTAGTAAGAGTCTCCTATATAAGGATGGGAAGACATACTTGAATTGGTTTCTGCAGAGGCCAGGACAGTCTCCTCAGCTCCTGGTCTATTGGATGTCCACCCGTGCATCAGGAGTCTCAGACCGGTTTAGTGGCAGTGGGTCAGGAACAGTTTTCACACTGGAAATCAGTAGAGTGAAGGCTGAGGATGTCGGTGTGTATTACTGTCAACAAGTTGTAGAGTATCCTC
IGKV2-112*02_S7480_LEWES	LEWES/EIJ	IGKV	MUSMUS IGKV2-112*02 F	98.3	GATATTGTGATAACCCAGGATGAACTCTCCAATCCTGTCACTTCTGGAGAATCGGTTTCCATCTCCTGCAGGTCTAGTAAGAGTCTCCTATATACAGATGGGAAGACATACTTGAATTGGTTTCTGCAGAGGCCAGGACAGTCTCCTCAGCTCCTGGTCTATTGGATGTCCACTCGTGCATCAGGAGTCTCAGACCGGTTTAGTGGCAGTGGGTCAGGAACAGATTTCACACTGGAAATCAGTAGAGTGAAGGCTGAGGATGTGGGTGTGTATTACTGTCAACAAGTTGTAGAGTATCCTC

IGKV2-137*01_129	129S1/SVIMJ	IGKV	MUSMUS IGKV2-137*01 F	100	GATATTGTGATGACTCAGGCTGCACCCTCTGTACCTGTCACTCCTGGAGAGTCAGTATCCATCTCCTGCAGGTCTAGTAAGAGTCTCCTGCATAGTAATGGCAACACTTACTTGTATTGGTTCCTGCAGAGGCCAGGCCAGTCTCCTCAGCTCCTGATATATCGGATGTCCAACCTTGCCTCAGGAGTCCCAGACAGGTTCAGTGGCAGTGGGTCAGGAACTGCTTTCACACTGAGAATCAGTAGAGTGGAGGCTGAGGATGTGGGTGTTTATTACTGTATGCAACATCTAGAATATCCTTT
IGKV2-137*01_AJ	A/J	IGKV	MUSMUS IGKV2-137*01 F	100	GATATTGTGATGACTCAGGCTGCACCCTCTGTACCTGTCACTCCTGGAGAGTCAGTATCCATCTCCTGCAGGTCTAGTAAGAGTCTCCTGCATAGTAATGGCAACACTTACTTGTATTGGTTCCTGCAGAGGCCAGGCCAGTCTCCTCAGCTCCTGATATATCGGATGTCCAACCTTGCCTCAGGAGTCCCAGACAGGTTCAGTGGCAGTGGGTCAGGAACTGCTTTCACACTGAGAATCAGTAGAGTGGAGGCTGAGGATGTGGGTGTTTATTACTGTATGCAACATCTAGAATATCCTTT
IGKV2-137*01_AKR	AKR/J	IGKV	MUSMUS IGKV2-137*01 F	100	GATATTGTGATGACTCAGGCTGCACCCTCTGTACCTGTCACTCCTGGAGAGTCAGTATCCATCTCCTGCAGGTCTAGTAAGAGTCTCCTGCATAGTAATGGCAACACTTACTTGTATTGGTTCCTGCAGAGGCCAGGCCAGTCTCCTCAGCTCCTGATATATCGGATGTCCAACCTTGCCTCAGGAGTCCCAGACAGGTTCAGTGGCAGTGGGTCAGGAACTGCTTTCACACTGAGAATCAGTAGAGTGGAGGCTGAGGATGTGGGTGTTTATTACTGTATGCAACATCTAGAATATCCTTT
IGKV2-137*01_B6	C57BL/6J	IGKV	MUSMUS IGKV2-137*01 F	100	GATATTGTGATGACTCAGGCTGCACCCTCTGTACCTGTCACTCCTGGAGAGTCAGTATCCATCTCCTGCAGGTCTAGTAAGAGTCTCCTGCATAGTAATGGCAACACTTACTTGTATTGGTTCCTGCAGAGGCCAGGCCAGTCTCCTCAGCTCCTGATATATCGGATGTCCAACCTTGCCTCAGGAGTCCCAGACAGGTTCAGTGGCAGTGGGTCAGGAACTGCTTTCACACTGAGAATCAGTAGAGTGGAGGCTGAGGATGTGGGTGTTTATTACTGTATGCAACATCTAGAATATCCTTT

IGKV2-137*01_BALB	BALB/CBY J	IGKV	MUSMUS IGKV2-137*01 F	100	GATATTGTGATGACTCAGGCTG CACCCTCTGTACCTGTCACTCC TGGAGAGTCAGTATCCATCTCCT GCAGGTCTAGTAAGAGTCTCCT GCATAGTAATGGCAACACTTACT TGTATTGGTTCCTGCAGAGGCC AGGCCAGTCTCCTCAGCTCCTG ATATATCGGATGTCCAACCTTGC CTCAGGAGTCCCAGACAGGTTC AGTGGCAGTGGGTCAGGAACT GCTTTCACACTGAGAATCAGTAG AGTGGAGGCTGAGGATGTGGGT GTTTATTACTGTATGCAACATCTA GAATATCCTTT
IGKV2-137*01_C3H	C3H/HEJ	IGKV	MUSMUS IGKV2-137*01 F	100	GATATTGTGATGACTCAGGCTG CACCCTCTGTACCTGTCACTCC TGGAGAGTCAGTATCCATCTCCT GCAGGTCTAGTAAGAGTCTCCT GCATAGTAATGGCAACACTTACT TGTATTGGTTCCTGCAGAGGCC AGGCCAGTCTCCTCAGCTCCTG ATATATCGGATGTCCAACCTTGC CTCAGGAGTCCCAGACAGGTTC AGTGGCAGTGGGTCAGGAACT GCTTTCACACTGAGAATCAGTAG AGTGGAGGCTGAGGATGTGGGT GTTTATTACTGTATGCAACATCTA GAATATCCTTT
IGKV2-137*01_CBA	CBA/J	IGKV	MUSMUS IGKV2-137*01 F	100	GATATTGTGATGACTCAGGCTG CACCCTCTGTACCTGTCACTCC TGGAGAGTCAGTATCCATCTCCT GCAGGTCTAGTAAGAGTCTCCT GCATAGTAATGGCAACACTTACT TGTATTGGTTCCTGCAGAGGCC AGGCCAGTCTCCTCAGCTCCTG ATATATCGGATGTCCAACCTTGC CTCAGGAGTCCCAGACAGGTTC AGTGGCAGTGGGTCAGGAACT GCTTTCACACTGAGAATCAGTAG AGTGGAGGCTGAGGATGTGGGT GTTTATTACTGTATGCAACATCTA GAATATCCTTT
IGKV2-137*01_DBA1	DBA/1J	IGKV	MUSMUS IGKV2-137*01 F	100	GATATTGTGATGACTCAGGCTG CACCCTCTGTACCTGTCACTCC TGGAGAGTCAGTATCCATCTCCT GCAGGTCTAGTAAGAGTCTCCT GCATAGTAATGGCAACACTTACT TGTATTGGTTCCTGCAGAGGCC AGGCCAGTCTCCTCAGCTCCTG ATATATCGGATGTCCAACCTTGC CTCAGGAGTCCCAGACAGGTTC AGTGGCAGTGGGTCAGGAACT GCTTTCACACTGAGAATCAGTAG AGTGGAGGCTGAGGATGTGGGT GTTTATTACTGTATGCAACATCTA GAATATCCTTT

IGKV2-137*01_DBA2	DBA/2J	IGKV	MUSMUS IGKV2-137*01 F	100	GATATTGTGATGACTCAGGCTGCACCCTCTGTACCTGTCACTCCTGGAGAGTCAGTATCCATCTCCTGCAGGTCTAGTAAGAGTCTCCTGCATAGTAATGGCAACACTTACTTGTATTGGTTCCTGCAGAGGCCAGGCCAGTCTCCTCAGCTCCTGATATATCGGATGTCCAACCTTGCCTCAGGAGTCCCAGACAGGTTCAGTGGCAGTGGGTCAGGAACTGCTTTCACACTGAGAATCAGTAGAGTGGAGGCTGAGGATGTGGGTGTTTATTACTGTATGCAACATCTAGAATATCCTTT
IGKV2-137*01_MRL	MRL/MPJ	IGKV	MUSMUS IGKV2-137*01 F	100	GATATTGTGATGACTCAGGCTGCACCCTCTGTACCTGTCACTCCTGGAGAGTCAGTATCCATCTCCTGCAGGTCTAGTAAGAGTCTCCTGCATAGTAATGGCAACACTTACTTGTATTGGTTCCTGCAGAGGCCAGGCCAGTCTCCTCAGCTCCTGATATATCGGATGTCCAACCTTGCCTCAGGAGTCCCAGACAGGTTCAGTGGCAGTGGGTCAGGAACTGCTTTCACACTGAGAATCAGTAGAGTGGAGGCTGAGGATGTGGGTGTTTATTACTGTATGCAACATCTAGAATATCCTTT
IGKV2-137*01_S0442_NOD	NOD/SHILTJ	IGKV	MUSMUS IGKV2-137*01 F	93.54	GATATTGTGATGACTCAGGCTGCACCCTCTGTACCTGTCACTCCTGGAGAGTCAGTATCCATCTCCTGCAGGTCTAGTACGAGTCTCCTGCACAGTAGTGGCAAGCATAGGTTGTATTGGTTCCTACAGAGGCCAGGCCAGTCTCCTCAGCTCCTGATATATTATATGTCCAACCTTGCCTCAGGAGTCCCAGACAGGTTCAGTGGCAGTGGGTCAGGAACTGATTTCACACTGAGAATCAGTAGAGTGGAGGCTGAGGATTTTGGTGTTTATTATTGTATGCAAAGTCTAGAATATCCTTT
IGKV2-137*01_S0442_NOR	NOR/LTJ	IGKV	MUSMUS IGKV2-137*01 F	93.54	GATATTGTGATGACTCAGGCTGCACCCTCTGTACCTGTCACTCCTGGAGAGTCAGTATCCATCTCCTGCAGGTCTAGTACGAGTCTCCTGCACAGTAGTGGCAAGCATAGGTTGTATTGGTTCCTACAGAGGCCAGGCCAGTCTCCTCAGCTCCTGATATATTATATGTCCAACCTTGCCTCAGGAGTCCCAGACAGGTTCAGTGGCAGTGGGTCAGGAACTGATTTCACACTGAGAATCAGTAGAGTGGAGGCTGAGGATTTTGGTGTTTATTATTGTATGCAAAGTCTAGAATATCCTTT

IGKV2-137*01_S0442_NZB	NZB/BLNJ	IGKV	MUSMUS IGKV2-137*01 F	93.54	GATATTGTGATGACTCAGGCTG<br>CACCCTCTGTACCTGTCACTCC<br>TGGAGAGTCAGTATCCATCTCCT<br>GCAGGTCTAGTACGAGTCTCCT<br>GCACAGTAGTGGCAAGCATAGG<br>TTGTATTGGTTCCTACAGAGGCC<br>AGGCCAGTCTCCTCAGCTCCTG<br>ATATATTATATGTCCAACCTTGC<br>CTCAGGAGTCCCAGACAGGTTC<br>AGTGGCAGTGGGTCAGGAACT<br>GATTTCACACTGAGAATCAGTAG<br>AGTGGAGGCTGAGGATTTTGGT<br>GTTTATTATTGTATGCAAAGTCTA<br>GAATATCCTTT
IGKV2-137*01_S0906_MSM	MSM/MSJ	IGKV	MUSMUS IGKV2-137*01 F	94.22	GATATTGTGATGACTCAGGCTG<br>CACCCTCTGTACCTGTCACTCC<br>TGGAGAGTCAGTATCCATCTCCT<br>GCAGATCTAGTAAGAGTCTCCT<br>GCACAGTAGTGGCAAGCATAGG<br>TTGTATTGGTTCCTACAGAGGCC<br>AGGCCAGTCTCCTCAGCGCCT<br>GATATATTATATGTCCAACCTTG<br>CCTCAGGAGTCCCAGACAGGTT<br>CAGTGGCAGTGGGTCAGGAAC<br>TGATTTCACACTGAGAATCAGTA<br>GAGTGGAGGCTGAGGATGTGG<br>GTGTTTATTACTGTATGCAAAGTC<br>TAGAATATCCTTT
IGKV2-137*01_S2608_CAST	CAST/EIJ	IGKV	MUSMUS IGKV2-137*01 F	94.22	GATATTGTGATGACTCAGGCTG<br>CACCCTCTGTACCTGTCACTCC<br>TGGAGAGTCAGTATCCATCTCCT<br>GCAGGTCTAGTAAGAGTCTCCT<br>GCACAGTAGTGGCAAGCATAGG<br>TTGTATTGGTTCCTACAGAGGCC<br>AGGCCAATCTCCTCAGCGCCT<br>GATATATTATATGTCCAACCTTG<br>CCTCAGGAGTCCCAGACAGGTT<br>CAGTGGCAGTGGGTCAGGAAC<br>TGATTTCACACTGAGAATCAGTA<br>GAGTGGAGGCTGAGGATGTGG<br>GTGTTTATTACTGTATGCAAAGTC<br>TAGAATATCCTTT
IGKV2-137*01_S4012_LEWES	LEWES/EIJ	IGKV	MUSMUS IGKV2-137*01 F	96.94	GATATTGTGATGACTCAGGCTG<br>CACCCTCTGTACCTGTCACTCC<br>TGGAGAGTCAGTATCCATCTCCT<br>GCAGGTCTAGTAAGAGTCTTCT<br>GCATAGTAATGGCAACACTTACT<br>TGTATTGGTTCCTGCAGAGGCC<br>AGGCCAGTCTCCTCAGCGCCT<br>GATATATTATATGTCCAACCTTG<br>CCTCAGGAGTCCCAGACAGGTT<br>CAGTGGCAGAGGGTCAGGAAC<br>TGATTTCACACTGAGAATCAGTA<br>GAGTGGAGGCTGAGGATGTGG<br>GTGTTTATTACTGTATGCAAAGTC<br>TAGAATATCCTTT

IGKV2-137*01_S4057_MSM	MSM/MSJ	IGKV	MUSMUS IGKV2-137*01 F	94.22	GATATTGTGATGACTCAGGCTGCACCCTCTGTACCTGTCACTCCTGGAGAGTCAGTATCCATCTCCTGCAGATCTAGTAAGAGTCTCCTGCACAGTAGTGGCAAGCATAGGTTGTATTGGTTCCTACAGAGGCCAGGCCAGTCTCCTCAGCGCCTGATATATTATATGTCCAACCTTGCCTCAGGAGTCCCAGACAGGTTCAGTGGCAGTGGGTCAGGAACTGATTTCACACTGAGAATCAGTAGAGTGGAGGCTGAGGATGTGGGTGTTTATTACTGTATGCAAAGTCTAGAATATCCATT
IGKV2-137*01_S5808_CAST	CAST/EIJ	IGKV	MUSMUS IGKV2-137*01 F	94.56	GATATTGTGATGACTCAGGCTGCACCCTCTGTACCTGTCACTACTGGAGAGTCAGTATCCATTTCTTGCAAGTCTAGTAAGAGTCTTCTGCATAGTAATGGCATCACTTATTTGTATTGGTTCCTACAGAGGCCAGGCCAGTCTCCTCAGCGCCTGATATATTATATGTCCAACCTTGCCTCAGGAGTCCCAGACAGGTTCAGTGGCAGAGGGTCAGGAACTGATTTCACACTGAGAATCAGTAGAGTGGAGGCTGAGGATGTGGGTGTTTATTACTGTATGCAAAGTCTAGAATATCCTTT
IGKV2-137*01_S6567_NOD	NOD/SHILTJ	IGKV	MUSMUS IGKV2-137*01 F	98.64	GATATTGTGATGACTCAGGCTGCACCCTCTGTACCTGTCACTCCTGGAGAGTCAGTATCCATCTCCTGCAGGTCTAGTAAGAGTCTCCTGCATAGTAATGGCAACACTTACTTGTATTGGTTCCTACAGAGGCCTGGCCAGTCTCCTCAGCTCCTGATATATCGGATGTCCAACCTTGCCTCAGGAGTCCCAGACAGGTTCAGTGGCAGTGGGTCAGGAACTGCTTTCACACTGAGAATCAGTAGAGTCGAGGCTGAGGATGTGGGTGTTTATTACTGTATGCAACATCTAGAGTATCCTTT
IGKV2-137*01_S6567_NOR	NOR/LTJ	IGKV	MUSMUS IGKV2-137*01 F	98.64	GATATTGTGATGACTCAGGCTGCACCCTCTGTACCTGTCACTCCTGGAGAGTCAGTATCCATCTCCTGCAGGTCTAGTAAGAGTCTCCTGCATAGTAATGGCAACACTTACTTGTATTGGTTCCTACAGAGGCCTGGCCAGTCTCCTCAGCTCCTGATATATCGGATGTCCAACCTTGCCTCAGGAGTCCCAGACAGGTTCAGTGGCAGTGGGTCAGGAACTGCTTTCACACTGAGAATCAGTAGAGTCGAGGCTGAGGATGTGGGTGTTTATTACTGTATGCAACATCTAGAGTATCCTTT

IGKV2-137*01_S7910_MSM	MSM/MSJ	IGKV	MUSMUS IGKV2-137*01 F	96.6	GATATTGTGATGACTCAGGCTGCACCCTCTGTACCTGTCACTCCTGGAGAGTCAGTATCCATCTCCTGCAGGTCTAGTAAGAGTCTCCTGCATAGTAATGGCAACCATTTGTTGTATTGGTTCCTGCAGAGGCCAGGCCAGTCTCCTCAGCTCCTGATATATTATATGTCCAACCTTGCCTCAGGAGTCCCAGACAGGTTCAGTGGCAGTGGGTCAGGAACTGATTTCACACTGAGAATCAGTAGAGTGGAGGCTGAGGATGTGGGTGTTTATTACTGTATGCAAAGTCTAGAATATCCT
IGKV2-137*01_S9859_PWD	PWD/PHJ	IGKV	MUSMUS IGKV2-137*01 F	94.56	GATATTGTGATGACTCAGGCTGCACCCTCTGTACCTGTCACTCCTGGAGAGTCAGTATCCATCTCCTGCAGGTCTAGTAAGAGTCTCCTGCACAGTAGTGGCAAGCATAGGTTGTATTGGTTCCTACAGAGGCCAGGCCAGTCTCCTCAGCGCCTGATATATTATATGTCCAACCTTGCCTCAGGAGTCCCAGACAGGTTCAGTGGCAGTGGGTCAGGAACTGATTTCACACTGAGAATCAGTAGAGTGGAGGCTGAGGATGTGGGTGTTTATTACTGTATGCAAAGTCTAGAATATCCATT
IGKV2-137*01_SJL	SJL/J	IGKV	MUSMUS IGKV2-137*01 F	100	GATATTGTGATGACTCAGGCTGCACCCTCTGTACCTGTCACTCCTGGAGAGTCAGTATCCATCTCCTGCAGGTCTAGTAAGAGTCTCCTGCATAGTAATGGCAACACTTACTTGTATTGGTTCCTGCAGAGGCCAGGCCAGTCTCCTCAGCTCCTGATATATCGGATGTCCAACCTTGCCTCAGGAGTCCCAGACAGGTTCAGTGGCAGTGGGTCAGGAACTGCTTTCACACTGAGAATCAGTAGAGTGGAGGCTGAGGATGTGGGTGTTTATTACTGTATGCAACATCTAGAATATCCTTT
IGKV20-101-2*01_S0678_CAST	CAST/EIJ	IGKV	MUSMUS IGKV20-101-2*01 F	98.19	AATATCCAGGTGATCCAGTCACCATTTCTGTCTGCATCTGTGGGAGAGAGGGTCACAATCAGCTGCAAGACACATCAGCATATTAACAGTTCCATAGCCTGGTACCAGCAAAAAGTTGGAAAAGCTCCCAAACTCCTGATAAGAGATGCAAGTTTTTCTCTAACAGACACCCCATCAAGGTTCACTGGGAATGGATTTGGCACAGATTTCACACTCAGCATCAGCAGTATGCAGCCTGAAGATGGTGCCACATACTTCTGCCAGCAGCATTTTAACTATTA

IGKV20-101-2*01_S8388_MSM	MSM/MSJ	IGKV	MUSMUS IGKV20-101-2*01 F	97.46	AATATCCAGGTGATCCAGTCACCATTTCTGTCTGCATCTGTGGGAGAGAGGGTCACAATCAGCTGCAAGACACATCAGCATATTAACAGTTCCATAGCCTGGTACCAGCAAAAATTTGGAAAAGCTCCCAAACTCCTGATAAGAGATGGAAGTTTTTCTCTAACAGACACCCCATCAAGGTTCAGTGGGAATGGATTTGGCACAGATTTCACACTCAGCATCAGCAGTATGCAGCCTGAAGATGGTGCCACATATTTCTGCCAGCAGCATTTTAACTATTAC
IGKV20-101-2*01_S8388_PWD	PWD/PHJ	IGKV	MUSMUS IGKV20-101-2*01 F	97.46	AATATCCAGGTGATCCAGTCACCATTTCTGTCTGCATCTGTGGGAGAGAGGGTCACAATCAGCTGCAAGACACATCAGCATATTAACAGTTCCATAGCCTGGTACCAGCAAAAATTTGGAAAAGCTCCCAAACTCCTGATAAGAGATGGAAGTTTTTCTCTAACAGACACCCCATCAAGGTTCAGTGGGAATGGATTTGGCACAGATTTCACACTCAGCATCAGCAGTATGCAGCCTGAAGATGGTGCCACATATTTCTGCCAGCAGCATTTTAACTATTAC
IGKV3-1*01_AJ	A/J	IGKV	MUSMUS IGKV3-1*01 F	100	GACATTGTGCTCACCCAATCTCCAGCTTCTTTGGCTGTGTCTCTAGGGCAGAGAGCCACCATCTCCTGCAGAGCCAGTGAAAGTGTTGAATATTATGGCACAAGTTTAATGCAGTGGTACCAACAGAAACCAGGACAGCCACCCAAACTCCTCATCTATGCTGCATCCAACGTAGAATCTGGGGTCCCTGCCAGGTTTAGTGGCAGTGGGTCTGGGACAGACTTCAGCCTCAACATCCATCCTGTGGAGGAGGATGATATTGCAATGTATTTCTGTCAGCAAAGTAGGAAGGTTCCTTC
IGKV3-1*01_BALB	BALB/CBYJ	IGKV	MUSMUS IGKV3-1*01 F	100	GACATTGTGCTCACCCAATCTCCAGCTTCTTTGGCTGTGTCTCTAGGGCAGAGAGCCACCATCTCCTGCAGAGCCAGTGAAAGTGTTGAATATTATGGCACAAGTTTAATGCAGTGGTACCAACAGAAACCAGGACAGCCACCCAAACTCCTCATCTATGCTGCATCCAACGTAGAATCTGGGGTCCCTGCCAGGTTTAGTGGCAGTGGGTCTGGGACAGACTTCAGCCTCAACATCCATCCTGTGGAGGAGGATGATATTGCAATGTATTTCTGTCAGCAAAGTAGGAAGGTTCCTTC

IGKV3-1*01_C3H	C3H/HEJ	IGKV	MUSMUS IGKV3-1*01 F	100	GACATTGTGCTCACCCAATCTCCAGCTTCTTTGGCTGTGTCTCTAGGGCAGAGAGCCACCATCTCCTGCAGAGCCAGTGAAAGTGTTGAATATTATGGCACAAGTTTAATGCAGTGGTACCAACAGAAACCAGGACAGCCACCCAAACTCCTCATCTATGCTGCATCCAACGTAGAATCTGGGGTCCCTGCCAGGTTTAGTGGCAGTGGGTCTGGGACAGACTTCAGCCTCAACATCCATCCTGTGGAGGAGGATGATATTGCAATGTATTTCTGTCAGCAAAGTAGGAAGGTTCCTTC
IGKV3-1*01_DBA1	DBA/1J	IGKV	MUSMUS IGKV3-1*01 F	100	GACATTGTGCTCACCCAATCTCCAGCTTCTTTGGCTGTGTCTCTAGGGCAGAGAGCCACCATCTCCTGCAGAGCCAGTGAAAGTGTTGAATATTATGGCACAAGTTTAATGCAGTGGTACCAACAGAAACCAGGACAGCCACCCAAACTCCTCATCTATGCTGCATCCAACGTAGAATCTGGGGTCCCTGCCAGGTTTAGTGGCAGTGGGTCTGGGACAGACTTCAGCCTCAACATCCATCCTGTGGAGGAGGATGATATTGCAATGTATTTCTGTCAGCAAAGTAGGAAGGTTCCTTC
IGKV3-1*01_DBA2	DBA/2J	IGKV	MUSMUS IGKV3-1*01 F	100	GACATTGTGCTCACCCAATCTCCAGCTTCTTTGGCTGTGTCTCTAGGGCAGAGAGCCACCATCTCCTGCAGAGCCAGTGAAAGTGTTGAATATTATGGCACAAGTTTAATGCAGTGGTACCAACAGAAACCAGGACAGCCACCCAAACTCCTCATCTATGCTGCATCCAACGTAGAATCTGGGGTCCCTGCCAGGTTTAGTGGCAGTGGGTCTGGGACAGACTTCAGCCTCAACATCCATCCTGTGGAGGAGGATGATATTGCAATGTATTTCTGTCAGCAAAGTAGGAAGGTTCCTTC
IGKV3-1*01_NZB	NZB/BLNJ	IGKV	MUSMUS IGKV3-1*01 F	100	GACATTGTGCTCACCCAATCTCCAGCTTCTTTGGCTGTGTCTCTAGGGCAGAGAGCCACCATCTCCTGCAGAGCCAGTGAAAGTGTTGAATATTATGGCACAAGTTTAATGCAGTGGTACCAACAGAAACCAGGACAGCCACCCAAACTCCTCATCTATGCTGCATCCAACGTAGAATCTGGGGTCCCTGCCAGGTTTAGTGGCAGTGGGTCTGGGACAGACTTCAGCCTCAACATCCATCCTGTGGAGGAGGATGATATTGCAATGTATTTCTGTCAGCAAAGTAGGAAGGTTCCTTC

IGKV3-1*01_S2114_MSM	MSM/MSJ	IGKV	MUSMUS IGKV3-1*01 F	99.66	GACATTGTGCTCACCCAATCTCCAGCTTCTTTGGCTGTGTCTCTAGGGCAGAGAGCCACCATCTCCTGCAGAGCCAGTGAAAGTGTTGAATATTATGGCACAAGTTTAATGCAGTGGTACCAACAGAAACCAGGACAGCCACCCAAAATCCTCATCTATGCTGCATCCAACGTAGAATCTGGGGTCCCTGCCAGGTTTAGTGGCAGTGGGTCTGGGACAGACTTCAGCCTCAACATCCATCCTGTGGAGGAGGATGATATTGCAATGTATTTCTGTCAGCAAAGTAGGAAGGTTCCTTC
IGKV3-1*01_S6564_CAST	CAST/EIJ	IGKV	MUSMUS IGKV3-1*01 F	95.88	GACATTGTGCTGACCCAATCTCCAGCTTCTTTGGCTGTGTCTCTAGGGCAGAGAGCCACCATCTCCTGCAGAACCAGTGAAAGTGTTGATTATGATGGCGATAGTTATATGAACTGGTACCAACAGAAACCAGGACAGCCACCCAAACTCCTCATCTATGGTGCATCCAACGTAGAATCTGGGGTCCCTGCCAGGTTTAGTGGCAGTGGGTCTGGGACAGACTTCAGCCTCAACATCCATCCTGTGGAGGAGGATGATATTGCAATGTATTTCTGTCAGCAAAGTAGGAAGGTTCCTTC
IGKV3-1*01_S7023_PWD	PWD/PHJ	IGKV	MUSMUS IGKV3-1*01 F	99.31	GACATTGTGCTGACCCAATCTCCAGCTTCTTTGGCTGTGTCTCTAGGGCAGAGAGCCACCATCTCCTGCAGAGCCAGTGAAAGTGTTGAATATTATGGCACAAGTTTAATGCAGTGGTACCAACAGAAACCAGGACAGCCACCCAAAATCCTCATCTATGCTGCATCCAACGTAGAATCTGGGGTCCCTGCCAGGTTTAGTGGCAGTGGGTCTGGGACAGACTTCAGCCTCAACATCCATCCTGTGGAGGAGGATGATATTGCAATGTATTTCTGTCAGCAAAGTAGGAAGGTTCCTTC
IGKV3-1*01_S7345_AKR	AKR/J	IGKV	MUSMUS IGKV3-1*01 F	98.63	GACATTGTGCTCACCCAATCTCCAGCTTCTTTGGCTGTGTCTCTAGGGCAGAGTGTCACCATCTCCTGCAGAGCCAGTGAAAGTGTTGAATATTATGGCACTAGTTTAATGCAGTGGTACCAACAGAAACCAGGACAGCCACCCAAACTCCTCATCTATGGTGCATCCAACGTAGAATCTGGGGTCCCTGCCAGGTTTAGTGGCAGTGGGTCTGGGACAGACTTCAGCCTCAACATCCATCCTGTGGAGGAGGATGATATTGCAATGTATTTCTGTCAGCAAAGTAGGAAGGTTCCTTC

IGKV3-1*01_S7345_MRL	MRL/MPJ	IGKV	MUSMUS IGKV3-1*01 F	98.63	GACATTGTGCTCACCCAATCTCCAGCTTCTTTGGCTGTGTCTCTAGGGCAGAGTGTCACCATCTCCTGCAGAGCCAGTGAAAGTGTTGAATATTATGGCACTAGTTTAATGCAGTGGTACCAACAGAAACCAGGACAGCCACCCAAACTCCTCATCTATGGTGCATCCAACGTAGAATCTGGGGTCCCTGCCAGGTTTAGTGGCAGTGGGTCTGGGACAGACTTCAGCCTCAACATCCATCCTGTGGAGGAGGATGATATTGCAATGTATTTCTGTCAGCAAAGTAGGAAGGTTCCTTC
IGKV3-1*01_S7345_NOD	NOD/SHILTJ	IGKV	MUSMUS IGKV3-1*01 F	98.63	GACATTGTGCTCACCCAATCTCCAGCTTCTTTGGCTGTGTCTCTAGGGCAGAGTGTCACCATCTCCTGCAGAGCCAGTGAAAGTGTTGAATATTATGGCACTAGTTTAATGCAGTGGTACCAACAGAAACCAGGACAGCCACCCAAACTCCTCATCTATGGTGCATCCAACGTAGAATCTGGGGTCCCTGCCAGGTTTAGTGGCAGTGGGTCTGGGACAGACTTCAGCCTCAACATCCATCCTGTGGAGGAGGATGATATTGCAATGTATTTCTGTCAGCAAAGTAGGAAGGTTCCTTC
IGKV3-1*01_S7345_NOR	NOR/LTJ	IGKV	MUSMUS IGKV3-1*01 F	98.63	GACATTGTGCTCACCCAATCTCCAGCTTCTTTGGCTGTGTCTCTAGGGCAGAGTGTCACCATCTCCTGCAGAGCCAGTGAAAGTGTTGAATATTATGGCACTAGTTTAATGCAGTGGTACCAACAGAAACCAGGACAGCCACCCAAACTCCTCATCTATGGTGCATCCAACGTAGAATCTGGGGTCCCTGCCAGGTTTAGTGGCAGTGGGTCTGGGACAGACTTCAGCCTCAACATCCATCCTGTGGAGGAGGATGATATTGCAATGTATTTCTGTCAGCAAAGTAGGAAGGTTCCTTC
IGKV3-10*01_129	129S1/SVIMJ	IGKV	MUSMUS IGKV3-10*01 F	100	AACATTGTGCTGACCCAATCTCCAGCTTCTTTGGCTGTGTCTCTAGGGCAGAGGGCCACCATATCCTGCAGAGCCAGTGAAAGTGTTGATAGTTATGGCAATAGTTTTATGCACTGGTACCAGCAGAAACCAGGACAGCCACCCAAACTCCTCATCTATCTTGCATCCAACCTAGAATCTGGGGTCCCTGCCAGGTTCAGTGGCAGTGGGTCTAGGACAGACTTCACCCTCACCATTGATCCTGTGGAGGCTGATGATGCTGCAACCTATTACTGTCAGCAAAATAATGAGGATCCTCC

IGKV3-10*01_AJ	A/J	IGKV	MUSMUS IGKV3-10*01 F	100	AACATTGTGCTGACCCAATCTCCAGCTTCTTTGGCTGTGTCTCTAGGGCAGAGGGCCACCATATCCTGCAGAGCCAGTGAAAGTGTTGATAGTTATGGCAATAGTTTTATGCACTGGTACCAGCAGAAACCAGGACAGCCACCCAAACTCCTCATCTATCTTGCATCCAACCTAGAATCTGGGGTCCCTGCCAGGTTCAGTGGCAGTGGGTCTAGGACAGACTTCACCCTCACCATTGATCCTGTGGAGGCTGATGATGCTGCAACCTATTACTGTCAGCAAAATAATGAGGATCCTCC
IGKV3-10*01_B6	C57BL/6J	IGKV	MUSMUS IGKV3-10*01 F	100	AACATTGTGCTGACCCAATCTCCAGCTTCTTTGGCTGTGTCTCTAGGGCAGAGGGCCACCATATCCTGCAGAGCCAGTGAAAGTGTTGATAGTTATGGCAATAGTTTTATGCACTGGTACCAGCAGAAACCAGGACAGCCACCCAAACTCCTCATCTATCTTGCATCCAACCTAGAATCTGGGGTCCCTGCCAGGTTCAGTGGCAGTGGGTCTAGGACAGACTTCACCCTCACCATTGATCCTGTGGAGGCTGATGATGCTGCAACCTATTACTGTCAGCAAAATAATGAGGATCCTCC
IGKV3-10*01_BALB	BALB/CBYJ	IGKV	MUSMUS IGKV3-10*01 F	100	AACATTGTGCTGACCCAATCTCCAGCTTCTTTGGCTGTGTCTCTAGGGCAGAGGGCCACCATATCCTGCAGAGCCAGTGAAAGTGTTGATAGTTATGGCAATAGTTTTATGCACTGGTACCAGCAGAAACCAGGACAGCCACCCAAACTCCTCATCTATCTTGCATCCAACCTAGAATCTGGGGTCCCTGCCAGGTTCAGTGGCAGTGGGTCTAGGACAGACTTCACCCTCACCATTGATCCTGTGGAGGCTGATGATGCTGCAACCTATTACTGTCAGCAAAATAATGAGGATCCTCC
IGKV3-10*01_C3H	C3H/HEJ	IGKV	MUSMUS IGKV3-10*01 F	100	AACATTGTGCTGACCCAATCTCCAGCTTCTTTGGCTGTGTCTCTAGGGCAGAGGGCCACCATATCCTGCAGAGCCAGTGAAAGTGTTGATAGTTATGGCAATAGTTTTATGCACTGGTACCAGCAGAAACCAGGACAGCCACCCAAACTCCTCATCTATCTTGCATCCAACCTAGAATCTGGGGTCCCTGCCAGGTTCAGTGGCAGTGGGTCTAGGACAGACTTCACCCTCACCATTGATCCTGTGGAGGCTGATGATGCTGCAACCTATTACTGTCAGCAAAATAATGAGGATCCTCC

IGKV3-10*01_CBA	CBA/J	IGKV	MUSMUS IGKV3-10*01 F	100	AACATTGTGCTGACCCAATCTCCAGCTTCTTTGGCTGTGTCTCTAGGGCAGAGGGCCACCATATCCTGCAGAGCCAGTGAAAGTGTTGATAGTTATGGCAATAGTTTTATGCACTGGTACCAGCAGAAACCAGGACAGCCACCCAAACTCCTCATCTATCTTGCATCCAACCTAGAATCTGGGGTCCCTGCCAGGTTCAGTGGCAGTGGGTCTAGGACAGACTTCACCCTCACCATTGATCCTGTGGAGGCTGATGATGCTGCAACCTATTACTGTCAGCAAAATAATGAGGATCCTCC
IGKV3-10*01_DBA1	DBA/1J	IGKV	MUSMUS IGKV3-10*01 F	100	AACATTGTGCTGACCCAATCTCCAGCTTCTTTGGCTGTGTCTCTAGGGCAGAGGGCCACCATATCCTGCAGAGCCAGTGAAAGTGTTGATAGTTATGGCAATAGTTTTATGCACTGGTACCAGCAGAAACCAGGACAGCCACCCAAACTCCTCATCTATCTTGCATCCAACCTAGAATCTGGGGTCCCTGCCAGGTTCAGTGGCAGTGGGTCTAGGACAGACTTCACCCTCACCATTGATCCTGTGGAGGCTGATGATGCTGCAACCTATTACTGTCAGCAAAATAATGAGGATCCTCC
IGKV3-10*01_DBA2	DBA/2J	IGKV	MUSMUS IGKV3-10*01 F	100	AACATTGTGCTGACCCAATCTCCAGCTTCTTTGGCTGTGTCTCTAGGGCAGAGGGCCACCATATCCTGCAGAGCCAGTGAAAGTGTTGATAGTTATGGCAATAGTTTTATGCACTGGTACCAGCAGAAACCAGGACAGCCACCCAAACTCCTCATCTATCTTGCATCCAACCTAGAATCTGGGGTCCCTGCCAGGTTCAGTGGCAGTGGGTCTAGGACAGACTTCACCCTCACCATTGATCCTGTGGAGGCTGATGATGCTGCAACCTATTACTGTCAGCAAAATAATGAGGATCCTCC
IGKV3-10*01_LEWES	LEWES/EIJ	IGKV	MUSMUS IGKV3-10*01 F	100	AACATTGTGCTGACCCAATCTCCAGCTTCTTTGGCTGTGTCTCTAGGGCAGAGGGCCACCATATCCTGCAGAGCCAGTGAAAGTGTTGATAGTTATGGCAATAGTTTTATGCACTGGTACCAGCAGAAACCAGGACAGCCACCCAAACTCCTCATCTATCTTGCATCCAACCTAGAATCTGGGGTCCCTGCCAGGTTCAGTGGCAGTGGGTCTAGGACAGACTTCACCCTCACCATTGATCCTGTGGAGGCTGATGATGCTGCAACCTATTACTGTCAGCAAAATAATGAGGATCCTCC

IGKV3-10*01_NZB	NZB/BLNJ	IGKV	MUSMUS IGKV3-10*01 F	100	AACATTGTGCTGACCCAATCTCCAGCTTCTTTGGCTGTGTCTCTAGGGCAGAGGGCCACCATATCCTGCAGAGCCAGTGAAAGTGTTGATAGTTATGGCAATAGTTTTATGCACTGGTACCAGCAGAAACCAGGACAGCCACCCAAACTCCTCATCTATCTTGCATCCAACCTAGAATCTGGGGTCCCTGCCAGGTTCAGTGGCAGTGGGTCTAGGACAGACTTCACCCTCACCATTGATCCTGTGGAGGCTGATGATGCTGCAACCTATTACTGTCAGCAAAATAATGAGGATCCTCC
IGKV3-10*01_S5437_AKR	AKR/J	IGKV	MUSMUS IGKV3-10*01 F	98.97	AAAATTGTGCTGACCCAATCTCCAGCTTCTTTGGCTGTGTCTCTAAGGCAGAGGGCCACCATATCCTGCAGAGCCAGTGAAAGTGTTGATAGTTATGGCAATAGTTTTATGCACTGGTACCAGCAGAAACCAGGACAGCCACCCAAACTCCTCATCTATCGTGCATCCAACCTAGAATCTGGGGTCCCTGCCAGGTTCAGTGGCAGTGGGTCTAGGACAGACTTCACCCTCACCATTGATCCTGTGGAGGCTGATGATGCTGCAACCTATTACTGTCAGCAAAATAATGAGGATCCTCC
IGKV3-10*01_S5437_MRL	MRL/MPJ	IGKV	MUSMUS IGKV3-10*01 F	98.97	AAAATTGTGCTGACCCAATCTCCAGCTTCTTTGGCTGTGTCTCTAAGGCAGAGGGCCACCATATCCTGCAGAGCCAGTGAAAGTGTTGATAGTTATGGCAATAGTTTTATGCACTGGTACCAGCAGAAACCAGGACAGCCACCCAAACTCCTCATCTATCGTGCATCCAACCTAGAATCTGGGGTCCCTGCCAGGTTCAGTGGCAGTGGGTCTAGGACAGACTTCACCCTCACCATTGATCCTGTGGAGGCTGATGATGCTGCAACCTATTACTGTCAGCAAAATAATGAGGATCCTCC
IGKV3-10*01_S5437_NOR	NOR/LTJ	IGKV	MUSMUS IGKV3-10*01 F	98.97	AAAATTGTGCTGACCCAATCTCCAGCTTCTTTGGCTGTGTCTCTAAGGCAGAGGGCCACCATATCCTGCAGAGCCAGTGAAAGTGTTGATAGTTATGGCAATAGTTTTATGCACTGGTACCAGCAGAAACCAGGACAGCCACCCAAACTCCTCATCTATCGTGCATCCAACCTAGAATCTGGGGTCCCTGCCAGGTTCAGTGGCAGTGGGTCTAGGACAGACTTCACCCTCACCATTGATCCTGTGGAGGCTGATGATGCTGCAACCTATTACTGTCAGCAAAATAATGAGGATCCTCC

IGKV3-10*01_S9898_MSM	MSM/MSJ	IGKV	MUSMUS IGKV3-10*01 F, OR MUSMUS IGKV3-5*01 F	98.97	GACATTGTGCTGACCCAATCTCCAGCTTCTTTGGCTGTGTCTCTAGGGCAGAGGGCCACCATATCCTGCAGAGCCAGTGAAAGTGTTGATAGTTATGGCAATAGTTTTATGCACTGGTACCAGCAGAAACCAGGACAGCCACCCAAACTCCTCATCTATCTTGCATCCAACCTAGAATCTGGGGTCCCTGCCAGGTTCAGTGGCAGTGGGTCTAGGACAGACTTCACCCTCACCATTAATCCTGTGGAGGCTGATGATGTTGCAACCTATTACTGTCAGCAAAATAATGAGGATCCTCC
IGKV3-10*01_S9898_PWD	PWD/PHJ	IGKV	MUSMUS IGKV3-10*01 F, OR MUSMUS IGKV3-5*01 F	98.97	GACATTGTGCTGACCCAATCTCCAGCTTCTTTGGCTGTGTCTCTAGGGCAGAGGGCCACCATATCCTGCAGAGCCAGTGAAAGTGTTGATAGTTATGGCAATAGTTTTATGCACTGGTACCAGCAGAAACCAGGACAGCCACCCAAACTCCTCATCTATCTTGCATCCAACCTAGAATCTGGGGTCCCTGCCAGGTTCAGTGGCAGTGGGTCTAGGACAGACTTCACCCTCACCATTAATCCTGTGGAGGCTGATGATGTTGCAACCTATTACTGTCAGCAAAATAATGAGGATCCTCC
IGKV3-12*01_129	129S1/SVIMJ	IGKV	MUSMUS IGKV3-12*01 F	100	GACATTGTGCTGACACAGTCTCCTGCTTCCTTAGCTGTATCTCTGGGGCAGAGGGCCACCATCTCATGCAGGGCCAGCAAAAGTGTCAGTACATCTGGCTATAGTTATATGCACTGGTACCAACAGAAACCAGGACAGCCACCCAAACTCCTCATCTATCTTGCATCCAACCTAGAATCTGGGGTCCCTGCCAGGTTCAGTGGCAGTGGGTCTGGGACAGACTTCACCCTCAACATCCATCCTGTGGAGGAGGAGGATGCTGCAACCTATTACTGTCAGCACAGTAGGGAGCTTCCTCC
IGKV3-12*01_AJ	A/J	IGKV	MUSMUS IGKV3-12*01 F	100	GACATTGTGCTGACACAGTCTCCTGCTTCCTTAGCTGTATCTCTGGGGCAGAGGGCCACCATCTCATGCAGGGCCAGCAAAAGTGTCAGTACATCTGGCTATAGTTATATGCACTGGTACCAACAGAAACCAGGACAGCCACCCAAACTCCTCATCTATCTTGCATCCAACCTAGAATCTGGGGTCCCTGCCAGGTTCAGTGGCAGTGGGTCTGGGACAGACTTCACCCTCAACATCCATCCTGTGGAGGAGGAGGATGCTGCAACCTATTACTGTCAGCACAGTAGGGAGCTTCCTCC

IGKV3-12*01_B6	C57BL/6J	IGKV	MUSMUS IGKV3-12*01 F	100	GACATTGTGCTGACACAGTCTCCTGCTTCCTTAGCTGTATCTCTGGGGCAGAGGGCCACCATCTCATGCAGGGCCAGCAAAAGTGTCAGTACATCTGGCTATAGTTATATGCACTGGTACCAACAGAAACCAGGACAGCCACCCAAACTCCTCATCTATCTTGCATCCAACCTAGAATCTGGGGTCCCTGCCAGGTTCAGTGGCAGTGGGTCTGGGACAGACTTCACCCTCAACATCCATCCTGTGGAGGAGGAGGATGCTGCAACCTATTACTGTCAGCACAGTAGGGAGCTTCCTCC
IGKV3-12*01_BALB	BALB/CBYJ	IGKV	MUSMUS IGKV3-12*01 F	100	GACATTGTGCTGACACAGTCTCCTGCTTCCTTAGCTGTATCTCTGGGGCAGAGGGCCACCATCTCATGCAGGGCCAGCAAAAGTGTCAGTACATCTGGCTATAGTTATATGCACTGGTACCAACAGAAACCAGGACAGCCACCCAAACTCCTCATCTATCTTGCATCCAACCTAGAATCTGGGGTCCCTGCCAGGTTCAGTGGCAGTGGGTCTGGGACAGACTTCACCCTCAACATCCATCCTGTGGAGGAGGAGGATGCTGCAACCTATTACTGTCAGCACAGTAGGGAGCTTCCTCC
IGKV3-12*01_C3H	C3H/HEJ	IGKV	MUSMUS IGKV3-12*01 F	100	GACATTGTGCTGACACAGTCTCCTGCTTCCTTAGCTGTATCTCTGGGGCAGAGGGCCACCATCTCATGCAGGGCCAGCAAAAGTGTCAGTACATCTGGCTATAGTTATATGCACTGGTACCAACAGAAACCAGGACAGCCACCCAAACTCCTCATCTATCTTGCATCCAACCTAGAATCTGGGGTCCCTGCCAGGTTCAGTGGCAGTGGGTCTGGGACAGACTTCACCCTCAACATCCATCCTGTGGAGGAGGAGGATGCTGCAACCTATTACTGTCAGCACAGTAGGGAGCTTCCTCC
IGKV3-12*01_CBA	CBA/J	IGKV	MUSMUS IGKV3-12*01 F	100	GACATTGTGCTGACACAGTCTCCTGCTTCCTTAGCTGTATCTCTGGGGCAGAGGGCCACCATCTCATGCAGGGCCAGCAAAAGTGTCAGTACATCTGGCTATAGTTATATGCACTGGTACCAACAGAAACCAGGACAGCCACCCAAACTCCTCATCTATCTTGCATCCAACCTAGAATCTGGGGTCCCTGCCAGGTTCAGTGGCAGTGGGTCTGGGACAGACTTCACCCTCAACATCCATCCTGTGGAGGAGGAGGATGCTGCAACCTATTACTGTCAGCACAGTAGGGAGCTTCCTCC

IGKV3-12*01_DBA1	DBA/1J	IGKV	MUSMUS IGKV3-12*01 F	100	GACATTGTGCTGACACAGTCTC<br>CTGCTTCCTTAGCTGTATCTCTG<br>GGGCAGAGGGCCACCATCTCA<br>TGCAGGGCCAGCAAAAGTGTCA<br>GTACATCTGGCTATAGTTATATG<br>CACTGGTACCAACAGAAACCAG<br>GACAGCCACCCAAACTCCTCAT<br>CTATCTTGCATCCAACCTAGAAT<br>CTGGGGTCCCTGCCAGGTTCA<br>GTGGCAGTGGGTCTGGGACAG<br>ACTTCACCCTCAACATCCATCC<br>TGTGGAGGAGGAGGATGCTGC<br>AACCTATTACTGTCAGCACAGTA<br>GGGAGCTTCCTCC
IGKV3-12*01_DBA2	DBA/2J	IGKV	MUSMUS IGKV3-12*01 F	100	GACATTGTGCTGACACAGTCTC<br>CTGCTTCCTTAGCTGTATCTCTG<br>GGGCAGAGGGCCACCATCTCA<br>TGCAGGGCCAGCAAAAGTGTCA<br>GTACATCTGGCTATAGTTATATG<br>CACTGGTACCAACAGAAACCAG<br>GACAGCCACCCAAACTCCTCAT<br>CTATCTTGCATCCAACCTAGAAT<br>CTGGGGTCCCTGCCAGGTTCA<br>GTGGCAGTGGGTCTGGGACAG<br>ACTTCACCCTCAACATCCATCC<br>TGTGGAGGAGGAGGATGCTGC<br>AACCTATTACTGTCAGCACAGTA<br>GGGAGCTTCCTCC
IGKV3-12*01_LEWES	LEWES/EIJ	IGKV	MUSMUS IGKV3-12*01 F	100	GACATTGTGCTGACACAGTCTC<br>CTGCTTCCTTAGCTGTATCTCTG<br>GGGCAGAGGGCCACCATCTCA<br>TGCAGGGCCAGCAAAAGTGTCA<br>GTACATCTGGCTATAGTTATATG<br>CACTGGTACCAACAGAAACCAG<br>GACAGCCACCCAAACTCCTCAT<br>CTATCTTGCATCCAACCTAGAAT<br>CTGGGGTCCCTGCCAGGTTCA<br>GTGGCAGTGGGTCTGGGACAG<br>ACTTCACCCTCAACATCCATCC<br>TGTGGAGGAGGAGGATGCTGC<br>AACCTATTACTGTCAGCACAGTA<br>GGGAGCTTCCTCC
IGKV3-12*01_NZB	NZB/BLNJ	IGKV	MUSMUS IGKV3-12*01 F	100	GACATTGTGCTGACACAGTCTC<br>CTGCTTCCTTAGCTGTATCTCTG<br>GGGCAGAGGGCCACCATCTCA<br>TGCAGGGCCAGCAAAAGTGTCA<br>GTACATCTGGCTATAGTTATATG<br>CACTGGTACCAACAGAAACCAG<br>GACAGCCACCCAAACTCCTCAT<br>CTATCTTGCATCCAACCTAGAAT<br>CTGGGGTCCCTGCCAGGTTCA<br>GTGGCAGTGGGTCTGGGACAG<br>ACTTCACCCTCAACATCCATCC<br>TGTGGAGGAGGAGGATGCTGC<br>AACCTATTACTGTCAGCACAGTA<br>GGGAGCTTCCTCC

IGKV3-12*01_S3040_CAST	CAST/EIJ	IGKV	MUSMUS IGKV3-12*01 F	99.31	GACATTGTGCTGACACAGTCTCCTGCTTCCTTAGCTGTATCTCTGGGGCAGAGGGCCACCATCTCATGCAGGGCCAGCAAAAGTGTCAGTACATCTAGCTATAGTTATATGCACTGGTACCAACAGAAACCAGGACAGCCACCCAAACTCCTCATCTATCGTGCATCCAACCTAGAATCTGGGGTCCCTGCCAGGTTCAGTGGCAGTGGGTCTGGGACAGACTTCACCCTCAACATCCATCCTGTGGAGGAGGAGGATGCTGCAACCTATTACTGTCAGCACAGTAGGGAGCTTCCTCC
IGKV3-12*01_S3543_PWD	PWD/PHJ	IGKV	MUSMUS IGKV3-12*01 F	96.56	GACATTGTGCTGACACAGTCTCCTGCTTCCTTAGCTGTATCTCTGGAGCAGAGGGCCACCATCTCATGCAGGGCCAGCCAAAGTGTCAGTACATCTAGCTATAGTTACATGCACTGGTACCAACAGAAACCAGGAAAGCCACCCAAACTCCTCATCTATGGTGCATCCAACCTAGAATCTGGGGTGCCTGCCAGGTTCAGTGGCAGTGGGTCTGGGACAGACTTCACCCTCAACATCCATCCTGTGGAGGAGGAGGATGCTGCAACATATTACTGTCAGCACAATAGGGAGATTCCTCC
IGKV3-12*01_S4919_MSM	MSM/MSJ	IGKV	MUSMUS IGKV3-12*01 F	98.28	GACATTGTGCTGACACAGTCTCCTGCTTCCTTAGCTGTATCTCTGGGGCAGAGGGCCACCATCTCATGCAGGGCCAGCCAAAGTGTCAGTACATCTAGCTATAGTTACATGCACTGGTACCAACAGAAACCAGGACAGCCACCCAAACTCCTCATCTATTTTGCATCCAACCTAGAATCTGGGGTCCCTGCCAGGTTCAGTGGCAGTGGGTCTGGGACAGACTTCACCCTCAACATCCATCCTGTGGAGGAGGAGGATGCTGCAACATATTACTGTCAGCACAGTAGGGAGTTTCCTCC
IGKV3-12*01_S5721_MSM	MSM/MSJ	IGKV	MUSMUS IGKV3-12*01 F	97.59	GACATTGTGCTGACACAGTCTCCTGCTTCCTTAGCTGTATCTCTGGAGCAGAGGGCCACCATCTCATGCAGGGCCAGCCAAAGTGTCAGTACATCTAGCTATAGTTACATGCACTGGTACCAACAGAAACCAGGACAGCCACCCAAACTCCTCATCTATTTTGCATCCAACCTAGAATCTGGGGTCCCTGCCAGGTTCAGTGGCAGTGGGTCTGGGACAGACTTCACCCTCAACATCCATCCTGTGGAGGAGGAGGATGCTGCAACATATTACTGTCAGCACAATAGGGAGATTCCTCC

IGKV3-12*01_S8984_PWD	PWD/PHJ	IGKV	MUSMUS IGKV3-12*01 F	98.28	GACATTGTGCTGACACAGTCTCCTGCTTCCTTAGCTGTATCTCTGGGGCAGAGGGCCACCATCTCCTGCAGGGCCAGCAAAAGTGTCAGTACATCTAGCTATAGTTACATGCACTGGTACCAACAGAAACCAGGACAGCCACCCAAACTCCTCATCTATTTTGCATCCAACCTAGAATCTGGGGTCCCTGCCAGGTTCAGTGGCAGTGGGTCTGGGACAGACTTCACCCTCAACATCCATCCTGTGGAGGAGGAGGATGCTGCAACATATTACTGTCAGCACAGTAGGGAGTTTCCTCC
IGKV3-2*01_129	129S1/SVIMJ	IGKV	MUSMUS IGKV3-2*01 F	100	GACATTGTGCTGACCCAATCTCCAGCTTCTTTGGCTGTGTCTCTAGGGCAGAGGGCCACCATCTCCTGCAGAGCCAGCGAAAGTGTTGATAATTATGGCATTAGTTTTATGAACTGGTTCCAACAGAAACCAGGACAGCCACCCAAACTCCTCATCTATGCTGCATCCAACCAAGGATCCGGGGTCCCTGCCAGGTTTAGTGGCAGTGGGTCTGGGACAGACTTCAGCCTCAACATCCATCCTATGGAGGAGGATGATACTGCAATGTATTTCTGTCAGCAAAGTAAGGAGGTTCCTCC
IGKV3-2*01_AJ	A/J	IGKV	MUSMUS IGKV3-2*01 F	100	GACATTGTGCTGACCCAATCTCCAGCTTCTTTGGCTGTGTCTCTAGGGCAGAGGGCCACCATCTCCTGCAGAGCCAGCGAAAGTGTTGATAATTATGGCATTAGTTTTATGAACTGGTTCCAACAGAAACCAGGACAGCCACCCAAACTCCTCATCTATGCTGCATCCAACCAAGGATCCGGGGTCCCTGCCAGGTTTAGTGGCAGTGGGTCTGGGACAGACTTCAGCCTCAACATCCATCCTATGGAGGAGGATGATACTGCAATGTATTTCTGTCAGCAAAGTAAGGAGGTTCCTCC
IGKV3-2*01_B6	C57BL/6J	IGKV	MUSMUS IGKV3-2*01 F	100	GACATTGTGCTGACCCAATCTCCAGCTTCTTTGGCTGTGTCTCTAGGGCAGAGGGCCACCATCTCCTGCAGAGCCAGCGAAAGTGTTGATAATTATGGCATTAGTTTTATGAACTGGTTCCAACAGAAACCAGGACAGCCACCCAAACTCCTCATCTATGCTGCATCCAACCAAGGATCCGGGGTCCCTGCCAGGTTTAGTGGCAGTGGGTCTGGGACAGACTTCAGCCTCAACATCCATCCTATGGAGGAGGATGATACTGCAATGTATTTCTGTCAGCAAAGTAAGGAGGTTCCTCC

IGKV3-2*01_BALB	BALB/CBYJ	IGKV	MUSMUS IGKV3-2*01 F	100	GACATTGTGCTGACCCAATCTCCAGCTTCTTTGGCTGTGTCTCTAGGGCAGAGGGCCACCATCTCCTGCAGAGCCAGCGAAAGTGTTGATAATTATGGCATTAGTTTTATGAACTGGTTCCAACAGAAACCAGGACAGCCACCCAAACTCCTCATCTATGCTGCATCCAACCAAGGATCCGGGGTCCCTGCCAGGTTTAGTGGCAGTGGGTCTGGGACAGACTTCAGCCTCAACATCCATCCTATGGAGGAGGATGATACTGCAATGTATTTCTGTCAGCAAAGTAAGGAGGTTCCTCC
IGKV3-2*01_C3H	C3H/HEJ	IGKV	MUSMUS IGKV3-2*01 F	100	GACATTGTGCTGACCCAATCTCCAGCTTCTTTGGCTGTGTCTCTAGGGCAGAGGGCCACCATCTCCTGCAGAGCCAGCGAAAGTGTTGATAATTATGGCATTAGTTTTATGAACTGGTTCCAACAGAAACCAGGACAGCCACCCAAACTCCTCATCTATGCTGCATCCAACCAAGGATCCGGGGTCCCTGCCAGGTTTAGTGGCAGTGGGTCTGGGACAGACTTCAGCCTCAACATCCATCCTATGGAGGAGGATGATACTGCAATGTATTTCTGTCAGCAAAGTAAGGAGGTTCCTCC
IGKV3-2*01_CBA	CBA/J	IGKV	MUSMUS IGKV3-2*01 F	100	GACATTGTGCTGACCCAATCTCCAGCTTCTTTGGCTGTGTCTCTAGGGCAGAGGGCCACCATCTCCTGCAGAGCCAGCGAAAGTGTTGATAATTATGGCATTAGTTTTATGAACTGGTTCCAACAGAAACCAGGACAGCCACCCAAACTCCTCATCTATGCTGCATCCAACCAAGGATCCGGGGTCCCTGCCAGGTTTAGTGGCAGTGGGTCTGGGACAGACTTCAGCCTCAACATCCATCCTATGGAGGAGGATGATACTGCAATGTATTTCTGTCAGCAAAGTAAGGAGGTTCCTCC
IGKV3-2*01_DBA1	DBA/1J	IGKV	MUSMUS IGKV3-2*01 F	100	GACATTGTGCTGACCCAATCTCCAGCTTCTTTGGCTGTGTCTCTAGGGCAGAGGGCCACCATCTCCTGCAGAGCCAGCGAAAGTGTTGATAATTATGGCATTAGTTTTATGAACTGGTTCCAACAGAAACCAGGACAGCCACCCAAACTCCTCATCTATGCTGCATCCAACCAAGGATCCGGGGTCCCTGCCAGGTTTAGTGGCAGTGGGTCTGGGACAGACTTCAGCCTCAACATCCATCCTATGGAGGAGGATGATACTGCAATGTATTTCTGTCAGCAAAGTAAGGAGGTTCCTCC

IGKV3-2*01_DBA2	DBA/2J	IGKV	MUSMUS IGKV3-2*01 F	100	GACATTGTGCTGACCCAATCTCCAGCTTCTTTGGCTGTGTCTCTAGGGCAGAGGGCCACCATCTCCTGCAGAGCCAGCGAAAGTGTTGATAATTATGGCATTAGTTTTATGAACTGGTTCCAACAGAAACCAGGACAGCCACCCAAACTCCTCATCTATGCTGCATCCAACCAAGGATCCGGGGTCCCTGCCAGGTTTAGTGGCAGTGGGTCTGGGACAGACTTCAGCCTCAACATCCATCCTATGGAGGAGGATGATACTGCAATGTATTTCTGTCAGCAAAGTAAGGAGGTTCCTCC
IGKV3-2*01_LEWES	LEWES/EIJ	IGKV	MUSMUS IGKV3-2*01 F	100	GACATTGTGCTGACCCAATCTCCAGCTTCTTTGGCTGTGTCTCTAGGGCAGAGGGCCACCATCTCCTGCAGAGCCAGCGAAAGTGTTGATAATTATGGCATTAGTTTTATGAACTGGTTCCAACAGAAACCAGGACAGCCACCCAAACTCCTCATCTATGCTGCATCCAACCAAGGATCCGGGGTCCCTGCCAGGTTTAGTGGCAGTGGGTCTGGGACAGACTTCAGCCTCAACATCCATCCTATGGAGGAGGATGATACTGCAATGTATTTCTGTCAGCAAAGTAAGGAGGTTCCTCC
IGKV3-2*01_NZB	NZB/BLNJ	IGKV	MUSMUS IGKV3-2*01 F	100	GACATTGTGCTGACCCAATCTCCAGCTTCTTTGGCTGTGTCTCTAGGGCAGAGGGCCACCATCTCCTGCAGAGCCAGCGAAAGTGTTGATAATTATGGCATTAGTTTTATGAACTGGTTCCAACAGAAACCAGGACAGCCACCCAAACTCCTCATCTATGCTGCATCCAACCAAGGATCCGGGGTCCCTGCCAGGTTTAGTGGCAGTGGGTCTGGGACAGACTTCAGCCTCAACATCCATCCTATGGAGGAGGATGATACTGCAATGTATTTCTGTCAGCAAAGTAAGGAGGTTCCTCC
IGKV3-2*01_PWD	PWD/PHJ	IGKV	MUSMUS IGKV3-2*01 F	100	GACATTGTGCTGACCCAATCTCCAGCTTCTTTGGCTGTGTCTCTAGGGCAGAGGGCCACCATCTCCTGCAGAGCCAGCGAAAGTGTTGATAATTATGGCATTAGTTTTATGAACTGGTTCCAACAGAAACCAGGACAGCCACCCAAACTCCTCATCTATGCTGCATCCAACCAAGGATCCGGGGTCCCTGCCAGGTTTAGTGGCAGTGGGTCTGGGACAGACTTCAGCCTCAACATCCATCCTATGGAGGAGGATGATACTGCAATGTATTTCTGTCAGCAAAGTAAGGAGGTTCCTCC

IGKV3-2*01_S2813_MSM	MSM/MSJ	IGKV	MUSMUS IGKV3-2*01 F	99.66	GACATTGTGCTGACCCAATCTCCAGCTTCTTTGGCTGTGTCTCTAGGGCAGAGGGCCACCATCTCCTGCAGAGCCAGCGAAAGTGTTGATAATTATGGCATTAGTTTTATGAACTGGTTCCAACAGAAACCAGGACAGCCACCCAAACTCCTCATCTATGCTGCATCCAACCAAGGATCTGGGGTCCCTGCCAGGTTTAGTGGCAGTGGGTCTGGGACAGACTTCAGCCTCAACATCCATCCTATGGAGGAGGATGATACTGCAATGTATTTCTGTCAGCAAAGTAAGGAGGTTCCTCC
IGKV3-3*01_AJ	A/J	IGKV	MUSMUS IGKV3-3*01 F	100	GACATTGTGCTGACCCAATCTCCAGCTTCTTTGGCTGTGTCTCTAGGACAGAGAGCCACTATCTTCTGCAGAGCCAGCCAGAGTGTCGATTATAATGGAATTAGTTATATGCACTGGTTCCAACAGAAACCAGGACAGCCACCCAAACTCCTCATCTATGCTGCATCCAACCTAGAATCTGGGATCCCTGCCAGGTTCAGTGGCAGTGGGTCTGGGACAGACTTCACCCTCAACATCCATCCTGTGGAGGAGGAAGATGCTGCAACCTATTACTGTCAGCAAAGTATTGAGGATCCTCC
IGKV3-3*01_DBA1	DBA/1J	IGKV	MUSMUS IGKV3-3*01 F	100	GACATTGTGCTGACCCAATCTCCAGCTTCTTTGGCTGTGTCTCTAGGACAGAGAGCCACTATCTTCTGCAGAGCCAGCCAGAGTGTCGATTATAATGGAATTAGTTATATGCACTGGTTCCAACAGAAACCAGGACAGCCACCCAAACTCCTCATCTATGCTGCATCCAACCTAGAATCTGGGATCCCTGCCAGGTTCAGTGGCAGTGGGTCTGGGACAGACTTCACCCTCAACATCCATCCTGTGGAGGAGGAAGATGCTGCAACCTATTACTGTCAGCAAAGTATTGAGGATCCTCC
IGKV3-3*01_DBA2	DBA/2J	IGKV	MUSMUS IGKV3-3*01 F	100	GACATTGTGCTGACCCAATCTCCAGCTTCTTTGGCTGTGTCTCTAGGACAGAGAGCCACTATCTTCTGCAGAGCCAGCCAGAGTGTCGATTATAATGGAATTAGTTATATGCACTGGTTCCAACAGAAACCAGGACAGCCACCCAAACTCCTCATCTATGCTGCATCCAACCTAGAATCTGGGATCCCTGCCAGGTTCAGTGGCAGTGGGTCTGGGACAGACTTCACCCTCAACATCCATCCTGTGGAGGAGGAAGATGCTGCAACCTATTACTGTCAGCAAAGTATTGAGGATCCTCC

IGKV3-<br>4*01_129	129S1/SVI<br>MJ	IGKV	MUSMUS IGKV3-4*01<br>F	100	GACATTGTGCTGACCCAATCTC<br>CAGCTTCTTTGGCTGTGTCTCTA<br>GGGCAGAGGGCCACCATCTCC<br>TGCAAGGCCAGCCAAAGTGTTG<br>ATTATGATGGTGATAGTTATATGA<br>ACTGGTACCAACAGAAACCAGG<br>ACAGCCACCCAAACTCCTCATC<br>TATGCTGCATCCAATCTAGAATC<br>TGGGATCCCAGCCAGGTTTAGT<br>GGCAGTGGGTCTGGGACAGAC<br>TTCACCCTCAACATCCATCCTGT<br>GGAGGAGGAGGATGCTGCAAC<br>CTATTACTGTCAGCAAAGTAATG<br>AGGATCCTCC
IGKV3-<br>4*01_AJ	A/J	IGKV	MUSMUS IGKV3-4*01<br>F	100	GACATTGTGCTGACCCAATCTC<br>CAGCTTCTTTGGCTGTGTCTCTA<br>GGGCAGAGGGCCACCATCTCC<br>TGCAAGGCCAGCCAAAGTGTTG<br>ATTATGATGGTGATAGTTATATGA<br>ACTGGTACCAACAGAAACCAGG<br>ACAGCCACCCAAACTCCTCATC<br>TATGCTGCATCCAATCTAGAATC<br>TGGGATCCCAGCCAGGTTTAGT<br>GGCAGTGGGTCTGGGACAGAC<br>TTCACCCTCAACATCCATCCTGT<br>GGAGGAGGAGGATGCTGCAAC<br>CTATTACTGTCAGCAAAGTAATG<br>AGGATCCTCC
IGKV3-<br>4*01_B6	C57BL/6J	IGKV	MUSMUS IGKV3-4*01<br>F	100	GACATTGTGCTGACCCAATCTC<br>CAGCTTCTTTGGCTGTGTCTCTA<br>GGGCAGAGGGCCACCATCTCC<br>TGCAAGGCCAGCCAAAGTGTTG<br>ATTATGATGGTGATAGTTATATGA<br>ACTGGTACCAACAGAAACCAGG<br>ACAGCCACCCAAACTCCTCATC<br>TATGCTGCATCCAATCTAGAATC<br>TGGGATCCCAGCCAGGTTTAGT<br>GGCAGTGGGTCTGGGACAGAC<br>TTCACCCTCAACATCCATCCTGT<br>GGAGGAGGAGGATGCTGCAAC<br>CTATTACTGTCAGCAAAGTAATG<br>AGGATCCTCC
IGKV3-<br>4*01_BALB	BALB/CBY<br>J	IGKV	MUSMUS IGKV3-4*01<br>F	100	GACATTGTGCTGACCCAATCTC<br>CAGCTTCTTTGGCTGTGTCTCTA<br>GGGCAGAGGGCCACCATCTCC<br>TGCAAGGCCAGCCAAAGTGTTG<br>ATTATGATGGTGATAGTTATATGA<br>ACTGGTACCAACAGAAACCAGG<br>ACAGCCACCCAAACTCCTCATC<br>TATGCTGCATCCAATCTAGAATC<br>TGGGATCCCAGCCAGGTTTAGT<br>GGCAGTGGGTCTGGGACAGAC<br>TTCACCCTCAACATCCATCCTGT<br>GGAGGAGGAGGATGCTGCAAC<br>CTATTACTGTCAGCAAAGTAATG<br>AGGATCCTCC

IGKV3-4*01_C3H	C3H/HEJ	IGKV	MUSMUS IGKV3-4*01 F	100	GACATTGTGCTGACCCAATCTCCAGCTTCTTTGGCTGTGTCTCTAGGGCAGAGGGCCACCATCTCCTGCAAGGCCAGCCAAAGTGTTGATTATGATGGTGATAGTTATATGAACTGGTACCAACAGAAACCAGGACAGCCACCCAAACTCCTCATCTATGCTGCATCCAATCTAGAATCTGGGATCCCAGCCAGGTTTAGTGGCAGTGGGTCTGGGACAGACTTCACCCTCAACATCCATCCTGTGGAGGAGGAGGATGCTGCAACCTATTACTGTCAGCAAAGTAATGAGGATCCTCC
IGKV3-4*01_CBA	CBA/J	IGKV	MUSMUS IGKV3-4*01 F	100	GACATTGTGCTGACCCAATCTCCAGCTTCTTTGGCTGTGTCTCTAGGGCAGAGGGCCACCATCTCCTGCAAGGCCAGCCAAAGTGTTGATTATGATGGTGATAGTTATATGAACTGGTACCAACAGAAACCAGGACAGCCACCCAAACTCCTCATCTATGCTGCATCCAATCTAGAATCTGGGATCCCAGCCAGGTTTAGTGGCAGTGGGTCTGGGACAGACTTCACCCTCAACATCCATCCTGTGGAGGAGGAGGATGCTGCAACCTATTACTGTCAGCAAAGTAATGAGGATCCTCC
IGKV3-4*01_DBA1	DBA/1J	IGKV	MUSMUS IGKV3-4*01 F	100	GACATTGTGCTGACCCAATCTCCAGCTTCTTTGGCTGTGTCTCTAGGGCAGAGGGCCACCATCTCCTGCAAGGCCAGCCAAAGTGTTGATTATGATGGTGATAGTTATATGAACTGGTACCAACAGAAACCAGGACAGCCACCCAAACTCCTCATCTATGCTGCATCCAATCTAGAATCTGGGATCCCAGCCAGGTTTAGTGGCAGTGGGTCTGGGACAGACTTCACCCTCAACATCCATCCTGTGGAGGAGGAGGATGCTGCAACCTATTACTGTCAGCAAAGTAATGAGGATCCTCC
IGKV3-4*01_DBA2	DBA/2J	IGKV	MUSMUS IGKV3-4*01 F	100	GACATTGTGCTGACCCAATCTCCAGCTTCTTTGGCTGTGTCTCTAGGGCAGAGGGCCACCATCTCCTGCAAGGCCAGCCAAAGTGTTGATTATGATGGTGATAGTTATATGAACTGGTACCAACAGAAACCAGGACAGCCACCCAAACTCCTCATCTATGCTGCATCCAATCTAGAATCTGGGATCCCAGCCAGGTTTAGTGGCAGTGGGTCTGGGACAGACTTCACCCTCAACATCCATCCTGTGGAGGAGGAGGATGCTGCAACCTATTACTGTCAGCAAAGTAATGAGGATCCTCC

IGKV3-4*01_LEWES	LEWES/EIJ	IGKV	MUSMUS IGKV3-4*01 F	100	GACATTGTGCTGACCCAATCTC<br>CAGCTTCTTTGGCTGTGTCTCTA<br>GGGCAGAGGGCCACCATCTCC<br>TGCAAGGCCAGCCAAAGTGTTG<br>ATTATGATGGTGATAGTTATATGA<br>ACTGGTACCAACAGAAACCAGG<br>ACAGCCACCCAAACTCCTCATC<br>TATGCTGCATCCAATCTAGAATC<br>TGGGATCCCAGCCAGGTTTAGT<br>GGCAGTGGGTCTGGGACAGAC<br>TTCACCCTCAACATCCATCCTGT<br>GGAGGAGGAGGATGCTGCAAC<br>CTATTACTGTCAGCAAAGTAATG<br>AGGATCCTCC
IGKV3-4*01_NZB	NZB/BLNJ	IGKV	MUSMUS IGKV3-4*01 F	100	GACATTGTGCTGACCCAATCTC<br>CAGCTTCTTTGGCTGTGTCTCTA<br>GGGCAGAGGGCCACCATCTCC<br>TGCAAGGCCAGCCAAAGTGTTG<br>ATTATGATGGTGATAGTTATATGA<br>ACTGGTACCAACAGAAACCAGG<br>ACAGCCACCCAAACTCCTCATC<br>TATGCTGCATCCAATCTAGAATC<br>TGGGATCCCAGCCAGGTTTAGT<br>GGCAGTGGGTCTGGGACAGAC<br>TTCACCCTCAACATCCATCCTGT<br>GGAGGAGGAGGATGCTGCAAC<br>CTATTACTGTCAGCAAAGTAATG<br>AGGATCCTCC
IGKV3-4*01_S0127_CAST	CAST/EIJ	IGKV	MUSMUS IGKV3-4*01 F	96.91	GACATTGTGCTGACCCAATCTC<br>CAGCTTCTTTGGCTGTGTCTCTA<br>GGGCAGAGGGCCACCATCTCC<br>TGCAGAGCCAGCGAAAGTGTTG<br>ATTATGATGGCGATAGTTATATGA<br>ACTGGTACCAACAGAAACCAGG<br>ACAGCCACCGAAACTCCTCATC<br>TATGCTGCATCCAATCTAGAATC<br>TGGGATCCCTGCCAGGTTTAGT<br>GGCAGTGGGTCTGGGACAGAC<br>TTTACCCTCAACATTCATCCTGT<br>GGAGGAGGAGGATGCTGCAAC<br>CTATTACTGTCTGCAAAGTAATG<br>AGGCTCCTCC
IGKV3-4*01_S3446_CAST	CAST/EIJ	IGKV	MUSMUS IGKV3-4*01 F	92.44	GACATTGTGCTGACCCAATCTC<br>CAGCTTCTTTGGCTGTGTCTATA<br>GGGCAGAGGGCCACCATATCC<br>TGCAAGGCCAGCCAGAGTATCA<br>ATTATTACGGTGATAATTATATGC<br>ACTGGTTCCAACAGAAACCAGG<br>ACAGCCAACCAAACTCCTTATC<br>TATGATGCATCCAACCTAGAATC<br>TGGAGTTCCTGACAGGTTCAGT<br>GGCAGTGGGTCTGGGACAGAC<br>TTCACCCTCAACATCCATCCTGT<br>GGAGGAGGAGGATGCTGCAAC<br>CTATTACTGTCAGCAAAGTAAGG<br>AGTTTCCTCC

IGKV3-4*01_S3473_AKR	AKR/J	IGKV	MUSMUS IGKV3-4*01 F	92.44	GACATTGTGCTGACCCAATCTCCAGCTTCTTTGGCTGTGTCTCTAGGGCAGAGGGCCACCATATCCTGCAAGGCCAGCCAGAGTATCAATTATTACGGTGATAATTATATGCACTGGTTCCAACAGAAACCAGGACAGCCAACCAAACTCCTTATCTATGATGCATCCAACCTAGAATCTGGAGTTCCTGACAGGTTCAGTGGCAGTGGGTCTGGGACAGACTTCACCCTCAACATCCATCCTGTGGAGGAGGAGGATGCTGCAACCTATTACTGTCAGCAAAGCAAGGAGTTTCCTCC
IGKV3-4*01_S3473_MRL	MRL/MPJ	IGKV	MUSMUS IGKV3-4*01 F	92.44	GACATTGTGCTGACCCAATCTCCAGCTTCTTTGGCTGTGTCTCTAGGGCAGAGGGCCACCATATCCTGCAAGGCCAGCCAGAGTATCAATTATTACGGTGATAATTATATGCACTGGTTCCAACAGAAACCAGGACAGCCAACCAAACTCCTTATCTATGATGCATCCAACCTAGAATCTGGAGTTCCTGACAGGTTCAGTGGCAGTGGGTCTGGGACAGACTTCACCCTCAACATCCATCCTGTGGAGGAGGAGGATGCTGCAACCTATTACTGTCAGCAAAGCAAGGAGTTTCCTCC
IGKV3-4*01_S3473_NOR	NOR/LTJ	IGKV	MUSMUS IGKV3-4*01 F	92.44	GACATTGTGCTGACCCAATCTCCAGCTTCTTTGGCTGTGTCTCTAGGGCAGAGGGCCACCATATCCTGCAAGGCCAGCCAGAGTATCAATTATTACGGTGATAATTATATGCACTGGTTCCAACAGAAACCAGGACAGCCAACCAAACTCCTTATCTATGATGCATCCAACCTAGAATCTGGAGTTCCTGACAGGTTCAGTGGCAGTGGGTCTGGGACAGACTTCACCCTCAACATCCATCCTGTGGAGGAGGAGGATGCTGCAACCTATTACTGTCAGCAAAGCAAGGAGTTTCCTCC
IGKV3-4*01_S4235_NOD	NOD/SHILTJ	IGKV	MUSMUS IGKV3-4*01 F	92.44	GACATTGTGCTGACCCAATCTCCAGCTTCTTTGGCTGTGTCTCTAGGGCAGAGGGCCACCATATCCTGCAAGGCCAGCCAGAGTATCAATTATTACGGTGATAATTATATGCACTGGTTCCAACAGAAACCAGGACAGCCAACCAAACTCCTTATCTATGATGCATCCAACCTAGAATCTGGAGTTCCTGACAGGTTCAGTGGCAGTGGGTCTGGGACAGACTTCACCCTCAACATCCATCCTGTGGAGGAGGAGGATGCTGCAACCTATTACTGTCAGCAAAGCAAGGAGTTTCCTC

IGKV3-4*01_S8857_AKR	AKR/J	IGKV	MUSMUS IGKV3-4*01 F	97.25	GACATTGTGCTGACCCAATCTCCAGCTTCTTTGGCTGTGTCTCTAGGGCAGAGGGCCACCATCTCCTGCAGAGCCAGCGAAAGTGTTGATTATGATGGCGATAGTTATATGAACTGGTACCAACAGAAACCAGGACAGCCACCGAAACTCCTCATCTATGCTGCATCCAATCTAGAATCTGGGATCCCTGCCAGGTTTAGTGGCAGTGGGTCTGGGACAGACTTCACCCTCAACATTCATCCTGTGGAGGAGGAGGATGCTGCAACCTATTACTGTCTGCAAAGTAATGAGGCTCCTCC
IGKV3-4*01_S8857_MRL	MRL/MPJ	IGKV	MUSMUS IGKV3-4*01 F	97.25	GACATTGTGCTGACCCAATCTCCAGCTTCTTTGGCTGTGTCTCTAGGGCAGAGGGCCACCATCTCCTGCAGAGCCAGCGAAAGTGTTGATTATGATGGCGATAGTTATATGAACTGGTACCAACAGAAACCAGGACAGCCACCGAAACTCCTCATCTATGCTGCATCCAATCTAGAATCTGGGATCCCTGCCAGGTTTAGTGGCAGTGGGTCTGGGACAGACTTCACCCTCAACATTCATCCTGTGGAGGAGGAGGATGCTGCAACCTATTACTGTCTGCAAAGTAATGAGGCTCCTCC
IGKV3-4*01_S8857_NOR	NOR/LTJ	IGKV	MUSMUS IGKV3-4*01 F	97.25	GACATTGTGCTGACCCAATCTCCAGCTTCTTTGGCTGTGTCTCTAGGGCAGAGGGCCACCATCTCCTGCAGAGCCAGCGAAAGTGTTGATTATGATGGCGATAGTTATATGAACTGGTACCAACAGAAACCAGGACAGCCACCGAAACTCCTCATCTATGCTGCATCCAATCTAGAATCTGGGATCCCTGCCAGGTTTAGTGGCAGTGGGTCTGGGACAGACTTCACCCTCAACATTCATCCTGTGGAGGAGGAGGATGCTGCAACCTATTACTGTCTGCAAAGTAATGAGGCTCCTCC
IGKV3-4*01_S9382_NOD	NOD/SHILTJ	IGKV	MUSMUS IGKV3-4*01 F	97.25	GACATTGTGCTGACCCAATCTCCAGCTTCTTTGGCTGTGTCTCTAGGGCAGAGGGCCACCATCTCCTGCAGAGCCAGCGAAAGTGTTGATTATGATGGCGATAGTTATATGAACTGGTACCAACAGAAACCAGGACAGCCACCGAAACTCCTCATCTATGCTGCATCCAATCTAGAATCTGGGATCCCTGCCAGGTTTAGTGGCAGTGGGTCTGGGACAGACTTCACCCTCAACATTCATCCTGTGGAGGAGGAGGATGCTGCAACCTATTACTGTCTGCAAAGTAATGAGGCTCCTC

IGKV3-5*01_129	129S1/SVIMJ	IGKV	MUSMUS IGKV3-5*01 F	100	GACATTGTGCTGACCCAATCTCCAGCTTCTTTGGCTGTGTCTCTAGGGCAGAGGGCCACCATATCCTGCAGAGCCAGTGAAAGTGTTGATAGTTATGGCAATAGTTTTATGCACTGGTACCAGCAGAAACCAGGACAGCCACCCAAACTCCTCATCTATCGTGCATCCAACCTAGAATCTGGGATCCCTGCCAGGTTCAGTGGCAGTGGGTCTAGGACAGACTTCACCCTCACCATTAATCCTGTGGAGGCTGATGATGTTGCAACCTATTACTGTCAGCAAAGTAATGAGGATCCTCC
IGKV3-5*01_AJ	A/J	IGKV	MUSMUS IGKV3-5*01 F	100	GACATTGTGCTGACCCAATCTCCAGCTTCTTTGGCTGTGTCTCTAGGGCAGAGGGCCACCATATCCTGCAGAGCCAGTGAAAGTGTTGATAGTTATGGCAATAGTTTTATGCACTGGTACCAGCAGAAACCAGGACAGCCACCCAAACTCCTCATCTATCGTGCATCCAACCTAGAATCTGGGATCCCTGCCAGGTTCAGTGGCAGTGGGTCTAGGACAGACTTCACCCTCACCATTAATCCTGTGGAGGCTGATGATGTTGCAACCTATTACTGTCAGCAAAGTAATGAGGATCCTCC
IGKV3-5*01_AKR	AKR/J	IGKV	MUSMUS IGKV3-5*01 F	100	GACATTGTGCTGACCCAATCTCCAGCTTCTTTGGCTGTGTCTCTAGGGCAGAGGGCCACCATATCCTGCAGAGCCAGTGAAAGTGTTGATAGTTATGGCAATAGTTTTATGCACTGGTACCAGCAGAAACCAGGACAGCCACCCAAACTCCTCATCTATCGTGCATCCAACCTAGAATCTGGGATCCCTGCCAGGTTCAGTGGCAGTGGGTCTAGGACAGACTTCACCCTCACCATTAATCCTGTGGAGGCTGATGATGTTGCAACCTATTACTGTCAGCAAAGTAATGAGGATCCTCC
IGKV3-5*01_B6	C57BL/6J	IGKV	MUSMUS IGKV3-5*01 F	100	GACATTGTGCTGACCCAATCTCCAGCTTCTTTGGCTGTGTCTCTAGGGCAGAGGGCCACCATATCCTGCAGAGCCAGTGAAAGTGTTGATAGTTATGGCAATAGTTTTATGCACTGGTACCAGCAGAAACCAGGACAGCCACCCAAACTCCTCATCTATCGTGCATCCAACCTAGAATCTGGGATCCCTGCCAGGTTCAGTGGCAGTGGGTCTAGGACAGACTTCACCCTCACCATTAATCCTGTGGAGGCTGATGATGTTGCAACCTATTACTGTCAGCAAAGTAATGAGGATCCTCC

IGKV3-5*01_BALB	BALB/CBYJ	IGKV	MUSMUS IGKV3-5*01 F	100	GACATTGTGCTGACCCAATCTCCAGCTTCTTTGGCTGTGTCTCTAGGGCAGAGGGCCACCATATCCTGCAGAGCCAGTGAAAGTGTTGATAGTTATGGCAATAGTTTTATGCACTGGTACCAGCAGAAACCAGGACAGCCACCCAAACTCCTCATCTATCGTGCATCCAACCTAGAATCTGGGATCCCTGCCAGGTTCAGTGGCAGTGGGTCTAGGACAGACTTCACCCTCACCATTAATCCTGTGGAGGCTGATGATGTTGCAACCTATTACTGTCAGCAAAGTAATGAGGATCCTCC
IGKV3-5*01_C3H	C3H/HEJ	IGKV	MUSMUS IGKV3-5*01 F	100	GACATTGTGCTGACCCAATCTCCAGCTTCTTTGGCTGTGTCTCTAGGGCAGAGGGCCACCATATCCTGCAGAGCCAGTGAAAGTGTTGATAGTTATGGCAATAGTTTTATGCACTGGTACCAGCAGAAACCAGGACAGCCACCCAAACTCCTCATCTATCGTGCATCCAACCTAGAATCTGGGATCCCTGCCAGGTTCAGTGGCAGTGGGTCTAGGACAGACTTCACCCTCACCATTAATCCTGTGGAGGCTGATGATGTTGCAACCTATTACTGTCAGCAAAGTAATGAGGATCCTCC
IGKV3-5*01_CBA	CBA/J	IGKV	MUSMUS IGKV3-5*01 F	100	GACATTGTGCTGACCCAATCTCCAGCTTCTTTGGCTGTGTCTCTAGGGCAGAGGGCCACCATATCCTGCAGAGCCAGTGAAAGTGTTGATAGTTATGGCAATAGTTTTATGCACTGGTACCAGCAGAAACCAGGACAGCCACCCAAACTCCTCATCTATCGTGCATCCAACCTAGAATCTGGGATCCCTGCCAGGTTCAGTGGCAGTGGGTCTAGGACAGACTTCACCCTCACCATTAATCCTGTGGAGGCTGATGATGTTGCAACCTATTACTGTCAGCAAAGTAATGAGGATCCTCC
IGKV3-5*01_DBA1	DBA/1J	IGKV	MUSMUS IGKV3-5*01 F	100	GACATTGTGCTGACCCAATCTCCAGCTTCTTTGGCTGTGTCTCTAGGGCAGAGGGCCACCATATCCTGCAGAGCCAGTGAAAGTGTTGATAGTTATGGCAATAGTTTTATGCACTGGTACCAGCAGAAACCAGGACAGCCACCCAAACTCCTCATCTATCGTGCATCCAACCTAGAATCTGGGATCCCTGCCAGGTTCAGTGGCAGTGGGTCTAGGACAGACTTCACCCTCACCATTAATCCTGTGGAGGCTGATGATGTTGCAACCTATTACTGTCAGCAAAGTAATGAGGATCCTCC

IGKV3-5*01_DBA2	DBA/2J	IGKV	MUSMUS IGKV3-5*01 F	100	GACATTGTGCTGACCCAATCTCCAGCTTCTTTGGCTGTGTCTCTAGGGCAGAGGGCCACCATATCCTGCAGAGCCAGTGAAAGTGTTGATAGTTATGGCAATAGTTTTATGCACTGGTACCAGCAGAAACCAGGACAGCCACCCAAACTCCTCATCTATCGTGCATCCAACCTAGAATCTGGGATCCCTGCCAGGTTCAGTGGCAGTGGGTCTAGGACAGACTTCACCCTCACCATTAATCCTGTGGAGGCTGATGATGTTGCAACCTATTACTGTCAGCAAAGTAATGAGGATCCTCC
IGKV3-5*01_LEWES	LEWES/EIJ	IGKV	MUSMUS IGKV3-5*01 F	100	GACATTGTGCTGACCCAATCTCCAGCTTCTTTGGCTGTGTCTCTAGGGCAGAGGGCCACCATATCCTGCAGAGCCAGTGAAAGTGTTGATAGTTATGGCAATAGTTTTATGCACTGGTACCAGCAGAAACCAGGACAGCCACCCAAACTCCTCATCTATCGTGCATCCAACCTAGAATCTGGGATCCCTGCCAGGTTCAGTGGCAGTGGGTCTAGGACAGACTTCACCCTCACCATTAATCCTGTGGAGGCTGATGATGTTGCAACCTATTACTGTCAGCAAAGTAATGAGGATCCTCC
IGKV3-5*01_NZB	NZB/BLNJ	IGKV	MUSMUS IGKV3-5*01 F	100	GACATTGTGCTGACCCAATCTCCAGCTTCTTTGGCTGTGTCTCTAGGGCAGAGGGCCACCATATCCTGCAGAGCCAGTGAAAGTGTTGATAGTTATGGCAATAGTTTTATGCACTGGTACCAGCAGAAACCAGGACAGCCACCCAAACTCCTCATCTATCGTGCATCCAACCTAGAATCTGGGATCCCTGCCAGGTTCAGTGGCAGTGGGTCTAGGACAGACTTCACCCTCACCATTAATCCTGTGGAGGCTGATGATGTTGCAACCTATTACTGTCAGCAAAGTAATGAGGATCCTCC
IGKV3-5*01_S5428_SJL	SJL/J	IGKV	MUSMUS IGKV3-5*01 F	97.94	GACATTGTGCTGACCCAATCTCCAGCTTCTTTGGCTGTGTCTCTAGGGCAGAGGGCCACCATCTCCTGCAGAGCCAGCGAAAGTGTTGATAATTATGGCATTAGTTTTATGCACTGGTACCAGCAGAAACCAGGACAGCCACCCAAACTCCTCATCTATCGTGCATCCAACCTAGAATCTGGGATCCCTGCCAGGTTCAGTGGCAGTGGGTCTAGGACAGACTTCACCCTCACCATTAATCCTGTGGAGACTGATGATGTTGCAACCTATTACTGTCAGCAAAGTAATAAGGATCCTCC

IGKV3-7*01_S5296_129	129S1/SVIMJ	IGKV	MUSMUS IGKV3-7*02 F	100	GACATTGTGCTGACACAGTCTCCTGCTTCCTTAGCTGTATCTCTGGGGCAGAGGGCCACCATCTCATGCAGGGCCAGCCAAAGTGTCAGTACATCTAGCTATAGTTATATGCACTGGTACCAACAGAAACCAGGACAGCCACCCAAACTCCTCATCAAGTATGCATCCAACCTAGAATCTGGGGTCCCTGCCAGGTTCAGTGGCAGTGGGTCTGGGACAGACTTCACCCTCAACATCCATCCTGTGGAGGAGGAGGATACTGCAACATATTACTGTCAGCACAGTTGGGAGATTCCTCC
IGKV3-7*01_S5296_AJ	A/J	IGKV	MUSMUS IGKV3-7*02 F	100	GACATTGTGCTGACACAGTCTCCTGCTTCCTTAGCTGTATCTCTGGGGCAGAGGGCCACCATCTCATGCAGGGCCAGCCAAAGTGTCAGTACATCTAGCTATAGTTATATGCACTGGTACCAACAGAAACCAGGACAGCCACCCAAACTCCTCATCAAGTATGCATCCAACCTAGAATCTGGGGTCCCTGCCAGGTTCAGTGGCAGTGGGTCTGGGACAGACTTCACCCTCAACATCCATCCTGTGGAGGAGGAGGATACTGCAACATATTACTGTCAGCACAGTTGGGAGATTCCTCC
IGKV3-7*01_S5296_B6	C57BL/6J	IGKV	MUSMUS IGKV3-7*02 F	100	GACATTGTGCTGACACAGTCTCCTGCTTCCTTAGCTGTATCTCTGGGGCAGAGGGCCACCATCTCATGCAGGGCCAGCCAAAGTGTCAGTACATCTAGCTATAGTTATATGCACTGGTACCAACAGAAACCAGGACAGCCACCCAAACTCCTCATCAAGTATGCATCCAACCTAGAATCTGGGGTCCCTGCCAGGTTCAGTGGCAGTGGGTCTGGGACAGACTTCACCCTCAACATCCATCCTGTGGAGGAGGAGGATACTGCAACATATTACTGTCAGCACAGTTGGGAGATTCCTCC
IGKV3-7*01_S5296_BALB	BALB/CBYJ	IGKV	MUSMUS IGKV3-7*02 F	100	GACATTGTGCTGACACAGTCTCCTGCTTCCTTAGCTGTATCTCTGGGGCAGAGGGCCACCATCTCATGCAGGGCCAGCCAAAGTGTCAGTACATCTAGCTATAGTTATATGCACTGGTACCAACAGAAACCAGGACAGCCACCCAAACTCCTCATCAAGTATGCATCCAACCTAGAATCTGGGGTCCCTGCCAGGTTCAGTGGCAGTGGGTCTGGGACAGACTTCACCCTCAACATCCATCCTGTGGAGGAGGAGGATACTGCAACATATTACTGTCAGCACAGTTGGGAGATTCCTCC

IGKV3-7*01_S5296_C3H	C3H/HEJ	IGKV	MUSMUS IGKV3-7*02 F	100	GACATTGTGCTGACACAGTCTC<br>CTGCTTCCTTAGCTGTATCTCTG<br>GGGCAGAGGGCCACCATCTCA<br>TGCAGGGCCAGCCAAAGTGTC<br>AGTACATCTAGCTATAGTTATATG<br>CACTGGTACCAACAGAAACCAG<br>GACAGCCACCCAAACTCCTCAT<br>CAAGTATGCATCCAACCTAGAAT<br>CTGGGGTCCCTGCCAGGTTCA<br>GTGGCAGTGGGTCTGGGACAG<br>ACTTCACCCTCAACATCCATCC<br>TGTGGAGGAGGAGGATACTGCA<br>ACATATTACTGTCAGCACAGTTG<br>GGAGATTCCTCC
IGKV3-7*01_S5296_CBA	CBA/J	IGKV	MUSMUS IGKV3-7*02 F	100	GACATTGTGCTGACACAGTCTC<br>CTGCTTCCTTAGCTGTATCTCTG<br>GGGCAGAGGGCCACCATCTCA<br>TGCAGGGCCAGCCAAAGTGTC<br>AGTACATCTAGCTATAGTTATATG<br>CACTGGTACCAACAGAAACCAG<br>GACAGCCACCCAAACTCCTCAT<br>CAAGTATGCATCCAACCTAGAAT<br>CTGGGGTCCCTGCCAGGTTCA<br>GTGGCAGTGGGTCTGGGACAG<br>ACTTCACCCTCAACATCCATCC<br>TGTGGAGGAGGAGGATACTGCA<br>ACATATTACTGTCAGCACAGTTG<br>GGAGATTCCTCC
IGKV3-7*01_S5296_DBA1	DBA/1J	IGKV	MUSMUS IGKV3-7*02 F	100	GACATTGTGCTGACACAGTCTC<br>CTGCTTCCTTAGCTGTATCTCTG<br>GGGCAGAGGGCCACCATCTCA<br>TGCAGGGCCAGCCAAAGTGTC<br>AGTACATCTAGCTATAGTTATATG<br>CACTGGTACCAACAGAAACCAG<br>GACAGCCACCCAAACTCCTCAT<br>CAAGTATGCATCCAACCTAGAAT<br>CTGGGGTCCCTGCCAGGTTCA<br>GTGGCAGTGGGTCTGGGACAG<br>ACTTCACCCTCAACATCCATCC<br>TGTGGAGGAGGAGGATACTGCA<br>ACATATTACTGTCAGCACAGTTG<br>GGAGATTCCTCC
IGKV3-7*01_S5296_DBA2	DBA/2J	IGKV	MUSMUS IGKV3-7*02 F	100	GACATTGTGCTGACACAGTCTC<br>CTGCTTCCTTAGCTGTATCTCTG<br>GGGCAGAGGGCCACCATCTCA<br>TGCAGGGCCAGCCAAAGTGTC<br>AGTACATCTAGCTATAGTTATATG<br>CACTGGTACCAACAGAAACCAG<br>GACAGCCACCCAAACTCCTCAT<br>CAAGTATGCATCCAACCTAGAAT<br>CTGGGGTCCCTGCCAGGTTCA<br>GTGGCAGTGGGTCTGGGACAG<br>ACTTCACCCTCAACATCCATCC<br>TGTGGAGGAGGAGGATACTGCA<br>ACATATTACTGTCAGCACAGTTG<br>GGAGATTCCTCC

IGKV3-7*01_S5296_LEWES	LEWES/EIJ	IGKV	MUSMUS IGKV3-7*02 F	100	GACATTGTGCTGACACAGTCTCCTGCTTCCTTAGCTGTATCTCTGGGGCAGAGGGCCACCATCTCATGCAGGGCCAGCCAAAGTGTCAGTACATCTAGCTATAGTTATATGCACTGGTACCAACAGAAACCAGGACAGCCACCCAAACTCCTCATCAAGTATGCATCCAACCTAGAATCTGGGGTCCCTGCCAGGTTCAGTGGCAGTGGGTCTGGGACAGACTTCACCCTCAACATCCATCCTGTGGAGGAGGAGGATACTGCAACATATTACTGTCAGCACAGTTGGGAGATTCCTCC
IGKV3-7*01_S5296_NZB	NZB/BLNJ	IGKV	MUSMUS IGKV3-7*02 F	100	GACATTGTGCTGACACAGTCTCCTGCTTCCTTAGCTGTATCTCTGGGGCAGAGGGCCACCATCTCATGCAGGGCCAGCCAAAGTGTCAGTACATCTAGCTATAGTTATATGCACTGGTACCAACAGAAACCAGGACAGCCACCCAAACTCCTCATCAAGTATGCATCCAACCTAGAATCTGGGGTCCCTGCCAGGTTCAGTGGCAGTGGGTCTGGGACAGACTTCACCCTCAACATCCATCCTGTGGAGGAGGAGGATACTGCAACATATTACTGTCAGCACAGTTGGGAGATTCCTCC
IGKV3-7*01_S5296_SJL	SJL/J	IGKV	MUSMUS IGKV3-7*02 F	100	GACATTGTGCTGACACAGTCTCCTGCTTCCTTAGCTGTATCTCTGGGGCAGAGGGCCACCATCTCATGCAGGGCCAGCCAAAGTGTCAGTACATCTAGCTATAGTTATATGCACTGGTACCAACAGAAACCAGGACAGCCACCCAAACTCCTCATCAAGTATGCATCCAACCTAGAATCTGGGGTCCCTGCCAGGTTCAGTGGCAGTGGGTCTGGGACAGACTTCACCCTCAACATCCATCCTGTGGAGGAGGAGGATACTGCAACATATTACTGTCAGCACAGTTGGGAGATTCCTCC
IGKV3-7*01_S6705_AKR	AKR/J	IGKV	MUSMUS IGKV3-7*02 F	97.59	GACATTGTGCTGACACAGTCTCCTGCTTCCTTAGCTGTATCTCTGGGGCAGAGGGCCACCATCTCCTGCAGGGCCAGCAAAAGTGTCAGTACATCTAGCTATAGTTACATGCACTGGTACCAACAGAAACCAGGACAGCCACCCAAACTCCTCATCAAGTATGCATCCTACCTAGAATCTGGGGTTCCTGCCAGGTTCAGTGGCAGTGGGTCTGGGACAGACTTCACCCTCAACATCCATCCTGTGGAGGAGGAGGATGCTGCAACATATTACTGTCAGCACAGTAGGGAGTTTCCTCC

IGKV3-7*01_S6705_NOD	NOD/SHILTJ	IGKV	MUSMUS IGKV3-7*02 F	97.59	GACATTGTGCTGACACAGTCTCCTGCTTCCTTAGCTGTATCTCTGGGGCAGAGGGCCACCATCTCCTGCAGGGCCAGCAAAAGTGTCAGTACATCTAGCTATAGTTACATGCACTGGTACCAACAGAAACCAGGACAGCCACCCAAACTCCTCATCAAGTATGCATCCTACCTAGAATCTGGGGTTCCTGCCAGGTTCAGTGGCAGTGGGTCTGGGACAGACTTCACCCTCAACATCCATCCTGTGGAGGAGGAGGATGCTGCAACATATTACTGTCAGCACAGTAGGGAGTTTCCTCC
IGKV3-7*01_S6705_NOR	NOR/LTJ	IGKV	MUSMUS IGKV3-7*02 F	97.59	GACATTGTGCTGACACAGTCTCCTGCTTCCTTAGCTGTATCTCTGGGGCAGAGGGCCACCATCTCCTGCAGGGCCAGCAAAAGTGTCAGTACATCTAGCTATAGTTACATGCACTGGTACCAACAGAAACCAGGACAGCCACCCAAACTCCTCATCAAGTATGCATCCTACCTAGAATCTGGGGTTCCTGCCAGGTTCAGTGGCAGTGGGTCTGGGACAGACTTCACCCTCAACATCCATCCTGTGGAGGAGGAGGATGCTGCAACATATTACTGTCAGCACAGTAGGGAGTTTCCTCC
IGKV3-7*01_S6705A_MRL	MRL/MPJ	IGKV	MUSMUS IGKV3-7*02 F	97.59	GACATTGTGCTGACACAGTCTCCTGCTTCCTTAGCTGTATCTCTGGGGCAGAGGGCCACCATCTCCTGCAGGGCCAGCAAAAGTGTCAGTACATCTAGCTATAGTTACATGCACTGGTACCAACAGAAACCAGGACAGCCACCCAAACTCCTCATCAAGTATGCATCCTACCTAGAATCTGGGGTTCCTGCCAGGTTCAGTGGCAGTGGGTCTGGGACAGACTTCACCCTCAACATCCATCCTGTGGAGGAGGAGGATGCTGCAACATATTACTGTCAGCACAGTAGGGAGTTTCCTCC
IGKV3-7*01_S8881_CAST	CAST/EIJ	IGKV	MUSMUS IGKV3-7*02 F	99.66	GACATTGTGCTGACACAGTCTCCTGCTTCCTTAGCTGTATCTCTGGGGCAGAGGGCCACCATCTCATGCAGGGCCAGCCAAAGTGTCAGTACATCTAGCTATAGTTATATGCACTGGTACCAACAGAAACCAGGACAGCCACCCAAACTCCTCATCAGGTATGCATCCAACCTAGAATCTGGGGTCCCTGCCAGGTTCAGTGGCAGTGGGTCTGGGACAGACTTCACCCTCAACATCCATCCTGTGGAGGAGGAGGATACTGCAACATATTACTGTCAGCACAGTTGGGAGATTCCTCC

IGKV3-9*01_BALB	BALB/CBYJ	IGKV	MUSMUS IGKV3-9*01 F	100	GACATTGTGCTGACCCAATCTCCAGCTTCTTTGGCTGTGTCTCTAGGACAGAGGGCCACCATATCCTGCCAAGCCAGCGAAAGTGTCAGTTTTGCTGGTACAAGTTTAATGCACTGGTACCAACAGAAACCAGGACAGCCACCCAAACTCCTCATCTATCGTGCATCCAACCTAGAATCTGGAGTCCCTGCCAGGTTCAGTGGCAGTGGGTCTGAGTCAGACTTCACTCTCACCATCGATCCTGTGGAGGAAGATGATGCTGCAATGTATTACTGTATGCAAAGTATGGAAGATCCTCC
IGKV3-9*01_DBA2	DBA/2J	IGKV	MUSMUS IGKV3-9*01 F	100	GACATTGTGCTGACCCAATCTCCAGCTTCTTTGGCTGTGTCTCTAGGACAGAGGGCCACCATATCCTGCCAAGCCAGCGAAAGTGTCAGTTTTGCTGGTACAAGTTTAATGCACTGGTACCAACAGAAACCAGGACAGCCACCCAAACTCCTCATCTATCGTGCATCCAACCTAGAATCTGGAGTCCCTGCCAGGTTCAGTGGCAGTGGGTCTGAGTCAGACTTCACTCTCACCATCGATCCTGTGGAGGAAGATGATGCTGCAATGTATTACTGTATGCAAAGTATGGAAGATCCTCC
IGKV3-9*01_NZB	NZB/BLNJ	IGKV	MUSMUS IGKV3-9*01 F	100	GACATTGTGCTGACCCAATCTCCAGCTTCTTTGGCTGTGTCTCTAGGACAGAGGGCCACCATATCCTGCCAAGCCAGCGAAAGTGTCAGTTTTGCTGGTACAAGTTTAATGCACTGGTACCAACAGAAACCAGGACAGCCACCCAAACTCCTCATCTATCGTGCATCCAACCTAGAATCTGGAGTCCCTGCCAGGTTCAGTGGCAGTGGGTCTGAGTCAGACTTCACTCTCACCATCGATCCTGTGGAGGAAGATGATGCTGCAATGTATTACTGTATGCAAAGTATGGAAGATCCTCC
IGKV3-9*01_S0307_MSM	MSM/MSJ	IGKV	MUSMUS IGKV3-9*01 F	91.07	GACATTGTGCTGACCCAATCTCCAGCTTCTTTGGCTGTGTCTCTAGGGCAGAGGGCCACCATCTCCTGCAGAGCCAGCGAAAGTGTCAGTATTCGTGGTACTAGTTTAATGCACTGGTACCAACAGAAACCAGGACAGCCACCCAAAGTCCTCATCTATCTTGCATCCAACCTAGAATCTGGGGTTCCTGCAAGGTTTAGTGGCAGTGGGTCTGGGACAGGCTTCACCCTCAATATCCATCCTGTGGAGGAAGATGATGCTGCAACCTATTACTGTCAGCAAAGTAGGGAATATCCTCC

IGKV3-9*01_S0307_PWD	PWD/PHJ	IGKV	MUSMUS IGKV3-9*01 F	91.07	GACATTGTGCTGACCCAATCTCCAGCTTCTTTGGCTGTGTCTCTAGGGCAGAGGGCCACCATCTCCTGCAGAGCCAGCGAAAGTGTCAGTATTCGTGGTACTAGTTTAATGCACTGGTACCAACAGAAACCAGGACAGCCACCCAAAGTCCTCATCTATCTTGCATCCAACCTAGAATCTGGGGTTCCTGCAAGGTTTAGTGGCAGTGGGTCTGGGACAGGCTTCACCCTCAATATCCATCCTGTGGAGGAAGATGATGCTGCAACCTATTACTGTCAGCAAAGTAGGGAATATCCTCC
IGKV3-9*01_S2885_DBA1	DBA/1J	IGKV	MUSMUS IGKV3-9*01 F	99.66	GACATTGTGCTGACCCAATCTCCAGCTTCTTTGGCTGTGTCTCTAGGACAGAGGGCCACCATATCCTGCCAAGCCAGCGAAAGTGTCAGTTTTGCTGGTACAAGTTTAATGCACTGGTACCAACAGAAACCAGGACAGCCACCCAAACTCCTCATCTATCGTGCATCCAACCTGGAATCTGGAGTCCCTGCCAGGTTCAGTGGCAGTGGGTCTGAGTCAGACTTCACTCTCACCATCGATCCTGTGGAGGAAGATGATGCTGCAATGTATTACTGTATGCAAAGTATGGAAGATCC
IGKV3-9*01_S4460_MSM	MSM/MSJ	IGKV	MUSMUS IGKV3-9*01 F	92.1	GACATTGTGCTGACCCAATCTCCAGCTTCTTTGGCTGTGTCTCTAGGGCAGAGGGCCACCATCTCCTGCAAGGCCAGCCAAAGTGTCAGTTTTGCTGGTACTAGTTTAATGCACTGGTACCAACAGAAACCAGGACAGCCACCCAAAGTCCTCATCTATCGTGCATCCAACCTAGAATCTGGGGTTCCTACCAGGTTTAGTGGCAGTGGGTCTGGGACAGACTTCACCCTCAATATCCACCCTGTGGAGGAAGATGATGCTGCAACCTATTACTGTCAGCAAAGTAGGGAATATCCTCC
IGKV3-9*01_S4460_PWD	PWD/PHJ	IGKV	MUSMUS IGKV3-9*01 F	92.1	GACATTGTGCTGACCCAATCTCCAGCTTCTTTGGCTGTGTCTCTAGGGCAGAGGGCCACCATCTCCTGCAAGGCCAGCCAAAGTGTCAGTTTTGCTGGTACTAGTTTAATGCACTGGTACCAACAGAAACCAGGACAGCCACCCAAAGTCCTCATCTATCGTGCATCCAACCTAGAATCTGGGGTTCCTACCAGGTTTAGTGGCAGTGGGTCTGGGACAGACTTCACCCTCAATATCCACCCTGTGGAGGAAGATGATGCTGCAACCTATTACTGTCAGCAAAGTAGGGAATATCCTCC

IGKV3-9*01_S5964_AKR	AKR/J	IGKV	MUSMUS IGKV3-9*01 F	90.38	GACATTGTGCTGACCCAATCTCCAGCTTCTTTGGCTGTGTCTCTAGGGCAGAGGGCCACCATCTCCTGCAGAGCCAGCGAAAGTGTCAGTATTCGTGGTACTAGTTTAATGCACTGGTACCAACAGAAACCAGGATATCCACCCAAACTCCTCATCTATGCTGCATCCAACCTAGAATCTGGGGTTCCTGCCAGGTTTAGTGGCAGAAGGTCTGGGACAGACTTCACCCTCAACATTCATCCTGTGGAGGAAGATGATGCTGCAACCTATTACTGTCAGCAAAGTAGGGAATATCCTCC
IGKV3-9*01_S7901_SJL	SJL/J	IGKV	MUSMUS IGKV3-9*01 F	91.41	GACATTGTGCTGACCCAATCTCCAGCTTCTTTGGCTGTGTCTCTAGGGCAGAGGGCCATCATCTCCTGCAAGGCCAGCCAAAGTGTCAGTTTTGCTGGTACTAGTTTAATGCACTGGTACCAACAGAAACCAGGACAGCAACCCAAACTCCTCATCTATCGTGCATCCAACCTAGAAGCTGGGGTTCCTACCAGGTTTAGTGGCAGTGGGTCTAGGACAGACTTCACCCTCAATATCCATCCTGTGGAGGAAGATGATGCTGCAACCTATTACTGTCAGCAAAGTAGGGAATATCCTCC
IGKV3-9*01_S8945_PWD	PWD/PHJ	IGKV	MUSMUS IGKV3-9*01 F	92.1	GACATTGTGCTGACCCAATCTCCAGCTTCTTTGACTGTGTCTCTAGGGCAGAGAGCCACCATCTCCTGCAGAGCCAGTGAAAGTGTCAGTTTTGCTGGTACTAGTTTAATGCACTGGTACCAACAGAAACCAGGACAGCCACCCAAACTCCTCATCTATCTTGCATCCAACCTAGAATCTGGGGTTCCTGCCAGGTTTAGTGGCAGTGGGTCTGGGACAGACTTCACCCTCAATATCCATCCTGTGGAGGAAGATGATGCTGCAACCTATTACTGTCAGCAAAGTAGGGAATATCCTCC
IGKV4-50*01_AJ	A/J	IGKV	MUSMUS IGKV4-50*01 F	100	GAAAATGTGCTCACCCAGTCTCCAGCAATCATGTCTGCATCTCTAGGGGAGAAGGTCACCATGAGCTGCAGGGCCAGCTCAAGTGTAAATTACATGTACTGGTACCAGCAGAAGTCAGATGCCTCCCCCAAACTATGGATTTATTACACATCCAACCTGGCTCCTGGAGTCCCAGCTCGCTTCAGTGGCAGTGGGTCTGGGAACTCTTATTCTCTCACAATCAGCAGCATGGAGGGTGAAGATGCTGCCACTTATTACTGCCAGCAGTTTACTAGTTCCCCATCCA

IGKV4-50*01_BALB	BALB/CBYJ	IGKV	MUSMUS IGKV4-50*01 F	100	GAAAATGTGCTCACCCAGTCTCCAGCAATCATGTCTGCATCTCTAGGGGAGAAGGTCACCATGAGCTGCAGGGCCAGCTCAAGTGTAAATTACATGTACTGGTACCAGCAGAAGTCAGATGCCTCCCCCAAACTATGGATTTATTACACATCCAACCTGGCTCCTGGAGTCCCAGCTCGCTTCAGTGGCAGTGGGTCTGGGAACTCTTATTCTCTCACAATCAGCAGCATGGAGGGTGAAGATGCTGCCACTTATTACTGCCAGCAGTTTACTAGTTCCCCATCCA
IGKV4-50*01_C3H	C3H/HEJ	IGKV	MUSMUS IGKV4-50*01 F	100	GAAAATGTGCTCACCCAGTCTCCAGCAATCATGTCTGCATCTCTAGGGGAGAAGGTCACCATGAGCTGCAGGGCCAGCTCAAGTGTAAATTACATGTACTGGTACCAGCAGAAGTCAGATGCCTCCCCCAAACTATGGATTTATTACACATCCAACCTGGCTCCTGGAGTCCCAGCTCGCTTCAGTGGCAGTGGGTCTGGGAACTCTTATTCTCTCACAATCAGCAGCATGGAGGGTGAAGATGCTGCCACTTATTACTGCCAGCAGTTTACTAGTTCCCCATCCA
IGKV4-50*01_CBA	CBA/J	IGKV	MUSMUS IGKV4-50*01 F	100	GAAAATGTGCTCACCCAGTCTCCAGCAATCATGTCTGCATCTCTAGGGGAGAAGGTCACCATGAGCTGCAGGGCCAGCTCAAGTGTAAATTACATGTACTGGTACCAGCAGAAGTCAGATGCCTCCCCCAAACTATGGATTTATTACACATCCAACCTGGCTCCTGGAGTCCCAGCTCGCTTCAGTGGCAGTGGGTCTGGGAACTCTTATTCTCTCACAATCAGCAGCATGGAGGGTGAAGATGCTGCCACTTATTACTGCCAGCAGTTTACTAGTTCCCCATCCA
IGKV4-50*01_LEWES	LEWES/EIJ	IGKV	MUSMUS IGKV4-50*01 F	100	GAAAATGTGCTCACCCAGTCTCCAGCAATCATGTCTGCATCTCTAGGGGAGAAGGTCACCATGAGCTGCAGGGCCAGCTCAAGTGTAAATTACATGTACTGGTACCAGCAGAAGTCAGATGCCTCCCCCAAACTATGGATTTATTACACATCCAACCTGGCTCCTGGAGTCCCAGCTCGCTTCAGTGGCAGTGGGTCTGGGAACTCTTATTCTCTCACAATCAGCAGCATGGAGGGTGAAGATGCTGCCACTTATTACTGCCAGCAGTTTACTAGTTCCCCATCCA

IGKV4-50*01_NZB	NZB/BLNJ	IGKV	MUSMUS IGKV4-50*01 F	100	GAAAATGTGCTCACCCAGTCTC<br>CAGCAATCATGTCTGCATCTCTA<br>GGGGAGAAGGTCACCATGAGC<br>TGCAGGGCCAGCTCAAGTGTAA<br>ATTACATGTACTGGTACCAGCAG<br>AAGTCAGATGCCTCCCCCAAAC<br>TATGGATTTATTACACATCCAAC<br>CTGGCTCCTGGAGTCCCAGCT<br>CGCTTCAGTGGCAGTGGGTCTG<br>GGAACTCTTATTCTCTCACAATC<br>AGCAGCATGGAGGGTGAAGAT<br>GCTGCCACTTATTACTGCCAGC<br>AGTTTACTAGTTCCCCATCCA
IGKV4-50*01_S0901_B6	C57BL/6J	IGKV	MUSMUS IGKV4-50*01 F	100	GAAAATGTGCTCACCCAGTCTC<br>CAGCAATCATGTCTGCATCTCTA<br>GGGGAGAAGGTCACCATGAGC<br>TGCAGGGCCAGCTCAAGTGTAA<br>ATTACATGTACTGGTACCAGCAG<br>AAGTCAGATGCCTCCCCCAAAC<br>TATGGATTTATTACACATCCAAC<br>CTGGCTCCTGGAGTCCCAGCT<br>CGCTTCAGTGGCAGTGGGTCTG<br>GGAACTCTTATTCTCTCACAATC<br>AGCAGCATGGAGGGTGAAGAT<br>GCTGCCACTTATTACTGCCAGC<br>AGTTTACTAGTTCCCCATACA
IGKV4-50*01_S0901_C3H	C3H/HEJ	IGKV	MUSMUS IGKV4-50*01 F	100	GAAAATGTGCTCACCCAGTCTC<br>CAGCAATCATGTCTGCATCTCTA<br>GGGGAGAAGGTCACCATGAGC<br>TGCAGGGCCAGCTCAAGTGTAA<br>ATTACATGTACTGGTACCAGCAG<br>AAGTCAGATGCCTCCCCCAAAC<br>TATGGATTTATTACACATCCAAC<br>CTGGCTCCTGGAGTCCCAGCT<br>CGCTTCAGTGGCAGTGGGTCTG<br>GGAACTCTTATTCTCTCACAATC<br>AGCAGCATGGAGGGTGAAGAT<br>GCTGCCACTTATTACTGCCAGC<br>AGTTTACTAGTTCCCCATACA
IGKV4-50*01_S0901_DBA1	DBA/1J	IGKV	MUSMUS IGKV4-50*01 F	100	GAAAATGTGCTCACCCAGTCTC<br>CAGCAATCATGTCTGCATCTCTA<br>GGGGAGAAGGTCACCATGAGC<br>TGCAGGGCCAGCTCAAGTGTAA<br>ATTACATGTACTGGTACCAGCAG<br>AAGTCAGATGCCTCCCCCAAAC<br>TATGGATTTATTACACATCCAAC<br>CTGGCTCCTGGAGTCCCAGCT<br>CGCTTCAGTGGCAGTGGGTCTG<br>GGAACTCTTATTCTCTCACAATC<br>AGCAGCATGGAGGGTGAAGAT<br>GCTGCCACTTATTACTGCCAGC<br>AGTTTACTAGTTCCCCATACA

IGKV4-50*01_S0901_DBA2	DBA/2J	IGKV	MUSMUS IGKV4-50*01 F	100	GAAAATGTGCTCACCCAGTCTCCAGCAATCATGTCTGCATCTCTAGGGGAGAAGGTCACCATGAGCTGCAGGGCCAGCTCAAGTGTAAATTACATGTACTGGTACCAGCAGAAGTCAGATGCCTCCCCCAAACTATGGATTTATTACACATCCAACCTGGCTCCTGGAGTCCCAGCTCGCTTCAGTGGCAGTGGGTCTGGGAACTCTTATTCTCTCACAATCAGCAGCATGGAGGGTGAAGATGCTGCCACTTATTACTGCCAGCAGTTTACTAGTTCCCCATACA
IGKV4-50*01_S0901_LEWES	LEWES/EIJ	IGKV	MUSMUS IGKV4-50*01 F	100	GAAAATGTGCTCACCCAGTCTCCAGCAATCATGTCTGCATCTCTAGGGGAGAAGGTCACCATGAGCTGCAGGGCCAGCTCAAGTGTAAATTACATGTACTGGTACCAGCAGAAGTCAGATGCCTCCCCCAAACTATGGATTTATTACACATCCAACCTGGCTCCTGGAGTCCCAGCTCGCTTCAGTGGCAGTGGGTCTGGGAACTCTTATTCTCTCACAATCAGCAGCATGGAGGGTGAAGATGCTGCCACTTATTACTGCCAGCAGTTTACTAGTTCCCCATACA
IGKV4-50*01_S0901_NZB	NZB/BLNJ	IGKV	MUSMUS IGKV4-50*01 F	100	GAAAATGTGCTCACCCAGTCTCCAGCAATCATGTCTGCATCTCTAGGGGAGAAGGTCACCATGAGCTGCAGGGCCAGCTCAAGTGTAAATTACATGTACTGGTACCAGCAGAAGTCAGATGCCTCCCCCAAACTATGGATTTATTACACATCCAACCTGGCTCCTGGAGTCCCAGCTCGCTTCAGTGGCAGTGGGTCTGGGAACTCTTATTCTCTCACAATCAGCAGCATGGAGGGTGAAGATGCTGCCACTTATTACTGCCAGCAGTTTACTAGTTCCCCATACA
IGKV4-50*01_S0901_SJL	SJL/J	IGKV	MUSMUS IGKV4-50*01 F	100	GAAAATGTGCTCACCCAGTCTCCAGCAATCATGTCTGCATCTCTAGGGGAGAAGGTCACCATGAGCTGCAGGGCCAGCTCAAGTGTAAATTACATGTACTGGTACCAGCAGAAGTCAGATGCCTCCCCCAAACTATGGATTTATTACACATCCAACCTGGCTCCTGGAGTCCCAGCTCGCTTCAGTGGCAGTGGGTCTGGGAACTCTTATTCTCTCACAATCAGCAGCATGGAGGGTGAAGATGCTGCCACTTATTACTGCCAGCAGTTTACTAGTTCCCCATACA

IGKV4-50*01_S2188_AKR	AKR/J	IGKV	MUSMUS IGKV4-50*01 F	97.1	GAAAATGTGCTCACCCAGTCTCCAGCAATCATGTCTGCAACTCTAGGGGAGAAGGTCACCATGAGCTGCAGGGCCAGCTCAAATGTAAAGTACATGTACTGGTACCAGCAGAAGTCAGGTGCCTCCCCCAAACTATGGATTTATTACACATCCAACCTGGCTTCTGGAGTCCCAGCTCGCTTCAGTGGCAGTGGGTCTGGGACCTCTTATTCTCTCACAATCAGCAGCGTGGAGGCTGAAGATGCTGCCACTTATTACTGCCAGCAGTTTACTAGTTCCCCATCCA
IGKV4-50*01_S2188_MRL	MRL/MPJ	IGKV	MUSMUS IGKV4-50*01 F	97.1	GAAAATGTGCTCACCCAGTCTCCAGCAATCATGTCTGCAACTCTAGGGGAGAAGGTCACCATGAGCTGCAGGGCCAGCTCAAATGTAAAGTACATGTACTGGTACCAGCAGAAGTCAGGTGCCTCCCCCAAACTATGGATTTATTACACATCCAACCTGGCTTCTGGAGTCCCAGCTCGCTTCAGTGGCAGTGGGTCTGGGACCTCTTATTCTCTCACAATCAGCAGCGTGGAGGCTGAAGATGCTGCCACTTATTACTGCCAGCAGTTTACTAGTTCCCCATCCA
IGKV4-50*01_S2188_NOD	NOD/SHILTJ	IGKV	MUSMUS IGKV4-50*01 F	97.1	GAAAATGTGCTCACCCAGTCTCCAGCAATCATGTCTGCAACTCTAGGGGAGAAGGTCACCATGAGCTGCAGGGCCAGCTCAAATGTAAAGTACATGTACTGGTACCAGCAGAAGTCAGGTGCCTCCCCCAAACTATGGATTTATTACACATCCAACCTGGCTTCTGGAGTCCCAGCTCGCTTCAGTGGCAGTGGGTCTGGGACCTCTTATTCTCTCACAATCAGCAGCGTGGAGGCTGAAGATGCTGCCACTTATTACTGCCAGCAGTTTACTAGTTCCCCATCCA
IGKV4-50*01_S2188_NOR	NOR/LTJ	IGKV	MUSMUS IGKV4-50*01 F	97.1	GAAAATGTGCTCACCCAGTCTCCAGCAATCATGTCTGCAACTCTAGGGGAGAAGGTCACCATGAGCTGCAGGGCCAGCTCAAATGTAAAGTACATGTACTGGTACCAGCAGAAGTCAGGTGCCTCCCCCAAACTATGGATTTATTACACATCCAACCTGGCTTCTGGAGTCCCAGCTCGCTTCAGTGGCAGTGGGTCTGGGACCTCTTATTCTCTCACAATCAGCAGCGTGGAGGCTGAAGATGCTGCCACTTATTACTGCCAGCAGTTTACTAGTTCCCCATCCA

IGKV4-50*01_S2743_DBA1	DBA/1J	IGKV	MUSMUS IGKV4-50*01 F	100	GAAAATGTGCTCACCCAGTCTCCAGCAATCATGTCTGCATCTCTAGGGGAGAAGGTCACCATGAGCTGCAGGGCCAGCTCAAGTGTAAATTACATGTACTGGTACCAGCAGAAGTCAGATGCCTCCCCCAAACTATGGATTTATTACACATCCAACCTGGCTCCTGGAGTCCCAGCTCGCTTCAGTGGCAGTGGGTCTGGGAACTCTTATTCTCTCACAATCAGCAGCATGGAGGGTGAAGATGCTGCCACTTATTACTGCCAGCAGTTTACTAGTTCCCCATTCA
IGKV4-50*01_S4730_MSM	MSM/MSJ	IGKV	MUSMUS IGKV4-50*01 F	97.46	GAAAATGTGCTCACCCAGTCTCCAGCACTCATGTCTGCATCTCCAGGGGAGAAGGTCACCATGAGCTGCAGGGCCAGCTCAAGTGTAAATTACATGTACTGGTACCAGCAGAAGTCAGGTGCCTCCCCCAAACTATGGATTTATTACACATCCAACCTGGCTCCTGGAATCCCAGCTCGCTTCAGTGGCAGTGGGTCTGGGACCTCTTATTCTCTCACAATCAGCAGCGTGGAGGCTGAAGATGCTGCCACTTATTACTGCCAGCAGTTTACTAGTTCCCCATCCA
IGKV4-50*01_S4988_MSM	MSM/MSJ	IGKV	MUSMUS IGKV4-50*01 F	96.74	GAAAATGTGCTCACCCAGTCTCCAGCAATCATGTCTGCAACTCTAGGGGAGAAGGTCACCATGAGCTGCAGGGCCAGCTCAAATGTAAAGAACATGTACTGGTACCAGCAGAAGTCAGGTGCCTCCCCCAAACTATGGATTTATTACACATCCAACCTGGCTTCTGGAGTCCCAGCTCGCTTCAGTGGCAGTGGGTCTGGGACCTCTTATTCTCTCACAATCAGCAGCGTGGAGGCTGAAGATGCTGCCACTTATTACTGCCAGCAGTTTACTAGTTCCCCATACA
IGKV4-50*01_S6254_CAST	CAST/EIJ	IGKV	MUSMUS IGKV4-50*01 F	97.83	GAAAATGTGCTCACCCAGTCTCCAGCAATCATGTCTGCATCTCTAGGGGAGAAGGTCACCATGAGCTGCAGGGCCAGCTCAAGAGTAAATTACATGTACTGGTACCAGCAGAAGTCAGGTGCCTCCCCCAAACTATGGATTTATTACACATCCAACCTGGCTCCTGGAGTCCCAGCTCGCTTCAGTGGCAGTGGGTCTGGGACCTCTTATTCTCTCACAATCAGCAGCGTGGAGGCTGAAGATGCTGCCACTTATTACTGCCAGCAGTTAACTAGTTCCCCATCCA

IGKV4-50*01_S6568_MSM	MSM/MSJ	IGKV	MUSMUS IGKV4-50*01 F	96.74	GAAAATGTGCTCACCCAGTCTC<br>CAGCAATCATGTCTGCAACTCTA<br>GGGGAGAAGGTCACCATGAGC<br>TGCAGGGCCAGCTCAAATGTAA<br>AGAACATGTACTGGTACCAGCA<br>GAAGTCAGGTGCCTCCCCCAA<br>ACTATGGATTTATTACACATCCA<br>ACCTGGCTTCTGGAGTCCCAGC<br>TCGCTTCAGTGGCAGTGGGTCT<br>GGGACCTCTTATTCTCTCACAAT<br>CAGCAGCGTGGAGGCTGAAGA<br>TGCTGCCACTTATTACTGCCAG<br>CAGTTTACTAGTTCCCCATCCA
IGKV4-50*01_S7633_PWD	PWD/PHJ	IGKV	MUSMUS IGKV4-50*01 F	96.74	GAAAATGTGCTCACCCAGTCTC<br>CAGCAATCATGTCTGCAACTCTA<br>GGGGAGAAGGTCACCATGAGC<br>TGCAGGGCCAGCTCAAATGTAA<br>AGTACATGTACTGGTACCAGCA<br>GAAGTCAGGTGCCTCCCCCAA<br>ACTATGGATTTATTACACATTCAA<br>CCTGGCTTCTGGAGTCCCAGCT<br>CGCTTCAGTGGCAGTGGGTCTG<br>GGACCTCTTATTCTCTCACAATC<br>AGCAGCGTGGAGGCTGAAGAT<br>GCTGCCACTTATTACTGCCAGC<br>AGTTTACTAGTTCCCCATCCA
IGKV4-50*01_S8668_129	129S1/SVIMJ	IGKV	MUSMUS IGKV4-50*01 F	100	GAAAATGTGCTCACCCAGTCTC<br>CAGCAATCATGTCTGCATCTCTA<br>GGGGAGAAGGTCACCATGAGC<br>TGCAGGGCCAGCTCAAGTGTAA<br>ATTACATGTACTGGTACCAGCAG<br>AAGTCAGATGCCTCCCCCAAAC<br>TATGGATTTATTACACATCCAAC<br>CTGGCTCCTGGAGTCCCAGCT<br>CGCTTCAGTGGCAGTGGGTCTG<br>GGAACTCTTATTCTCTCACAATC<br>AGCAGCATGGAGGGTGAAGAT<br>GCTGCCACTTATTACTGCCAGC<br>AGTTTACTAGTTCCCCGTACA
IGKV4-51*01_129	129S1/SVIMJ	IGKV	MUSMUS IGKV4-51*01 F	100	GAAAATGTGCTCACCCAGTCTC<br>CAGCAATAATGGCTGCCTCTCT<br>GGGGGAGAAGGTCACCATGAC<br>CTGCAGTGCCAGCTCAAGTGTA<br>AGTTCCAGCTACTTGCACTGGTA<br>CCAGCAGAAGTCAGGCACTTC<br>CCCCAAACTCTGGATTTATGGC<br>ACATCCAACCTGGCTTCTGGAG<br>TCCCAGCTCGCTTCAGTGGCAG<br>TGGGGCTGGGATCTCTTACTCT<br>CTCACAATCAGCAGCATGGAGG<br>CTGAAAATGATGCAACTTATTAC<br>TGCCAGCAGTGGAGTGGTTACC<br>CATTCA

IGKV4-51*01_B6	C57BL/6J	IGKV	MUSMUS IGKV4-51*01 F	100	GAAAATGTGCTCACCCAGTCTC<br>CAGCAATAATGGCTGCCTCTCT<br>GGGGGAGAAGGTCACCATGAC<br>CTGCAGTGCCAGCTCAAGTGTA<br>AGTTCCAGCTACTTGCACTGGTA<br>CCAGCAGAAGTCAGGCACTTC<br>CCCCAAACTCTGGATTTATGGC<br>ACATCCAACCTGGCTTCTGGAG<br>TCCCAGCTCGCTTCAGTGGCAG<br>TGGGGCTGGGATCTCTTACTCT<br>CTCACAATCAGCAGCATGGAGG<br>CTGAAAATGATGCAACTTATTAC<br>TGCCAGCAGTGGAGTGGTTACC<br>CATTCA
IGKV4-51*01_BALB	BALB/CBYJ	IGKV	MUSMUS IGKV4-51*01 F	100	GAAAATGTGCTCACCCAGTCTC<br>CAGCAATAATGGCTGCCTCTCT<br>GGGGGAGAAGGTCACCATGAC<br>CTGCAGTGCCAGCTCAAGTGTA<br>AGTTCCAGCTACTTGCACTGGTA<br>CCAGCAGAAGTCAGGCACTTC<br>CCCCAAACTCTGGATTTATGGC<br>ACATCCAACCTGGCTTCTGGAG<br>TCCCAGCTCGCTTCAGTGGCAG<br>TGGGGCTGGGATCTCTTACTCT<br>CTCACAATCAGCAGCATGGAGG<br>CTGAAAATGATGCAACTTATTAC<br>TGCCAGCAGTGGAGTGGTTACC<br>CATTCA
IGKV4-51*01_C3H	C3H/HEJ	IGKV	MUSMUS IGKV4-51*01 F	100	GAAAATGTGCTCACCCAGTCTC<br>CAGCAATAATGGCTGCCTCTCT<br>GGGGGAGAAGGTCACCATGAC<br>CTGCAGTGCCAGCTCAAGTGTA<br>AGTTCCAGCTACTTGCACTGGTA<br>CCAGCAGAAGTCAGGCACTTC<br>CCCCAAACTCTGGATTTATGGC<br>ACATCCAACCTGGCTTCTGGAG<br>TCCCAGCTCGCTTCAGTGGCAG<br>TGGGGCTGGGATCTCTTACTCT<br>CTCACAATCAGCAGCATGGAGG<br>CTGAAAATGATGCAACTTATTAC<br>TGCCAGCAGTGGAGTGGTTACC<br>CATTCA
IGKV4-51*01_CBA	CBA/J	IGKV	MUSMUS IGKV4-51*01 F	100	GAAAATGTGCTCACCCAGTCTC<br>CAGCAATAATGGCTGCCTCTCT<br>GGGGGAGAAGGTCACCATGAC<br>CTGCAGTGCCAGCTCAAGTGTA<br>AGTTCCAGCTACTTGCACTGGTA<br>CCAGCAGAAGTCAGGCACTTC<br>CCCCAAACTCTGGATTTATGGC<br>ACATCCAACCTGGCTTCTGGAG<br>TCCCAGCTCGCTTCAGTGGCAG<br>TGGGGCTGGGATCTCTTACTCT<br>CTCACAATCAGCAGCATGGAGG<br>CTGAAAATGATGCAACTTATTAC<br>TGCCAGCAGTGGAGTGGTTACC<br>CATTCA

IGKV4-51*01_DBA1	DBA/1J	IGKV	MUSMUS IGKV4-51*01 F	100	GAAAATGTGCTCACCCAGTCTC<br>CAGCAATAATGGCTGCCTCTCT<br>GGGGGAGAAGGTCACCATGAC<br>CTGCAGTGCCAGCTCAAGTGTA<br>AGTTCCAGCTACTTGCACTGGTA<br>CCAGCAGAAGTCAGGCACTTC<br>CCCCAAACTCTGGATTTATGGC<br>ACATCCAACCTGGCTTCTGGAG<br>TCCCAGCTCGCTTCAGTGGCAG<br>TGGGGCTGGGATCTCTTACTCT<br>CTCACAATCAGCAGCATGGAGG<br>CTGAAAATGATGCAACTTATTAC<br>TGCCAGCAGTGGAGTGGTTACC<br>CATTCA
IGKV4-51*01_LEWES	LEWES/EIJ	IGKV	MUSMUS IGKV4-51*01 F	100	GAAAATGTGCTCACCCAGTCTC<br>CAGCAATAATGGCTGCCTCTCT<br>GGGGGAGAAGGTCACCATGAC<br>CTGCAGTGCCAGCTCAAGTGTA<br>AGTTCCAGCTACTTGCACTGGTA<br>CCAGCAGAAGTCAGGCACTTC<br>CCCCAAACTCTGGATTTATGGC<br>ACATCCAACCTGGCTTCTGGAG<br>TCCCAGCTCGCTTCAGTGGCAG<br>TGGGGCTGGGATCTCTTACTCT<br>CTCACAATCAGCAGCATGGAGG<br>CTGAAAATGATGCAACTTATTAC<br>TGCCAGCAGTGGAGTGGTTACC<br>CATTCA
IGKV4-51*01_S0136_AJ	A/J	IGKV	MUSMUS IGKV4-51*01 F	100	GAAAATGTGCTCACCCAGTCTC<br>CAGCAATAATGGCTGCCTCTCT<br>GGGGGAGAAGGTCACCATGAC<br>CTGCAGTGCCAGCTCAAGTGTA<br>AGTTCCAGCTACTTGCACTGGTA<br>CCAGCAGAAGTCAGGCACTTC<br>CCCCAAACTCTGGATTTATGGC<br>ACATCCAACCTGGCTTCTGGAG<br>TCCCAGCTCGCTTCAGTGGCAG<br>TGGGGCTGGGATCTCTTACTCT<br>CTCACAATCAGCAGCATGGAGG<br>CTGAAAATGATGCAACTTATTAC<br>TGCCAGCAGTGGAGTGGTTACC<br>CGTACA
IGKV4-51*01_S4777_DBA2	DBA/2J	IGKV	MUSMUS IGKV4-51*01 F	100	GAAAATGTGCTCACCCAGTCTC<br>CAGCAATAATGGCTGCCTCTCT<br>GGGGGAGAAGGTCACCATGAC<br>CTGCAGTGCCAGCTCAAGTGTA<br>AGTTCCAGCTACTTGCACTGGTA<br>CCAGCAGAAGTCAGGCACTTC<br>CCCCAAACTCTGGATTTATGGC<br>ACATCCAACCTGGCTTCTGGAG<br>TCCCAGCTCGCTTCAGTGGCAG<br>TGGGGCTGGGATCTCTTACTCT<br>CTCACAATCAGCAGCATGGAGG<br>CTGAAAATGATGCAACTTATTAC<br>TGCCAGCAGTGGAGTGGTTACC<br>CATACA

IGKV4-51*01_S7391_MSM	MSM/MSJ	IGKV	MUSMUS IGKV4-51*01 F, OR MUSMUS IGKV4-57-1*01 F	92.55	GAAAATGTGCTCACCCAGTCTCCAGCACTCATGGCTGCATCTCCAGGGGAGAAGGTCACCATGACCTGCAGTGCCAGCTCAAGTGTAAGTTCTAGCTACTTGCACTGGTACCAGCAGAAGTCAGGTGCCTCCCCCAAACTCTGGATTTACCGCACATCCAACCTGGCTTCCGGAGTCCCAGCTCGCTTCAGTGGCAGTGGGTCTGGGACCTCTTACTCTCTCACAATCAGCAACGTGGAGGCTGAAGATGATGCAACTTATTACTGCCAGCAGGGGTGGGATTACCCATTCA
IGKV4-51*01_S7391_PWD	PWD/PHJ	IGKV	MUSMUS IGKV4-51*01 F, OR MUSMUS IGKV4-57-1*01 F	92.55	GAAAATGTGCTCACCCAGTCTCCAGCACTCATGGCTGCATCTCCAGGGGAGAAGGTCACCATGACCTGCAGTGCCAGCTCAAGTGTAAGTTCTAGCTACTTGCACTGGTACCAGCAGAAGTCAGGTGCCTCCCCCAAACTCTGGATTTACCGCACATCCAACCTGGCTTCCGGAGTCCCAGCTCGCTTCAGTGGCAGTGGGTCTGGGACCTCTTACTCTCTCACAATCAGCAACGTGGAGGCTGAAGATGATGCAACTTATTACTGCCAGCAGGGGTGGGATTACCCATTCA
IGKV4-52*01_AJ	A/J	IGKV	MUSMUS IGKV4-52*01 ORF, OR MUSMUS IGKV4-54*01 ORF	100	CAAATTGTTCTCACCCAGTCTCCAGCAATCCTGTCTGCATCTCCAGGGGAGAAGGTCACCATGACCTGCAGTGCCAGCTCAAGTGTAAGTTACATGTACAGGTACCAGCAGAAGCCAGGATCCTCACCCAAACCCTGGATTTATGGCACATCCAACCTGGCTTCTGGAGTCCCTGCTCGCTTCAGTGGCAGTGGATCTGGGACCTCTTATTCTCTCACAATCAGCAGCATGGAGGCTGAAGATGCTGCCACTTATTACTGCCAGCAATATCATAGTTACCCACCCA
IGKV4-52*01_BALB	BALB/CBYJ	IGKV	MUSMUS IGKV4-52*01 ORF, OR MUSMUS IGKV4-54*01 ORF	100	CAAATTGTTCTCACCCAGTCTCCAGCAATCCTGTCTGCATCTCCAGGGGAGAAGGTCACCATGACCTGCAGTGCCAGCTCAAGTGTAAGTTACATGTACAGGTACCAGCAGAAGCCAGGATCCTCACCCAAACCCTGGATTTATGGCACATCCAACCTGGCTTCTGGAGTCCCTGCTCGCTTCAGTGGCAGTGGATCTGGGACCTCTTATTCTCTCACAATCAGCAGCATGGAGGCTGAAGATGCTGCCACTTATTACTGCCAGCAATATCATAGTTACCCACCCA

IGKV4-52*01_DBA2	DBA/2J	IGKV	MUSMUS IGKV4-52*01 ORF, OR MUSMUS IGKV4-54*01 ORF	100	CAAATTGTTCTCACCCAGTCTCC AGCAATCCTGTCTGCATCTCCA GGGGAGAAGGTCACCATGACC TGCAGTGCCAGCTCAAGTGTAA GTTACATGTACAGGTACCAGCA GAAGCCAGGATCCTCACCCAA ACCCTGGATTTATGGCACATCC AACCTGGCTTCTGGAGTCCCTG CTCGCTTCAGTGGCAGTGGATC TGGGACCTCTTATTCTCTCACAA TCAGCAGCATGGAGGCTGAAGA TGCTGCCACTTATTACTGCCAG CAATATCATAGTTACCCACCCA
IGKV4-52*01_NZB	NZB/BLNJ	IGKV	MUSMUS IGKV4-52*01 ORF, OR MUSMUS IGKV4-54*01 ORF	100	CAAATTGTTCTCACCCAGTCTCC AGCAATCCTGTCTGCATCTCCA GGGGAGAAGGTCACCATGACC TGCAGTGCCAGCTCAAGTGTAA GTTACATGTACAGGTACCAGCA GAAGCCAGGATCCTCACCCAA ACCCTGGATTTATGGCACATCC AACCTGGCTTCTGGAGTCCCTG CTCGCTTCAGTGGCAGTGGATC TGGGACCTCTTATTCTCTCACAA TCAGCAGCATGGAGGCTGAAGA TGCTGCCACTTATTACTGCCAG CAATATCATAGTTACCCACCCA
IGKV4-52*01_S2975_B6	C57BL/6J	IGKV	MUSMUS IGKV4-52*01 ORF, OR MUSMUS IGKV4-54*01 ORF	100	CAAATTGTTCTCACCCAGTCTCC AGCAATCCTGTCTGCATCTCCA GGGGAGAAGGTCACCATGACC TGCAGTGCCAGCTCAAGTGTAA GTTACATGTACAGGTACCAGCA GAAGCCAGGATCCTCACCCAA ACCCTGGATTTATGGCACATCC AACCTGGCTTCTGGAGTCCCTG CTCGCTTCAGTGGCAGTGGATC TGGGACCTCTTATTCTCTCACAA TCAGCAGCATGGAGGCTGAAGA TGCTGCCACTTATTACTGCCAG CAATATCATAGTTACCCGCTCA
IGKV4-52*01_S2975_DBA1	DBA/1J	IGKV	MUSMUS IGKV4-52*01 ORF, OR MUSMUS IGKV4-54*01 ORF	100	CAAATTGTTCTCACCCAGTCTCC AGCAATCCTGTCTGCATCTCCA GGGGAGAAGGTCACCATGACC TGCAGTGCCAGCTCAAGTGTAA GTTACATGTACAGGTACCAGCA GAAGCCAGGATCCTCACCCAA ACCCTGGATTTATGGCACATCC AACCTGGCTTCTGGAGTCCCTG CTCGCTTCAGTGGCAGTGGATC TGGGACCTCTTATTCTCTCACAA TCAGCAGCATGGAGGCTGAAGA TGCTGCCACTTATTACTGCCAG CAATATCATAGTTACCCGCTCA

IGKV4-52*01_S5019_129	129S1/SVIMJ	IGKV	MUSMUS IGKV4-52*01 ORF, OR MUSMUS IGKV4-54*01 ORF	100	CAAATTGTTCTCACCCAGTCTCCAGCAATCCTGTCTGCATCTCCAGGGGAGAAGGTCACCATGACCTGCAGTGCCAGCTCAAGTGTAAGTTACATGTACAGGTACCAGCAGAAGCCAGGATCCTCACCCAAACCCTGGATTTATGGCACATCCAACCTGGCTTCTGGAGTCCCTGCTCGCTTCAGTGGCAGTGGATCTGGGACCTCTTATTCTCTCACAATCAGCAGCATGGAGGCTGAAGATGCTGCCACTTATTACTGCCAGCAATATCATAGTTACCCACTCA
IGKV4-52*01_S5019_AJ	A/J	IGKV	MUSMUS IGKV4-52*01 ORF, OR MUSMUS IGKV4-54*01 ORF	100	CAAATTGTTCTCACCCAGTCTCCAGCAATCCTGTCTGCATCTCCAGGGGAGAAGGTCACCATGACCTGCAGTGCCAGCTCAAGTGTAAGTTACATGTACAGGTACCAGCAGAAGCCAGGATCCTCACCCAAACCCTGGATTTATGGCACATCCAACCTGGCTTCTGGAGTCCCTGCTCGCTTCAGTGGCAGTGGATCTGGGACCTCTTATTCTCTCACAATCAGCAGCATGGAGGCTGAAGATGCTGCCACTTATTACTGCCAGCAATATCATAGTTACCCACTCA
IGKV4-52*01_S5019_C3H	C3H/HEJ	IGKV	MUSMUS IGKV4-52*01 ORF, OR MUSMUS IGKV4-54*01 ORF	100	CAAATTGTTCTCACCCAGTCTCCAGCAATCCTGTCTGCATCTCCAGGGGAGAAGGTCACCATGACCTGCAGTGCCAGCTCAAGTGTAAGTTACATGTACAGGTACCAGCAGAAGCCAGGATCCTCACCCAAACCCTGGATTTATGGCACATCCAACCTGGCTTCTGGAGTCCCTGCTCGCTTCAGTGGCAGTGGATCTGGGACCTCTTATTCTCTCACAATCAGCAGCATGGAGGCTGAAGATGCTGCCACTTATTACTGCCAGCAATATCATAGTTACCCACTCA
IGKV4-52*01_S5019_DBA1	DBA/1J	IGKV	MUSMUS IGKV4-52*01 ORF, OR MUSMUS IGKV4-54*01 ORF	100	CAAATTGTTCTCACCCAGTCTCCAGCAATCCTGTCTGCATCTCCAGGGGAGAAGGTCACCATGACCTGCAGTGCCAGCTCAAGTGTAAGTTACATGTACAGGTACCAGCAGAAGCCAGGATCCTCACCCAAACCCTGGATTTATGGCACATCCAACCTGGCTTCTGGAGTCCCTGCTCGCTTCAGTGGCAGTGGATCTGGGACCTCTTATTCTCTCACAATCAGCAGCATGGAGGCTGAAGATGCTGCCACTTATTACTGCCAGCAATATCATAGTTACCCACTCA

IGKV4-52*01_S5019_DBA2	DBA/2J	IGKV	MUSMUS IGKV4-52*01 ORF, OR MUSMUS IGKV4-54*01 ORF	100	CAAATTGTTCTCACCCAGTCTCCAGCAATCCTGTCTGCATCTCCAGGGGAGAAGGTCACCATGACCTGCAGTGCCAGCTCAAGTGTAAGTTACATGTACAGGTACCAGCAGAAGCCAGGATCCTCACCCAAACCCTGGATTTATGGCACATCCAACCTGGCTTCTGGAGTCCCTGCTCGCTTCAGTGGCAGTGGATCTGGGACCTCTTATTCTCTCACAATCAGCAGCATGGAGGCTGAAGATGCTGCCACTTATTACTGCCAGCAATATCATAGTTACCCACTCA
IGKV4-52*01_S5019_NZB	NZB/BLNJ	IGKV	MUSMUS IGKV4-52*01 ORF, OR MUSMUS IGKV4-54*01 ORF	100	CAAATTGTTCTCACCCAGTCTCCAGCAATCCTGTCTGCATCTCCAGGGGAGAAGGTCACCATGACCTGCAGTGCCAGCTCAAGTGTAAGTTACATGTACAGGTACCAGCAGAAGCCAGGATCCTCACCCAAACCCTGGATTTATGGCACATCCAACCTGGCTTCTGGAGTCCCTGCTCGCTTCAGTGGCAGTGGATCTGGGACCTCTTATTCTCTCACAATCAGCAGCATGGAGGCTGAAGATGCTGCCACTTATTACTGCCAGCAATATCATAGTTACCCACTCA
IGKV4-52*01_S5019_SJL	SJL/J	IGKV	MUSMUS IGKV4-52*01 ORF, OR MUSMUS IGKV4-54*01 ORF	100	CAAATTGTTCTCACCCAGTCTCCAGCAATCCTGTCTGCATCTCCAGGGGAGAAGGTCACCATGACCTGCAGTGCCAGCTCAAGTGTAAGTTACATGTACAGGTACCAGCAGAAGCCAGGATCCTCACCCAAACCCTGGATTTATGGCACATCCAACCTGGCTTCTGGAGTCCCTGCTCGCTTCAGTGGCAGTGGATCTGGGACCTCTTATTCTCTCACAATCAGCAGCATGGAGGCTGAAGATGCTGCCACTTATTACTGCCAGCAATATCATAGTTACCCACTCA
IGKV4-52*01_SJL	SJL/J	IGKV	MUSMUS IGKV4-52*01 ORF, OR MUSMUS IGKV4-54*01 ORF	100	CAAATTGTTCTCACCCAGTCTCCAGCAATCCTGTCTGCATCTCCAGGGGAGAAGGTCACCATGACCTGCAGTGCCAGCTCAAGTGTAAGTTACATGTACAGGTACCAGCAGAAGCCAGGATCCTCACCCAAACCCTGGATTTATGGCACATCCAACCTGGCTTCTGGAGTCCCTGCTCGCTTCAGTGGCAGTGGATCTGGGACCTCTTATTCTCTCACAATCAGCAGCATGGAGGCTGAAGATGCTGCCACTTATTACTGCCAGCAATATCATAGTTACCCACCCA

IGKV4-53*01_129	129S1/SVIMJ	IGKV	MUSMUS IGKV4-53*01 F	100	GAAATTGTGCTCACCCAGTCTCCAGCACTCATGGCTGCATCTCCAGGGGAGAAGGTCACCATCACCTGCAGTGTCAGCTCAAGTATAAGTTCCAGCAACTTGCACTGGTACCAGCAGAAGTCAGAAACCTCCCCCAAACCCTGGATTTATGGCACATCCAACCTGGCTTCTGGAGTCCCTGTTCGCTTCAGTGGCAGTGGATCTGGGACCTCTTATTCTCTCACAATCAGCAGCATGGAGGCTGAAGATGCTGCCACTTATTACTGTCAACAGTGGAGTAGTTACCCACTCA
IGKV4-53*01_AJ	A/J	IGKV	MUSMUS IGKV4-53*01 F	100	GAAATTGTGCTCACCCAGTCTCCAGCACTCATGGCTGCATCTCCAGGGGAGAAGGTCACCATCACCTGCAGTGTCAGCTCAAGTATAAGTTCCAGCAACTTGCACTGGTACCAGCAGAAGTCAGAAACCTCCCCCAAACCCTGGATTTATGGCACATCCAACCTGGCTTCTGGAGTCCCTGTTCGCTTCAGTGGCAGTGGATCTGGGACCTCTTATTCTCTCACAATCAGCAGCATGGAGGCTGAAGATGCTGCCACTTATTACTGTCAACAGTGGAGTAGTTACCCACTCA
IGKV4-53*01_B6	C57BL/6J	IGKV	MUSMUS IGKV4-53*01 F	100	GAAATTGTGCTCACCCAGTCTCCAGCACTCATGGCTGCATCTCCAGGGGAGAAGGTCACCATCACCTGCAGTGTCAGCTCAAGTATAAGTTCCAGCAACTTGCACTGGTACCAGCAGAAGTCAGAAACCTCCCCCAAACCCTGGATTTATGGCACATCCAACCTGGCTTCTGGAGTCCCTGTTCGCTTCAGTGGCAGTGGATCTGGGACCTCTTATTCTCTCACAATCAGCAGCATGGAGGCTGAAGATGCTGCCACTTATTACTGTCAACAGTGGAGTAGTTACCCACTCA
IGKV4-53*01_BALB	BALB/CBYJ	IGKV	MUSMUS IGKV4-53*01 F	100	GAAATTGTGCTCACCCAGTCTCCAGCACTCATGGCTGCATCTCCAGGGGAGAAGGTCACCATCACCTGCAGTGTCAGCTCAAGTATAAGTTCCAGCAACTTGCACTGGTACCAGCAGAAGTCAGAAACCTCCCCCAAACCCTGGATTTATGGCACATCCAACCTGGCTTCTGGAGTCCCTGTTCGCTTCAGTGGCAGTGGATCTGGGACCTCTTATTCTCTCACAATCAGCAGCATGGAGGCTGAAGATGCTGCCACTTATTACTGTCAACAGTGGAGTAGTTACCCACTCA

IGKV4-53*01_C3H	C3H/HEJ	IGKV	MUSMUS IGKV4-53*01 F	100	GAAATTGTGCTCACCCAGTCTCCAGCACTCATGGCTGCATCTCCAGGGGAGAAGGTCACCATCACCTGCAGTGTCAGCTCAAGTATAAGTTCCAGCAACTTGCACTGGTACCAGCAGAAGTCAGAAACCTCCCCCAAACCCTGGATTTATGGCACATCCAACCTGGCTTCTGGAGTCCCTGTTCGCTTCAGTGGCAGTGGATCTGGGACCTCTTATTCTCTCACAATCAGCAGCATGGAGGCTGAAGATGCTGCCACTTATTACTGTCAACAGTGGAGTAGTTACCCACTCA
IGKV4-53*01_CBA	CBA/J	IGKV	MUSMUS IGKV4-53*01 F	100	GAAATTGTGCTCACCCAGTCTCCAGCACTCATGGCTGCATCTCCAGGGGAGAAGGTCACCATCACCTGCAGTGTCAGCTCAAGTATAAGTTCCAGCAACTTGCACTGGTACCAGCAGAAGTCAGAAACCTCCCCCAAACCCTGGATTTATGGCACATCCAACCTGGCTTCTGGAGTCCCTGTTCGCTTCAGTGGCAGTGGATCTGGGACCTCTTATTCTCTCACAATCAGCAGCATGGAGGCTGAAGATGCTGCCACTTATTACTGTCAACAGTGGAGTAGTTACCCACTCA
IGKV4-53*01_DBA1	DBA/1J	IGKV	MUSMUS IGKV4-53*01 F	100	GAAATTGTGCTCACCCAGTCTCCAGCACTCATGGCTGCATCTCCAGGGGAGAAGGTCACCATCACCTGCAGTGTCAGCTCAAGTATAAGTTCCAGCAACTTGCACTGGTACCAGCAGAAGTCAGAAACCTCCCCCAAACCCTGGATTTATGGCACATCCAACCTGGCTTCTGGAGTCCCTGTTCGCTTCAGTGGCAGTGGATCTGGGACCTCTTATTCTCTCACAATCAGCAGCATGGAGGCTGAAGATGCTGCCACTTATTACTGTCAACAGTGGAGTAGTTACCCACTCA
IGKV4-53*01_DBA2	DBA/2J	IGKV	MUSMUS IGKV4-53*01 F	100	GAAATTGTGCTCACCCAGTCTCCAGCACTCATGGCTGCATCTCCAGGGGAGAAGGTCACCATCACCTGCAGTGTCAGCTCAAGTATAAGTTCCAGCAACTTGCACTGGTACCAGCAGAAGTCAGAAACCTCCCCCAAACCCTGGATTTATGGCACATCCAACCTGGCTTCTGGAGTCCCTGTTCGCTTCAGTGGCAGTGGATCTGGGACCTCTTATTCTCTCACAATCAGCAGCATGGAGGCTGAAGATGCTGCCACTTATTACTGTCAACAGTGGAGTAGTTACCCACTCA

IGKV4-53*01_LEWES	LEWES/EIJ	IGKV	MUSMUS IGKV4-53*01 F	100	GAAATTGTGCTCACCCAGTCTC<br>CAGCACTCATGGCTGCATCTCC<br>AGGGGAGAAGGTCACCATCAC<br>CTGCAGTGTCAGCTCAAGTATAA<br>GTTCCAGCAACTTGCACTGGTA<br>CCAGCAGAAGTCAGAAACCTC<br>CCCCAAACCCTGGATTTATGGC<br>ACATCCAACCTGGCTTCTGGAG<br>TCCCTGTTCGCTTCAGTGGCAG<br>TGGATCTGGGACCTCTTATTCTC<br>TCACAATCAGCAGCATGGAGGC<br>TGAAGATGCTGCCACTTATTACT<br>GTCAACAGTGGAGTAGTTACCC<br>ACTCA
IGKV4-53*01_NZB	NZB/BLNJ	IGKV	MUSMUS IGKV4-53*01 F	100	GAAATTGTGCTCACCCAGTCTC<br>CAGCACTCATGGCTGCATCTCC<br>AGGGGAGAAGGTCACCATCAC<br>CTGCAGTGTCAGCTCAAGTATAA<br>GTTCCAGCAACTTGCACTGGTA<br>CCAGCAGAAGTCAGAAACCTC<br>CCCCAAACCCTGGATTTATGGC<br>ACATCCAACCTGGCTTCTGGAG<br>TCCCTGTTCGCTTCAGTGGCAG<br>TGGATCTGGGACCTCTTATTCTC<br>TCACAATCAGCAGCATGGAGGC<br>TGAAGATGCTGCCACTTATTACT<br>GTCAACAGTGGAGTAGTTACCC<br>ACTCA
IGKV4-53*01_S1762_CAST	CAST/EIJ	IGKV	MUSMUS IGKV4-53*01 F	99.65	GAAATTGTGCTCACCCAGTCTC<br>CAGCACTCATGGCTGCATCTCC<br>AGGGGAGAAGGTCACCATCAC<br>CTGCAGTGTCAGCTCAAGTATAA<br>GTTCCAGCAACTTGCACTGGTA<br>CCAGCAGAAGTCAGGAACCTC<br>CCCCAAACCCTGGATTTATGGC<br>ACATCCAACCTGGCTTCTGGAG<br>TCCCTGTTCGCTTCAGTGGCAG<br>TGGATCTGGGACCTCTTATTCTC<br>TCACAATCAGCAGCATGGAGGC<br>TGAAGATGCTGCCACTTATTACT<br>GTCAACAGTGGAGTAGTTACCC<br>ACTCA
IGKV4-53*01_S3575_AKR	AKR/J	IGKV	MUSMUS IGKV4-53*01 F	98.94	GAAATTGTGCTCACCCAGTCTC<br>CAGCACTCATGGCTGCATCTCC<br>AGGGGAGAAGGTCACCATCAC<br>CTGCAGTGTCAGCTCAAGTATAA<br>GTTCCAGCAACTTACACTGGTA<br>CCAGCAGAAGTCAGGAACCTC<br>CCCCAAACCCTGGATTTATGGC<br>ACATCCAACCTTGCTTCTGGAGT<br>CCCTGTTCGCTTCAGTGGCAGT<br>GGATCTGGGACCTCTTATTCTCT<br>CACAATCAGCAGCATGGAGGCT<br>GAAGATGCTGCCACTTATTACTG<br>TCAACAGTGGAGTAGTTACCCA<br>CTCA

IGKV4-53*01_S3575_MRL	MRL/MPJ	IGKV	MUSMUS IGKV4-53*01 F	98.94	GAAATTGTGCTCACCCAGTCTCCAGCACTCATGGCTGCATCTCCAGGGGAGAAGGTCACCATCACCTGCAGTGTCAGCTCAAGTATAAGTTCCAGCAACTTACACTGGTACCAGCAGAAGTCAGGAACCTCCCCCAAACCCTGGATTTATGGCACATCCAACCTTGCTTCTGGAGTCCCTGTTCGCTTCAGTGGCAGTGGATCTGGGACCTCTTATTCTCTCACAATCAGCAGCATGGAGGCTGAAGATGCTGCCACTTATTACTGTCAACAGTGGAGTAGTTACCCACTCA
IGKV4-53*01_S3575_NOD	NOD/SHILTJ	IGKV	MUSMUS IGKV4-53*01 F	98.94	GAAATTGTGCTCACCCAGTCTCCAGCACTCATGGCTGCATCTCCAGGGGAGAAGGTCACCATCACCTGCAGTGTCAGCTCAAGTATAAGTTCCAGCAACTTACACTGGTACCAGCAGAAGTCAGGAACCTCCCCCAAACCCTGGATTTATGGCACATCCAACCTTGCTTCTGGAGTCCCTGTTCGCTTCAGTGGCAGTGGATCTGGGACCTCTTATTCTCTCACAATCAGCAGCATGGAGGCTGAAGATGCTGCCACTTATTACTGTCAACAGTGGAGTAGTTACCCACTCA
IGKV4-53*01_S3575_NOR	NOR/LTJ	IGKV	MUSMUS IGKV4-53*01 F	98.94	GAAATTGTGCTCACCCAGTCTCCAGCACTCATGGCTGCATCTCCAGGGGAGAAGGTCACCATCACCTGCAGTGTCAGCTCAAGTATAAGTTCCAGCAACTTACACTGGTACCAGCAGAAGTCAGGAACCTCCCCCAAACCCTGGATTTATGGCACATCCAACCTTGCTTCTGGAGTCCCTGTTCGCTTCAGTGGCAGTGGATCTGGGACCTCTTATTCTCTCACAATCAGCAGCATGGAGGCTGAAGATGCTGCCACTTATTACTGTCAACAGTGGAGTAGTTACCCACTCA
IGKV4-53*01_S4588_MRL	MRL/MPJ	IGKV	MUSMUS IGKV4-53*01 F	98.94	GAAATTGTGCTCACCCAGTCTCCAGCACTCATGGCTGCATCTCCAGGGGAGAAGGTCACCATCACCTGCAGTGTCAGCTCAAGTATAAGTTCCAGCAACTTACACTGGTACCAGCAGAAGTCAGGAACCTCCCCCAAACCCTGGATTTATGGCACATCCAACCTTGCTTCTGGAGTCCCTGTTCGCTTCAGTGGCAGTGGATCTGGGACCTCTTATTCTCTCACAATCAGCAGCATGGAGGCTGAAGATGCTGCCACTTATTACTGTCAACAGTGGAGTAGTTACCCGCTCA

IGKV4-53*01_S8918_PWD	PWD/PHJ	IGKV	MUSMUS IGKV4-60*01 P	98.23	GAAATTGTGCTCACCCAGTCTCCAGCACTCATGGCTGCATCTCCAGGGGAGAAGGTCAGCATCACCTGCAGTGTCAGCTCAAGTATAAGTTCCAGCCACTTACACTGGTACCAGCAGAAGTCAGGAACCTCCCCCAAACTCTGGATTTATGGCACATCCAACCTGGCTTCTGGAGTCCCTGCTCGCTTCAGTGGCAGTGGATCTGGGACCTCTTACTCTCTCACAATCAGCAGCATGGAGGCTGAAGATGCTGCCACTTATTACTGTCAACAGTGGAGTAGTTACCCACTCA
IGKV4-53*01_SJL	SJL/J	IGKV	MUSMUS IGKV4-53*01 F	100	GAAATTGTGCTCACCCAGTCTCCAGCACTCATGGCTGCATCTCCAGGGGAGAAGGTCACCATCACCTGCAGTGTCAGCTCAAGTATAAGTTCCAGCAACTTGCACTGGTACCAGCAGAAGTCAGAAACCTCCCCCAAACCCTGGATTTATGGCACATCCAACCTGGCTTCTGGAGTCCCTGTTCGCTTCAGTGGCAGTGGATCTGGGACCTCTTATTCTCTCACAATCAGCAGCATGGAGGCTGAAGATGCTGCCACTTATTACTGTCAACAGTGGAGTAGTTACCCACTCA
IGKV4-55*01_AJ	A/J	IGKV	MUSMUS IGKV4-55*01 F	100	CAAATTGTTCTCACCCAGTCTCCAGCAATCATGTCTGCATCTCCAGGGGAGAAGGTCACCATGACCTGCAGTGCCAGCTCAAGTGTAAGTTACATGTACTGGTACCAGCAGAAGCCAGGATCCTCCCCCAGACTCCTGATTTATGACACATCCAACCTGGCTTCTGGAGTCCCTGTTCGCTTCAGTGGCAGTGGGTCTGGGACCTCTTACTCTCTCACAATCAGCCGAATGGAGGCTGAAGATGCTGCCACTTATTACTGCCAGCAGTGGAGTAGTTACCCACCCA
IGKV4-55*01_AKR	AKR/J	IGKV	MUSMUS IGKV4-55*01 F	100	CAAATTGTTCTCACCCAGTCTCCAGCAATCATGTCTGCATCTCCAGGGGAGAAGGTCACCATGACCTGCAGTGCCAGCTCAAGTGTAAGTTACATGTACTGGTACCAGCAGAAGCCAGGATCCTCCCCCAGACTCCTGATTTATGACACATCCAACCTGGCTTCTGGAGTCCCTGTTCGCTTCAGTGGCAGTGGGTCTGGGACCTCTTACTCTCTCACAATCAGCCGAATGGAGGCTGAAGATGCTGCCACTTATTACTGCCAGCAGTGGAGTAGTTACCCACCCA

IGKV4-55*01_B6	C57BL/6J	IGKV	MUSMUS IGKV4-55*01 F	100	CAAATTGTTCTCACCCAGTCTCCAGCAATCATGTCTGCATCTCCAGGGGAGAAGGTCACCATGACCTGCAGTGCCAGCTCAAGTGTAAGTTACATGTACTGGTACCAGCAGAAGCCAGGATCCTCCCCCAGACTCCTGATTTATGACACATCCAACCTGGCTTCTGGAGTCCCTGTTCGCTTCAGTGGCAGTGGGTCTGGGACCTCTTACTCTCTCACAATCAGCCGAATGGAGGCTGAAGATGCTGCCACTTATTACTGCCAGCAGTGGAGTAGTTACCCACCCA
IGKV4-55*01_BALB	BALB/CBYJ	IGKV	MUSMUS IGKV4-55*01 F	100	CAAATTGTTCTCACCCAGTCTCCAGCAATCATGTCTGCATCTCCAGGGGAGAAGGTCACCATGACCTGCAGTGCCAGCTCAAGTGTAAGTTACATGTACTGGTACCAGCAGAAGCCAGGATCCTCCCCCAGACTCCTGATTTATGACACATCCAACCTGGCTTCTGGAGTCCCTGTTCGCTTCAGTGGCAGTGGGTCTGGGACCTCTTACTCTCTCACAATCAGCCGAATGGAGGCTGAAGATGCTGCCACTTATTACTGCCAGCAGTGGAGTAGTTACCCACCCA
IGKV4-55*01_DBA1	DBA/1J	IGKV	MUSMUS IGKV4-55*01 F	100	CAAATTGTTCTCACCCAGTCTCCAGCAATCATGTCTGCATCTCCAGGGGAGAAGGTCACCATGACCTGCAGTGCCAGCTCAAGTGTAAGTTACATGTACTGGTACCAGCAGAAGCCAGGATCCTCCCCCAGACTCCTGATTTATGACACATCCAACCTGGCTTCTGGAGTCCCTGTTCGCTTCAGTGGCAGTGGGTCTGGGACCTCTTACTCTCTCACAATCAGCCGAATGGAGGCTGAAGATGCTGCCACTTATTACTGCCAGCAGTGGAGTAGTTACCCACCCA
IGKV4-55*01_NOR	NOR/LTJ	IGKV	MUSMUS IGKV4-55*01 F	100	CAAATTGTTCTCACCCAGTCTCCAGCAATCATGTCTGCATCTCCAGGGGAGAAGGTCACCATGACCTGCAGTGCCAGCTCAAGTGTAAGTTACATGTACTGGTACCAGCAGAAGCCAGGATCCTCCCCCAGACTCCTGATTTATGACACATCCAACCTGGCTTCTGGAGTCCCTGTTCGCTTCAGTGGCAGTGGGTCTGGGACCTCTTACTCTCTCACAATCAGCCGAATGGAGGCTGAAGATGCTGCCACTTATTACTGCCAGCAGTGGAGTAGTTACCCACCCA

IGKV4-55*01_NZB	NZB/BLNJ	IGKV	MUSMUS IGKV4-55*01 F	100	CAAATTGTTCTCACCCAGTCTCCAGCAATCATGTCTGCATCTCCAGGGGAGAAGGTCACCATGACCTGCAGTGCCAGCTCAAGTGTAAGTTACATGTACTGGTACCAGCAGAAGCCAGGATCCTCCCCCAGACTCCTGATTTATGACACATCCAACCTGGCTTCTGGAGTCCCTGTTCGCTTCAGTGGCAGTGGGTCTGGGACCTCTTACTCTCTCACAATCAGCCGAATGGAGGCTGAAGATGCTGCCACTTATTACTGCCAGCAGTGGAGTAGTTACCCACCCA
IGKV4-55*01_S5216_CAST	CAST/EIJ	IGKV	MUSMUS IGKV4-55*01 F	97.46	CAAATTGTTCTCACCCAGTCTCCAGCAATCATGTCTGCATCTCCAGGGGAGAAGGTCACCATGACCTGCAGTGCCAGCTCAAGTGTAAGTTACATGTACTGGTACCAGCAGAAGCCAGGATCCTCCCCCAGACTCTGGATTTATGACACATCCAACCTGGCTTCTGGAGTCCCCGCTCGCTTCAGTGGCAGTGGGTCTGGGACCTCTTATTCTCTCACAATCAGCAGCATGGAGGCTGAAGATGCTGCCACTTATTACTGCCAGCAGTGGAGTAGTTACCCACTCA
IGKV4-55*01_S6279_129	129S1/SVIMJ	IGKV	MUSMUS IGKV4-55*01 F	100	CAAATTGTTCTCACCCAGTCTCCAGCAATCATGTCTGCATCTCCAGGGGAGAAGGTCACCATGACCTGCAGTGCCAGCTCAAGTGTAAGTTACATGTACTGGTACCAGCAGAAGCCAGGATCCTCCCCCAGACTCCTGATTTATGACACATCCAACCTGGCTTCTGGAGTCCCTGTTCGCTTCAGTGGCAGTGGGTCTGGGACCTCTTACTCTCTCACAATCAGCCGAATGGAGGCTGAAGATGCTGCCACTTATTACTGCCAGCAGTGGAGTAGTTACCCACTCA
IGKV4-55*01_S6279_C3H	C3H/HEJ	IGKV	MUSMUS IGKV4-55*01 F	100	CAAATTGTTCTCACCCAGTCTCCAGCAATCATGTCTGCATCTCCAGGGGAGAAGGTCACCATGACCTGCAGTGCCAGCTCAAGTGTAAGTTACATGTACTGGTACCAGCAGAAGCCAGGATCCTCCCCCAGACTCCTGATTTATGACACATCCAACCTGGCTTCTGGAGTCCCTGTTCGCTTCAGTGGCAGTGGGTCTGGGACCTCTTACTCTCTCACAATCAGCCGAATGGAGGCTGAAGATGCTGCCACTTATTACTGCCAGCAGTGGAGTAGTTACCCACTCA

IGKV4-55*01_S6279_CBA	CBA/J	IGKV	MUSMUS IGKV4-55*01 F	100	CAAATTGTTCTCACCCAGTCTCCAGCAATCATGTCTGCATCTCCAGGGGAGAAGGTCACCATGACCTGCAGTGCCAGCTCAAGTGTAAGTTACATGTACTGGTACCAGCAGAAGCCAGGATCCTCCCCCAGACTCCTGATTTATGACACATCCAACCTGGCTTCTGGAGTCCCTGTTCGCTTCAGTGGCAGTGGGTCTGGGACCTCTTACTCTCTCACAATCAGCCGAATGGAGGCTGAAGATGCTGCCACTTATTACTGCCAGCAGTGGAGTAGTTACCCACTCA
IGKV4-55*01_S6279_DBA1	DBA/1J	IGKV	MUSMUS IGKV4-55*01 F	100	CAAATTGTTCTCACCCAGTCTCCAGCAATCATGTCTGCATCTCCAGGGGAGAAGGTCACCATGACCTGCAGTGCCAGCTCAAGTGTAAGTTACATGTACTGGTACCAGCAGAAGCCAGGATCCTCCCCCAGACTCCTGATTTATGACACATCCAACCTGGCTTCTGGAGTCCCTGTTCGCTTCAGTGGCAGTGGGTCTGGGACCTCTTACTCTCTCACAATCAGCCGAATGGAGGCTGAAGATGCTGCCACTTATTACTGCCAGCAGTGGAGTAGTTACCCACTCA
IGKV4-55*01_S6279_DBA2	DBA/2J	IGKV	MUSMUS IGKV4-55*01 F	100	CAAATTGTTCTCACCCAGTCTCCAGCAATCATGTCTGCATCTCCAGGGGAGAAGGTCACCATGACCTGCAGTGCCAGCTCAAGTGTAAGTTACATGTACTGGTACCAGCAGAAGCCAGGATCCTCCCCCAGACTCCTGATTTATGACACATCCAACCTGGCTTCTGGAGTCCCTGTTCGCTTCAGTGGCAGTGGGTCTGGGACCTCTTACTCTCTCACAATCAGCCGAATGGAGGCTGAAGATGCTGCCACTTATTACTGCCAGCAGTGGAGTAGTTACCCACTCA
IGKV4-55*01_S7109_MSM	MSM/MSJ	IGKV	MUSMUS IGKV4-55*01 F	97.46	CAAATTGTTCTCACCCAGTCTCCAGCAATCATGTCTGCATCTCCAGGGGAGAAGGTCACCATGACCTGCAGTGCCAGCTCAAGTGTAAGTTACATGTACTGGTACCAGCAGAAGCCAGGATCCTCCCCCAGACTCTGGATTTATGACACATCCAACCTGGCTTCTGGAGTCCCCGCTCGCTTCAGTGGCAGTGGGTCTGGGACCTCTTATTCTCTCACAATCAGCAGCATGGAGGCTGAAGATGCTGCCACTTATTACTGCCAGCAGTGGAGTAGTAACCAACCCA

IGKV4-55*01_S7109_PWD	PWD/PHJ	IGKV	MUSMUS IGKV4-55*01 F	97.46	CAAATTGTTCTCACCCAGTCTCC<br>AGCAATCATGTCTGCATCTCCA<br>GGGGAGAAGGTCACCATGACC<br>TGCAGTGCCAGCTCAAGTGTAA<br>GTTACATGTACTGGTACCAGCA<br>GAAGCCAGGATCCTCCCCCAG<br>ACTCTGGATTTATGACACATCCA<br>ACCTGGCTTCTGGAGTCCCCG<br>CTCGCTTCAGTGGCAGTGGGTC<br>TGGGACCTCTTATTCTCTCACAA<br>TCAGCAGCATGGAGGCTGAAGA<br>TGCTGCCACTTATTACTGCCAG<br>CAGTGGAGTAGTAACCAACCCA
IGKV4-55*01_S8231_CAST	CAST/EIJ	IGKV	MUSMUS IGKV4-55*01 F	95.65	CAAATTGTTCTCACCCAGTCTCC<br>AGCAATCATGTCTGCATCTCCA<br>GGGGAGAAGGTCACCATGACC<br>TGCAGTGCCAGCTCAAGTGTAA<br>GTTACATGTACTGGTACCAGCA<br>GAAGCCAGGATCCTCCCCCAG<br>ACTCTGGATTTATGACACATCCA<br>ACCTGGCTTCTGGAGTTTCCGC<br>TCGCTTCCGTGGCAGTGGGTCT<br>GGGACCTCTTATTCTCTCACAAT<br>CAGCAGCATGGAGGCTGAAGAT<br>GCTGCCACTTATTACTGCTATCA<br>GTGGAGTAGTTACCCACCCA
IGKV4-55*01_S8319_LEWES	LEWES/EIJ	IGKV	MUSMUS IGKV4-55*01 F	100	CAAATTGTTCTCACCCAGTCTCC<br>AGCAATCATGTCTGCATCTCCA<br>GGGGAGAAGGTCACCATGACC<br>TGCAGTGCCAGCTCAAGTGTAA<br>GTTACATGTACTGGTACCAGCA<br>GAAGCCAGGATCCTCCCCCAG<br>ACTCCTGATTTATGACACATCCA<br>ACCTGGCTTCTGGAGTCCCTGT<br>TCGCTTCAGTGGCAGTGGGTCT<br>GGGACCTCTTACTCTCTCACAAT<br>CAGCCGAATGGAGGCTGAAGAT<br>GCTGCCACTTATTACTGCCAGC<br>AGTGGAGTAGTTACCCGCTCA
IGKV4-55*01_SJL	SJL/J	IGKV	MUSMUS IGKV4-55*01 F	100	CAAATTGTTCTCACCCAGTCTCC<br>AGCAATCATGTCTGCATCTCCA<br>GGGGAGAAGGTCACCATGACC<br>TGCAGTGCCAGCTCAAGTGTAA<br>GTTACATGTACTGGTACCAGCA<br>GAAGCCAGGATCCTCCCCCAG<br>ACTCCTGATTTATGACACATCCA<br>ACCTGGCTTCTGGAGTCCCTGT<br>TCGCTTCAGTGGCAGTGGGTCT<br>GGGACCTCTTACTCTCTCACAAT<br>CAGCCGAATGGAGGCTGAAGAT<br>GCTGCCACTTATTACTGCCAGC<br>AGTGGAGTAGTTACCCACCCA

IGKV4-56*01_AJ	A/J	IGKV	MUSMUS IGKV4-56*01 P	100	CAAATTGTTCTCACCCAGTCTCCAGCAATCATGTCTGCATCTCCAGGGCAGAAAGTCACCATAACCTGCAGTGCCATCTCAAGTGTAAATTACATGCACTGGTACCAGCAGAAGCCAGGATCCTCCCCCAAACTCTGGATTTATGCAACATCCAAACTGGCTCTTGGAGTCCCTGCTTGCTTCAGTGGCAGTGGGTCTGGGACCTCTTACTCTCTCACAATCAGCAGCATGGTGGCTGAAGATGCCACCTCTTATTTCTGTCATCAGTGGAGTAGTTACCCACCCA
IGKV4-56*01_BALB	BALB/CBYJ	IGKV	MUSMUS IGKV4-56*01 P	100	CAAATTGTTCTCACCCAGTCTCCAGCAATCATGTCTGCATCTCCAGGGCAGAAAGTCACCATAACCTGCAGTGCCATCTCAAGTGTAAATTACATGCACTGGTACCAGCAGAAGCCAGGATCCTCCCCCAAACTCTGGATTTATGCAACATCCAAACTGGCTCTTGGAGTCCCTGCTTGCTTCAGTGGCAGTGGGTCTGGGACCTCTTACTCTCTCACAATCAGCAGCATGGTGGCTGAAGATGCCACCTCTTATTTCTGTCATCAGTGGAGTAGTTACCCACCCA
IGKV4-56*01_CBA	CBA/J	IGKV	MUSMUS IGKV4-56*01 P	100	CAAATTGTTCTCACCCAGTCTCCAGCAATCATGTCTGCATCTCCAGGGCAGAAAGTCACCATAACCTGCAGTGCCATCTCAAGTGTAAATTACATGCACTGGTACCAGCAGAAGCCAGGATCCTCCCCCAAACTCTGGATTTATGCAACATCCAAACTGGCTCTTGGAGTCCCTGCTTGCTTCAGTGGCAGTGGGTCTGGGACCTCTTACTCTCTCACAATCAGCAGCATGGTGGCTGAAGATGCCACCTCTTATTTCTGTCATCAGTGGAGTAGTTACCCACCCA
IGKV4-56*01_DBA1	DBA/1J	IGKV	MUSMUS IGKV4-56*01 P	100	CAAATTGTTCTCACCCAGTCTCCAGCAATCATGTCTGCATCTCCAGGGCAGAAAGTCACCATAACCTGCAGTGCCATCTCAAGTGTAAATTACATGCACTGGTACCAGCAGAAGCCAGGATCCTCCCCCAAACTCTGGATTTATGCAACATCCAAACTGGCTCTTGGAGTCCCTGCTTGCTTCAGTGGCAGTGGGTCTGGGACCTCTTACTCTCTCACAATCAGCAGCATGGTGGCTGAAGATGCCACCTCTTATTTCTGTCATCAGTGGAGTAGTTACCCACCCA

IGKV4-56*01_DBA2	DBA/2J	IGKV	MUSMUS IGKV4-56*01 P	100	CAAATTGTTCTCACCCAGTCTCCAGCAATCATGTCTGCATCTCCAGGGCAGAAAGTCACCATAACCTGCAGTGCCATCTCAAGTGTAAATTACATGCACTGGTACCAGCAGAAGCCAGGATCCTCCCCCAAACTCTGGATTTATGCAACATCCAAACTGGCTCTTGGAGTCCCTGCTTGCTTCAGTGGCAGTGGGTCTGGGACCTCTTACTCTCTCACAATCAGCAGCATGGTGGCTGAAGATGCCACCTCTTATTTCTGTCATCAGTGGAGTAGTTACCCACCCA
IGKV4-56*01_NZB	NZB/BLNJ	IGKV	MUSMUS IGKV4-56*01 P	100	CAAATTGTTCTCACCCAGTCTCCAGCAATCATGTCTGCATCTCCAGGGCAGAAAGTCACCATAACCTGCAGTGCCATCTCAAGTGTAAATTACATGCACTGGTACCAGCAGAAGCCAGGATCCTCCCCCAAACTCTGGATTTATGCAACATCCAAACTGGCTCTTGGAGTCCCTGCTTGCTTCAGTGGCAGTGGGTCTGGGACCTCTTACTCTCTCACAATCAGCAGCATGGTGGCTGAAGATGCCACCTCTTATTTCTGTCATCAGTGGAGTAGTTACCCACCCA
IGKV4-56*01_S2201_CAST	CAST/EIJ	IGKV	MUSMUS IGKV4-56*01 P	96.38	CAAATTGTTCTCACCCAGTCTCCAGCAATCATGTCTGCATCTCCGGGGCAGAAAGTCACCATAACCTGCAGTGCCAGCTCAAGTGTAAATTACATGCACTGGTACCAGCAGAAGCCAGGATCCTCCCCCAAACTCTGGATTTATGACACATCCAAACTGGCTCCTGGAGTCCCTGCTCGCTTCAGTGGCAGTGGGTCTGGGACCTCTTACTCTCTCACAATCAGCAGCATGGAGGCTGAAGATGCTGCCTCTTATTTCTGCCATCAGTGGAGTAGTTACCCACCCA
IGKV4-56*01_S4810_MSM	MSM/MSJ	IGKV	MUSMUS IGKV4-59*01 F	94.57	CAAATTGTTCTCACCCAGTCTCCAGCAATCATTTCTGCATCTCCGGGGCAGAAAGTCACCATAACCTGCAGTGCCAGCTCCAGTGTAAATTACATGCACTGGTTCCAGCAGAAGCCAGGATCCTCCCCCAAACTCTGGATTTATGACACATCCAAACTGGCTCCTGGAGTCCCTGCTCGCTTCAGTGGCAGTGGGTCTGGGACCTCTTACTCTCTCACAATCAGCAGCATGGAGGCTGAAGATGCTGCCACTTATTACTGCCAGCAGTGGAGTAGTTACCCACCCA

IGKV4-56*01_S4810_PWD	PWD/PHJ	IGKV	MUSMUS IGKV4-59*01 F	94.57	CAAATTGTTCTCACCCAGTCTCCAGCAATCATTTCTGCATCTCCGGGGCAGAAAGTCACCATAACCTGCAGTGCCAGCTCCAGTGTAAATTACATGCACTGGTTCCAGCAGAAGCCAGGATCCTCCCCCAAACTCTGGATTTATGACACATCCAAACTGGCTCCTGGAGTCCCTGCTCGCTTCAGTGGCAGTGGGTCTGGGACCTCTTACTCTCTCACAATCAGCAGCATGGAGGCTGAAGATGCTGCCACTTATTACTGCCAGCAGTGGAGTAGTTACCCACCCA
IGKV4-56*01_S5696_AKR	AKR/J	IGKV	MUSMUS IGKV4-56*01 P	96.38	CAAATTGTTCTCACCCAGTCTCCAGCAATCATGTCTGCATCTCCAGGGCAGAAAGTCACCATAACCTGCAGTGCCAGCTCAAGTGTAAATTACATGCACTGGTACCAGCAGAAGCTAGGATCCTCCCCCAAACTCTGGATTTATGACACATCCAAACTGGCTCCTGGAGTCCCTGCTCGCTTCAGTGGCAGTGGGTCTGGGACCTCTTACTCTCTCACAATCAGCAGCATGGAGGCTGAAGATGCTGCCTCTTATTTCTGCCATCAGTGGAGTAGTTACCCACCCA
IGKV4-56*01_S5696_MRL	MRL/MPJ	IGKV	MUSMUS IGKV4-56*01 P	96.38	CAAATTGTTCTCACCCAGTCTCCAGCAATCATGTCTGCATCTCCAGGGCAGAAAGTCACCATAACCTGCAGTGCCAGCTCAAGTGTAAATTACATGCACTGGTACCAGCAGAAGCTAGGATCCTCCCCCAAACTCTGGATTTATGACACATCCAAACTGGCTCCTGGAGTCCCTGCTCGCTTCAGTGGCAGTGGGTCTGGGACCTCTTACTCTCTCACAATCAGCAGCATGGAGGCTGAAGATGCTGCCTCTTATTTCTGCCATCAGTGGAGTAGTTACCCACCCA
IGKV4-56*01_S5696_NOD	NOD/SHILTJ	IGKV	MUSMUS IGKV4-56*01 P	96.38	CAAATTGTTCTCACCCAGTCTCCAGCAATCATGTCTGCATCTCCAGGGCAGAAAGTCACCATAACCTGCAGTGCCAGCTCAAGTGTAAATTACATGCACTGGTACCAGCAGAAGCTAGGATCCTCCCCCAAACTCTGGATTTATGACACATCCAAACTGGCTCCTGGAGTCCCTGCTCGCTTCAGTGGCAGTGGGTCTGGGACCTCTTACTCTCTCACAATCAGCAGCATGGAGGCTGAAGATGCTGCCTCTTATTTCTGCCATCAGTGGAGTAGTTACCCACCCA

IGKV4-56*01_S5696_NOR	NOR/LTJ	IGKV	MUSMUS IGKV4-56*01 P	96.38	CAAATTGTTCTCACCCAGTCTCCAGCAATCATGTCTGCATCTCCAGGGCAGAAAGTCACCATAACCTGCAGTGCCAGCTCAAGTGTAAATTACATGCACTGGTACCAGCAGAAGCTAGGATCCTCCCCCAAACTCTGGATTTATGACACATCCAAACTGGCTCCTGGAGTCCCTGCTCGCTTCAGTGGCAGTGGGTCTGGGACCTCTTACTCTCTCACAATCAGCAGCATGGAGGCTGAAGATGCTGCCTCTTATTTCTGCCATCAGTGGAGTAGTTACCCACCCA
IGKV4-56*01_S5954_DBA2	DBA/2J	IGKV	MUSMUS IGKV4-56*01 P	100	CAAATTGTTCTCACCCAGTCTCCAGCAATCATGTCTGCATCTCCAGGGCAGAAAGTCACCATAACCTGCAGTGCCATCTCAAGTGTAAATTACATGCACTGGTACCAGCAGAAGCCAGGATCCTCCCCCAAACTCTGGATTTATGCAACATCCAAACTGGCTCTTGGAGTCCCTGCTTGCTTCAGTGGCAGTGGGTCTGGGACCTCTTACTCTCTCACAATCAGCAGCATGGTGGCTGAAGATGCCACCTCTTATTTCTGTCATCAGTGGAGTAGTTACCCACTCA
IGKV4-56*01_S9438_MSM	MSM/MSJ	IGKV	MUSMUS IGKV4-59*01 F	94.57	CAAATTGTTCTCACCCAGTCTCCAGCAATCATTTCTGCATCTCCGGGGCAGAAAGTCACCATAACCTGCAGTGCCAGCTCCAGTGTAAATTACATGCACTGGTTCCAGCAGAAGCCAGGATCCTCCCCCAAACTCTGGATTTATGACACATCCAAACTGGCTCCTGGAGTCCCTGCTCGCTTCAGTGGCAGTGGGTCTGGGACCTCTTACTCTCTCACAATCAGCAGCATGGAGGCTGAAGATGCTGCCACTTATTACTGCCAGCAGTGGAGTAGTTACCCGCTCA
IGKV4-57-1*01_129	129S1/SVIMJ	IGKV	MUSMUS IGKV4-57*01 F	100	GAAAATGTGCTCACCCAGTCTCCAGCAATCATGTCTGCATCTCCAGGGGAAAAGGTCACCATGACCTGCAGGGCCAGCTCAAGTGTAAGTTCCAGTTACTTGCACTGGTACCAGCAGAAGTCAGGTGCCTCCCCCAAACTCTGGATTTATAGCACATCCAACTTGGCTTCTGGAGTCCCTGCTCGCTTCAGTGGCAGTGGGTCTGGGACCTCTTACTCTCTCACAATCAGCAGTGTGGAGGCTGAAGATGCTGCCACTTATTACTGCCAGCAGTACAGTGGTTACCCACTCA

IGKV4-57-1*01_AJ	A/J	IGKV	MUSMUS IGKV4-57*01 F	100	GAAAATGTGCTCACCCAGTCTCCAGCAATCATGTCTGCATCTCCAGGGGAAAAGGTCACCATGACCTGCAGGGCCAGCTCAAGTGTAAGTTCCAGTTACTTGCACTGGTACCAGCAGAAGTCAGGTGCCTCCCCCAAACTCTGGATTTATAGCACATCCAACTTGGCTTCTGGAGTCCCTGCTCGCTTCAGTGGCAGTGGGTCTGGGACCTCTTACTCTCTCACAATCAGCAGTGTGGAGGCTGAAGATGCTGCCACTTATTACTGCCAGCAGTACAGTGGTTACCCACTCA
IGKV4-57-1*01_B6	C57BL/6J	IGKV	MUSMUS IGKV4-57*01 F	100	GAAAATGTGCTCACCCAGTCTCCAGCAATCATGTCTGCATCTCCAGGGGAAAAGGTCACCATGACCTGCAGGGCCAGCTCAAGTGTAAGTTCCAGTTACTTGCACTGGTACCAGCAGAAGTCAGGTGCCTCCCCCAAACTCTGGATTTATAGCACATCCAACTTGGCTTCTGGAGTCCCTGCTCGCTTCAGTGGCAGTGGGTCTGGGACCTCTTACTCTCTCACAATCAGCAGTGTGGAGGCTGAAGATGCTGCCACTTATTACTGCCAGCAGTACAGTGGTTACCCACTCA
IGKV4-57-1*01_BALB	BALB/CBYJ	IGKV	MUSMUS IGKV4-57*01 F	100	GAAAATGTGCTCACCCAGTCTCCAGCAATCATGTCTGCATCTCCAGGGGAAAAGGTCACCATGACCTGCAGGGCCAGCTCAAGTGTAAGTTCCAGTTACTTGCACTGGTACCAGCAGAAGTCAGGTGCCTCCCCCAAACTCTGGATTTATAGCACATCCAACTTGGCTTCTGGAGTCCCTGCTCGCTTCAGTGGCAGTGGGTCTGGGACCTCTTACTCTCTCACAATCAGCAGTGTGGAGGCTGAAGATGCTGCCACTTATTACTGCCAGCAGTACAGTGGTTACCCACTCA
IGKV4-57-1*01_C3H	C3H/HEJ	IGKV	MUSMUS IGKV4-57*01 F	100	GAAAATGTGCTCACCCAGTCTCCAGCAATCATGTCTGCATCTCCAGGGGAAAAGGTCACCATGACCTGCAGGGCCAGCTCAAGTGTAAGTTCCAGTTACTTGCACTGGTACCAGCAGAAGTCAGGTGCCTCCCCCAAACTCTGGATTTATAGCACATCCAACTTGGCTTCTGGAGTCCCTGCTCGCTTCAGTGGCAGTGGGTCTGGGACCTCTTACTCTCTCACAATCAGCAGTGTGGAGGCTGAAGATGCTGCCACTTATTACTGCCAGCAGTACAGTGGTTACCCACTCA

IGKV4-57-1*01_CBA	CBA/J	IGKV	MUSMUS IGKV4-57*01 F	100	GAAAATGTGCTCACCCAGTCTCCAGCAATCATGTCTGCATCTCCAGGGGAAAAGGTCACCATGACCTGCAGGGCCAGCTCAAGTGTAAGTTCCAGTTACTTGCACTGGTACCAGCAGAAGTCAGGTGCCTCCCCCAAACTCTGGATTTATAGCACATCCAACTTGGCTTCTGGAGTCCCTGCTCGCTTCAGTGGCAGTGGGTCTGGGACCTCTTACTCTCTCACAATCAGCAGTGTGGAGGCTGAAGATGCTGCCACTTATTACTGCCAGCAGTACAGTGGTTACCCACTCA
IGKV4-57-1*01_DBA1	DBA/1J	IGKV	MUSMUS IGKV4-57*01 F	100	GAAAATGTGCTCACCCAGTCTCCAGCAATCATGTCTGCATCTCCAGGGGAAAAGGTCACCATGACCTGCAGGGCCAGCTCAAGTGTAAGTTCCAGTTACTTGCACTGGTACCAGCAGAAGTCAGGTGCCTCCCCCAAACTCTGGATTTATAGCACATCCAACTTGGCTTCTGGAGTCCCTGCTCGCTTCAGTGGCAGTGGGTCTGGGACCTCTTACTCTCTCACAATCAGCAGTGTGGAGGCTGAAGATGCTGCCACTTATTACTGCCAGCAGTACAGTGGTTACCCACTCA
IGKV4-57-1*01_DBA2	DBA/2J	IGKV	MUSMUS IGKV4-57*01 F	100	GAAAATGTGCTCACCCAGTCTCCAGCAATCATGTCTGCATCTCCAGGGGAAAAGGTCACCATGACCTGCAGGGCCAGCTCAAGTGTAAGTTCCAGTTACTTGCACTGGTACCAGCAGAAGTCAGGTGCCTCCCCCAAACTCTGGATTTATAGCACATCCAACTTGGCTTCTGGAGTCCCTGCTCGCTTCAGTGGCAGTGGGTCTGGGACCTCTTACTCTCTCACAATCAGCAGTGTGGAGGCTGAAGATGCTGCCACTTATTACTGCCAGCAGTACAGTGGTTACCCACTCA
IGKV4-57-1*01_LEWES	LEWES/EiJ	IGKV	MUSMUS IGKV4-57*01 F	100	GAAAATGTGCTCACCCAGTCTCCAGCAATCATGTCTGCATCTCCAGGGGAAAAGGTCACCATGACCTGCAGGGCCAGCTCAAGTGTAAGTTCCAGTTACTTGCACTGGTACCAGCAGAAGTCAGGTGCCTCCCCCAAACTCTGGATTTATAGCACATCCAACTTGGCTTCTGGAGTCCCTGCTCGCTTCAGTGGCAGTGGGTCTGGGACCTCTTACTCTCTCACAATCAGCAGTGTGGAGGCTGAAGATGCTGCCACTTATTACTGCCAGCAGTACAGTGGTTACCCACTCA

IGKV4-57-1*01_NZB	NZB/BLNJ	IGKV	MUSMUS IGKV4-57*01 F	100	GAAAATGTGCTCACCCAGTCTC<br>CAGCAATCATGTCTGCATCTCC<br>AGGGGAAAAGGTCACCATGAC<br>CTGCAGGGCCAGCTCAAGTGTA<br>AGTTCCAGTTACTTGCACTGGTA<br>CCAGCAGAAGTCAGGTGCCTC<br>CCCCAAACTCTGGATTTATAGCA<br>CATCCAACTTGGCTTCTGGAGT<br>CCCTGCTCGCTTCAGTGGCAGT<br>GGGTCTGGGACCTCTTACTCTC<br>TCACAATCAGCAGTGTGGAGGC<br>TGAAGATGCTGCCACTTATTACT<br>GCCAGCAGTACAGTGGTTACCC<br>ACTCA
IGKV4-57-1*01_S1667_PWD	PWD/PHJ	IGKV	MUSMUS IGKV4-57*01 F	95.65	GAAAATGTGCTCACCCAGTCTC<br>CAGCACTCATGGCTGCATCTCC<br>AGGGGAGAAGGTCACCATGAC<br>CTGCAGTGCCAGCTCAAGTGTA<br>AGTTCCAGTTACTTGCACTGGTA<br>CCAGCAGAAGTCAGGTGCCTC<br>CCCCAAACTCTGGATTTACGGC<br>ACATCCAACCTGGCTTCTGGAG<br>TCCCAGCTCGCTTCAGTGGCAG<br>TGGGTCTGGGACCTCTTACTCT<br>CTCACAATCAGCAGCGTGGAG<br>GCTGAAGATGATGCAACTTATTA<br>CTGCCAGCAGGCGTGGAATTAC<br>CCATTCA
IGKV4-57-1*01_S1729_AKR	AKR/J	IGKV	MUSMUS IGKV4-57*01 F	100	GAAATTGTGCTCACCCAGTCTC<br>CAGCACTCATGGCTGCATCTCC<br>AGGGGAGAAGGTCACCATGAC<br>CTGCAGTGCCAGCTCAAGTGTA<br>GGTTCCAGTTACTTGCACTGGTA<br>CCAGCAGAAGTCAGGAGCCTC<br>CCCCAAACTCTGGATTTACGGC<br>ACATCCAACCTGGCTTCTGGAG<br>TCCCTGCTTGCTTCAGTGGCAG<br>TGGGTCTGGGACCTCTTACTCT<br>CTCACAATCAGCAGCGTGGAG<br>GCTGAAGATGATGCAACTTATTA<br>CTGCCAGCAGGGGTGGGATTA<br>CCCACTCA
IGKV4-57-1*01_S1729_NOD	NOD/SHILTJ	IGKV	MUSMUS IGKV4-57-1*01 F	92.91	GAAATTGTGCTCACCCAGTCTC<br>CAGCACTCATGGCTGCATCTCC<br>AGGGGAGAAGGTCACCATGAC<br>CTGCAGTGCCAGCTCAAGTGTA<br>GGTTCCAGTTACTTGCACTGGTA<br>CCAGCAGAAGTCAGGAGCCTC<br>CCCCAAACTCTGGATTTACGGC<br>ACATCCAACCTGGCTTCTGGAG<br>TCCCTGCTTGCTTCAGTGGCAG<br>TGGGTCTGGGACCTCTTACTCT<br>CTCACAATCAGCAGCGTGGAG<br>GCTGAAGATGATGCAACTTATTA<br>CTGCCAGCAGGGGTGGGATTA<br>CCCACTCA

IGKV4-57-1*01_S4182_PWD	PWD/PHJ	IGKV	MUSMUS IGKV4-57-1*01 F	93.62	GAAATTGTGCTCACCCAGTCTCCAACACTCATGACTGTATCTCCAGGGGAGAAGGTCACCATGACCTGCAGTGCCAGCTCAAGTGTAAGTTCCAGTTACTTGTTCTGGTACCAGCAGAAGTCAGGTGCCACCCCTAAACTCTGGATTTACGGCACATCCAACCTGGCTTCTGGAGTCCCAGCTCGCTTCAGTGGCAGTGGGTCTGGGACCTCTTACTCTCTCACAATCAGCAGCATGGAGGCTGAAGATGCTGCCACTTATTACTGCCAGCAGTGGAATGGTGCCCCACTCA
IGKV4-57-1*01_S5694_MSM	MSM/MSJ	IGKV	MUSMUS IGKV4-57*01 F	95.65	GAAAATGTGCTCACCCAGTCTCCAGCACTCATGGCTGCATCTCCAGGGGAGAAGGTCACCATGACCTGCAGTGCCAGCTCAAGTGTAAGTTCCAGTTACTTGCACTGGTACCAGCAGAAGTCAGGTGCCTCCCCCAAACTCTGGATTTACGGCACATCCAACCTAGCTTCTGGAGTCCCAGCTCGCTTCAGTGGCAGTGGGTCTGGGACCTCTTACTCTCTCACAATCAGCAGCGTGGAGGCTGAAGATGATGCAACTTATTACTGCCAGCAGGCGTGGAATTACCCATTCA
IGKV4-57-1*01_S8265_PWD	PWD/PHJ	IGKV	MUSMUS IGKV4-57-1*01 F	92.91	GAAAATGTGCTCACCCAGTCTCCAGCACTCATGGCTGCATCTCCAGGGGAGAAGGTCACCATGACCTGCAGTGCCAGCTCAAGTGTAAGTTCCAGTTACTTGCACTGGTACCAGCAGAAGTCAGGTGCCTCCCCCAAACTCTGGATTTACGGCACATCCAACCTGGCTTCTGGAGTCCCAGCTCGCTTCAGTGGCAGTGGGTCTGGGACCTCTTACTCTCTCACAATCAGCAGCGTGGAGGCTGAAGATGATGCAACTTATTACTGCCAGCAGGCGTGGAATTACCCACTCA
IGKV4-57-1*01_S8603_AKR	AKR/J	IGKV	MUSMUS IGKV4-57-1*01 F	92.91	CAAATTGTTCTCACCCAGTCTCCAGCAATCATGTCTGCCTCTCCAGGGGAGAAGGTCACCATGACCTGCAGTGCCAGCTCAAGTGTAAGTTCCAGGTACTTGCACTGGTACCAGCAGAAGTCAGGAGCCTCCCCCAAACTCTGGATTTATGGCACATCCAACCTGGCTTCTGGAGTCCCTGCTCGCTTCAGTGGCAGTGGGTCTGGGACCTCTTACTCTCTCACAATCAGCAGCGTGGAGGCTGAAGATGCTGCCACTTATTACTGCCAGCAGTATCATAGTGACCCACTCA

IGKV4-57-1*01_S8603_MRL	MRL/MPJ	IGKV	MUSMUS IGKV4-74*01 F	95.04	CAAATTGTTCTCACCCAGTCTCCAGCAATCATGTCTGCCTCTCCAGGGGAGAAGGTCACCATGACCTGCAGTGCCAGCTCAAGTGTAAGTTCCAGGTACTTGCACTGGTACCAGCAGAAGTCAGGAGCCTCCCCCAAACTCTGGATTTATGGCACATCCAACCTGGCTTCTGGAGTCCCTGCTCGCTTCAGTGGCAGTGGGTCTGGGACCTCTTACTCTCTCACAATCAGCAGCGTGGAGGCTGAAGATGCTGCCACTTATTACTGCCAGCAGTATCATAGTGACCCACTCA
IGKV4-57-1*01_S8603_NOD	NOD/SHILTJ	IGKV	MUSMUS IGKV4-74*01 F	95.04	CAAATTGTTCTCACCCAGTCTCCAGCAATCATGTCTGCCTCTCCAGGGGAGAAGGTCACCATGACCTGCAGTGCCAGCTCAAGTGTAAGTTCCAGGTACTTGCACTGGTACCAGCAGAAGTCAGGAGCCTCCCCCAAACTCTGGATTTATGGCACATCCAACCTGGCTTCTGGAGTCCCTGCTCGCTTCAGTGGCAGTGGGTCTGGGACCTCTTACTCTCTCACAATCAGCAGCGTGGAGGCTGAAGATGCTGCCACTTATTACTGCCAGCAGTATCATAGTGACCCACTCA
IGKV4-57-1*01_S8603_NOR	NOR/LTJ	IGKV	MUSMUS IGKV4-74*01 F	95.04	CAAATTGTTCTCACCCAGTCTCCAGCAATCATGTCTGCCTCTCCAGGGGAGAAGGTCACCATGACCTGCAGTGCCAGCTCAAGTGTAAGTTCCAGGTACTTGCACTGGTACCAGCAGAAGTCAGGAGCCTCCCCCAAACTCTGGATTTATGGCACATCCAACCTGGCTTCTGGAGTCCCTGCTCGCTTCAGTGGCAGTGGGTCTGGGACCTCTTACTCTCTCACAATCAGCAGCGTGGAGGCTGAAGATGCTGCCACTTATTACTGCCAGCAGTATCATAGTGACCCACTCA
IGKV4-57-1*01_SJL	SJL/J	IGKV	MUSMUS IGKV4-57*01 F	100	GAAAATGTGCTCACCCAGTCTCCAGCAATCATGTCTGCATCTCCAGGGGAAAAGGTCACCATGACCTGCAGGGCCAGCTCAAGTGTAAGTTCCAGTTACTTGCACTGGTACCAGCAGAAGTCAGGTGCCTCCCCCAAACTCTGGATTTATAGCACATCCAACTTGGCTTCTGGAGTCCCTGCTCGCTTCAGTGGCAGTGGGTCTGGGACCTCTTACTCTCTCACAATCAGCAGTGTGGAGGCTGAAGATGCTGCCACTTATTACTGCCAGCAGTACAGTGGTTACCCACTCA

IGKV4-57*01_129	129S1/SVIMJ	IGKV	MUSMUS IGKV4-57*01 F	100	CAAATTGTTCTCACCCAGTCTCCAGCAATCATGTCTGCATCTCCAGGGGAGAAGGTCACCATAACCTGCAGTGCCAGCTCAAGTGTAAGTTACATGCACTGGTTCCAGCAGAAGCCAGGCACTTCTCCCAAACTCTGGATTTATAGCACATCCAACCTGGCTTCTGGAGTCCCTGCTCGCTTCAGTGGCAGTGGATCTGGGACCTCTTACTCTCTCACAATCAGCCGAATGGAGGCTGAAGATGCTGCCACTTATTACTGCCAGCAAAGGAGTAGTTACCCACCCA
IGKV4-57*01_AKR	AKR/J	IGKV	MUSMUS IGKV4-74*01 F	95.04	CAAATTGTTCTCACCCAGTCTCCAGCAATCATGTCTGCATCTCCAGGGGAGAAGGTCACCATAACCTGCAGTGCCAGCTCAAGTGTAAGTTACATGCACTGGTTCCAGCAGAAGCCAGGCACTTCTCCCAAACTCTGGATTTATAGCACATCCAACCTGGCTTCTGGAGTCCCTGCTCGCTTCAGTGGCAGTGGATCTGGGACCTCTTACTCTCTCACAATCAGCCGAATGGAGGCTGAAGATGCTGCCACTTATTACTGCCAGCAAAGGAGTAGTTACCCACCCA
IGKV4-57*01_LEWES	LEWES/EIJ	IGKV	MUSMUS IGKV4-57*01 F	100	CAAATTGTTCTCACCCAGTCTCCAGCAATCATGTCTGCATCTCCAGGGGAGAAGGTCACCATAACCTGCAGTGCCAGCTCAAGTGTAAGTTACATGCACTGGTTCCAGCAGAAGCCAGGCACTTCTCCCAAACTCTGGATTTATAGCACATCCAACCTGGCTTCTGGAGTCCCTGCTCGCTTCAGTGGCAGTGGATCTGGGACCTCTTACTCTCTCACAATCAGCCGAATGGAGGCTGAAGATGCTGCCACTTATTACTGCCAGCAAAGGAGTAGTTACCCACCCA
IGKV4-57*01_NZB	NZB/BLNJ	IGKV	MUSMUS IGKV4-57*01 F	100	CAAATTGTTCTCACCCAGTCTCCAGCAATCATGTCTGCATCTCCAGGGGAGAAGGTCACCATAACCTGCAGTGCCAGCTCAAGTGTAAGTTACATGCACTGGTTCCAGCAGAAGCCAGGCACTTCTCCCAAACTCTGGATTTATAGCACATCCAACCTGGCTTCTGGAGTCCCTGCTCGCTTCAGTGGCAGTGGATCTGGGACCTCTTACTCTCTCACAATCAGCCGAATGGAGGCTGAAGATGCTGCCACTTATTACTGCCAGCAAAGGAGTAGTTACCCACCCA

IGKV4-57*01_S0701_PWD	PWD/PHJ	IGKV	MUSMUS IGKV4-57-1*01 F	93.62	CAAATTGTTCTCACCCAGTCTCCAGCAATCATGGCTGCATCTCCAGGGGAGAAGTTCACCATAACCTGCAGTGCCAGCTCAAGTGTAAGTTACATGCACTGGTTCCAGCAGAAGCCAGGCACTTCTCCCAAACTCTGGATTTATAGGACATCGAACCTGGCTTCTGGAGTCCCTGCTCGCTTCAGTGGCAGTGGGTCTGGGACCTCTTATTCTCTCACAATCAGCAGCATGGATGCTGAAGATGCTGCCACTTATTACTGCCTGCAGCGGAGTAGTTACCCACCCA
IGKV4-57*01_S6684_AJ	A/J	IGKV	MUSMUS IGKV4-57-1*01 F	100	CAAATTGTTCTCACCCAGTCTCCAGCAATCATGTCTGCATCTCCAGGGGAGAAGGTCACCATAACCTGCAGTGCCAGCTCAAGTGTAAGTTACATGCACTGGTTCCAGCAGAAGCCAGGCACTTCTCCCAAACTCTGGATTTATAGCACATCCAACCTGGCTTCTGGAGTCCCTGCTCGCTTCAGTGGCAGTGGATCTGGGACCTCTTACTCTCTCACAATCAGCCGAATGGAGGCTGAAGATGCTGCCACTTATTACTGCCAGCAAAGGAGTAGTTACCCGCTCA
IGKV4-57*01_S6684_B6	C57BL/6J	IGKV	MUSMUS IGKV4-57-1*01 F	100	CAAATTGTTCTCACCCAGTCTCCAGCAATCATGTCTGCATCTCCAGGGGAGAAGGTCACCATAACCTGCAGTGCCAGCTCAAGTGTAAGTTACATGCACTGGTTCCAGCAGAAGCCAGGCACTTCTCCCAAACTCTGGATTTATAGCACATCCAACCTGGCTTCTGGAGTCCCTGCTCGCTTCAGTGGCAGTGGATCTGGGACCTCTTACTCTCTCACAATCAGCCGAATGGAGGCTGAAGATGCTGCCACTTATTACTGCCAGCAAAGGAGTAGTTACCCGCTCA
IGKV4-57*01_S6684_CBA	CBA/J	IGKV	MUSMUS IGKV4-57-1*01 F	100	CAAATTGTTCTCACCCAGTCTCCAGCAATCATGTCTGCATCTCCAGGGGAGAAGGTCACCATAACCTGCAGTGCCAGCTCAAGTGTAAGTTACATGCACTGGTTCCAGCAGAAGCCAGGCACTTCTCCCAAACTCTGGATTTATAGCACATCCAACCTGGCTTCTGGAGTCCCTGCTCGCTTCAGTGGCAGTGGATCTGGGACCTCTTACTCTCTCACAATCAGCCGAATGGAGGCTGAAGATGCTGCCACTTATTACTGCCAGCAAAGGAGTAGTTACCCGCTCA

IGKV4-57*01_S8859_MSM	MSM/MSJ	IGKV	MUSMUS IGKV4-57-1*01 F	93.26	CAAATTGTTCTCACCCAGTCTCCAGCAATCATGGCTGCATCTCCAGGGGAGAAGTTCACCATAACCTGCAGTGCCAGCTCAAGTGTAAGTTACATGCACTGGTTCCAGCAGAAGCCAGGCACTTCTCCCAAACTCTGGATTTATAGGACATCGAACCTGGCTTCTGGAGTCCCTGCTCGCTTCAGTGGCAGTGGGTCTGGGACCTCTTATTCTCTCACAATCAGCAGCATGGAGGCTGAAGATGCTGCCACTTATTACTGCCTGCAGCAGAGTAGTTACCCACCCA
IGKV4-57*01_S9174_C3H	C3H/HEJ	IGKV	MUSMUS IGKV4-57-1*01 F	100	CAAATTGTTCTCACCCAGTCTCCAGCAATCATGTCTGCATCTCCAGGGGAGAAGGTCACCATAACCTGCAGTGCCAGCTCAAGTGTAAGTTACATGCACTGGTTCCAGCAGAAGCCAGGCACTTCTCCCAAACTCTGGATTTATAGCACATCCAACCTGGCTTCTGGAGTCCCTGCTCGCTTCAGTGGCAGTGGATCTGGGACCTCTTACTCTCTCACAATCAGCCGAATGGAGGCTGAAGATGCTGCCACTTATTACTGCCAGCAAAGGAGTAGTTACCCACACA
IGKV4-57*01_S9219_129	129S1/SVIMJ	IGKV	MUSMUS IGKV4-57-1*01 F	100	CAAATTGTTCTCACCCAGTCTCCAGCAATCATGTCTGCATCTCCAGGGGAGAAGGTCACCATAACCTGCAGTGCCAGCTCAAGTGTAAGTTACATGCACTGGTTCCAGCAGAAGCCAGGCACTTCTCCCAAACTCTGGATTTATAGCACATCCAACCTGGCTTCTGGAGTCCCTGCTCGCTTCAGTGGCAGTGGATCTGGGACCTCTTACTCTCTCACAATCAGCCGAATGGAGGCTGAAGATGCTGCCACTTATTACTGCCAGCAAAGGAGTAGTTACCCACTCA
IGKV4-57*01_S9219_BALB	BALB/CBYJ	IGKV	MUSMUS IGKV4-57-1*01 F	100	CAAATTGTTCTCACCCAGTCTCCAGCAATCATGTCTGCATCTCCAGGGGAGAAGGTCACCATAACCTGCAGTGCCAGCTCAAGTGTAAGTTACATGCACTGGTTCCAGCAGAAGCCAGGCACTTCTCCCAAACTCTGGATTTATAGCACATCCAACCTGGCTTCTGGAGTCCCTGCTCGCTTCAGTGGCAGTGGATCTGGGACCTCTTACTCTCTCACAATCAGCCGAATGGAGGCTGAAGATGCTGCCACTTATTACTGCCAGCAAAGGAGTAGTTACCCACTCA

IGKV4-57*01_S9219_DBA1	DBA/1J	IGKV	MUSMUS IGKV4-57-1*01 F	100	CAAATTGTTCTCACCCAGTCTCCAGCAATCATGTCTGCATCTCCAGGGGAGAAGGTCACCATAACCTGCAGTGCCAGCTCAAGTGTAAGTTACATGCACTGGTTCCAGCAGAAGCCAGGCACTTCTCCCAAACTCTGGATTTATAGCACATCCAACCTGGCTTCTGGAGTCCCTGCTCGCTTCAGTGGCAGTGGATCTGGGACCTCTTACTCTCTCACAATCAGCCGAATGGAGGCTGAAGATGCTGCCACTTATTACTGCCAGCAAAGGAGTAGTTACCCACTCA
IGKV4-57*01_S9219_DBA2	DBA/2J	IGKV	MUSMUS IGKV4-57-1*01 F	100	CAAATTGTTCTCACCCAGTCTCCAGCAATCATGTCTGCATCTCCAGGGGAGAAGGTCACCATAACCTGCAGTGCCAGCTCAAGTGTAAGTTACATGCACTGGTTCCAGCAGAAGCCAGGCACTTCTCCCAAACTCTGGATTTATAGCACATCCAACCTGGCTTCTGGAGTCCCTGCTCGCTTCAGTGGCAGTGGATCTGGGACCTCTTACTCTCTCACAATCAGCCGAATGGAGGCTGAAGATGCTGCCACTTATTACTGCCAGCAAAGGAGTAGTTACCCACTCA
IGKV4-57*01_S9219_LEWES	LEWES/EIJ	IGKV	MUSMUS IGKV4-57-1*01 F	100	CAAATTGTTCTCACCCAGTCTCCAGCAATCATGTCTGCATCTCCAGGGGAGAAGGTCACCATAACCTGCAGTGCCAGCTCAAGTGTAAGTTACATGCACTGGTTCCAGCAGAAGCCAGGCACTTCTCCCAAACTCTGGATTTATAGCACATCCAACCTGGCTTCTGGAGTCCCTGCTCGCTTCAGTGGCAGTGGATCTGGGACCTCTTACTCTCTCACAATCAGCCGAATGGAGGCTGAAGATGCTGCCACTTATTACTGCCAGCAAAGGAGTAGTTACCCACTCA
IGKV4-57*01_S9219_NZB	NZB/BLNJ	IGKV	MUSMUS IGKV4-57-1*01 F	100	CAAATTGTTCTCACCCAGTCTCCAGCAATCATGTCTGCATCTCCAGGGGAGAAGGTCACCATAACCTGCAGTGCCAGCTCAAGTGTAAGTTACATGCACTGGTTCCAGCAGAAGCCAGGCACTTCTCCCAAACTCTGGATTTATAGCACATCCAACCTGGCTTCTGGAGTCCCTGCTCGCTTCAGTGGCAGTGGATCTGGGACCTCTTACTCTCTCACAATCAGCCGAATGGAGGCTGAAGATGCTGCCACTTATTACTGCCAGCAAAGGAGTAGTTACCCACTCA

IGKV4-57*01_S9219_SJL	SJL/J	IGKV	MUSMUS IGKV4-57-1*01 F	100	CAAATTGTTCTCACCCAGTCTCCAGCAATCATGTCTGCATCTCCAGGGGAGAAGGTCACCATAACCTGCAGTGCCAGCTCAAGTGTAAGTTACATGCACTGGTTCCAGCAGAAGCCAGGCACTTCTCCCAAACTCTGGATTTATAGCACATCCAACCTGGCTTCTGGAGTCCCTGCTCGCTTCAGTGGCAGTGGATCTGGGACCTCTTACTCTCTCACAATCAGCCGAATGGAGGCTGAAGATGCTGCCACTTATTACTGCCAGCAAAGGAGTAGTTACCCACTCA
IGKV4-58*01_129	129S1/SVIMJ	IGKV	MUSMUS IGKV4-58*01 F	100	GAAAATGTGCTCACCCAGTCTCCAGCAATAATGGCTGCCTCTCTGGGGCAGAAGGTCACCATGACCTGCAGTGCCAGCTCAAGTGTAAGTTCCAGTTACTTGCACTGGTACCAGCAGAAGTCAGGCGCTTCCCCCAAACCCTTGATTCATAGGACATCCAACCTGGCTTCTGGAGTCCCAGCTCGCTTCAGTGGCAGTGGGTCTGGGACCTCTTACTCTCTCACAATCAGCAGCGTGGAGGCTGAAGATGATGCAACTTATTACTGCCAGCAGTGGAGTGGTTACCCATTCA
IGKV4-58*01_AJ	A/J	IGKV	MUSMUS IGKV4-58*01 F	100	GAAAATGTGCTCACCCAGTCTCCAGCAATAATGGCTGCCTCTCTGGGGCAGAAGGTCACCATGACCTGCAGTGCCAGCTCAAGTGTAAGTTCCAGTTACTTGCACTGGTACCAGCAGAAGTCAGGCGCTTCCCCCAAACCCTTGATTCATAGGACATCCAACCTGGCTTCTGGAGTCCCAGCTCGCTTCAGTGGCAGTGGGTCTGGGACCTCTTACTCTCTCACAATCAGCAGCGTGGAGGCTGAAGATGATGCAACTTATTACTGCCAGCAGTGGAGTGGTTACCCATTCA
IGKV4-58*01_B6	C57BL/6J	IGKV	MUSMUS IGKV4-58*01 F	100	GAAAATGTGCTCACCCAGTCTCCAGCAATAATGGCTGCCTCTCTGGGGCAGAAGGTCACCATGACCTGCAGTGCCAGCTCAAGTGTAAGTTCCAGTTACTTGCACTGGTACCAGCAGAAGTCAGGCGCTTCCCCCAAACCCTTGATTCATAGGACATCCAACCTGGCTTCTGGAGTCCCAGCTCGCTTCAGTGGCAGTGGGTCTGGGACCTCTTACTCTCTCACAATCAGCAGCGTGGAGGCTGAAGATGATGCAACTTATTACTGCCAGCAGTGGAGTGGTTACCCATTCA

IGKV4-58*01_BALB	BALB/CBYJ	IGKV	MUSMUS IGKV4-58*01 F	100	GAAAATGTGCTCACCCAGTCTCCAGCAATAATGGCTGCCTCTCTGGGGCAGAAGGTCACCATGACCTGCAGTGCCAGCTCAAGTGTAAGTTCCAGTTACTTGCACTGGTACCAGCAGAAGTCAGGCGCTTCCCCCAAACCCTTGATTCATAGGACATCCAACCTGGCTTCTGGAGTCCCAGCTCGCTTCAGTGGCAGTGGGTCTGGGACCTCTTACTCTCTCACAATCAGCAGCGTGGAGGCTGAAGATGATGCAACTTATTACTGCCAGCAGTGGAGTGGTTACCCATTCA
IGKV4-58*01_C3H	C3H/HEJ	IGKV	MUSMUS IGKV4-58*01 F	100	GAAAATGTGCTCACCCAGTCTCCAGCAATAATGGCTGCCTCTCTGGGGCAGAAGGTCACCATGACCTGCAGTGCCAGCTCAAGTGTAAGTTCCAGTTACTTGCACTGGTACCAGCAGAAGTCAGGCGCTTCCCCCAAACCCTTGATTCATAGGACATCCAACCTGGCTTCTGGAGTCCCAGCTCGCTTCAGTGGCAGTGGGTCTGGGACCTCTTACTCTCTCACAATCAGCAGCGTGGAGGCTGAAGATGATGCAACTTATTACTGCCAGCAGTGGAGTGGTTACCCATTCA
IGKV4-58*01_CBA	CBA/J	IGKV	MUSMUS IGKV4-58*01 F	100	GAAAATGTGCTCACCCAGTCTCCAGCAATAATGGCTGCCTCTCTGGGGCAGAAGGTCACCATGACCTGCAGTGCCAGCTCAAGTGTAAGTTCCAGTTACTTGCACTGGTACCAGCAGAAGTCAGGCGCTTCCCCCAAACCCTTGATTCATAGGACATCCAACCTGGCTTCTGGAGTCCCAGCTCGCTTCAGTGGCAGTGGGTCTGGGACCTCTTACTCTCTCACAATCAGCAGCGTGGAGGCTGAAGATGATGCAACTTATTACTGCCAGCAGTGGAGTGGTTACCCATTCA
IGKV4-58*01_DBA1	DBA/1J	IGKV	MUSMUS IGKV4-58*01 F	100	GAAAATGTGCTCACCCAGTCTCCAGCAATAATGGCTGCCTCTCTGGGGCAGAAGGTCACCATGACCTGCAGTGCCAGCTCAAGTGTAAGTTCCAGTTACTTGCACTGGTACCAGCAGAAGTCAGGCGCTTCCCCCAAACCCTTGATTCATAGGACATCCAACCTGGCTTCTGGAGTCCCAGCTCGCTTCAGTGGCAGTGGGTCTGGGACCTCTTACTCTCTCACAATCAGCAGCGTGGAGGCTGAAGATGATGCAACTTATTACTGCCAGCAGTGGAGTGGTTACCCATTCA

IGKV4-58*01_DBA2	DBA/2J	IGKV	MUSMUS IGKV4-58*01 F	100	GAAAATGTGCTCACCCAGTCTCCAGCAATAATGGCTGCCTCTCTGGGGCAGAAGGTCACCATGACCTGCAGTGCCAGCTCAAGTGTAAGTTCCAGTTACTTGCACTGGTACCAGCAGAAGTCAGGCGCTTCCCCCAAACCCTTGATTCATAGGACATCCAACCTGGCTTCTGGAGTCCCAGCTCGCTTCAGTGGCAGTGGGTCTGGGACCTCTTACTCTCTCACAATCAGCAGCGTGGAGGCTGAAGATGATGCAACTTATTACTGCCAGCAGTGGAGTGGTTACCCATTCA
IGKV4-58*01_LEWES	LEWES/EIJ	IGKV	MUSMUS IGKV4-58*01 F	100	GAAAATGTGCTCACCCAGTCTCCAGCAATAATGGCTGCCTCTCTGGGGCAGAAGGTCACCATGACCTGCAGTGCCAGCTCAAGTGTAAGTTCCAGTTACTTGCACTGGTACCAGCAGAAGTCAGGCGCTTCCCCCAAACCCTTGATTCATAGGACATCCAACCTGGCTTCTGGAGTCCCAGCTCGCTTCAGTGGCAGTGGGTCTGGGACCTCTTACTCTCTCACAATCAGCAGCGTGGAGGCTGAAGATGATGCAACTTATTACTGCCAGCAGTGGAGTGGTTACCCATTCA
IGKV4-58*01_NZB	NZB/BLNJ	IGKV	MUSMUS IGKV4-58*01 F	100	GAAAATGTGCTCACCCAGTCTCCAGCAATAATGGCTGCCTCTCTGGGGCAGAAGGTCACCATGACCTGCAGTGCCAGCTCAAGTGTAAGTTCCAGTTACTTGCACTGGTACCAGCAGAAGTCAGGCGCTTCCCCCAAACCCTTGATTCATAGGACATCCAACCTGGCTTCTGGAGTCCCAGCTCGCTTCAGTGGCAGTGGGTCTGGGACCTCTTACTCTCTCACAATCAGCAGCGTGGAGGCTGAAGATGATGCAACTTATTACTGCCAGCAGTGGAGTGGTTACCCATTCA
IGKV4-58*01_S3374_CAST	CAST/EIJ	IGKV	MUSMUS IGKV4-58*01 F	99.29	GAAAATGTGCTCACCCAGTCTCCAGCAATAATGGCTGCCTCTCTGGGGGAGAAGGTCACCATGACCTGCAGTGCCAGCTCAAGTGTAAGTTCCAGTTACTTGCACTGGTACCAGCAGAAGTCAGGCGCTTCCCCCAAACCCTTGATTCATAGGACATCCAACCTGGCTTCTGGTGTCCCAGCTCGCTTCAGTGGCAGTGGGTCTGGGACCTCTTACTCTCTCACAATCAGCAGCGTGGAGGCTGAAGATGATGCAACTTATTACTGCCAGCAGTGGAGTGGTTACCCATTCA

IGKV4-59*01_129	129S1/SVIMJ	IGKV	MUSMUS IGKV4-59*01 F	100	CAAATTGTTCTCACCCAGTCTCCAGCAATCATGTCTGCATCTCCAGGGGAGAAGGTCACCATGACCTGCAGTGCCAGCTCAAGTGTAAGTTACATGCACTGGTACCAGCAGAAGTCAGGCACCTCCCCCAAAAGATGGATTTATGACACATCCAAACTGGCTTCTGGAGTCCCTGCTCGCTTCAGTGGCAGTGGGTCTGGGACCTCTTACTCTCTCACAATCAGCAGCATGGAGGCTGAAGATGCTGCCACTTATTACTGCCAGCAGTGGAGTAGTAACCCACCCA
IGKV4-59*01_AJ	A/J	IGKV	MUSMUS IGKV4-59*01 F	100	CAAATTGTTCTCACCCAGTCTCCAGCAATCATGTCTGCATCTCCAGGGGAGAAGGTCACCATGACCTGCAGTGCCAGCTCAAGTGTAAGTTACATGCACTGGTACCAGCAGAAGTCAGGCACCTCCCCCAAAAGATGGATTTATGACACATCCAAACTGGCTTCTGGAGTCCCTGCTCGCTTCAGTGGCAGTGGGTCTGGGACCTCTTACTCTCTCACAATCAGCAGCATGGAGGCTGAAGATGCTGCCACTTATTACTGCCAGCAGTGGAGTAGTAACCCACCCA
IGKV4-59*01_B6	C57BL/6J	IGKV	MUSMUS IGKV4-59*01 F	100	CAAATTGTTCTCACCCAGTCTCCAGCAATCATGTCTGCATCTCCAGGGGAGAAGGTCACCATGACCTGCAGTGCCAGCTCAAGTGTAAGTTACATGCACTGGTACCAGCAGAAGTCAGGCACCTCCCCCAAAAGATGGATTTATGACACATCCAAACTGGCTTCTGGAGTCCCTGCTCGCTTCAGTGGCAGTGGGTCTGGGACCTCTTACTCTCTCACAATCAGCAGCATGGAGGCTGAAGATGCTGCCACTTATTACTGCCAGCAGTGGAGTAGTAACCCACCCA
IGKV4-59*01_BALB	BALB/CBYJ	IGKV	MUSMUS IGKV4-59*01 F	100	CAAATTGTTCTCACCCAGTCTCCAGCAATCATGTCTGCATCTCCAGGGGAGAAGGTCACCATGACCTGCAGTGCCAGCTCAAGTGTAAGTTACATGCACTGGTACCAGCAGAAGTCAGGCACCTCCCCCAAAAGATGGATTTATGACACATCCAAACTGGCTTCTGGAGTCCCTGCTCGCTTCAGTGGCAGTGGGTCTGGGACCTCTTACTCTCTCACAATCAGCAGCATGGAGGCTGAAGATGCTGCCACTTATTACTGCCAGCAGTGGAGTAGTAACCCACCCA

IGKV4-59*01_C3H	C3H/HEJ	IGKV	MUSMUS IGKV4-59*01 F	100	CAAATTGTTCTCACCCAGTCTCCAGCAATCATGTCTGCATCTCCAGGGGAGAAGGTCACCATGACCTGCAGTGCCAGCTCAAGTGTAAGTTACATGCACTGGTACCAGCAGAAGTCAGGCACCTCCCCCAAAAGATGGATTTATGACACATCCAAACTGGCTTCTGGAGTCCCTGCTCGCTTCAGTGGCAGTGGGTCTGGGACCTCTTACTCTCTCACAATCAGCAGCATGGAGGCTGAAGATGCTGCCACTTATTACTGCCAGCAGTGGAGTAGTAACCCACCCA
IGKV4-59*01_CBA	CBA/J	IGKV	MUSMUS IGKV4-59*01 F	100	CAAATTGTTCTCACCCAGTCTCCAGCAATCATGTCTGCATCTCCAGGGGAGAAGGTCACCATGACCTGCAGTGCCAGCTCAAGTGTAAGTTACATGCACTGGTACCAGCAGAAGTCAGGCACCTCCCCCAAAAGATGGATTTATGACACATCCAAACTGGCTTCTGGAGTCCCTGCTCGCTTCAGTGGCAGTGGGTCTGGGACCTCTTACTCTCTCACAATCAGCAGCATGGAGGCTGAAGATGCTGCCACTTATTACTGCCAGCAGTGGAGTAGTAACCCACCCA
IGKV4-59*01_DBA1	DBA/1J	IGKV	MUSMUS IGKV4-59*01 F	100	CAAATTGTTCTCACCCAGTCTCCAGCAATCATGTCTGCATCTCCAGGGGAGAAGGTCACCATGACCTGCAGTGCCAGCTCAAGTGTAAGTTACATGCACTGGTACCAGCAGAAGTCAGGCACCTCCCCCAAAAGATGGATTTATGACACATCCAAACTGGCTTCTGGAGTCCCTGCTCGCTTCAGTGGCAGTGGGTCTGGGACCTCTTACTCTCTCACAATCAGCAGCATGGAGGCTGAAGATGCTGCCACTTATTACTGCCAGCAGTGGAGTAGTAACCCACCCA
IGKV4-59*01_DBA2	DBA/2J	IGKV	MUSMUS IGKV4-59*01 F	100	CAAATTGTTCTCACCCAGTCTCCAGCAATCATGTCTGCATCTCCAGGGGAGAAGGTCACCATGACCTGCAGTGCCAGCTCAAGTGTAAGTTACATGCACTGGTACCAGCAGAAGTCAGGCACCTCCCCCAAAAGATGGATTTATGACACATCCAAACTGGCTTCTGGAGTCCCTGCTCGCTTCAGTGGCAGTGGGTCTGGGACCTCTTACTCTCTCACAATCAGCAGCATGGAGGCTGAAGATGCTGCCACTTATTACTGCCAGCAGTGGAGTAGTAACCCACCCA

IGKV4-59*01_LEWES	LEWES/EIJ	IGKV	MUSMUS IGKV4-59*01 F	100	CAAATTGTTCTCACCCAGTCTCCAGCAATCATGTCTGCATCTCCAGGGGAGAAGGTCACCATGACCTGCAGTGCCAGCTCAAGTGTAAGTTACATGCACTGGTACCAGCAGAAGTCAGGCACCTCCCCCAAAAGATGGATTTATGACACATCCAAACTGGCTTCTGGAGTCCCTGCTCGCTTCAGTGGCAGTGGGTCTGGGACCTCTTACTCTCTCACAATCAGCAGCATGGAGGCTGAAGATGCTGCCACTTATTACTGCCAGCAGTGGAGTAGTAACCCACCCA
IGKV4-59*01_NZB	NZB/BLNJ	IGKV	MUSMUS IGKV4-59*01 F	100	CAAATTGTTCTCACCCAGTCTCCAGCAATCATGTCTGCATCTCCAGGGGAGAAGGTCACCATGACCTGCAGTGCCAGCTCAAGTGTAAGTTACATGCACTGGTACCAGCAGAAGTCAGGCACCTCCCCCAAAAGATGGATTTATGACACATCCAAACTGGCTTCTGGAGTCCCTGCTCGCTTCAGTGGCAGTGGGTCTGGGACCTCTTACTCTCTCACAATCAGCAGCATGGAGGCTGAAGATGCTGCCACTTATTACTGCCAGCAGTGGAGTAGTAACCCACCCA
IGKV4-59*01_S1025_AKR	AKR/J	IGKV	MUSMUS IGKV4-59*01 F	98.19	CAAATTGTTCTCACCCAGTCTCCAGCAATCATGTCTGCATCTCCAGGGGAGAAGGTCACCATGACATGCAGTGCCAGCTCAAGTGTAAGTTACATGCACTGGTACCAGCAGAAGTCAGGCACCTCCCCCAAAAGATGGATTTATGACACATCCAAACTGGATTCTGGAGTCCCTGCTCGCTTCAGTGGCAGTGGGTCTGGGACCTCTTATTCTCTCACAATCAGCAGCATGGAGGCTGAAGATGCTGCCACTTATTACTGCTATCAGTGGAGTAGTTACCCACCCA
IGKV4-59*01_S1025_NOD	NOD/SHILTJ	IGKV	MUSMUS IGKV4-59*01 F	98.19	CAAATTGTTCTCACCCAGTCTCCAGCAATCATGTCTGCATCTCCAGGGGAGAAGGTCACCATGACATGCAGTGCCAGCTCAAGTGTAAGTTACATGCACTGGTACCAGCAGAAGTCAGGCACCTCCCCCAAAAGATGGATTTATGACACATCCAAACTGGATTCTGGAGTCCCTGCTCGCTTCAGTGGCAGTGGGTCTGGGACCTCTTATTCTCTCACAATCAGCAGCATGGAGGCTGAAGATGCTGCCACTTATTACTGCTATCAGTGGAGTAGTTACCCACCCA

IGKV4-59*01_S3253_CAST	CAST/EIJ	IGKV	MUSMUS IGKV4-59*01 F	100	CAAATTGTTCTCACCCAGTCTCCAGCAATCATGTCTGCATCTCCAGGGGAGAAGGTCACCATGACCTGCAGTGCCAGCTCAAGTGTAAGTTACATGCACTGGTACCAGCAGAAGTCAGGCACCTCCCCCAAAAGATGGATTTATGACACATCCAAACTGGCTTCTGGAGTCCCTGCTCGCTTCAGTGGCAGTGGGTCTGGGACCTCTTACTCTCTCACAATCAGCAGCATGGAGGCTGAAGATGCTGCCACTTATTACTGCCAGCAGTGGAGTAGTTACCCACTCA
IGKV4-59*01_S3489_MRL	MRL/MPJ	IGKV	MUSMUS IGKV4-59*01 F	98.19	CAAATTGTTCTCACCCAGTCTCCAGCAATCATGTCTGCATCTCCAGGGGAGAAGGTCACCATGACATGCAGTGCCAGCTCAAGTGTAAGTTACATGCACTGGTACCAGCAGAAGTCAGGCACCTCCCCCAAAAGATGGATTTATGACACATCCAAACTGGATTCTGGAGTCCCTGCTCGCTTCAGTGGCAGTGGGTCTGGGACCTCTTATTCTCTCACAATCAGCAGCATGGAGGCTGAAGATGCTGCCACTTATTACTGCTATCAGTGGAGTAGTTACCCACTCA
IGKV4-59*01_S4622_CBA	CBA/J	IGKV	MUSMUS IGKV4-59*01 F	100	CAAATTGTTCTCACCCAGTCTCCAGCAATCATGTCTGCATCTCCAGGGGAGAAGGTCACCATGACCTGCAGTGCCAGCTCAAGTGTAAGTTACATGCACTGGTACCAGCAGAAGTCAGGCACCTCCCCCAAAAGATGGATTTATGACACATCCAAACTGGCTTCTGGAGTCCCTGCTCGCTTCAGTGGCAGTGGGTCTGGGACCTCTTACTCTCTCACAATCAGCAGCATGGAGGCTGAAGATGCTGCCACTTATTACTGCCAGCAGTGGAGTAGTAACCCACTCA
IGKV4-59*01_S5176_MSM	MSM/MSJ	IGKV	MUSMUS IGKV4-59*01 F	97.1	CAAATTGTTCTCACCCAGTCTCCAGCAATCATGTCTGCATCTCCAGGGGAGAAGGTCACCATGACCTGCAGTGCCAGCTCAAGTGTAAGTTACATGTACTGGTACCAGCAGAAGCCAGGATCCTCCCCCAAAAAATGGATTTATGACACATCCAACCTGGCTTCTGGAGTCCCCGCTCGCTTCAGTGGCAGTGGGTCTGGGACCTCTTATTCTCTCACAATCAGCAGCATGGAGGCTGAAGATGCTGCCACTTATTACTGCCAGCAGTGGAGTAGTTACCCATCCA

IGKV4-59*01_S5176_PWD	PWD/PHJ	IGKV	MUSMUS IGKV4-59*01 F	97.1	CAAATTGTTCTCACCCAGTCTCCAGCAATCATGTCTGCATCTCCAGGGGAGAAGGTCACCATGACCTGCAGTGCCAGCTCAAGTGTAAGTTACATGTACTGGTACCAGCAGAAGCCAGGATCCTCCCCCAAAAAATGGATTTATGACACATCCAACCTGGCTTCTGGAGTCCCCGCTCGCTTCAGTGGCAGTGGGTCTGGGACCTCTTATTCTCTCACAATCAGCAGCATGGAGGCTGAAGATGCTGCCACTTATTACTGCCAGCAGTGGAGTAGTTACCCATCCA
IGKV4-59*01_S5858_MSM	MSM/MSJ	IGKV	MUSMUS IGKV4-59*01 F	97.1	CAAATTGTTCTCACCCAGTCTCCAGCAATCATGTCTGCATCTCCAGGGGAGAAGGTCACCATGACCTGCAGTGCCAGCTCAAGTGTAAGTTACATGTACTGGTACCAGCAGAAGCCAGGATCCTCCCCCAAAAAATGGATTTATGACACATCCAACCTGGCTTCTGGAGTCCCCGCTCGCTTCAGTGGCAGTGGGTCTGGGACCTCTTATTCTCTCACAATCAGCAGCATGGAGGCTGAAGATGCTGCCACTTATTACTGCCAGCAGTGGAGTAGTTACCCACCCA
IGKV4-59*01_S5858A_PWD	PWD/PHJ	IGKV	MUSMUS IGKV4-59*01 F	97.1	CAAATTGTTCTCACCCAGTCTCCAGCAATCATGTCTGCATCTCCAGGGGAGAAGGTCACCATGACCTGCAGTGCCAGCTCAAGTGTAAGTTACATGTACTGGTACCAGCAGAAGCCAGGATCCTCCCCCAAAAAATGGATTTATGACACATCCAACCTGGCTTCTGGAGTCCCCGCTCGCTTCAGTGGCAGTGGGTCTGGGACCTCTTATTCTCTCACAATCAGCAGCATGGAGGCTGAAGATGCTGCCACTTATTACTGCCAGCAGTGGAGTAGTTACCCACCCA
IGKV4-59*01_S6619_MSM	MSM/MSJ	IGKV	MUSMUS IGKV4-59*01 F	97.46	CAAATTGTTCTCACCCAGTCTCCAGCAATCATGTCTGCATCTCCAGGGGAGAAGGTCACCATGACATGCAGTGCCAGCTCAAATGTAAATTATATGCACTGGTACCAGCAGAAGTCAGGCACCTCCCCCAAAAGATGGATTTATGACACATCCAAACTGGATTCTGGAGTCCCTGCTTGCTTCAGTGGCAGTGGGTCTGGGACCTCTTATTCTCTCACAATCAGCAGCATGGAGGCTGAAGATGCTGCCACTTATTACTGCCAGCAGTGGAGTAGTAACCCACCCA

IGKV4-59*01_S9913_NOR	NOR/LTJ	IGKV	MUSMUS IGKV4-59*01 F	98.19	CAAATTGTTCTCACCCAGTCTCCAGCAATCATGTCTGCATCTCCAGGGGAGAAGGTCACCATGACATGCAGTGCCAGCTCAAGTGTAAGTTACATGCACTGGTACCAGCAGAAGTCAGGCACCTCCCCCAAAAGATGGATTTATGACACATCCAAACTGGATTCTGGAGTCCCTGCTCGCTTCAGTGGCAGTGGGTCTGGGACCTCTTATTCTCTCACAATCAGCAGCATGGAGGCTGAAGATGCTGCCACTTATTACTGCTATCAGTGGAGTAGTTACCCA
IGKV4-59*01_SJL	SJL/J	IGKV	MUSMUS IGKV4-59*01 F	100	CAAATTGTTCTCACCCAGTCTCCAGCAATCATGTCTGCATCTCCAGGGGAGAAGGTCACCATGACCTGCAGTGCCAGCTCAAGTGTAAGTTACATGCACTGGTACCAGCAGAAGTCAGGCACCTCCCCCAAAAGATGGATTTATGACACATCCAACTGGCTTCTGGAGTCCCTGCTCGCTTCAGTGGCAGTGGGTCTGGGACCTCTTACTCTCTCACAATCAGCAGCATGGAGGCTGAAGATGCTGCCACTTATTACTGCCAGCAGTGGAGTAGTAACCCACCCA
IGKV4-60*01_BALB	BALB/CBYJ	IGKV	MUSMUS IGKV4-60*01 P	100	GAAATTGTGCTCACCCAGTCTCCAGCACTCATGGCTGCATCTCCAGGGGAGAAGGTCACCATCACCTGCAGTGTCAGCTCAAGTATAAGTTCCAGCTACTTGCACTGGTACCAGCAGAAGTCAGGAATCTCCCCCAAACCCTGGATTTATGGCACATCCAACCTGGCTTCTGGAGTCCCTGCTCGCTTCAGTGGCAGTGGATCTGGGACCTCTTACTCTCTCACAATCAGCAGCATGGAGGCTGAAGATGCTGCCACTTATTACTGTCAACAGTGGAGTAGTTCCCCACCCA
IGKV4-60*01_DBA2	DBA/2J	IGKV	MUSMUS IGKV4-60*01 P	100	GAAATTGTGCTCACCCAGTCTCCAGCACTCATGGCTGCATCTCCAGGGGAGAAGGTCACCATCACCTGCAGTGTCAGCTCAAGTATAAGTTCCAGCTACTTGCACTGGTACCAGCAGAAGTCAGGAATCTCCCCCAAACCCTGGATTTATGGCACATCCAACCTGGCTTCTGGAGTCCCTGCTCGCTTCAGTGGCAGTGGATCTGGGACCTCTTACTCTCTCACAATCAGCAGCATGGAGGCTGAAGATGCTGCCACTTATTACTGTCAACAGTGGAGTAGTTCCCCACCCA

IGKV4-60*01_NZB	NZB/BLNJ	IGKV	MUSMUS IGKV4-60*01 P	100	GAAATTGTGCTCACCCAGTCTCCAGCACTCATGGCTGCATCTCCAGGGGAGAAGGTCACCATCACCTGCAGTGTCAGCTCAAGTATAAGTTCCAGCTACTTGCACTGGTACCAGCAGAAGTCAGGAATCTCCCCCAAACCCTGGATTTATGGCACATCCAACCTGGCTTCTGGAGTCCCTGCTCGCTTCAGTGGCAGTGGATCTGGGACCTCTTACTCTCTCACAATCAGCAGCATGGAGGCTGAAGATGCTGCCACTTATTACTGTCAACAGTGGAGTAGTTCCCCACCCA
IGKV4-60*01_S1112_AJ	A/J	IGKV	MUSMUS IGKV4-60*01 P	100	GAAATTGTGCTCACCCAGTCTCCAGCACTCATGGCTGCATCTCCAGGGGAGAAGGTCACCATCACCTGCAGTGTCAGCTCAAGTATAAGTTCCAGCTACTTGCACTGGTACCAGCAGAAGTCAGGAATCTCCCCCAAACCCTGGATTTATGGCACATCCAACCTGGCTTCTGGAGTCCCTGCTCGCTTCAGTGGCAGTGGATCTGGGACCTCTTACTCTCTCACAATCAGCAGCATGGAGGCTGAAGATGCTGCCACTTATTACTGTCAACAGTGGAGTAGTTCCCCACTCA
IGKV4-60*01_S1112_B6	C57BL/6J	IGKV	MUSMUS IGKV4-60*01 P	100	GAAATTGTGCTCACCCAGTCTCCAGCACTCATGGCTGCATCTCCAGGGGAGAAGGTCACCATCACCTGCAGTGTCAGCTCAAGTATAAGTTCCAGCTACTTGCACTGGTACCAGCAGAAGTCAGGAATCTCCCCCAAACCCTGGATTTATGGCACATCCAACCTGGCTTCTGGAGTCCCTGCTCGCTTCAGTGGCAGTGGATCTGGGACCTCTTACTCTCTCACAATCAGCAGCATGGAGGCTGAAGATGCTGCCACTTATTACTGTCAACAGTGGAGTAGTTCCCCACTCA
IGKV4-60*01_S1112_C3H	C3H/HEJ	IGKV	MUSMUS IGKV4-60*01 P	100	GAAATTGTGCTCACCCAGTCTCCAGCACTCATGGCTGCATCTCCAGGGGAGAAGGTCACCATCACCTGCAGTGTCAGCTCAAGTATAAGTTCCAGCTACTTGCACTGGTACCAGCAGAAGTCAGGAATCTCCCCCAAACCCTGGATTTATGGCACATCCAACCTGGCTTCTGGAGTCCCTGCTCGCTTCAGTGGCAGTGGATCTGGGACCTCTTACTCTCTCACAATCAGCAGCATGGAGGCTGAAGATGCTGCCACTTATTACTGTCAACAGTGGAGTAGTTCCCCACTCA

IGKV4-60*01_S2188_MSM	MSM/MSJ	IGKV	MUSMUS IGKV4-60*01 P	98.58	GAAATTGTGCTCACCCAGTCTCCAGCACTCATGGCTGCATCTCCAGGGGAGAAGGTCAGCATCACCTGCAGTGTCAGCTCAAGTATAAGTTCCAGCCACTTGCACTGGTACCAGCAGAAGTCAGGAACCTCCCCCAAACTCTGGATTTATGGCACATCCAACCTGGCTTCTGGAGTCCCTGCTCGCTTCAGTGGCAGTGGATCTGGGACCTCTTACTCTCTCACAATCAGCAGCATGGAGGCTGAAGATGCTGCCACTTATTACTGTCAACAGTGGAGTAGTTACCCAACCA
IGKV4-60*01_S2341_PWD	PWD/PHJ	IGKV	MUSMUS IGKV4-60*01 P	98.23	GAAATTGTGCTCACCCAGTCTCCAGCACTCATGGCTGCATCTCCAGGGGAGAAGGTCAGCATCACCTGCAGTGTCAGCTCAAGTATAAGTTCCAGCCACTTACACTGGTACCAGCAGAAGTCAGGAACCTCCCCCAAACTCTGGATTTATGGCACATCCAACCTGGCTTCTGGAGTCCCTGCTCGCTTCAGTGGCAGTGGATCTGGGACCTCTTACTCTCTCACAATCAGCAGCATGGAGGCTGAAGATGCTGCCACTTATTACTGTCAACAGTGGAGTAGTTACCCACCCA
IGKV4-61*01_B6	C57BL/6J	IGKV	MUSMUS IGKV4-61*01 F	100	CAAATTGTTCTCACCCAGTCTCCAGCAATCATGTCTGCATCTCCAGGGGAGAAGGTCACCATATCCTGCAGTGCCAGCTCAAGTGTAAGTTACATGTACTGGTACCAGCAGAAGCCAGGATCCTCCCCCAAACCCTGGATTTATCGCACATCCAACCTGGCTTCTGGAGTCCCTGCTCGCTTCAGTGGCAGTGGGTCTGGGACCTCTTACTCTCTCACAATCAGCAGCATGGAGGCTGAAGATGCTGCCACTTATTACTGCCAGCAGTATCATAGTTACCCACCCA
IGKV4-61*01_BALB	BALB/CBYJ	IGKV	MUSMUS IGKV4-61*01 F	100	CAAATTGTTCTCACCCAGTCTCCAGCAATCATGTCTGCATCTCCAGGGGAGAAGGTCACCATATCCTGCAGTGCCAGCTCAAGTGTAAGTTACATGTACTGGTACCAGCAGAAGCCAGGATCCTCCCCCAAACCCTGGATTTATCGCACATCCAACCTGGCTTCTGGAGTCCCTGCTCGCTTCAGTGGCAGTGGGTCTGGGACCTCTTACTCTCTCACAATCAGCAGCATGGAGGCTGAAGATGCTGCCACTTATTACTGCCAGCAGTATCATAGTTACCCACCCA

IGKV4-61*01_DBA1	DBA/1J	IGKV	MUSMUS IGKV4-61*01 F	100	CAAATTGTTCTCACCCAGTCTCCAGCAATCATGTCTGCATCTCCAGGGGAGAAGGTCACCATATCCTGCAGTGCCAGCTCAAGTGTAAGTTACATGTACTGGTACCAGCAGAAGCCAGGATCCTCCCCCAAACCCTGGATTTATCGCACATCCAACCTGGCTTCTGGAGTCCCTGCTCGCTTCAGTGGCAGTGGGTCTGGGACCTCTTACTCTCTCACAATCAGCAGCATGGAGGCTGAAGATGCTGCCACTTATTACTGCCAGCAGTATCATAGTTACCCACCCA
IGKV4-61*01_DBA2	DBA/2J	IGKV	MUSMUS IGKV4-61*01 F	100	CAAATTGTTCTCACCCAGTCTCCAGCAATCATGTCTGCATCTCCAGGGGAGAAGGTCACCATATCCTGCAGTGCCAGCTCAAGTGTAAGTTACATGTACTGGTACCAGCAGAAGCCAGGATCCTCCCCCAAACCCTGGATTTATCGCACATCCAACCTGGCTTCTGGAGTCCCTGCTCGCTTCAGTGGCAGTGGGTCTGGGACCTCTTACTCTCTCACAATCAGCAGCATGGAGGCTGAAGATGCTGCCACTTATTACTGCCAGCAGTATCATAGTTACCCACCCA
IGKV4-61*01_LEWES	LEWES/EIJ	IGKV	MUSMUS IGKV4-61*01 F	100	CAAATTGTTCTCACCCAGTCTCCAGCAATCATGTCTGCATCTCCAGGGGAGAAGGTCACCATATCCTGCAGTGCCAGCTCAAGTGTAAGTTACATGTACTGGTACCAGCAGAAGCCAGGATCCTCCCCCAAACCCTGGATTTATCGCACATCCAACCTGGCTTCTGGAGTCCCTGCTCGCTTCAGTGGCAGTGGGTCTGGGACCTCTTACTCTCTCACAATCAGCAGCATGGAGGCTGAAGATGCTGCCACTTATTACTGCCAGCAGTATCATAGTTACCCACCCA
IGKV4-61*01_NZB	NZB/BLNJ	IGKV	MUSMUS IGKV4-61*01 F	100	CAAATTGTTCTCACCCAGTCTCCAGCAATCATGTCTGCATCTCCAGGGGAGAAGGTCACCATATCCTGCAGTGCCAGCTCAAGTGTAAGTTACATGTACTGGTACCAGCAGAAGCCAGGATCCTCCCCCAAACCCTGGATTTATCGCACATCCAACCTGGCTTCTGGAGTCCCTGCTCGCTTCAGTGGCAGTGGGTCTGGGACCTCTTACTCTCTCACAATCAGCAGCATGGAGGCTGAAGATGCTGCCACTTATTACTGCCAGCAGTATCATAGTTACCCACCCA

IGKV4-61*01_S1531_MSM	MSM/MSJ	IGKV	MUSMUS IGKV4-61*01 F	99.64	CAAATTGTTCTCACCCAGTCTCCAGCAATCCTGTCTGCATCTCCAGGGGAGAAGGTCACCATATCCTGCAGTGCCAGCTCAAGTGTAAGTTACATGTACTGGTACCAGCAGAAGCCAGGATCCTCCCCCAAACCCTGGATTTATCGCACATCCAACCTGGCTTCTGGAGTCCCTGCTCGCTTCAGTGGCAGTGGGTCTGGGACCTCTTACTCTCTCACAATCAGCAGCATGGAGGCTGAAGATGCTGCCACTTATTACTGCCAGCAGTATCATAGTTACCCACCCA
IGKV4-61*01_S1531_PWD	PWD/PHJ	IGKV	MUSMUS IGKV4-61*01 F	99.64	CAAATTGTTCTCACCCAGTCTCCAGCAATCCTGTCTGCATCTCCAGGGGAGAAGGTCACCATATCCTGCAGTGCCAGCTCAAGTGTAAGTTACATGTACTGGTACCAGCAGAAGCCAGGATCCTCCCCCAAACCCTGGATTTATCGCACATCCAACCTGGCTTCTGGAGTCCCTGCTCGCTTCAGTGGCAGTGGGTCTGGGACCTCTTACTCTCTCACAATCAGCAGCATGGAGGCTGAAGATGCTGCCACTTATTACTGCCAGCAGTATCATAGTTACCCACCCA
IGKV4-61*01_S2955_129	129S1/SVIMJ	IGKV	MUSMUS IGKV4-61*01 F	100	CAAATTGTTCTCACCCAGTCTCCAGCAATCATGTCTGCATCTCCAGGGGAGAAGGTCACCATATCCTGCAGTGCCAGCTCAAGTGTAAGTTACATGTACTGGTACCAGCAGAAGCCAGGATCCTCCCCCAAACCCTGGATTTATCGCACATCCAACCTGGCTTCTGGAGTCCCTGCTCGCTTCAGTGGCAGTGGGTCTGGGACCTCTTACTCTCTCACAATCAGCAGCATGGAGGCTGAAGATGCTGCCACTTATTACTGCCAGCAGTATCATAGTTACCCACTCA
IGKV4-61*01_S2955_AJ	A/J	IGKV	MUSMUS IGKV4-61*01 F	100	CAAATTGTTCTCACCCAGTCTCCAGCAATCATGTCTGCATCTCCAGGGGAGAAGGTCACCATATCCTGCAGTGCCAGCTCAAGTGTAAGTTACATGTACTGGTACCAGCAGAAGCCAGGATCCTCCCCCAAACCCTGGATTTATCGCACATCCAACCTGGCTTCTGGAGTCCCTGCTCGCTTCAGTGGCAGTGGGTCTGGGACCTCTTACTCTCTCACAATCAGCAGCATGGAGGCTGAAGATGCTGCCACTTATTACTGCCAGCAGTATCATAGTTACCCACTCA

IGKV4-61*01_S2955_B6	C57BL/6J	IGKV	MUSMUS IGKV4-61*01 F	100	CAAATTGTTCTCACCCAGTCTCCAGCAATCATGTCTGCATCTCCAGGGGAGAAGGTCACCATATCCTGCAGTGCCAGCTCAAGTGTAAGTTACATGTACTGGTACCAGCAGAAGCCAGGATCCTCCCCCAAACCCTGGATTTATCGCACATCCAACCTGGCTTCTGGAGTCCCTGCTCGCTTCAGTGGCAGTGGGTCTGGGACCTCTTACTCTCTCACAATCAGCAGCATGGAGGCTGAAGATGCTGCCACTTATTACTGCCAGCAGTATCATAGTTACCCACTCA
IGKV4-61*01_S2955_C3H	C3H/HEJ	IGKV	MUSMUS IGKV4-61*01 F	100	CAAATTGTTCTCACCCAGTCTCCAGCAATCATGTCTGCATCTCCAGGGGAGAAGGTCACCATATCCTGCAGTGCCAGCTCAAGTGTAAGTTACATGTACTGGTACCAGCAGAAGCCAGGATCCTCCCCCAAACCCTGGATTTATCGCACATCCAACCTGGCTTCTGGAGTCCCTGCTCGCTTCAGTGGCAGTGGGTCTGGGACCTCTTACTCTCTCACAATCAGCAGCATGGAGGCTGAAGATGCTGCCACTTATTACTGCCAGCAGTATCATAGTTACCCACTCA
IGKV4-61*01_S2955_CBA	CBA/J	IGKV	MUSMUS IGKV4-61*01 F	100	CAAATTGTTCTCACCCAGTCTCCAGCAATCATGTCTGCATCTCCAGGGGAGAAGGTCACCATATCCTGCAGTGCCAGCTCAAGTGTAAGTTACATGTACTGGTACCAGCAGAAGCCAGGATCCTCCCCCAAACCCTGGATTTATCGCACATCCAACCTGGCTTCTGGAGTCCCTGCTCGCTTCAGTGGCAGTGGGTCTGGGACCTCTTACTCTCTCACAATCAGCAGCATGGAGGCTGAAGATGCTGCCACTTATTACTGCCAGCAGTATCATAGTTACCCACTCA
IGKV4-61*01_S2955_DBA1	DBA/1J	IGKV	MUSMUS IGKV4-61*01 F	100	CAAATTGTTCTCACCCAGTCTCCAGCAATCATGTCTGCATCTCCAGGGGAGAAGGTCACCATATCCTGCAGTGCCAGCTCAAGTGTAAGTTACATGTACTGGTACCAGCAGAAGCCAGGATCCTCCCCCAAACCCTGGATTTATCGCACATCCAACCTGGCTTCTGGAGTCCCTGCTCGCTTCAGTGGCAGTGGGTCTGGGACCTCTTACTCTCTCACAATCAGCAGCATGGAGGCTGAAGATGCTGCCACTTATTACTGCCAGCAGTATCATAGTTACCCACTCA

IGKV4-61*01_S2955_NZB	NZB/BLNJ	IGKV	MUSMUS IGKV4-61*01 F	100	CAAATTGTTCTCACCCAGTCTCC<br>AGCAATCATGTCTGCATCTCCA<br>GGGGAGAAGGTCACCATATCCT<br>GCAGTGCCAGCTCAAGTGTAAG<br>TTACATGTACTGGTACCAGCAGA<br>AGCCAGGATCCTCCCCCAAAC<br>CCTGGATTTATCGCACATCCAA<br>CCTGGCTTCTGGAGTCCCTGCT<br>CGCTTCAGTGGCAGTGGGTCTG<br>GGACCTCTTACTCTCTCACAATC<br>AGCAGCATGGAGGCTGAAGAT<br>GCTGCCACTTATTACTGCCAGC<br>AGTATCATAGTTACCCACTCA
IGKV4-61*01_S4622_CAST	CAST/EIJ	IGKV	MUSMUS IGKV4-61*01 F	96.01	CAAATTGTTCTCACCCAGTCCC<br>CAGCAATCCTGTCTGCATCTCC<br>AGGGGAGAAGGTCACCATATCC<br>TGCAGTGCCAGCTCAAGTGTAA<br>ATTACATGTACTGGTACCAGCAG<br>AAGCCAGGATCCTCACCCAAA<br>CCCTGGATTTATCCCACATCCA<br>ACCTGGCTTCTGGAGTCCCTGC<br>TCGCTTCAATGGCAGTGGGTCT<br>GCGACCTCTTATTCTCTCACAAT<br>CAGCAGCATGGAGGCTGAAGAT<br>GCTGCCACTTATTACTGCCAGC<br>AGTTTACTAGTTCCCCATCCA
IGKV4-61*01_SJL	SJL/J	IGKV	MUSMUS IGKV4-61*01 F	100	CAAATTGTTCTCACCCAGTCTCC<br>AGCAATCATGTCTGCATCTCCA<br>GGGGAGAAGGTCACCATATCCT<br>GCAGTGCCAGCTCAAGTGTAAG<br>TTACATGTACTGGTACCAGCAGA<br>AGCCAGGATCCTCCCCCAAAC<br>CCTGGATTTATCGCACATCCAA<br>CCTGGCTTCTGGAGTCCCTGCT<br>CGCTTCAGTGGCAGTGGGTCTG<br>GGACCTCTTACTCTCTCACAATC<br>AGCAGCATGGAGGCTGAAGAT<br>GCTGCCACTTATTACTGCCAGC<br>AGTATCATAGTTACCCACCCA
IGKV4-62*01_BALB	BALB/CBYJ	IGKV	MUSMUS IGKV4-62*01 ORF	100	CAAATTGTTCTCACCCAGTCTCC<br>AGCAATCATGTCTGCATCTCCA<br>GGGGAAAAGGTCACCATGACCT<br>GCAGTGCCAGCTCAAGTGTAAG<br>TTACATGTACTGGTACCAGCAGA<br>AGCCAGGCTCCTCCCCCAGAC<br>TCTGGATTTATGACACATCCAAC<br>CTGGTTTCTGGAGTCCCTGCTC<br>GCTTCAGTGGCAGTAGGTCTGG<br>GACCTCTTATTCTCTCACAATCA<br>GCAGCATGGAGGCTGAAGATG<br>CTGCCACTTATTACTGCCAGCA<br>GTACAGTGGTTACCCATCCA

IGKV4-62*01_S0389_MSM	MSM/MSJ	IGKV	MUSMUS IGKV4-55*01 F	97.46	CAAATTGTTCTCACCCAGTCTCCAGCAATCATGTCTGCATCTCCAGGGGAGAAGGTCACCATGACCTGCAGTGCCAGCTCAAGTGTAAGTTACATGTACTGGTACCAGCAGAAGCCAGGATCCTCCCCCAGACTCTGGATTTATGACACATCCAACCTGGCTTCTGGAGTCCCCGCTCGCTTCAGTGGCAGTGGGTCTGGGACCTCTTATTCTCTCACAATCAGCAGCATGGAGGCTGAAGATGCTGCCACTTATTACTGCCAGCAGTGGAGTAGTAACCAATTCA
IGKV4-62*01_S2199_AKR	AKR/J	IGKV	MUSMUS IGKV4-62*01 ORF	96.74	CAAATTGTTCTCACCCAGTCTCCAGCAATCATGTCTGCATCTCCAGGGGAGAAGGTCACCATGACCTGCAGTGCCAGCTCAAGTGTAAGTTACATGTACTGGTACCAGCAGAAGCCAGGATCCTCCCCCAGACTCTGGATTTATGACACATCCAACCTGGCTTCTGGAGTCCCCGCTCGCTTCAGTGGCAGTAGGTCTGGGACCTCTTATTCTCTCACAATCAGCAGCATGGAGGCTGAAGATGCTGCCACTTATTACTGCCATCAGCGGAGTAGTTACCCATTCA
IGKV4-62*01_S2199_MRL	MRL/MPJ	IGKV	MUSMUS IGKV4-62*01 ORF	96.74	CAAATTGTTCTCACCCAGTCTCCAGCAATCATGTCTGCATCTCCAGGGGAGAAGGTCACCATGACCTGCAGTGCCAGCTCAAGTGTAAGTTACATGTACTGGTACCAGCAGAAGCCAGGATCCTCCCCCAGACTCTGGATTTATGACACATCCAACCTGGCTTCTGGAGTCCCCGCTCGCTTCAGTGGCAGTAGGTCTGGGACCTCTTATTCTCTCACAATCAGCAGCATGGAGGCTGAAGATGCTGCCACTTATTACTGCCATCAGCGGAGTAGTTACCCATTCA
IGKV4-62*01_S2199_NOD	NOD/SHILTJ	IGKV	MUSMUS IGKV4-62*01 ORF	96.74	CAAATTGTTCTCACCCAGTCTCCAGCAATCATGTCTGCATCTCCAGGGGAGAAGGTCACCATGACCTGCAGTGCCAGCTCAAGTGTAAGTTACATGTACTGGTACCAGCAGAAGCCAGGATCCTCCCCCAGACTCTGGATTTATGACACATCCAACCTGGCTTCTGGAGTCCCCGCTCGCTTCAGTGGCAGTAGGTCTGGGACCTCTTATTCTCTCACAATCAGCAGCATGGAGGCTGAAGATGCTGCCACTTATTACTGCCATCAGCGGAGTAGTTACCCATTCA

IGKV4-62*01_S2199_NOR	NOR/LTJ	IGKV	MUSMUS IGKV4-62*01 ORF	96.74	CAAATTGTTCTCACCCAGTCTCCAGCAATCATGTCTGCATCTCCAGGGGAGAAGGTCACCATGACCTGCAGTGCCAGCTCAAGTGTAAGTTACATGTACTGGTACCAGCAGAAGCCAGGATCCTCCCCCAGACTCTGGATTTATGACACATCCAACCTGGCTTCTGGAGTCCCCGCTCGCTTCAGTGGCAGTAGGTCTGGGACCTCTTATTCTCTCACAATCAGCAGCATGGAGGCTGAAGATGCTGCCACTTATTACTGCCATCAGCGGAGTAGTTACCCATTCA
IGKV4-62*01_S4904_MSM	MSM/MSJ	IGKV	MUSMUS IGKV4-62*01 ORF, OR MUSMUS IGKV4-68*01 F	96.74	CAAATTGTTCTCACCCAGTCTCCAGCAATCATGTCTGCATCTCCAGGGGAGAAGGTCACCATGACCTGCAGTGCCAGCTCAAGTGTAAGTTACATGTACTGGTACCAGCAGAAGCCAGGATCTTCCCCCAAACCCTGGATTTATGACACATCCAACCTGGCTTCTGGAGTCCCTGCTCGCTTCAGTGGCAGTGGGTCTGGGACCTCTTACTCTCTCACAATCAGCCGCATGGAGGCTGAAGATGCTGCCACTTATTACTGCCAGCAGTACAGTGGTTACCCATTCA
IGKV4-62*01_S5834_AJ	A/J	IGKV	MUSMUS IGKV4-62*01 ORF	100	CAAATTGTTCTCACCCAGTCTCCAGCAATCATGTCTGCATCTCCAGGGGAAAAGGTCACCATGACCTGCAGTGCCAGCTCAAGTGTAAGTTACATGTACTGGTACCAGCAGAAGCCAGGCTCCTCCCCCAGACTCTGGATTTATGACACATCCAACCTGGTTTCTGGAGTCCCTGCTCGCTTCAGTGGCAGTAGGTCTGGGACCTCTTATTCTCTCACAATCAGCAGCATGGAGGCTGAAGATGCTGCCACTTATTACTGCCAGCAGTACAGTGGTTACCCATACA
IGKV4-62*01_S5834_DBA1	DBA/1J	IGKV	MUSMUS IGKV4-62*01 ORF	100	CAAATTGTTCTCACCCAGTCTCCAGCAATCATGTCTGCATCTCCAGGGGAAAAGGTCACCATGACCTGCAGTGCCAGCTCAAGTGTAAGTTACATGTACTGGTACCAGCAGAAGCCAGGCTCCTCCCCCAGACTCTGGATTTATGACACATCCAACCTGGTTTCTGGAGTCCCTGCTCGCTTCAGTGGCAGTAGGTCTGGGACCTCTTATTCTCTCACAATCAGCAGCATGGAGGCTGAAGATGCTGCCACTTATTACTGCCAGCAGTACAGTGGTTACCCATACA

IGKV4-62*01_S7049_CAST	CAST/EIJ	IGKV	MUSMUS IGKV4-55*01 F	95.65	CAAATTGTTCTCACCCAGTCTCCAGCAATCATGTCTGCATCTCCAGGGGAGAAGGTCACCATGACCTGCAGTGCCAGCTCAAGTGTAAGTTACATGTACTGGTACCAGCAGAAGCCAGGATCCTCCCCCAGACTCTGGATTTATGACACATCCAACCTGGCTTCTGGAGTTTCCGCTCGCTTCCGTGGCAGTGGGTCTGGGACCTCTTATTCTCTCACAATCAGCAGCATGGAGGCTGAAGATGCTGCCACTTATTACTGCTATCAGTGGAGTAGTTACCCATTCA
IGKV4-62*01_S7295_PWD	PWD/PHJ	IGKV	MUSMUS IGKV4-62*01 ORF, OR MUSMUS IGKV4-68*01 F	96.74	CAAATTGTTCTCACCCAGTCTCCAGCAATCATGTCTGCATCTCCAGGGGAGAAGGTCACCATGACCTGCAGTGCCAGCTCAAGTGTAAGTTACATGTACTGGTACCAGCAGAAGCCAGGATCTTCCCCCAAACCCTGGATTTATGACACATCCAACCTGGCTTCTGGAGTCCCTGCTCGCTTCAGTGGCAGTGGGTCTGGGACCTCTTACTCTCTCACAATCAGCCGCATGGAGGCTGAAGATGCTGCCACTTATTACTGCCAGCAGTACAGTGGTTACCCATACA
IGKV4-62*01_S8002_MRL	MRL/MPJ	IGKV	MUSMUS IGKV4-62*01 ORF	96.74	CAAATTGTTCTCACCCAGTCTCCAGCAATCATGTCTGCATCTCCAGGGGAGAAGGTCACCATGACCTGCAGTGCCAGCTCAAGTGTAAGTTACATGTACTGGTACCAGCAGAAGCCAGGATCCTCCCCCAGACTCTGGATTTATGACACATCCAACCTGGCTTCTGGAGTCCCCGCTCGCTTCAGTGGCAGTAGGTCTGGGACCTCTTATTCTCTCACAATCAGCAGCATGGAGGCTGAAGATGCTGCCACTTATTACTGCCATCAGCGGAGTAGTTACCCATCCA
IGKV4-63*01_129	129S1/SVIMJ	IGKV	MUSMUS IGKV4-63*01 F	100	GAAAATGTTCTCACCCAGTCTCCAGCAATCATGTCTGCATCTCCAGGGGAAAAGGTCACCATGACCTGCAGTGCCAGCTCAAGTGTAAGTTACATGCACTGGTACCAGCAGAAGTCAAGCACCTCCCCCAAACTCTGGATTTATGACACATCCAAACTGGCTTCTGGAGTCCCAGGTCGCTTCAGTGGCAGTGGGTCTGGAAACTCTTACTCTCTCACGATCAGCAGCATGGAGGCTGAAGATGTTGCCACTTATTACTGTTTTCAGGGGAGTGGGTACCCACTCA

IGKV4-63*01_AJ	A/J	IGKV	MUSMUS IGKV4-63*01 F	100	GAAAATGTTCTCACCCAGTCTCCAGCAATCATGTCTGCATCTCCAGGGGAAAAGGTCACCATGACCTGCAGTGCCAGCTCAAGTGTAAGTTACATGCACTGGTACCAGCAGAAGTCAAGCACCTCCCCCAAACTCTGGATTTATGACACATCCAAACTGGCTTCTGGAGTCCCAGGTCGCTTCAGTGGCAGTGGGTCTGGAAACTCTTACTCTCTCACGATCAGCAGCATGGAGGCTGAAGATGTTGCCACTTATTACTGTTTTCAGGGGAGTGGGTACCCACTCA
IGKV4-63*01_B6	C57BL/6J	IGKV	MUSMUS IGKV4-63*01 F	100	GAAAATGTTCTCACCCAGTCTCCAGCAATCATGTCTGCATCTCCAGGGGAAAAGGTCACCATGACCTGCAGTGCCAGCTCAAGTGTAAGTTACATGCACTGGTACCAGCAGAAGTCAAGCACCTCCCCCAAACTCTGGATTTATGACACATCCAAACTGGCTTCTGGAGTCCCAGGTCGCTTCAGTGGCAGTGGGTCTGGAAACTCTTACTCTCTCACGATCAGCAGCATGGAGGCTGAAGATGTTGCCACTTATTACTGTTTTCAGGGGAGTGGGTACCCACTCA
IGKV4-63*01_BALB	BALB/CBYJ	IGKV	MUSMUS IGKV4-63*01 F	100	GAAAATGTTCTCACCCAGTCTCCAGCAATCATGTCTGCATCTCCAGGGGAAAAGGTCACCATGACCTGCAGTGCCAGCTCAAGTGTAAGTTACATGCACTGGTACCAGCAGAAGTCAAGCACCTCCCCCAAACTCTGGATTTATGACACATCCAAACTGGCTTCTGGAGTCCCAGGTCGCTTCAGTGGCAGTGGGTCTGGAAACTCTTACTCTCTCACGATCAGCAGCATGGAGGCTGAAGATGTTGCCACTTATTACTGTTTTCAGGGGAGTGGGTACCCACTCA
IGKV4-63*01_C3H	C3H/HEJ	IGKV	MUSMUS IGKV4-63*01 F	100	GAAAATGTTCTCACCCAGTCTCCAGCAATCATGTCTGCATCTCCAGGGGAAAAGGTCACCATGACCTGCAGTGCCAGCTCAAGTGTAAGTTACATGCACTGGTACCAGCAGAAGTCAAGCACCTCCCCCAAACTCTGGATTTATGACACATCCAAACTGGCTTCTGGAGTCCCAGGTCGCTTCAGTGGCAGTGGGTCTGGAAACTCTTACTCTCTCACGATCAGCAGCATGGAGGCTGAAGATGTTGCCACTTATTACTGTTTTCAGGGGAGTGGGTACCCACTCA

IGKV4-63*01_DBA1	DBA/1J	IGKV	MUSMUS IGKV4-63*01 F	100	GAAAATGTTCTCACCCAGTCTCCAGCAATCATGTCTGCATCTCCAGGGGAAAAGGTCACCATGACCTGCAGTGCCAGCTCAAGTGTAAGTTACATGCACTGGTACCAGCAGAAGTCAAGCACCTCCCCCAAACTCTGGATTTATGACACATCCAAACTGGCTTCTGGAGTCCCAGGTCGCTTCAGTGGCAGTGGGTCTGGAAACTCTTACTCTCTCACGATCAGCAGCATGGAGGCTGAAGATGTTGCCACTTATTACTGTTTTCAGGGGAGTGGGTACCCACTCA
IGKV4-63*01_DBA2	DBA/2J	IGKV	MUSMUS IGKV4-63*01 F	100	GAAAATGTTCTCACCCAGTCTCCAGCAATCATGTCTGCATCTCCAGGGGAAAAGGTCACCATGACCTGCAGTGCCAGCTCAAGTGTAAGTTACATGCACTGGTACCAGCAGAAGTCAAGCACCTCCCCCAAACTCTGGATTTATGACACATCCAAACTGGCTTCTGGAGTCCCAGGTCGCTTCAGTGGCAGTGGGTCTGGAAACTCTTACTCTCTCACGATCAGCAGCATGGAGGCTGAAGATGTTGCCACTTATTACTGTTTTCAGGGGAGTGGGTACCCACTCA
IGKV4-63*01_LEWES	LEWES/EIJ	IGKV	MUSMUS IGKV4-63*01 F	100	GAAAATGTTCTCACCCAGTCTCCAGCAATCATGTCTGCATCTCCAGGGGAAAAGGTCACCATGACCTGCAGTGCCAGCTCAAGTGTAAGTTACATGCACTGGTACCAGCAGAAGTCAAGCACCTCCCCCAAACTCTGGATTTATGACACATCCAAACTGGCTTCTGGAGTCCCAGGTCGCTTCAGTGGCAGTGGGTCTGGAAACTCTTACTCTCTCACGATCAGCAGCATGGAGGCTGAAGATGTTGCCACTTATTACTGTTTTCAGGGGAGTGGGTACCCACTCA
IGKV4-63*01_NZB	NZB/BLNJ	IGKV	MUSMUS IGKV4-63*01 F	100	GAAAATGTTCTCACCCAGTCTCCAGCAATCATGTCTGCATCTCCAGGGGAAAAGGTCACCATGACCTGCAGTGCCAGCTCAAGTGTAAGTTACATGCACTGGTACCAGCAGAAGTCAAGCACCTCCCCCAAACTCTGGATTTATGACACATCCAAACTGGCTTCTGGAGTCCCAGGTCGCTTCAGTGGCAGTGGGTCTGGAAACTCTTACTCTCTCACGATCAGCAGCATGGAGGCTGAAGATGTTGCCACTTATTACTGTTTTCAGGGGAGTGGGTACCCACTCA

IGKV4-63*01_S2117_CBA	CBA/J	IGKV	MUSMUS IGKV4-63*01 F	100	GAAAATGTTCTCACCCAGTCTCCAGCAATCATGTCTGCATCTCCAGGGGAAAAGGTCACCATGACCTGCAGTGCCAGCTCAAGTGTAAGTTACATGCACTGGTACCAGCAGAAGTCAAGCACCTCCCCCAAACTCTGGATTTATGACACATCCAAACTGGCTTCTGGAGTCCCAGGTCGCTTCAGTGGCAGTGGGTCTGGAAACTCTTACTCTCTCACGATCAGCAGCATGGAGGCTGAAGATGTTGCCACTTATTACTGTTTTCAGGGGAGTGGGTACCCATTCA
IGKV4-63*01_S6121_CAST	CAST/EIJ	IGKV	MUSMUS IGKV4-63*01 F	97.1	GAAAATGTTCTCACCCAGTCTCCAGCAATCATGTCTGCATCTCCAGGGGAGAAGGTCACCATGACCTGCAGTGCCAGCTCAAGTGTAAGTTACATGCACTGGTACCAGCAGAAGTCAGGCACCTCTCCCAAACTCTGGATTTATGACACATCCAAACTGGCTTCTGGAGTCCCAGGTCGCTTCAGTGGCAATGGGTCTGGAAACTCTTACTCTCTCACGATCAGCAGCATGGAGGCTGAAGATGCTGCCACTTATTACTGCTTTCAGGGGAGTCTGTACCCATTCA
IGKV4-63*01_SJL	SJL/J	IGKV	MUSMUS IGKV4-63*01 F	100	GAAAATGTTCTCACCCAGTCTCCAGCAATCATGTCTGCATCTCCAGGGGAAAAGGTCACCATGACCTGCAGTGCCAGCTCAAGTGTAAGTTACATGCACTGGTACCAGCAGAAGTCAAGCACCTCCCCCAAACTCTGGATTTATGACACATCCAAACTGGCTTCTGGAGTCCCAGGTCGCTTCAGTGGCAGTGGGTCTGGAAACTCTTACTCTCTCACGATCAGCAGCATGGAGGCTGAAGATGTTGCCACTTATTACTGTTTTCAGGGGAGTGGGTACCCACTCA
IGKV4-68*01_129	129S1/SVIMJ	IGKV	MUSMUS IGKV4-68*01 F	100	CAAATTGTTCTCACCCAGTCTCCAGCACTCATGTCTGCATCTCCAGGGGAGAAGGTCACCATGACCTGCAGTGCCAGCTCAAGTGTAAGTTACATGTACTGGTACCAGCAGAAGCCAAGATCCTCCCCCAAACCCTGGATTTATCTCACATCCAACCTGGCTTCTGGAGTCCCTGCTCGCTTCAGTGGCAGTGGGTCTGGGACCTCTTACTCTCTCACAATCAGCAGCATGGAGGCTGAAGATGCTGCCACTTATTACTGCCAGCAGTGGAGTAGTAACCCACCCA

IGKV4-68*01_AJ	A/J	IGKV	MUSMUS IGKV4-68*01 F	100	CAAATTGTTCTCACCCAGTCTCCAGCACTCATGTCTGCATCTCCAGGGGAGAAGGTCACCATGACCTGCAGTGCCAGCTCAAGTGTAAGTTACATGTACTGGTACCAGCAGAAGCCAAGATCCTCCCCCAAACCCTGGATTTATCTCACATCCAACCTGGCTTCTGGAGTCCCTGCTCGCTTCAGTGGCAGTGGGTCTGGGACCTCTTACTCTCTCACAATCAGCAGCATGGAGGCTGAAGATGCTGCCACTTATTACTGCCAGCAGTGGAGTAGTAACCCACCCA
IGKV4-68*01_BALB	BALB/CBYJ	IGKV	MUSMUS IGKV4-68*01 F	100	CAAATTGTTCTCACCCAGTCTCCAGCACTCATGTCTGCATCTCCAGGGGAGAAGGTCACCATGACCTGCAGTGCCAGCTCAAGTGTAAGTTACATGTACTGGTACCAGCAGAAGCCAAGATCCTCCCCCAAACCCTGGATTTATCTCACATCCAACCTGGCTTCTGGAGTCCCTGCTCGCTTCAGTGGCAGTGGGTCTGGGACCTCTTACTCTCTCACAATCAGCAGCATGGAGGCTGAAGATGCTGCCACTTATTACTGCCAGCAGTGGAGTAGTAACCCACCCA
IGKV4-68*01_C3H	C3H/HEJ	IGKV	MUSMUS IGKV4-68*01 F	100	CAAATTGTTCTCACCCAGTCTCCAGCACTCATGTCTGCATCTCCAGGGGAGAAGGTCACCATGACCTGCAGTGCCAGCTCAAGTGTAAGTTACATGTACTGGTACCAGCAGAAGCCAAGATCCTCCCCCAAACCCTGGATTTATCTCACATCCAACCTGGCTTCTGGAGTCCCTGCTCGCTTCAGTGGCAGTGGGTCTGGGACCTCTTACTCTCTCACAATCAGCAGCATGGAGGCTGAAGATGCTGCCACTTATTACTGCCAGCAGTGGAGTAGTAACCCACCCA
IGKV4-68*01_DBA1	DBA/1J	IGKV	MUSMUS IGKV4-68*01 F	100	CAAATTGTTCTCACCCAGTCTCCAGCACTCATGTCTGCATCTCCAGGGGAGAAGGTCACCATGACCTGCAGTGCCAGCTCAAGTGTAAGTTACATGTACTGGTACCAGCAGAAGCCAAGATCCTCCCCCAAACCCTGGATTTATCTCACATCCAACCTGGCTTCTGGAGTCCCTGCTCGCTTCAGTGGCAGTGGGTCTGGGACCTCTTACTCTCTCACAATCAGCAGCATGGAGGCTGAAGATGCTGCCACTTATTACTGCCAGCAGTGGAGTAGTAACCCACCCA

IGKV4-68*01_DBA2	DBA/2J	IGKV	MUSMUS IGKV4-68*01 F	100	CAAATTGTTCTCACCCAGTCTCC<br>AGCACTCATGTCTGCATCTCCA<br>GGGGAGAAGGTCACCATGACC<br>TGCAGTGCCAGCTCAAGTGTAA<br>GTTACATGTACTGGTACCAGCA<br>GAAGCCAAGATCCTCCCCCAA<br>ACCCTGGATTTATCTCACATCCA<br>ACCTGGCTTCTGGAGTCCCTGC<br>TCGCTTCAGTGGCAGTGGGTCT<br>GGGACCTCTTACTCTCTCACAAT<br>CAGCAGCATGGAGGCTGAAGAT<br>GCTGCCACTTATTACTGCCAGC<br>AGTGGAGTAGTAACCCACCCA
IGKV4-68*01_NZB	NZB/BLNJ	IGKV	MUSMUS IGKV4-68*01 F	100	CAAATTGTTCTCACCCAGTCTCC<br>AGCACTCATGTCTGCATCTCCA<br>GGGGAGAAGGTCACCATGACC<br>TGCAGTGCCAGCTCAAGTGTAA<br>GTTACATGTACTGGTACCAGCA<br>GAAGCCAAGATCCTCCCCCAA<br>ACCCTGGATTTATCTCACATCCA<br>ACCTGGCTTCTGGAGTCCCTGC<br>TCGCTTCAGTGGCAGTGGGTCT<br>GGGACCTCTTACTCTCTCACAAT<br>CAGCAGCATGGAGGCTGAAGAT<br>GCTGCCACTTATTACTGCCAGC<br>AGTGGAGTAGTAACCCACCCA
IGKV4-68*01_S2180_CAST	CAST/EIJ	IGKV	MUSMUS IGKV4-68*01 F	97.83	CAAATTGTTCTCACCCAGTCTCC<br>AGCAATCATGTCTGCATCTCCA<br>GGGGAGAAGGTCACCATGACC<br>TGCAGTGCCAGCTCAAGTGTAA<br>GTTACATGTACTGGTACCAGCA<br>GAAGCCAGGATCCTCACCCAA<br>ACCCTGGATTTATCGCACATCC<br>AACCTGGCTTCTGGAGTCCCAA<br>CTCGCTTCAGTGGCAGTGGGTC<br>TGGGACCTCTTACTCTCTCACAA<br>TCAGCAGCATGGAGGCTGAAGA<br>TGCTGCCACTTATTACTGCCAG<br>CAGTGGAGTAGTAACCCACTCA
IGKV4-68*01_S3092_AJ	A/J	IGKV	MUSMUS IGKV4-68*01 F	100	CAAATTGTTCTCACCCAGTCTCC<br>AGCACTCATGTCTGCATCTCCA<br>GGGGAGAAGGTCACCATGACC<br>TGCAGTGCCAGCTCAAGTGTAA<br>GTTACATGTACTGGTACCAGCA<br>GAAGCCAAGATCCTCCCCCAA<br>ACCCTGGATTTATCTCACATCCA<br>ACCTGGCTTCTGGAGTCCCTGC<br>TCGCTTCAGTGGCAGTGGGTCT<br>GGGACCTCTTACTCTCTCACAAT<br>CAGCAGCATGGAGGCTGAAGAT<br>GCTGCCACTTATTACTGCCAGC<br>AGTGGAGTAGTAACCCACTCA

IGKV4-68*01_S3092_B6	C57BL/6J	IGKV	MUSMUS IGKV4-68*01 F	100	CAAATTGTTCTCACCCAGTCTCCAGCACTCATGTCTGCATCTCCAGGGGAGAAGGTCACCATGACCTGCAGTGCCAGCTCAAGTGTAAGTTACATGTACTGGTACCAGCAGAAGCCAAGATCCTCCCCCAAACCCTGGATTTATCTCACATCCAACCTGGCTTCTGGAGTCCCTGCTCGCTTCAGTGGCAGTGGGTCTGGGACCTCTTACTCTCTCACAATCAGCAGCATGGAGGCTGAAGATGCTGCCACTTATTACTGCCAGCAGTGGAGTAGTAACCCACTCA
IGKV4-68*01_S3092_C3H	C3H/HEJ	IGKV	MUSMUS IGKV4-68*01 F	100	CAAATTGTTCTCACCCAGTCTCCAGCACTCATGTCTGCATCTCCAGGGGAGAAGGTCACCATGACCTGCAGTGCCAGCTCAAGTGTAAGTTACATGTACTGGTACCAGCAGAAGCCAAGATCCTCCCCCAAACCCTGGATTTATCTCACATCCAACCTGGCTTCTGGAGTCCCTGCTCGCTTCAGTGGCAGTGGGTCTGGGACCTCTTACTCTCTCACAATCAGCAGCATGGAGGCTGAAGATGCTGCCACTTATTACTGCCAGCAGTGGAGTAGTAACCCACTCA
IGKV4-68*01_S3092_CBA	CBA/J	IGKV	MUSMUS IGKV4-68*01 F	100	CAAATTGTTCTCACCCAGTCTCCAGCACTCATGTCTGCATCTCCAGGGGAGAAGGTCACCATGACCTGCAGTGCCAGCTCAAGTGTAAGTTACATGTACTGGTACCAGCAGAAGCCAAGATCCTCCCCCAAACCCTGGATTTATCTCACATCCAACCTGGCTTCTGGAGTCCCTGCTCGCTTCAGTGGCAGTGGGTCTGGGACCTCTTACTCTCTCACAATCAGCAGCATGGAGGCTGAAGATGCTGCCACTTATTACTGCCAGCAGTGGAGTAGTAACCCACTCA
IGKV4-68*01_S3092_DBA1	DBA/1J	IGKV	MUSMUS IGKV4-68*01 F	100	CAAATTGTTCTCACCCAGTCTCCAGCACTCATGTCTGCATCTCCAGGGGAGAAGGTCACCATGACCTGCAGTGCCAGCTCAAGTGTAAGTTACATGTACTGGTACCAGCAGAAGCCAAGATCCTCCCCCAAACCCTGGATTTATCTCACATCCAACCTGGCTTCTGGAGTCCCTGCTCGCTTCAGTGGCAGTGGGTCTGGGACCTCTTACTCTCTCACAATCAGCAGCATGGAGGCTGAAGATGCTGCCACTTATTACTGCCAGCAGTGGAGTAGTAACCCACTCA

IGKV4-68*01_S3092_LEWES	LEWES/EIJ	IGKV	MUSMUS IGKV4-68*01 F	100	CAAATTGTTCTCACCCAGTCTCCAGCACTCATGTCTGCATCTCCAGGGGAGAAGGTCACCATGACCTGCAGTGCCAGCTCAAGTGTAAGTTACATGTACTGGTACCAGCAGAAGCCAAGATCCTCCCCCAAACCCTGGATTTATCTCACATCCAACCTGGCTTCTGGAGTCCCTGCTCGCTTCAGTGGCAGTGGGTCTGGGACCTCTTACTCTCTCACAATCAGCAGCATGGAGGCTGAAGATGCTGCCACTTATTACTGCCAGCAGTGGAGTAGTAACCCACTCA
IGKV4-68*01_S3092_SJL	SJL/J	IGKV	MUSMUS IGKV4-68*01 F	100	CAAATTGTTCTCACCCAGTCTCCAGCACTCATGTCTGCATCTCCAGGGGAGAAGGTCACCATGACCTGCAGTGCCAGCTCAAGTGTAAGTTACATGTACTGGTACCAGCAGAAGCCAAGATCCTCCCCCAAACCCTGGATTTATCTCACATCCAACCTGGCTTCTGGAGTCCCTGCTCGCTTCAGTGGCAGTGGGTCTGGGACCTCTTACTCTCTCACAATCAGCAGCATGGAGGCTGAAGATGCTGCCACTTATTACTGCCAGCAGTGGAGTAGTAACCCACTCA
IGKV4-68*01_S6732_CAST	CAST/EIJ	IGKV	MUSMUS IGKV4-68*01 F	97.83	CAAATTGTTCTCACCCAGTCTCCAGCAATCATGTCTGCATCTCCAGGGGAGAAGGTCACCATGACCTGCAGTGCCAGCTCAAGTGTAAGTTACATGTACTGGTACCAGCAGAAGCCAGGATCCTCACCCAAACCCTGGATTTATCGCACATCCAACCTGGCTTCTGGAGTCCCAACTCGCTTCAGTGGCAGTGGGTCTGGGACCTCTTACTCTCTCACAATCAGCAGCATGGAGGCTGAAGATGCTGCCACTTATTACTGCCAGCAGTGGAGTAGTAACCCACCCA
IGKV4-68*01_S8363_MSM	MSM/MSJ	IGKV	MUSMUS IGKV4-68*01 F	96.74	CAAATTGTTCTCACCCAGTCTCCAGCAATCATGTCTGCATCTCCAGGGGAGAAGGTCACCATGACCTGCAGTGCCAGCTCAAGTGTAAGTTATATGCACTGGTACCAGCAGAAGCCAGGATCCTCACCCAAACCCTGGATTTATCGCACATCCAACCTGGCTTCTGGAGTCCCTGCTCGCTTCAGTGGCAGTGGGTCTGGGACCTCTTACTCTCTCACAATTACCAGCATTGAGGCTGAAGATGCTGCCACTTATTACTGCCAGCAGTGGAGTAGTAACCCACCCA

IGKV4-68*01_S8814_PWD	PWD/PHJ	IGKV	MUSMUS IGKV4-68*01 F	96.74	CAAATTGTTCTCACCCAGTCTCCAGCAATCATGTCTGCATCTCCAGGGGAGAAGGTCACCATGACCTGCAGTGCCAGCTCAAGTGTAAGTTATATGCACTGGTACCAGCAGAAGCCAGGATCCTCACCCAAACCCTGGATTTATCGCACATCCAACCTGGCTTCTGGAGTCCCTGCTCGCTTCAGTGGCAGTGGGTCTGGGACCTCTTACTCTCTCACAATTACCAGCATTGAGGCTGAAGATGCTGCCACTTATTACTGCCAGCAGTGGAGTAGTAACCCACTCA
IGKV4-69*01_129	129S1/SVIMJ	IGKV	MUSMUS IGKV4-69*01 F	100	CAAATTCTTCTCACCCAGTCTCCAGCAATCATGTCTGCATCTCCAGGGGAGAAGGTCACCATGACCTGCAGTGCCAGCTCAAGTGTAAGTTACATGCACTGGTACCAGCAGAAGCCAGGATCCTCGCCCAAACCCTGGATTTATGACACATCCAACCTGGCTTCTGGATTCCCTGCTCGCTTCAGTGGCAGTGGGTCTGGGACCTCTTACTCTCTCATAATCAGCAGCATGGAGGCTGAAGATGCTGCCACTTATTACTGCCATCAGCGGAGTAGTTACCCA
IGKV4-69*01_AJ	A/J	IGKV	MUSMUS IGKV4-69*01 F	100	CAAATTCTTCTCACCCAGTCTCCAGCAATCATGTCTGCATCTCCAGGGGAGAAGGTCACCATGACCTGCAGTGCCAGCTCAAGTGTAAGTTACATGCACTGGTACCAGCAGAAGCCAGGATCCTCGCCCAAACCCTGGATTTATGACACATCCAACCTGGCTTCTGGATTCCCTGCTCGCTTCAGTGGCAGTGGGTCTGGGACCTCTTACTCTCTCATAATCAGCAGCATGGAGGCTGAAGATGCTGCCACTTATTACTGCCATCAGCGGAGTAGTTACCCA
IGKV4-69*01_B6	C57BL/6J	IGKV	MUSMUS IGKV4-69*01 F	100	CAAATTCTTCTCACCCAGTCTCCAGCAATCATGTCTGCATCTCCAGGGGAGAAGGTCACCATGACCTGCAGTGCCAGCTCAAGTGTAAGTTACATGCACTGGTACCAGCAGAAGCCAGGATCCTCGCCCAAACCCTGGATTTATGACACATCCAACCTGGCTTCTGGATTCCCTGCTCGCTTCAGTGGCAGTGGGTCTGGGACCTCTTACTCTCTCATAATCAGCAGCATGGAGGCTGAAGATGCTGCCACTTATTACTGCCATCAGCGGAGTAGTTACCCA

IGKV4-69*01_BALB	BALB/CBYJ	IGKV	MUSMUS IGKV4-69*01 F	100	CAAATTCTTCTCACCCAGTCTCCAGCAATCATGTCTGCATCTCCAGGGGAGAAGGTCACCATGACCTGCAGTGCCAGCTCAAGTGTAAGTTACATGCACTGGTACCAGCAGAAGCCAGGATCCTCGCCCAAACCCTGGATTTATGACACATCCAACCTGGCTTCTGGATTCCCTGCTCGCTTCAGTGGCAGTGGGTCTGGGACCTCTTACTCTCTCATAATCAGCAGCATGGAGGCTGAAGATGCTGCCACTTATTACTGCCATCAGCGGAGTAGTTACCCA
IGKV4-69*01_C3H	C3H/HEJ	IGKV	MUSMUS IGKV4-69*01 F	100	CAAATTCTTCTCACCCAGTCTCCAGCAATCATGTCTGCATCTCCAGGGGAGAAGGTCACCATGACCTGCAGTGCCAGCTCAAGTGTAAGTTACATGCACTGGTACCAGCAGAAGCCAGGATCCTCGCCCAAACCCTGGATTTATGACACATCCAACCTGGCTTCTGGATTCCCTGCTCGCTTCAGTGGCAGTGGGTCTGGGACCTCTTACTCTCTCATAATCAGCAGCATGGAGGCTGAAGATGCTGCCACTTATTACTGCCATCAGCGGAGTAGTTACCCA
IGKV4-69*01_CBA	CBA/J	IGKV	MUSMUS IGKV4-69*01 F	100	CAAATTCTTCTCACCCAGTCTCCAGCAATCATGTCTGCATCTCCAGGGGAGAAGGTCACCATGACCTGCAGTGCCAGCTCAAGTGTAAGTTACATGCACTGGTACCAGCAGAAGCCAGGATCCTCGCCCAAACCCTGGATTTATGACACATCCAACCTGGCTTCTGGATTCCCTGCTCGCTTCAGTGGCAGTGGGTCTGGGACCTCTTACTCTCTCATAATCAGCAGCATGGAGGCTGAAGATGCTGCCACTTATTACTGCCATCAGCGGAGTAGTTACCCA
IGKV4-69*01_DBA1	DBA/1J	IGKV	MUSMUS IGKV4-69*01 F	100	CAAATTCTTCTCACCCAGTCTCCAGCAATCATGTCTGCATCTCCAGGGGAGAAGGTCACCATGACCTGCAGTGCCAGCTCAAGTGTAAGTTACATGCACTGGTACCAGCAGAAGCCAGGATCCTCGCCCAAACCCTGGATTTATGACACATCCAACCTGGCTTCTGGATTCCCTGCTCGCTTCAGTGGCAGTGGGTCTGGGACCTCTTACTCTCTCATAATCAGCAGCATGGAGGCTGAAGATGCTGCCACTTATTACTGCCATCAGCGGAGTAGTTACCCA

IGKV4-69*01_DBA2	DBA/2J	IGKV	MUSMUS IGKV4-69*01 F	100	CAAATTCTTCTCACCCAGTCTCCAGCAATCATGTCTGCATCTCCAGGGGAGAAGGTCACCATGACCTGCAGTGCCAGCTCAAGTGTAAGTTACATGCACTGGTACCAGCAGAAGCCAGGATCCTCGCCCAAACCCTGGATTTATGACACATCCAACCTGGCTTCTGGATTCCCTGCTCGCTTCAGTGGCAGTGGGTCTGGGACCTCTTACTCTCTCATAATCAGCAGCATGGAGGCTGAAGATGCTGCCACTTATTACTGCCATCAGCGGAGTAGTTACCCA
IGKV4-69*01_NZB	NZB/BLNJ	IGKV	MUSMUS IGKV4-69*01 F	100	CAAATTCTTCTCACCCAGTCTCCAGCAATCATGTCTGCATCTCCAGGGGAGAAGGTCACCATGACCTGCAGTGCCAGCTCAAGTGTAAGTTACATGCACTGGTACCAGCAGAAGCCAGGATCCTCGCCCAAACCCTGGATTTATGACACATCCAACCTGGCTTCTGGATTCCCTGCTCGCTTCAGTGGCAGTGGGTCTGGGACCTCTTACTCTCTCATAATCAGCAGCATGGAGGCTGAAGATGCTGCCACTTATTACTGCCATCAGCGGAGTAGTTACCCA
IGKV4-69*01_S2827_CAST	CAST/EIJ	IGKV	MUSMUS IGKV4-69*01 F	97.1	CAAATTCTTCTCACCCAGTCTCCAGCAATCATGTCTGCATCTCCAGGAGAGAAGGTCACCATGACCTGCAGTGCCAGCTCAAGTGTAAGTTATATGCACTGGTACCAGCAGAAGCCAGGATCCTCGCCCAAACCCTGGATTTATGGCACATCCAACCTGGCTTCTGGAGTCCCTGTTCGCTTCAGTGGCAGTGGGTCTGGGACCTCTTACTCTCTCACAATCAGCAGCATGGAGGCTGAAGATGCTGCCACTTATTACTGCCAGCAGTGGAGTAGTTACCCA
IGKV4-69*01_S6643_LEWES	LEWES/EIJ	IGKV	MUSMUS IGKV4-69*01 F	99.64	CAAATTCTTCTCACCCAGTCTCCAGCAATCATGTCTGCATCTCCAGGGGAGAAGGTCACCATGACCTGCAGTGCCAGCTCAAGTGTAAGTTACATGCACTGGTACCAGCAGAAGCCAGGATCCTCGCCCAAACCCTGGATTTATGACACATCCAACCTGGCTTCTGGATTCCCTGCTCGCTTCAGTGGCTGTGGGTCTGGGACCTCTTACTCTCTCATAATCAGCAGCATGGAGGCTGAAGATGCTGCCACTTATTACTGCCATCAGCGGAGTAGTTACCC

IGKV4-69*01_SJL	SJL/J	IGKV	MUSMUS IGKV4-69*01 F	100	CAAATTCTTCTCACCCAGTCTCC<br>AGCAATCATGTCTGCATCTCCA<br>GGGGAGAAGGTCACCATGACC<br>TGCAGTGCCAGCTCAAGTGTAA<br>GTTACATGCACTGGTACCAGCA<br>GAAGCCAGGATCCTCGCCCAA<br>ACCCTGGATTTATGACACATCCA<br>ACCTGGCTTCTGGATTCCCTGC<br>TCGCTTCAGTGGCAGTGGGTCT<br>GGGACCTCTTACTCTCTCATAAT<br>CAGCAGCATGGAGGCTGAAGAT<br>GCTGCCACTTATTACTGCCATCA<br>GCGGAGTAGTTACCCA
IGKV4-70*01_129	129S1/SVIMJ	IGKV	MUSMUS IGKV4-70*01 F	100	CAAATTGTTCTCACCCAGTCTCC<br>AGCAATCATGTCTGCATCTCCA<br>GGGGAGAAGGTCACCATGACC<br>TGCAGTGCCAGCTCAAGTATAA<br>GTTACATGCACTGGTACCAGCA<br>GAAGCCAGGCACCTCCCCCAA<br>AAGATGGATTTATGACACATCCA<br>AACTGGCTTCTGGAGTCCCTGC<br>TCGCTTCAGTGGCAGTGGGTCT<br>GGGACCTCTTATTCTCTCACAAT<br>CAGCAGCATGGAGGCTGAAGAT<br>GCTGCCACTTATTACTGCCATCA<br>GCGGAGTAGTTACCCA
IGKV4-70*01_AJ	A/J	IGKV	MUSMUS IGKV4-70*01 F	100	CAAATTGTTCTCACCCAGTCTCC<br>AGCAATCATGTCTGCATCTCCA<br>GGGGAGAAGGTCACCATGACC<br>TGCAGTGCCAGCTCAAGTATAA<br>GTTACATGCACTGGTACCAGCA<br>GAAGCCAGGCACCTCCCCCAA<br>AAGATGGATTTATGACACATCCA<br>AACTGGCTTCTGGAGTCCCTGC<br>TCGCTTCAGTGGCAGTGGGTCT<br>GGGACCTCTTATTCTCTCACAAT<br>CAGCAGCATGGAGGCTGAAGAT<br>GCTGCCACTTATTACTGCCATCA<br>GCGGAGTAGTTACCCA
IGKV4-70*01_B6	C57BL/6J	IGKV	MUSMUS IGKV4-70*01 F	100	CAAATTGTTCTCACCCAGTCTCC<br>AGCAATCATGTCTGCATCTCCA<br>GGGGAGAAGGTCACCATGACC<br>TGCAGTGCCAGCTCAAGTATAA<br>GTTACATGCACTGGTACCAGCA<br>GAAGCCAGGCACCTCCCCCAA<br>AAGATGGATTTATGACACATCCA<br>AACTGGCTTCTGGAGTCCCTGC<br>TCGCTTCAGTGGCAGTGGGTCT<br>GGGACCTCTTATTCTCTCACAAT<br>CAGCAGCATGGAGGCTGAAGAT<br>GCTGCCACTTATTACTGCCATCA<br>GCGGAGTAGTTACCCA

IGKV4-70*01_BALB	BALB/CBYJ	IGKV	MUSMUS IGKV4-70*01 F	100	CAAATTGTTCTCACCCAGTCTCCAGCAATCATGTCTGCATCTCCAGGGGAGAAGGTCACCATGACCTGCAGTGCCAGCTCAAGTATAAGTTACATGCACTGGTACCAGCAGAAGCCAGGCACCTCCCCCAAAAGATGGATTTATGACACATCCAAACTGGCTTCTGGAGTCCCTGCTCGCTTCAGTGGCAGTGGGTCTGGGACCTCTTATTCTCTCACAATCAGCAGCATGGAGGCTGAAGATGCTGCCACTTATTACTGCCATCAGCGGAGTAGTTACCCA
IGKV4-70*01_C3H	C3H/HEJ	IGKV	MUSMUS IGKV4-70*01 F	100	CAAATTGTTCTCACCCAGTCTCCAGCAATCATGTCTGCATCTCCAGGGGAGAAGGTCACCATGACCTGCAGTGCCAGCTCAAGTATAAGTTACATGCACTGGTACCAGCAGAAGCCAGGCACCTCCCCCAAAAGATGGATTTATGACACATCCAAACTGGCTTCTGGAGTCCCTGCTCGCTTCAGTGGCAGTGGGTCTGGGACCTCTTATTCTCTCACAATCAGCAGCATGGAGGCTGAAGATGCTGCCACTTATTACTGCCATCAGCGGAGTAGTTACCCA
IGKV4-70*01_CBA	CBA/J	IGKV	MUSMUS IGKV4-70*01 F	100	CAAATTGTTCTCACCCAGTCTCCAGCAATCATGTCTGCATCTCCAGGGGAGAAGGTCACCATGACCTGCAGTGCCAGCTCAAGTATAAGTTACATGCACTGGTACCAGCAGAAGCCAGGCACCTCCCCCAAAAGATGGATTTATGACACATCCAAACTGGCTTCTGGAGTCCCTGCTCGCTTCAGTGGCAGTGGGTCTGGGACCTCTTATTCTCTCACAATCAGCAGCATGGAGGCTGAAGATGCTGCCACTTATTACTGCCATCAGCGGAGTAGTTACCCA
IGKV4-70*01_DBA1	DBA/1J	IGKV	MUSMUS IGKV4-70*01 F	100	CAAATTGTTCTCACCCAGTCTCCAGCAATCATGTCTGCATCTCCAGGGGAGAAGGTCACCATGACCTGCAGTGCCAGCTCAAGTATAAGTTACATGCACTGGTACCAGCAGAAGCCAGGCACCTCCCCCAAAAGATGGATTTATGACACATCCAAACTGGCTTCTGGAGTCCCTGCTCGCTTCAGTGGCAGTGGGTCTGGGACCTCTTATTCTCTCACAATCAGCAGCATGGAGGCTGAAGATGCTGCCACTTATTACTGCCATCAGCGGAGTAGTTACCCA

IGKV4-70*01_DBA2	DBA/2J	IGKV	MUSMUS IGKV4-70*01 F	100	CAAATTGTTCTCACCCAGTCTCCAGCAATCATGTCTGCATCTCCAGGGGAGAAGGTCACCATGACCTGCAGTGCCAGCTCAAGTATAAGTTACATGCACTGGTACCAGCAGAAGCCAGGCACCTCCCCCAAAAGATGGATTTATGACACATCCAAACTGGCTTCTGGAGTCCCTGCTCGCTTCAGTGGCAGTGGGTCTGGGACCTCTTATTCTCTCACAATCAGCAGCATGGAGGCTGAAGATGCTGCCACTTATTACTGCCATCAGCGGAGTAGTTACCCA
IGKV4-70*01_LEWES	LEWES/EIJ	IGKV	MUSMUS IGKV4-70*01 F	100	CAAATTGTTCTCACCCAGTCTCCAGCAATCATGTCTGCATCTCCAGGGGAGAAGGTCACCATGACCTGCAGTGCCAGCTCAAGTATAAGTTACATGCACTGGTACCAGCAGAAGCCAGGCACCTCCCCCAAAAGATGGATTTATGACACATCCAAACTGGCTTCTGGAGTCCCTGCTCGCTTCAGTGGCAGTGGGTCTGGGACCTCTTATTCTCTCACAATCAGCAGCATGGAGGCTGAAGATGCTGCCACTTATTACTGCCATCAGCGGAGTAGTTACCCA
IGKV4-70*01_NZB	NZB/BLNJ	IGKV	MUSMUS IGKV4-70*01 F	100	CAAATTGTTCTCACCCAGTCTCCAGCAATCATGTCTGCATCTCCAGGGGAGAAGGTCACCATGACCTGCAGTGCCAGCTCAAGTATAAGTTACATGCACTGGTACCAGCAGAAGCCAGGCACCTCCCCCAAAAGATGGATTTATGACACATCCAAACTGGCTTCTGGAGTCCCTGCTCGCTTCAGTGGCAGTGGGTCTGGGACCTCTTATTCTCTCACAATCAGCAGCATGGAGGCTGAAGATGCTGCCACTTATTACTGCCATCAGCGGAGTAGTTACCCA
IGKV4-70*01_SJL	SJL/J	IGKV	MUSMUS IGKV4-70*01 F	100	CAAATTGTTCTCACCCAGTCTCCAGCAATCATGTCTGCATCTCCAGGGGAGAAGGTCACCATGACCTGCAGTGCCAGCTCAAGTATAAGTTACATGCACTGGTACCAGCAGAAGCCAGGCACCTCCCCCAAAAGATGGATTTATGACACATCCAAACTGGCTTCTGGAGTCCCTGCTCGCTTCAGTGGCAGTGGGTCTGGGACCTCTTATTCTCTCACAATCAGCAGCATGGAGGCTGAAGATGCTGCCACTTATTACTGCCATCAGCGGAGTAGTTACCCA

IGKV4-71*01_129	129S1/SVIMJ	IGKV	MUSMUS IGKV4-71*01 F	100	CAAATTGTTCTCACCCAGTCTCCAGCAATCATGTCTGCATCTCCAGGGGAGAAGGTCACCATGACCTGCAGTGCCAGCTCAAGTGTAAGTTACATGCACTGGTACCAGCAGAAGCCAGGATCCTCCCCCAGACTCTGGATTTATTTAACATTCAACTTGGCTTCTGGAGTCCCTGCTCGCTTCAGTGGCAGTGGGTCTGGGACCTCTTACTCTCTCTCAATCAGCAGCATGGAGGCTGAAGATGCTGCCACTTATTACTGCCAGCAGTGGAGTAGTAACCCACCCA
IGKV4-71*01_B6	C57BL/6J	IGKV	MUSMUS IGKV4-71*01 F	100	CAAATTGTTCTCACCCAGTCTCCAGCAATCATGTCTGCATCTCCAGGGGAGAAGGTCACCATGACCTGCAGTGCCAGCTCAAGTGTAAGTTACATGCACTGGTACCAGCAGAAGCCAGGATCCTCCCCCAGACTCTGGATTTATTTAACATTCAACTTGGCTTCTGGAGTCCCTGCTCGCTTCAGTGGCAGTGGGTCTGGGACCTCTTACTCTCTCTCAATCAGCAGCATGGAGGCTGAAGATGCTGCCACTTATTACTGCCAGCAGTGGAGTAGTAACCCACCCA
IGKV4-71*01_NZB	NZB/BLNJ	IGKV	MUSMUS IGKV4-71*01 F	100	CAAATTGTTCTCACCCAGTCTCCAGCAATCATGTCTGCATCTCCAGGGGAGAAGGTCACCATGACCTGCAGTGCCAGCTCAAGTGTAAGTTACATGCACTGGTACCAGCAGAAGCCAGGATCCTCCCCCAGACTCTGGATTTATTTAACATTCAACTTGGCTTCTGGAGTCCCTGCTCGCTTCAGTGGCAGTGGGTCTGGGACCTCTTACTCTCTCTCAATCAGCAGCATGGAGGCTGAAGATGCTGCCACTTATTACTGCCAGCAGTGGAGTAGTAACCCACCCA
IGKV4-72*01_129	129S1/SVIMJ	IGKV	MUSMUS IGKV4-72*01 F	100	CAAATTGTTCTCTCCCAGTCTCCAGCAATCCTGTCTGCATCTCCAGGGGAGAAGGTCACAATGACTTGCAGGGCCAGCTCAAGTGTAAGTTACATGCACTGGTACCAGCAGAAGCCAGGATCCTCCCCCAAACCCTGGATTTATGCCACATCCAACCTGGCTTCTGGAGTCCCTGCTCGCTTCAGTGGCAGTGGGTCTGGGACCTCTTACTCTCTCACAATCAGCAGAGTGGAGGCTGAAGATGCTGCCACTTATTACTGCCAGCAGTGGAGTAGTAACCCACCCA

IGKV4-72*01_AKR	AKR/J	IGKV	MUSMUS IGKV4-72*01 F	100	CAAATTGTTCTCTCCCAGTCTCCAGCAATCCTGTCTGCATCTCCAGGGGAGAAGGTCACAATGACTTGCAGGGCCAGCTCAAGTGTAAGTTACATGCACTGGTACCAGCAGAAGCCAGGATCCTCCCCCAAACCCTGGATTTATGCCACATCCAACCTGGCTTCTGGAGTCCCTGCTCGCTTCAGTGGCAGTGGGTCTGGGACCTCTTACTCTCTCACAATCAGCAGAGTGGAGGCTGAAGATGCTGCCACTTATTACTGCCAGCAGTGGAGTAGTAACCCACCCA
IGKV4-72*01_B6	C57BL/6J	IGKV	MUSMUS IGKV4-72*01 F	100	CAAATTGTTCTCTCCCAGTCTCCAGCAATCCTGTCTGCATCTCCAGGGGAGAAGGTCACAATGACTTGCAGGGCCAGCTCAAGTGTAAGTTACATGCACTGGTACCAGCAGAAGCCAGGATCCTCCCCCAAACCCTGGATTTATGCCACATCCAACCTGGCTTCTGGAGTCCCTGCTCGCTTCAGTGGCAGTGGGTCTGGGACCTCTTACTCTCTCACAATCAGCAGAGTGGAGGCTGAAGATGCTGCCACTTATTACTGCCAGCAGTGGAGTAGTAACCCACCCA
IGKV4-72*01_BALB	BALB/CBYJ	IGKV	MUSMUS IGKV4-72*01 F	100	CAAATTGTTCTCTCCCAGTCTCCAGCAATCCTGTCTGCATCTCCAGGGGAGAAGGTCACAATGACTTGCAGGGCCAGCTCAAGTGTAAGTTACATGCACTGGTACCAGCAGAAGCCAGGATCCTCCCCCAAACCCTGGATTTATGCCACATCCAACCTGGCTTCTGGAGTCCCTGCTCGCTTCAGTGGCAGTGGGTCTGGGACCTCTTACTCTCTCACAATCAGCAGAGTGGAGGCTGAAGATGCTGCCACTTATTACTGCCAGCAGTGGAGTAGTAACCCACCCA
IGKV4-72*01_C3H	C3H/HEJ	IGKV	MUSMUS IGKV4-72*01 F	100	CAAATTGTTCTCTCCCAGTCTCCAGCAATCCTGTCTGCATCTCCAGGGGAGAAGGTCACAATGACTTGCAGGGCCAGCTCAAGTGTAAGTTACATGCACTGGTACCAGCAGAAGCCAGGATCCTCCCCCAAACCCTGGATTTATGCCACATCCAACCTGGCTTCTGGAGTCCCTGCTCGCTTCAGTGGCAGTGGGTCTGGGACCTCTTACTCTCTCACAATCAGCAGAGTGGAGGCTGAAGATGCTGCCACTTATTACTGCCAGCAGTGGAGTAGTAACCCACCCA

IGKV4-72*01_CBA	CBA/J	IGKV	MUSMUS IGKV4-72*01 F	100	CAAATTGTTCTCTCCCAGTCTCC<br>AGCAATCCTGTCTGCATCTCCA<br>GGGGAGAAGGTCACAATGACTT<br>GCAGGGCCAGCTCAAGTGTAA<br>GTTACATGCACTGGTACCAGCA<br>GAAGCCAGGATCCTCCCCCAA<br>ACCCTGGATTTATGCCACATCC<br>AACCTGGCTTCTGGAGTCCCTG<br>CTCGCTTCAGTGGCAGTGGGTC<br>TGGGACCTCTTACTCTCTCACAA<br>TCAGCAGAGTGGAGGCTGAAG<br>ATGCTGCCACTTATTACTGCCAG<br>CAGTGGAGTAGTAACCCACCCA
IGKV4-72*01_DBA1	DBA/1J	IGKV	MUSMUS IGKV4-72*01 F	100	CAAATTGTTCTCTCCCAGTCTCC<br>AGCAATCCTGTCTGCATCTCCA<br>GGGGAGAAGGTCACAATGACTT<br>GCAGGGCCAGCTCAAGTGTAA<br>GTTACATGCACTGGTACCAGCA<br>GAAGCCAGGATCCTCCCCCAA<br>ACCCTGGATTTATGCCACATCC<br>AACCTGGCTTCTGGAGTCCCTG<br>CTCGCTTCAGTGGCAGTGGGTC<br>TGGGACCTCTTACTCTCTCACAA<br>TCAGCAGAGTGGAGGCTGAAG<br>ATGCTGCCACTTATTACTGCCAG<br>CAGTGGAGTAGTAACCCACCCA
IGKV4-72*01_DBA2	DBA/2J	IGKV	MUSMUS IGKV4-72*01 F	100	CAAATTGTTCTCTCCCAGTCTCC<br>AGCAATCCTGTCTGCATCTCCA<br>GGGGAGAAGGTCACAATGACTT<br>GCAGGGCCAGCTCAAGTGTAA<br>GTTACATGCACTGGTACCAGCA<br>GAAGCCAGGATCCTCCCCCAA<br>ACCCTGGATTTATGCCACATCC<br>AACCTGGCTTCTGGAGTCCCTG<br>CTCGCTTCAGTGGCAGTGGGTC<br>TGGGACCTCTTACTCTCTCACAA<br>TCAGCAGAGTGGAGGCTGAAG<br>ATGCTGCCACTTATTACTGCCAG<br>CAGTGGAGTAGTAACCCACCCA
IGKV4-72*01_NOR	NOR/LTJ	IGKV	MUSMUS IGKV4-72*01 F	100	CAAATTGTTCTCTCCCAGTCTCC<br>AGCAATCCTGTCTGCATCTCCA<br>GGGGAGAAGGTCACAATGACTT<br>GCAGGGCCAGCTCAAGTGTAA<br>GTTACATGCACTGGTACCAGCA<br>GAAGCCAGGATCCTCCCCCAA<br>ACCCTGGATTTATGCCACATCC<br>AACCTGGCTTCTGGAGTCCCTG<br>CTCGCTTCAGTGGCAGTGGGTC<br>TGGGACCTCTTACTCTCTCACAA<br>TCAGCAGAGTGGAGGCTGAAG<br>ATGCTGCCACTTATTACTGCCAG<br>CAGTGGAGTAGTAACCCACCCA

IGKV4-72*01_NZB	NZB/BLNJ	IGKV	MUSMUS IGKV4-72*01 F	100	CAAATTGTTCTCTCCCAGTCTCC AGCAATCCTGTCTGCATCTCCA GGGGAGAAGGTCACAATGACTT GCAGGGCCAGCTCAAGTGTAA GTTACATGCACTGGTACCAGCA GAAGCCAGGATCCTCCCCCAA ACCCTGGATTTATGCCACATCC AACCTGGCTTCTGGAGTCCCTG CTCGCTTCAGTGGCAGTGGGTC TGGGACCTCTTACTCTCTCACAA TCAGCAGAGTGGAGGCTGAAG ATGCTGCCACTTATTACTGCCAG CAGTGGAGTAGTAACCCACCCA
IGKV4-72*01_S3540_AJ	A/J	IGKV	MUSMUS IGKV4-72*01 F	100	CAAATTGTTCTCTCCCAGTCTCC AGCAATCCTGTCTGCATCTCCA GGGGAGAAGGTCACAATGACTT GCAGGGCCAGCTCAAGTGTAA GTTACATGCACTGGTACCAGCA GAAGCCAGGATCCTCCCCCAA ACCCTGGATTTATGCCACATCC AACCTGGCTTCTGGAGTCCCTG CTCGCTTCAGTGGCAGTGGGTC TGGGACCTCTTACTCTCTCACAA TCAGCAGAGTGGAGGCTGAAG ATGCTGCCACTTATTACTGCCAG CAGTGGAGTAGTAACCCACTCA
IGKV4-72*01_S3540_C3H	C3H/HEJ	IGKV	MUSMUS IGKV4-72*01 F	100	CAAATTGTTCTCTCCCAGTCTCC AGCAATCCTGTCTGCATCTCCA GGGGAGAAGGTCACAATGACTT GCAGGGCCAGCTCAAGTGTAA GTTACATGCACTGGTACCAGCA GAAGCCAGGATCCTCCCCCAA ACCCTGGATTTATGCCACATCC AACCTGGCTTCTGGAGTCCCTG CTCGCTTCAGTGGCAGTGGGTC TGGGACCTCTTACTCTCTCACAA TCAGCAGAGTGGAGGCTGAAG ATGCTGCCACTTATTACTGCCAG CAGTGGAGTAGTAACCCACTCA
IGKV4-72*01_S3540_LEWES	LEWES/EIJ	IGKV	MUSMUS IGKV4-72*01 F	100	CAAATTGTTCTCTCCCAGTCTCC AGCAATCCTGTCTGCATCTCCA GGGGAGAAGGTCACAATGACTT GCAGGGCCAGCTCAAGTGTAA GTTACATGCACTGGTACCAGCA GAAGCCAGGATCCTCCCCCAA ACCCTGGATTTATGCCACATCC AACCTGGCTTCTGGAGTCCCTG CTCGCTTCAGTGGCAGTGGGTC TGGGACCTCTTACTCTCTCACAA TCAGCAGAGTGGAGGCTGAAG ATGCTGCCACTTATTACTGCCAG CAGTGGAGTAGTAACCCACTCA

IGKV4-72*01_S3540_SJL	SJL/J	IGKV	MUSMUS IGKV4-72*01 F	100	CAAATTGTTCTCTCCCAGTCTCCAGCAATCCTGTCTGCATCTCCAGGGGAGAAGGTCACAATGACTTGCAGGGCCAGCTCAAGTGTAAGTTACATGCACTGGTACCAGCAGAAGCCAGGATCCTCCCCCAAACCCTGGATTTATGCCACATCCAACCTGGCTTCTGGAGTCCCTGCTCGCTTCAGTGGCAGTGGGTCTGGGACCTCTTACTCTCTCACAATCAGCAGAGTGGAGGCTGAAGATGCTGCCACTTATTACTGCCAGCAGTGGAGTAGTAACCCACTCA
IGKV4-73*01_BALB	BALB/CBYJ	IGKV	MUSMUS IGKV4-73*01 ORF	100	CAAATTGTTCTCACCCAGTCTCCAGCAATCATGTCTGCATCTCCTGGGGAACGGGTCACCATGACCTGCAGTGCCAGCTCAAGTGTAAGTTCCAGCTACTTGTACTGGTACCAGCAGAAGTCAGGATCCTCCCCAAAACTCTGGATTTATAGCATATCCAACCTGGCTTCTGGAGTCCCAGCTCGCTTCAGTGGCAGTGGGTCTGGGACCTCTTACTCTCTCACAATCAACAGCATGGAGGCTGAAGATGCTGCCACTTATTACTGCCAGCAGTGGAGTAGTAACCCACCCA
IGKV4-73*01_NZB	NZB/BLNJ	IGKV	MUSMUS IGKV4-73*01 ORF	100	CAAATTGTTCTCACCCAGTCTCCAGCAATCATGTCTGCATCTCCTGGGGAACGGGTCACCATGACCTGCAGTGCCAGCTCAAGTGTAAGTTCCAGCTACTTGTACTGGTACCAGCAGAAGTCAGGATCCTCCCCAAAACTCTGGATTTATAGCATATCCAACCTGGCTTCTGGAGTCCCAGCTCGCTTCAGTGGCAGTGGGTCTGGGACCTCTTACTCTCTCACAATCAACAGCATGGAGGCTGAAGATGCTGCCACTTATTACTGCCAGCAGTGGAGTAGTAACCCACCCA
IGKV4-73*01_S3184_PWD	PWD/PHJ	IGKV	MUSMUS IGKV4-73*01 ORF	97.87	CAAATTGTTCTCACCCAGTCTCCAGCAATCATGTCTGCATCTCCTGGGGAACGGGTCACCATGACCTGCAGTGCCAGCTCAAGTGTAAGTTCCAGCTACTTGTACTGGTACCAGCAGAAGCCAGGATCCTCCCCCAAACTCTGGATTTATAGCACATCCAACCTGGCTTCTGGAGTCCCTGCTCGCTTCAGTGGCAGTGGGTCTGGGACCTCTTATTCTCTCACAATCAGCAGCATGGAGGCTGAAGATGCTGCCACTTATTACTGCCAGCAGTGGAGTAGTAACCAACCCA

IGKV4-73*01_S7826_MSM	MSM/MSJ	IGKV	MUSMUS IGKV4-73*01 ORF	97.87	CAAATTGTTCTCACCCAGTCTCCAGCAATCATGTCTGCATCTCCTGGGGAACGGGTCACCATGACCTGCAGTGCCAGCTCAAGTGTAAGTTCCAGCTACTTGCACTGGTACCAGCAGAAGCTAGGTTCCTCCCCAAAACTCTGGATTTATAGCACATCCAACCTGGCTTCTGGAGTCCCAGCTCGCTTCAGTGGCAGTGGGTCTGGGACCTCTTACTCTCTCACAATCAGCAGCATGGAGGCTGAAGATGCTGCCACTTATTACTGCCAGCAGTGGAGTAGTAACCAACTCA
IGKV4-73*01_S8836_B6	C57BL/6J	IGKV	MUSMUS IGKV4-73*01 ORF	100	CAAATTGTTCTCACCCAGTCTCCAGCAATCATGTCTGCATCTCCTGGGGAACGGGTCACCATGACCTGCAGTGCCAGCTCAAGTGTAAGTTCCAGCTACTTGTACTGGTACCAGCAGAAGTCAGGATCCTCCCCAAAACTCTGGATTTATAGCATATCCAACCTGGCTTCTGGAGTCCCAGCTCGCTTCAGTGGCAGTGGGTCTGGGACCTCTTACTCTCTCACAATCAACAGCATGGAGGCTGAAGATGCTGCCACTTATTACTGCCAGCAGTGGAGTAGTAACCCACTCA
IGKV4-73*01_S8836_DBA1	DBA/1J	IGKV	MUSMUS IGKV4-73*01 ORF	100	CAAATTGTTCTCACCCAGTCTCCAGCAATCATGTCTGCATCTCCTGGGGAACGGGTCACCATGACCTGCAGTGCCAGCTCAAGTGTAAGTTCCAGCTACTTGTACTGGTACCAGCAGAAGTCAGGATCCTCCCCAAAACTCTGGATTTATAGCATATCCAACCTGGCTTCTGGAGTCCCAGCTCGCTTCAGTGGCAGTGGGTCTGGGACCTCTTACTCTCTCACAATCAACAGCATGGAGGCTGAAGATGCTGCCACTTATTACTGCCAGCAGTGGAGTAGTAACCCACTCA
IGKV4-73*01_S9912_NZB	NZB/BLNJ	IGKV	MUSMUS IGKV4-73*01 ORF	100	CAAATTGTTCTCACCCAGTCTCCAGCAATCATGTCTGCATCTCCTGGGGAACGGGTCACCATGACCTGCAGTGCCAGCTCAAGTGTAAGTTCCAGCTACTTGTACTGGTACCAGCAGAAGTCAGGATCCTCCCCAAAACTCTGGATTTATAGCATATCCAACCTGGCTTCTGGAGTCCCAGCTCGCTTCAGTGGCAGTGGGTCTGGGACCTCTTACTCTCTCACAATCAACAGCATGGAGGCTGAAGATGCTGCCACTTATTACTGCCAGCAGTGGAGTAGTAACCCGCTCA

IGKV4-74*01_129	129S1/SVIMJ	IGKV	MUSMUS IGKV4-74*01 F	100	CAAATTGTTCTCACCCAGTCTCCAGCAATCATGTCTGCATCTCTAGGGGAACGGGTCACCATGACCTGCACTGCCAGCTCAAGTGTAAGTTCCAGTTACTTGCACTGGTACCAGCAGAAGCCAGGATCCTCCCCCAAACTCTGGATTTATAGCACATCCAACCTGGCTTCTGGAGTCCCAGCTCGCTTCAGTGGCAGTGGGTCTGGGACCTCTTACTCTCTCACAATCAGCAGCATGGAGGCTGAAGATGCTGCCACTTATTACTGCCACCAGTATCATCGTTCCCCACCCA
IGKV4-74*01_B6	C57BL/6J	IGKV	MUSMUS IGKV4-74*01 F	100	CAAATTGTTCTCACCCAGTCTCCAGCAATCATGTCTGCATCTCTAGGGGAACGGGTCACCATGACCTGCACTGCCAGCTCAAGTGTAAGTTCCAGTTACTTGCACTGGTACCAGCAGAAGCCAGGATCCTCCCCCAAACTCTGGATTTATAGCACATCCAACCTGGCTTCTGGAGTCCCAGCTCGCTTCAGTGGCAGTGGGTCTGGGACCTCTTACTCTCTCACAATCAGCAGCATGGAGGCTGAAGATGCTGCCACTTATTACTGCCACCAGTATCATCGTTCCCCACCCA
IGKV4-74*01_BALB	BALB/CBYJ	IGKV	MUSMUS IGKV4-74*01 F	100	CAAATTGTTCTCACCCAGTCTCCAGCAATCATGTCTGCATCTCTAGGGGAACGGGTCACCATGACCTGCACTGCCAGCTCAAGTGTAAGTTCCAGTTACTTGCACTGGTACCAGCAGAAGCCAGGATCCTCCCCCAAACTCTGGATTTATAGCACATCCAACCTGGCTTCTGGAGTCCCAGCTCGCTTCAGTGGCAGTGGGTCTGGGACCTCTTACTCTCTCACAATCAGCAGCATGGAGGCTGAAGATGCTGCCACTTATTACTGCCACCAGTATCATCGTTCCCCACCCA
IGKV4-74*01_CBA	CBA/J	IGKV	MUSMUS IGKV4-74*01 F	100	CAAATTGTTCTCACCCAGTCTCCAGCAATCATGTCTGCATCTCTAGGGGAACGGGTCACCATGACCTGCACTGCCAGCTCAAGTGTAAGTTCCAGTTACTTGCACTGGTACCAGCAGAAGCCAGGATCCTCCCCCAAACTCTGGATTTATAGCACATCCAACCTGGCTTCTGGAGTCCCAGCTCGCTTCAGTGGCAGTGGGTCTGGGACCTCTTACTCTCTCACAATCAGCAGCATGGAGGCTGAAGATGCTGCCACTTATTACTGCCACCAGTATCATCGTTCCCCACCCA

IGKV4-74*01_DBA2	DBA/2J	IGKV	MUSMUS IGKV4-74*01 F	100	CAAATTGTTCTCACCCAGTCTCCAGCAATCATGTCTGCATCTCTAGGGGAACGGGTCACCATGACCTGCACTGCCAGCTCAAGTGTAAGTTCCAGTTACTTGCACTGGTACCAGCAGAAGCCAGGATCCTCCCCCAAACTCTGGATTTATAGCACATCCAACCTGGCTTCTGGAGTCCCAGCTCGCTTCAGTGGCAGTGGGTCTGGGACCTCTTACTCTCTCACAATCAGCAGCATGGAGGCTGAAGATGCTGCCACTTATTACTGCCACCAGTATCATCGTTCCCCACCCA
IGKV4-74*01_LEWES	LEWES/EIJ	IGKV	MUSMUS IGKV4-74*01 F	100	CAAATTGTTCTCACCCAGTCTCCAGCAATCATGTCTGCATCTCTAGGGGAACGGGTCACCATGACCTGCACTGCCAGCTCAAGTGTAAGTTCCAGTTACTTGCACTGGTACCAGCAGAAGCCAGGATCCTCCCCCAAACTCTGGATTTATAGCACATCCAACCTGGCTTCTGGAGTCCCAGCTCGCTTCAGTGGCAGTGGGTCTGGGACCTCTTACTCTCTCACAATCAGCAGCATGGAGGCTGAAGATGCTGCCACTTATTACTGCCACCAGTATCATCGTTCCCCACCCA
IGKV4-74*01_NZB	NZB/BLNJ	IGKV	MUSMUS IGKV4-74*01 F	100	CAAATTGTTCTCACCCAGTCTCCAGCAATCATGTCTGCATCTCTAGGGGAACGGGTCACCATGACCTGCACTGCCAGCTCAAGTGTAAGTTCCAGTTACTTGCACTGGTACCAGCAGAAGCCAGGATCCTCCCCCAAACTCTGGATTTATAGCACATCCAACCTGGCTTCTGGAGTCCCAGCTCGCTTCAGTGGCAGTGGGTCTGGGACCTCTTACTCTCTCACAATCAGCAGCATGGAGGCTGAAGATGCTGCCACTTATTACTGCCACCAGTATCATCGTTCCCCACCCA
IGKV4-74*01_S0897_NOD	NOD/SHILTJ	IGKV	MUSMUS IGKV4-74*01 F	94.68	CAGATTGTTCTCACCCAGTCTCCAGCAATCATGTCTGCATCTCCAGGGGAGAAGGTCACCATGACCTGCAGGGCCAGCTCAAGTGTAAGTTCCAGTTACTTGCACTGGTACCAGCAGAAGCCAGGATCTTCCCCCAAACTCTGGATTTATAGCACATCCAACCTGGCTTCAGGAGTCCCAGCTCGCTTCAGTGGCAGTGGGTCTGGGACCTCTTACTCTCTCACAATCAGCAGTGTGGAGGCTGAGGATGCTGCCACTTATTACTGCCAGCAGTATGATAGTTCCCCACTCA

IGKV4-74*01_S2000_NOR	NOR/LTJ	IGKV	MUSMUS IGKV4-74*01 F	94.68	CAGATTGTTCTCACCCAGTCTCCAGCAATCATGTCTGCATCTCCAGGGGAGAAGGTCACCATGACCTGCAGGGCCAGCTCAAGTGTAAGTTCCAGTTACTTGCACTGGTACCAGCAGAAGCCAGGATCTTCCCCCAAACTCTGGATTTATAGCACATCCAACCTGGCTTCAGGAGTCCCAGCTCGCTTCAGTGGCAGTGGGTCTGGGACCTCTTACTCTCTCACAATCAGCAGTGTGGAGGCTGAGGATGCTGCCACTTATTACTGCCAGCAGTATGATAGTTCCCCATCCA
IGKV4-74*01_S2922_129	129S1/SVIMJ	IGKV	MUSMUS IGKV4-74*01 F	100	CAAATTGTTCTCACCCAGTCTCCAGCAATCATGTCTGCATCTCTAGGGGAACGGGTCACCATGACCTGCACTGCCAGCTCAAGTGTAAGTTCCAGTTACTTGCACTGGTACCAGCAGAAGCCAGGATCCTCCCCCAAACTCTGGATTTATAGCACATCCAACCTGGCTTCTGGAGTCCCAGCTCGCTTCAGTGGCAGTGGGTCTGGGACCTCTTACTCTCTCACAATCAGCAGCATGGAGGCTGAAGATGCTGCCACTTATTACTGCCACCAGTATCATCGTTCCCCACTCA
IGKV4-74*01_S2922_AJ	A/J	IGKV	MUSMUS IGKV4-74*01 F	100	CAAATTGTTCTCACCCAGTCTCCAGCAATCATGTCTGCATCTCTAGGGGAACGGGTCACCATGACCTGCACTGCCAGCTCAAGTGTAAGTTCCAGTTACTTGCACTGGTACCAGCAGAAGCCAGGATCCTCCCCCAAACTCTGGATTTATAGCACATCCAACCTGGCTTCTGGAGTCCCAGCTCGCTTCAGTGGCAGTGGGTCTGGGACCTCTTACTCTCTCACAATCAGCAGCATGGAGGCTGAAGATGCTGCCACTTATTACTGCCACCAGTATCATCGTTCCCCACTCA
IGKV4-74*01_S2922_C3H	C3H/HEJ	IGKV	MUSMUS IGKV4-74*01 F	100	CAAATTGTTCTCACCCAGTCTCCAGCAATCATGTCTGCATCTCTAGGGGAACGGGTCACCATGACCTGCACTGCCAGCTCAAGTGTAAGTTCCAGTTACTTGCACTGGTACCAGCAGAAGCCAGGATCCTCCCCCAAACTCTGGATTTATAGCACATCCAACCTGGCTTCTGGAGTCCCAGCTCGCTTCAGTGGCAGTGGGTCTGGGACCTCTTACTCTCTCACAATCAGCAGCATGGAGGCTGAAGATGCTGCCACTTATTACTGCCACCAGTATCATCGTTCCCCACTCA

IGKV4-74*01_S2922_CBA	CBA/J	IGKV	MUSMUS IGKV4-74*01 F	100	CAAATTGTTCTCACCCAGTCTCCAGCAATCATGTCTGCATCTCTAGGGGAACGGGTCACCATGACCTGCACTGCCAGCTCAAGTGTAAGTTCCAGTTACTTGCACTGGTACCAGCAGAAGCCAGGATCCTCCCCCAAACTCTGGATTTATAGCACATCCAACCTGGCTTCTGGAGTCCCAGCTCGCTTCAGTGGCAGTGGGTCTGGGACCTCTTACTCTCTCACAATCAGCAGCATGGAGGCTGAAGATGCTGCCACTTATTACTGCCACCAGTATCATCGTTCCCCACTCA
IGKV4-74*01_S2922_DBA1	DBA/1J	IGKV	MUSMUS IGKV4-74*01 F	100	CAAATTGTTCTCACCCAGTCTCCAGCAATCATGTCTGCATCTCTAGGGGAACGGGTCACCATGACCTGCACTGCCAGCTCAAGTGTAAGTTCCAGTTACTTGCACTGGTACCAGCAGAAGCCAGGATCCTCCCCCAAACTCTGGATTTATAGCACATCCAACCTGGCTTCTGGAGTCCCAGCTCGCTTCAGTGGCAGTGGGTCTGGGACCTCTTACTCTCTCACAATCAGCAGCATGGAGGCTGAAGATGCTGCCACTTATTACTGCCACCAGTATCATCGTTCCCCACTCA
IGKV4-74*01_S3301_AKR	AKR/J	IGKV	MUSMUS IGKV4-74*01 F	94.68	CAGATTGTTCTCACCCAGTCTCCAGCAATCATGTCTGCATCTCCAGGGGAGAAGGTCACCATGACCTGCAGGGCCAGCTCAAGTGTAAGTTCCAGTTACTTGCACTGGTACCAGCAGAAGCCAGGATCTTCCCCCAAACTCTGGATTTATAGCACATCCAACCTGGCTTCAGGAGTCCCAGCTCGCTTCAGTGGCAGTGGGTCTGGGACCTCTTACTCTCTCACAATCAGCAGTGTGGAGGCTGAGGATGCTGCCACTTATTACTGCCAGCAGTATGATAGTTCCCCACCCA
IGKV4-74*01_S3301_MRL	MRL/MPJ	IGKV	MUSMUS IGKV4-74*01 F	94.68	CAGATTGTTCTCACCCAGTCTCCAGCAATCATGTCTGCATCTCCAGGGGAGAAGGTCACCATGACCTGCAGGGCCAGCTCAAGTGTAAGTTCCAGTTACTTGCACTGGTACCAGCAGAAGCCAGGATCTTCCCCCAAACTCTGGATTTATAGCACATCCAACCTGGCTTCAGGAGTCCCAGCTCGCTTCAGTGGCAGTGGGTCTGGGACCTCTTACTCTCTCACAATCAGCAGTGTGGAGGCTGAGGATGCTGCCACTTATTACTGCCAGCAGTATGATAGTTCCCCACCCA

IGKV4-74*01_S3301_NOD	NOD/SHILTJ	IGKV	MUSMUS IGKV4-74*01 F	94.68	CAGATTGTTCTCACCCAGTCTC<br>CAGCAATCATGTCTGCATCTCC<br>AGGGGAGAAGGTCACCATGAC<br>CTGCAGGGCCAGCTCAAGTGTA<br>AGTTCCAGTTACTTGCACTGGTA<br>CCAGCAGAAGCCAGGATCTTC<br>CCCCAAACTCTGGATTTATAGCA<br>CATCCAACCTGGCTTCAGGAGT<br>CCCAGCTCGCTTCAGTGGCAGT<br>GGGTCTGGGACCTCTTACTCTC<br>TCACAATCAGCAGTGTGGAGGC<br>TGAGGATGCTGCCACTTATTACT<br>GCCAGCAGTATGATAGTTCCCC<br>ACCCA
IGKV4-74*01_S3773_CAST	CAST/EIJ	IGKV	MUSMUS IGKV4-74*01 F	95.04	CAAATTGTTCTCACCCAGTCTCC<br>AGCAATCATGTCTGCATCTCCA<br>GGGGAGAAGGTCACCATGACC<br>TGCAGGGCCAGCTCAAGTGTAA<br>GTTCCAGTTACTTGCACTGGTAC<br>CAGCAGAAGCCAGGATCTTCC<br>CCCAAACTCTGGATTTATAGCAC<br>ATCCAACCTGGCTTCAGGAGTC<br>CCAGCTCGCTTCAGTGGCAGTG<br>GGTCTGGGACCTCTTACTCTCT<br>CACAATCAGCAGTGTGGAGGCT<br>GAGGATGCTGCCACTTATTACT<br>GCCAGCAGTATGATAGTTCCCC<br>ATCCA
IGKV4-74*01_S3912_AKR	AKR/J	IGKV	MUSMUS IGKV4-74*01 F	95.04	CAAATTGTTCTCACCCAGTCTCC<br>AGCAATCATGTCTGCCTCTCCA<br>GGGGAGAAGGTCACCATGACC<br>TGCAGTGCCAGCTCAAGTGTAA<br>GTTCCAGGTACTTGCACTGGTA<br>CCAGCAGAAGTCAGGAGCCTC<br>CCCCAAACTCTGGATTTATGGC<br>ACATCCAACCTGGCTTCTGGAG<br>TCCCTGCTCGCTTCAGTGGCAG<br>TGGGTCTGGGACCTCTTACTCT<br>CTCACAATCAGCAGCGTGGAG<br>GCTGAAGATGCTGCCACTTATTA<br>CTGCCAGCAGTATCATAGTGAC<br>CCACCCA
IGKV4-74*01_S3912_MRL	MRL/MPJ	IGKV	MUSMUS IGKV4-74*01 F	95.04	CAAATTGTTCTCACCCAGTCTCC<br>AGCAATCATGTCTGCCTCTCCA<br>GGGGAGAAGGTCACCATGACC<br>TGCAGTGCCAGCTCAAGTGTAA<br>GTTCCAGGTACTTGCACTGGTA<br>CCAGCAGAAGTCAGGAGCCTC<br>CCCCAAACTCTGGATTTATGGC<br>ACATCCAACCTGGCTTCTGGAG<br>TCCCTGCTCGCTTCAGTGGCAG<br>TGGGTCTGGGACCTCTTACTCT<br>CTCACAATCAGCAGCGTGGAG<br>GCTGAAGATGCTGCCACTTATTA<br>CTGCCAGCAGTATCATAGTGAC<br>CCACCCA

IGKV4-74*01_S3912_NOD	NOD/SHILTJ	IGKV	MUSMUS IGKV4-74*01 F	95.04	CAAATTGTTCTCACCCAGTCTCCAGCAATCATGTCTGCCTCTCCAGGGGAGAAGGTCACCATGACCTGCAGTGCCAGCTCAAGTGTAAGTTCCAGGTACTTGCACTGGTACCAGCAGAAGTCAGGAGCCTCCCCCAAACTCTGGATTTATGGCACATCCAACCTGGCTTCTGGAGTCCCTGCTCGCTTCAGTGGCAGTGGGTCTGGGACCTCTTACTCTCTCACAATCAGCAGCGTGGAGGCTGAAGATGCTGCCACTTATTACTGCCAGCAGTATCATAGTGACCCACCCA
IGKV4-74*01_S3912_NOR	NOR/LTJ	IGKV	MUSMUS IGKV4-74*01 F	95.04	CAAATTGTTCTCACCCAGTCTCCAGCAATCATGTCTGCCTCTCCAGGGGAGAAGGTCACCATGACCTGCAGTGCCAGCTCAAGTGTAAGTTCCAGGTACTTGCACTGGTACCAGCAGAAGTCAGGAGCCTCCCCCAAACTCTGGATTTATGGCACATCCAACCTGGCTTCTGGAGTCCCTGCTCGCTTCAGTGGCAGTGGGTCTGGGACCTCTTACTCTCTCACAATCAGCAGCGTGGAGGCTGAAGATGCTGCCACTTATTACTGCCAGCAGTATCATAGTGACCCACCCA
IGKV4-74*01_S4432_CAST	CAST/EIJ	IGKV	MUSMUS IGKV4-74*01 F	95.04	CAAATTGTTCTCACCCAGTCTCCAGCAATCATGTCTGCATCTCCAGGGGAGAAGGTCACCATGACCTGCAGGGCCAGCTCAAGTGTAAGTTCCAGTTACTTGCACTGGTACCAGCAGAAGCCAGGATCTTCCCCCAAACTCTGGATTTATAGCACATCCAACCTGGCTTCAGGAGTCCCAGCTCGCTTCAGTGGCAGTGGGTCTGGGACCTCTTACTCTCTCACAATCAGCAGTGTGGAGGCTGAGGATGCTGCCACTTATTACTGCCAGCAGTATGATAGTTCCCCACTCA
IGKV4-74*01_S8852_MSM	MSM/MSJ	IGKV	MUSMUS IGKV4-74*01 F	94.33	CAAATTGTTCTCACCCAGTCTCCAGCAATCATGTCTGCATCTCCAGGGGAGAAGGTCACCATGACCTGCAGGGCCAGCTCAAGTGTACGTTCCAGTTACTTGCACTGGTACCAGCAGAAGCCAGGATCTTCCCCCAAACTCTGGATTTATAGAACATCCAACCTGGCTTCAGGAGTCCCAGCTCGCTTCAGTGGCAGTGGGTCTGGGACCTCTTACTCTCTCACAATCAGCAGTGTGGAGGCTGAGGATGCTGCCACTTATTACTGCCAGCAGTATGATAGTTCCCCATCCA

IGKV4-74*01_S8852_PWD	PWD/PHJ	IGKV	MUSMUS IGKV4-74*01 F	94.33	CAAATTGTTCTCACCCAGTCTCCAGCAATCATGTCTGCATCTCCAGGGGAGAAGGTCACCATGACCTGCAGGGCCAGCTCAAGTGTACGTTCCAGTTACTTGCACTGGTACCAGCAGAAGCCAGGATCTTCCCCCAAACTCTGGATTTATAGAACATCCAACCTGGCTTCAGGAGTCCCAGCTCGCTTCAGTGGCAGTGGGTCTGGGACCTCTTACTCTCTCACAATCAGCAGTGTGGAGGCTGAGGATGCTGCCACTTATTACTGCCAGCAGTATGATAGTTCCCCATCCA
IGKV4-74*01_SJL	SJL/J	IGKV	MUSMUS IGKV4-74*01 F	100	CAAATTGTTCTCACCCAGTCTCCAGCAATCATGTCTGCATCTCTAGGGGAACGGGTCACCATGACCTGCACTGCCAGCTCAAGTGTAAGTTCCAGTTACTTGCACTGGTACCAGCAGAAGCCAGGATCCTCCCCCAAACTCTGGATTTATAGCACATCCAACCTGGCTTCTGGAGTCCCAGCTCGCTTCAGTGGCAGTGGGTCTGGGACCTCTTACTCTCTCACAATCAGCAGCATGGAGGCTGAAGATGCTGCCACTTATTACTGCCACCAGTATCATCGTTCCCCACCCA
IGKV4-77*01_S4503_CAST	CAST/EIJ	IGKV	MUSMUS IGKV4-77*01 P	97.83	CAAATTGTTCTCACCCAGTCTCCAGCAATCCTGACTGCATCTCCAGGGGAGAAGGTCACCATGACCTGCAGTGCCAGCTCAAGTGTAAGTTACATGTACTGGTTCCAGCAGAAGCCAGGATCCTCCCCCAAACTCTGGATTTATAGCATATCCAACCTGGCTTCTGGAGTCCCTGCTCGCTTCAGTGGCAGTGGGTCTGGGACCTCTTACTCTCTTACAATCAGCAGTGTGAAGGCTGAAGATGCTGCCACTTATTACTGCCAGCAATGGAGTAGTTCCCCACCCA
IGKV4-78*01_AJ	A/J	IGKV	MUSMUS IGKV4-78*01 F	100	CAAATTGTTCTCACCCAGTCTCCAGCAATCATGTCTGCATCTCCTGGGGAGAAGGTCACCATGACCTGCAGTGCCAGATCAAGTGTAAGTTCCAGCTACTTGTACTGGTACCAGCAGAAGCCAGGATCCTCCCCCAAACTCTGGATTTATAGCACATCCAACCTGGCTTCTGGAGTCCCTGCTCGCTTCAGTGGCAGTGGGTCTGGGACCTCTTATTCTCTCACAATCAGCAGCATGGAGGCTGAAGATGCTGCCACTTTTTACTGCCAGCAGTACAGTGGTTACCCATCCA

IGKV4-78*01_B6	C57BL/6J	IGKV	MUSMUS IGKV4-78*01 F	100	CAAATTGTTCTCACCCAGTCTCCAGCAATCATGTCTGCATCTCCTGGGGAGAAGGTCACCATGACCTGCAGTGCCAGATCAAGTGTAAGTTCCAGCTACTTGTACTGGTACCAGCAGAAGCCAGGATCCTCCCCCAAACTCTGGATTTATAGCACATCCAACCTGGCTTCTGGAGTCCCTGCTCGCTTCAGTGGCAGTGGGTCTGGGACCTCTTATTCTCTCACAATCAGCAGCATGGAGGCTGAAGATGCTGCCACTTTTTACTGCCAGCAGTACAGTGGTTACCCATCCA
IGKV4-78*01_BALB	BALB/CBYJ	IGKV	MUSMUS IGKV4-78*01 F	100	CAAATTGTTCTCACCCAGTCTCCAGCAATCATGTCTGCATCTCCTGGGGAGAAGGTCACCATGACCTGCAGTGCCAGATCAAGTGTAAGTTCCAGCTACTTGTACTGGTACCAGCAGAAGCCAGGATCCTCCCCCAAACTCTGGATTTATAGCACATCCAACCTGGCTTCTGGAGTCCCTGCTCGCTTCAGTGGCAGTGGGTCTGGGACCTCTTATTCTCTCACAATCAGCAGCATGGAGGCTGAAGATGCTGCCACTTTTTACTGCCAGCAGTACAGTGGTTACCCATCCA
IGKV4-78*01_C3H	C3H/HEJ	IGKV	MUSMUS IGKV4-78*01 F	100	CAAATTGTTCTCACCCAGTCTCCAGCAATCATGTCTGCATCTCCTGGGGAGAAGGTCACCATGACCTGCAGTGCCAGATCAAGTGTAAGTTCCAGCTACTTGTACTGGTACCAGCAGAAGCCAGGATCCTCCCCCAAACTCTGGATTTATAGCACATCCAACCTGGCTTCTGGAGTCCCTGCTCGCTTCAGTGGCAGTGGGTCTGGGACCTCTTATTCTCTCACAATCAGCAGCATGGAGGCTGAAGATGCTGCCACTTTTTACTGCCAGCAGTACAGTGGTTACCCATCCA
IGKV4-78*01_DBA2	DBA/2J	IGKV	MUSMUS IGKV4-78*01 F	100	CAAATTGTTCTCACCCAGTCTCCAGCAATCATGTCTGCATCTCCTGGGGAGAAGGTCACCATGACCTGCAGTGCCAGATCAAGTGTAAGTTCCAGCTACTTGTACTGGTACCAGCAGAAGCCAGGATCCTCCCCCAAACTCTGGATTTATAGCACATCCAACCTGGCTTCTGGAGTCCCTGCTCGCTTCAGTGGCAGTGGGTCTGGGACCTCTTATTCTCTCACAATCAGCAGCATGGAGGCTGAAGATGCTGCCACTTTTTACTGCCAGCAGTACAGTGGTTACCCATCCA

IGKV4-78*01_S3637_CAST	CAST/EIJ	IGKV	MUSMUS IGKV4-78*01 F	98.23	CAAATTGTTCTCACCCAGTCTCCAGCAATCATGTCTGCATCTCCTGGGGAACGGGTCACCATGACCTGCAGTGCCAGCTCAAGTGTAAGTTCCAGCTACTTGTACTGGTACCAGCAGAAGCCAGGATCCTCCCCCAAACTCTGGATTTATAGCACATCCAACCTGGCTTCTGGAGTCCCTGCTCGCTTCAGTGGCAGTGGGTCTGGGACCTCTTATTCTCTCACAATCAGCAGCATGGAGGCTGAAGATGCTGCCACTTATTACTGCCAGCAGTACAGTGGTTACCCATTCA
IGKV4-78*01_S3978_AKR	AKR/J	IGKV	MUSMUS IGKV4-78*01 F	97.87	CAAATTGTTCTCACCCAGTCTCCAGCAATCATGTCTGCATCTCCTGGGGAACGGGTCACCATGACCTGCAGTGCCAGCTCAAGTGTAAGTTCCAGCTACTTGTACTGGTACCAGCAGAAGCCAGGATCCTCCCCCAAACTATGGATTTATAGCACATCCAACCTGGCTTCTGGAGTCCCTGCTCGCTTCAGTGGCAGTGGGTCTGGGACCTCTTATTCTCTCACAATCAGCAGCATGGAGGCTGAAGATGCTGCCACTTATTACTGCCAGCAGTACAGTGGTTACCCATCCA
IGKV4-78*01_S3978_MRL	MRL/MPJ	IGKV	MUSMUS IGKV4-78*01 F	97.87	CAAATTGTTCTCACCCAGTCTCCAGCAATCATGTCTGCATCTCCTGGGGAACGGGTCACCATGACCTGCAGTGCCAGCTCAAGTGTAAGTTCCAGCTACTTGTACTGGTACCAGCAGAAGCCAGGATCCTCCCCCAAACTATGGATTTATAGCACATCCAACCTGGCTTCTGGAGTCCCTGCTCGCTTCAGTGGCAGTGGGTCTGGGACCTCTTATTCTCTCACAATCAGCAGCATGGAGGCTGAAGATGCTGCCACTTATTACTGCCAGCAGTACAGTGGTTACCCATCCA
IGKV4-78*01_S3978_NOD	NOD/SHILTJ	IGKV	MUSMUS IGKV4-78*01 F	97.87	CAAATTGTTCTCACCCAGTCTCCAGCAATCATGTCTGCATCTCCTGGGGAACGGGTCACCATGACCTGCAGTGCCAGCTCAAGTGTAAGTTCCAGCTACTTGTACTGGTACCAGCAGAAGCCAGGATCCTCCCCCAAACTATGGATTTATAGCACATCCAACCTGGCTTCTGGAGTCCCTGCTCGCTTCAGTGGCAGTGGGTCTGGGACCTCTTATTCTCTCACAATCAGCAGCATGGAGGCTGAAGATGCTGCCACTTATTACTGCCAGCAGTACAGTGGTTACCCATCCA

IGKV4-78*01_S4828_NOD	NOD/SHILTJ	IGKV	MUSMUS IGKV4-78*01 F	97.52	CAAATTGTTCTCACCCAGTCTCCAGCAATCATGTCTGCATCTCCTGGGGAACGGGTCACCATGACCTGCAGTGCCAGCTCAAGTGTAAGTTCCAGCTACTTGTACTGGTACCAGCAGAAGCCAGGATCCTCCCCCAAACTATGGATTTATAGCACATCCAACCTGGCTTCTGGAGTCCCTGCTCGCTTCAGTGGCAGTGGGTCTGGGACCTCTTATTCTCTCACAATCAGCAGCATGGAGGCTGAAGATGCTGCCACTTATTACTGCCAGCAGTACAATGGTTACCCATCCA
IGKV4-78*01_S5980_CBA	CBA/J	IGKV	MUSMUS IGKV4-78*01 F	100	CAAATTGTTCTCACCCAGTCTCCAGCAATCATGTCTGCATCTCCTGGGGAGAAGGTCACCATGACCTGCAGTGCCAGATCAAGTGTAAGTTCCAGCTACTTGTACTGGTACCAGCAGAAGCCAGGATCCTCCCCCAAACTCTGGATTTATAGCACATCCAACCTGGCTTCTGGAGTCCCTGCTCGCTTCAGTGGCAGTGGGTCTGGGACCTCTTATTCTCTCACAATCAGCAGCATGGAGGCTGAAGATGCTGCCACTTTTTACTGCCAGCAGTACAGTGGTTACCCATTCA
IGKV4-78*01_S6302_DBA1	DBA/1J	IGKV	MUSMUS IGKV4-78*01 F	100	CAAATTGTTCTCACCCAGTCTCCAGCAATCATGTCTGCATCTCCTGGGGAGAAGGTCACCATGACCTGCAGTGCCAGATCAAGTGTAAGTTCCAGCTACTTGTACTGGTACCAGCAGAAGCCAGGATCCTCCCCCAAACTCTGGATTTATAGCACATCCAACCTGGCTTCTGGAGTCCCTGCTCGCTTCAGTGGCAGTGGGTCTGGGACCTCTTATTCTCTCACAATCAGCAGCATGGAGGCTGAAGATGCTGCCACTTTTTACTGCCAGCAGTACAGTGGTTACCCATACA
IGKV4-78*01_S9188_NOR	NOR/LTJ	IGKV	MUSMUS IGKV4-78*01 F	97.87	CAAATTGTTCTCACCCAGTCTCCAGCAATCATGTCTGCATCTCCTGGGGAACGGGTCACCATGACCTGCAGTGCCAGCTCAAGTGTAAGTTCCAGCTACTTGTACTGGTACCAGCAGAAGCCAGGATCCTCCCCCAAACTATGGATTTATAGCACATCCAACCTGGCTTCTGGAGTCCCTGCTCGCTTCAGTGGCAGTGGGTCTGGGACCTCTTATTCTCTCACAATCAGCAGCATGGAGGCTGAAGATGCTGCCACTTATTACTGCCAGCAGTACAGTGGTTACCCATTCA

IGKV4-78*01_S9469_CAST	CAST/EIJ	IGKV	MUSMUS IGKV4-78*01 F	98.23	CAAATTGTTCTCACCCAGTCTCCAGCAATCATGTCTGCATCTCCTGGGGAACGGGTCACCATGACCTGCAGTGCCAGCTCAAGTGTAAGTTCCAGCTACTTGTACTGGTACCAGCAGAAGCCAGGATCCTCCCCCAAACTCTGGATTTATAGCACATCCAACCTGGCTTCTGGAGTCCCTGCTCGCTTCAGTGGCAGTGGGTCTGGGACCTCTTATTCTCTCACAATCAGCAGCATGGAGGCTGAAGATGCTGCCACTTATTACTGCCAGCAGTACAGTGGTTACCCATCCA
IGKV4-79*01_129	129S1/SVIMJ	IGKV	MUSMUS IGKV4-79*01 F	100	CAAATTGTTCTCACCCAGTCTCCAGCAATCATGTCTGCATCTCCTGGGGAGAAGGTCACCTTGACCTGCAGTGCCAGCTCAAGTGTAAGTTCCAGCTACTTGTACTGGTACCAGCAGAAGCCAGGATCCTCCCCCAAACTCTGGATTTATAGCACATCCAACCTGGCTTCTGGAGTCCCTGCTCGCTTCAGTGGCAGTGGGTCTGGGACCTCTTACTCTCTCACAATCAGCAGCATGGAGGCTGAAGATGCTGCCTCTTATTTCTGCCATCAGTGGAGTAGTTACCCACCCC
IGKV4-79*01_AJ	A/J	IGKV	MUSMUS IGKV4-79*01 F	100	CAAATTGTTCTCACCCAGTCTCCAGCAATCATGTCTGCATCTCCTGGGGAGAAGGTCACCTTGACCTGCAGTGCCAGCTCAAGTGTAAGTTCCAGCTACTTGTACTGGTACCAGCAGAAGCCAGGATCCTCCCCCAAACTCTGGATTTATAGCACATCCAACCTGGCTTCTGGAGTCCCTGCTCGCTTCAGTGGCAGTGGGTCTGGGACCTCTTACTCTCTCACAATCAGCAGCATGGAGGCTGAAGATGCTGCCTCTTATTTCTGCCATCAGTGGAGTAGTTACCCACCCC
IGKV4-79*01_B6	C57BL/6J	IGKV	MUSMUS IGKV4-79*01 F	100	CAAATTGTTCTCACCCAGTCTCCAGCAATCATGTCTGCATCTCCTGGGGAGAAGGTCACCTTGACCTGCAGTGCCAGCTCAAGTGTAAGTTCCAGCTACTTGTACTGGTACCAGCAGAAGCCAGGATCCTCCCCCAAACTCTGGATTTATAGCACATCCAACCTGGCTTCTGGAGTCCCTGCTCGCTTCAGTGGCAGTGGGTCTGGGACCTCTTACTCTCTCACAATCAGCAGCATGGAGGCTGAAGATGCTGCCTCTTATTTCTGCCATCAGTGGAGTAGTTACCCACCCC

IGKV4-79*01_BALB	BALB/CBYJ	IGKV	MUSMUS IGKV4-79*01 F	100	CAAATTGTTCTCACCCAGTCTCCAGCAATCATGTCTGCATCTCCTGGGGAGAAGGTCACCTTGACCTGCAGTGCCAGCTCAAGTGTAAGTTCCAGCTACTTGTACTGGTACCAGCAGAAGCCAGGATCCTCCCCCAAACTCTGGATTTATAGCACATCCAACCTGGCTTCTGGAGTCCCTGCTCGCTTCAGTGGCAGTGGGTCTGGGACCTCTTACTCTCTCACAATCAGCAGCATGGAGGCTGAAGATGCTGCCTCTTATTTCTGCCATCAGTGGAGTAGTTACCCACCCC
IGKV4-79*01_C3H	C3H/HEJ	IGKV	MUSMUS IGKV4-79*01 F	100	CAAATTGTTCTCACCCAGTCTCCAGCAATCATGTCTGCATCTCCTGGGGAGAAGGTCACCTTGACCTGCAGTGCCAGCTCAAGTGTAAGTTCCAGCTACTTGTACTGGTACCAGCAGAAGCCAGGATCCTCCCCCAAACTCTGGATTTATAGCACATCCAACCTGGCTTCTGGAGTCCCTGCTCGCTTCAGTGGCAGTGGGTCTGGGACCTCTTACTCTCTCACAATCAGCAGCATGGAGGCTGAAGATGCTGCCTCTTATTTCTGCCATCAGTGGAGTAGTTACCCACCCC
IGKV4-79*01_CBA	CBA/J	IGKV	MUSMUS IGKV4-79*01 F	100	CAAATTGTTCTCACCCAGTCTCCAGCAATCATGTCTGCATCTCCTGGGGAGAAGGTCACCTTGACCTGCAGTGCCAGCTCAAGTGTAAGTTCCAGCTACTTGTACTGGTACCAGCAGAAGCCAGGATCCTCCCCCAAACTCTGGATTTATAGCACATCCAACCTGGCTTCTGGAGTCCCTGCTCGCTTCAGTGGCAGTGGGTCTGGGACCTCTTACTCTCTCACAATCAGCAGCATGGAGGCTGAAGATGCTGCCTCTTATTTCTGCCATCAGTGGAGTAGTTACCCACCCC
IGKV4-79*01_DBA1	DBA/1J	IGKV	MUSMUS IGKV4-79*01 F	100	CAAATTGTTCTCACCCAGTCTCCAGCAATCATGTCTGCATCTCCTGGGGAGAAGGTCACCTTGACCTGCAGTGCCAGCTCAAGTGTAAGTTCCAGCTACTTGTACTGGTACCAGCAGAAGCCAGGATCCTCCCCCAAACTCTGGATTTATAGCACATCCAACCTGGCTTCTGGAGTCCCTGCTCGCTTCAGTGGCAGTGGGTCTGGGACCTCTTACTCTCTCACAATCAGCAGCATGGAGGCTGAAGATGCTGCCTCTTATTTCTGCCATCAGTGGAGTAGTTACCCACCCC

IGKV4-79*01_DBA2	DBA/2J	IGKV	MUSMUS IGKV4-79*01 F	100	CAAATTGTTCTCACCCAGTCTCCAGCAATCATGTCTGCATCTCCTGGGGAGAAGGTCACCTTGACCTGCAGTGCCAGCTCAAGTGTAAGTTCCAGCTACTTGTACTGGTACCAGCAGAAGCCAGGATCCTCCCCCAAACTCTGGATTTATAGCACATCCAACCTGGCTTCTGGAGTCCCTGCTCGCTTCAGTGGCAGTGGGTCTGGGACCTCTTACTCTCTCACAATCAGCAGCATGGAGGCTGAAGATGCTGCCTCTTATTTCTGCCATCAGTGGAGTAGTTACCCACCCC
IGKV4-79*01_LEWES	LEWES/EIJ	IGKV	MUSMUS IGKV4-79*01 F	100	CAAATTGTTCTCACCCAGTCTCCAGCAATCATGTCTGCATCTCCTGGGGAGAAGGTCACCTTGACCTGCAGTGCCAGCTCAAGTGTAAGTTCCAGCTACTTGTACTGGTACCAGCAGAAGCCAGGATCCTCCCCCAAACTCTGGATTTATAGCACATCCAACCTGGCTTCTGGAGTCCCTGCTCGCTTCAGTGGCAGTGGGTCTGGGACCTCTTACTCTCTCACAATCAGCAGCATGGAGGCTGAAGATGCTGCCTCTTATTTCTGCCATCAGTGGAGTAGTTACCCACCCC
IGKV4-79*01_NZB	NZB/BLNJ	IGKV	MUSMUS IGKV4-79*01 F	100	CAAATTGTTCTCACCCAGTCTCCAGCAATCATGTCTGCATCTCCTGGGGAGAAGGTCACCTTGACCTGCAGTGCCAGCTCAAGTGTAAGTTCCAGCTACTTGTACTGGTACCAGCAGAAGCCAGGATCCTCCCCCAAACTCTGGATTTATAGCACATCCAACCTGGCTTCTGGAGTCCCTGCTCGCTTCAGTGGCAGTGGGTCTGGGACCTCTTACTCTCTCACAATCAGCAGCATGGAGGCTGAAGATGCTGCCTCTTATTTCTGCCATCAGTGGAGTAGTTACCCACCCC
IGKV4-79*01_S6901_PWD	PWD/PHJ	IGKV	MUSMUS IGKV4-79*01 F	95.39	CAAATTGTTCTCACCCAGTCTCCAGCAATCATGTCTGCATCTCCAGGGGAGAAGGTCACCATGACCTGCAGGGCCAGCTCAAGTGTACGTTCCAGTTACTTGCACTGGTACCAGCAGAAGCCAGGATCTTCCCCCAAACTCTGGATTTATAGAACATCCAACCTGGCTTCAGGAGTCCCAGCTCGCTTCAGTGGCAGTGGGTCTGGGACCTCTTACTCTCTCACAATCAGCAGCATGGAGGCTGAAGATGCTGCCACTTATTACTGCCAGCAGTGGAGTAGTTACCCACCC

IGKV4-79*01_SJL	SJL/J	IGKV	MUSMUS IGKV4-79*01 F	100	CAAATTGTTCTCACCCAGTCTCCAGCAATCATGTCTGCATCTCCTGGGGAGAAGGTCACCTTGACCTGCAGTGCCAGCTCAAGTGTAAGTTCCAGCTACTTGTACTGGTACCAGCAGAAGCCAGGATCCTCCCCCAAACTCTGGATTTATAGCACATCCAACCTGGCTTCTGGAGTCCCTGCTCGCTTCAGTGGCAGTGGGTCTGGGACCTCTTACTCTCTCACAATCAGCAGCATGGAGGCTGAAGATGCTGCCTCTTATTTCTGCCATCAGTGGAGTAGTTACCCACCCC
IGKV4-80*01_129	129S1/SVIMJ	IGKV	MUSMUS IGKV4-80*01 F	100	CAAATTGTTCTCACCCAGTCTCCAGCAATCATGTCTGCATCTCTAGGGGAGGAGATCACCCTAACCTGCAGTGCCAGCTCGAGTGTAAGTTACATGCACTGGTACCAGCAGAAGTCAGGCACTTCTCCCAAACTCTTGATTTATAGCACATCCAACCTGGCTTCTGGAGTCCCTTCTCGCTTCAGTGGCAGTGGGTCTGGGACCTTTTATTCTCTCACAATCAGCAGTGTGGAGGCTGAAGATGCTGCCGATTATTACTGCCATCAGTGGAGTAGTTATCCA
IGKV4-80*01_AJ	A/J	IGKV	MUSMUS IGKV4-80*01 F	100	CAAATTGTTCTCACCCAGTCTCCAGCAATCATGTCTGCATCTCTAGGGGAGGAGATCACCCTAACCTGCAGTGCCAGCTCGAGTGTAAGTTACATGCACTGGTACCAGCAGAAGTCAGGCACTTCTCCCAAACTCTTGATTTATAGCACATCCAACCTGGCTTCTGGAGTCCCTTCTCGCTTCAGTGGCAGTGGGTCTGGGACCTTTTATTCTCTCACAATCAGCAGTGTGGAGGCTGAAGATGCTGCCGATTATTACTGCCATCAGTGGAGTAGTTATCCA
IGKV4-80*01_B6	C57BL/6J	IGKV	MUSMUS IGKV4-80*01 F	100	CAAATTGTTCTCACCCAGTCTCCAGCAATCATGTCTGCATCTCTAGGGGAGGAGATCACCCTAACCTGCAGTGCCAGCTCGAGTGTAAGTTACATGCACTGGTACCAGCAGAAGTCAGGCACTTCTCCCAAACTCTTGATTTATAGCACATCCAACCTGGCTTCTGGAGTCCCTTCTCGCTTCAGTGGCAGTGGGTCTGGGACCTTTTATTCTCTCACAATCAGCAGTGTGGAGGCTGAAGATGCTGCCGATTATTACTGCCATCAGTGGAGTAGTTATCCA

IGKV4-80*01_BALB	BALB/CBYJ	IGKV	MUSMUS IGKV4-80*01 F	100	CAAATTGTTCTCACCCAGTCTCCAGCAATCATGTCTGCATCTCTAGGGGAGGAGATCACCCTAACCTGCAGTGCCAGCTCGAGTGTAAGTTACATGCACTGGTACCAGCAGAAGTCAGGCACTTCTCCCAAACTCTTGATTTATAGCACATCCAACCTGGCTTCTGGAGTCCCTTCTCGCTTCAGTGGCAGTGGGTCTGGGACCTTTTATTCTCTCACAATCAGCAGTGTGGAGGCTGAAGATGCTGCCGATTATTACTGCCATCAGTGGAGTAGTTATCCA
IGKV4-80*01_C3H	C3H/HEJ	IGKV	MUSMUS IGKV4-80*01 F	100	CAAATTGTTCTCACCCAGTCTCCAGCAATCATGTCTGCATCTCTAGGGGAGGAGATCACCCTAACCTGCAGTGCCAGCTCGAGTGTAAGTTACATGCACTGGTACCAGCAGAAGTCAGGCACTTCTCCCAAACTCTTGATTTATAGCACATCCAACCTGGCTTCTGGAGTCCCTTCTCGCTTCAGTGGCAGTGGGTCTGGGACCTTTTATTCTCTCACAATCAGCAGTGTGGAGGCTGAAGATGCTGCCGATTATTACTGCCATCAGTGGAGTAGTTATCCA
IGKV4-80*01_CBA	CBA/J	IGKV	MUSMUS IGKV4-80*01 F	100	CAAATTGTTCTCACCCAGTCTCCAGCAATCATGTCTGCATCTCTAGGGGAGGAGATCACCCTAACCTGCAGTGCCAGCTCGAGTGTAAGTTACATGCACTGGTACCAGCAGAAGTCAGGCACTTCTCCCAAACTCTTGATTTATAGCACATCCAACCTGGCTTCTGGAGTCCCTTCTCGCTTCAGTGGCAGTGGGTCTGGGACCTTTTATTCTCTCACAATCAGCAGTGTGGAGGCTGAAGATGCTGCCGATTATTACTGCCATCAGTGGAGTAGTTATCCA
IGKV4-80*01_DBA1	DBA/1J	IGKV	MUSMUS IGKV4-80*01 F	100	CAAATTGTTCTCACCCAGTCTCCAGCAATCATGTCTGCATCTCTAGGGGAGGAGATCACCCTAACCTGCAGTGCCAGCTCGAGTGTAAGTTACATGCACTGGTACCAGCAGAAGTCAGGCACTTCTCCCAAACTCTTGATTTATAGCACATCCAACCTGGCTTCTGGAGTCCCTTCTCGCTTCAGTGGCAGTGGGTCTGGGACCTTTTATTCTCTCACAATCAGCAGTGTGGAGGCTGAAGATGCTGCCGATTATTACTGCCATCAGTGGAGTAGTTATCCA

IGKV4-80*01_DBA2	DBA/2J	IGKV	MUSMUS IGKV4-80*01 F	100	CAAATTGTTCTCACCCAGTCTCCAGCAATCATGTCTGCATCTCTAGGGGAGGAGATCACCCTAACCTGCAGTGCCAGCTCGAGTGTAAGTTACATGCACTGGTACCAGCAGAAGTCAGGCACTTCTCCCAAACTCTTGATTTATAGCACATCCAACCTGGCTTCTGGAGTCCCTTCTCGCTTCAGTGGCAGTGGGTCTGGGACCTTTTATTCTCTCACAATCAGCAGTGTGGAGGCTGAAGATGCTGCCGATTATTACTGCCATCAGTGGAGTAGTTATCCA
IGKV4-80*01_LEWES	LEWES/EIJ	IGKV	MUSMUS IGKV4-80*01 F	100	CAAATTGTTCTCACCCAGTCTCCAGCAATCATGTCTGCATCTCTAGGGGAGGAGATCACCCTAACCTGCAGTGCCAGCTCGAGTGTAAGTTACATGCACTGGTACCAGCAGAAGTCAGGCACTTCTCCCAAACTCTTGATTTATAGCACATCCAACCTGGCTTCTGGAGTCCCTTCTCGCTTCAGTGGCAGTGGGTCTGGGACCTTTTATTCTCTCACAATCAGCAGTGTGGAGGCTGAAGATGCTGCCGATTATTACTGCCATCAGTGGAGTAGTTATCCA
IGKV4-80*01_NZB	NZB/BLNJ	IGKV	MUSMUS IGKV4-80*01 F	100	CAAATTGTTCTCACCCAGTCTCCAGCAATCATGTCTGCATCTCTAGGGGAGGAGATCACCCTAACCTGCAGTGCCAGCTCGAGTGTAAGTTACATGCACTGGTACCAGCAGAAGTCAGGCACTTCTCCCAAACTCTTGATTTATAGCACATCCAACCTGGCTTCTGGAGTCCCTTCTCGCTTCAGTGGCAGTGGGTCTGGGACCTTTTATTCTCTCACAATCAGCAGTGTGGAGGCTGAAGATGCTGCCGATTATTACTGCCATCAGTGGAGTAGTTATCCA
IGKV4-80*01_S4247_AKR	AKR/J	IGKV	MUSMUS IGKV4-80*01 F	99.64	CAAATTGTTCTCACCCAGTCTCCAGCAATCATGTCTGCATCTCTAGGGGAGGAGATCACCCTAACCTGCAGTGCCAGCTCGAGTGTAAGTTACATGCACTGGTTCCAGCAGAAGTCAGGCACTTCTCCCAAACTCTTGATTTATAGCACATCCAACCTGGCTTCTGGAGTCCCTTCTCGCTTCAGTGGCAGTGGGTCTGGGACCTTTTATTCTCTCACAATCAGCAGTGTGGAGGCTGAAGATGCTGCCGATTATTACTGCCATCAGTGGAGTAGTTATCCA

IGKV4-80*01_S4247_CAST	CAST/EIJ	IGKV	MUSMUS IGKV4-80*01 F	99.64	CAAATTGTTCTCACCCAGTCTCCAGCAATCATGTCTGCATCTCTAGGGGAGGAGATCACCCTAACCTGCAGTGCCAGCTCGAGTGTAAGTTACATGCACTGGTTCCAGCAGAAGTCAGGCACTTCTCCCAAACTCTTGATTTATAGCACATCCAACCTGGCTTCTGGAGTCCCTTCTCGCTTCAGTGGCAGTGGGTCTGGGACCTTTTATTCTCTCACAATCAGCAGTGTGGAGGCTGAAGATGCTGCCGATTATTACTGCCATCAGTGGAGTAGTTATCCA
IGKV4-80*01_S4247_MRL	MRL/MPJ	IGKV	MUSMUS IGKV4-80*01 F	99.64	CAAATTGTTCTCACCCAGTCTCCAGCAATCATGTCTGCATCTCTAGGGGAGGAGATCACCCTAACCTGCAGTGCCAGCTCGAGTGTAAGTTACATGCACTGGTTCCAGCAGAAGTCAGGCACTTCTCCCAAACTCTTGATTTATAGCACATCCAACCTGGCTTCTGGAGTCCCTTCTCGCTTCAGTGGCAGTGGGTCTGGGACCTTTTATTCTCTCACAATCAGCAGTGTGGAGGCTGAAGATGCTGCCGATTATTACTGCCATCAGTGGAGTAGTTATCCA
IGKV4-80*01_S4247_MSM	MSM/MSJ	IGKV	MUSMUS IGKV4-80*01 F	99.64	CAAATTGTTCTCACCCAGTCTCCAGCAATCATGTCTGCATCTCTAGGGGAGGAGATCACCCTAACCTGCAGTGCCAGCTCGAGTGTAAGTTACATGCACTGGTTCCAGCAGAAGTCAGGCACTTCTCCCAAACTCTTGATTTATAGCACATCCAACCTGGCTTCTGGAGTCCCTTCTCGCTTCAGTGGCAGTGGGTCTGGGACCTTTTATTCTCTCACAATCAGCAGTGTGGAGGCTGAAGATGCTGCCGATTATTACTGCCATCAGTGGAGTAGTTATCCA
IGKV4-80*01_S4247_NOR	NOR/LTJ	IGKV	MUSMUS IGKV4-80*01 F	99.64	CAAATTGTTCTCACCCAGTCTCCAGCAATCATGTCTGCATCTCTAGGGGAGGAGATCACCCTAACCTGCAGTGCCAGCTCGAGTGTAAGTTACATGCACTGGTTCCAGCAGAAGTCAGGCACTTCTCCCAAACTCTTGATTTATAGCACATCCAACCTGGCTTCTGGAGTCCCTTCTCGCTTCAGTGGCAGTGGGTCTGGGACCTTTTATTCTCTCACAATCAGCAGTGTGGAGGCTGAAGATGCTGCCGATTATTACTGCCATCAGTGGAGTAGTTATCCA

IGKV4-80*01_S5342_NOD	NOD/SHILTJ	IGKV	MUSMUS IGKV4-80*01 F	99.64	CAAATTGTTCTCACCCAGTCTCCAGCAATCATGTCTGCATCTCTAGGGGAGGAGATCACCCTAACCTGCAGTGCCAGCTCGAGTGTAAGTTACATGCACTGGTTCCAGCAGAAGTCAGGCACTTCTCCCAAACTCTTGATTTATAGCACATCCAACCTGGCTTCTGGAGTCCCTTCTCGCTTCAGTGGCAGTGGGTCTGGGACCTTTTATTCTCTCACAATCAGCAGTGTGGAGGCTGAAGATGCTGCCGATTATTACTGCCATCAGTGGAGTAGTTATCC
IGKV4-80*01_S9832_PWD	PWD/PHJ	IGKV	MUSMUS IGKV4-80*01 F	99.28	CAAATTGTTCTCACCCAGTCTCCAGCAATCATGTCTGCATCTCTAGGGGAGGAGATCACCCTAACTTGCAGTGCCAGCTCGAGTGTAAGTTACATGCACTGGTTCCAGCAGAAGTCAGGCACTTCTCCCAAACTCTTGATTTATAGCACATCCAACCTGGCTTCTGGAGTCCCTTCTCGCTTCAGTGGCAGTGGGTCTGGGACCTTTTATTCTCTCACAATCAGCAGTGTGGAGGCTGAAGATGCTGCCGATTATTACTGCCATCAGTGGAGTAGTTATCCA
IGKV4-80*01_SJL	SJL/J	IGKV	MUSMUS IGKV4-80*01 F	100	CAAATTGTTCTCACCCAGTCTCCAGCAATCATGTCTGCATCTCTAGGGGAGGAGATCACCCTAACCTGCAGTGCCAGCTCGAGTGTAAGTTACATGCACTGGTACCAGCAGAAGTCAGGCACTTCTCCCAAACTCTTGATTTATAGCACATCCAACCTGGCTTCTGGAGTCCCTTCTCGCTTCAGTGGCAGTGGGTCTGGGACCTTTTATTCTCTCACAATCAGCAGTGTGGAGGCTGAAGATGCTGCCGATTATTACTGCCATCAGTGGAGTAGTTATCCA
IGKV4-81*01_AJ	A/J	IGKV	MUSMUS IGKV4-81*01 F	100	GAAAATGTGCTGACCCAGTCTCCAGCAATCATGGCTGCATCTCCAGGGGAGAAGGTCACCATGACCTGCAGTGCCAGCTCAAGTGTAAGTTCTAGTAACTTGCACTGGTACCAGCAGAAGTCAGGCACTTCTACCAAATTCTGGATTTATAGGACATCCAACCTGGCTTCAGAAGTCCCAGCTCCCTTCAGTGGCAGTGGGTCTGGGACCTCTTACTCTCTTACAATCAGCAGCGTGGAGGCCGAAGATGCTGCCACTTATTACTGCCAGCAGTGGAGTGGTTACCCACCCA

IGKV4-81*01_B6	C57BL/6J	IGKV	MUSMUS IGKV4-81*01 F	100	GAAAATGTGCTGACCCAGTCTC<br>CAGCAATCATGGCTGCATCTCC<br>AGGGGAGAAGGTCACCATGAC<br>CTGCAGTGCCAGCTCAAGTGTA<br>AGTTCTAGTAACTTGCACTGGTA<br>CCAGCAGAAGTCAGGCACTTCT<br>ACCAAATTCTGGATTTATAGGAC<br>ATCCAACCTGGCTTCAGAAGTC<br>CCAGCTCCCTTCAGTGGCAGTG<br>GGTCTGGGACCTCTTACTCTCTT<br>ACAATCAGCAGCGTGGAGGCC<br>GAAGATGCTGCCACTTATTACTG<br>CCAGCAGTGGAGTGGTTACCCA<br>CCCA
IGKV4-81*01_C3H	C3H/HEJ	IGKV	MUSMUS IGKV4-81*01 F	100	GAAAATGTGCTGACCCAGTCTC<br>CAGCAATCATGGCTGCATCTCC<br>AGGGGAGAAGGTCACCATGAC<br>CTGCAGTGCCAGCTCAAGTGTA<br>AGTTCTAGTAACTTGCACTGGTA<br>CCAGCAGAAGTCAGGCACTTCT<br>ACCAAATTCTGGATTTATAGGAC<br>ATCCAACCTGGCTTCAGAAGTC<br>CCAGCTCCCTTCAGTGGCAGTG<br>GGTCTGGGACCTCTTACTCTCTT<br>ACAATCAGCAGCGTGGAGGCC<br>GAAGATGCTGCCACTTATTACTG<br>CCAGCAGTGGAGTGGTTACCCA<br>CCCA
IGKV4-81*01_DBA2	DBA/2J	IGKV	MUSMUS IGKV4-81*01 F	100	GAAAATGTGCTGACCCAGTCTC<br>CAGCAATCATGGCTGCATCTCC<br>AGGGGAGAAGGTCACCATGAC<br>CTGCAGTGCCAGCTCAAGTGTA<br>AGTTCTAGTAACTTGCACTGGTA<br>CCAGCAGAAGTCAGGCACTTCT<br>ACCAAATTCTGGATTTATAGGAC<br>ATCCAACCTGGCTTCAGAAGTC<br>CCAGCTCCCTTCAGTGGCAGTG<br>GGTCTGGGACCTCTTACTCTCTT<br>ACAATCAGCAGCGTGGAGGCC<br>GAAGATGCTGCCACTTATTACTG<br>CCAGCAGTGGAGTGGTTACCCA<br>CCCA
IGKV4-81*01_NZB	NZB/BLNJ	IGKV	MUSMUS IGKV4-81*01 F	100	GAAAATGTGCTGACCCAGTCTC<br>CAGCAATCATGGCTGCATCTCC<br>AGGGGAGAAGGTCACCATGAC<br>CTGCAGTGCCAGCTCAAGTGTA<br>AGTTCTAGTAACTTGCACTGGTA<br>CCAGCAGAAGTCAGGCACTTCT<br>ACCAAATTCTGGATTTATAGGAC<br>ATCCAACCTGGCTTCAGAAGTC<br>CCAGCTCCCTTCAGTGGCAGTG<br>GGTCTGGGACCTCTTACTCTCTT<br>ACAATCAGCAGCGTGGAGGCC<br>GAAGATGCTGCCACTTATTACTG<br>CCAGCAGTGGAGTGGTTACCCA<br>CCCA

IGKV4-81*01_S0130_AJ	A/J	IGKV	MUSMUS IGKV4-81*01 F	100	GAAAATGTGCTGACCCAGTCTC<br>CAGCAATCATGGCTGCATCTCC<br>AGGGGAGAAGGTCACCATGAC<br>CTGCAGTGCCAGCTCAAGTGTA<br>AGTTCTAGTAACTTGCACTGGTA<br>CCAGCAGAAGTCAGGCACTTCT<br>ACCAAATTCTGGATTTATAGGAC<br>ATCCAACCTGGCTTCAGAAGTC<br>CCAGCTCCCTTCAGTGGCAGTG<br>GGTCTGGGACCTCTTACTCTCTT<br>ACAATCAGCAGCGTGGAGGCC<br>GAAGATGCTGCCACTTATTACTG<br>CCAGCAGTGGAGTGGTTACCCA<br>CTCA
IGKV4-81*01_S0130_B6	C57BL/6J	IGKV	MUSMUS IGKV4-81*01 F	100	GAAAATGTGCTGACCCAGTCTC<br>CAGCAATCATGGCTGCATCTCC<br>AGGGGAGAAGGTCACCATGAC<br>CTGCAGTGCCAGCTCAAGTGTA<br>AGTTCTAGTAACTTGCACTGGTA<br>CCAGCAGAAGTCAGGCACTTCT<br>ACCAAATTCTGGATTTATAGGAC<br>ATCCAACCTGGCTTCAGAAGTC<br>CCAGCTCCCTTCAGTGGCAGTG<br>GGTCTGGGACCTCTTACTCTCTT<br>ACAATCAGCAGCGTGGAGGCC<br>GAAGATGCTGCCACTTATTACTG<br>CCAGCAGTGGAGTGGTTACCCA<br>CTCA
IGKV4-81*01_S0130_BALB	BALB/CBYJ	IGKV	MUSMUS IGKV4-81*01 F	100	GAAAATGTGCTGACCCAGTCTC<br>CAGCAATCATGGCTGCATCTCC<br>AGGGGAGAAGGTCACCATGAC<br>CTGCAGTGCCAGCTCAAGTGTA<br>AGTTCTAGTAACTTGCACTGGTA<br>CCAGCAGAAGTCAGGCACTTCT<br>ACCAAATTCTGGATTTATAGGAC<br>ATCCAACCTGGCTTCAGAAGTC<br>CCAGCTCCCTTCAGTGGCAGTG<br>GGTCTGGGACCTCTTACTCTCTT<br>ACAATCAGCAGCGTGGAGGCC<br>GAAGATGCTGCCACTTATTACTG<br>CCAGCAGTGGAGTGGTTACCCA<br>CTCA
IGKV4-81*01_S1013_PWD	PWD/PHJ	IGKV	MUSMUS IGKV4-81*01 F	96.1	GAAAATGTGCTGACCCAGTATC<br>CAGCAATCATGGCTGCATCTCC<br>AGGGGAGAAGGTCACCATGAC<br>CTGCAGTGCCAGCTCAAGTGTA<br>AGTTCTGATAACTTTCACTGGTA<br>CCAGCAGAAGTCAGGCACTTCT<br>CCCAAACTCTGGATTTATAGGAC<br>ATCCAAATTGGCTTCTGGAGTCC<br>CAGCTCGCTTCAGTGGCAGTGG<br>GTCTGGGACCTCTTACTCTCTTA<br>CAATCAGCAGCGTGGAGGCCG<br>AAGATGCTGCCACTTATTACTGC<br>CAGCAGTGGAGTGGTTACCCAC<br>TCA

IGKV4-81*01_S2422_AKR	AKR/J	IGKV	MUSMUS IGKV4-81*01 F	96.45	GAAAATGTGCTGACCCAGTCTCCAGCAATCATGGCTGCATCTCCAGGGGAGAAGGTCACCATGACCTGCAGTGCCAGCTCAAGTGTAAGTTCTGGTAACTTTCACTGGTACCAGCAGAAGCCAGGCACTTCTCCCAAACTCTGGATTTATAGGACATCCAACCTGGCTTCTGGAGTCCCCGCTCGCTTCAGTGGCAGTGGGTCTGGGACCTCTTACTCTCTTACAATCAGCAGCATGGAGGCCGAAGATGCTGCCACTTATTACTGCCAGCAGTGGAGTGGTTACCCACCCA
IGKV4-81*01_S2422_NOD	NOD/SHILTJ	IGKV	MUSMUS IGKV4-81*01 F	96.45	GAAAATGTGCTGACCCAGTCTCCAGCAATCATGGCTGCATCTCCAGGGGAGAAGGTCACCATGACCTGCAGTGCCAGCTCAAGTGTAAGTTCTGGTAACTTTCACTGGTACCAGCAGAAGCCAGGCACTTCTCCCAAACTCTGGATTTATAGGACATCCAACCTGGCTTCTGGAGTCCCCGCTCGCTTCAGTGGCAGTGGGTCTGGGACCTCTTACTCTCTTACAATCAGCAGCATGGAGGCCGAAGATGCTGCCACTTATTACTGCCAGCAGTGGAGTGGTTACCCACCCA
IGKV4-81*01_S2422_NOR	NOR/LTJ	IGKV	MUSMUS IGKV4-81*01 F	96.45	GAAAATGTGCTGACCCAGTCTCCAGCAATCATGGCTGCATCTCCAGGGGAGAAGGTCACCATGACCTGCAGTGCCAGCTCAAGTGTAAGTTCTGGTAACTTTCACTGGTACCAGCAGAAGCCAGGCACTTCTCCCAAACTCTGGATTTATAGGACATCCAACCTGGCTTCTGGAGTCCCCGCTCGCTTCAGTGGCAGTGGGTCTGGGACCTCTTACTCTCTTACAATCAGCAGCATGGAGGCCGAAGATGCTGCCACTTATTACTGCCAGCAGTGGAGTGGTTACCCACCCA
IGKV4-81*01_S3230_PWD	PWD/PHJ	IGKV	MUSMUS IGKV4-81*01 F	96.1	GAAAATGTGCTGACCCAGTATCCAGCAATCATGGCTGCATCTCCAGGGGAGAAGGTCACCATGACCTGCAGTGCCAGCTCAAGTGTAAGTTCTGATAACTTTCACTGGTACCAGCAGAAGTCAGGCACTTCTCCCAAACTCTGGATTTATAGGACATCCAAATTGGCTTCTGGAGTCCCAGCTCGCTTCAGTGGCAGTGGGTCTGGGACCTCTTACTCTCTTACAATCAGCAGCGTGGAGGCCGAAGATGCTGCCACTTATTACTGCCAGCAGTGGAGTGGTTACCCACCCA

IGKV4-81*01_S7273_MRL	MRL/MPJ	IGKV	MUSMUS IGKV4-81*01 F	96.45	GAAAATGTGCTGACCCAGTCTCCAGCAATCATGGCTGCATCTCCAGGGGAGAAGGTCACCATGACCTGCAGTGCCAGCTCAAGTGTAAGTTCTGGTAACTTTCACTGGTACCAGCAGAAGCCAGGCACTTCTCCCAAACTCTGGATTTATAGGACATCCAACCTGGCTTCTGGAGTCCCCGCTCGCTTCAGTGGCAGTGGGTCTGGGACCTCTTACTCTCTTACAATCAGCAGCATGGAGGCCGAAGATGCTGCCACTTATTACTGCCAGCAGTGGAGTGGTTACCCACC
IGKV4-81*01_S7470_MSM	MSM/MSJ	IGKV	MUSMUS IGKV4-81*01 F	96.81	GAAAATGTGCTGACCCAGTCTCCAGCAATCATGGCTGCATCTCCAGGGGAGAAGGTCACCATGACCTGCAGTGCCAGCTCAAGTGTAAGTTCTGGTAACTTTCACTGGTACCAGCAGAAGCCAGGCACTTCTCCCAAACTCTGGATTTATAGGACATCCAACCTGGCTTCTGGAGTCCCAGCTCGCTTCAGTGGCAGTGGGTCTGGGACCTCTTACTCTCTTACAATCAGCAGCATGGAGGCCGAAGATGCTGCCACTTATTACTGCCAGCAGTGGAGTGGTTACCCACCCA
IGKV4-81*01_S7470_PWD	PWD/PHJ	IGKV	MUSMUS IGKV4-81*01 F	96.81	GAAAATGTGCTGACCCAGTCTCCAGCAATCATGGCTGCATCTCCAGGGGAGAAGGTCACCATGACCTGCAGTGCCAGCTCAAGTGTAAGTTCTGGTAACTTTCACTGGTACCAGCAGAAGCCAGGCACTTCTCCCAAACTCTGGATTTATAGGACATCCAACCTGGCTTCTGGAGTCCCAGCTCGCTTCAGTGGCAGTGGGTCTGGGACCTCTTACTCTCTTACAATCAGCAGCATGGAGGCCGAAGATGCTGCCACTTATTACTGCCAGCAGTGGAGTGGTTACCCACCCA
IGKV4-81*01_S8073_CAST	CAST/EIJ	IGKV	MUSMUS IGKV4-81*01 F	95.39	GAAAATGTGCTGACCCAGTCTCCAGCAATCATGGCTGCATCTCCAGGGGAGAAGGTCACCATGACCTGCAGTGCCAGCTCAAGTGTAAGTTCTGGTAACTTTCACTGGTACCAGCAGAAGCCAGGCACTTCTCACAAACTCTGGATTTATAGGACATCCAACCTGGCTTCTGGAGTCCCAGCTCGCTTCAGTGGCAGTGGGTCTGGGACCTCTTATTCTCTTACAATCAACAGCATGGAAGCCGAAGATGCTGCCACTTATTACTGCCAGCAGTGGAGTGGTTACCCACC

IGKV4-81*01_S9153_MRL	MRL/MPJ	IGKV	MUSMUS IGKV4-81*01 F	96.45	GAAAATGTGCTGACCCAGTCTCCAGCAATCATGGCTGCATCTCCAGGGGAGAAGGTCACCATGACCTGCAGTGCCAGCTCAAGTGTAAGTTCTGGTAACTTTCACTGGTACCAGCAGAAGCCAGGCACTTCTCCCAAACTCTGGATTTATAGGACATCCAACCTGGCTTCTGGAGTCCCCGCTCGCTTCAGTGGCAGTGGGTCTGGGACCTCTTACTCTCTTACAATCAGCAGCATGGAGGCCGAAGATGCTGCCACTTATTACTGCCAGCAGTGGAGTGGTTACCCACTCA
IGKV4-81*01_S9628_MSM	MSM/MSJ	IGKV	MUSMUS IGKV4-81*01 F	96.45	GAAAATGTGCTGACCCAGTCTCCAGCAATCATGGCTGCATCTCCAGGGGAGAAGGTCACCATGACCTGCAGTGCCAGCTCAAGTGTAAGTTCTGATAACTTTCACTGGTACCAGCAGAAGTCAGGCACTTCTCCCAAACTCTGGATTTATAGGACATCCAAATTGGCTTCTGGAGTCCCAGCTCGCTTCAGTGGCAGTGGGTCTGGGACCTCTTACTCTCTTACAATCAGCAGCGTGGAGGCCGAAGATGCTGCCACTTATTACTGCCAGCAGTGGAGTGGTTACCCGCTCA
IGKV4-86*01_129	129S1/SVIMJ	IGKV	MUSMUS IGKV4-86*01 F	100	GAAATTGTGCTCACTCAGTCTCCAGCCATCACAGCTGCATCTCTGGGGCAAAAGGTCACCATCACCTGCAGTGCCAGCTCAAGTGTAAGTTACATGCACTGGTACCAGCAGAAGTCAGGCACCTCCCCCAAACCATGGATTTATGAAATATCCAAACTGGCTTCTGGAGTCCCAGCTCGCTTCAGTGGCAGTGGGTCTGGGACCTCTTACTCTCTCACAATCAGCAGCATGGAGGCTGAAGATGCTGCCATTTATTACTGCCAGCAGTGGAATTATCCTCTTA
IGKV4-86*01_AJ	A/J	IGKV	MUSMUS IGKV4-86*01 F	100	GAAATTGTGCTCACTCAGTCTCCAGCCATCACAGCTGCATCTCTGGGGCAAAAGGTCACCATCACCTGCAGTGCCAGCTCAAGTGTAAGTTACATGCACTGGTACCAGCAGAAGTCAGGCACCTCCCCCAAACCATGGATTTATGAAATATCCAAACTGGCTTCTGGAGTCCCAGCTCGCTTCAGTGGCAGTGGGTCTGGGACCTCTTACTCTCTCACAATCAGCAGCATGGAGGCTGAAGATGCTGCCATTTATTACTGCCAGCAGTGGAATTATCCTCTTA

IGKV4-86*01_B6	C57BL/6J	IGKV	MUSMUS IGKV4-86*01 F	100	GAAATTGTGCTCACTCAGTCTCCAGCCATCACAGCTGCATCTCTGGGGCAAAAGGTCACCATCACCTGCAGTGCCAGCTCAAGTGTAAGTTACATGCACTGGTACCAGCAGAAGTCAGGCACCTCCCCCAAACCATGGATTTATGAAATATCCAAACTGGCTTCTGGAGTCCCAGCTCGCTTCAGTGGCAGTGGGTCTGGGACCTCTTACTCTCTCACAATCAGCAGCATGGAGGCTGAAGATGCTGCCATTTATTACTGCCAGCAGTGGAATTATCCTCTTA
IGKV4-86*01_BALB	BALB/CBYJ	IGKV	MUSMUS IGKV4-86*01 F	100	GAAATTGTGCTCACTCAGTCTCCAGCCATCACAGCTGCATCTCTGGGGCAAAAGGTCACCATCACCTGCAGTGCCAGCTCAAGTGTAAGTTACATGCACTGGTACCAGCAGAAGTCAGGCACCTCCCCCAAACCATGGATTTATGAAATATCCAAACTGGCTTCTGGAGTCCCAGCTCGCTTCAGTGGCAGTGGGTCTGGGACCTCTTACTCTCTCACAATCAGCAGCATGGAGGCTGAAGATGCTGCCATTTATTACTGCCAGCAGTGGAATTATCCTCTTA
IGKV4-86*01_C3H	C3H/HEJ	IGKV	MUSMUS IGKV4-86*01 F	100	GAAATTGTGCTCACTCAGTCTCCAGCCATCACAGCTGCATCTCTGGGGCAAAAGGTCACCATCACCTGCAGTGCCAGCTCAAGTGTAAGTTACATGCACTGGTACCAGCAGAAGTCAGGCACCTCCCCCAAACCATGGATTTATGAAATATCCAAACTGGCTTCTGGAGTCCCAGCTCGCTTCAGTGGCAGTGGGTCTGGGACCTCTTACTCTCTCACAATCAGCAGCATGGAGGCTGAAGATGCTGCCATTTATTACTGCCAGCAGTGGAATTATCCTCTTA
IGKV4-86*01_CBA	CBA/J	IGKV	MUSMUS IGKV4-86*01 F	100	GAAATTGTGCTCACTCAGTCTCCAGCCATCACAGCTGCATCTCTGGGGCAAAAGGTCACCATCACCTGCAGTGCCAGCTCAAGTGTAAGTTACATGCACTGGTACCAGCAGAAGTCAGGCACCTCCCCCAAACCATGGATTTATGAAATATCCAAACTGGCTTCTGGAGTCCCAGCTCGCTTCAGTGGCAGTGGGTCTGGGACCTCTTACTCTCTCACAATCAGCAGCATGGAGGCTGAAGATGCTGCCATTTATTACTGCCAGCAGTGGAATTATCCTCTTA

IGKV4-86*01_DBA1	DBA/1J	IGKV	MUSMUS IGKV4-86*01 F	100	GAAATTGTGCTCACTCAGTCTCC<br>AGCCATCACAGCTGCATCTCTG<br>GGGCAAAAGGTCACCATCACCT<br>GCAGTGCCAGCTCAAGTGTAAG<br>TTACATGCACTGGTACCAGCAG<br>AAGTCAGGCACCTCCCCCAAA<br>CCATGGATTTATGAAATATCCAA<br>ACTGGCTTCTGGAGTCCCAGCT<br>CGCTTCAGTGGCAGTGGGTCTG<br>GGACCTCTTACTCTCTCACAATC<br>AGCAGCATGGAGGCTGAAGAT<br>GCTGCCATTTATTACTGCCAGCA<br>GTGGAATTATCCTCTTA
IGKV4-86*01_DBA2	DBA/2J	IGKV	MUSMUS IGKV4-86*01 F	100	GAAATTGTGCTCACTCAGTCTCC<br>AGCCATCACAGCTGCATCTCTG<br>GGGCAAAAGGTCACCATCACCT<br>GCAGTGCCAGCTCAAGTGTAAG<br>TTACATGCACTGGTACCAGCAG<br>AAGTCAGGCACCTCCCCCAAA<br>CCATGGATTTATGAAATATCCAA<br>ACTGGCTTCTGGAGTCCCAGCT<br>CGCTTCAGTGGCAGTGGGTCTG<br>GGACCTCTTACTCTCTCACAATC<br>AGCAGCATGGAGGCTGAAGAT<br>GCTGCCATTTATTACTGCCAGCA<br>GTGGAATTATCCTCTTA
IGKV4-86*01_LEWES	LEWES/EIJ	IGKV	MUSMUS IGKV4-86*01 F	100	GAAATTGTGCTCACTCAGTCTCC<br>AGCCATCACAGCTGCATCTCTG<br>GGGCAAAAGGTCACCATCACCT<br>GCAGTGCCAGCTCAAGTGTAAG<br>TTACATGCACTGGTACCAGCAG<br>AAGTCAGGCACCTCCCCCAAA<br>CCATGGATTTATGAAATATCCAA<br>ACTGGCTTCTGGAGTCCCAGCT<br>CGCTTCAGTGGCAGTGGGTCTG<br>GGACCTCTTACTCTCTCACAATC<br>AGCAGCATGGAGGCTGAAGAT<br>GCTGCCATTTATTACTGCCAGCA<br>GTGGAATTATCCTCTTA
IGKV4-86*01_NZB	NZB/BLNJ	IGKV	MUSMUS IGKV4-86*01 F	100	GAAATTGTGCTCACTCAGTCTCC<br>AGCCATCACAGCTGCATCTCTG<br>GGGCAAAAGGTCACCATCACCT<br>GCAGTGCCAGCTCAAGTGTAAG<br>TTACATGCACTGGTACCAGCAG<br>AAGTCAGGCACCTCCCCCAAA<br>CCATGGATTTATGAAATATCCAA<br>ACTGGCTTCTGGAGTCCCAGCT<br>CGCTTCAGTGGCAGTGGGTCTG<br>GGACCTCTTACTCTCTCACAATC<br>AGCAGCATGGAGGCTGAAGAT<br>GCTGCCATTTATTACTGCCAGCA<br>GTGGAATTATCCTCTTA

IGKV4-86*01_S4316_AKR	AKR/J	IGKV	MUSMUS IGKV4-86*01 F	100	GAAATTGTGCTCACTCAGTCTCCAGCCATCACAGCTGCATCTCTGGGGCAAAAGGTCACCATCACCTGCAGTGCCAGCTCAAGTGTAAGTTACATGCACTGGTACCAGCAGAAGTCAGGCACCTCCCCCAAACCATGGATTTATGAAATATCCAAACTGGCTTCTGGAGTCCCAGCTCGCTTCAGTGGCAGTGGGTCTGGGACCTCTTACTCTCTCACAATCAGCAGCATGGAGGCTGAAGATGCTGCCATTTATTACTGCCAGCAGTGGAATTATCCACTTA
IGKV4-86*01_S4316_CAST	CAST/EIJ	IGKV	MUSMUS IGKV4-86*01 F	100	GAAATTGTGCTCACTCAGTCTCCAGCCATCACAGCTGCATCTCTGGGGCAAAAGGTCACCATCACCTGCAGTGCCAGCTCAAGTGTAAGTTACATGCACTGGTACCAGCAGAAGTCAGGCACCTCCCCCAAACCATGGATTTATGAAATATCCAAACTGGCTTCTGGAGTCCCAGCTCGCTTCAGTGGCAGTGGGTCTGGGACCTCTTACTCTCTCACAATCAGCAGCATGGAGGCTGAAGATGCTGCCATTTATTACTGCCAGCAGTGGAATTATCCACTTA
IGKV4-86*01_S4316_MRL	MRL/MPJ	IGKV	MUSMUS IGKV4-86*01 F	100	GAAATTGTGCTCACTCAGTCTCCAGCCATCACAGCTGCATCTCTGGGGCAAAAGGTCACCATCACCTGCAGTGCCAGCTCAAGTGTAAGTTACATGCACTGGTACCAGCAGAAGTCAGGCACCTCCCCCAAACCATGGATTTATGAAATATCCAAACTGGCTTCTGGAGTCCCAGCTCGCTTCAGTGGCAGTGGGTCTGGGACCTCTTACTCTCTCACAATCAGCAGCATGGAGGCTGAAGATGCTGCCATTTATTACTGCCAGCAGTGGAATTATCCACTTA
IGKV4-86*01_S4316_NOR	NOR/LTJ	IGKV	MUSMUS IGKV4-86*01 F	100	GAAATTGTGCTCACTCAGTCTCCAGCCATCACAGCTGCATCTCTGGGGCAAAAGGTCACCATCACCTGCAGTGCCAGCTCAAGTGTAAGTTACATGCACTGGTACCAGCAGAAGTCAGGCACCTCCCCCAAACCATGGATTTATGAAATATCCAAACTGGCTTCTGGAGTCCCAGCTCGCTTCAGTGGCAGTGGGTCTGGGACCTCTTACTCTCTCACAATCAGCAGCATGGAGGCTGAAGATGCTGCCATTTATTACTGCCAGCAGTGGAATTATCCACTTA

IGKV4-86*01_S6393_MSM	MSM/MSJ	IGKV	MUSMUS IGKV4-86*01 F	94.93	GAAATTGTGCTCACTCAGTCTCCAGCCATCATAGCTGCATCTCTGGGGCAAAAGGTCACCATGACCTGCAGTGCCAGCTCAAGTGTAAGTTACATGCACTGGTACCAGCAGAAGTCAGGCACCTCCCCCAAACTCTGGATTTACGGCACATCCAACCTGGCTTCTGGAGTCCCAGCTCGCTTCAGTGGCAGTGGGTCTGGGACCTCTTACTCTCTCACAATCAGCAGCGTGGAGGCTGAAGATGATGCAACTTATTACTGCCAGCAGTGGAATTACCCACT
IGKV4-86*01_S6393_PWD	PWD/PHJ	IGKV	MUSMUS IGKV4-86*01 F	94.93	GAAATTGTGCTCACTCAGTCTCCAGCCATCATAGCTGCATCTCTGGGGCAAAAGGTCACCATGACCTGCAGTGCCAGCTCAAGTGTAAGTTACATGCACTGGTACCAGCAGAAGTCAGGCACCTCCCCCAAACTCTGGATTTACGGCACATCCAACCTGGCTTCTGGAGTCCCAGCTCGCTTCAGTGGCAGTGGGTCTGGGACCTCTTACTCTCTCACAATCAGCAGCGTGGAGGCTGAAGATGATGCAACTTATTACTGCCAGCAGTGGAATTACCCACT
IGKV4-90*01_AJ	A/J	IGKV	MUSMUS IGKV4-90*01 F	100	GAAATTTTGCTCACCCAGTCTCCAGCAATCATAGCTGCATCTCCTGGGGAGAAGGTCACCATCACCTGCAGTGCCAGCTCAAGTGTAAGTTACATGAACTGGTACCAGCAGAAACCAGGATCCTCCCCCAAAATATGGATTTATGGTATATCCAACCTGGCTTCTGGAGTTCCTGCTCGCTTCAGTGGCAGTGGGTCTGGGACATCTTTCTCTTTCACAATCAACAGCATGGAGGCTGAAGATGTTGCCACTTATTACTGTCAGCAAAGGAGTAGTTACCCACCC
IGKV4-90*01_BALB	BALB/CBYJ	IGKV	MUSMUS IGKV4-90*01 F	100	GAAATTTTGCTCACCCAGTCTCCAGCAATCATAGCTGCATCTCCTGGGGAGAAGGTCACCATCACCTGCAGTGCCAGCTCAAGTGTAAGTTACATGAACTGGTACCAGCAGAAACCAGGATCCTCCCCCAAAATATGGATTTATGGTATATCCAACCTGGCTTCTGGAGTTCCTGCTCGCTTCAGTGGCAGTGGGTCTGGGACATCTTTCTCTTTCACAATCAACAGCATGGAGGCTGAAGATGTTGCCACTTATTACTGTCAGCAAAGGAGTAGTTACCCACCC

IGKV4-90*01_C3H	C3H/HEJ	IGKV	MUSMUS IGKV4-90*01 F	100	GAAATTTTGCTCACCCAGTCTCCAGCAATCATAGCTGCATCTCCTGGGGAGAAGGTCACCATCACCTGCAGTGCCAGCTCAAGTGTAAGTTACATGAACTGGTACCAGCAGAAACCAGGATCCTCCCCCAAAATATGGATTTATGGTATATCCAACCTGGCTTCTGGAGTTCCTGCTCGCTTCAGTGGCAGTGGGTCTGGGACATCTTTCTCTTTCACAATCAACAGCATGGAGGCTGAAGATGTTGCCACTTATTACTGTCAGCAAAGGAGTAGTTACCCACCC
IGKV4-90*01_DBA1	DBA/1J	IGKV	MUSMUS IGKV4-90*01 F	100	GAAATTTTGCTCACCCAGTCTCCAGCAATCATAGCTGCATCTCCTGGGGAGAAGGTCACCATCACCTGCAGTGCCAGCTCAAGTGTAAGTTACATGAACTGGTACCAGCAGAAACCAGGATCCTCCCCCAAAATATGGATTTATGGTATATCCAACCTGGCTTCTGGAGTTCCTGCTCGCTTCAGTGGCAGTGGGTCTGGGACATCTTTCTCTTTCACAATCAACAGCATGGAGGCTGAAGATGTTGCCACTTATTACTGTCAGCAAAGGAGTAGTTACCCACCC
IGKV4-90*01_DBA2	DBA/2J	IGKV	MUSMUS IGKV4-90*01 F	100	GAAATTTTGCTCACCCAGTCTCCAGCAATCATAGCTGCATCTCCTGGGGAGAAGGTCACCATCACCTGCAGTGCCAGCTCAAGTGTAAGTTACATGAACTGGTACCAGCAGAAACCAGGATCCTCCCCCAAAATATGGATTTATGGTATATCCAACCTGGCTTCTGGAGTTCCTGCTCGCTTCAGTGGCAGTGGGTCTGGGACATCTTTCTCTTTCACAATCAACAGCATGGAGGCTGAAGATGTTGCCACTTATTACTGTCAGCAAAGGAGTAGTTACCCACCC
IGKV4-90*01_LEWES	LEWES/EIJ	IGKV	MUSMUS IGKV4-90*01 F	100	GAAATTTTGCTCACCCAGTCTCCAGCAATCATAGCTGCATCTCCTGGGGAGAAGGTCACCATCACCTGCAGTGCCAGCTCAAGTGTAAGTTACATGAACTGGTACCAGCAGAAACCAGGATCCTCCCCCAAAATATGGATTTATGGTATATCCAACCTGGCTTCTGGAGTTCCTGCTCGCTTCAGTGGCAGTGGGTCTGGGACATCTTTCTCTTTCACAATCAACAGCATGGAGGCTGAAGATGTTGCCACTTATTACTGTCAGCAAAGGAGTAGTTACCCACCC

IGKV4-90*01_NZB	NZB/BLNJ	IGKV	MUSMUS IGKV4-90*01 F	100	GAAATTTTGCTCACCCAGTCTCCAGCAATCATAGCTGCATCTCCTGGGGAGAAGGTCACCATCACCTGCAGTGCCAGCTCAAGTGTAAGTTACATGAACTGGTACCAGCAGAAACCAGGATCCTCCCCCAAAATATGGATTTATGGTATATCCAACCTGGCTTCTGGAGTTCCTGCTCGCTTCAGTGGCAGTGGGTCTGGGACATCTTTCTCTTTCACAATCAACAGCATGGAGGCTGAAGATGTTGCCACTTATTACTGTCAGCAAAGGAGTAGTTACCCACCC
IGKV4-90*01_S4752_PWD	PWD/PHJ	IGKV	MUSMUS IGKV4-90*01 F	99.64	GAAATTTTGCTCACCCAGTCTCCAGCAATCATAGCTGCATCTCCTGGGGAGAAGGTCACCATCACCTGCAGTGCCAGCTCAAGTGTAAGTTACATGAACTGGTACCAGCAGAAACCAGGATCCTCTCCCAAAATATGGATTTATGGTATATCCAACCTGGCTTCTGGAGTTCCTGCTCGCTTCAGTGGCAGTGGGTCTGGGACATCTTTCTCTTTCACAATCAACAGCATGGAGGCTGAAGATGTTGCCACTTATTACTGTCAGCAAAGGAGTAGTTACCCACCC
IGKV4-90*01_S5868_CAST	CAST/EIJ	IGKV	MUSMUS IGKV4-90*01 F	99.28	GAAATTTTGCTCACCCAGTCTCCAGCAATCATAGCTGCATCTCCTGGGGAGAAGGTCACCATCACCTGCAGTGCCAGCTCAAGTGTAAGTTACATGCACTGGTACCAGCAGAAACCAGGATCCTCTCCCAAAATATGGATTTATGGTATATCCAACCTGGCTTCTGGAGTTCCTGCTCGCTTCAGTGGCAGTGGGTCTGGGACATCTTTCTCTTTCACAATCAACAGCATGGAGGCTGAAGATGTTGCCACTTATTACTGTCAGCAAAGGAGTAGTTACCCACCC
IGKV4-91*01_DBA1	DBA/1J	IGKV	MUSMUS IGKV4-91*01 F	100	GAAATTGTGCTCACCCAGTCTCCAACCACCATGGCTGCATCTCCCGGGGAGAAGATCACTATCACCTGCAGTGCCAGCTCAAGTATAAGTTCCAATTACTTGCATTGGTATCAGCAGAAGCCAGGATTCTCCCCTAAACTCTTGATTTATAGGACATCCAATCTGGCTTCTGGAGTCCCAGCTCGCTTCAGTGGCAGTGGGTCTGGGACCTCTTACTCTCTCACAATTGGCACCATGGAGGCTGAAGATGTTGCCACTTACTACTGCCAGCAGGGTAGTAGTATACCACGCA

IGKV4-91*01_NZB	NZB/BLNJ	IGKV	MUSMUS IGKV4-91*01 F	100	GAAATTGTGCTCACCCAGTCTCCAACCACCATGGCTGCATCTCCCGGGGAGAAGATCACTATCACCTGCAGTGCCAGCTCAAGTATAAGTTCCAATTACTTGCATTGGTATCAGCAGAAGCCAGGATTCTCCCCTAAACTCTTGATTTATAGGACATCCAATCTGGCTTCTGGAGTCCCAGCTCGCTTCAGTGGCAGTGGGTCTGGGACCTCTTACTCTCTCACAATTGGCACCATGGAGGCTGAAGATGTTGCCACTTACTACTGCCAGCAGGGTAGTAGTATACCACGCA
IGKV4-91*01_S3435_CAST	CAST/EIJ	IGKV	MUSMUS IGKV4-91*01 F	96.45	GAAATTGTGCTCACCCAGTCTCCAACCATCATGGCTGCATCTCCCGGGGAGAAGGTCACCATCACCTGCAGTGCCAGCTCAAGTATAAGTTCCAATTACTTGCACTGGTATCAGCAGAAGCCAGGATTCCCTCCTAAACTCTTGATATATAGGACATCCAATCTGGCTTCTGGAGTCCCAGCTCGCTTCAGTGGCAGTGGGTCTGGGACCTCTTACTCTCTCACAATTGGCACCATGGAGGCTGAAGGTGCTGCCACTTATTACTGCCAGCAGGGTAGTAGTTTACCACTCA
IGKV4-91*01_S4567_MSM	MSM/MSJ	IGKV	MUSMUS IGKV4-91*01 F	96.81	GAAATTGTGCTCACCCAGTCTCCAACCACCATGGCTGCATCTCCCGGGGAGAAGGTCACCATCACCTGCAGTGCCAGCTCAAGTATAAGTTCCAATTACTTGCACTGGTATCAGCAGAAGCCAGGATTCCCTCCTAAACTCTTGATATATAGGACATCCAATCTGGCTTCTGGAGTCCCAGCTCGCTTCAGTGGCAGTGGGTCTGGGACCTCTTACTCTCTCACAATTGGCACCATGGAGGCTGAAGGTGCTGCCACTTATTACTGCCAGCAGGGTAGTAGTTTACCACACA
IGKV4-91*01_S4567_PWD	PWD/PHJ	IGKV	MUSMUS IGKV4-91*01 F	96.81	GAAATTGTGCTCACCCAGTCTCCAACCACCATGGCTGCATCTCCCGGGGAGAAGGTCACCATCACCTGCAGTGCCAGCTCAAGTATAAGTTCCAATTACTTGCACTGGTATCAGCAGAAGCCAGGATTCCCTCCTAAACTCTTGATATATAGGACATCCAATCTGGCTTCTGGAGTCCCAGCTCGCTTCAGTGGCAGTGGGTCTGGGACCTCTTACTCTCTCACAATTGGCACCATGGAGGCTGAAGGTGCTGCCACTTATTACTGCCAGCAGGGTAGTAGTTTACCACACA

IGKV4-91*01_S4811_AKR	AKR/J	IGKV	MUSMUS IGKV4-91*01 F	95.74	GAAATTGTGCTTACCCAGTCTCCAACCACCATGGCTGCATCTCCTGGGGAGAAGGTCACCATCACCTGCAGTGCCAGCTCAAGTATAAGTTCCAATTACTTGCACTGGTATCAGCAGAAGCCAGGATTCCCTCCTAAACTCTTGATATATAGGACATCCAATCTGGCTTCTGGAGTCCCAGCTCGCTTCAGTGGCAGTGGGTCTGGGACCTCTTACTCTCTCACAATTGGCACCATGGAGGCTGAAGGTGCTGCCACTTATTACTGCCAGCATGGTAGTAGTTTACTACGCA
IGKV4-91*01_S4811_MRL	MRL/MPJ	IGKV	MUSMUS IGKV4-91*01 F	95.74	GAAATTGTGCTTACCCAGTCTCCAACCACCATGGCTGCATCTCCTGGGGAGAAGGTCACCATCACCTGCAGTGCCAGCTCAAGTATAAGTTCCAATTACTTGCACTGGTATCAGCAGAAGCCAGGATTCCCTCCTAAACTCTTGATATATAGGACATCCAATCTGGCTTCTGGAGTCCCAGCTCGCTTCAGTGGCAGTGGGTCTGGGACCTCTTACTCTCTCACAATTGGCACCATGGAGGCTGAAGGTGCTGCCACTTATTACTGCCAGCATGGTAGTAGTTTACTACGCA
IGKV4-91*01_S5760_129	129S1/SVIMJ	IGKV	MUSMUS IGKV4-91*01 F	100	GAAATTGTGCTCACCCAGTCTCCAACCACCATGGCTGCATCTCCCGGGGAGAAGATCACTATCACCTGCAGTGCCAGCTCAAGTATAAGTTCCAATTACTTGCATTGGTATCAGCAGAAGCCAGGATTCTCCCCTAAACTCTTGATTTATAGGACATCCAATCTGGCTTCTGGAGTCCCAGCTCGCTTCAGTGGCAGTGGGTCTGGGACCTCTTACTCTCTCACAATTGGCACCATGGAGGCTGAAGATGTTGCCACTTACTACTGCCAGCAGGGTAGTAGTATACCGCTCA
IGKV4-91*01_S5853_PWD	PWD/PHJ	IGKV	MUSMUS IGKV4-91*01 F	96.81	GAAATTGTGCTCACCCAGTCTCCAACCACCATGGCTGCATCTCCCGGGGAGAAGGTCACCATCACCTGCAGTGCCAGCTCAAGTATAAGTTCCAATTACTTGCACTGGTATCAGCAGAAGCCAGGATTCCCTCCTAAACTCTTGATATATAGGACATCCAATCTGGCTTCTGGAGTCCCAGCTCGCTTCAGTGGCAGTGGGTCTGGGACCTCTTACTCTCTCACAATTGGCACCATGGAGGCTGAAGGTGCTGCCACTTATTACTGCCAGCAGGGTAGTAGTTTACCACTCA

IGKV4-91*01_S5997_129	129S1/SVIMJ	IGKV	MUSMUS IGKV4-91*01 F	100	GAAATTGTGCTCACCCAGTCTCCAACCACCATGGCTGCATCTCCCGGGGAGAAGATCACTATCACCTGCAGTGCCAGCTCAAGTATAAGTTCCAATTACTTGCATTGGTATCAGCAGAAGCCAGGATTCTCCCCTAAACTCTTGATTTATAGGACATCCAATCTGGCTTCTGGAGTCCCAGCTCGCTTCAGTGGCAGTGGGTCTGGGACCTCTTACTCTCTCACAATTGGCACCATGGAGGCTGAAGATGTTGCCACTTACTACTGCCAGCAGGGTAGTAGTATACCACTCA
IGKV4-91*01_S5997_AJ	A/J	IGKV	MUSMUS IGKV4-91*01 F	100	GAAATTGTGCTCACCCAGTCTCCAACCACCATGGCTGCATCTCCCGGGGAGAAGATCACTATCACCTGCAGTGCCAGCTCAAGTATAAGTTCCAATTACTTGCATTGGTATCAGCAGAAGCCAGGATTCTCCCCTAAACTCTTGATTTATAGGACATCCAATCTGGCTTCTGGAGTCCCAGCTCGCTTCAGTGGCAGTGGGTCTGGGACCTCTTACTCTCTCACAATTGGCACCATGGAGGCTGAAGATGTTGCCACTTACTACTGCCAGCAGGGTAGTAGTATACCACTCA
IGKV4-91*01_S5997_B6	C57BL/6J	IGKV	MUSMUS IGKV4-91*01 F	100	GAAATTGTGCTCACCCAGTCTCCAACCACCATGGCTGCATCTCCCGGGGAGAAGATCACTATCACCTGCAGTGCCAGCTCAAGTATAAGTTCCAATTACTTGCATTGGTATCAGCAGAAGCCAGGATTCTCCCCTAAACTCTTGATTTATAGGACATCCAATCTGGCTTCTGGAGTCCCAGCTCGCTTCAGTGGCAGTGGGTCTGGGACCTCTTACTCTCTCACAATTGGCACCATGGAGGCTGAAGATGTTGCCACTTACTACTGCCAGCAGGGTAGTAGTATACCACTCA
IGKV4-91*01_S5997_BALB	BALB/CBYJ	IGKV	MUSMUS IGKV4-91*01 F	100	GAAATTGTGCTCACCCAGTCTCCAACCACCATGGCTGCATCTCCCGGGGAGAAGATCACTATCACCTGCAGTGCCAGCTCAAGTATAAGTTCCAATTACTTGCATTGGTATCAGCAGAAGCCAGGATTCTCCCCTAAACTCTTGATTTATAGGACATCCAATCTGGCTTCTGGAGTCCCAGCTCGCTTCAGTGGCAGTGGGTCTGGGACCTCTTACTCTCTCACAATTGGCACCATGGAGGCTGAAGATGTTGCCACTTACTACTGCCAGCAGGGTAGTAGTATACCACTCA

IGKV4-91*01_S5997_C3H	C3H/HEJ	IGKV	MUSMUS IGKV4-91*01 F	100	GAAATTGTGCTCACCCAGTCTCCAACCACCATGGCTGCATCTCCCGGGGAGAAGATCACTATCACCTGCAGTGCCAGCTCAAGTATAAGTTCCAATTACTTGCATTGGTATCAGCAGAAGCCAGGATTCTCCCCTAAACTCTTGATTTATAGGACATCCAATCTGGCTTCTGGAGTCCCAGCTCGCTTCAGTGGCAGTGGGTCTGGGACCTCTTACTCTCTCACAATTGGCACCATGGAGGCTGAAGATGTTGCCACTTACTACTGCCAGCAGGGTAGTAGTATACCACTCA
IGKV4-91*01_S5997_CBA	CBA/J	IGKV	MUSMUS IGKV4-91*01 F	100	GAAATTGTGCTCACCCAGTCTCCAACCACCATGGCTGCATCTCCCGGGGAGAAGATCACTATCACCTGCAGTGCCAGCTCAAGTATAAGTTCCAATTACTTGCATTGGTATCAGCAGAAGCCAGGATTCTCCCCTAAACTCTTGATTTATAGGACATCCAATCTGGCTTCTGGAGTCCCAGCTCGCTTCAGTGGCAGTGGGTCTGGGACCTCTTACTCTCTCACAATTGGCACCATGGAGGCTGAAGATGTTGCCACTTACTACTGCCAGCAGGGTAGTAGTATACCACTCA
IGKV4-91*01_S5997_DBA1	DBA/1J	IGKV	MUSMUS IGKV4-91*01 F	100	GAAATTGTGCTCACCCAGTCTCCAACCACCATGGCTGCATCTCCCGGGGAGAAGATCACTATCACCTGCAGTGCCAGCTCAAGTATAAGTTCCAATTACTTGCATTGGTATCAGCAGAAGCCAGGATTCTCCCCTAAACTCTTGATTTATAGGACATCCAATCTGGCTTCTGGAGTCCCAGCTCGCTTCAGTGGCAGTGGGTCTGGGACCTCTTACTCTCTCACAATTGGCACCATGGAGGCTGAAGATGTTGCCACTTACTACTGCCAGCAGGGTAGTAGTATACCACTCA
IGKV4-91*01_S5997_DBA2	DBA/2J	IGKV	MUSMUS IGKV4-91*01 F	100	GAAATTGTGCTCACCCAGTCTCCAACCACCATGGCTGCATCTCCCGGGGAGAAGATCACTATCACCTGCAGTGCCAGCTCAAGTATAAGTTCCAATTACTTGCATTGGTATCAGCAGAAGCCAGGATTCTCCCCTAAACTCTTGATTTATAGGACATCCAATCTGGCTTCTGGAGTCCCAGCTCGCTTCAGTGGCAGTGGGTCTGGGACCTCTTACTCTCTCACAATTGGCACCATGGAGGCTGAAGATGTTGCCACTTACTACTGCCAGCAGGGTAGTAGTATACCACTCA

IGKV4-91*01_S5997_LEWES	LEWES/EIJ	IGKV	MUSMUS IGKV4-91*01 F	100	GAAATTGTGCTCACCCAGTCTCCAACCACCATGGCTGCATCTCCCGGGGAGAAGATCACTATCACCTGCAGTGCCAGCTCAAGTATAAGTTCCAATTACTTGCATTGGTATCAGCAGAAGCCAGGATTCTCCCCTAAACTCTTGATTTATAGGACATCCAATCTGGCTTCTGGAGTCCCAGCTCGCTTCAGTGGCAGTGGGTCTGGGACCTCTTACTCTCTCACAATTGGCACCATGGAGGCTGAAGATGTTGCCACTTACTACTGCCAGCAGGGTAGTAGTATACCACTCA
IGKV4-91*01_S5997_NOR	NOR/LTJ	IGKV	MUSMUS IGKV4-91*01 F	100	GAAATTGTGCTCACCCAGTCTCCAACCACCATGGCTGCATCTCCCGGGGAGAAGATCACTATCACCTGCAGTGCCAGCTCAAGTATAAGTTCCAATTACTTGCATTGGTATCAGCAGAAGCCAGGATTCTCCCCTAAACTCTTGATTTATAGGACATCCAATCTGGCTTCTGGAGTCCCAGCTCGCTTCAGTGGCAGTGGGTCTGGGACCTCTTACTCTCTCACAATTGGCACCATGGAGGCTGAAGATGTTGCCACTTACTACTGCCAGCAGGGTAGTAGTATACCACTCA
IGKV4-91*01_S5997_SJL	SJL/J	IGKV	MUSMUS IGKV4-91*01 F	100	GAAATTGTGCTCACCCAGTCTCCAACCACCATGGCTGCATCTCCCGGGGAGAAGATCACTATCACCTGCAGTGCCAGCTCAAGTATAAGTTCCAATTACTTGCATTGGTATCAGCAGAAGCCAGGATTCTCCCCTAAACTCTTGATTTATAGGACATCCAATCTGGCTTCTGGAGTCCCAGCTCGCTTCAGTGGCAGTGGGTCTGGGACCTCTTACTCTCTCACAATTGGCACCATGGAGGCTGAAGATGTTGCCACTTACTACTGCCAGCAGGGTAGTAGTATACCACTCA
IGKV4-92*01_129	129S1/SVIMJ	IGKV	MUSMUS IGKV4-92*01 F	100	GAAATGGTTCTCACCCAGTCTCCAGTATCCATAACTGCATCTCGAGGGGAGAAGGTCACCATCACCTGCCGTGCCAGCTCAAGTATAAGTTCCAATTACTTACACTGGTACCAGCAGAAGCCAGGATCCTCCCCTAAACTTTTGATTTATAGGACATCCATCCTGGCATCTGGAGTCCTAGACAGCTTCAGTGGCAGTGGGTCTGAGAGCTCTTACACTCTGACAATCAGCTGCATGCAGGACGAAGTTGCTGCCACTTACTATTGTCAGCAGGGGAGTAGTAGCCCACCA

IGKV4-92*01_AJ	A/J	IGKV	MUSMUS IGKV4-92*01 F	100	GAAATGGTTCTCACCCAGTCTCCAGTATCCATAACTGCATCTCGAGGGGAGAAGGTCACCATCACCTGCCGTGCCAGCTCAAGTATAAGTTCCAATTACTTACACTGGTACCAGCAGAAGCCAGGATCCTCCCCTAAACTTTTGATTTATAGGACATCCATCCTGGCATCTGGAGTCCTAGACAGCTTCAGTGGCAGTGGGTCTGAGAGCTCTTACACTCTGACAATCAGCTGCATGCAGGACGAAGTTGCTGCCACTTACTATTGTCAGCAGGGGAGTAGTAGCCCACCA
IGKV4-92*01_B6	C57BL/6J	IGKV	MUSMUS IGKV4-92*01 F	100	GAAATGGTTCTCACCCAGTCTCCAGTATCCATAACTGCATCTCGAGGGGAGAAGGTCACCATCACCTGCCGTGCCAGCTCAAGTATAAGTTCCAATTACTTACACTGGTACCAGCAGAAGCCAGGATCCTCCCCTAAACTTTTGATTTATAGGACATCCATCCTGGCATCTGGAGTCCTAGACAGCTTCAGTGGCAGTGGGTCTGAGAGCTCTTACACTCTGACAATCAGCTGCATGCAGGACGAAGTTGCTGCCACTTACTATTGTCAGCAGGGGAGTAGTAGCCCACCA
IGKV4-92*01_BALB	BALB/CBYJ	IGKV	MUSMUS IGKV4-92*01 F	100	GAAATGGTTCTCACCCAGTCTCCAGTATCCATAACTGCATCTCGAGGGGAGAAGGTCACCATCACCTGCCGTGCCAGCTCAAGTATAAGTTCCAATTACTTACACTGGTACCAGCAGAAGCCAGGATCCTCCCCTAAACTTTTGATTTATAGGACATCCATCCTGGCATCTGGAGTCCTAGACAGCTTCAGTGGCAGTGGGTCTGAGAGCTCTTACACTCTGACAATCAGCTGCATGCAGGACGAAGTTGCTGCCACTTACTATTGTCAGCAGGGGAGTAGTAGCCCACCA
IGKV4-92*01_C3H	C3H/HEJ	IGKV	MUSMUS IGKV4-92*01 F	100	GAAATGGTTCTCACCCAGTCTCCAGTATCCATAACTGCATCTCGAGGGGAGAAGGTCACCATCACCTGCCGTGCCAGCTCAAGTATAAGTTCCAATTACTTACACTGGTACCAGCAGAAGCCAGGATCCTCCCCTAAACTTTTGATTTATAGGACATCCATCCTGGCATCTGGAGTCCTAGACAGCTTCAGTGGCAGTGGGTCTGAGAGCTCTTACACTCTGACAATCAGCTGCATGCAGGACGAAGTTGCTGCCACTTACTATTGTCAGCAGGGGAGTAGTAGCCCACCA

IGKV4-92*01_CBA	CBA/J	IGKV	MUSMUS IGKV4-92*01 F	100	GAAATGGTTCTCACCCAGTCTCCAGTATCCATAACTGCATCTCGAGGGGAGAAGGTCACCATCACCTGCCGTGCCAGCTCAAGTATAAGTTCCAATTACTTACACTGGTACCAGCAGAAGCCAGGATCCTCCCCTAAACTTTTGATTTATAGGACATCCATCCTGGCATCTGGAGTCCTAGACAGCTTCAGTGGCAGTGGGTCTGAGAGCTCTTACACTCTGACAATCAGCTGCATGCAGGACGAAGTTGCTGCCACTTACTATTGTCAGCAGGGGAGTAGTAGCCCACCA
IGKV4-92*01_DBA1	DBA/1J	IGKV	MUSMUS IGKV4-92*01 F	100	GAAATGGTTCTCACCCAGTCTCCAGTATCCATAACTGCATCTCGAGGGGAGAAGGTCACCATCACCTGCCGTGCCAGCTCAAGTATAAGTTCCAATTACTTACACTGGTACCAGCAGAAGCCAGGATCCTCCCCTAAACTTTTGATTTATAGGACATCCATCCTGGCATCTGGAGTCCTAGACAGCTTCAGTGGCAGTGGGTCTGAGAGCTCTTACACTCTGACAATCAGCTGCATGCAGGACGAAGTTGCTGCCACTTACTATTGTCAGCAGGGGAGTAGTAGCCCACCA
IGKV4-92*01_DBA2	DBA/2J	IGKV	MUSMUS IGKV4-92*01 F	100	GAAATGGTTCTCACCCAGTCTCCAGTATCCATAACTGCATCTCGAGGGGAGAAGGTCACCATCACCTGCCGTGCCAGCTCAAGTATAAGTTCCAATTACTTACACTGGTACCAGCAGAAGCCAGGATCCTCCCCTAAACTTTTGATTTATAGGACATCCATCCTGGCATCTGGAGTCCTAGACAGCTTCAGTGGCAGTGGGTCTGAGAGCTCTTACACTCTGACAATCAGCTGCATGCAGGACGAAGTTGCTGCCACTTACTATTGTCAGCAGGGGAGTAGTAGCCCACCA
IGKV4-92*01_LEWES	LEWES/EIJ	IGKV	MUSMUS IGKV4-92*01 F	100	GAAATGGTTCTCACCCAGTCTCCAGTATCCATAACTGCATCTCGAGGGGAGAAGGTCACCATCACCTGCCGTGCCAGCTCAAGTATAAGTTCCAATTACTTACACTGGTACCAGCAGAAGCCAGGATCCTCCCCTAAACTTTTGATTTATAGGACATCCATCCTGGCATCTGGAGTCCTAGACAGCTTCAGTGGCAGTGGGTCTGAGAGCTCTTACACTCTGACAATCAGCTGCATGCAGGACGAAGTTGCTGCCACTTACTATTGTCAGCAGGGGAGTAGTAGCCCACCA

IGKV4-92*01_NZB	NZB/BLNJ	IGKV	MUSMUS IGKV4-92*01 F	100	GAAATGGTTCTCACCCAGTCTCCAGTATCCATAACTGCATCTCGAGGGGAGAAGGTCACCATCACCTGCCGTGCCAGCTCAAGTATAAGTTCCAATTACTTACACTGGTACCAGCAGAAGCCAGGATCCTCCCCTAAACTTTTGATTTATAGGACATCCATCCTGGCATCTGGAGTCCTAGACAGCTTCAGTGGCAGTGGGTCTGAGAGCTCTTACACTCTGACAATCAGCTGCATGCAGGACGAAGTTGCTGCCACTTACTATTGTCAGCAGGGGGAGTAGTAGCCCACCA
IGKV4-92*01_S1372_MSM	MSM/MSJ	IGKV	MUSMUS IGKV4-92*01 F	97.52	GAAATTGTTCTCACCCAGTCTCCAATATCCATAACTGCATCTCAAGGGGAGAAGGTCACCATCACCTGCCGTGCCAGCTCAAGTATAAGTTCCAATTACTTACACTGGTACCAGCAGAAGCCAGAATCCTCCCCTAAACTTTTGATTTATAGGACATCCATCCTGGCATCTGGAGTACTAGACAGCTTCAGTGGCAGTGGGTCTGAGAGCTCTTACACTCTGACAATCAGCTGCATGCAGGCCGAAGTTGCTTCCACTTACTATTGTCAGCAGGGGGAGTAGTAGTCCACCA
IGKV4-92*01_S4169_CAST	CAST/EIJ	IGKV	MUSMUS IGKV4-92*01 F	98.94	GAAATTGTTCTCACCCAGTCTCCAGTATCCATAACTGCATCTCGAGGGGAGAAGGTCACCATCACCTGCCGTGCCAGCTCAAGTATAAGTTCCAAATACTTACACTGGTACCAGCAGAAGCCAGAATCCTCCCCTAAACTTTTGATTTATAGGACATCCATCCTGGCATCTGGAGTCCTAGACAGCTTCAGTGGCAGTGGGTCTGAGAGCTCTTACACTCTGACAATCAGCTGCATGCAGGACGAAGTTGCTGCCACTTACTATTGTCAGCAGGGGGAGTAGTAGCCCACCA
IGKV4-92*01_S4902_AKR	AKR/J	IGKV	MUSMUS IGKV4-92*01 F	98.58	GAAATGGTTCTCACCCAGTCTCCAGTATCCATAACTGCATCTCGAGGGGAGAAGATCACCATCACCTGCCGTGCCAGCTCAAGTATAAGTTCCAATTACTTACACTGGTACCAGCAGAAGCCAGGATCCTCCCCTAAACTTTTGATTTATAGGACATCCATCCTGGCATCTGGAGTCCTAGACAACTTCAGTGGCAGTGGGTCTGAGAGCTCTTACACTCTGACAATCAGCTGCATGCAGGACGATGTTGCTGCCACTTACTACTGTCAGCAGGGGGAGTAGTAGCCCACCA

IGKV4-92*01_S5510_PWD	PWD/PHJ	IGKV	MUSMUS IGKV4-92*01 F	99.65	GAAATGGTTCTCACCCAGTCTCCAGTATCCATAACTGCATCTCGAGGGGAGAAGGTCACCATCACCTGCCGTGCCAGCTCAAGTATAAGTTCCAATTACTTACACTGGTACCAGCAGAAGCCAGGATCCTCCCCTAAACTTTTGATTTATAGGACATCCATCCTGGCATCTGGAGTCCTAGACAGCTTCAGTGGCAGTGGGTCTGAGAGCTCTTACACTCTGACAATCAGCTGCATGCAGGACGAAGTTGCTGCCACTTACTATTATCAGCAGGGGAGTAGTAGCCCACCA
IGKV4-92*01_SJL	SJL/J	IGKV	MUSMUS IGKV4-92*01 F	100	GAAATGGTTCTCACCCAGTCTCCAGTATCCATAACTGCATCTCGAGGGGAGAAGGTCACCATCACCTGCCGTGCCAGCTCAAGTATAAGTTCCAATTACTTACACTGGTACCAGCAGAAGCCAGGATCCTCCCCTAAACTTTTGATTTATAGGACATCCATCCTGGCATCTGGAGTCCTAGACAGCTTCAGTGGCAGTGGGTCTGAGAGCTCTTACACTCTGACAATCAGCTGCATGCAGGACGAAGTTGCTGCCACTTACTATTGTCAGCAGGGGAGTAGTAGCCCACCA
IGKV5-37*01_129	129S1/SVIMJ	IGKV	MUSMUS IGKV5-37*01 F	100	GACATCCTGCTGACCCAGTCTCCAGCCACCCTGTCTGTGACTCCAGGAGAAACAGTCAGTCTTTCCTGTAGGGCCAGCCAGAGTATTTACAAGAACCTACACTGGTATCAACAGAAATCACATCGGTCTCCAAGGCTTCTCATCAAGTATGCTTCTGATTCCATCTCTGGGATCCCCTCCAGGTTCACTGGCAGTGGATCAGGGACAGATTACACTCTCAGTATCAACAGTGTGAAGCCCGAAGATGAAGGAATATATTACTGTCTTCAAGGTTACAGCACACCTTC
IGKV5-37*01_AJ	A/J	IGKV	MUSMUS IGKV5-37*01 F	100	GACATCCTGCTGACCCAGTCTCCAGCCACCCTGTCTGTGACTCCAGGAGAAACAGTCAGTCTTTCCTGTAGGGCCAGCCAGAGTATTTACAAGAACCTACACTGGTATCAACAGAAATCACATCGGTCTCCAAGGCTTCTCATCAAGTATGCTTCTGATTCCATCTCTGGGATCCCCTCCAGGTTCACTGGCAGTGGATCAGGGACAGATTACACTCTCAGTATCAACAGTGTGAAGCCCGAAGATGAAGGAATATATTACTGTCTTCAAGGTTACAGCACACCTTC

IGKV5-37*01_B6	C57BL/6J	IGKV	MUSMUS IGKV5-37*01 F	100	GACATCCTGCTGACCCAGTCTCCAGCCACCCTGTCTGTGACTCCAGGAGAAACAGTCAGTCTTTCCTGTAGGGCCAGCCAGAGTATTTACAAGAACCTACACTGGTATCAACAGAAATCACATCGGTCTCCAAGGCTTCTCATCAAGTATGCTTCTGATTCCATCTCTGGGATCCCCTCCAGGTTCACTGGCAGTGGATCAGGGACAGATTACACTCTCAGTATCAACAGTGTGAAGCCCGAAGATGAAGGAATATATTACTGTCTTCAAGGTTACAGCACACCTTC
IGKV5-37*01_BALB	BALB/CBYJ	IGKV	MUSMUS IGKV5-37*01 F	100	GACATCCTGCTGACCCAGTCTCCAGCCACCCTGTCTGTGACTCCAGGAGAAACAGTCAGTCTTTCCTGTAGGGCCAGCCAGAGTATTTACAAGAACCTACACTGGTATCAACAGAAATCACATCGGTCTCCAAGGCTTCTCATCAAGTATGCTTCTGATTCCATCTCTGGGATCCCCTCCAGGTTCACTGGCAGTGGATCAGGGACAGATTACACTCTCAGTATCAACAGTGTGAAGCCCGAAGATGAAGGAATATATTACTGTCTTCAAGGTTACAGCACACCTTC
IGKV5-37*01_C3H	C3H/HEJ	IGKV	MUSMUS IGKV5-37*01 F	100	GACATCCTGCTGACCCAGTCTCCAGCCACCCTGTCTGTGACTCCAGGAGAAACAGTCAGTCTTTCCTGTAGGGCCAGCCAGAGTATTTACAAGAACCTACACTGGTATCAACAGAAATCACATCGGTCTCCAAGGCTTCTCATCAAGTATGCTTCTGATTCCATCTCTGGGATCCCCTCCAGGTTCACTGGCAGTGGATCAGGGACAGATTACACTCTCAGTATCAACAGTGTGAAGCCCGAAGATGAAGGAATATATTACTGTCTTCAAGGTTACAGCACACCTTC
IGKV5-37*01_DBA1	DBA/1J	IGKV	MUSMUS IGKV5-37*01 F	100	GACATCCTGCTGACCCAGTCTCCAGCCACCCTGTCTGTGACTCCAGGAGAAACAGTCAGTCTTTCCTGTAGGGCCAGCCAGAGTATTTACAAGAACCTACACTGGTATCAACAGAAATCACATCGGTCTCCAAGGCTTCTCATCAAGTATGCTTCTGATTCCATCTCTGGGATCCCCTCCAGGTTCACTGGCAGTGGATCAGGGACAGATTACACTCTCAGTATCAACAGTGTGAAGCCCGAAGATGAAGGAATATATTACTGTCTTCAAGGTTACAGCACACCTTC

IGKV5-37*01_DBA2	DBA/2J	IGKV	MUSMUS IGKV5-37*01 F	100	GACATCCTGCTGACCCAGTCTC<br>CAGCCACCCTGTCTGTGACTCC<br>AGGAGAAACAGTCAGTCTTTCCT<br>GTAGGGCCAGCCAGAGTATTTA<br>CAAGAACCTACACTGGTATCAA<br>CAGAAATCACATCGGTCTCCAA<br>GGCTTCTCATCAAGTATGCTTCT<br>GATTCCATCTCTGGGATCCCCT<br>CCAGGTTCACTGGCAGTGGATC<br>AGGGACAGATTACACTCTCAGT<br>ATCAACAGTGTGAAGCCCGAAG<br>ATGAAGGAATATATTACTGTCTTC<br>AAGGTTACAGCACACCTTC
IGKV5-37*01_LEWES	LEWES/EIJ	IGKV	MUSMUS IGKV5-37*01 F	100	GACATCCTGCTGACCCAGTCTC<br>CAGCCACCCTGTCTGTGACTCC<br>AGGAGAAACAGTCAGTCTTTCCT<br>GTAGGGCCAGCCAGAGTATTTA<br>CAAGAACCTACACTGGTATCAA<br>CAGAAATCACATCGGTCTCCAA<br>GGCTTCTCATCAAGTATGCTTCT<br>GATTCCATCTCTGGGATCCCCT<br>CCAGGTTCACTGGCAGTGGATC<br>AGGGACAGATTACACTCTCAGT<br>ATCAACAGTGTGAAGCCCGAAG<br>ATGAAGGAATATATTACTGTCTTC<br>AAGGTTACAGCACACCTTC
IGKV5-37*01_NZB	NZB/BLNJ	IGKV	MUSMUS IGKV5-37*01 F	100	GACATCCTGCTGACCCAGTCTC<br>CAGCCACCCTGTCTGTGACTCC<br>AGGAGAAACAGTCAGTCTTTCCT<br>GTAGGGCCAGCCAGAGTATTTA<br>CAAGAACCTACACTGGTATCAA<br>CAGAAATCACATCGGTCTCCAA<br>GGCTTCTCATCAAGTATGCTTCT<br>GATTCCATCTCTGGGATCCCCT<br>CCAGGTTCACTGGCAGTGGATC<br>AGGGACAGATTACACTCTCAGT<br>ATCAACAGTGTGAAGCCCGAAG<br>ATGAAGGAATATATTACTGTCTTC<br>AAGGTTACAGCACACCTTC
IGKV5-37*01_S6352_CAST	CAST/EIJ	IGKV	MUSMUS IGKV5-37*01 F	97.85	GACATCCTGATGACCCAGTCTC<br>CAGCCACCCTGTCTGTGACTCC<br>AGGAGAAACAGTCAGTCTTTCCT<br>GTAGGGCCAGCCAGAATATTTA<br>CAAGAACCTACACTGGTATCAA<br>CAGAAATCACATGGGACTCCAA<br>GGCTTCTCATCAAGTATGCATCT<br>GATCCCATCTCTGGGATCCCCT<br>CCAGGTTCACTGGCAGTGGATC<br>AGGGACAGATTACACTCTCAGT<br>ATCAACAGTGTGAAGCCCGAAG<br>ATGAAGGAATATATTACTGTCTTC<br>AAGGTTACAGCATGCCT

IGKV5-37*01_S7203_NOR	NOR/LTJ	IGKV	MUSMUS IGKV5-37*01 F	97.13	GACATCCTGATGACCCAGTCTCCAGCCACCCTGTCTGTGACTCCAGGAGAAACAGTCAGTCTTTCCTGTAGGGCCAGCCAGAATACTTACAAGAACCTACACTGGTATCAACAGAAATCACATGGGACTCCAAAGCTTCTCATCAAGTATGCATCTGATCCCATCTCTGGGATCCCCTCCAGGTTCACTGGCAGTGGATCAGGGACAGATTACACTCTCAGTATCAACAGTGTGAAGCCCGAAGATGAAGGAATATATTACTGTCTTCAAGGTTACAGCATGCCTT
IGKV5-37*01_S9729_AKR	AKR/J	IGKV	MUSMUS IGKV5-37*01 F	97.13	GACATCCTGATGACCCAGTCTCCAGCCACCCTGTCTGTGACTCCAGGAGAAACAGTCAGTCTTTCCTGTAGGGCCAGCCAGAATACTTACAAGAACCTACACTGGTATCAACAGAAATCACATGGGACTCCAAAGCTTCTCATCAAGTATGCATCTGATCCCATCTCTGGGATCCCCTCCAGGTTCACTGGCAGTGGATCAGGGACAGATTACACTCTCAGTATCAACAGTGTGAAGCCCGAAGATGAAGGAATATATTACTGTCTTCAAGGTTACAGCATGCCT
IGKV5-37*01_S9729_MRL	MRL/MPJ	IGKV	MUSMUS IGKV5-37*01 F	97.13	GACATCCTGATGACCCAGTCTCCAGCCACCCTGTCTGTGACTCCAGGAGAAACAGTCAGTCTTTCCTGTAGGGCCAGCCAGAATACTTACAAGAACCTACACTGGTATCAACAGAAATCACATGGGACTCCAAAGCTTCTCATCAAGTATGCATCTGATCCCATCTCTGGGATCCCCTCCAGGTTCACTGGCAGTGGATCAGGGACAGATTACACTCTCAGTATCAACAGTGTGAAGCCCGAAGATGAAGGAATATATTACTGTCTTCAAGGTTACAGCATGCCT
IGKV5-39*01_129	129S1/SVIMJ	IGKV	MUSMUS IGKV5-39*01 F	100	GACATTGTGATGACTCAGTCTCCAGCCACCCTGTCTGTGACTCCAGGAGATAGAGTCTCTCTTTCCTGCAGGGCCAGCCAGAGTATTAGCGACTACTTACACTGGTATCAACAAAAATCACATGAGTCTCCAAGGCTTCTCATCAAATATGCTTCCCAATCCATCTCTGGGATCCCCTCCAGGTTCAGTGGCAGTGGATCAGGGTCAGATTTCACTCTCAGTATCAACAGTGTGGAACCTGAAGATGTTGGAGTGTATTACTGTCAAAATGGTCACAGCTTTCCTCC

IGKV5-39*01_AJ	A/J	IGKV	MUSMUS IGKV5-39*01 F	100	GACATTGTGATGACTCAGTCTCCAGCCACCCTGTCTGTGACTCCAGGAGATAGAGTCTCTCTTTCCTGCAGGGCCAGCCAGAGTATTAGCGACTACTTACACTGGTATCAACAAAAATCACATGAGTCTCCAAGGCTTCTCATCAAATATGCTTCCCAATCCATCTCTGGGATCCCCTCCAGGTTCAGTGGCAGTGGATCAGGGTCAGATTTCACTCTCAGTATCAACAGTGTGGAACCTGAAGATGTTGGAGTGTATTACTGTCAAAATGGTCACAGCTTTCCTCC
IGKV5-39*01_B6	C57BL/6J	IGKV	MUSMUS IGKV5-39*01 F	100	GACATTGTGATGACTCAGTCTCCAGCCACCCTGTCTGTGACTCCAGGAGATAGAGTCTCTCTTTCCTGCAGGGCCAGCCAGAGTATTAGCGACTACTTACACTGGTATCAACAAAAATCACATGAGTCTCCAAGGCTTCTCATCAAATATGCTTCCCAATCCATCTCTGGGATCCCCTCCAGGTTCAGTGGCAGTGGATCAGGGTCAGATTTCACTCTCAGTATCAACAGTGTGGAACCTGAAGATGTTGGAGTGTATTACTGTCAAAATGGTCACAGCTTTCCTCC
IGKV5-39*01_BALB	BALB/CBYJ	IGKV	MUSMUS IGKV5-39*01 F	100	GACATTGTGATGACTCAGTCTCCAGCCACCCTGTCTGTGACTCCAGGAGATAGAGTCTCTCTTTCCTGCAGGGCCAGCCAGAGTATTAGCGACTACTTACACTGGTATCAACAAAAATCACATGAGTCTCCAAGGCTTCTCATCAAATATGCTTCCCAATCCATCTCTGGGATCCCCTCCAGGTTCAGTGGCAGTGGATCAGGGTCAGATTTCACTCTCAGTATCAACAGTGTGGAACCTGAAGATGTTGGAGTGTATTACTGTCAAAATGGTCACAGCTTTCCTCC
IGKV5-39*01_C3H	C3H/HEJ	IGKV	MUSMUS IGKV5-39*01 F	100	GACATTGTGATGACTCAGTCTCCAGCCACCCTGTCTGTGACTCCAGGAGATAGAGTCTCTCTTTCCTGCAGGGCCAGCCAGAGTATTAGCGACTACTTACACTGGTATCAACAAAAATCACATGAGTCTCCAAGGCTTCTCATCAAATATGCTTCCCAATCCATCTCTGGGATCCCCTCCAGGTTCAGTGGCAGTGGATCAGGGTCAGATTTCACTCTCAGTATCAACAGTGTGGAACCTGAAGATGTTGGAGTGTATTACTGTCAAAATGGTCACAGCTTTCCTCC

IGKV5-39*01_CBA	CBA/J	IGKV	MUSMUS IGKV5-39*01 F	100	GACATTGTGATGACTCAGTCTCCAGCCACCCTGTCTGTGACTCCAGGAGATAGAGTCTCTCTTTCCTGCAGGGCCAGCCAGAGTATTAGCGACTACTTACACTGGTATCAACAAAAATCACATGAGTCTCCAAGGCTTCTCATCAAATATGCTTCCCAATCCATCTCTGGGATCCCCTCCAGGTTCAGTGGCAGTGGATCAGGGTCAGATTTCACTCTCAGTATCAACAGTGTGGAACCTGAAGATGTTGGAGTGTATTACTGTCAAAATGGTCACAGCTTTCCTCC
IGKV5-39*01_DBA1	DBA/1J	IGKV	MUSMUS IGKV5-39*01 F	100	GACATTGTGATGACTCAGTCTCCAGCCACCCTGTCTGTGACTCCAGGAGATAGAGTCTCTCTTTCCTGCAGGGCCAGCCAGAGTATTAGCGACTACTTACACTGGTATCAACAAAAATCACATGAGTCTCCAAGGCTTCTCATCAAATATGCTTCCCAATCCATCTCTGGGATCCCCTCCAGGTTCAGTGGCAGTGGATCAGGGTCAGATTTCACTCTCAGTATCAACAGTGTGGAACCTGAAGATGTTGGAGTGTATTACTGTCAAAATGGTCACAGCTTTCCTCC
IGKV5-39*01_DBA2	DBA/2J	IGKV	MUSMUS IGKV5-39*01 F	100	GACATTGTGATGACTCAGTCTCCAGCCACCCTGTCTGTGACTCCAGGAGATAGAGTCTCTCTTTCCTGCAGGGCCAGCCAGAGTATTAGCGACTACTTACACTGGTATCAACAAAAATCACATGAGTCTCCAAGGCTTCTCATCAAATATGCTTCCCAATCCATCTCTGGGATCCCCTCCAGGTTCAGTGGCAGTGGATCAGGGTCAGATTTCACTCTCAGTATCAACAGTGTGGAACCTGAAGATGTTGGAGTGTATTACTGTCAAAATGGTCACAGCTTTCCTCC
IGKV5-39*01_LEWES	LEWES/EIJ	IGKV	MUSMUS IGKV5-39*01 F	100	GACATTGTGATGACTCAGTCTCCAGCCACCCTGTCTGTGACTCCAGGAGATAGAGTCTCTCTTTCCTGCAGGGCCAGCCAGAGTATTAGCGACTACTTACACTGGTATCAACAAAAATCACATGAGTCTCCAAGGCTTCTCATCAAATATGCTTCCCAATCCATCTCTGGGATCCCCTCCAGGTTCAGTGGCAGTGGATCAGGGTCAGATTTCACTCTCAGTATCAACAGTGTGGAACCTGAAGATGTTGGAGTGTATTACTGTCAAAATGGTCACAGCTTTCCTCC

IGKV5-39*01_NZB	NZB/BLNJ	IGKV	MUSMUS IGKV5-39*01 F	100	GACATTGTGATGACTCAGTCTCCAGCCACCCTGTCTGTGACTCCAGGAGATAGAGTCTCTCTTTCCTGCAGGGCCAGCCAGAGTATTAGCGACTACTTACACTGGTATCAACAAAAATCACATGAGTCTCCAAGGCTTCTCATCAAATATGCTTCCCAATCCATCTCTGGGATCCCCTCCAGGTTCAGTGGCAGTGGATCAGGGTCAGATTTCACTCTCAGTATCAACAGTGTGGAACCTGAAGATGTTGGAGTGTATTACTGTCAAAATGGTCACAGCTTTCCTCC
IGKV5-39*01_S0817_MSM	MSM/MSJ	IGKV	MUSMUS IGKV5-39*01 F	99.64	GACATTGTGATGACTCAGTCTCCAGCCACCCTGTCTGTGACTCCAGGAGATAGAGTCTCTCTTTCCTGCAGGGCCAGCCAGAGTATTAGCAACTACTTACACTGGTATCAACAAAAATCACATGAGTCTCCAAGGCTTCTCATCAAATATGCTTCCCAATCCATCTCTGGGATCCCCTCCAGGTTCAGTGGCAGTGGATCAGGGTCAGATTTCACTCTCAGTATCAACAGTGTGGAACCTGAAGATGTTGGAGTGTATTACTGTCAAAATGGTCACAGCTTTCCTCC
IGKV5-39*01_S0817_PWD	PWD/PHJ	IGKV	MUSMUS IGKV5-39*01 F	99.64	GACATTGTGATGACTCAGTCTCCAGCCACCCTGTCTGTGACTCCAGGAGATAGAGTCTCTCTTTCCTGCAGGGCCAGCCAGAGTATTAGCAACTACTTACACTGGTATCAACAAAAATCACATGAGTCTCCAAGGCTTCTCATCAAATATGCTTCCCAATCCATCTCTGGGATCCCCTCCAGGTTCAGTGGCAGTGGATCAGGGTCAGATTTCACTCTCAGTATCAACAGTGTGGAACCTGAAGATGTTGGAGTGTATTACTGTCAAAATGGTCACAGCTTTCCTCC
IGKV5-39*01_S3142_AKR	AKR/J	IGKV	MUSMUS IGKV5-39*01 F	98.92	GACATTGTGATGACTCAGTCTCCAGCCACCCTGTCTGTGACTCCAGGAGATAGGGTCTCTCTTTCCTGCAGGGCCAGTCAGAGTATTAGCGACTACTTACACTGGTATCAACAAAAATCACATGAGTCTCCAAGGCTTCTCATCAAGTATGCTTCCCAATCCATCTCTGGGATCCCCTCCAGGTTCAGTGGCAGTGGATCAGGGTCAGATTTCACTCTCAGTATCAACAGTGTGGAACCTGAAGATGTTGGAGTGTATTACTGTCAAAATGGTCACAGCTTTCCTCC

IGKV5-39*01_S3142_MRL	MRL/MPJ	IGKV	MUSMUS IGKV5-39*01 F	98.92	GACATTGTGATGACTCAGTCTCC<br>AGCCACCCTGTCTGTGACTCCA<br>GGAGATAGGGTCTCTCTTTCCT<br>GCAGGGCCAGTCAGAGTATTAG<br>CGACTACTTACACTGGTATCAAC<br>AAAAATCACATGAGTCTCCAAG<br>GCTTCTCATCAAGTATGCTTCCC<br>AATCCATCTCTGGGATCCCCTC<br>CAGGTTCAGTGGCAGTGGATCA<br>GGGTCAGATTTCACTCTCAGTAT<br>CAACAGTGTGGAACCTGAAGAT<br>GTTGGAGTGTATTACTGTCAAAA<br>TGGTCACAGCTTTCCTCC
IGKV5-39*01_S3142_NOR	NOR/LTJ	IGKV	MUSMUS IGKV5-39*01 F	98.92	GACATTGTGATGACTCAGTCTCC<br>AGCCACCCTGTCTGTGACTCCA<br>GGAGATAGGGTCTCTCTTTCCT<br>GCAGGGCCAGTCAGAGTATTAG<br>CGACTACTTACACTGGTATCAAC<br>AAAAATCACATGAGTCTCCAAG<br>GCTTCTCATCAAGTATGCTTCCC<br>AATCCATCTCTGGGATCCCCTC<br>CAGGTTCAGTGGCAGTGGATCA<br>GGGTCAGATTTCACTCTCAGTAT<br>CAACAGTGTGGAACCTGAAGAT<br>GTTGGAGTGTATTACTGTCAAAA<br>TGGTCACAGCTTTCCTCC
IGKV5-39*01_S7300_CAST	CAST/EIJ	IGKV	MUSMUS IGKV5-39*01 F	99.28	GACATTGTGATGACTCAGTCTCC<br>AGCCACCCTGTCTGTGACTCCA<br>GGAGATAGAGTCTCTCTTTCCTG<br>CAGGGCCAGCCAGAGTATTAG<br>CAACTACTTACATTGGTATCAAC<br>AAAAATCACATGAGTCTCCAAG<br>GCTTCTCATCAAATATGCTTCCC<br>AATCCATCTCTGGGATCCCCTC<br>CAGGTTCAGTGGCAGTGGATCA<br>GGGTCAGATTTCACTCTCAGTAT<br>CAACAGTGTGGAACCTGAAGAT<br>GTTGGAGTGTATTACTGTCAAAA<br>TGGTCACAGCTTTCCTCC
IGKV5-39*01_SJL	SJL/J	IGKV	MUSMUS IGKV5-39*01 F	100	GACATTGTGATGACTCAGTCTCC<br>AGCCACCCTGTCTGTGACTCCA<br>GGAGATAGAGTCTCTCTTTCCTG<br>CAGGGCCAGCCAGAGTATTAG<br>CGACTACTTACACTGGTATCAAC<br>AAAAATCACATGAGTCTCCAAG<br>GCTTCTCATCAAATATGCTTCCC<br>AATCCATCTCTGGGATCCCCTC<br>CAGGTTCAGTGGCAGTGGATCA<br>GGGTCAGATTTCACTCTCAGTAT<br>CAACAGTGTGGAACCTGAAGAT<br>GTTGGAGTGTATTACTGTCAAAA<br>TGGTCACAGCTTTCCTCC

IGKV5-43*01_129	129S1/SVIMJ	IGKV	MUSMUS IGKV5-43*01 F	100	GATATTGTGCTAACTCAGTCTCC<br>AGCCACCCTGTCTGTGACTCCA<br>GGAGATAGCGTCAGTCTTTCCT<br>GCAGGGCCAGCCAAAGTATTAG<br>CAACAACCTACACTGGTATCAA<br>CAAAAATCACATGAGTCTCCAA<br>GGCTTCTCATCAAGTATGCTTCC<br>CAGTCCATCTCTGGGATCCCCT<br>CCAGGTTCAGTGGCAGTGGATC<br>AGGGACAGATTTCACTCTCAGTA<br>TCAACAGTGTGGAGACTGAAGA<br>TTTTGGAATGTATTTCTGTCAACA<br>GAGTAACAGCTGGCCTCA
IGKV5-43*01_AJ	A/J	IGKV	MUSMUS IGKV5-43*01 F	100	GATATTGTGCTAACTCAGTCTCC<br>AGCCACCCTGTCTGTGACTCCA<br>GGAGATAGCGTCAGTCTTTCCT<br>GCAGGGCCAGCCAAAGTATTAG<br>CAACAACCTACACTGGTATCAA<br>CAAAAATCACATGAGTCTCCAA<br>GGCTTCTCATCAAGTATGCTTCC<br>CAGTCCATCTCTGGGATCCCCT<br>CCAGGTTCAGTGGCAGTGGATC<br>AGGGACAGATTTCACTCTCAGTA<br>TCAACAGTGTGGAGACTGAAGA<br>TTTTGGAATGTATTTCTGTCAACA<br>GAGTAACAGCTGGCCTCA
IGKV5-43*01_B6	C57BL/6J	IGKV	MUSMUS IGKV5-43*01 F	100	GATATTGTGCTAACTCAGTCTCC<br>AGCCACCCTGTCTGTGACTCCA<br>GGAGATAGCGTCAGTCTTTCCT<br>GCAGGGCCAGCCAAAGTATTAG<br>CAACAACCTACACTGGTATCAA<br>CAAAAATCACATGAGTCTCCAA<br>GGCTTCTCATCAAGTATGCTTCC<br>CAGTCCATCTCTGGGATCCCCT<br>CCAGGTTCAGTGGCAGTGGATC<br>AGGGACAGATTTCACTCTCAGTA<br>TCAACAGTGTGGAGACTGAAGA<br>TTTTGGAATGTATTTCTGTCAACA<br>GAGTAACAGCTGGCCTCA
IGKV5-43*01_BALB	BALB/CBYJ	IGKV	MUSMUS IGKV5-43*01 F	100	GATATTGTGCTAACTCAGTCTCC<br>AGCCACCCTGTCTGTGACTCCA<br>GGAGATAGCGTCAGTCTTTCCT<br>GCAGGGCCAGCCAAAGTATTAG<br>CAACAACCTACACTGGTATCAA<br>CAAAAATCACATGAGTCTCCAA<br>GGCTTCTCATCAAGTATGCTTCC<br>CAGTCCATCTCTGGGATCCCCT<br>CCAGGTTCAGTGGCAGTGGATC<br>AGGGACAGATTTCACTCTCAGTA<br>TCAACAGTGTGGAGACTGAAGA<br>TTTTGGAATGTATTTCTGTCAACA<br>GAGTAACAGCTGGCCTCA

IGKV5-43*01_C3H	C3H/HEJ	IGKV	MUSMUS IGKV5-43*01 F	100	GATATTGTGCTAACTCAGTCTCCAGCCACCCTGTCTGTGACTCCAGGAGATAGCGTCAGTCTTTCCTGCAGGGCCAGCCAAAGTATTAGCAACAACCTACACTGGTATCAACAAAAATCACATGAGTCTCCAAGGCTTCTCATCAAGTATGCTTCCCAGTCCATCTCTGGGATCCCCTCCAGGTTCAGTGGCAGTGGATCAGGGACAGATTTCACTCTCAGTATCAACAGTGTGGAGACTGAAGATTTTGGAATGTATTTCTGTCAACAGAGTAACAGCTGGCCTCA
IGKV5-43*01_CBA	CBA/J	IGKV	MUSMUS IGKV5-43*01 F	100	GATATTGTGCTAACTCAGTCTCCAGCCACCCTGTCTGTGACTCCAGGAGATAGCGTCAGTCTTTCCTGCAGGGCCAGCCAAAGTATTAGCAACAACCTACACTGGTATCAACAAAAATCACATGAGTCTCCAAGGCTTCTCATCAAGTATGCTTCCCAGTCCATCTCTGGGATCCCCTCCAGGTTCAGTGGCAGTGGATCAGGGACAGATTTCACTCTCAGTATCAACAGTGTGGAGACTGAAGATTTTGGAATGTATTTCTGTCAACAGAGTAACAGCTGGCCTCA
IGKV5-43*01_DBA1	DBA/1J	IGKV	MUSMUS IGKV5-43*01 F	100	GATATTGTGCTAACTCAGTCTCCAGCCACCCTGTCTGTGACTCCAGGAGATAGCGTCAGTCTTTCCTGCAGGGCCAGCCAAAGTATTAGCAACAACCTACACTGGTATCAACAAAAATCACATGAGTCTCCAAGGCTTCTCATCAAGTATGCTTCCCAGTCCATCTCTGGGATCCCCTCCAGGTTCAGTGGCAGTGGATCAGGGACAGATTTCACTCTCAGTATCAACAGTGTGGAGACTGAAGATTTTGGAATGTATTTCTGTCAACAGAGTAACAGCTGGCCTCA
IGKV5-43*01_DBA2	DBA/2J	IGKV	MUSMUS IGKV5-43*01 F	100	GATATTGTGCTAACTCAGTCTCCAGCCACCCTGTCTGTGACTCCAGGAGATAGCGTCAGTCTTTCCTGCAGGGCCAGCCAAAGTATTAGCAACAACCTACACTGGTATCAACAAAAATCACATGAGTCTCCAAGGCTTCTCATCAAGTATGCTTCCCAGTCCATCTCTGGGATCCCCTCCAGGTTCAGTGGCAGTGGATCAGGGACAGATTTCACTCTCAGTATCAACAGTGTGGAGACTGAAGATTTTGGAATGTATTTCTGTCAACAGAGTAACAGCTGGCCTCA

IGKV5-43*01_LEWES	LEWES/EIJ	IGKV	MUSMUS IGKV5-43*01 F	100	GATATTGTGCTAACTCAGTCTCC<br>AGCCACCCTGTCTGTGACTCCA<br>GGAGATAGCGTCAGTCTTTCCT<br>GCAGGGCCAGCCAAAGTATTAG<br>CAACAACCTACACTGGTATCAA<br>CAAAAATCACATGAGTCTCCAA<br>GGCTTCTCATCAAGTATGCTTCC<br>CAGTCCATCTCTGGGATCCCCT<br>CCAGGTTCAGTGGCAGTGGATC<br>AGGGACAGATTTCACTCTCAGTA<br>TCAACAGTGTGGAGACTGAAGA<br>TTTTGGAATGTATTTCTGTCAACA<br>GAGTAACAGCTGGCCTCA
IGKV5-43*01_NZB	NZB/BLNJ	IGKV	MUSMUS IGKV5-43*01 F	100	GATATTGTGCTAACTCAGTCTCC<br>AGCCACCCTGTCTGTGACTCCA<br>GGAGATAGCGTCAGTCTTTCCT<br>GCAGGGCCAGCCAAAGTATTAG<br>CAACAACCTACACTGGTATCAA<br>CAAAAATCACATGAGTCTCCAA<br>GGCTTCTCATCAAGTATGCTTCC<br>CAGTCCATCTCTGGGATCCCCT<br>CCAGGTTCAGTGGCAGTGGATC<br>AGGGACAGATTTCACTCTCAGTA<br>TCAACAGTGTGGAGACTGAAGA<br>TTTTGGAATGTATTTCTGTCAACA<br>GAGTAACAGCTGGCCTCA
IGKV5-43*01_S4957_CAST	CAST/EIJ	IGKV	MUSMUS IGKV5-43*01 F	98.57	GATATTGTGCTAACTCAGTCTCC<br>AGCCACCCTGTCTGTGATTCCA<br>GGAGATAGCGTCAGTCTTTCCT<br>GCAGGGCGAGCCAAAGTATTAG<br>CAACAACCTACACTGGTATCAA<br>CAAAAAACACATGAGTCTCCAA<br>GGCTTCTCATCAAGTATGCTTCC<br>CAATCCATCTCTGGGATCCCCT<br>CCAGGTTCAGTGGCAGTGGATC<br>AGGGACAGATTTCACTCTCAGTA<br>TCAACAGTGTGGAGACTGAAGA<br>TTTTGGAATGTATTTCTGTCAACA<br>GAGTAACAGCTGGCCTCA
IGKV5-43*01_S9511_AKR	AKR/J	IGKV	MUSMUS IGKV5-43*01 F	99.64	GATATTGTGCTAACTCAGTCTCC<br>AGCCACCCTGTCTGTGACTCCA<br>GGAGATAGCGTCAGTCTTTCCT<br>GCAGGGCCAGCCAAAGTATTAG<br>CAACAACCTACACTGGTATCAA<br>CAAAAATCACATGAGTCTCCAA<br>GGCTTCTCATCAAGTATGCTTCC<br>CAGTCCATCTCTGGGATCCCCT<br>CCAAGTTCAGTGGCAGTGGATC<br>AGGGACAGATTTCACTCTCAGTA<br>TCAACAGTGTGGAGACTGAAGA<br>TTTTGGAATGTATTTCTGTCAACA<br>GAGTAACAGCTGGCCTCA

IGKV5-43*01_S9511_MRL	MRL/MPJ	IGKV	MUSMUS IGKV5-43*01 F	99.64	GATATTGTGCTAACTCAGTCTCCAGCCACCCTGTCTGTGACTCCAGGAGATAGCGTCAGTCTTTCCTGCAGGGCCAGCCAAAGTATTAGCAACAACCTACACTGGTATCAACAAAAATCACATGAGTCTCCAAGGCTTCTCATCAAGTATGCTTCCCAGTCCATCTCTGGGATCCCCTCCAAGTTCAGTGGCAGTGGATCAGGGACAGATTTCACTCTCAGTATCAACAGTGTGGAGACTGAAGATTTTGGAATGTATTTCTGTCAACAGAGTAACAGCTGGCCTCA
IGKV5-43*01_S9511_NOD	NOD/SHILTJ	IGKV	MUSMUS IGKV5-43*01 F	99.64	GATATTGTGCTAACTCAGTCTCCAGCCACCCTGTCTGTGACTCCAGGAGATAGCGTCAGTCTTTCCTGCAGGGCCAGCCAAAGTATTAGCAACAACCTACACTGGTATCAACAAAAATCACATGAGTCTCCAAGGCTTCTCATCAAGTATGCTTCCCAGTCCATCTCTGGGATCCCCTCCAAGTTCAGTGGCAGTGGATCAGGGACAGATTTCACTCTCAGTATCAACAGTGTGGAGACTGAAGATTTTGGAATGTATTTCTGTCAACAGAGTAACAGCTGGCCTCA
IGKV5-43*01_S9511_NOR	NOR/LTJ	IGKV	MUSMUS IGKV5-43*01 F	99.64	GATATTGTGCTAACTCAGTCTCCAGCCACCCTGTCTGTGACTCCAGGAGATAGCGTCAGTCTTTCCTGCAGGGCCAGCCAAAGTATTAGCAACAACCTACACTGGTATCAACAAAAATCACATGAGTCTCCAAGGCTTCTCATCAAGTATGCTTCCCAGTCCATCTCTGGGATCCCCTCCAAGTTCAGTGGCAGTGGATCAGGGACAGATTTCACTCTCAGTATCAACAGTGTGGAGACTGAAGATTTTGGAATGTATTTCTGTCAACAGAGTAACAGCTGGCCTCA
IGKV5-43*01_SJL	SJL/J	IGKV	MUSMUS IGKV5-43*01 F	100	GATATTGTGCTAACTCAGTCTCCAGCCACCCTGTCTGTGACTCCAGGAGATAGCGTCAGTCTTTCCTGCAGGGCCAGCCAAAGTATTAGCAACAACCTACACTGGTATCAACAAAAATCACATGAGTCTCCAAGGCTTCTCATCAAGTATGCTTCCCAGTCCATCTCTGGGATCCCCTCCAGGTTCAGTGGCAGTGGATCAGGGACAGATTTCACTCTCAGTATCAACAGTGTGGAGACTGAAGATTTTGGAATGTATTTCTGTCAACAGAGTAACAGCTGGCCTCA

IGKV5-45*01_129	129S1/SVIMJ	IGKV	MUSMUS IGKV5-45*01 F	100	GATATTGTGCTAACTCAGTCTCCAGCCACCCTGTCTGTGACTCCAGGAGATAGAGTCAGTCTTTCCTGCAGGGCCAGTCAAAGTATTAGCAACTACCTACACTGGTATCAACAAAAATCACATGAGTCTCCAAGGCTTCTCATCAAGTATGCTTCCCAGTCCATCTCTGGGATCCCCTCCAGGTTCAGTGGCAGTGGATCAGGGACAGATTTCACTCTCAGTATCAACAGTGTGGAGACTGAAGATTTTGGAATGTATTTCTGTCAACAGAGTAACAGCTGGCCTCA
IGKV5-45*01_AJ	A/J	IGKV	MUSMUS IGKV5-45*01 F	100	GATATTGTGCTAACTCAGTCTCCAGCCACCCTGTCTGTGACTCCAGGAGATAGAGTCAGTCTTTCCTGCAGGGCCAGTCAAAGTATTAGCAACTACCTACACTGGTATCAACAAAAATCACATGAGTCTCCAAGGCTTCTCATCAAGTATGCTTCCCAGTCCATCTCTGGGATCCCCTCCAGGTTCAGTGGCAGTGGATCAGGGACAGATTTCACTCTCAGTATCAACAGTGTGGAGACTGAAGATTTTGGAATGTATTTCTGTCAACAGAGTAACAGCTGGCCTCA
IGKV5-45*01_B6	C57BL/6J	IGKV	MUSMUS IGKV5-45*01 F	100	GATATTGTGCTAACTCAGTCTCCAGCCACCCTGTCTGTGACTCCAGGAGATAGAGTCAGTCTTTCCTGCAGGGCCAGTCAAAGTATTAGCAACTACCTACACTGGTATCAACAAAAATCACATGAGTCTCCAAGGCTTCTCATCAAGTATGCTTCCCAGTCCATCTCTGGGATCCCCTCCAGGTTCAGTGGCAGTGGATCAGGGACAGATTTCACTCTCAGTATCAACAGTGTGGAGACTGAAGATTTTGGAATGTATTTCTGTCAACAGAGTAACAGCTGGCCTCA
IGKV5-45*01_BALB	BALB/CBYJ	IGKV	MUSMUS IGKV5-45*01 F	100	GATATTGTGCTAACTCAGTCTCCAGCCACCCTGTCTGTGACTCCAGGAGATAGAGTCAGTCTTTCCTGCAGGGCCAGTCAAAGTATTAGCAACTACCTACACTGGTATCAACAAAAATCACATGAGTCTCCAAGGCTTCTCATCAAGTATGCTTCCCAGTCCATCTCTGGGATCCCCTCCAGGTTCAGTGGCAGTGGATCAGGGACAGATTTCACTCTCAGTATCAACAGTGTGGAGACTGAAGATTTTGGAATGTATTTCTGTCAACAGAGTAACAGCTGGCCTCA

IGKV5-45*01_C3H	C3H/HEJ	IGKV	MUSMUS IGKV5-45*01 F	100	GATATTGTGCTAACTCAGTCTCCAGCCACCCTGTCTGTGACTCCAGGAGATAGAGTCAGTCTTTCCTGCAGGGCCAGTCAAAGTATTAGCAACTACCTACACTGGTATCAACAAAAATCACATGAGTCTCCAAGGCTTCTCATCAAGTATGCTTCCCAGTCCATCTCTGGGATCCCCTCCAGGTTCAGTGGCAGTGGATCAGGGACAGATTTCACTCTCAGTATCAACAGTGTGGAGACTGAAGATTTTGGAATGTATTTCTGTCAACAGAGTAACAGCTGGCCTCA
IGKV5-45*01_CBA	CBA/J	IGKV	MUSMUS IGKV5-45*01 F	100	GATATTGTGCTAACTCAGTCTCCAGCCACCCTGTCTGTGACTCCAGGAGATAGAGTCAGTCTTTCCTGCAGGGCCAGTCAAAGTATTAGCAACTACCTACACTGGTATCAACAAAAATCACATGAGTCTCCAAGGCTTCTCATCAAGTATGCTTCCCAGTCCATCTCTGGGATCCCCTCCAGGTTCAGTGGCAGTGGATCAGGGACAGATTTCACTCTCAGTATCAACAGTGTGGAGACTGAAGATTTTGGAATGTATTTCTGTCAACAGAGTAACAGCTGGCCTCA
IGKV5-45*01_DBA1	DBA/1J	IGKV	MUSMUS IGKV5-45*01 F	100	GATATTGTGCTAACTCAGTCTCCAGCCACCCTGTCTGTGACTCCAGGAGATAGAGTCAGTCTTTCCTGCAGGGCCAGTCAAAGTATTAGCAACTACCTACACTGGTATCAACAAAAATCACATGAGTCTCCAAGGCTTCTCATCAAGTATGCTTCCCAGTCCATCTCTGGGATCCCCTCCAGGTTCAGTGGCAGTGGATCAGGGACAGATTTCACTCTCAGTATCAACAGTGTGGAGACTGAAGATTTTGGAATGTATTTCTGTCAACAGAGTAACAGCTGGCCTCA
IGKV5-45*01_DBA2	DBA/2J	IGKV	MUSMUS IGKV5-45*01 F	100	GATATTGTGCTAACTCAGTCTCCAGCCACCCTGTCTGTGACTCCAGGAGATAGAGTCAGTCTTTCCTGCAGGGCCAGTCAAAGTATTAGCAACTACCTACACTGGTATCAACAAAAATCACATGAGTCTCCAAGGCTTCTCATCAAGTATGCTTCCCAGTCCATCTCTGGGATCCCCTCCAGGTTCAGTGGCAGTGGATCAGGGACAGATTTCACTCTCAGTATCAACAGTGTGGAGACTGAAGATTTTGGAATGTATTTCTGTCAACAGAGTAACAGCTGGCCTCA

IGKV5-45*01_LEWES	LEWES/EIJ	IGKV	MUSMUS IGKV5-45*01 F	100	GATATTGTGCTAACTCAGTCTCCAGCCACCCTGTCTGTGACTCCAGGAGATAGAGTCAGTCTTTCCTGCAGGGCCAGTCAAAGTATTAGCAACTACCTACACTGGTATCAACAAAAATCACATGAGTCTCCAAGGCTTCTCATCAAGTATGCTTCCCAGTCCATCTCTGGGATCCCCTCCAGGTTCAGTGGCAGTGGATCAGGGACAGATTTCACTCTCAGTATCAACAGTGTGGAGACTGAAGATTTTGGAATGTATTTCTGTCAACAGAGTAACAGCTGGCCTCA
IGKV5-45*01_NZB	NZB/BLNJ	IGKV	MUSMUS IGKV5-45*01 F	100	GATATTGTGCTAACTCAGTCTCCAGCCACCCTGTCTGTGACTCCAGGAGATAGAGTCAGTCTTTCCTGCAGGGCCAGTCAAAGTATTAGCAACTACCTACACTGGTATCAACAAAAATCACATGAGTCTCCAAGGCTTCTCATCAAGTATGCTTCCCAGTCCATCTCTGGGATCCCCTCCAGGTTCAGTGGCAGTGGATCAGGGACAGATTTCACTCTCAGTATCAACAGTGTGGAGACTGAAGATTTTGGAATGTATTTCTGTCAACAGAGTAACAGCTGGCCTCA
IGKV5-45*01_S0430_AKR	AKR/J	IGKV	MUSMUS IGKV5-45*01 F	99.64	GATATTGTGCTAACTCAGTCTCCAGCCACCCTGTCTGTGACTCCAGGAGATAGAGTCAGTCTTTCCTGCAGGGCCAGCCAAAGTATTAGCAACTACCTACACTGGTATCAACAAAAATCACATGAGTCTCCAAGGCTTCTCATCAAGTATGCTTCCCAGTCCATCTCTGGGATCCCCTCCAGGTTCAGTGGCAGTGGATCAGGGACAGATTTCACTCTCAGTATCAACAGTGTGGAGACTGAAGATTTTGGAATGTATTTCTGTCAACAGAGTAACAGCTGGCCTCA
IGKV5-45*01_S0430_MRL	MRL/MPJ	IGKV	MUSMUS IGKV5-45*01 F	99.64	GATATTGTGCTAACTCAGTCTCCAGCCACCCTGTCTGTGACTCCAGGAGATAGAGTCAGTCTTTCCTGCAGGGCCAGCCAAAGTATTAGCAACTACCTACACTGGTATCAACAAAAATCACATGAGTCTCCAAGGCTTCTCATCAAGTATGCTTCCCAGTCCATCTCTGGGATCCCCTCCAGGTTCAGTGGCAGTGGATCAGGGACAGATTTCACTCTCAGTATCAACAGTGTGGAGACTGAAGATTTTGGAATGTATTTCTGTCAACAGAGTAACAGCTGGCCTCA

IGKV5-45*01_S0430_NOD	NOD/SHILTJ	IGKV	MUSMUS IGKV5-45*01 F	99.64	GATATTGTGCTAACTCAGTCTCCAGCCACCCTGTCTGTGACTCCAGGAGATAGAGTCAGTCTTTCCTGCAGGGCCAGCCAAAGTATTAGCAACTACCTACACTGGTATCAACAAAAATCACATGAGTCTCCAAGGCTTCTCATCAAGTATGCTTCCCAGTCCATCTCTGGGATCCCCTCCAGGTTCAGTGGCAGTGGATCAGGGACAGATTTCACTCTCAGTATCAACAGTGTGGAGACTGAAGATTTTGGAATGTATTTCTGTCAACAGAGTAACAGCTGGCCTCA
IGKV5-45*01_S0430_NOR	NOR/LTJ	IGKV	MUSMUS IGKV5-45*01 F	99.64	GATATTGTGCTAACTCAGTCTCCAGCCACCCTGTCTGTGACTCCAGGAGATAGAGTCAGTCTTTCCTGCAGGGCCAGCCAAAGTATTAGCAACTACCTACACTGGTATCAACAAAAATCACATGAGTCTCCAAGGCTTCTCATCAAGTATGCTTCCCAGTCCATCTCTGGGATCCCCTCCAGGTTCAGTGGCAGTGGATCAGGGACAGATTTCACTCTCAGTATCAACAGTGTGGAGACTGAAGATTTTGGAATGTATTTCTGTCAACAGAGTAACAGCTGGCCTCA
IGKV5-45*01_S0430_PWD	PWD/PHJ	IGKV	MUSMUS IGKV5-45*01 F	99.64	GATATTGTGCTAACTCAGTCTCCAGCCACCCTGTCTGTGACTCCAGGAGATAGAGTCAGTCTTTCCTGCAGGGCCAGCCAAAGTATTAGCAACTACCTACACTGGTATCAACAAAAATCACATGAGTCTCCAAGGCTTCTCATCAAGTATGCTTCCCAGTCCATCTCTGGGATCCCCTCCAGGTTCAGTGGCAGTGGATCAGGGACAGATTTCACTCTCAGTATCAACAGTGTGGAGACTGAAGATTTTGGAATGTATTTCTGTCAACAGAGTAACAGCTGGCCTCA
IGKV5-45*01_S2588_MSM	MSM/MSJ	IGKV	MUSMUS IGKV5-45*01 F	98.92	GATATTGTGCTAACTCAGTCTCCAGCCACCCTGTCTGTGACTCCAGGAGATAGAGTCAGTCTTTCCTGCAGGGCCAGCCAAAGTATTAGCAACTACCTACACAGGTATCAACAAAAATCACATGAGTCTCCAAGGCTTCTCATCAAGTATGCTTCCCAGTCCATCTCTGGGATCCCCTCCAGGTTCAGTGGCAGTGGATCAGGGACAGATTTCACTCTCAGTATCAACAGTGTGGAGACTGAAGATTTTGGAATGTATTTTTGTCAACAGAGTAACAGCTGGCCTCA

IGKV5-48*01_129	129S1/SVIMJ	IGKV	MUSMUS IGKV5-48*01 F	100	GACATCTTGCTGACTCAGTCTCCAGCCATCCTGTCTGTGAGTCCAGGAGAAAGAGTCAGTTTCTCCTGCAGGGCCAGTCAGAGCATTGGCACAAGCATACACTGGTATCAGCAAAGAACAAATGGTTCTCCAAGGCTTCTCATAAAGTATGCTTCTGAGTCTATCTCTGGGATCCCTTCCAGGTTTAGTGGCAGTGGATCAGGGACAGATTTTACTCTTAGCATCAACAGTGTGGAGTCTGAAGATATTGCAGATTATTACTGTCAACAAAGTAATAGCTGGCCAAC
IGKV5-48*01_AJ	A/J	IGKV	MUSMUS IGKV5-48*01 F	100	GACATCTTGCTGACTCAGTCTCCAGCCATCCTGTCTGTGAGTCCAGGAGAAAGAGTCAGTTTCTCCTGCAGGGCCAGTCAGAGCATTGGCACAAGCATACACTGGTATCAGCAAAGAACAAATGGTTCTCCAAGGCTTCTCATAAAGTATGCTTCTGAGTCTATCTCTGGGATCCCTTCCAGGTTTAGTGGCAGTGGATCAGGGACAGATTTTACTCTTAGCATCAACAGTGTGGAGTCTGAAGATATTGCAGATTATTACTGTCAACAAAGTAATAGCTGGCCAAC
IGKV5-48*01_B6	C57BL/6J	IGKV	MUSMUS IGKV5-48*01 F	100	GACATCTTGCTGACTCAGTCTCCAGCCATCCTGTCTGTGAGTCCAGGAGAAAGAGTCAGTTTCTCCTGCAGGGCCAGTCAGAGCATTGGCACAAGCATACACTGGTATCAGCAAAGAACAAATGGTTCTCCAAGGCTTCTCATAAAGTATGCTTCTGAGTCTATCTCTGGGATCCCTTCCAGGTTTAGTGGCAGTGGATCAGGGACAGATTTTACTCTTAGCATCAACAGTGTGGAGTCTGAAGATATTGCAGATTATTACTGTCAACAAAGTAATAGCTGGCCAAC
IGKV5-48*01_BALB	BALB/CBYJ	IGKV	MUSMUS IGKV5-48*01 F	100	GACATCTTGCTGACTCAGTCTCCAGCCATCCTGTCTGTGAGTCCAGGAGAAAGAGTCAGTTTCTCCTGCAGGGCCAGTCAGAGCATTGGCACAAGCATACACTGGTATCAGCAAAGAACAAATGGTTCTCCAAGGCTTCTCATAAAGTATGCTTCTGAGTCTATCTCTGGGATCCCTTCCAGGTTTAGTGGCAGTGGATCAGGGACAGATTTTACTCTTAGCATCAACAGTGTGGAGTCTGAAGATATTGCAGATTATTACTGTCAACAAAGTAATAGCTGGCCAAC

IGKV5-48*01_C3H	C3H/HEJ	IGKV	MUSMUS IGKV5-48*01 F	100	GACATCTTGCTGACTCAGTCTC<br>CAGCCATCCTGTCTGTGAGTCC<br>AGGAGAAAGAGTCAGTTTCTCC<br>TGCAGGGCCAGTCAGAGCATTG<br>GCACAAGCATACACTGGTATCA<br>GCAAAGAACAAATGGTTCTCCA<br>AGGCTTCTCATAAAGTATGCTTC<br>TGAGTCTATCTCTGGGATCCCTT<br>CCAGGTTTAGTGGCAGTGGATC<br>AGGGACAGATTTTACTCTTAGCA<br>TCAACAGTGTGGAGTCTGAAGA<br>TATTGCAGATTATTACTGTCAACA<br>AAGTAATAGCTGGCCAAC
IGKV5-48*01_CBA	CBA/J	IGKV	MUSMUS IGKV5-48*01 F	100	GACATCTTGCTGACTCAGTCTC<br>CAGCCATCCTGTCTGTGAGTCC<br>AGGAGAAAGAGTCAGTTTCTCC<br>TGCAGGGCCAGTCAGAGCATTG<br>GCACAAGCATACACTGGTATCA<br>GCAAAGAACAAATGGTTCTCCA<br>AGGCTTCTCATAAAGTATGCTTC<br>TGAGTCTATCTCTGGGATCCCTT<br>CCAGGTTTAGTGGCAGTGGATC<br>AGGGACAGATTTTACTCTTAGCA<br>TCAACAGTGTGGAGTCTGAAGA<br>TATTGCAGATTATTACTGTCAACA<br>AAGTAATAGCTGGCCAAC
IGKV5-48*01_DBA1	DBA/1J	IGKV	MUSMUS IGKV5-48*01 F	100	GACATCTTGCTGACTCAGTCTC<br>CAGCCATCCTGTCTGTGAGTCC<br>AGGAGAAAGAGTCAGTTTCTCC<br>TGCAGGGCCAGTCAGAGCATTG<br>GCACAAGCATACACTGGTATCA<br>GCAAAGAACAAATGGTTCTCCA<br>AGGCTTCTCATAAAGTATGCTTC<br>TGAGTCTATCTCTGGGATCCCTT<br>CCAGGTTTAGTGGCAGTGGATC<br>AGGGACAGATTTTACTCTTAGCA<br>TCAACAGTGTGGAGTCTGAAGA<br>TATTGCAGATTATTACTGTCAACA<br>AAGTAATAGCTGGCCAAC
IGKV5-48*01_DBA2	DBA/2J	IGKV	MUSMUS IGKV5-48*01 F	100	GACATCTTGCTGACTCAGTCTC<br>CAGCCATCCTGTCTGTGAGTCC<br>AGGAGAAAGAGTCAGTTTCTCC<br>TGCAGGGCCAGTCAGAGCATTG<br>GCACAAGCATACACTGGTATCA<br>GCAAAGAACAAATGGTTCTCCA<br>AGGCTTCTCATAAAGTATGCTTC<br>TGAGTCTATCTCTGGGATCCCTT<br>CCAGGTTTAGTGGCAGTGGATC<br>AGGGACAGATTTTACTCTTAGCA<br>TCAACAGTGTGGAGTCTGAAGA<br>TATTGCAGATTATTACTGTCAACA<br>AAGTAATAGCTGGCCAAC

IGKV5-48*01_LEWES	LEWES/EIJ	IGKV	MUSMUS IGKV5-48*01 F	100	GACATCTTGCTGACTCAGTCTC<br>CAGCCATCCTGTCTGTGAGTCC<br>AGGAGAAAGAGTCAGTTTCTCC<br>TGCAGGGCCAGTCAGAGCATTG<br>GCACAAGCATACACTGGTATCA<br>GCAAAGAACAAATGGTTCTCCA<br>AGGCTTCTCATAAAGTATGCTTC<br>TGAGTCTATCTCTGGGATCCCTT<br>CCAGGTTTAGTGGCAGTGGATC<br>AGGGACAGATTTTACTCTTAGCA<br>TCAACAGTGTGGAGTCTGAAGA<br>TATTGCAGATTATTACTGTCAACA<br>AAGTAATAGCTGGCCAAC
IGKV5-48*01_MSM	MSM/MSJ	IGKV	MUSMUS IGKV5-48*01 F	100	GACATCTTGCTGACTCAGTCTC<br>CAGCCATCCTGTCTGTGAGTCC<br>AGGAGAAAGAGTCAGTTTCTCC<br>TGCAGGGCCAGTCAGAGCATTG<br>GCACAAGCATACACTGGTATCA<br>GCAAAGAACAAATGGTTCTCCA<br>AGGCTTCTCATAAAGTATGCTTC<br>TGAGTCTATCTCTGGGATCCCTT<br>CCAGGTTTAGTGGCAGTGGATC<br>AGGGACAGATTTTACTCTTAGCA<br>TCAACAGTGTGGAGTCTGAAGA<br>TATTGCAGATTATTACTGTCAACA<br>AAGTAATAGCTGGCCAAC
IGKV5-48*01_NZB	NZB/BLNJ	IGKV	MUSMUS IGKV5-48*01 F	100	GACATCTTGCTGACTCAGTCTC<br>CAGCCATCCTGTCTGTGAGTCC<br>AGGAGAAAGAGTCAGTTTCTCC<br>TGCAGGGCCAGTCAGAGCATTG<br>GCACAAGCATACACTGGTATCA<br>GCAAAGAACAAATGGTTCTCCA<br>AGGCTTCTCATAAAGTATGCTTC<br>TGAGTCTATCTCTGGGATCCCTT<br>CCAGGTTTAGTGGCAGTGGATC<br>AGGGACAGATTTTACTCTTAGCA<br>TCAACAGTGTGGAGTCTGAAGA<br>TATTGCAGATTATTACTGTCAACA<br>AAGTAATAGCTGGCCAAC
IGKV5-48*01_S4608_CAST	CAST/EIJ	IGKV	MUSMUS IGKV5-48*01 F	97.13	GATATCTTGCTGACTCAGTCTCC<br>AGCCATCCTGTCTGTGACTCTA<br>GGAGAAAGAGTCAGTTTCTCCT<br>GCAGGGCCAGTCAGAGCATTG<br>GCACAAGCATACACTGGTATCA<br>GCAAAGAACAAATGGTTCTCCA<br>AGGCTTCTCATAAAGTATGCTTC<br>TGAGTCTATCTCTGGGATCCCAT<br>CCAGGTTTAGTGGCAGTGGATC<br>AGGGACAGATTTCACTCTTAGCA<br>TCAACAGTGTGGAGTCTGAAGA<br>TGTTGCAGATTATTACTGTCAAC<br>AGAGTTATAGCTGGCCAAC

IGKV5-48*01_S4867_PWD	PWD/PHJ	IGKV	MUSMUS IGKV5-48*01 F	99.64	GACATCTTGCTGACTCAGTCTCCAGCCATCCTGTCTGTGAGTCCAGGAGAAAGAGTCAGTTTCTCCTGCAGGGCCAGTCAGAGCATTGGCACAAGCATACACTGGTATCAGCAAAGAACAAATGGTTCTCCAAGGCTTCTCATAAAGTATGCTTCTGAGTCTATCTCTGGGATCCCTTCCAGGTTTAGTGGCAGTGGATCAGGGTCAGATTTTACTCTTAGCATCAACAGTGTGGAGTCTGAAGATATTGCAGATTATTACTGTCAACAAAGTAATAGCTGGCCAAC
IGKV5-48*01_SJL	SJL/J	IGKV	MUSMUS IGKV5-48*01 F	100	GACATCTTGCTGACTCAGTCTCCAGCCATCCTGTCTGTGAGTCCAGGAGAAAGAGTCAGTTTCTCCTGCAGGGCCAGTCAGAGCATTGGCACAAGCATACACTGGTATCAGCAAAGAACAAATGGTTCTCCAAGGCTTCTCATAAAGTATGCTTCTGAGTCTATCTCTGGGATCCCTTCCAGGTTTAGTGGCAGTGGATCAGGGACAGATTTTACTCTTAGCATCAACAGTGTGGAGTCTGAAGATATTGCAGATTATTACTGTCAACAAAGTAATAGCTGGCCAAC
IGKV6-13*01_129	129S1/SVIMJ	IGKV	MUSMUS IGKV6-13*01 F	100	GACATTGTGATGACCCAGTCTCAAAAATTCATGTCCACATCAGTAGGAGACAGGGTCAGCATCACCTGCAAGGCCAGTCAGAATGTGGGTACTGCTGTAGCCTGGTATCAACAGAAACCAGGACAATCTCCTAAACTACTGATTTACTCGGCATCCAATCGGTACACTGGAGTCCCTGATCGCTTCACAGGCAGTGGATCTGGGACAGATTTCACTCTCACCATCAGCAATATGCAGTCTGAAGACCTGGCAGATTATTTCTGCCAGCAATATAGCAGCTATCCT
IGKV6-13*01_AJ	A/J	IGKV	MUSMUS IGKV6-13*01 F	100	GACATTGTGATGACCCAGTCTCAAAAATTCATGTCCACATCAGTAGGAGACAGGGTCAGCATCACCTGCAAGGCCAGTCAGAATGTGGGTACTGCTGTAGCCTGGTATCAACAGAAACCAGGACAATCTCCTAAACTACTGATTTACTCGGCATCCAATCGGTACACTGGAGTCCCTGATCGCTTCACAGGCAGTGGATCTGGGACAGATTTCACTCTCACCATCAGCAATATGCAGTCTGAAGACCTGGCAGATTATTTCTGCCAGCAATATAGCAGCTATCCT

IGKV6-13*01_B6	C57BL/6J	IGKV	MUSMUS IGKV6-13*01 F	100	GACATTGTGATGACCCAGTCTCAAAAATTCATGTCCACATCAGTAGGAGACAGGGTCAGCATCACCTGCAAGGCCAGTCAGAATGTGGGTACTGCTGTAGCCTGGTATCAACAGAAACCAGGACAATCTCCTAAACTACTGATTTACTCGGCATCCAATCGGTACACTGGAGTCCCTGATCGCTTCACAGGCAGTGGATCTGGGACAGATTTCACTCTCACCATCAGCAATATGCAGTCTGAAGACCTGGCAGATTATTTCTGCCAGCAATATAGCAGCTATCCT
IGKV6-13*01_BALB	BALB/CBYJ	IGKV	MUSMUS IGKV6-13*01 F	100	GACATTGTGATGACCCAGTCTCAAAAATTCATGTCCACATCAGTAGGAGACAGGGTCAGCATCACCTGCAAGGCCAGTCAGAATGTGGGTACTGCTGTAGCCTGGTATCAACAGAAACCAGGACAATCTCCTAAACTACTGATTTACTCGGCATCCAATCGGTACACTGGAGTCCCTGATCGCTTCACAGGCAGTGGATCTGGGACAGATTTCACTCTCACCATCAGCAATATGCAGTCTGAAGACCTGGCAGATTATTTCTGCCAGCAATATAGCAGCTATCCT
IGKV6-13*01_C3H	C3H/HEJ	IGKV	MUSMUS IGKV6-13*01 F	100	GACATTGTGATGACCCAGTCTCAAAAATTCATGTCCACATCAGTAGGAGACAGGGTCAGCATCACCTGCAAGGCCAGTCAGAATGTGGGTACTGCTGTAGCCTGGTATCAACAGAAACCAGGACAATCTCCTAAACTACTGATTTACTCGGCATCCAATCGGTACACTGGAGTCCCTGATCGCTTCACAGGCAGTGGATCTGGGACAGATTTCACTCTCACCATCAGCAATATGCAGTCTGAAGACCTGGCAGATTATTTCTGCCAGCAATATAGCAGCTATCCT
IGKV6-13*01_CBA	CBA/J	IGKV	MUSMUS IGKV6-13*01 F	100	GACATTGTGATGACCCAGTCTCAAAAATTCATGTCCACATCAGTAGGAGACAGGGTCAGCATCACCTGCAAGGCCAGTCAGAATGTGGGTACTGCTGTAGCCTGGTATCAACAGAAACCAGGACAATCTCCTAAACTACTGATTTACTCGGCATCCAATCGGTACACTGGAGTCCCTGATCGCTTCACAGGCAGTGGATCTGGGACAGATTTCACTCTCACCATCAGCAATATGCAGTCTGAAGACCTGGCAGATTATTTCTGCCAGCAATATAGCAGCTATCCT

IGKV6-13*01_DBA1	DBA/1J	IGKV	MUSMUS IGKV6-13*01 F	100	GACATTGTGATGACCCAGTCTC<br>AAAAATTCATGTCCACATCAGTA<br>GGAGACAGGGTCAGCATCACC<br>TGCAAGGCCAGTCAGAATGTGG<br>GTACTGCTGTAGCCTGGTATCA<br>ACAGAAACCAGGACAATCTCCT<br>AAACTACTGATTTACTCGGCATC<br>CAATCGGTACACTGGAGTCCCT<br>GATCGCTTCACAGGCAGTGGAT<br>CTGGGACAGATTTCACTCTCAC<br>CATCAGCAATATGCAGTCTGAA<br>GACCTGGCAGATTATTTCTGCCA<br>GCAATATAGCAGCTATCCT
IGKV6-13*01_DBA2	DBA/2J	IGKV	MUSMUS IGKV6-13*01 F	100	GACATTGTGATGACCCAGTCTC<br>AAAAATTCATGTCCACATCAGTA<br>GGAGACAGGGTCAGCATCACC<br>TGCAAGGCCAGTCAGAATGTGG<br>GTACTGCTGTAGCCTGGTATCA<br>ACAGAAACCAGGACAATCTCCT<br>AAACTACTGATTTACTCGGCATC<br>CAATCGGTACACTGGAGTCCCT<br>GATCGCTTCACAGGCAGTGGAT<br>CTGGGACAGATTTCACTCTCAC<br>CATCAGCAATATGCAGTCTGAA<br>GACCTGGCAGATTATTTCTGCCA<br>GCAATATAGCAGCTATCCT
IGKV6-13*01_LEWES	LEWES/EIJ	IGKV	MUSMUS IGKV6-13*01 F	100	GACATTGTGATGACCCAGTCTC<br>AAAAATTCATGTCCACATCAGTA<br>GGAGACAGGGTCAGCATCACC<br>TGCAAGGCCAGTCAGAATGTGG<br>GTACTGCTGTAGCCTGGTATCA<br>ACAGAAACCAGGACAATCTCCT<br>AAACTACTGATTTACTCGGCATC<br>CAATCGGTACACTGGAGTCCCT<br>GATCGCTTCACAGGCAGTGGAT<br>CTGGGACAGATTTCACTCTCAC<br>CATCAGCAATATGCAGTCTGAA<br>GACCTGGCAGATTATTTCTGCCA<br>GCAATATAGCAGCTATCCT
IGKV6-13*01_NZB	NZB/BLNJ	IGKV	MUSMUS IGKV6-13*01 F	100	GACATTGTGATGACCCAGTCTC<br>AAAAATTCATGTCCACATCAGTA<br>GGAGACAGGGTCAGCATCACC<br>TGCAAGGCCAGTCAGAATGTGG<br>GTACTGCTGTAGCCTGGTATCA<br>ACAGAAACCAGGACAATCTCCT<br>AAACTACTGATTTACTCGGCATC<br>CAATCGGTACACTGGAGTCCCT<br>GATCGCTTCACAGGCAGTGGAT<br>CTGGGACAGATTTCACTCTCAC<br>CATCAGCAATATGCAGTCTGAA<br>GACCTGGCAGATTATTTCTGCCA<br>GCAATATAGCAGCTATCCT

IGKV6-13*01_S1558_PWD	PWD/PHJ	IGKV	MUSMUS IGKV6-13*01 F	99.64	GACATTGTGATGACCCAGTCTC<br>AAAAATTCATGTCCACATCAGTA<br>GGAGACAGGGTCAGCGTCACC<br>TGCAAGGCCAGTCAGAATGTGG<br>GTACTGCTGTAGCCTGGTATCA<br>ACAGAAACCAGGACAATCTCCT<br>AAACTACTGATTTACTCGGCATC<br>CAATCGGTACACTGGAGTCCCT<br>GATCGCTTCACAGGCAGTGGAT<br>CTGGGACAGATTTCACTCTCAC<br>CATCAGCAATATGCAGTCTGAA<br>GACCTGGCAGATTATTTCTGCCA<br>GCAATATAGCAGCTATCCT
IGKV6-13*01_S4184_PWD	PWD/PHJ	IGKV	MUSMUS IGKV6-13*01 F	89.61	GACATTGTGATGACCCAGTCAC<br>AAAAATTCATGTCCACATCAGTA<br>GGAGAGAGGGTCAGCATCAGC<br>TGCAAGGCCAGTCAGAATGTGG<br>GTAATATTATAGCCTGGTATCAA<br>CAGAAACCAGGACAGTCTCCTA<br>AAGCACTGATTTACTGGGCTTCC<br>AATCGGCACACTGGAGTCCCTG<br>ATCGCTTCACAGGCAGTGGATC<br>TGGGACAGATTTCACTCTGACC<br>ATCAGCAGTGTGCAGGCTGAAG<br>ACCTTGCAGATTATCACTGTGGA<br>CAGAGTTACAGCTATCCT
IGKV6-13*01_S5013_MSM	MSM/MSJ	IGKV	MUSMUS IGKV6-13*01 F	99.28	GACATTGTGATGCCCCAGTCTC<br>AAAAATTCATGTCCACATCAGTA<br>GGAGACAGGGTCAGCGTCACC<br>TGCAAGGCCAGTCAGAATGTGG<br>GTACTGCTGTAGCCTGGTATCA<br>ACAGAAACCAGGACAATCTCCT<br>AAACTACTGATTTACTCGGCATC<br>CAATCGGTACACTGGAGTCCCT<br>GATCGCTTCACAGGCAGTGGAT<br>CTGGGACAGATTTCACTCTCAC<br>CATCAGCAATATGCAGTCTGAA<br>GACCTGGCAGATTATTTCTGCCA<br>GCAATATAGCAGCTATCCT
IGKV6-13*01_S9207_SJL	SJL/J	IGKV	MUSMUS IGKV6-13*01 F	98.57	GACATTGTGATGACCCAGTCTC<br>AAAAATTCATGTCCACAACAGTA<br>GGAGACAGGGTCAGCATCACC<br>TGCAAGGCCAGTCAGAATGTGG<br>GTACTGCTGTAGCCTGGTATCA<br>ACAGAAACCAGGACAATCTCCT<br>AAACTACTGATTTACTCAGCATC<br>CAATCGGTACACTGGAGTCCCT<br>GATCGCTTCACAGGCAGTGGAT<br>CTGGGACAGATTTCACTCTCAC<br>CATTAGCAATATGCAGTCTGAAG<br>ACCTGGCAGATTATTTCTGTCAG<br>CAATATAGCAGCTATCCT

IGKV6-14*01_129	129S1/SVIMJ	IGKV	MUSMUS IGKV6-14*01 F	100	GACATTGTGATGACCCAGTCTCAAAAATTCATGTCCACATCAGTAGGAGACAGGGTCAGCATCACCTGCAAGGCCAGTCAGAATGTTCGTACTGCTGTAGCCTGGTATCAACAGAAACCAGGGCAGTCTCCTAAAGCACTGATTTACTTGGCATCCAACCGGCACACTGGAGTCCCTGATCGCTTCACAGGCAGTGGATCTGGGACAGATTTCACTCTCACCATTAGCAATGTGCAATCTGAAGACCTGGCAGATTATTTCTGTCTGCAACATTGGAATTATCCTCT
IGKV6-14*01_AJ	A/J	IGKV	MUSMUS IGKV6-14*01 F	100	GACATTGTGATGACCCAGTCTCAAAAATTCATGTCCACATCAGTAGGAGACAGGGTCAGCATCACCTGCAAGGCCAGTCAGAATGTTCGTACTGCTGTAGCCTGGTATCAACAGAAACCAGGGCAGTCTCCTAAAGCACTGATTTACTTGGCATCCAACCGGCACACTGGAGTCCCTGATCGCTTCACAGGCAGTGGATCTGGGACAGATTTCACTCTCACCATTAGCAATGTGCAATCTGAAGACCTGGCAGATTATTTCTGTCTGCAACATTGGAATTATCCTCT
IGKV6-14*01_BALB	BALB/CBYJ	IGKV	MUSMUS IGKV6-14*01 F	100	GACATTGTGATGACCCAGTCTCAAAAATTCATGTCCACATCAGTAGGAGACAGGGTCAGCATCACCTGCAAGGCCAGTCAGAATGTTCGTACTGCTGTAGCCTGGTATCAACAGAAACCAGGGCAGTCTCCTAAAGCACTGATTTACTTGGCATCCAACCGGCACACTGGAGTCCCTGATCGCTTCACAGGCAGTGGATCTGGGACAGATTTCACTCTCACCATTAGCAATGTGCAATCTGAAGACCTGGCAGATTATTTCTGTCTGCAACATTGGAATTATCCTCT
IGKV6-14*01_C3H	C3H/HEJ	IGKV	MUSMUS IGKV6-14*01 F	100	GACATTGTGATGACCCAGTCTCAAAAATTCATGTCCACATCAGTAGGAGACAGGGTCAGCATCACCTGCAAGGCCAGTCAGAATGTTCGTACTGCTGTAGCCTGGTATCAACAGAAACCAGGGCAGTCTCCTAAAGCACTGATTTACTTGGCATCCAACCGGCACACTGGAGTCCCTGATCGCTTCACAGGCAGTGGATCTGGGACAGATTTCACTCTCACCATTAGCAATGTGCAATCTGAAGACCTGGCAGATTATTTCTGTCTGCAACATTGGAATTATCCTCT

IGKV6-14*01_DBA1	DBA/1J	IGKV	MUSMUS IGKV6-14*01 F	100	GACATTGTGATGACCCAGTCTCAAAAATTCATGTCCACATCAGTAGGAGACAGGGTCAGCATCACCTGCAAGGCCAGTCAGAATGTTCGTACTGCTGTAGCCTGGTATCAACAGAAACCAGGGCAGTCTCCTAAAGCACTGATTTACTTGGCATCCAACCGGCACACTGGAGTCCCTGATCGCTTCACAGGCAGTGGATCTGGGACAGATTTCACTCTCACCATTAGCAATGTGCAATCTGAAGACCTGGCAGATTATTTCTGTCTGCAACATTGGAATTATCCTCT
IGKV6-14*01_DBA2	DBA/2J	IGKV	MUSMUS IGKV6-14*01 F	100	GACATTGTGATGACCCAGTCTCAAAAATTCATGTCCACATCAGTAGGAGACAGGGTCAGCATCACCTGCAAGGCCAGTCAGAATGTTCGTACTGCTGTAGCCTGGTATCAACAGAAACCAGGGCAGTCTCCTAAAGCACTGATTTACTTGGCATCCAACCGGCACACTGGAGTCCCTGATCGCTTCACAGGCAGTGGATCTGGGACAGATTTCACTCTCACCATTAGCAATGTGCAATCTGAAGACCTGGCAGATTATTTCTGTCTGCAACATTGGAATTATCCTCT
IGKV6-14*01_NZB	NZB/BLNJ	IGKV	MUSMUS IGKV6-14*01 F	100	GACATTGTGATGACCCAGTCTCAAAAATTCATGTCCACATCAGTAGGAGACAGGGTCAGCATCACCTGCAAGGCCAGTCAGAATGTTCGTACTGCTGTAGCCTGGTATCAACAGAAACCAGGGCAGTCTCCTAAAGCACTGATTTACTTGGCATCCAACCGGCACACTGGAGTCCCTGATCGCTTCACAGGCAGTGGATCTGGGACAGATTTCACTCTCACCATTAGCAATGTGCAATCTGAAGACCTGGCAGATTATTTCTGTCTGCAACATTGGAATTATCCTCT
IGKV6-14*01_S4666_PWD	PWD/PHJ	IGKV	MUSMUS IGKV6-14*01 F	96.42	GACATTGTGATGACCCAGTCTCAAAAATTCATGTCCACATCAGTAGAAGACAGGGTCAGCATCACCTGCAAGGCCAGTCAGAATGTAGGTACTGCTGTAGCCTGGTATCAACAGAAACCAGGGCAGTCTCCTAAACTACTGATTTACTTGGCATCCAATCGGCACACTGGAGTCCCTGATCGCTTCACAGGCAGTGGATCTGGGACAGATTTCACTCTCACCATCAGCAATGTGCAGTCTGAAGACCTGGCAGATTATTTTTGTCTGCAACATTGGAGTTATCCTCT

IGKV6-14*01_S5086_MSM	MSM/MSJ	IGKV	MUSMUS IGKV6-14*01 F	95.7	GACATTGTGATGACCCAGTCTC<br>AAAAATTCATGTCCACATCAGTA<br>GGAGAGAGGGTCATCATCACCT<br>GCAAGGCCAGTCAGAATGTGG<br>GTACTGCTGTAGCCTGGTATCA<br>ACAGAAACCAGGACAATCTCCT<br>AAAGCACTGATTTACTCGGCATC<br>CTACCGGTACACTGGAGTCCCT<br>GATCGCTTCACAGGCAGTGGAT<br>CTAGGACAGATTTCACTCTCAC<br>CATTAGCAATGTGCAGTCTGAA<br>GACCTGGCAGATTATTTCTGTCT<br>GCAACATTGGAGTTATCCTCT
IGKV6-15*01_129	129S1/SVIMJ	IGKV	MUSMUS IGKV6-15*01 F	100	GACATTGTGATGACCCAGTCTC<br>AAAAATTCATGTCCACATCAGTA<br>GGAGACAGGGTCAGCGTCACC<br>TGCAAGGCCAGTCAGAATGTGG<br>GTACTAATGTAGCCTGGTATCAA<br>CAGAAACCAGGGCAATCTCCTA<br>AAGCACTGATTTACTCGGCATC<br>CTACCGGTACAGTGGAGTCCCT<br>GATCGCTTCACAGGCAGTGGAT<br>CTGGGACAGATTTCACTCTCAC<br>CATCAGCAATGTGCAGTCTGAA<br>GACTTGGCAGAGTATTTCTGTCA<br>GCAATATAACAGCTATCCTCT
IGKV6-15*01_AJ	A/J	IGKV	MUSMUS IGKV6-15*01 F	100	GACATTGTGATGACCCAGTCTC<br>AAAAATTCATGTCCACATCAGTA<br>GGAGACAGGGTCAGCGTCACC<br>TGCAAGGCCAGTCAGAATGTGG<br>GTACTAATGTAGCCTGGTATCAA<br>CAGAAACCAGGGCAATCTCCTA<br>AAGCACTGATTTACTCGGCATC<br>CTACCGGTACAGTGGAGTCCCT<br>GATCGCTTCACAGGCAGTGGAT<br>CTGGGACAGATTTCACTCTCAC<br>CATCAGCAATGTGCAGTCTGAA<br>GACTTGGCAGAGTATTTCTGTCA<br>GCAATATAACAGCTATCCTCT
IGKV6-15*01_B6	C57BL/6J	IGKV	MUSMUS IGKV6-15*01 F	100	GACATTGTGATGACCCAGTCTC<br>AAAAATTCATGTCCACATCAGTA<br>GGAGACAGGGTCAGCGTCACC<br>TGCAAGGCCAGTCAGAATGTGG<br>GTACTAATGTAGCCTGGTATCAA<br>CAGAAACCAGGGCAATCTCCTA<br>AAGCACTGATTTACTCGGCATC<br>CTACCGGTACAGTGGAGTCCCT<br>GATCGCTTCACAGGCAGTGGAT<br>CTGGGACAGATTTCACTCTCAC<br>CATCAGCAATGTGCAGTCTGAA<br>GACTTGGCAGAGTATTTCTGTCA<br>GCAATATAACAGCTATCCTCT

IGKV6-15*01_BALB	BALB/CBYJ	IGKV	MUSMUS IGKV6-15*01 F	100	GACATTGTGATGACCCAGTCTCAAAAATTCATGTCCACATCAGTAGGAGACAGGGTCAGCGTCACCTGCAAGGCCAGTCAGAATGTGGGTACTAATGTAGCCTGGTATCAACAGAAACCAGGGCAATCTCCTAAAGCACTGATTTACTCGGCATCCTACCGGTACAGTGGAGTCCCTGATCGCTTCACAGGCAGTGGATCTGGGACAGATTTCACTCTCACCATCAGCAATGTGCAGTCTGAAGACTTGGCAGAGTATTTCTGTCAGCAATATAACAGCTATCCTCT
IGKV6-15*01_C3H	C3H/HEJ	IGKV	MUSMUS IGKV6-15*01 F	100	GACATTGTGATGACCCAGTCTCAAAAATTCATGTCCACATCAGTAGGAGACAGGGTCAGCGTCACCTGCAAGGCCAGTCAGAATGTGGGTACTAATGTAGCCTGGTATCAACAGAAACCAGGGCAATCTCCTAAAGCACTGATTTACTCGGCATCCTACCGGTACAGTGGAGTCCCTGATCGCTTCACAGGCAGTGGATCTGGGACAGATTTCACTCTCACCATCAGCAATGTGCAGTCTGAAGACTTGGCAGAGTATTTCTGTCAGCAATATAACAGCTATCCTCT
IGKV6-15*01_CBA	CBA/J	IGKV	MUSMUS IGKV6-15*01 F	100	GACATTGTGATGACCCAGTCTCAAAAATTCATGTCCACATCAGTAGGAGACAGGGTCAGCGTCACCTGCAAGGCCAGTCAGAATGTGGGTACTAATGTAGCCTGGTATCAACAGAAACCAGGGCAATCTCCTAAAGCACTGATTTACTCGGCATCCTACCGGTACAGTGGAGTCCCTGATCGCTTCACAGGCAGTGGATCTGGGACAGATTTCACTCTCACCATCAGCAATGTGCAGTCTGAAGACTTGGCAGAGTATTTCTGTCAGCAATATAACAGCTATCCTCT
IGKV6-15*01_DBA1	DBA/1J	IGKV	MUSMUS IGKV6-15*01 F	100	GACATTGTGATGACCCAGTCTCAAAAATTCATGTCCACATCAGTAGGAGACAGGGTCAGCGTCACCTGCAAGGCCAGTCAGAATGTGGGTACTAATGTAGCCTGGTATCAACAGAAACCAGGGCAATCTCCTAAAGCACTGATTTACTCGGCATCCTACCGGTACAGTGGAGTCCCTGATCGCTTCACAGGCAGTGGATCTGGGACAGATTTCACTCTCACCATCAGCAATGTGCAGTCTGAAGACTTGGCAGAGTATTTCTGTCAGCAATATAACAGCTATCCTCT

IGKV6-15*01_DBA2	DBA/2J	IGKV	MUSMUS IGKV6-15*01 F	100	GACATTGTGATGACCCAGTCTC<br>AAAAATTCATGTCCACATCAGTA<br>GGAGACAGGGTCAGCGTCACC<br>TGCAAGGCCAGTCAGAATGTGG<br>GTACTAATGTAGCCTGGTATCAA<br>CAGAAACCAGGGCAATCTCCTA<br>AAGCACTGATTTACTCGGCATC<br>CTACCGGTACAGTGGAGTCCCT<br>GATCGCTTCACAGGCAGTGGAT<br>CTGGGACAGATTTCACTCTCAC<br>CATCAGCAATGTGCAGTCTGAA<br>GACTTGGCAGAGTATTTCTGTCA<br>GCAATATAACAGCTATCCTCT
IGKV6-15*01_LEWES	LEWES/EIJ	IGKV	MUSMUS IGKV6-15*01 F	100	GACATTGTGATGACCCAGTCTC<br>AAAAATTCATGTCCACATCAGTA<br>GGAGACAGGGTCAGCGTCACC<br>TGCAAGGCCAGTCAGAATGTGG<br>GTACTAATGTAGCCTGGTATCAA<br>CAGAAACCAGGGCAATCTCCTA<br>AAGCACTGATTTACTCGGCATC<br>CTACCGGTACAGTGGAGTCCCT<br>GATCGCTTCACAGGCAGTGGAT<br>CTGGGACAGATTTCACTCTCAC<br>CATCAGCAATGTGCAGTCTGAA<br>GACTTGGCAGAGTATTTCTGTCA<br>GCAATATAACAGCTATCCTCT
IGKV6-15*01_NZB	NZB/BLNJ	IGKV	MUSMUS IGKV6-15*01 F	100	GACATTGTGATGACCCAGTCTC<br>AAAAATTCATGTCCACATCAGTA<br>GGAGACAGGGTCAGCGTCACC<br>TGCAAGGCCAGTCAGAATGTGG<br>GTACTAATGTAGCCTGGTATCAA<br>CAGAAACCAGGGCAATCTCCTA<br>AAGCACTGATTTACTCGGCATC<br>CTACCGGTACAGTGGAGTCCCT<br>GATCGCTTCACAGGCAGTGGAT<br>CTGGGACAGATTTCACTCTCAC<br>CATCAGCAATGTGCAGTCTGAA<br>GACTTGGCAGAGTATTTCTGTCA<br>GCAATATAACAGCTATCCTCT
IGKV6-15*01_S1998_PWD	PWD/PHJ	IGKV	MUSMUS IGKV6-15*01 F	96.42	GACATTGTGATGACCCAGTCTC<br>AAAAATTCATGTCCACATCAGTA<br>GGAGACAGGGTCAGCGTCACC<br>TGCAAGGCCAGTCAGAATGTGG<br>GTACTTATGTAGCCTGGTATCAA<br>CAGAAACCAGGGCAATCTCCTA<br>AACTACTGATTTACTCGGCATCC<br>AATCGGCACACTGGATTCCCTG<br>ATCGCTTCACAGGCAGTGGATC<br>TGGGACAGATTTCACTCTCACC<br>ATCAGCAATGTGCAGTCTGAAG<br>ACTTGGCAGAGTATTTCTGTGAG<br>CAATATAGCAGCTATCCTCT

IGKV6-15*01_S4042_CAST	CAST/EIJ	IGKV	MUSMUS IGKV6-15*01 F	97.13	GACATTGTGATGACCCAGTCTCAAAAATTCATGTCCACATCAGTAGGAGACAGGGTCAGCGTCACCTGCAAGGCCAGTCAGTATGTGGGTACTTATGTAGCCTGGTATCAACAGAAACCAGGGCAATCTCCTAAAGCACTGATTTACTCGGCATCCACCCGGCACACTGGAGTCCCTGATCGCTTCACAGGCAGTGGATCTGGGACAGATTTCACTCTCACCATTAGCAATGTGCAGTCTGAAGACTTGGCAGAGTATTTCTGTCAGCAATATAGCAGCTCTCCTCT
IGKV6-15*01_S8899_MSM	MSM/MSJ	IGKV	MUSMUS IGKV6-15*01 F	97.13	GACATTGTGATGACCCAGTCTCAAAAATTCATGTCCACATCAGTAGGAGACAGGGTCAGCGTCACCTGCAAGGCCAGTCAGTATGTGGGTACTTATGTAGCCTGGTATCAACAGAAACCAGGGCAATCTCCTAAAGCACTGATTTACTCGGCATCCTACCGGTACACTGGAGTCACTGATCGCTTCACAGGCAGTGGATCTGGGACAGATTTCACTCTCACCATTAGCAATGTGCAGTCTGAAGACTTAGCAGATTATTTCTGTCAGCAATATAGCAGCTCTCCTCT
IGKV6-15*01_S9094_AKR	AKR/J	IGKV	MUSMUS IGKV6-15*01 F	96.06	GACATTGTGATGACCCAGTCTCAAAAATTCATGTCCACATCAGTAGGAGACAGGGTCAGCGTCACCTGCAAGGCCAGTCAGTATGTGGGTACTTATGTAGCCTGGTATCAACAGAAACCAGGACAATCTCCTAAAGCACTGATTTACTCGGCATCCACCCGGCACACTGGAGTCCCTGATCGCTTCACAGGCAGTGGATCTGGGACAGATTTCACTCTCACCATTAGCAATGTGCAGTCTGAAGACTTGGCAGAGTATTTCTGTGAGCAATACAGCAGCTCTCCTCT
IGKV6-15*01_S9094_MRL	MRL/MPJ	IGKV	MUSMUS IGKV6-15*01 F	96.06	GACATTGTGATGACCCAGTCTCAAAAATTCATGTCCACATCAGTAGGAGACAGGGTCAGCGTCACCTGCAAGGCCAGTCAGTATGTGGGTACTTATGTAGCCTGGTATCAACAGAAACCAGGACAATCTCCTAAAGCACTGATTTACTCGGCATCCACCCGGCACACTGGAGTCCCTGATCGCTTCACAGGCAGTGGATCTGGGACAGATTTCACTCTCACCATTAGCAATGTGCAGTCTGAAGACTTGGCAGAGTATTTCTGTGAGCAATACAGCAGCTCTCCTCT

IGKV6-15*01_S9094_NOR	NOR/LTJ	IGKV	MUSMUS IGKV6-15*01 F	96.06	GACATTGTGATGACCCAGTCTC<br>AAAAATTCATGTCCACATCAGTA<br>GGAGACAGGGTCAGCGTCACC<br>TGCAAGGCCAGTCAGTATGTGG<br>GTACTTATGTAGCCTGGTATCAA<br>CAGAAACCAGGACAATCTCCTA<br>AAGCACTGATTTACTCGGCATC<br>CACCCGGCACACTGGAGTCCC<br>TGATCGCTTCACAGGCAGTGGA<br>TCTGGGACAGATTTCACTCTCAC<br>CATTAGCAATGTGCAGTCTGAA<br>GACTTGGCAGAGTATTTCTGTGA<br>GCAATACAGCAGCTCTCCTCT
IGKV6-15*01_S9094_PWD	PWD/PHJ	IGKV	MUSMUS IGKV6-15*01 F	96.06	GACATTGTGATGACCCAGTCTC<br>AAAAATTCATGTCCACATCAGTA<br>GGAGACAGGGTCAGCGTCACC<br>TGCAAGGCCAGTCAGTATGTGG<br>GTACTTATGTAGCCTGGTATCAA<br>CAGAAACCAGGACAATCTCCTA<br>AAGCACTGATTTACTCGGCATC<br>CACCCGGCACACTGGAGTCCC<br>TGATCGCTTCACAGGCAGTGGA<br>TCTGGGACAGATTTCACTCTCAC<br>CATTAGCAATGTGCAGTCTGAA<br>GACTTGGCAGAGTATTTCTGTGA<br>GCAATACAGCAGCTCTCCTCT
IGKV6-15*01_S9105_AKR	AKR/J	IGKV	MUSMUS IGKV6-15*01 F	97.13	GACATTGTGATGACCCAGTCTC<br>AAAAATTCATGTCCACATCAGTA<br>GGAGACAGGGTCAGCATCAGC<br>TGCAAGGCCAGTCAGAATGTGG<br>GTAATATTATAGCCTGGTATCAA<br>CAGAAACCAGGGCAATCTCCTA<br>AAGCACTGATTTACTTGGCATCC<br>TACCGGTACAGTGGAGTCCCTG<br>ATCGCTTCACAGGCAGTGGATC<br>TGGGACAGATTTCACTCTCACC<br>ATTAGCAATGTGCAGTCTGAAGA<br>CTTGGCAGAGTATTTCTGTCAGC<br>AATATAGCAGCTCTCCTCT
IGKV6-15*01_S9105_MRL	MRL/MPJ	IGKV	MUSMUS IGKV6-15*01 F	97.13	GACATTGTGATGACCCAGTCTC<br>AAAAATTCATGTCCACATCAGTA<br>GGAGACAGGGTCAGCATCAGC<br>TGCAAGGCCAGTCAGAATGTGG<br>GTAATATTATAGCCTGGTATCAA<br>CAGAAACCAGGGCAATCTCCTA<br>AAGCACTGATTTACTTGGCATCC<br>TACCGGTACAGTGGAGTCCCTG<br>ATCGCTTCACAGGCAGTGGATC<br>TGGGACAGATTTCACTCTCACC<br>ATTAGCAATGTGCAGTCTGAAGA<br>CTTGGCAGAGTATTTCTGTCAGC<br>AATATAGCAGCTCTCCTCT

IGKV6-15*01_S9105_NOD	NOD/SHILTJ	IGKV	MUSMUS IGKV6-15*01 F	97.13	GACATTGTGATGACCCAGTCTCAAAAATTCATGTCCACATCAGTAGGAGACAGGGTCAGCATCAGCTGCAAGGCCAGTCAGAATGTGGGTAATATTATAGCCTGGTATCAACAGAAACCAGGGCAATCTCCTAAAGCACTGATTTACTTGGCATCCTACCGGTACAGTGGAGTCCCTGATCGCTTCACAGGCAGTGGATCTGGGACAGATTTCACTCTCACCATTAGCAATGTGCAGTCTGAAGACTTGGCAGAGTATTTCTGTCAGCAATATAGCAGCTCTCCTCT
IGKV6-15*01_S9105_NOR	NOR/LTJ	IGKV	MUSMUS IGKV6-15*01 F	97.13	GACATTGTGATGACCCAGTCTCAAAAATTCATGTCCACATCAGTAGGAGACAGGGTCAGCATCAGCTGCAAGGCCAGTCAGAATGTGGGTAATATTATAGCCTGGTATCAACAGAAACCAGGGCAATCTCCTAAAGCACTGATTTACTTGGCATCCTACCGGTACAGTGGAGTCCCTGATCGCTTCACAGGCAGTGGATCTGGGACAGATTTCACTCTCACCATTAGCAATGTGCAGTCTGAAGACTTGGCAGAGTATTTCTGTCAGCAATATAGCAGCTCTCCTCT
IGKV6-15*01_SJL	SJL/J	IGKV	MUSMUS IGKV6-15*01 F	100	GACATTGTGATGACCCAGTCTCAAAAATTCATGTCCACATCAGTAGGAGACAGGGTCAGCGTCACCTGCAAGGCCAGTCAGAATGTGGGTACTAATGTAGCCTGGTATCAACAGAAACCAGGGCAATCTCCTAAAGCACTGATTTACTCGGCATCCTACCGGTACAGTGGAGTCCCTGATCGCTTCACAGGCAGTGGATCTGGGACAGATTTCACTCTCACCATCAGCAATGTGCAGTCTGAAGACTTGGCAGAGTATTTCTGTCAGCAATATAACAGCTATCCTCT
IGKV6-17*01_129	129S1/SVIMJ	IGKV	MUSMUS IGKV6-17*01 F	100	GACATTGTGATGACCCAGTCTCACAAATTCATGTCCACATCAGTAGGAGACAGGGTCAGCATCACCTGCAAGGCCAGTCAGGATGTGAGTACTGCTGTAGCCTGGTATCAACAGAAACCAGGACAATCTCCTAAACTACTGATTTACTCGGCATCCTACCGGTACACTGGAGTCCCTGATCGCTTCACTGGCAGTGGATCTGGGACGGATTTCACTTTCACCATCAGCAGTGTGCAGGCTGAAGACCTGGCAGTTTATTACTGTCAGCAACATTATAGTACTCCTCC

IGKV6-17*01_AJ	A/J	IGKV	MUSMUS IGKV6-17*01 F	100	GACATTGTGATGACCCAGTCTCACAAATTCATGTCCACATCAGTAGGAGACAGGGTCAGCATCACCTGCAAGGCCAGTCAGGATGTGAGTACTGCTGTAGCCTGGTATCAACAGAAACCAGGACAATCTCCTAAACTACTGATTTACTCGGCATCCTACCGGTACACTGGAGTCCCTGATCGCTTCACTGGCAGTGGATCTGGGACGGATTTCACTTTCACCATCAGCAGTGTGCAGGCTGAAGACCTGGCAGTTTATTACTGTCAGCAACATTATAGTACTCCTCC
IGKV6-17*01_B6	C57BL/6J	IGKV	MUSMUS IGKV6-17*01 F	100	GACATTGTGATGACCCAGTCTCACAAATTCATGTCCACATCAGTAGGAGACAGGGTCAGCATCACCTGCAAGGCCAGTCAGGATGTGAGTACTGCTGTAGCCTGGTATCAACAGAAACCAGGACAATCTCCTAAACTACTGATTTACTCGGCATCCTACCGGTACACTGGAGTCCCTGATCGCTTCACTGGCAGTGGATCTGGGACGGATTTCACTTTCACCATCAGCAGTGTGCAGGCTGAAGACCTGGCAGTTTATTACTGTCAGCAACATTATAGTACTCCTCC
IGKV6-17*01_BALB	BALB/CBYJ	IGKV	MUSMUS IGKV6-17*01 F	100	GACATTGTGATGACCCAGTCTCACAAATTCATGTCCACATCAGTAGGAGACAGGGTCAGCATCACCTGCAAGGCCAGTCAGGATGTGAGTACTGCTGTAGCCTGGTATCAACAGAAACCAGGACAATCTCCTAAACTACTGATTTACTCGGCATCCTACCGGTACACTGGAGTCCCTGATCGCTTCACTGGCAGTGGATCTGGGACGGATTTCACTTTCACCATCAGCAGTGTGCAGGCTGAAGACCTGGCAGTTTATTACTGTCAGCAACATTATAGTACTCCTCC
IGKV6-17*01_C3H	C3H/HEJ	IGKV	MUSMUS IGKV6-17*01 F	100	GACATTGTGATGACCCAGTCTCACAAATTCATGTCCACATCAGTAGGAGACAGGGTCAGCATCACCTGCAAGGCCAGTCAGGATGTGAGTACTGCTGTAGCCTGGTATCAACAGAAACCAGGACAATCTCCTAAACTACTGATTTACTCGGCATCCTACCGGTACACTGGAGTCCCTGATCGCTTCACTGGCAGTGGATCTGGGACGGATTTCACTTTCACCATCAGCAGTGTGCAGGCTGAAGACCTGGCAGTTTATTACTGTCAGCAACATTATAGTACTCCTCC

IGKV6-17*01_CBA	CBA/J	IGKV	MUSMUS IGKV6-17*01 F	100	GACATTGTGATGACCCAGTCTC<br>ACAAATTCATGTCCACATCAGTA<br>GGAGACAGGGTCAGCATCACC<br>TGCAAGGCCAGTCAGGATGTGA<br>GTACTGCTGTAGCCTGGTATCA<br>ACAGAAACCAGGACAATCTCCT<br>AAACTACTGATTTACTCGGCATC<br>CTACCGGTACACTGGAGTCCCT<br>GATCGCTTCACTGGCAGTGGAT<br>CTGGGACGGATTTCACTTTCAC<br>CATCAGCAGTGTGCAGGCTGAA<br>GACCTGGCAGTTTATTACTGTCA<br>GCAACATTATAGTACTCCTCC
IGKV6-17*01_DBA1	DBA/1J	IGKV	MUSMUS IGKV6-17*01 F	100	GACATTGTGATGACCCAGTCTC<br>ACAAATTCATGTCCACATCAGTA<br>GGAGACAGGGTCAGCATCACC<br>TGCAAGGCCAGTCAGGATGTGA<br>GTACTGCTGTAGCCTGGTATCA<br>ACAGAAACCAGGACAATCTCCT<br>AAACTACTGATTTACTCGGCATC<br>CTACCGGTACACTGGAGTCCCT<br>GATCGCTTCACTGGCAGTGGAT<br>CTGGGACGGATTTCACTTTCAC<br>CATCAGCAGTGTGCAGGCTGAA<br>GACCTGGCAGTTTATTACTGTCA<br>GCAACATTATAGTACTCCTCC
IGKV6-17*01_DBA2	DBA/2J	IGKV	MUSMUS IGKV6-17*01 F	100	GACATTGTGATGACCCAGTCTC<br>ACAAATTCATGTCCACATCAGTA<br>GGAGACAGGGTCAGCATCACC<br>TGCAAGGCCAGTCAGGATGTGA<br>GTACTGCTGTAGCCTGGTATCA<br>ACAGAAACCAGGACAATCTCCT<br>AAACTACTGATTTACTCGGCATC<br>CTACCGGTACACTGGAGTCCCT<br>GATCGCTTCACTGGCAGTGGAT<br>CTGGGACGGATTTCACTTTCAC<br>CATCAGCAGTGTGCAGGCTGAA<br>GACCTGGCAGTTTATTACTGTCA<br>GCAACATTATAGTACTCCTCC
IGKV6-17*01_LEWES	LEWES/EIJ	IGKV	MUSMUS IGKV6-17*01 F	100	GACATTGTGATGACCCAGTCTC<br>ACAAATTCATGTCCACATCAGTA<br>GGAGACAGGGTCAGCATCACC<br>TGCAAGGCCAGTCAGGATGTGA<br>GTACTGCTGTAGCCTGGTATCA<br>ACAGAAACCAGGACAATCTCCT<br>AAACTACTGATTTACTCGGCATC<br>CTACCGGTACACTGGAGTCCCT<br>GATCGCTTCACTGGCAGTGGAT<br>CTGGGACGGATTTCACTTTCAC<br>CATCAGCAGTGTGCAGGCTGAA<br>GACCTGGCAGTTTATTACTGTCA<br>GCAACATTATAGTACTCCTCC

IGKV6-17*01_NZB	NZB/BLNJ	IGKV	MUSMUS IGKV6-17*01 F	100	GACATTGTGATGACCCAGTCTCACAAATTCATGTCCACATCAGTAGGAGACAGGGTCAGCATCACCTGCAAGGCCAGTCAGGATGTGAGTACTGCTGTAGCCTGGTATCAACAGAAACCAGGACAATCTCCTAAACTACTGATTTACTCGGCATCCTACCGGTACACTGGAGTCCCTGATCGCTTCACTGGCAGTGGATCTGGGACGGATTTCACTTTCACCATCAGCAGTGTGCAGGCTGAAGACCTGGCAGTTTATTACTGTCAGCAACATTATAGTACTCCTCC
IGKV6-17*01_SJL	SJL/J	IGKV	MUSMUS IGKV6-17*01 F	100	GACATTGTGATGACCCAGTCTCACAAATTCATGTCCACATCAGTAGGAGACAGGGTCAGCATCACCTGCAAGGCCAGTCAGGATGTGAGTACTGCTGTAGCCTGGTATCAACAGAAACCAGGACAATCTCCTAAACTACTGATTTACTCGGCATCCTACCGGTACACTGGAGTCCCTGATCGCTTCACTGGCAGTGGATCTGGGACGGATTTCACTTTCACCATCAGCAGTGTGCAGGCTGAAGACCTGGCAGTTTATTACTGTCAGCAACATTATAGTACTCCTCC
IGKV6-20*01_129	129S1/SVIMJ	IGKV	MUSMUS IGKV6-20*01 F	100	AACATTGTAATGACCCAATCTCCCAAATCCATGTCCATGTCAGTAGGAGAGAGGGTCACCTTGAGCTGCAAGGCCAGTGAGAATGTGGGTACTTATGTATCCTGGTATCAACAGAAACCAGAGCAGTCTCCTAAACTGCTGATATACGGGGCATCCAACCGGTACACTGGGGTCCCCGATCGCTTCACAGGCAGTGGATCTGCAACAGATTTCACTCTGACCATCAGCAGTGTGCAGGCTGAAGACCTTGCAGATTATCACTGTGGACAGAGTTACAGCTATCCTCC
IGKV6-20*01_AJ	A/J	IGKV	MUSMUS IGKV6-20*01 F	100	AACATTGTAATGACCCAATCTCCCAAATCCATGTCCATGTCAGTAGGAGAGAGGGTCACCTTGAGCTGCAAGGCCAGTGAGAATGTGGGTACTTATGTATCCTGGTATCAACAGAAACCAGAGCAGTCTCCTAAACTGCTGATATACGGGGCATCCAACCGGTACACTGGGGTCCCCGATCGCTTCACAGGCAGTGGATCTGCAACAGATTTCACTCTGACCATCAGCAGTGTGCAGGCTGAAGACCTTGCAGATTATCACTGTGGACAGAGTTACAGCTATCCTCC

IGKV6-20*01_B6	C57BL/6J	IGKV	MUSMUS IGKV6-20*01 F	100	AACATTGTAATGACCCAATCTCCCAAATCCATGTCCATGTCAGTAGGAGAGAGGGTCACCTTGAGCTGCAAGGCCAGTGAGAATGTGGGTACTTATGTATCCTGGTATCAACAGAAACCAGAGCAGTCTCCTAAACTGCTGATATACGGGGCATCCAACCGGTACACTGGGGTCCCCGATCGCTTCACAGGCAGTGGATCTGCAACAGATTTCACTCTGACCATCAGCAGTGTGCAGGCTGAAGACCTTGCAGATTATCACTGTGGACAGAGTTACAGCTATCCTCC
IGKV6-20*01_BALB	BALB/CBYJ	IGKV	MUSMUS IGKV6-20*01 F	100	AACATTGTAATGACCCAATCTCCCAAATCCATGTCCATGTCAGTAGGAGAGAGGGTCACCTTGAGCTGCAAGGCCAGTGAGAATGTGGGTACTTATGTATCCTGGTATCAACAGAAACCAGAGCAGTCTCCTAAACTGCTGATATACGGGGCATCCAACCGGTACACTGGGGTCCCCGATCGCTTCACAGGCAGTGGATCTGCAACAGATTTCACTCTGACCATCAGCAGTGTGCAGGCTGAAGACCTTGCAGATTATCACTGTGGACAGAGTTACAGCTATCCTCC
IGKV6-20*01_C3H	C3H/HEJ	IGKV	MUSMUS IGKV6-20*01 F	100	AACATTGTAATGACCCAATCTCCCAAATCCATGTCCATGTCAGTAGGAGAGAGGGTCACCTTGAGCTGCAAGGCCAGTGAGAATGTGGGTACTTATGTATCCTGGTATCAACAGAAACCAGAGCAGTCTCCTAAACTGCTGATATACGGGGCATCCAACCGGTACACTGGGGTCCCCGATCGCTTCACAGGCAGTGGATCTGCAACAGATTTCACTCTGACCATCAGCAGTGTGCAGGCTGAAGACCTTGCAGATTATCACTGTGGACAGAGTTACAGCTATCCTCC
IGKV6-20*01_CBA	CBA/J	IGKV	MUSMUS IGKV6-20*01 F	100	AACATTGTAATGACCCAATCTCCCAAATCCATGTCCATGTCAGTAGGAGAGAGGGTCACCTTGAGCTGCAAGGCCAGTGAGAATGTGGGTACTTATGTATCCTGGTATCAACAGAAACCAGAGCAGTCTCCTAAACTGCTGATATACGGGGCATCCAACCGGTACACTGGGGTCCCCGATCGCTTCACAGGCAGTGGATCTGCAACAGATTTCACTCTGACCATCAGCAGTGTGCAGGCTGAAGACCTTGCAGATTATCACTGTGGACAGAGTTACAGCTATCCTCC

IGKV6-20*01_DBA1	DBA/1J	IGKV	MUSMUS IGKV6-20*01 F	100	AACATTGTAATGACCCAATCTCCCAAATCCATGTCCATGTCAGTAGGAGAGAGGGTCACCTTGAGCTGCAAGGCCAGTGAGAATGTGGGTACTTATGTATCCTGGTATCAACAGAAACCAGAGCAGTCTCCTAAACTGCTGATATACGGGGCATCCAACCGGTACACTGGGGTCCCCGATCGCTTCACAGGCAGTGGATCTGCAACAGATTTCACTCTGACCATCAGCAGTGTGCAGGCTGAAGACCTTGCAGATTATCACTGTGGACAGAGTTACAGCTATCCTCC
IGKV6-20*01_DBA2	DBA/2J	IGKV	MUSMUS IGKV6-20*01 F	100	AACATTGTAATGACCCAATCTCCCAAATCCATGTCCATGTCAGTAGGAGAGAGGGTCACCTTGAGCTGCAAGGCCAGTGAGAATGTGGGTACTTATGTATCCTGGTATCAACAGAAACCAGAGCAGTCTCCTAAACTGCTGATATACGGGGCATCCAACCGGTACACTGGGGTCCCCGATCGCTTCACAGGCAGTGGATCTGCAACAGATTTCACTCTGACCATCAGCAGTGTGCAGGCTGAAGACCTTGCAGATTATCACTGTGGACAGAGTTACAGCTATCCTCC
IGKV6-20*01_LEWES	LEWES/EIJ	IGKV	MUSMUS IGKV6-20*01 F	100	AACATTGTAATGACCCAATCTCCCAAATCCATGTCCATGTCAGTAGGAGAGAGGGTCACCTTGAGCTGCAAGGCCAGTGAGAATGTGGGTACTTATGTATCCTGGTATCAACAGAAACCAGAGCAGTCTCCTAAACTGCTGATATACGGGGCATCCAACCGGTACACTGGGGTCCCCGATCGCTTCACAGGCAGTGGATCTGCAACAGATTTCACTCTGACCATCAGCAGTGTGCAGGCTGAAGACCTTGCAGATTATCACTGTGGACAGAGTTACAGCTATCCTCC
IGKV6-20*01_NZB	NZB/BLNJ	IGKV	MUSMUS IGKV6-20*01 F	100	AACATTGTAATGACCCAATCTCCCAAATCCATGTCCATGTCAGTAGGAGAGAGGGTCACCTTGAGCTGCAAGGCCAGTGAGAATGTGGGTACTTATGTATCCTGGTATCAACAGAAACCAGAGCAGTCTCCTAAACTGCTGATATACGGGGCATCCAACCGGTACACTGGGGTCCCCGATCGCTTCACAGGCAGTGGATCTGCAACAGATTTCACTCTGACCATCAGCAGTGTGCAGGCTGAAGACCTTGCAGATTATCACTGTGGACAGAGTTACAGCTATCCTCC

IGKV6-20*01_S2082_AKR	AKR/J	IGKV	MUSMUS IGKV6-20*01 F	99.64	AACATTGTAATGACCCAATCTCCCAAATCCATGTCCATGTCAGTAGGAGAGAGGGTCACCTTGAGCTGCAAGGCCAGTGAGAATGTGGGTACTTATGTATCCTGGTATCAACAGAAACCAGAGCAGTCTCCTAAACTGCTGATATACGGGGCATCCAACCGGTACACTGGGGTCCCCGATCGCTTCACAGGCAGTGGATCTGCAACAGATTTCACTCTGACCATCAGCAGTGTGCAGGCTGAAGACCTTGCAGATTATTACTGTGGACAGAGTTACAGCTATCCTCC
IGKV6-20*01_S2082_CAST	CAST/EIJ	IGKV	MUSMUS IGKV6-20*01 F	99.64	AACATTGTAATGACCCAATCTCCCAAATCCATGTCCATGTCAGTAGGAGAGAGGGTCACCTTGAGCTGCAAGGCCAGTGAGAATGTGGGTACTTATGTATCCTGGTATCAACAGAAACCAGAGCAGTCTCCTAAACTGCTGATATACGGGGCATCCAACCGGTACACTGGGGTCCCCGATCGCTTCACAGGCAGTGGATCTGCAACAGATTTCACTCTGACCATCAGCAGTGTGCAGGCTGAAGACCTTGCAGATTATTACTGTGGACAGAGTTACAGCTATCCTCC
IGKV6-20*01_S2082_MRL	MRL/MPJ	IGKV	MUSMUS IGKV6-20*01 F	99.64	AACATTGTAATGACCCAATCTCCCAAATCCATGTCCATGTCAGTAGGAGAGAGGGTCACCTTGAGCTGCAAGGCCAGTGAGAATGTGGGTACTTATGTATCCTGGTATCAACAGAAACCAGAGCAGTCTCCTAAACTGCTGATATACGGGGCATCCAACCGGTACACTGGGGTCCCCGATCGCTTCACAGGCAGTGGATCTGCAACAGATTTCACTCTGACCATCAGCAGTGTGCAGGCTGAAGACCTTGCAGATTATTACTGTGGACAGAGTTACAGCTATCCTCC
IGKV6-20*01_S2082_NOD	NOD/SHILTJ	IGKV	MUSMUS IGKV6-20*01 F	99.64	AACATTGTAATGACCCAATCTCCCAAATCCATGTCCATGTCAGTAGGAGAGAGGGTCACCTTGAGCTGCAAGGCCAGTGAGAATGTGGGTACTTATGTATCCTGGTATCAACAGAAACCAGAGCAGTCTCCTAAACTGCTGATATACGGGGCATCCAACCGGTACACTGGGGTCCCCGATCGCTTCACAGGCAGTGGATCTGCAACAGATTTCACTCTGACCATCAGCAGTGTGCAGGCTGAAGACCTTGCAGATTATTACTGTGGACAGAGTTACAGCTATCCTCC

IGKV6-20*01_S2082_NOR	NOR/LTJ	IGKV	MUSMUS IGKV6-20*01 F	99.64	AACATTGTAATGACCCAATCTCCCAAATCCATGTCCATGTCAGTAGGAGAGAGGGTCACCTTGAGCTGCAAGGCCAGTGAGAATGTGGGTACTTATGTATCCTGGTATCAACAGAAACCAGAGCAGTCTCCTAAACTGCTGATATACGGGGCATCCAACCGGTACACTGGGGTCCCCGATCGCTTCACAGGCAGTGGATCTGCAACAGATTTCACTCTGACCATCAGCAGTGTGCAGGCTGAAGACCTTGCAGATTATTACTGTGGACAGAGTTACAGCTATCCTCC
IGKV6-20*01_S2863_MSM	MSM/MSJ	IGKV	MUSMUS IGKV6-20*01 F	99.28	AACATTGTAATGACCCAATCTCCCAAATCCATGTCCATGTCAGTAGGAGAGAGGGTCACCTTGAGCTGCAAGGCCAGTGAGAATGTGGGTACTTATGTATCCTGGTATCAACAGAAACCAGAGCAGTCTCCTAAACTGCTGATATACGGGGCATCCAACCGGTACACTGGGGTCCCTGATCGCTTCACAGGCAGTGGATCTGCAACAGATTTCACTCTGACCATCAGCAGTGTGCAGGCTGAAGACTTTGCAGATTATCACTGTGGACAGAGTTACAGCTATCCTCC
IGKV6-20*01_S7621_PWD	PWD/PHJ	IGKV	MUSMUS IGKV6-20*01 F	99.64	AACATTGTAATGACCCAATCTCCCAAATCCATGTCCATGTCAGTAGGAGAGAGGGTCACCTTGAGCTGCAAGGCCAGTGAGAATGTGGGTACTTATGTATCCTGGTATCAACAGAAACCAGAGCAGTCTCCTAAACTGCTGATATACGGGGCATCCAACCGGTACACTGGGGTCCCCGATCGCTTCACAGGCAGTGGATCTGCAACAGATTTCACTCTGACCATCAGCAGTGTGCAGGCTGAAGACCTTGCAGATTATCACTGTGCACAGAGTTACAGCTATCCTCC
IGKV6-20*01_SJL	SJL/J	IGKV	MUSMUS IGKV6-20*01 F	100	AACATTGTAATGACCCAATCTCCCAAATCCATGTCCATGTCAGTAGGAGAGAGGGTCACCTTGAGCTGCAAGGCCAGTGAGAATGTGGGTACTTATGTATCCTGGTATCAACAGAAACCAGAGCAGTCTCCTAAACTGCTGATATACGGGGCATCCAACCGGTACACTGGGGTCCCCGATCGCTTCACAGGCAGTGGATCTGCAACAGATTTCACTCTGACCATCAGCAGTGTGCAGGCTGAAGACCTTGCAGATTATCACTGTGGACAGAGTTACAGCTATCCTCC

IGKV6-23*01_129	129S1/SVIMJ	IGKV	MUSMUS IGKV6-23*01 F	100	GACATTGTGATGACCCAGTCTCACAAATTCATGTCCACATCAGTAGGAGACAGGGTCAGCATCACCTGCAAGGCCAGTCAGGATGTGGGTACTGCTGTAGCCTGGTATCAACAGAAACCAGGGCAATCTCCTAAACTACTGATTTACTGGGCATCCACCCGGCACACTGGAGTCCCTGATCGCTTCACAGGCAGTGGATCTGGGACAGATTTCACTCTCACCATTAGCAATGTGCAGTCTGAAGACTTGGCAGATTATTTCTGTCAGCAATATAGCAGCTATCCTCT
IGKV6-23*01_AJ	A/J	IGKV	MUSMUS IGKV6-23*01 F	100	GACATTGTGATGACCCAGTCTCACAAATTCATGTCCACATCAGTAGGAGACAGGGTCAGCATCACCTGCAAGGCCAGTCAGGATGTGGGTACTGCTGTAGCCTGGTATCAACAGAAACCAGGGCAATCTCCTAAACTACTGATTTACTGGGCATCCACCCGGCACACTGGAGTCCCTGATCGCTTCACAGGCAGTGGATCTGGGACAGATTTCACTCTCACCATTAGCAATGTGCAGTCTGAAGACTTGGCAGATTATTTCTGTCAGCAATATAGCAGCTATCCTCT
IGKV6-23*01_B6	C57BL/6J	IGKV	MUSMUS IGKV6-23*01 F	100	GACATTGTGATGACCCAGTCTCACAAATTCATGTCCACATCAGTAGGAGACAGGGTCAGCATCACCTGCAAGGCCAGTCAGGATGTGGGTACTGCTGTAGCCTGGTATCAACAGAAACCAGGGCAATCTCCTAAACTACTGATTTACTGGGCATCCACCCGGCACACTGGAGTCCCTGATCGCTTCACAGGCAGTGGATCTGGGACAGATTTCACTCTCACCATTAGCAATGTGCAGTCTGAAGACTTGGCAGATTATTTCTGTCAGCAATATAGCAGCTATCCTCT
IGKV6-23*01_BALB	BALB/CBYJ	IGKV	MUSMUS IGKV6-23*01 F	100	GACATTGTGATGACCCAGTCTCACAAATTCATGTCCACATCAGTAGGAGACAGGGTCAGCATCACCTGCAAGGCCAGTCAGGATGTGGGTACTGCTGTAGCCTGGTATCAACAGAAACCAGGGCAATCTCCTAAACTACTGATTTACTGGGCATCCACCCGGCACACTGGAGTCCCTGATCGCTTCACAGGCAGTGGATCTGGGACAGATTTCACTCTCACCATTAGCAATGTGCAGTCTGAAGACTTGGCAGATTATTTCTGTCAGCAATATAGCAGCTATCCTCT

IGKV6-23*01_C3H	C3H/HEJ	IGKV	MUSMUS IGKV6-23*01 F	100	GACATTGTGATGACCCAGTCTC<br>ACAAATTCATGTCCACATCAGTA<br>GGAGACAGGGTCAGCATCACC<br>TGCAAGGCCAGTCAGGATGTG<br>GGTACTGCTGTAGCCTGGTATC<br>AACAGAAACCAGGGCAATCTCC<br>TAAACTACTGATTTACTGGGCAT<br>CCACCCGGCACACTGGAGTCC<br>CTGATCGCTTCACAGGCAGTGG<br>ATCTGGGACAGATTTCACTCTCA<br>CCATTAGCAATGTGCAGTCTGAA<br>GACTTGGCAGATTATTTCTGTCA<br>GCAATATAGCAGCTATCCTCT
IGKV6-23*01_CBA	CBA/J	IGKV	MUSMUS IGKV6-23*01 F	100	GACATTGTGATGACCCAGTCTC<br>ACAAATTCATGTCCACATCAGTA<br>GGAGACAGGGTCAGCATCACC<br>TGCAAGGCCAGTCAGGATGTG<br>GGTACTGCTGTAGCCTGGTATC<br>AACAGAAACCAGGGCAATCTCC<br>TAAACTACTGATTTACTGGGCAT<br>CCACCCGGCACACTGGAGTCC<br>CTGATCGCTTCACAGGCAGTGG<br>ATCTGGGACAGATTTCACTCTCA<br>CCATTAGCAATGTGCAGTCTGAA<br>GACTTGGCAGATTATTTCTGTCA<br>GCAATATAGCAGCTATCCTCT
IGKV6-23*01_DBA1	DBA/1J	IGKV	MUSMUS IGKV6-23*01 F	100	GACATTGTGATGACCCAGTCTC<br>ACAAATTCATGTCCACATCAGTA<br>GGAGACAGGGTCAGCATCACC<br>TGCAAGGCCAGTCAGGATGTG<br>GGTACTGCTGTAGCCTGGTATC<br>AACAGAAACCAGGGCAATCTCC<br>TAAACTACTGATTTACTGGGCAT<br>CCACCCGGCACACTGGAGTCC<br>CTGATCGCTTCACAGGCAGTGG<br>ATCTGGGACAGATTTCACTCTCA<br>CCATTAGCAATGTGCAGTCTGAA<br>GACTTGGCAGATTATTTCTGTCA<br>GCAATATAGCAGCTATCCTCT
IGKV6-23*01_DBA2	DBA/2J	IGKV	MUSMUS IGKV6-23*01 F	100	GACATTGTGATGACCCAGTCTC<br>ACAAATTCATGTCCACATCAGTA<br>GGAGACAGGGTCAGCATCACC<br>TGCAAGGCCAGTCAGGATGTG<br>GGTACTGCTGTAGCCTGGTATC<br>AACAGAAACCAGGGCAATCTCC<br>TAAACTACTGATTTACTGGGCAT<br>CCACCCGGCACACTGGAGTCC<br>CTGATCGCTTCACAGGCAGTGG<br>ATCTGGGACAGATTTCACTCTCA<br>CCATTAGCAATGTGCAGTCTGAA<br>GACTTGGCAGATTATTTCTGTCA<br>GCAATATAGCAGCTATCCTCT

IGKV6-23*01_LEWES	LEWES/EIJ	IGKV	MUSMUS IGKV6-23*01 F	100	GACATTGTGATGACCCAGTCTCACAAATTCATGTCCACATCAGTAGGAGACAGGGTCAGCATCACCTGCAAGGCCAGTCAGGATGTGGGTACTGCTGTAGCCTGGTATCAACAGAAACCAGGGCAATCTCCTAAACTACTGATTTACTGGGCATCCACCCGGCACACTGGAGTCCCTGATCGCTTCACAGGCAGTGGATCTGGGACAGATTTCACTCTCACCATTAGCAATGTGCAGTCTGAAGACTTGGCAGATTATTTCTGTCAGCAATATAGCAGCTATCCTCT
IGKV6-23*01_NZB	NZB/BLNJ	IGKV	MUSMUS IGKV6-23*01 F	100	GACATTGTGATGACCCAGTCTCACAAATTCATGTCCACATCAGTAGGAGACAGGGTCAGCATCACCTGCAAGGCCAGTCAGGATGTGGGTACTGCTGTAGCCTGGTATCAACAGAAACCAGGGCAATCTCCTAAACTACTGATTTACTGGGCATCCACCCGGCACACTGGAGTCCCTGATCGCTTCACAGGCAGTGGATCTGGGACAGATTTCACTCTCACCATTAGCAATGTGCAGTCTGAAGACTTGGCAGATTATTTCTGTCAGCAATATAGCAGCTATCCTCT
IGKV6-23*01_S0027_MSM	MSM/MSJ	IGKV	MUSMUS IGKV6-23*01 F	93.91	GACATTGTGATGACCCAGTCGCAAAAATTCATGTCCACATCAGTAGGAGAGAGGGTCGGCATCAGCTGCAAGGCCAGTCAGAATGTGGGTACTGCTGTAGCCTGGTATCAACAGAAACCAGGGCAGTCTCCTAAACTACTGATTTACTGGGCATCCACCCGGCACACTGAAGTCCCTGATCGCTTCACAGGCAGTGGATCTGGGACAGATTTCACTCTTACCATCAGCAGTATGCAGTCTGAAGACCTGGCAGATTATTTCTGTCAGCAACATTATAGCACTCCTCT
IGKV6-23*01_S4960_AKR	AKR/J	IGKV	MUSMUS IGKV6-23*01 F	92.11	GACATTGTGATGACCCAGTCTCAAAAATTCATGTCCACATCAGTAGGAGAGAGGGTCAGCATCACCTGCAAGGCCAGTCAGAATGTAGGTACTAATGTAGCCTGGTATCAGCAGAAAGCAGGGCAGTCTCTTGAACTGCTGATCTATGGGGCATCCAACCGGCACACTGGAGTCCCTGATCGCTTCACAGGCAGTGGATCTGGGACAGATTTCACTCTCACCATCACCAATGTGCAGTCTGAAGACATGACAAATTATTTCTGTGAGCAATATAGCAGCTATCCTCT

IGKV6-23*01_S4960_CAST	CAST/EIJ	IGKV	MUSMUS IGKV6-23*01 F	92.11	GACATTGTGATGACCCAGTCTCAAAAATTCATGTCCACATCAGTAGGAGAGAGGGTCAGCATCACCTGCAAGGCCAGTCAGAATGTAGGTACTAATGTAGCCTGGTATCAGCAGAAAGCAGGGCAGTCTCTTGAACTGCTGATCTATGGGGCATCCAACCGGCACACTGGAGTCCCTGATCGCTTCACAGGCAGTGGATCTGGGACAGATTTCACTCTCACCATCACCAATGTGCAGTCTGAAGACATGACAAATTATTTCTGTGAGCAATATAGCAGCTATCCTCT
IGKV6-23*01_S4960_MRL	MRL/MPJ	IGKV	MUSMUS IGKV6-23*01 F	92.11	GACATTGTGATGACCCAGTCTCAAAAATTCATGTCCACATCAGTAGGAGAGAGGGTCAGCATCACCTGCAAGGCCAGTCAGAATGTAGGTACTAATGTAGCCTGGTATCAGCAGAAAGCAGGGCAGTCTCTTGAACTGCTGATCTATGGGGCATCCAACCGGCACACTGGAGTCCCTGATCGCTTCACAGGCAGTGGATCTGGGACAGATTTCACTCTCACCATCACCAATGTGCAGTCTGAAGACATGACAAATTATTTCTGTGAGCAATATAGCAGCTATCCTCT
IGKV6-23*01_S4960_NOD	NOD/SHILTJ	IGKV	MUSMUS IGKV6-23*01 F	92.11	GACATTGTGATGACCCAGTCTCAAAAATTCATGTCCACATCAGTAGGAGAGAGGGTCAGCATCACCTGCAAGGCCAGTCAGAATGTAGGTACTAATGTAGCCTGGTATCAGCAGAAAGCAGGGCAGTCTCTTGAACTGCTGATCTATGGGGCATCCAACCGGCACACTGGAGTCCCTGATCGCTTCACAGGCAGTGGATCTGGGACAGATTTCACTCTCACCATCACCAATGTGCAGTCTGAAGACATGACAAATTATTTCTGTGAGCAATATAGCAGCTATCCTCT
IGKV6-23*01_S4960_NOR	NOR/LTJ	IGKV	MUSMUS IGKV6-23*01 F	92.11	GACATTGTGATGACCCAGTCTCAAAAATTCATGTCCACATCAGTAGGAGAGAGGGTCAGCATCACCTGCAAGGCCAGTCAGAATGTAGGTACTAATGTAGCCTGGTATCAGCAGAAAGCAGGGCAGTCTCTTGAACTGCTGATCTATGGGGCATCCAACCGGCACACTGGAGTCCCTGATCGCTTCACAGGCAGTGGATCTGGGACAGATTTCACTCTCACCATCACCAATGTGCAGTCTGAAGACATGACAAATTATTTCTGTGAGCAATATAGCAGCTATCCTCT

IGKV6-23*01_S5820_CAST	CAST/EIJ	IGKV	MUSMUS IGKV6-23*01 F	92.11	GACATTGTGATGACCCAGTCTCAAAAATTCATGTCCACATCAGTAGGAGAGAGGGTCAGCATCACCTGCAAGGCCAGTCAGAATGTAGGTACTAATGTAGCCTGGTATCAGCAGAAAGCAGGGCAGTCTCTTGAACTGCTGATCTATGGGGCATCCAACCGGCACACTGGAGTCCCTGATCGCTTCACAGGCAGTGGATCTGGGACAGATTTCACTCTCACCATCACCAATGTGCAGTCTGAAGACATGACAAATTATTTCTGTGAGCAATATAGCAGCTATCCGCT
IGKV6-23*01_S5820_NOD	NOD/SHILTJ	IGKV	MUSMUS IGKV6-23*01 F	92.11	GACATTGTGATGACCCAGTCTCAAAAATTCATGTCCACATCAGTAGGAGAGAGGGTCAGCATCACCTGCAAGGCCAGTCAGAATGTAGGTACTAATGTAGCCTGGTATCAGCAGAAAGCAGGGCAGTCTCTTGAACTGCTGATCTATGGGGCATCCAACCGGCACACTGGAGTCCCTGATCGCTTCACAGGCAGTGGATCTGGGACAGATTTCACTCTCACCATCACCAATGTGCAGTCTGAAGACATGACAAATTATTTCTGTGAGCAATATAGCAGCTATCCGCT
IGKV6-23*01_SJL	SJL/J	IGKV	MUSMUS IGKV6-23*01 F	100	GACATTGTGATGACCCAGTCTCACAAATTCATGTCCACATCAGTAGGAGACAGGGTCAGCATCACCTGCAAGGCCAGTCAGGATGTGGGTACTGCTGTAGCCTGGTATCAACAGAAACCAGGGCAATCTCCTAAACTACTGATTTACTGGGCATCCACCCGGCACACTGGAGTCCCTGATCGCTTCACAGGCAGTGGATCTGGGACAGATTTCACTCTCACCATTAGCAATGTGCAGTCTGAAGACTTGGCAGATTATTTCTGTCAGCAATATAGCAGCTATCCTCT
IGKV6-25*01_129	129S1/SVIMJ	IGKV	MUSMUS IGKV6-25*01 F	100	GACATTGTGATGACCCAGTCTCACAAATTCATGTCCACATCAGTAGGAGACAGGGTCAGCATCACCTGCAAGGCCAGTCAGGATGTGAGTACTGCTGTAGCCTGGTATCAACAAAAACCAGGGCAATCTCCTAAACTACTGATTTACTGGGCATCCACCCGGCACACTGGAGTCCCTGATCGCTTCACAGGCAGTGGATCTGGGACAGATTATACTCTCACCATCAGCAGTGTGCAGGCTGAAGACCTGGCACTTTATTACTGTCAGCAACATTATAGCACTCCTCC

IGKV6-25*01_AJ	A/J	IGKV	MUSMUS IGKV6-25*01 F	100	GACATTGTGATGACCCAGTCTCACAAATTCATGTCCACATCAGTAGGAGACAGGGTCAGCATCACCTGCAAGGCCAGTCAGGATGTGAGTACTGCTGTAGCCTGGTATCAACAAAAACCAGGGCAATCTCCTAAACTACTGATTTACTGGGCATCCACCCGGCACACTGGAGTCCCTGATCGCTTCACAGGCAGTGGATCTGGGACAGATTATACTCTCACCATCAGCAGTGTGCAGGCTGAAGACCTGGCACTTTATTACTGTCAGCAACATTATAGCACTCCTCC
IGKV6-25*01_B6	C57BL/6J	IGKV	MUSMUS IGKV6-25*01 F	100	GACATTGTGATGACCCAGTCTCACAAATTCATGTCCACATCAGTAGGAGACAGGGTCAGCATCACCTGCAAGGCCAGTCAGGATGTGAGTACTGCTGTAGCCTGGTATCAACAAAAACCAGGGCAATCTCCTAAACTACTGATTTACTGGGCATCCACCCGGCACACTGGAGTCCCTGATCGCTTCACAGGCAGTGGATCTGGGACAGATTATACTCTCACCATCAGCAGTGTGCAGGCTGAAGACCTGGCACTTTATTACTGTCAGCAACATTATAGCACTCCTCC
IGKV6-25*01_BALB	BALB/CBYJ	IGKV	MUSMUS IGKV6-25*01 F	100	GACATTGTGATGACCCAGTCTCACAAATTCATGTCCACATCAGTAGGAGACAGGGTCAGCATCACCTGCAAGGCCAGTCAGGATGTGAGTACTGCTGTAGCCTGGTATCAACAAAAACCAGGGCAATCTCCTAAACTACTGATTTACTGGGCATCCACCCGGCACACTGGAGTCCCTGATCGCTTCACAGGCAGTGGATCTGGGACAGATTATACTCTCACCATCAGCAGTGTGCAGGCTGAAGACCTGGCACTTTATTACTGTCAGCAACATTATAGCACTCCTCC
IGKV6-25*01_C3H	C3H/HEJ	IGKV	MUSMUS IGKV6-25*01 F	100	GACATTGTGATGACCCAGTCTCACAAATTCATGTCCACATCAGTAGGAGACAGGGTCAGCATCACCTGCAAGGCCAGTCAGGATGTGAGTACTGCTGTAGCCTGGTATCAACAAAAACCAGGGCAATCTCCTAAACTACTGATTTACTGGGCATCCACCCGGCACACTGGAGTCCCTGATCGCTTCACAGGCAGTGGATCTGGGACAGATTATACTCTCACCATCAGCAGTGTGCAGGCTGAAGACCTGGCACTTTATTACTGTCAGCAACATTATAGCACTCCTCC

IGKV6-25*01_CBA	CBA/J	IGKV	MUSMUS IGKV6-25*01 F	100	GACATTGTGATGACCCAGTCTC<br>ACAAATTCATGTCCACATCAGTA<br>GGAGACAGGGTCAGCATCACC<br>TGCAAGGCCAGTCAGGATGTGA<br>GTACTGCTGTAGCCTGGTATCA<br>ACAAAAACCAGGGCAATCTCCT<br>AAACTACTGATTTACTGGGCATC<br>CACCCGGCACACTGGAGTCCC<br>TGATCGCTTCACAGGCAGTGGA<br>TCTGGGACAGATTATACTCTCAC<br>CATCAGCAGTGTGCAGGCTGAA<br>GACCTGGCACTTTATTACTGTCA<br>GCAACATTATAGCACTCCTCC
IGKV6-25*01_DBA1	DBA/1J	IGKV	MUSMUS IGKV6-25*01 F	100	GACATTGTGATGACCCAGTCTC<br>ACAAATTCATGTCCACATCAGTA<br>GGAGACAGGGTCAGCATCACC<br>TGCAAGGCCAGTCAGGATGTGA<br>GTACTGCTGTAGCCTGGTATCA<br>ACAAAAACCAGGGCAATCTCCT<br>AAACTACTGATTTACTGGGCATC<br>CACCCGGCACACTGGAGTCCC<br>TGATCGCTTCACAGGCAGTGGA<br>TCTGGGACAGATTATACTCTCAC<br>CATCAGCAGTGTGCAGGCTGAA<br>GACCTGGCACTTTATTACTGTCA<br>GCAACATTATAGCACTCCTCC
IGKV6-25*01_DBA2	DBA/2J	IGKV	MUSMUS IGKV6-25*01 F	100	GACATTGTGATGACCCAGTCTC<br>ACAAATTCATGTCCACATCAGTA<br>GGAGACAGGGTCAGCATCACC<br>TGCAAGGCCAGTCAGGATGTGA<br>GTACTGCTGTAGCCTGGTATCA<br>ACAAAAACCAGGGCAATCTCCT<br>AAACTACTGATTTACTGGGCATC<br>CACCCGGCACACTGGAGTCCC<br>TGATCGCTTCACAGGCAGTGGA<br>TCTGGGACAGATTATACTCTCAC<br>CATCAGCAGTGTGCAGGCTGAA<br>GACCTGGCACTTTATTACTGTCA<br>GCAACATTATAGCACTCCTCC
IGKV6-25*01_LEWES	LEWES/EIJ	IGKV	MUSMUS IGKV6-25*01 F	100	GACATTGTGATGACCCAGTCTC<br>ACAAATTCATGTCCACATCAGTA<br>GGAGACAGGGTCAGCATCACC<br>TGCAAGGCCAGTCAGGATGTGA<br>GTACTGCTGTAGCCTGGTATCA<br>ACAAAAACCAGGGCAATCTCCT<br>AAACTACTGATTTACTGGGCATC<br>CACCCGGCACACTGGAGTCCC<br>TGATCGCTTCACAGGCAGTGGA<br>TCTGGGACAGATTATACTCTCAC<br>CATCAGCAGTGTGCAGGCTGAA<br>GACCTGGCACTTTATTACTGTCA<br>GCAACATTATAGCACTCCTCC

IGKV6-25*01_NZB	NZB/BLNJ	IGKV	MUSMUS IGKV6-25*01 F	100	GACATTGTGATGACCCAGTCTCACAAATTCATGTCCACATCAGTAGGAGACAGGGTCAGCATCACCTGCAAGGCCAGTCAGGATGTGAGTACTGCTGTAGCCTGGTATCAACAAAAACCAGGGCAATCTCCTAAACTACTGATTTACTGGGCATCCACCCGGCACACTGGAGTCCCTGATCGCTTCACAGGCAGTGGATCTGGGACAGATTATACTCTCACCATCAGCAGTGTGCAGGCTGAAGACCTGGCACTTTATTACTGTCAGCAACATTATAGCACTCCTCC
IGKV6-25*01_S2065_PWD	PWD/PHJ	IGKV	MUSMUS IGKV6-13*01 F, OR MUSMUS IGKV6-14*01 F	92.11	GACATTGTGATGACCCAGTCACAAAAATTCATGTCCACATCAGTAGGAGAGAGGGTCAGCATCAGCTGCAAGGCCAGTCAGAATGTGGGTAATATTATAGCCTGGTATCAACAGAAACCAGGGCAGTCTCCTAAACCACTGATTTACTTGGCTTCCAATCGGCACACTGGAGTCCCTGATCGCTTCACAGGCAGTGGATCTGGGACAGATTTCACTCTGACCATCAGCAGTGTGCAGGCTGAAGACCTGGCAGATTATTTCTGTCAGCAACATTATAGCACTCCTCC
IGKV6-25*01_S3519_AKR	AKR/J	IGKV	MUSMUS IGKV6-25*01 F	98.21	TGCATTGTGATGACCCAGTCTCACAAATTCATGTCCACATCAGTAGGAGACAGGGTCAGCATCACCTGCAAGGCCAGTCAGGATGTGAGTACTGCTGTAGCCTGGTATCAACAAAAACCAGGGCAATCTCCTAAACTACTGATTTACTGGGCATCCACCCGGCACACTGGAGTCCCTGATCGCTTCACAGGCAGTGGATCTGGGACAGATTTCACTCTTACCATCAGCAGTGTGCAGGCTGAAGACCTGGCACTTTATTACTGTCAGCAACATTATAGCACTCCTCC
IGKV6-25*01_S3519_CAST	CAST/EIJ	IGKV	MUSMUS IGKV6-25*01 F	98.21	TGCATTGTGATGACCCAGTCTCACAAATTCATGTCCACATCAGTAGGAGACAGGGTCAGCATCACCTGCAAGGCCAGTCAGGATGTGAGTACTGCTGTAGCCTGGTATCAACAAAAACCAGGGCAATCTCCTAAACTACTGATTTACTGGGCATCCACCCGGCACACTGGAGTCCCTGATCGCTTCACAGGCAGTGGATCTGGGACAGATTTCACTCTTACCATCAGCAGTGTGCAGGCTGAAGACCTGGCACTTTATTACTGTCAGCAACATTATAGCACTCCTCC

IGKV6-25*01_S3805_MSM	MSM/MSJ	IGKV	MUSMUS IGKV6-23*01 F	93.91	GACATTGTGATGACCCAGTCGCAAAAATTCATGTCCACATCAGTAGGAGAGAGGGTCGGCATCAGCTGCAAGGCCAGTCAGAATGTGGGTACTGCTGTAGCCTGGTATCAACAGAAACCAGGGCAGTCTCCTAAACTACTGATTTACTGGGCATCCACCCGGCACACTGAAGTCCCTGATCGCTTCACAGGCAGTGGATCTGGGACAGATTTCACTCTTACCATCAGCAGTATGCAGTCTGAAGACCTGGCAGATTATTTCTGTCAGCAACATTATAGCACTCCTCC
IGKV6-25*01_S5447_MRL	MRL/MPJ	IGKV	MUSMUS IGKV6-25*01 F	98.21	TGCATTGTGATGACCCAGTCTCACAAATTCATGTCCACATCAGTAGGAGACAGGGTCAGCATCACCTGCAAGGCCAGTCAGGATGTGAGTACTGCTGTAGCCTGGTATCAACAAAAACCAGGGCAATCTCCTAAACTACTGATTTACTGGGCATCCACCCGGCACACTGGAGTCCCTGATCGCTTCACAGGCAGTGGATCTGGGACAGATTTCACTCTTACCATCAGCAGTGTGCAGGCTGAAGACCTGGCACTTTATTACTGTCAGCAACATTATAGCACTCCTC
IGKV6-25*01_S5447_NOR	NOR/LTJ	IGKV	MUSMUS IGKV6-25*01 F	98.21	TGCATTGTGATGACCCAGTCTCACAAATTCATGTCCACATCAGTAGGAGACAGGGTCAGCATCACCTGCAAGGCCAGTCAGGATGTGAGTACTGCTGTAGCCTGGTATCAACAAAAACCAGGGCAATCTCCTAAACTACTGATTTACTGGGCATCCACCCGGCACACTGGAGTCCCTGATCGCTTCACAGGCAGTGGATCTGGGACAGATTTCACTCTTACCATCAGCAGTGTGCAGGCTGAAGACCTGGCACTTTATTACTGTCAGCAACATTATAGCACTCCTC
IGKV6-25*01_S9703_AKR	AKR/J	IGKV	MUSMUS IGKV6-25*01 F	97.49	GACATTGTGATGACCCAGTCTCACAAATTCATGTCCACATCAGTAGGAGACAGGGTCAGCATCACCTGCAAGGCCAGTCAGGATGTGGGTACTGCTGTAGCCTGGTATCAACAGAAACCAGGGCAATCTCCTAAACTACTGATTTACTGGGCATCCACCCGGCACACTGGAGTCCCTGATCCCTTCACAGGCAGTGGATCTGGGACAGATTTCACTCTCACCATCAGCAGTGTGCAGGCTGAAGACCTGGCAGTTTATTACTGTCAGCAAGATTATAGCACTCCTCC

IGKV6-25*01_SJL	SJL/J	IGKV	MUSMUS IGKV6-25*01 F	100	GACATTGTGATGACCCAGTCTCACAAATTCATGTCCACATCAGTAGGAGACAGGGTCAGCATCACCTGCAAGGCCAGTCAGGATGTGAGTACTGCTGTAGCCTGGTATCAACAAAAACCAGGGCAATCTCCTAAACTACTGATTTACTGGGCATCCACCCGGCACACTGGAGTCCCTGATCGCTTCACAGGCAGTGGATCTGGGACAGATTATACTCTCACCATCAGCAGTGTGCAGGCTGAAGACCTGGCACTTTATTACTGTCAGCAACATTATAGCACTCCTCC
IGKV6-29*01_AJ	A/J	IGKV	MUSMUS IGKV6-29*01 F	100	AACATTGTAATGACCCAATCTCCCAAATCCATGTCCATGTCAGTAGGAGAGAGGGTCACCTTGAGCTGCAAGGCCAGTGAGAATGTGGGTACTTATGTATCCTGGTATCAACAGAAACCAGAGCAGTCTCCTAAACTGCTGATATACGGGGCATCCAACCGGTACCCTGGGGTCCCTGATCGCTTCACAGGCAGTGGATCTGCAACAGATTTCACTCTGACCATCAGCAGTCTGCAGGCTGAAGACCTTGCAGATTATCACTGTGGACAGGGTTACAGCTATCTTCC
IGKV6-29*01_BALB	BALB/CBYJ	IGKV	MUSMUS IGKV6-29*01 F	100	AACATTGTAATGACCCAATCTCCCAAATCCATGTCCATGTCAGTAGGAGAGAGGGTCACCTTGAGCTGCAAGGCCAGTGAGAATGTGGGTACTTATGTATCCTGGTATCAACAGAAACCAGAGCAGTCTCCTAAACTGCTGATATACGGGGCATCCAACCGGTACCCTGGGGTCCCTGATCGCTTCACAGGCAGTGGATCTGCAACAGATTTCACTCTGACCATCAGCAGTCTGCAGGCTGAAGACCTTGCAGATTATCACTGTGGACAGGGTTACAGCTATCTTCC
IGKV6-29*01_C3H	C3H/HEJ	IGKV	MUSMUS IGKV6-29*01 F	100	AACATTGTAATGACCCAATCTCCCAAATCCATGTCCATGTCAGTAGGAGAGAGGGTCACCTTGAGCTGCAAGGCCAGTGAGAATGTGGGTACTTATGTATCCTGGTATCAACAGAAACCAGAGCAGTCTCCTAAACTGCTGATATACGGGGCATCCAACCGGTACCCTGGGGTCCCTGATCGCTTCACAGGCAGTGGATCTGCAACAGATTTCACTCTGACCATCAGCAGTCTGCAGGCTGAAGACCTTGCAGATTATCACTGTGGACAGGGTTACAGCTATCTTCC

IGKV6-29*01_DBA1	DBA/1J	IGKV	MUSMUS IGKV6-29*01 F	100	AACATTGTAATGACCCAATCTCCCAAATCCATGTCCATGTCAGTAGGAGAGAGGGTCACCTTGAGCTGCAAGGCCAGTGAGAATGTGGGTACTTATGTATCCTGGTATCAACAGAAACCAGAGCAGTCTCCTAAACTGCTGATATACGGGGCATCCAACCGGTACCCTGGGGTCCCTGATCGCTTCACAGGCAGTGGATCTGCAACAGATTTCACTCTGACCATCAGCAGTCTGCAGGCTGAAGACCTTGCAGATTATCACTGTGGACAGGGTTACAGCTATCTTCC
IGKV6-29*01_DBA2	DBA/2J	IGKV	MUSMUS IGKV6-29*01 F	100	AACATTGTAATGACCCAATCTCCCAAATCCATGTCCATGTCAGTAGGAGAGAGGGTCACCTTGAGCTGCAAGGCCAGTGAGAATGTGGGTACTTATGTATCCTGGTATCAACAGAAACCAGAGCAGTCTCCTAAACTGCTGATATACGGGGCATCCAACCGGTACCCTGGGGTCCCTGATCGCTTCACAGGCAGTGGATCTGCAACAGATTTCACTCTGACCATCAGCAGTCTGCAGGCTGAAGACCTTGCAGATTATCACTGTGGACAGGGTTACAGCTATCTTCC
IGKV6-29*01_NZB	NZB/BLNJ	IGKV	MUSMUS IGKV6-29*01 F	100	AACATTGTAATGACCCAATCTCCCAAATCCATGTCCATGTCAGTAGGAGAGAGGGTCACCTTGAGCTGCAAGGCCAGTGAGAATGTGGGTACTTATGTATCCTGGTATCAACAGAAACCAGAGCAGTCTCCTAAACTGCTGATATACGGGGCATCCAACCGGTACCCTGGGGTCCCTGATCGCTTCACAGGCAGTGGATCTGCAACAGATTTCACTCTGACCATCAGCAGTCTGCAGGCTGAAGACCTTGCAGATTATCACTGTGGACAGGGTTACAGCTATCTTCC
IGKV6-32*01_129	129S1/SVIMJ	IGKV	MUSMUS IGKV6-32*01 F	100	AGTATTGTGATGACCCAGACTCCCAAATTCCTGCTTGTATCAGCAGGAGACAGGGTTACCATAACCTGCAAGGCCAGTCAGAGTGTGAGTAATGATGTAGCTTGGTACCAACAGAAGCCAGGGCAGTCTCCTAAACTGCTGATATACTATGCATCCAATCGCTACACTGGAGTCCCTGATCGCTTCACTGGCAGTGGATATGGGACGGATTTCACTTTCACCATCAGCACTGTGCAGGCTGAAGACCTGGCAGTTTATTTCTGTCAGCAGGATTATAGCTCTCCTCC

IGKV6-32*01_AJ	A/J	IGKV	MUSMUS IGKV6-32*01 F	100	AGTATTGTGATGACCCAGACTC<br>CCAAATTCCTGCTTGTATCAGCA<br>GGAGACAGGGTTACCATAACCT<br>GCAAGGCCAGTCAGAGTGTGA<br>GTAATGATGTAGCTTGGTACCAA<br>CAGAAGCCAGGGCAGTCTCCT<br>AAACTGCTGATATACTATGCATC<br>CAATCGCTACACTGGAGTCCCT<br>GATCGCTTCACTGGCAGTGGAT<br>ATGGGACGGATTTCACTTTCACC<br>ATCAGCACTGTGCAGGCTGAAG<br>ACCTGGCAGTTTATTTCTGTCAG<br>CAGGATTATAGCTCTCCTCC
IGKV6-32*01_AKR	AKR/J	IGKV	MUSMUS IGKV6-32*01 F	100	AGTATTGTGATGACCCAGACTC<br>CCAAATTCCTGCTTGTATCAGCA<br>GGAGACAGGGTTACCATAACCT<br>GCAAGGCCAGTCAGAGTGTGA<br>GTAATGATGTAGCTTGGTACCAA<br>CAGAAGCCAGGGCAGTCTCCT<br>AAACTGCTGATATACTATGCATC<br>CAATCGCTACACTGGAGTCCCT<br>GATCGCTTCACTGGCAGTGGAT<br>ATGGGACGGATTTCACTTTCACC<br>ATCAGCACTGTGCAGGCTGAAG<br>ACCTGGCAGTTTATTTCTGTCAG<br>CAGGATTATAGCTCTCCTCC
IGKV6-32*01_B6	C57BL/6J	IGKV	MUSMUS IGKV6-32*01 F	100	AGTATTGTGATGACCCAGACTC<br>CCAAATTCCTGCTTGTATCAGCA<br>GGAGACAGGGTTACCATAACCT<br>GCAAGGCCAGTCAGAGTGTGA<br>GTAATGATGTAGCTTGGTACCAA<br>CAGAAGCCAGGGCAGTCTCCT<br>AAACTGCTGATATACTATGCATC<br>CAATCGCTACACTGGAGTCCCT<br>GATCGCTTCACTGGCAGTGGAT<br>ATGGGACGGATTTCACTTTCACC<br>ATCAGCACTGTGCAGGCTGAAG<br>ACCTGGCAGTTTATTTCTGTCAG<br>CAGGATTATAGCTCTCCTCC
IGKV6-32*01_BALB	BALB/CBYJ	IGKV	MUSMUS IGKV6-32*01 F	100	AGTATTGTGATGACCCAGACTC<br>CCAAATTCCTGCTTGTATCAGCA<br>GGAGACAGGGTTACCATAACCT<br>GCAAGGCCAGTCAGAGTGTGA<br>GTAATGATGTAGCTTGGTACCAA<br>CAGAAGCCAGGGCAGTCTCCT<br>AAACTGCTGATATACTATGCATC<br>CAATCGCTACACTGGAGTCCCT<br>GATCGCTTCACTGGCAGTGGAT<br>ATGGGACGGATTTCACTTTCACC<br>ATCAGCACTGTGCAGGCTGAAG<br>ACCTGGCAGTTTATTTCTGTCAG<br>CAGGATTATAGCTCTCCTCC

IGKV6-32*01_C3H	C3H/HEJ	IGKV	MUSMUS IGKV6-32*01 F	100	AGTATTGTGATGACCCAGACTC CCAAATTCCTGCTTGTATCAGCA GGAGACAGGGTTACCATAACCT GCAAGGCCAGTCAGAGTGTGA GTAATGATGTAGCTTGGTACCAA CAGAAGCCAGGGCAGTCTCCT AAACTGCTGATATACTATGCATC CAATCGCTACACTGGAGTCCCT GATCGCTTCACTGGCAGTGGAT ATGGGACGGATTTCACTTTCACC ATCAGCACTGTGCAGGCTGAAG ACCTGGCAGTTTATTTCTGTCAG CAGGATTATAGCTCTCCTCC
IGKV6-32*01_CBA	CBA/J	IGKV	MUSMUS IGKV6-32*01 F	100	AGTATTGTGATGACCCAGACTC CCAAATTCCTGCTTGTATCAGCA GGAGACAGGGTTACCATAACCT GCAAGGCCAGTCAGAGTGTGA GTAATGATGTAGCTTGGTACCAA CAGAAGCCAGGGCAGTCTCCT AAACTGCTGATATACTATGCATC CAATCGCTACACTGGAGTCCCT GATCGCTTCACTGGCAGTGGAT ATGGGACGGATTTCACTTTCACC ATCAGCACTGTGCAGGCTGAAG ACCTGGCAGTTTATTTCTGTCAG CAGGATTATAGCTCTCCTCC
IGKV6-32*01_DBA1	DBA/1J	IGKV	MUSMUS IGKV6-32*01 F	100	AGTATTGTGATGACCCAGACTC CCAAATTCCTGCTTGTATCAGCA GGAGACAGGGTTACCATAACCT GCAAGGCCAGTCAGAGTGTGA GTAATGATGTAGCTTGGTACCAA CAGAAGCCAGGGCAGTCTCCT AAACTGCTGATATACTATGCATC CAATCGCTACACTGGAGTCCCT GATCGCTTCACTGGCAGTGGAT ATGGGACGGATTTCACTTTCACC ATCAGCACTGTGCAGGCTGAAG ACCTGGCAGTTTATTTCTGTCAG CAGGATTATAGCTCTCCTCC
IGKV6-32*01_DBA2	DBA/2J	IGKV	MUSMUS IGKV6-32*01 F	100	AGTATTGTGATGACCCAGACTC CCAAATTCCTGCTTGTATCAGCA GGAGACAGGGTTACCATAACCT GCAAGGCCAGTCAGAGTGTGA GTAATGATGTAGCTTGGTACCAA CAGAAGCCAGGGCAGTCTCCT AAACTGCTGATATACTATGCATC CAATCGCTACACTGGAGTCCCT GATCGCTTCACTGGCAGTGGAT ATGGGACGGATTTCACTTTCACC ATCAGCACTGTGCAGGCTGAAG ACCTGGCAGTTTATTTCTGTCAG CAGGATTATAGCTCTCCTCC

IGKV6-32*01_LEWES	LEWES/EIJ	IGKV	MUSMUS IGKV6-32*01 F	100	AGTATTGTGATGACCCAGACTCCCAAATTCCTGCTTGTATCAGCAGGAGACAGGGTTACCATAACCTGCAAGGCCAGTCAGAGTGTGAGTAATGATGTAGCTTGGTACCAACAGAAGCCAGGGCAGTCTCCTAAACTGCTGATATACTATGCATCCAATCGCTACACTGGAGTCCCTGATCGCTTCACTGGCAGTGGATATGGGACGGATTTCACTTTCACCATCAGCACTGTGCAGGCTGAAGACCTGGCAGTTTATTTCTGTCAGCAGGATTATAGCTCTCCTCC
IGKV6-32*01_NZB	NZB/BLNJ	IGKV	MUSMUS IGKV6-32*01 F	100	AGTATTGTGATGACCCAGACTCCCAAATTCCTGCTTGTATCAGCAGGAGACAGGGTTACCATAACCTGCAAGGCCAGTCAGAGTGTGAGTAATGATGTAGCTTGGTACCAACAGAAGCCAGGGCAGTCTCCTAAACTGCTGATATACTATGCATCCAATCGCTACACTGGAGTCCCTGATCGCTTCACTGGCAGTGGATATGGGACGGATTTCACTTTCACCATCAGCACTGTGCAGGCTGAAGACCTGGCAGTTTATTTCTGTCAGCAGGATTATAGCTCTCCTCC
IGKV6-32*01_SJL	SJL/J	IGKV	MUSMUS IGKV6-32*01 F	100	AGTATTGTGATGACCCAGACTCCCAAATTCCTGCTTGTATCAGCAGGAGACAGGGTTACCATAACCTGCAAGGCCAGTCAGAGTGTGAGTAATGATGTAGCTTGGTACCAACAGAAGCCAGGGCAGTCTCCTAAACTGCTGATATACTATGCATCCAATCGCTACACTGGAGTCCCTGATCGCTTCACTGGCAGTGGATATGGGACGGATTTCACTTTCACCATCAGCACTGTGCAGGCTGAAGACCTGGCAGTTTATTTCTGTCAGCAGGATTATAGCTCTCCTCC
IGKV6-B*01_AKR	AKR/J	IGKV	MUSMUS IGKV6-B*01 F	100	AGTATTGTGATGACCCAGACTCCCAAATTCCTGCCTGTATCAGCAGGAGACAGGGTTACCATGACCTGCAAGGCCAGTCAGAGTGTGGGTAATAATGTAGCCTGGTACCAACAGAAGCCAGGACAGTCTCCTAAACTGCTGATATACTATGCATCCAATCGCTACACTGGAGTCCCTGATCGCTTCACTGGCAGTGGATCTGGGACAGATTTCACTTTCACCATCAGCAGTGTGCAGGTTGAAGACCTGGCAGTTTATTTCTGTCAGCAGCATTATAGCTCTCCTCC

IGKV6-B*01_CAST	CAST/EIJ	IGKV	MUSMUS IGKV6-B*01 F	100	AGTATTGTGATGACCCAGACTCCCAAATTCCTGCCTGTATCAGCAGGAGACAGGGTTACCATGACCTGCAAGGCCAGTCAGAGTGTGGGTAATAATGTAGCCTGGTACCAACAGAAGCCAGGACAGTCTCCTAAACTGCTGATATACTATGCATCCAATCGCTACACTGGAGTCCCTGATCGCTTCACTGGCAGTGGATCTGGGACAGATTTCACTTTCACCATCAGCAGTGTGCAGGTTGAAGACCTGGCAGTTTATTTCTGTCAGCAGCATTATAGCTCTCCTCC
IGKV6-B*01_DBA1	DBA/1J	IGKV	MUSMUS IGKV6-B*01 F	100	AGTATTGTGATGACCCAGACTCCCAAATTCCTGCCTGTATCAGCAGGAGACAGGGTTACCATGACCTGCAAGGCCAGTCAGAGTGTGGGTAATAATGTAGCCTGGTACCAACAGAAGCCAGGACAGTCTCCTAAACTGCTGATATACTATGCATCCAATCGCTACACTGGAGTCCCTGATCGCTTCACTGGCAGTGGATCTGGGACAGATTTCACTTTCACCATCAGCAGTGTGCAGGTTGAAGACCTGGCAGTTTATTTCTGTCAGCAGCATTATAGCTCTCCTCC
IGKV6-B*01_MRL	MRL/MPJ	IGKV	MUSMUS IGKV6-B*01 F	100	AGTATTGTGATGACCCAGACTCCCAAATTCCTGCCTGTATCAGCAGGAGACAGGGTTACCATGACCTGCAAGGCCAGTCAGAGTGTGGGTAATAATGTAGCCTGGTACCAACAGAAGCCAGGACAGTCTCCTAAACTGCTGATATACTATGCATCCAATCGCTACACTGGAGTCCCTGATCGCTTCACTGGCAGTGGATCTGGGACAGATTTCACTTTCACCATCAGCAGTGTGCAGGTTGAAGACCTGGCAGTTTATTTCTGTCAGCAGCATTATAGCTCTCCTCC
IGKV6-B*01_NOD	NOD/SHILTJ	IGKV	MUSMUS IGKV6-B*01 F	100	AGTATTGTGATGACCCAGACTCCCAAATTCCTGCCTGTATCAGCAGGAGACAGGGTTACCATGACCTGCAAGGCCAGTCAGAGTGTGGGTAATAATGTAGCCTGGTACCAACAGAAGCCAGGACAGTCTCCTAAACTGCTGATATACTATGCATCCAATCGCTACACTGGAGTCCCTGATCGCTTCACTGGCAGTGGATCTGGGACAGATTTCACTTTCACCATCAGCAGTGTGCAGGTTGAAGACCTGGCAGTTTATTTCTGTCAGCAGCATTATAGCTCTCCTCC

IGKV6-B*01_NOR	NOR/LTJ	IGKV	MUSMUS IGKV6-B*01 F	100	AGTATTGTGATGACCCAGACTC<br>CCAAATTCCTGCCTGTATCAGC<br>AGGAGACAGGGTTACCATGACC<br>TGCAAGGCCAGTCAGAGTGTG<br>GGTAATAATGTAGCCTGGTACC<br>AACAGAAGCCAGGACAGTCTC<br>CTAAACTGCTGATATACTATGCA<br>TCCAATCGCTACACTGGAGTCC<br>CTGATCGCTTCACTGGCAGTGG<br>ATCTGGGACAGATTTCACTTTCA<br>CCATCAGCAGTGTGCAGGTTGA<br>AGACCTGGCAGTTTATTTCTGTC<br>AGCAGCATTATAGCTCTCCTCC
IGKV6-D*01_S0533_PWD	PWD/PHJ	IGKV	MUSMUS IGKV6-D*01 F	98.2	AGCATTGTGATGACCCAGTCTC<br>CCAAATCCCTGCCTGTATCAGC<br>AGGAGACAGGGTTACCATGACC<br>TGCAAGGCCAGTCAGAGTGTGA<br>GTAATGATGTAGCCTGGTACCA<br>ACAGAAGCCAGGGCAGTCTCC<br>TAAACTGGTGATATACTATGCAT<br>CCAATCGGTACACTGGAGTCCC<br>TGATTGCTTCACTGGCAGTGGAT<br>CTGGGACGGATTTCACTTTCAC<br>CAGCAGCAGTGTGCAGGCTGA<br>AGACCTGGCAGTTTATTTCTGTC<br>AGCAGCATTATACTACTCCTCC
IGKV7-33*01_B6	C57BL/6J	IGKV	MUSMUS IGKV7-33*01 F	100	GACATTGTGATGACTCAGTCTCC<br>AACTTTCCTTGCTGTGACAGCAA<br>GTAAGAAGGTCACCATTAGTTG<br>CACGGCCAGTGAGAGCCTTTAT<br>TCAAGCAAACACAAGGTGCACT<br>ACTTGGCTTGGTACCAGAAGAA<br>ACCAGAGCAATCTCCTAAACTG<br>CTGATATACGGGGCATCCAACC<br>GATACATTGGGGTCCCTGATCG<br>CTTCACAGGCAGTGGATCTGGG<br>ACAGATTTCACTCTGACCATCAG<br>CAGTGTACAGGTTGAAGACCTC<br>ACACATTATTACTGTGCACAGTT<br>TTACAGCTATCCTCT
IGKV7-33*01_BALB	BALB/CBYJ	IGKV	MUSMUS IGKV7-33*01 F	100	GACATTGTGATGACTCAGTCTCC<br>AACTTTCCTTGCTGTGACAGCAA<br>GTAAGAAGGTCACCATTAGTTG<br>CACGGCCAGTGAGAGCCTTTAT<br>TCAAGCAAACACAAGGTGCACT<br>ACTTGGCTTGGTACCAGAAGAA<br>ACCAGAGCAATCTCCTAAACTG<br>CTGATATACGGGGCATCCAACC<br>GATACATTGGGGTCCCTGATCG<br>CTTCACAGGCAGTGGATCTGGG<br>ACAGATTTCACTCTGACCATCAG<br>CAGTGTACAGGTTGAAGACCTC<br>ACACATTATTACTGTGCACAGTT<br>TTACAGCTATCCTCT

IGKV7-33*01_DBA1	DBA/1J	IGKV	MUSMUS IGKV7-33*01 F	100	GACATTGTGATGACTCAGTCTCCAACTTTCCTTGCTGTGACAGCAAGTAAGAAGGTCACCATTAGTTGCACGGCCAGTGAGAGCCTTTATTCAAGCAAACACAAGGTGCACTACTTGGCTTGGTACCAGAAGAAACCAGAGCAATCTCCTAAACTGCTGATATACGGGGCATCCAACCGATACATTGGGGTCCCTGATCGCTTCACAGGCAGTGGATCTGGGACAGATTTCACTCTGACCATCAGCAGTGTACAGGTTGAAGACCTCACACATTATTACTGTGCACAGTTTTACAGCTATCCTCT
IGKV7-33*01_DBA2	DBA/2J	IGKV	MUSMUS IGKV7-33*01 F	100	GACATTGTGATGACTCAGTCTCCAACTTTCCTTGCTGTGACAGCAAGTAAGAAGGTCACCATTAGTTGCACGGCCAGTGAGAGCCTTTATTCAAGCAAACACAAGGTGCACTACTTGGCTTGGTACCAGAAGAAACCAGAGCAATCTCCTAAACTGCTGATATACGGGGCATCCAACCGATACATTGGGGTCCCTGATCGCTTCACAGGCAGTGGATCTGGGACAGATTTCACTCTGACCATCAGCAGTGTACAGGTTGAAGACCTCACACATTATTACTGTGCACAGTTTTACAGCTATCCTCT
IGKV7-33*01_LEWES	LEWES/EIJ	IGKV	MUSMUS IGKV7-33*01 F	100	GACATTGTGATGACTCAGTCTCCAACTTTCCTTGCTGTGACAGCAAGTAAGAAGGTCACCATTAGTTGCACGGCCAGTGAGAGCCTTTATTCAAGCAAACACAAGGTGCACTACTTGGCTTGGTACCAGAAGAAACCAGAGCAATCTCCTAAACTGCTGATATACGGGGCATCCAACCGATACATTGGGGTCCCTGATCGCTTCACAGGCAGTGGATCTGGGACAGATTTCACTCTGACCATCAGCAGTGTACAGGTTGAAGACCTCACACATTATTACTGTGCACAGTTTTACAGCTATCCTCT
IGKV7-33*01_NZB	NZB/BLNJ	IGKV	MUSMUS IGKV7-33*01 F	100	GACATTGTGATGACTCAGTCTCCAACTTTCCTTGCTGTGACAGCAAGTAAGAAGGTCACCATTAGTTGCACGGCCAGTGAGAGCCTTTATTCAAGCAAACACAAGGTGCACTACTTGGCTTGGTACCAGAAGAAACCAGAGCAATCTCCTAAACTGCTGATATACGGGGCATCCAACCGATACATTGGGGTCCCTGATCGCTTCACAGGCAGTGGATCTGGGACAGATTTCACTCTGACCATCAGCAGTGTACAGGTTGAAGACCTCACACATTATTACTGTGCACAGTTTTACAGCTATCCTCT

IGKV7-33*01_S0872_MSM	MSM/MSJ	IGKV	MUSMUS IGKV7-33*01 F	97.64	GACATTGTGATGACTCAGTCTCCAACTTTCCTTGCTGTGACAGCAAGTAAGAAGGTCACCATTAGTTGCACGGCCAGTGAGAGCCTTTATTCAAGCAAACACAAGGTGAACTACTTGGCTTGGTACCAGAAGAAACCAGAGCAATCTCCTAAACTGCTGATTTATGGGGCATCCAACCGATTCACTGGGGTCCCTGATCGCTTCACAGGCAGTGGATCTGGGACAGATTTCACTCTGACCATCAGCAGTGTGCAGGCTGAAGACCTCACACATTATTACTGTGCACAGTTTTACAGCTATCCTCT
IGKV7-33*01_S2856_AKR	AKR/J	IGKV	MUSMUS IGKV7-33*01 F	97.31	GACATTGTGATGACTCAGTCTCCAACTTTCCTTGCTGTGACAGCAAGTAAGAAGGTCACCATTAGTTGCACGGCCAGTGAGAGCCTTTATTCAAGCAAACACAAGGTGAACTACTTAGCTTGGTACCAGAAGAAACCAGAGCAATCTCCTAAACTGCTGATTTATGGGGCATCCAACCGATTCACTGGGGTCCCTGATCGCTTCACAGGCAGTGGATCTGGGACAGATTTCACTCTGACCATCAGCAGTGTGCAGGCTGAAGACCTCACACATTATTACTGTGCACAGTTTTACAGCTATCCTCT
IGKV7-33*01_S2856_CAST	CAST/EIJ	IGKV	MUSMUS IGKV7-33*01 F	97.31	GACATTGTGATGACTCAGTCTCCAACTTTCCTTGCTGTGACAGCAAGTAAGAAGGTCACCATTAGTTGCACGGCCAGTGAGAGCCTTTATTCAAGCAAACACAAGGTGAACTACTTAGCTTGGTACCAGAAGAAACCAGAGCAATCTCCTAAACTGCTGATTTATGGGGCATCCAACCGATTCACTGGGGTCCCTGATCGCTTCACAGGCAGTGGATCTGGGACAGATTTCACTCTGACCATCAGCAGTGTGCAGGCTGAAGACCTCACACATTATTACTGTGCACAGTTTTACAGCTATCCTCT
IGKV7-33*01_S2856_MRL	MRL/MPJ	IGKV	MUSMUS IGKV7-33*01 F	97.31	GACATTGTGATGACTCAGTCTCCAACTTTCCTTGCTGTGACAGCAAGTAAGAAGGTCACCATTAGTTGCACGGCCAGTGAGAGCCTTTATTCAAGCAAACACAAGGTGAACTACTTAGCTTGGTACCAGAAGAAACCAGAGCAATCTCCTAAACTGCTGATTTATGGGGCATCCAACCGATTCACTGGGGTCCCTGATCGCTTCACAGGCAGTGGATCTGGGACAGATTTCACTCTGACCATCAGCAGTGTGCAGGCTGAAGACCTCACACATTATTACTGTGCACAGTTTTACAGCTATCCTCT

IGKV7-33*01_S2856_NOR	NOR/LTJ	IGKV	MUSMUS IGKV7-33*01 F	97.31	GACATTGTGATGACTCAGTCTCCAACTTTCCTTGCTGTGACAGCAAGTAAGAAGGTCACCATTAGTTGCACGGCCAGTGAGAGCCTTTATTCAAGCAAACACAAGGTGAACTACTTAGCTTGGTACCAGAAGAAACCAGAGCAATCTCCTAAACTGCTGATTTATGGGGCATCCAACCGATTCACTGGGGTCCCTGATCGCTTCACAGGCAGTGGATCTGGGACAGATTTCACTCTGACCATCAGCAGTGTGCAGGCTGAAGACCTCACACATTATTACTGTGCACAGTTTTACAGCTATCCTCT
IGKV7-33*01_S5071_PWD	PWD/PHJ	IGKV	MUSMUS IGKV7-33*01 F	99.66	GACATTGTGATGACTCAGTCTCCAACTTTCCTTGCTGTGACAGCAAGTAAGAAGGTCACCATTAGTTGCACGGCCAGTGAGAGCCTTTATTCAAGCAAACACAAGGTGCACTACTTGGCTTGGTACCAGAAGAAACCAGAGCAATCTCCTAAACTGCTGATATACGGGGCATCCAACCGATACATTGGGGTCCCTGATCGCTTCACAGGCAGTGGATCTGGGACAGATTTCACTCTGACCATCAGCAGTGTACAGGCTGAAGACCTCACACATTATTACTGTGCACAGTTTTACAGCTATCCTCT
IGKV7-33*01_S6039_129	129S1/SVIMJ	IGKV	MUSMUS IGKV7-33*01 F	100	GACATTGTGATGACTCAGTCTCCAACTTTCCTTGCTGTGACAGCAAGTAAGAAGGTCACCATTAGTTGCACGGCCAGTGAGAGCCTTTATTCAAGCAAACACAAGGTGCACTACTTGGCTTGGTACCAGAAGAAACCAGAGCAATCTCCTAAACTGCTGATATACGGGGCATCCAACCGATACATTGGGGTCCCTGATCGCTTCACAGGCAGTGGATCTGGGACAGATTTCACTCTGACCATCAGCAGTGTACAGGTTGAAGACCTCACACATTATTACTGTGCACAGTTTTACAGCTATCCGCT
IGKV7-33*01_S6039_AJ	A/J	IGKV	MUSMUS IGKV7-33*01 F	100	GACATTGTGATGACTCAGTCTCCAACTTTCCTTGCTGTGACAGCAAGTAAGAAGGTCACCATTAGTTGCACGGCCAGTGAGAGCCTTTATTCAAGCAAACACAAGGTGCACTACTTGGCTTGGTACCAGAAGAAACCAGAGCAATCTCCTAAACTGCTGATATACGGGGCATCCAACCGATACATTGGGGTCCCTGATCGCTTCACAGGCAGTGGATCTGGGACAGATTTCACTCTGACCATCAGCAGTGTACAGGTTGAAGACCTCACACATTATTACTGTGCACAGTTTTACAGCTATCCGCT

IGKV8-16*01_129	129S1/SVIMJ	IGKV	MUSMUS IGKV8-16*01 F	100	GAAATTGTGTTGACCCAGTCTATACCATCCCTGACTGTGTCAGCAGGAGAGAGGGTCACTATCAGCTGCAAATCCAATCAGAATCTTTTATGGAGTGGAAACCAAAGGTACTGTTTGGTCTGGCACCAGTGGAAACCAGGGCAAACTCCTACACCGTTGATCACCTGGACATCTGATAGGTACTCTGGAGTCCCTGATCGTTTCATAGGCAGTGGATCTGTGACAGATTTCACTCTGACCATCAGCAGTGTGCAGGCTGAAGATGTGGCAGTTTATTTCTGTCAGCAGCATTTACACATTCCTCC
IGKV8-16*01_AJ	A/J	IGKV	MUSMUS IGKV8-16*01 F	100	GAAATTGTGTTGACCCAGTCTATACCATCCCTGACTGTGTCAGCAGGAGAGAGGGTCACTATCAGCTGCAAATCCAATCAGAATCTTTTATGGAGTGGAAACCAAAGGTACTGTTTGGTCTGGCACCAGTGGAAACCAGGGCAAACTCCTACACCGTTGATCACCTGGACATCTGATAGGTACTCTGGAGTCCCTGATCGTTTCATAGGCAGTGGATCTGTGACAGATTTCACTCTGACCATCAGCAGTGTGCAGGCTGAAGATGTGGCAGTTTATTTCTGTCAGCAGCATTTACACATTCCTCC
IGKV8-16*01_B6	C57BL/6J	IGKV	MUSMUS IGKV8-16*01 F	100	GAAATTGTGTTGACCCAGTCTATACCATCCCTGACTGTGTCAGCAGGAGAGAGGGTCACTATCAGCTGCAAATCCAATCAGAATCTTTTATGGAGTGGAAACCAAAGGTACTGTTTGGTCTGGCACCAGTGGAAACCAGGGCAAACTCCTACACCGTTGATCACCTGGACATCTGATAGGTACTCTGGAGTCCCTGATCGTTTCATAGGCAGTGGATCTGTGACAGATTTCACTCTGACCATCAGCAGTGTGCAGGCTGAAGATGTGGCAGTTTATTTCTGTCAGCAGCATTTACACATTCCTCC
IGKV8-16*01_BALB	BALB/CBYJ	IGKV	MUSMUS IGKV8-16*01 F	100	GAAATTGTGTTGACCCAGTCTATACCATCCCTGACTGTGTCAGCAGGAGAGAGGGTCACTATCAGCTGCAAATCCAATCAGAATCTTTTATGGAGTGGAAACCAAAGGTACTGTTTGGTCTGGCACCAGTGGAAACCAGGGCAAACTCCTACACCGTTGATCACCTGGACATCTGATAGGTACTCTGGAGTCCCTGATCGTTTCATAGGCAGTGGATCTGTGACAGATTTCACTCTGACCATCAGCAGTGTGCAGGCTGAAGATGTGGCAGTTTATTTCTGTCAGCAGCATTTACACATTCCTCC

IGKV8-16*01_C3H	C3H/HEJ	IGKV	MUSMUS IGKV8-16*01 F	100	GAAATTGTGTTGACCCAGTCTATACCATCCCTGACTGTGTCAGCAGGAGAGAGGGTCACTATCAGCTGCAAATCCAATCAGAATCTTTTATGGAGTGGAAACCAAAGGTACTGTTTGGTCTGGCACCAGTGGAAACCAGGGCAAACTCCTACACCGTTGATCACCTGGACATCTGATAGGTACTCTGGAGTCCCTGATCGTTTCATAGGCAGTGGATCTGTGACAGATTTCACTCTGACCATCAGCAGTGTGCAGGCTGAAGATGTGGCAGTTTATTTCTGTCAGCAGCATTTACACATTCCTCC
IGKV8-16*01_CBA	CBA/J	IGKV	MUSMUS IGKV8-16*01 F	100	GAAATTGTGTTGACCCAGTCTATACCATCCCTGACTGTGTCAGCAGGAGAGAGGGTCACTATCAGCTGCAAATCCAATCAGAATCTTTTATGGAGTGGAAACCAAAGGTACTGTTTGGTCTGGCACCAGTGGAAACCAGGGCAAACTCCTACACCGTTGATCACCTGGACATCTGATAGGTACTCTGGAGTCCCTGATCGTTTCATAGGCAGTGGATCTGTGACAGATTTCACTCTGACCATCAGCAGTGTGCAGGCTGAAGATGTGGCAGTTTATTTCTGTCAGCAGCATTTACACATTCCTCC
IGKV8-16*01_DBA1	DBA/1J	IGKV	MUSMUS IGKV8-16*01 F	100	GAAATTGTGTTGACCCAGTCTATACCATCCCTGACTGTGTCAGCAGGAGAGAGGGTCACTATCAGCTGCAAATCCAATCAGAATCTTTTATGGAGTGGAAACCAAAGGTACTGTTTGGTCTGGCACCAGTGGAAACCAGGGCAAACTCCTACACCGTTGATCACCTGGACATCTGATAGGTACTCTGGAGTCCCTGATCGTTTCATAGGCAGTGGATCTGTGACAGATTTCACTCTGACCATCAGCAGTGTGCAGGCTGAAGATGTGGCAGTTTATTTCTGTCAGCAGCATTTACACATTCCTCC
IGKV8-16*01_DBA2	DBA/2J	IGKV	MUSMUS IGKV8-16*01 F	100	GAAATTGTGTTGACCCAGTCTATACCATCCCTGACTGTGTCAGCAGGAGAGAGGGTCACTATCAGCTGCAAATCCAATCAGAATCTTTTATGGAGTGGAAACCAAAGGTACTGTTTGGTCTGGCACCAGTGGAAACCAGGGCAAACTCCTACACCGTTGATCACCTGGACATCTGATAGGTACTCTGGAGTCCCTGATCGTTTCATAGGCAGTGGATCTGTGACAGATTTCACTCTGACCATCAGCAGTGTGCAGGCTGAAGATGTGGCAGTTTATTTCTGTCAGCAGCATTTACACATTCCTCC

IGKV8-16*01_LEWES	LEWES/EIJ	IGKV	MUSMUS IGKV8-16*01 F	100	GAAATTGTGTTGACCCAGTCTAT<br>ACCATCCCTGACTGTGTCAGCA<br>GGAGAGAGGGTCACTATCAGCT<br>GCAAATCCAATCAGAATCTTTTA<br>TGGAGTGGAAACCAAAGGTACT<br>GTTTGGTCTGGCACCAGTGGAA<br>ACCAGGGCAAACTCCTACACC<br>GTTGATCACCTGGACATCTGATA<br>GGTACTCTGGAGTCCCTGATCG<br>TTTCATAGGCAGTGGATCTGTGA<br>CAGATTTCACTCTGACCATCAG<br>CAGTGTGCAGGCTGAAGATGTG<br>GCAGTTTATTTCTGTCAGCAGCA<br>TTTACACATTCCTCC
IGKV8-16*01_NZB	NZB/BLNJ	IGKV	MUSMUS IGKV8-16*01 F	100	GAAATTGTGTTGACCCAGTCTAT<br>ACCATCCCTGACTGTGTCAGCA<br>GGAGAGAGGGTCACTATCAGCT<br>GCAAATCCAATCAGAATCTTTTA<br>TGGAGTGGAAACCAAAGGTACT<br>GTTTGGTCTGGCACCAGTGGAA<br>ACCAGGGCAAACTCCTACACC<br>GTTGATCACCTGGACATCTGATA<br>GGTACTCTGGAGTCCCTGATCG<br>TTTCATAGGCAGTGGATCTGTGA<br>CAGATTTCACTCTGACCATCAG<br>CAGTGTGCAGGCTGAAGATGTG<br>GCAGTTTATTTCTGTCAGCAGCA<br>TTTACACATTCCTCC
IGKV8-16*01_PWD	PWD/PHJ	IGKV	MUSMUS IGKV8-16*01 F	100	GAAATTGTGTTGACCCAGTCTAT<br>ACCATCCCTGACTGTGTCAGCA<br>GGAGAGAGGGTCACTATCAGCT<br>GCAAATCCAATCAGAATCTTTTA<br>TGGAGTGGAAACCAAAGGTACT<br>GTTTGGTCTGGCACCAGTGGAA<br>ACCAGGGCAAACTCCTACACC<br>GTTGATCACCTGGACATCTGATA<br>GGTACTCTGGAGTCCCTGATCG<br>TTTCATAGGCAGTGGATCTGTGA<br>CAGATTTCACTCTGACCATCAG<br>CAGTGTGCAGGCTGAAGATGTG<br>GCAGTTTATTTCTGTCAGCAGCA<br>TTTACACATTCCTCC
IGKV8-16*01_S1056_AKR	AKR/J	IGKV	MUSMUS IGKV8-16*01 F	98.99	GAAATTGTGTTGACACAGTCTAT<br>ACCATCCCTGACTGTGTCAGCA<br>GGAGAGAGGGTCACTATCAGCT<br>GCAAATCCAATCAGAATCTTTTA<br>TGGAGTGGAAACCAACGGTACT<br>GTTTGGTCTGGCACCAGTGGAA<br>ACCAGGGCAAACTCCTACACC<br>GTTGATCACCTGGACATCTGATA<br>GGTACTCTGGAGTCCCTGATCG<br>TTTCATAGGCAGTGGATCTGTGA<br>CAGATTTCACTCTGACCATCAG<br>CAGTGTGCAGGATGAAGATGTG<br>GCAGTTTATTTCTGTCAGCAGCA<br>TTTACACATTCCTCC

IGKV8-16*01_S1056_MRL	MRL/MPJ	IGKV	MUSMUS IGKV8-16*01 F	98.99	GAAATTGTGTTGACACAGTCTAT<br>ACCATCCCTGACTGTGTCAGCA<br>GGAGAGAGGGTCACTATCAGCT<br>GCAAATCCAATCAGAATCTTTTA<br>TGGAGTGGAAACCAACGGTACT<br>GTTTGGTCTGGCACCAGTGGAA<br>ACCAGGGCAAACTCCTACACC<br>GTTGATCACCTGGACATCTGATA<br>GGTACTCTGGAGTCCCTGATCG<br>TTTCATAGGCAGTGGATCTGTGA<br>CAGATTTCACTCTGACCATCAG<br>CAGTGTGCAGGATGAAGATGTG<br>GCAGTTTATTTCTGTCAGCAGCA<br>TTTACACATTCCTCC
IGKV8-16*01_S1056_NOR	NOR/LTJ	IGKV	MUSMUS IGKV8-16*01 F	98.99	GAAATTGTGTTGACACAGTCTAT<br>ACCATCCCTGACTGTGTCAGCA<br>GGAGAGAGGGTCACTATCAGCT<br>GCAAATCCAATCAGAATCTTTTA<br>TGGAGTGGAAACCAACGGTACT<br>GTTTGGTCTGGCACCAGTGGAA<br>ACCAGGGCAAACTCCTACACC<br>GTTGATCACCTGGACATCTGATA<br>GGTACTCTGGAGTCCCTGATCG<br>TTTCATAGGCAGTGGATCTGTGA<br>CAGATTTCACTCTGACCATCAG<br>CAGTGTGCAGGATGAAGATGTG<br>GCAGTTTATTTCTGTCAGCAGCA<br>TTTACACATTCCTCC
IGKV8-16*01_S4105_NOD	NOD/SHILTJ	IGKV	MUSMUS IGKV8-16*01 F	98.99	GAAATTGTGTTGACACAGTCTAT<br>ACCATCCCTGACTGTGTCAGCA<br>GGAGAGAGGGTCACTATCAGCT<br>GCAAATCCAATCAGAATCTTTTA<br>TGGAGTGGAAACCAACGGTACT<br>GTTTGGTCTGGCACCAGTGGAA<br>ACCAGGGCAAACTCCTACACC<br>GTTGATCACCTGGACATCTGATA<br>GGTACTCTGGAGTCCCTGATCG<br>TTTCATAGGCAGTGGATCTGTGA<br>CAGATTTCACTCTGACCATCAG<br>CAGTGTGCAGGATGAAGATGTG<br>GCAGTTTATTTCTGTCAGCAGCA<br>TTTACACATTCCTC
IGKV8-16*01_S7495_AKR	AKR/J	IGKV	MUSMUS IGKV8-16*01 F	99.33	GAAATTGTGTTGACGCAGTCTAT<br>ACCATCCCTGACTGTGTCAGCA<br>GGAGAGAGGGTCACTATCAGCT<br>GCAAATCCAATCAGAATCTTTTA<br>TGGAGTGGAAACCAAAGGTACT<br>GTTTGGTCTGGCACCAGTGGAA<br>ACCAGGGCAAACTCCTACACC<br>GTTGATCACCTGGACATCTGATA<br>GATACTCTGGAGTCCCTGATCG<br>TTTCATAGGCAGTGGATCTGTGA<br>CAGATTTCACTCTGACCATCAG<br>CAGTGTGCAGGCTGAAGATGTG<br>GCAGTTTATTTCTGTCAGCAGCA<br>TTTACACATTCCTCC

IGKV8-16*01_S7495_CAST	CAST/EIJ	IGKV	MUSMUS IGKV8-16*01 F	99.33	GAAATTGTGTTGACGCAGTCTATACCATCCCTGACTGTGTCAGCAGGAGAGAGGGTCACTATCAGCTGCAAATCCAATCAGAATCTTTTATGGAGTGGAAACCAAAGGTACTGTTTGGTCTGGCACCAGTGGAAACCAGGGCAAACTCCTACACCGTTGATCACCTGGACATCTGATAGATACTCTGGAGTCCCTGATCGTTTCATAGGCAGTGGATCTGTGACAGATTTCACTCTGACCATCAGCAGTGTGCAGGCTGAAGATGTGGCAGTTTATTTCTGTCAGCAGCATTTACACATTCCTCC
IGKV8-16*01_S7495_MRL	MRL/MPJ	IGKV	MUSMUS IGKV8-16*01 F	99.33	GAAATTGTGTTGACGCAGTCTATACCATCCCTGACTGTGTCAGCAGGAGAGAGGGTCACTATCAGCTGCAAATCCAATCAGAATCTTTTATGGAGTGGAAACCAAAGGTACTGTTTGGTCTGGCACCAGTGGAAACCAGGGCAAACTCCTACACCGTTGATCACCTGGACATCTGATAGATACTCTGGAGTCCCTGATCGTTTCATAGGCAGTGGATCTGTGACAGATTTCACTCTGACCATCAGCAGTGTGCAGGCTGAAGATGTGGCAGTTTATTTCTGTCAGCAGCATTTACACATTCCTCC
IGKV8-16*01_S7495_NOD	NOD/SHILTJ	IGKV	MUSMUS IGKV8-16*01 F	99.33	GAAATTGTGTTGACGCAGTCTATACCATCCCTGACTGTGTCAGCAGGAGAGAGGGTCACTATCAGCTGCAAATCCAATCAGAATCTTTTATGGAGTGGAAACCAAAGGTACTGTTTGGTCTGGCACCAGTGGAAACCAGGGCAAACTCCTACACCGTTGATCACCTGGACATCTGATAGATACTCTGGAGTCCCTGATCGTTTCATAGGCAGTGGATCTGTGACAGATTTCACTCTGACCATCAGCAGTGTGCAGGCTGAAGATGTGGCAGTTTATTTCTGTCAGCAGCATTTACACATTCCTCC
IGKV8-16*01_S7495_NOR	NOR/LTJ	IGKV	MUSMUS IGKV8-16*01 F	99.33	GAAATTGTGTTGACGCAGTCTATACCATCCCTGACTGTGTCAGCAGGAGAGAGGGTCACTATCAGCTGCAAATCCAATCAGAATCTTTTATGGAGTGGAAACCAAAGGTACTGTTTGGTCTGGCACCAGTGGAAACCAGGGCAAACTCCTACACCGTTGATCACCTGGACATCTGATAGATACTCTGGAGTCCCTGATCGTTTCATAGGCAGTGGATCTGTGACAGATTTCACTCTGACCATCAGCAGTGTGCAGGCTGAAGATGTGGCAGTTTATTTCTGTCAGCAGCATTTACACATTCCTCC

IGKV8-16*01_SJL	SJL/J	IGKV	MUSMUS IGKV8-16*01 F	100	GAAATTGTGTTGACCCAGTCTATACCATCCCTGACTGTGTCAGCAGGAGAGAGGGTCACTATCAGCTGCAAATCCAATCAGAATCTTTTATGGAGTGGAAACCAAAGGTACTGTTTGGTCTGGCACCAGTGGAAACCAGGGCAAACTCCTACACCGTTGATCACCTGGACATCTGATAGGTACTCTGGAGTCCCTGATCGTTTCATAGGCAGTGGATCTGTGACAGATTTCACTCTGACCATCAGCAGTGTGCAGGCTGAAGATGTGGCAGTTTATTTCTGTCAGCAGCATTTACACATTCCTCC
IGKV8-18*01_129	129S1/SVIMJ	IGKV	MUSMUS IGKV8-18*01 ORF	100	GACATTGTGATGACCCAGTCTCCATCCTCCCTGGCTGTGACAGCAGGAGAGAAGGTCACTATGAGATGCAAGTCCAGTCAGAGTCTTTTGTGGAGTGTAAACCAAAATAACTACTTATCCTGGTACCAGCAGAAACAAGGGCAGCCTCCTAAACTGCTTATCTATGGGGCATCCATTAGAGAATCTTGGGTCCCTGATCGATTCACAGGAAGTGGATCTGGGACAGACTTCACTCTCACCATTAGCAATGTGCATGCTGAAGACCTAGCAGTTTATTACTGTCAGCACAATCATGGCAGCTTTCTCCCCC
IGKV8-18*01_AJ	A/J	IGKV	MUSMUS IGKV8-18*01 ORF	100	GACATTGTGATGACCCAGTCTCCATCCTCCCTGGCTGTGACAGCAGGAGAGAAGGTCACTATGAGATGCAAGTCCAGTCAGAGTCTTTTGTGGAGTGTAAACCAAAATAACTACTTATCCTGGTACCAGCAGAAACAAGGGCAGCCTCCTAAACTGCTTATCTATGGGGCATCCATTAGAGAATCTTGGGTCCCTGATCGATTCACAGGAAGTGGATCTGGGACAGACTTCACTCTCACCATTAGCAATGTGCATGCTGAAGACCTAGCAGTTTATTACTGTCAGCACAATCATGGCAGCTTTCTCCCCC
IGKV8-18*01_B6	C57BL/6J	IGKV	MUSMUS IGKV8-18*01 ORF	100	GACATTGTGATGACCCAGTCTCCATCCTCCCTGGCTGTGACAGCAGGAGAGAAGGTCACTATGAGATGCAAGTCCAGTCAGAGTCTTTTGTGGAGTGTAAACCAAAATAACTACTTATCCTGGTACCAGCAGAAACAAGGGCAGCCTCCTAAACTGCTTATCTATGGGGCATCCATTAGAGAATCTTGGGTCCCTGATCGATTCACAGGAAGTGGATCTGGGACAGACTTCACTCTCACCATTAGCAATGTGCATGCTGAAGACCTAGCAGTTTATTACTGTCAGCACAATCATGGCAGCTTTCTCCCCC

IGKV8-18*01_BALB	BALB/CBYJ	IGKV	MUSMUS IGKV8-18*01 ORF	100	GACATTGTGATGACCCAGTCTCCATCCTCCCTGGCTGTGACAGCAGGAGAGAAGGTCACTATGAGATGCAAGTCCAGTCAGAGTCTTTTGTGGAGTGTAAACCAAAATAACTACTTATCCTGGTACCAGCAGAAACAAGGGCAGCCTCCTAAACTGCTTATCTATGGGGCATCCATTAGAGAATCTTGGGTCCCTGATCGATTCACAGGAAGTGGATCTGGGACAGACTTCACTCTCACCATTAGCAATGTGCATGCTGAAGACCTAGCAGTTTATTACTGTCAGCACAATCATGGCAGCTTTCTCCCCC
IGKV8-18*01_C3H	C3H/HEJ	IGKV	MUSMUS IGKV8-18*01 ORF	100	GACATTGTGATGACCCAGTCTCCATCCTCCCTGGCTGTGACAGCAGGAGAGAAGGTCACTATGAGATGCAAGTCCAGTCAGAGTCTTTTGTGGAGTGTAAACCAAAATAACTACTTATCCTGGTACCAGCAGAAACAAGGGCAGCCTCCTAAACTGCTTATCTATGGGGCATCCATTAGAGAATCTTGGGTCCCTGATCGATTCACAGGAAGTGGATCTGGGACAGACTTCACTCTCACCATTAGCAATGTGCATGCTGAAGACCTAGCAGTTTATTACTGTCAGCACAATCATGGCAGCTTTCTCCCCC
IGKV8-18*01_CBA	CBA/J	IGKV	MUSMUS IGKV8-18*01 ORF	100	GACATTGTGATGACCCAGTCTCCATCCTCCCTGGCTGTGACAGCAGGAGAGAAGGTCACTATGAGATGCAAGTCCAGTCAGAGTCTTTTGTGGAGTGTAAACCAAAATAACTACTTATCCTGGTACCAGCAGAAACAAGGGCAGCCTCCTAAACTGCTTATCTATGGGGCATCCATTAGAGAATCTTGGGTCCCTGATCGATTCACAGGAAGTGGATCTGGGACAGACTTCACTCTCACCATTAGCAATGTGCATGCTGAAGACCTAGCAGTTTATTACTGTCAGCACAATCATGGCAGCTTTCTCCCCC
IGKV8-18*01_DBA1	DBA/1J	IGKV	MUSMUS IGKV8-18*01 ORF	100	GACATTGTGATGACCCAGTCTCCATCCTCCCTGGCTGTGACAGCAGGAGAGAAGGTCACTATGAGATGCAAGTCCAGTCAGAGTCTTTTGTGGAGTGTAAACCAAAATAACTACTTATCCTGGTACCAGCAGAAACAAGGGCAGCCTCCTAAACTGCTTATCTATGGGGCATCCATTAGAGAATCTTGGGTCCCTGATCGATTCACAGGAAGTGGATCTGGGACAGACTTCACTCTCACCATTAGCAATGTGCATGCTGAAGACCTAGCAGTTTATTACTGTCAGCACAATCATGGCAGCTTTCTCCCCC

IGKV8-18*01_DBA2	DBA/2J	IGKV	MUSMUS IGKV8-18*01 ORF	100	GACATTGTGATGACCCAGTCTCCATCCTCCCTGGCTGTGACAGCAGGAGAGAAGGTCACTATGAGATGCAAGTCCAGTCAGAGTCTTTTGTGGAGTGTAAACCAAAATAACTACTTATCCTGGTACCAGCAGAAACAAGGGCAGCCTCCTAAACTGCTTATCTATGGGGCATCCATTAGAGAATCTTGGGTCCCTGATCGATTCACAGGAAGTGGATCTGGGACAGACTTCACTCTCACCATTAGCAATGTGCATGCTGAAGACCTAGCAGTTTATTACTGTCAGCACAATCATGGCAGCTTTCTCCCCC
IGKV8-18*01_NZB	NZB/BLNJ	IGKV	MUSMUS IGKV8-18*01 ORF	100	GACATTGTGATGACCCAGTCTCCATCCTCCCTGGCTGTGACAGCAGGAGAGAAGGTCACTATGAGATGCAAGTCCAGTCAGAGTCTTTTGTGGAGTGTAAACCAAAATAACTACTTATCCTGGTACCAGCAGAAACAAGGGCAGCCTCCTAAACTGCTTATCTATGGGGCATCCATTAGAGAATCTTGGGTCCCTGATCGATTCACAGGAAGTGGATCTGGGACAGACTTCACTCTCACCATTAGCAATGTGCATGCTGAAGACCTAGCAGTTTATTACTGTCAGCACAATCATGGCAGCTTTCTCCCCC
IGKV8-18*01_S2898_AKR	AKR/J	IGKV	MUSMUS IGKV8-18*01 ORF	99.66	GACATTGTGATGACCCAGTCTCCATCCTCCCTGGCTGTGACAGCAGGAGAGAAGGTCACTATGAGATGCAAGTCCAGTCAGAGTCTTTTGTGGAGTGTAAACCAAAAGAACTACTTATCCTGGTACCAGCAGAAACAAGGGCAGCCTCCTAAACTGCTTATCTATGGGGCATCCATTAGAGAATCTTGGGTCCCTGATCGATTCACAGGAAGTGGATCTGGGACAGACTTCACTCTCACCATTAGCAATGTGCATGCTGAAGACCTAGCAGTTTATTACTGTCAGCACAATCATGGCAGCTTTCTCCCCC
IGKV8-18*01_S2898_MSM	MSM/MSJ	IGKV	MUSMUS IGKV8-18*01 ORF	99.66	GACATTGTGATGACCCAGTCTCCATCCTCCCTGGCTGTGACAGCAGGAGAGAAGGTCACTATGAGATGCAAGTCCAGTCAGAGTCTTTTGTGGAGTGTAAACCAAAAGAACTACTTATCCTGGTACCAGCAGAAACAAGGGCAGCCTCCTAAACTGCTTATCTATGGGGCATCCATTAGAGAATCTTGGGTCCCTGATCGATTCACAGGAAGTGGATCTGGGACAGACTTCACTCTCACCATTAGCAATGTGCATGCTGAAGACCTAGCAGTTTATTACTGTCAGCACAATCATGGCAGCTTTCTCCCCC

IGKV8-18*01_S3987_PWD	PWD/PHJ	IGKV	MUSMUS IGKV8-18*01 ORF, OR MUSMUS IGKV8-26*01 ORF	97.98	GACATTGTGATGACCCAGTCTCCATCCTCCCTGGCTGTGACAGCAGGAGAGAAGGTCACTATGAGCTGCAAGTCCAGTCAGAGTCTTTTGTGGAGTGTAAACCAAAAGAACTACTTGTCCTGGTACCAGCAGAAACAAGGGAAGCCTCCTAAACTCCTTATCTATGGGGCATCCATTAGAGAATCTTGGGTCCCTGATCGATTCACAGGAAGTGGATCTGGGACAGACTTCACTCTCACCATTAGCAATGTGCATGCCGAAGACCTAGCAGTTTATTACTGTCAGCACAATCATGGCAGCTTTCTCCCCC
IGKV8-19*01_129	129S1/SVIMJ	IGKV	MUSMUS IGKV8-19*01 F	100	GACATTGTGATGACACAGTCTCCATCCTCCCTGACTGTGACAGCAGGAGAGAAGGTCACTATGAGCTGCAAGTCCAGTCAGAGTCTGTTAAACAGTGGAAATCAAAAGAACTACTTGACCTGGTACCAGCAGAAACCAGGGCAGCCTCCTAAACTGTTGATCTACTGGGCATCCACTAGGGAATCTGGGGTCCCTGATCGCTTCACAGGCAGTGGATCTGGAACAGATTTCACTCTCACCATCAGCAGTGTGCAGGCTGAAGACCTGGCAGTTTATTACTGTCAGAATGATTATAGTTATCCTCC
IGKV8-19*01_AJ	A/J	IGKV	MUSMUS IGKV8-19*01 F	100	GACATTGTGATGACACAGTCTCCATCCTCCCTGACTGTGACAGCAGGAGAGAAGGTCACTATGAGCTGCAAGTCCAGTCAGAGTCTGTTAAACAGTGGAAATCAAAAGAACTACTTGACCTGGTACCAGCAGAAACCAGGGCAGCCTCCTAAACTGTTGATCTACTGGGCATCCACTAGGGAATCTGGGGTCCCTGATCGCTTCACAGGCAGTGGATCTGGAACAGATTTCACTCTCACCATCAGCAGTGTGCAGGCTGAAGACCTGGCAGTTTATTACTGTCAGAATGATTATAGTTATCCTCC
IGKV8-19*01_AKR	AKR/J	IGKV	MUSMUS IGKV8-19*01 F	100	GACATTGTGATGACACAGTCTCCATCCTCCCTGACTGTGACAGCAGGAGAGAAGGTCACTATGAGCTGCAAGTCCAGTCAGAGTCTGTTAAACAGTGGAAATCAAAAGAACTACTTGACCTGGTACCAGCAGAAACCAGGGCAGCCTCCTAAACTGTTGATCTACTGGGCATCCACTAGGGAATCTGGGGTCCCTGATCGCTTCACAGGCAGTGGATCTGGAACAGATTTCACTCTCACCATCAGCAGTGTGCAGGCTGAAGACCTGGCAGTTTATTACTGTCAGAATGATTATAGTTATCCTCC

IGKV8-19*01_B6	C57BL/6J	IGKV	MUSMUS IGKV8-19*01 F	100	GACATTGTGATGACACAGTCTCCATCCTCCCTGACTGTGACAGCAGGAGAGAAGGTCACTATGAGCTGCAAGTCCAGTCAGAGTCTGTTAAACAGTGGAAATCAAAAGAACTACTTGACCTGGTACCAGCAGAAACCAGGGCAGCCTCCTAAACTGTTGATCTACTGGGCATCCACTAGGGAATCTGGGGTCCCTGATCGCTTCACAGGCAGTGGATCTGGAACAGATTTCACTCTCACCATCAGCAGTGTGCAGGCTGAAGACCTGGCAGTTTATTACTGTCAGAATGATTATAGTTATCCTCC
IGKV8-19*01_BALB	BALB/CBYJ	IGKV	MUSMUS IGKV8-19*01 F	100	GACATTGTGATGACACAGTCTCCATCCTCCCTGACTGTGACAGCAGGAGAGAAGGTCACTATGAGCTGCAAGTCCAGTCAGAGTCTGTTAAACAGTGGAAATCAAAAGAACTACTTGACCTGGTACCAGCAGAAACCAGGGCAGCCTCCTAAACTGTTGATCTACTGGGCATCCACTAGGGAATCTGGGGTCCCTGATCGCTTCACAGGCAGTGGATCTGGAACAGATTTCACTCTCACCATCAGCAGTGTGCAGGCTGAAGACCTGGCAGTTTATTACTGTCAGAATGATTATAGTTATCCTCC
IGKV8-19*01_C3H	C3H/HEJ	IGKV	MUSMUS IGKV8-19*01 F	100	GACATTGTGATGACACAGTCTCCATCCTCCCTGACTGTGACAGCAGGAGAGAAGGTCACTATGAGCTGCAAGTCCAGTCAGAGTCTGTTAAACAGTGGAAATCAAAAGAACTACTTGACCTGGTACCAGCAGAAACCAGGGCAGCCTCCTAAACTGTTGATCTACTGGGCATCCACTAGGGAATCTGGGGTCCCTGATCGCTTCACAGGCAGTGGATCTGGAACAGATTTCACTCTCACCATCAGCAGTGTGCAGGCTGAAGACCTGGCAGTTTATTACTGTCAGAATGATTATAGTTATCCTCC
IGKV8-19*01_CAST	CAST/EIJ	IGKV	MUSMUS IGKV8-19*01 F	100	GACATTGTGATGACACAGTCTCCATCCTCCCTGACTGTGACAGCAGGAGAGAAGGTCACTATGAGCTGCAAGTCCAGTCAGAGTCTGTTAAACAGTGGAAATCAAAAGAACTACTTGACCTGGTACCAGCAGAAACCAGGGCAGCCTCCTAAACTGTTGATCTACTGGGCATCCACTAGGGAATCTGGGGTCCCTGATCGCTTCACAGGCAGTGGATCTGGAACAGATTTCACTCTCACCATCAGCAGTGTGCAGGCTGAAGACCTGGCAGTTTATTACTGTCAGAATGATTATAGTTATCCTCC

IGKV8-19*01_CBA	CBA/J	IGKV	MUSMUS IGKV8-19*01 F	100	GACATTGTGATGACACAGTCTCCATCCTCCCTGACTGTGACAGCAGGAGAGAAGGTCACTATGAGCTGCAAGTCCAGTCAGAGTCTGTTAAACAGTGGAAATCAAAAGAACTACTTGACCTGGTACCAGCAGAAACCAGGGCAGCCTCCTAAACTGTTGATCTACTGGGCATCCACTAGGGAATCTGGGGTCCCTGATCGCTTCACAGGCAGTGGATCTGGAACAGATTTCACTCTCACCATCAGCAGTGTGCAGGCTGAAGACCTGGCAGTTTATTACTGTCAGAATGATTATAGTTATCCTCC
IGKV8-19*01_DBA1	DBA/1J	IGKV	MUSMUS IGKV8-19*01 F	100	GACATTGTGATGACACAGTCTCCATCCTCCCTGACTGTGACAGCAGGAGAGAAGGTCACTATGAGCTGCAAGTCCAGTCAGAGTCTGTTAAACAGTGGAAATCAAAAGAACTACTTGACCTGGTACCAGCAGAAACCAGGGCAGCCTCCTAAACTGTTGATCTACTGGGCATCCACTAGGGAATCTGGGGTCCCTGATCGCTTCACAGGCAGTGGATCTGGAACAGATTTCACTCTCACCATCAGCAGTGTGCAGGCTGAAGACCTGGCAGTTTATTACTGTCAGAATGATTATAGTTATCCTCC
IGKV8-19*01_DBA2	DBA/2J	IGKV	MUSMUS IGKV8-19*01 F	100	GACATTGTGATGACACAGTCTCCATCCTCCCTGACTGTGACAGCAGGAGAGAAGGTCACTATGAGCTGCAAGTCCAGTCAGAGTCTGTTAAACAGTGGAAATCAAAAGAACTACTTGACCTGGTACCAGCAGAAACCAGGGCAGCCTCCTAAACTGTTGATCTACTGGGCATCCACTAGGGAATCTGGGGTCCCTGATCGCTTCACAGGCAGTGGATCTGGAACAGATTTCACTCTCACCATCAGCAGTGTGCAGGCTGAAGACCTGGCAGTTTATTACTGTCAGAATGATTATAGTTATCCTCC
IGKV8-19*01_LEWES	LEWES/EIJ	IGKV	MUSMUS IGKV8-19*01 F	100	GACATTGTGATGACACAGTCTCCATCCTCCCTGACTGTGACAGCAGGAGAGAAGGTCACTATGAGCTGCAAGTCCAGTCAGAGTCTGTTAAACAGTGGAAATCAAAAGAACTACTTGACCTGGTACCAGCAGAAACCAGGGCAGCCTCCTAAACTGTTGATCTACTGGGCATCCACTAGGGAATCTGGGGTCCCTGATCGCTTCACAGGCAGTGGATCTGGAACAGATTTCACTCTCACCATCAGCAGTGTGCAGGCTGAAGACCTGGCAGTTTATTACTGTCAGAATGATTATAGTTATCCTCC

IGKV8-19*01_MRL	MRL/MPJ	IGKV	MUSMUS IGKV8-19*01 F	100	GACATTGTGATGACACAGTCTCCATCCTCCCTGACTGTGACAGCAGGAGAGAAGGTCACTATGAGCTGCAAGTCCAGTCAGAGTCTGTTAAACAGTGGAAATCAAAAGAACTACTTGACCTGGTACCAGCAGAAACCAGGGCAGCCTCCTAAACTGTTGATCTACTGGGCATCCACTAGGGAATCTGGGGTCCCTGATCGCTTCACAGGCAGTGGATCTGGAACAGATTTCACTCTCACCATCAGCAGTGTGCAGGCTGAAGACCTGGCAGTTTATTACTGTCAGAATGATTATAGTTATCCTCC
IGKV8-19*01_MSM	MSM/MSJ	IGKV	MUSMUS IGKV8-19*01 F	100	GACATTGTGATGACACAGTCTCCATCCTCCCTGACTGTGACAGCAGGAGAGAAGGTCACTATGAGCTGCAAGTCCAGTCAGAGTCTGTTAAACAGTGGAAATCAAAAGAACTACTTGACCTGGTACCAGCAGAAACCAGGGCAGCCTCCTAAACTGTTGATCTACTGGGCATCCACTAGGGAATCTGGGGTCCCTGATCGCTTCACAGGCAGTGGATCTGGAACAGATTTCACTCTCACCATCAGCAGTGTGCAGGCTGAAGACCTGGCAGTTTATTACTGTCAGAATGATTATAGTTATCCTCC
IGKV8-19*01_NOD	NOD/SHILTJ	IGKV	MUSMUS IGKV8-19*01 F	100	GACATTGTGATGACACAGTCTCCATCCTCCCTGACTGTGACAGCAGGAGAGAAGGTCACTATGAGCTGCAAGTCCAGTCAGAGTCTGTTAAACAGTGGAAATCAAAAGAACTACTTGACCTGGTACCAGCAGAAACCAGGGCAGCCTCCTAAACTGTTGATCTACTGGGCATCCACTAGGGAATCTGGGGTCCCTGATCGCTTCACAGGCAGTGGATCTGGAACAGATTTCACTCTCACCATCAGCAGTGTGCAGGCTGAAGACCTGGCAGTTTATTACTGTCAGAATGATTATAGTTATCCTCC
IGKV8-19*01_NOR	NOR/LTJ	IGKV	MUSMUS IGKV8-19*01 F	100	GACATTGTGATGACACAGTCTCCATCCTCCCTGACTGTGACAGCAGGAGAGAAGGTCACTATGAGCTGCAAGTCCAGTCAGAGTCTGTTAAACAGTGGAAATCAAAAGAACTACTTGACCTGGTACCAGCAGAAACCAGGGCAGCCTCCTAAACTGTTGATCTACTGGGCATCCACTAGGGAATCTGGGGTCCCTGATCGCTTCACAGGCAGTGGATCTGGAACAGATTTCACTCTCACCATCAGCAGTGTGCAGGCTGAAGACCTGGCAGTTTATTACTGTCAGAATGATTATAGTTATCCTCC

IGKV8-19*01_NZB	NZB/BLNJ	IGKV	MUSMUS IGKV8-19*01 F	100	GACATTGTGATGACACAGTCTCCATCCTCCCTGACTGTGACAGCAGGAGAGAAGGTCACTATGAGCTGCAAGTCCAGTCAGAGTCTGTTAAACAGTGGAAATCAAAAGAACTACTTGACCTGGTACCAGCAGAAACCAGGGCAGCCTCCTAAACTGTTGATCTACTGGGCATCCACTAGGGAATCTGGGGTCCCTGATCGCTTCACAGGCAGTGGATCTGGAACAGATTTCACTCTCACCATCAGCAGTGTGCAGGCTGAAGACCTGGCAGTTTATTACTGTCAGAATGATTATAGTTATCCTCC
IGKV8-19*01_SJL	SJL/J	IGKV	MUSMUS IGKV8-19*01 F	100	GACATTGTGATGACACAGTCTCCATCCTCCCTGACTGTGACAGCAGGAGAGAAGGTCACTATGAGCTGCAAGTCCAGTCAGAGTCTGTTAAACAGTGGAAATCAAAAGAACTACTTGACCTGGTACCAGCAGAAACCAGGGCAGCCTCCTAAACTGTTGATCTACTGGGCATCCACTAGGGAATCTGGGGTCCCTGATCGCTTCACAGGCAGTGGATCTGGAACAGATTTCACTCTCACCATCAGCAGTGTGCAGGCTGAAGACCTGGCAGTTTATTACTGTCAGAATGATTATAGTTATCCTCC
IGKV8-21*01_129	129S1/SVIMJ	IGKV	MUSMUS IGKV8-21*01 F	100	GACATTGTGATGTCACAGTCTCCATCCTCCCTGGCTGTGTCAGCAGGAGAGAAGGTCACTATGAGCTGCAAATCCAGTCAGAGTCTGCTCAACAGTAGAACCCGAAAGAACTACTTGGCTTGGTACCAGCAGAAACCAGGGCAGTCTCCTAAACTGCTGATCTACTGGGCATCCACTAGGGAATCTGGGGTCCCTGATCGCTTCACAGGCAGTGGATCTGGGACAGATTTCACTCTCACCATCAGCAGTGTGCAGGCTGAAGACCTGGCAGTTTATTACTGCAAGCAATCTTATAATCTTCC
IGKV8-21*01_AJ	A/J	IGKV	MUSMUS IGKV8-21*01 F	100	GACATTGTGATGTCACAGTCTCCATCCTCCCTGGCTGTGTCAGCAGGAGAGAAGGTCACTATGAGCTGCAAATCCAGTCAGAGTCTGCTCAACAGTAGAACCCGAAAGAACTACTTGGCTTGGTACCAGCAGAAACCAGGGCAGTCTCCTAAACTGCTGATCTACTGGGCATCCACTAGGGAATCTGGGGTCCCTGATCGCTTCACAGGCAGTGGATCTGGGACAGATTTCACTCTCACCATCAGCAGTGTGCAGGCTGAAGACCTGGCAGTTTATTACTGCAAGCAATCTTATAATCTTCC

IGKV8-21*01_B6	C57BL/6J	IGKV	MUSMUS IGKV8-21*01 F	100	GACATTGTGATGTCACAGTCTCCATCCTCCCTGGCTGTGTCAGCAGGAGAGAAGGTCACTATGAGCTGCAAATCCAGTCAGAGTCTGCTCAACAGTAGAACCCGAAAGAACTACTTGGCTTGGTACCAGCAGAAACCAGGGCAGTCTCCTAAACTGCTGATCTACTGGGCATCCACTAGGGAATCTGGGGTCCCTGATCGCTTCACAGGCAGTGGATCTGGGACAGATTTCACTCTCACCATCAGCAGTGTGCAGGCTGAAGACCTGGCAGTTTATTACTGCAAGCAATCTTATAATCTTCC
IGKV8-21*01_BALB	BALB/CBYJ	IGKV	MUSMUS IGKV8-21*01 F	100	GACATTGTGATGTCACAGTCTCCATCCTCCCTGGCTGTGTCAGCAGGAGAGAAGGTCACTATGAGCTGCAAATCCAGTCAGAGTCTGCTCAACAGTAGAACCCGAAAGAACTACTTGGCTTGGTACCAGCAGAAACCAGGGCAGTCTCCTAAACTGCTGATCTACTGGGCATCCACTAGGGAATCTGGGGTCCCTGATCGCTTCACAGGCAGTGGATCTGGGACAGATTTCACTCTCACCATCAGCAGTGTGCAGGCTGAAGACCTGGCAGTTTATTACTGCAAGCAATCTTATAATCTTCC
IGKV8-21*01_C3H	C3H/HEJ	IGKV	MUSMUS IGKV8-21*01 F	100	GACATTGTGATGTCACAGTCTCCATCCTCCCTGGCTGTGTCAGCAGGAGAGAAGGTCACTATGAGCTGCAAATCCAGTCAGAGTCTGCTCAACAGTAGAACCCGAAAGAACTACTTGGCTTGGTACCAGCAGAAACCAGGGCAGTCTCCTAAACTGCTGATCTACTGGGCATCCACTAGGGAATCTGGGGTCCCTGATCGCTTCACAGGCAGTGGATCTGGGACAGATTTCACTCTCACCATCAGCAGTGTGCAGGCTGAAGACCTGGCAGTTTATTACTGCAAGCAATCTTATAATCTTCC
IGKV8-21*01_CBA	CBA/J	IGKV	MUSMUS IGKV8-21*01 F	100	GACATTGTGATGTCACAGTCTCCATCCTCCCTGGCTGTGTCAGCAGGAGAGAAGGTCACTATGAGCTGCAAATCCAGTCAGAGTCTGCTCAACAGTAGAACCCGAAAGAACTACTTGGCTTGGTACCAGCAGAAACCAGGGCAGTCTCCTAAACTGCTGATCTACTGGGCATCCACTAGGGAATCTGGGGTCCCTGATCGCTTCACAGGCAGTGGATCTGGGACAGATTTCACTCTCACCATCAGCAGTGTGCAGGCTGAAGACCTGGCAGTTTATTACTGCAAGCAATCTTATAATCTTCC

IGKV8-21*01_DBA1	DBA/1J	IGKV	MUSMUS IGKV8-21*01 F	100	GACATTGTGATGTCACAGTCTCCATCCTCCCTGGCTGTGTCAGCAGGAGAGAAGGTCACTATGAGCTGCAAATCCAGTCAGAGTCTGCTCAACAGTAGAACCCGAAAGAACTACTTGGCTTGGTACCAGCAGAAACCAGGGCAGTCTCCTAAACTGCTGATCTACTGGGCATCCACTAGGGAATCTGGGGTCCCTGATCGCTTCACAGGCAGTGGATCTGGGACAGATTTCACTCTCACCATCAGCAGTGTGCAGGCTGAAGACCTGGCAGTTTATTACTGCAAGCAATCTTATAATCTTCC
IGKV8-21*01_DBA2	DBA/2J	IGKV	MUSMUS IGKV8-21*01 F	100	GACATTGTGATGTCACAGTCTCCATCCTCCCTGGCTGTGTCAGCAGGAGAGAAGGTCACTATGAGCTGCAAATCCAGTCAGAGTCTGCTCAACAGTAGAACCCGAAAGAACTACTTGGCTTGGTACCAGCAGAAACCAGGGCAGTCTCCTAAACTGCTGATCTACTGGGCATCCACTAGGGAATCTGGGGTCCCTGATCGCTTCACAGGCAGTGGATCTGGGACAGATTTCACTCTCACCATCAGCAGTGTGCAGGCTGAAGACCTGGCAGTTTATTACTGCAAGCAATCTTATAATCTTCC
IGKV8-21*01_LEWES	LEWES/EIJ	IGKV	MUSMUS IGKV8-21*01 F	100	GACATTGTGATGTCACAGTCTCCATCCTCCCTGGCTGTGTCAGCAGGAGAGAAGGTCACTATGAGCTGCAAATCCAGTCAGAGTCTGCTCAACAGTAGAACCCGAAAGAACTACTTGGCTTGGTACCAGCAGAAACCAGGGCAGTCTCCTAAACTGCTGATCTACTGGGCATCCACTAGGGAATCTGGGGTCCCTGATCGCTTCACAGGCAGTGGATCTGGGACAGATTTCACTCTCACCATCAGCAGTGTGCAGGCTGAAGACCTGGCAGTTTATTACTGCAAGCAATCTTATAATCTTCC
IGKV8-21*01_NZB	NZB/BLNJ	IGKV	MUSMUS IGKV8-21*01 F	100	GACATTGTGATGTCACAGTCTCCATCCTCCCTGGCTGTGTCAGCAGGAGAGAAGGTCACTATGAGCTGCAAATCCAGTCAGAGTCTGCTCAACAGTAGAACCCGAAAGAACTACTTGGCTTGGTACCAGCAGAAACCAGGGCAGTCTCCTAAACTGCTGATCTACTGGGCATCCACTAGGGAATCTGGGGTCCCTGATCGCTTCACAGGCAGTGGATCTGGGACAGATTTCACTCTCACCATCAGCAGTGTGCAGGCTGAAGACCTGGCAGTTTATTACTGCAAGCAATCTTATAATCTTCC

IGKV8-21*01_S1419_AKR	AKR/J	IGKV	MUSMUS IGKV8-21*01 F	99.33	GACATTGTGATGTCACAGTCTCCATCCTCCCTGGCTGTGTCAGCAGGAGAGAAGGTCACTATGAGCTGCAAATCCAGTCAGAGTCTGTTCAACAGTAGAACCCGAAAGAACTACTTGGCTTGGTACCAGCAGAAACCAGGGCAGTCTCCTAAACTGCTGATCTACTGGGCATCCACTAGGGAATCTGGGGTCCCTGATCGCTTCACAGGCAGTGGATCTGGGACAGATTTCACTCTCACCATCAGCAGTGTGCAGGCTGAAGACCTGGCAGTTTATTACTGCAAGCAATCTTATTATCTTCC
IGKV8-21*01_S1419_CAST	CAST/EIJ	IGKV	MUSMUS IGKV8-21*01 F	99.33	GACATTGTGATGTCACAGTCTCCATCCTCCCTGGCTGTGTCAGCAGGAGAGAAGGTCACTATGAGCTGCAAATCCAGTCAGAGTCTGTTCAACAGTAGAACCCGAAAGAACTACTTGGCTTGGTACCAGCAGAAACCAGGGCAGTCTCCTAAACTGCTGATCTACTGGGCATCCACTAGGGAATCTGGGGTCCCTGATCGCTTCACAGGCAGTGGATCTGGGACAGATTTCACTCTCACCATCAGCAGTGTGCAGGCTGAAGACCTGGCAGTTTATTACTGCAAGCAATCTTATTATCTTCC
IGKV8-21*01_S1419_MRL	MRL/MPJ	IGKV	MUSMUS IGKV8-21*01 F	99.33	GACATTGTGATGTCACAGTCTCCATCCTCCCTGGCTGTGTCAGCAGGAGAGAAGGTCACTATGAGCTGCAAATCCAGTCAGAGTCTGTTCAACAGTAGAACCCGAAAGAACTACTTGGCTTGGTACCAGCAGAAACCAGGGCAGTCTCCTAAACTGCTGATCTACTGGGCATCCACTAGGGAATCTGGGGTCCCTGATCGCTTCACAGGCAGTGGATCTGGGACAGATTTCACTCTCACCATCAGCAGTGTGCAGGCTGAAGACCTGGCAGTTTATTACTGCAAGCAATCTTATTATCTTCC
IGKV8-21*01_S1419_NOD	NOD/SHILTJ	IGKV	MUSMUS IGKV8-21*01 F	99.33	GACATTGTGATGTCACAGTCTCCATCCTCCCTGGCTGTGTCAGCAGGAGAGAAGGTCACTATGAGCTGCAAATCCAGTCAGAGTCTGTTCAACAGTAGAACCCGAAAGAACTACTTGGCTTGGTACCAGCAGAAACCAGGGCAGTCTCCTAAACTGCTGATCTACTGGGCATCCACTAGGGAATCTGGGGTCCCTGATCGCTTCACAGGCAGTGGATCTGGGACAGATTTCACTCTCACCATCAGCAGTGTGCAGGCTGAAGACCTGGCAGTTTATTACTGCAAGCAATCTTATTATCTTCC

IGKV8-21*01_S1419_NOR	NOR/LTJ	IGKV	MUSMUS IGKV8-21*01 F	99.33	GACATTGTGATGTCACAGTCTCCATCCTCCCTGGCTGTGTCAGCAGGAGAGAAGGTCACTATGAGCTGCAAATCCAGTCAGAGTCTGTTCAACAGTAGAACCCGAAAGAACTACTTGGCTTGGTACCAGCAGAAACCAGGGCAGTCTCCTAAACTGCTGATCTACTGGGCATCCACTAGGGAATCTGGGGTCCCTGATCGCTTCACAGGCAGTGGATCTGGGACAGATTTCACTCTCACCATCAGCAGTGTGCAGGCTGAAGACCTGGCAGTTTATTACTGCAAGCAATCTTATTATCTTCC
IGKV8-21*01_SJL	SJL/J	IGKV	MUSMUS IGKV8-21*01 F	100	GACATTGTGATGTCACAGTCTCCATCCTCCCTGGCTGTGTCAGCAGGAGAGAAGGTCACTATGAGCTGCAAATCCAGTCAGAGTCTGCTCAACAGTAGAACCCGAAAGAACTACTTGGCTTGGTACCAGCAGAAACCAGGGCAGTCTCCTAAACTGCTGATCTACTGGGCATCCACTAGGGAATCTGGGGTCCCTGATCGCTTCACAGGCAGTGGATCTGGGACAGATTTCACTCTCACCATCAGCAGTGTGCAGGCTGAAGACCTGGCAGTTTATTACTGCAAGCAATCTTATAATCTTCC
IGKV8-23-1*01_129	129S1/SVIMJ	IGKV	MUSMUS IGKV8-16*01 F, OR MUSMUS IGKV8-23-1*01 ORF	100	GAAATTGTGCTGACTGTGTCAGCAGGAGAGAGGGTCACTATCAGCTGCAAATCCAATCAGAATCTTTTATGGAGTGGAAACCAAAGGTACTGTTTGGTCTGGCACCAGTGGAAACCAGGGCAAACTCCTACACCGTTGATCACCTGGACATCTGATAGGTACTCTGGAGTCCCTGATCGTTTCATAGGCAGTGGATCTGTGACAGATTTCACTCTGACCATCAGCAGTGTGCAGGCTGAAGATGTGGCAGTTTATTTCTGTCAGCAGCATTTACACATTCCTCC
IGKV8-23-1*01_AJ	A/J	IGKV	MUSMUS IGKV8-16*01 F, OR MUSMUS IGKV8-23-1*01 ORF	100	GAAATTGTGCTGACTGTGTCAGCAGGAGAGAGGGTCACTATCAGCTGCAAATCCAATCAGAATCTTTTATGGAGTGGAAACCAAAGGTACTGTTTGGTCTGGCACCAGTGGAAACCAGGGCAAACTCCTACACCGTTGATCACCTGGACATCTGATAGGTACTCTGGAGTCCCTGATCGTTTCATAGGCAGTGGATCTGTGACAGATTTCACTCTGACCATCAGCAGTGTGCAGGCTGAAGATGTGGCAGTTTATTTCTGTCAGCAGCATTTACACATTCCTCC

IGKV8-23-1*01_B6	C57BL/6J	IGKV	MUSMUS IGKV8-16*01 F, OR MUSMUS IGKV8-23-1*01 ORF	100	GAAATTGTGCTGACTGTGTCAGCAGGAGAGAGGGTCACTATCAGCTGCAAATCCAATCAGAATCTTTTATGGAGTGGAAACCAAAGGTACTGTTTGGTCTGGCACCAGTGGAAACCAGGGCAAACTCCTACACCGTTGATCACCTGGACATCTGATAGGTACTCTGGAGTCCCTGATCGTTTCATAGGCAGTGGATCTGTGACAGATTTCACTCTGACCATCAGCAGTGTGCAGGCTGAAGATGTGGCAGTTTATTTCTGTCAGCAGCATTTACACATTCCTCC
IGKV8-23-1*01_BALB	BALB/CBYJ	IGKV	MUSMUS IGKV8-16*01 F, OR MUSMUS IGKV8-23-1*01 ORF	100	GAAATTGTGCTGACTGTGTCAGCAGGAGAGAGGGTCACTATCAGCTGCAAATCCAATCAGAATCTTTTATGGAGTGGAAACCAAAGGTACTGTTTGGTCTGGCACCAGTGGAAACCAGGGCAAACTCCTACACCGTTGATCACCTGGACATCTGATAGGTACTCTGGAGTCCCTGATCGTTTCATAGGCAGTGGATCTGTGACAGATTTCACTCTGACCATCAGCAGTGTGCAGGCTGAAGATGTGGCAGTTTATTTCTGTCAGCAGCATTTACACATTCCTCC
IGKV8-23-1*01_C3H	C3H/HEJ	IGKV	MUSMUS IGKV8-16*01 F, OR MUSMUS IGKV8-23-1*01 ORF	100	GAAATTGTGCTGACTGTGTCAGCAGGAGAGAGGGTCACTATCAGCTGCAAATCCAATCAGAATCTTTTATGGAGTGGAAACCAAAGGTACTGTTTGGTCTGGCACCAGTGGAAACCAGGGCAAACTCCTACACCGTTGATCACCTGGACATCTGATAGGTACTCTGGAGTCCCTGATCGTTTCATAGGCAGTGGATCTGTGACAGATTTCACTCTGACCATCAGCAGTGTGCAGGCTGAAGATGTGGCAGTTTATTTCTGTCAGCAGCATTTACACATTCCTCC
IGKV8-23-1*01_CBA	CBA/J	IGKV	MUSMUS IGKV8-16*01 F, OR MUSMUS IGKV8-23-1*01 ORF	100	GAAATTGTGCTGACTGTGTCAGCAGGAGAGAGGGTCACTATCAGCTGCAAATCCAATCAGAATCTTTTATGGAGTGGAAACCAAAGGTACTGTTTGGTCTGGCACCAGTGGAAACCAGGGCAAACTCCTACACCGTTGATCACCTGGACATCTGATAGGTACTCTGGAGTCCCTGATCGTTTCATAGGCAGTGGATCTGTGACAGATTTCACTCTGACCATCAGCAGTGTGCAGGCTGAAGATGTGGCAGTTTATTTCTGTCAGCAGCATTTACACATTCCTCC

IGKV8-23-1*01_DBA1	DBA/1J	IGKV	MUSMUS IGKV8-16*01 F, OR MUSMUS IGKV8-23-1*01 ORF	100	GAAATTGTGCTGACTGTGTCAG CAGGAGAGAGGGTCACTATCAG CTGCAAATCCAATCAGAATCTTT TATGGAGTGGAAACCAAAGGTA CTGTTTGGTCTGGCACCAGTGG AAACCAGGGCAAACTCCTACAC CGTTGATCACCTGGACATCTGAT AGGTACTCTGGAGTCCCTGATC GTTTCATAGGCAGTGGATCTGTG ACAGATTTCACTCTGACCATCAG CAGTGTGCAGGCTGAAGATGTG GCAGTTTATTTCTGTCAGCAGCA TTTACACATTCCTCC
IGKV8-23-1*01_DBA2	DBA/2J	IGKV	MUSMUS IGKV8-16*01 F, OR MUSMUS IGKV8-23-1*01 ORF	100	GAAATTGTGCTGACTGTGTCAG CAGGAGAGAGGGTCACTATCAG CTGCAAATCCAATCAGAATCTTT TATGGAGTGGAAACCAAAGGTA CTGTTTGGTCTGGCACCAGTGG AAACCAGGGCAAACTCCTACAC CGTTGATCACCTGGACATCTGAT AGGTACTCTGGAGTCCCTGATC GTTTCATAGGCAGTGGATCTGTG ACAGATTTCACTCTGACCATCAG CAGTGTGCAGGCTGAAGATGTG GCAGTTTATTTCTGTCAGCAGCA TTTACACATTCCTCC
IGKV8-23-1*01_NZB	NZB/BLNJ	IGKV	MUSMUS IGKV8-16*01 F, OR MUSMUS IGKV8-23-1*01 ORF	100	GAAATTGTGCTGACTGTGTCAG CAGGAGAGAGGGTCACTATCAG CTGCAAATCCAATCAGAATCTTT TATGGAGTGGAAACCAAAGGTA CTGTTTGGTCTGGCACCAGTGG AAACCAGGGCAAACTCCTACAC CGTTGATCACCTGGACATCTGAT AGGTACTCTGGAGTCCCTGATC GTTTCATAGGCAGTGGATCTGTG ACAGATTTCACTCTGACCATCAG CAGTGTGCAGGCTGAAGATGTG GCAGTTTATTTCTGTCAGCAGCA TTTACACATTCCTCC
IGKV8-23-1*01_SJL	SJL/J	IGKV	MUSMUS IGKV8-16*01 F, OR MUSMUS IGKV8-23-1*01 ORF	100	GAAATTGTGCTGACTGTGTCAG CAGGAGAGAGGGTCACTATCAG CTGCAAATCCAATCAGAATCTTT TATGGAGTGGAAACCAAAGGTA CTGTTTGGTCTGGCACCAGTGG AAACCAGGGCAAACTCCTACAC CGTTGATCACCTGGACATCTGAT AGGTACTCTGGAGTCCCTGATC GTTTCATAGGCAGTGGATCTGTG ACAGATTTCACTCTGACCATCAG CAGTGTGCAGGCTGAAGATGTG GCAGTTTATTTCTGTCAGCAGCA TTTACACATTCCTCC

IGKV8-24*01_129	129S1/SVIMJ	IGKV	MUSMUS IGKV8-24*01 F	100	GACATTGTGATGACACAGTCTCCATCCTCCCTGGCTATGTCAGTAGGACAGAAGGTCACTATGAGCTGCAAGTCCAGTCAGAGCCTTTTAAATAGTAGCAATCAAAAGAACTATTTGGCCTGGTACCAGCAGAAACCAGGACAGTCTCCTAAACTTCTGGTATACTTTGCATCCACTAGGGAATCTGGGGTCCCTGATCGCTTCATAGGCAGTGGATCTGGGACAGATTTCACTCTTACCATCAGCAGTGTGCAGGCTGAAGACCTGGCAGATTACTTCTGTCAGCAACATTATAGCACTCCTCC
IGKV8-24*01_AJ	A/J	IGKV	MUSMUS IGKV8-24*01 F	100	GACATTGTGATGACACAGTCTCCATCCTCCCTGGCTATGTCAGTAGGACAGAAGGTCACTATGAGCTGCAAGTCCAGTCAGAGCCTTTTAAATAGTAGCAATCAAAAGAACTATTTGGCCTGGTACCAGCAGAAACCAGGACAGTCTCCTAAACTTCTGGTATACTTTGCATCCACTAGGGAATCTGGGGTCCCTGATCGCTTCATAGGCAGTGGATCTGGGACAGATTTCACTCTTACCATCAGCAGTGTGCAGGCTGAAGACCTGGCAGATTACTTCTGTCAGCAACATTATAGCACTCCTCC
IGKV8-24*01_B6	C57BL/6J	IGKV	MUSMUS IGKV8-24*01 F	100	GACATTGTGATGACACAGTCTCCATCCTCCCTGGCTATGTCAGTAGGACAGAAGGTCACTATGAGCTGCAAGTCCAGTCAGAGCCTTTTAAATAGTAGCAATCAAAAGAACTATTTGGCCTGGTACCAGCAGAAACCAGGACAGTCTCCTAAACTTCTGGTATACTTTGCATCCACTAGGGAATCTGGGGTCCCTGATCGCTTCATAGGCAGTGGATCTGGGACAGATTTCACTCTTACCATCAGCAGTGTGCAGGCTGAAGACCTGGCAGATTACTTCTGTCAGCAACATTATAGCACTCCTCC
IGKV8-24*01_BALB	BALB/CBYJ	IGKV	MUSMUS IGKV8-24*01 F	100	GACATTGTGATGACACAGTCTCCATCCTCCCTGGCTATGTCAGTAGGACAGAAGGTCACTATGAGCTGCAAGTCCAGTCAGAGCCTTTTAAATAGTAGCAATCAAAAGAACTATTTGGCCTGGTACCAGCAGAAACCAGGACAGTCTCCTAAACTTCTGGTATACTTTGCATCCACTAGGGAATCTGGGGTCCCTGATCGCTTCATAGGCAGTGGATCTGGGACAGATTTCACTCTTACCATCAGCAGTGTGCAGGCTGAAGACCTGGCAGATTACTTCTGTCAGCAACATTATAGCACTCCTCC

IGKV8-24*01_C3H	C3H/HEJ	IGKV	MUSMUS IGKV8-24*01 F	100	GACATTGTGATGACACAGTCTCCATCCTCCCTGGCTATGTCAGTAGGACAGAAGGTCACTATGAGCTGCAAGTCCAGTCAGAGCCTTTTAAATAGTAGCAATCAAAAGAACTATTTGGCCTGGTACCAGCAGAAACCAGGACAGTCTCCTAAACTTCTGGTATACTTTGCATCCACTAGGGAATCTGGGGTCCCTGATCGCTTCATAGGCAGTGGATCTGGGACAGATTTCACTCTTACCATCAGCAGTGTGCAGGCTGAAGACCTGGCAGATTACTTCTGTCAGCAACATTATAGCACTCCTCC
IGKV8-24*01_CBA	CBA/J	IGKV	MUSMUS IGKV8-24*01 F	100	GACATTGTGATGACACAGTCTCCATCCTCCCTGGCTATGTCAGTAGGACAGAAGGTCACTATGAGCTGCAAGTCCAGTCAGAGCCTTTTAAATAGTAGCAATCAAAAGAACTATTTGGCCTGGTACCAGCAGAAACCAGGACAGTCTCCTAAACTTCTGGTATACTTTGCATCCACTAGGGAATCTGGGGTCCCTGATCGCTTCATAGGCAGTGGATCTGGGACAGATTTCACTCTTACCATCAGCAGTGTGCAGGCTGAAGACCTGGCAGATTACTTCTGTCAGCAACATTATAGCACTCCTCC
IGKV8-24*01_DBA1	DBA/1J	IGKV	MUSMUS IGKV8-24*01 F	100	GACATTGTGATGACACAGTCTCCATCCTCCCTGGCTATGTCAGTAGGACAGAAGGTCACTATGAGCTGCAAGTCCAGTCAGAGCCTTTTAAATAGTAGCAATCAAAAGAACTATTTGGCCTGGTACCAGCAGAAACCAGGACAGTCTCCTAAACTTCTGGTATACTTTGCATCCACTAGGGAATCTGGGGTCCCTGATCGCTTCATAGGCAGTGGATCTGGGACAGATTTCACTCTTACCATCAGCAGTGTGCAGGCTGAAGACCTGGCAGATTACTTCTGTCAGCAACATTATAGCACTCCTCC
IGKV8-24*01_DBA2	DBA/2J	IGKV	MUSMUS IGKV8-24*01 F	100	GACATTGTGATGACACAGTCTCCATCCTCCCTGGCTATGTCAGTAGGACAGAAGGTCACTATGAGCTGCAAGTCCAGTCAGAGCCTTTTAAATAGTAGCAATCAAAAGAACTATTTGGCCTGGTACCAGCAGAAACCAGGACAGTCTCCTAAACTTCTGGTATACTTTGCATCCACTAGGGAATCTGGGGTCCCTGATCGCTTCATAGGCAGTGGATCTGGGACAGATTTCACTCTTACCATCAGCAGTGTGCAGGCTGAAGACCTGGCAGATTACTTCTGTCAGCAACATTATAGCACTCCTCC

IGKV8-24*01_LEWES	LEWES/EIJ	IGKV	MUSMUS IGKV8-24*01 F	100	GACATTGTGATGACACAGTCTCCATCCTCCCTGGCTATGTCAGTAGGACAGAAGGTCACTATGAGCTGCAAGTCCAGTCAGAGCCTTTTAAATAGTAGCAATCAAAAGAACTATTTGGCCTGGTACCAGCAGAAACCAGGACAGTCTCCTAAACTTCTGGTATACTTTGCATCCACTAGGGAATCTGGGGTCCCTGATCGCTTCATAGGCAGTGGATCTGGGACAGATTTCACTCTTACCATCAGCAGTGTGCAGGCTGAAGACCTGGCAGATTACTTCTGTCAGCAACATTATAGCACTCCTCC
IGKV8-24*01_NZB	NZB/BLNJ	IGKV	MUSMUS IGKV8-24*01 F	100	GACATTGTGATGACACAGTCTCCATCCTCCCTGGCTATGTCAGTAGGACAGAAGGTCACTATGAGCTGCAAGTCCAGTCAGAGCCTTTTAAATAGTAGCAATCAAAAGAACTATTTGGCCTGGTACCAGCAGAAACCAGGACAGTCTCCTAAACTTCTGGTATACTTTGCATCCACTAGGGAATCTGGGGTCCCTGATCGCTTCATAGGCAGTGGATCTGGGACAGATTTCACTCTTACCATCAGCAGTGTGCAGGCTGAAGACCTGGCAGATTACTTCTGTCAGCAACATTATAGCACTCCTCC
IGKV8-24*01_S6188_PWD	PWD/PHJ	IGKV	MUSMUS IGKV8-24*01 F	98.32	GACATTGTGATGACACAGTCTCCATCCTCCCTGGCTATGTCAGTAGGACAGAAGGTCACTATGAGCTGCAAGTCCAGTCAGAGCCTTTTAAATAGTAGCAATCAAAAGAACTATTTGGCCTGGTACCAGCAGAAACCAGGACAGTCTCCTAAACTTCTGGTATACTTTGCATCCACTAGGGAATCTGGGGTCCCTGATCGCTTCACAGGCAGTGGATCTGGGACAGATTTCACTCTTGCCATCAGCAGTGTGCAGGCTGAAGACCTGGCAGTTTATTACTGTCAGCAACATTATAGCACTCCTCC
IGKV8-24*01_S8843_AKR	AKR/J	IGKV	MUSMUS IGKV8-24*01 F	98.32	GACATTGTGATGACACAGTCTCCATCCTCCCTGGCTATGTCAGTAGGACAGAAGGTCACTATGAGCTGCAAGTCCAGTCAGAGCCTTTTAAGTAGTAGCAATCAAAAGAACTATTTGGCCTGGTACCAGCAGAAACCAGGACAGTCTCCTAAACTTCTGGTATACTTTGCATCCACTAGGGAATCTGGGGTCCCTGATCGCTTCATAGGCAGTGGATCTGGGACAGATTTCACTCTTACCATCAGCAGTGTGCAGGCTGAAGACCTGGCACTTTATTACTGTCAGCAACATTATAGCACTCCTCC

IGKV8-24*01_S8843_CAST	CAST/EIJ	IGKV	MUSMUS IGKV8-24*01 F	98.32	GACATTGTGATGACACAGTCTCCATCCTCCCTGGCTATGTCAGTAGGACAGAAGGTCACTATGAGCTGCAAGTCCAGTCAGAGCCTTTTAAGTAGTAGCAATCAAAAGAACTATTTGGCCTGGTACCAGCAGAAACCAGGACAGTCTCCTAAACTTCTGGTATACTTTGCATCCACTAGGGAATCTGGGGTCCCTGATCGCTTCATAGGCAGTGGATCTGGGACAGATTTCACTCTTACCATCAGCAGTGTGCAGGCTGAAGACCTGGCACTTTATTACTGTCAGCAACATTATAGCACTCCTCC
IGKV8-24*01_S8843_MRL	MRL/MPJ	IGKV	MUSMUS IGKV8-24*01 F	98.32	GACATTGTGATGACACAGTCTCCATCCTCCCTGGCTATGTCAGTAGGACAGAAGGTCACTATGAGCTGCAAGTCCAGTCAGAGCCTTTTAAGTAGTAGCAATCAAAAGAACTATTTGGCCTGGTACCAGCAGAAACCAGGACAGTCTCCTAAACTTCTGGTATACTTTGCATCCACTAGGGAATCTGGGGTCCCTGATCGCTTCATAGGCAGTGGATCTGGGACAGATTTCACTCTTACCATCAGCAGTGTGCAGGCTGAAGACCTGGCACTTTATTACTGTCAGCAACATTATAGCACTCCTCC
IGKV8-24*01_S8843_NOD	NOD/SHILTJ	IGKV	MUSMUS IGKV8-24*01 F	98.32	GACATTGTGATGACACAGTCTCCATCCTCCCTGGCTATGTCAGTAGGACAGAAGGTCACTATGAGCTGCAAGTCCAGTCAGAGCCTTTTAAGTAGTAGCAATCAAAAGAACTATTTGGCCTGGTACCAGCAGAAACCAGGACAGTCTCCTAAACTTCTGGTATACTTTGCATCCACTAGGGAATCTGGGGTCCCTGATCGCTTCATAGGCAGTGGATCTGGGACAGATTTCACTCTTACCATCAGCAGTGTGCAGGCTGAAGACCTGGCACTTTATTACTGTCAGCAACATTATAGCACTCCTCC
IGKV8-24*01_S8843_NOR	NOR/LTJ	IGKV	MUSMUS IGKV8-24*01 F	98.32	GACATTGTGATGACACAGTCTCCATCCTCCCTGGCTATGTCAGTAGGACAGAAGGTCACTATGAGCTGCAAGTCCAGTCAGAGCCTTTTAAGTAGTAGCAATCAAAAGAACTATTTGGCCTGGTACCAGCAGAAACCAGGACAGTCTCCTAAACTTCTGGTATACTTTGCATCCACTAGGGAATCTGGGGTCCCTGATCGCTTCATAGGCAGTGGATCTGGGACAGATTTCACTCTTACCATCAGCAGTGTGCAGGCTGAAGACCTGGCACTTTATTACTGTCAGCAACATTATAGCACTCCTCC

IGKV8-24*01_SJL	SJL/J	IGKV	MUSMUS IGKV8-24*01 F	100	GACATTGTGATGACACAGTCTCCATCCTCCCTGGCTATGTCAGTAGGACAGAAGGTCACTATGAGCTGCAAGTCCAGTCAGAGCCTTTTAAATAGTAGCAATCAAAAGAACTATTTGGCCTGGTACCAGCAGAAACCAGGACAGTCTCCTAAACTTCTGGTATACTTTGCATCCACTAGGGAATCTGGGGTCCCTGATCGCTTCATAGGCAGTGGATCTGGGACAGATTTCACTCTTACCATCAGCAGTGTGCAGGCTGAAGACCTGGCAGATTACTTCTGTCAGCAACATTATAGCACTCCTCC
IGKV8-26*01_AJ	A/J	IGKV	MUSMUS IGKV8-26*01 ORF	100	GACATTGTGATGACCCAGTCTCCATCCTCCCTGGCTGTGACAGCAGGAGAGAAGGTCACTATGAGCTGCAAGTCCAGTCAGAGTCTTTTGTGGAGTGTAAACCAAAAGAACTACTTGTCCTGGTACCAGCAGAAACAAAGGCAGCCTCCTAAACTGCTTATCTATGGGGCATCCATTAGAGAATCTTGGGTCCCTGATCGGTTCACAGGAAGTGGATCTGGGACAGACTTCACTCTCACCATTAGCAATGTGCATGCTGAAGACCTAGCAGTTTATTACTGTCAACACAATCATGGCAGCTTTCTCCCCCC
IGKV8-26*01_BALB	BALB/CBYJ	IGKV	MUSMUS IGKV8-26*01 ORF	100	GACATTGTGATGACCCAGTCTCCATCCTCCCTGGCTGTGACAGCAGGAGAGAAGGTCACTATGAGCTGCAAGTCCAGTCAGAGTCTTTTGTGGAGTGTAAACCAAAAGAACTACTTGTCCTGGTACCAGCAGAAACAAAGGCAGCCTCCTAAACTGCTTATCTATGGGGCATCCATTAGAGAATCTTGGGTCCCTGATCGGTTCACAGGAAGTGGATCTGGGACAGACTTCACTCTCACCATTAGCAATGTGCATGCTGAAGACCTAGCAGTTTATTACTGTCAACACAATCATGGCAGCTTTCTCCCCCC
IGKV8-26*01_C3H	C3H/HEJ	IGKV	MUSMUS IGKV8-26*01 ORF	100	GACATTGTGATGACCCAGTCTCCATCCTCCCTGGCTGTGACAGCAGGAGAGAAGGTCACTATGAGCTGCAAGTCCAGTCAGAGTCTTTTGTGGAGTGTAAACCAAAAGAACTACTTGTCCTGGTACCAGCAGAAACAAAGGCAGCCTCCTAAACTGCTTATCTATGGGGCATCCATTAGAGAATCTTGGGTCCCTGATCGGTTCACAGGAAGTGGATCTGGGACAGACTTCACTCTCACCATTAGCAATGTGCATGCTGAAGACCTAGCAGTTTATTACTGTCAACACAATCATGGCAGCTTTCTCCCCCC

IGKV8-26*01_DBA2	DBA/2J	IGKV	MUSMUS IGKV8-26*01 ORF	100	GACATTGTGATGACCCAGTCTCCATCCTCCCTGGCTGTGACAGCAGGAGAGAAGGTCACTATGAGCTGCAAGTCCAGTCAGAGTCTTTTGTGGAGTGTAAACCAAAAGAACTACTTGTCCTGGTACCAGCAGAAACAAAGGCAGCCTCCTAAACTGCTTATCTATGGGGCATCCATTAGAGAATCTTGGGTCCCTGATCGGTTCACAGGAAGTGGATCTGGGACAGACTTCACTCTCACCATTAGCAATGTGCATGCTGAAGACCTAGCAGTTTATTACTGTCAACACAATCATGGCAGCTTTCTCCCCCC
IGKV8-26*01_NZB	NZB/BLNJ	IGKV	MUSMUS IGKV8-26*01 ORF	100	GACATTGTGATGACCCAGTCTCCATCCTCCCTGGCTGTGACAGCAGGAGAGAAGGTCACTATGAGCTGCAAGTCCAGTCAGAGTCTTTTGTGGAGTGTAAACCAAAAGAACTACTTGTCCTGGTACCAGCAGAAACAAAGGCAGCCTCCTAAACTGCTTATCTATGGGGCATCCATTAGAGAATCTTGGGTCCCTGATCGGTTCACAGGAAGTGGATCTGGGACAGACTTCACTCTCACCATTAGCAATGTGCATGCTGAAGACCTAGCAGTTTATTACTGTCAACACAATCATGGCAGCTTTCTCCCCCC
IGKV8-27*01_129	129S1/SVIMJ	IGKV	MUSMUS IGKV8-27*01 F	100	AACATTATGATGACACAGTCGCCATCATCTCTGGCTGTGTCTGCAGGAGAAAAGGTCACTATGAGCTGTAAGTCCAGTCAAAGTGTTTTATACAGTTCAAATCAGAAGAACTACTTGGCCTGGTACCAGCAGAAACCAGGGCAGTCTCCTAAACTGCTGATCTACTGGGCATCCACTAGGGAATCTGGTGTCCCTGATCGCTTCACAGGCAGTGGATCTGGGACAGATTTTACTCTTACCATCAGCAGTGTACAAGCTGAAGACCTGGCAGTTTATTACTGTCATCAATACCTCTCCTCG
IGKV8-27*01_AJ	A/J	IGKV	MUSMUS IGKV8-27*01 F	100	AACATTATGATGACACAGTCGCCATCATCTCTGGCTGTGTCTGCAGGAGAAAAGGTCACTATGAGCTGTAAGTCCAGTCAAAGTGTTTTATACAGTTCAAATCAGAAGAACTACTTGGCCTGGTACCAGCAGAAACCAGGGCAGTCTCCTAAACTGCTGATCTACTGGGCATCCACTAGGGAATCTGGTGTCCCTGATCGCTTCACAGGCAGTGGATCTGGGACAGATTTTACTCTTACCATCAGCAGTGTACAAGCTGAAGACCTGGCAGTTTATTACTGTCATCAATACCTCTCCTCG

IGKV8-27*01_AKR	AKR/J	IGKV	MUSMUS IGKV8-27*01 F	100	AACATTATGATGACACAGTCGCCATCATCTCTGGCTGTGTCTGCAGGAGAAAAGGTCACTATGAGCTGTAAGTCCAGTCAAAGTGTTTTATACAGTTCAAATCAGAAGAACTACTTGGCCTGGTACCAGCAGAAACCAGGGCAGTCTCCTAAACTGCTGATCTACTGGGCATCCACTAGGGAATCTGGTGTCCCTGATCGCTTCACAGGCAGTGGATCTGGGACAGATTTTACTCTTACCATCAGCAGTGTACAAGCTGAAGACCTGGCAGTTTATTACTGTCATCAATACCTCTCCTCG
IGKV8-27*01_B6	C57BL/6J	IGKV	MUSMUS IGKV8-27*01 F	100	AACATTATGATGACACAGTCGCCATCATCTCTGGCTGTGTCTGCAGGAGAAAAGGTCACTATGAGCTGTAAGTCCAGTCAAAGTGTTTTATACAGTTCAAATCAGAAGAACTACTTGGCCTGGTACCAGCAGAAACCAGGGCAGTCTCCTAAACTGCTGATCTACTGGGCATCCACTAGGGAATCTGGTGTCCCTGATCGCTTCACAGGCAGTGGATCTGGGACAGATTTTACTCTTACCATCAGCAGTGTACAAGCTGAAGACCTGGCAGTTTATTACTGTCATCAATACCTCTCCTCG
IGKV8-27*01_BALB	BALB/CBYJ	IGKV	MUSMUS IGKV8-27*01 F	100	AACATTATGATGACACAGTCGCCATCATCTCTGGCTGTGTCTGCAGGAGAAAAGGTCACTATGAGCTGTAAGTCCAGTCAAAGTGTTTTATACAGTTCAAATCAGAAGAACTACTTGGCCTGGTACCAGCAGAAACCAGGGCAGTCTCCTAAACTGCTGATCTACTGGGCATCCACTAGGGAATCTGGTGTCCCTGATCGCTTCACAGGCAGTGGATCTGGGACAGATTTTACTCTTACCATCAGCAGTGTACAAGCTGAAGACCTGGCAGTTTATTACTGTCATCAATACCTCTCCTCG
IGKV8-27*01_C3H	C3H/HEJ	IGKV	MUSMUS IGKV8-27*01 F	100	AACATTATGATGACACAGTCGCCATCATCTCTGGCTGTGTCTGCAGGAGAAAAGGTCACTATGAGCTGTAAGTCCAGTCAAAGTGTTTTATACAGTTCAAATCAGAAGAACTACTTGGCCTGGTACCAGCAGAAACCAGGGCAGTCTCCTAAACTGCTGATCTACTGGGCATCCACTAGGGAATCTGGTGTCCCTGATCGCTTCACAGGCAGTGGATCTGGGACAGATTTTACTCTTACCATCAGCAGTGTACAAGCTGAAGACCTGGCAGTTTATTACTGTCATCAATACCTCTCCTCG

IGKV8-27*01_CBA	CBA/J	IGKV	MUSMUS IGKV8-27*01 F	100	AACATTATGATGACACAGTCGCCATCATCTCTGGCTGTGTCTGCAGGAGAAAAGGTCACTATGAGCTGTAAGTCCAGTCAAAGTGTTTTATACAGTTCAAATCAGAAGAACTACTTGGCCTGGTACCAGCAGAAACCAGGGCAGTCTCCTAAACTGCTGATCTACTGGGCATCCACTAGGGAATCTGGTGTCCCTGATCGCTTCACAGGCAGTGGATCTGGGACAGATTTTACTCTTACCATCAGCAGTGTACAAGCTGAAGACCTGGCAGTTTATTACTGTCATCAATACCTCTCCTCG
IGKV8-27*01_DBA1	DBA/1J	IGKV	MUSMUS IGKV8-27*01 F	100	AACATTATGATGACACAGTCGCCATCATCTCTGGCTGTGTCTGCAGGAGAAAAGGTCACTATGAGCTGTAAGTCCAGTCAAAGTGTTTTATACAGTTCAAATCAGAAGAACTACTTGGCCTGGTACCAGCAGAAACCAGGGCAGTCTCCTAAACTGCTGATCTACTGGGCATCCACTAGGGAATCTGGTGTCCCTGATCGCTTCACAGGCAGTGGATCTGGGACAGATTTTACTCTTACCATCAGCAGTGTACAAGCTGAAGACCTGGCAGTTTATTACTGTCATCAATACCTCTCCTCG
IGKV8-27*01_DBA2	DBA/2J	IGKV	MUSMUS IGKV8-27*01 F	100	AACATTATGATGACACAGTCGCCATCATCTCTGGCTGTGTCTGCAGGAGAAAAGGTCACTATGAGCTGTAAGTCCAGTCAAAGTGTTTTATACAGTTCAAATCAGAAGAACTACTTGGCCTGGTACCAGCAGAAACCAGGGCAGTCTCCTAAACTGCTGATCTACTGGGCATCCACTAGGGAATCTGGTGTCCCTGATCGCTTCACAGGCAGTGGATCTGGGACAGATTTTACTCTTACCATCAGCAGTGTACAAGCTGAAGACCTGGCAGTTTATTACTGTCATCAATACCTCTCCTCG
IGKV8-27*01_LEWES	LEWES/EIJ	IGKV	MUSMUS IGKV8-27*01 F	100	AACATTATGATGACACAGTCGCCATCATCTCTGGCTGTGTCTGCAGGAGAAAAGGTCACTATGAGCTGTAAGTCCAGTCAAAGTGTTTTATACAGTTCAAATCAGAAGAACTACTTGGCCTGGTACCAGCAGAAACCAGGGCAGTCTCCTAAACTGCTGATCTACTGGGCATCCACTAGGGAATCTGGTGTCCCTGATCGCTTCACAGGCAGTGGATCTGGGACAGATTTTACTCTTACCATCAGCAGTGTACAAGCTGAAGACCTGGCAGTTTATTACTGTCATCAATACCTCTCCTCG

IGKV8-27*01_MRL	MRL/MPJ	IGKV	MUSMUS IGKV8-27*01 F	100	AACATTATGATGACACAGTCGCCATCATCTCTGGCTGTGTCTGCAGGAGAAAAGGTCACTATGAGCTGTAAGTCCAGTCAAAGTGTTTTATACAGTTCAAATCAGAAGAACTACTTGGCCTGGTACCAGCAGAAACCAGGGCAGTCTCCTAAACTGCTGATCTACTGGGCATCCACTAGGGAATCTGGTGTCCCTGATCGCTTCACAGGCAGTGGATCTGGGACAGATTTTACTCTTACCATCAGCAGTGTACAAGCTGAAGACCTGGCAGTTTATTACTGTCATCAATACCTCTCCTCG
IGKV8-27*01_NOD	NOD/SHILTJ	IGKV	MUSMUS IGKV8-27*01 F	100	AACATTATGATGACACAGTCGCCATCATCTCTGGCTGTGTCTGCAGGAGAAAAGGTCACTATGAGCTGTAAGTCCAGTCAAAGTGTTTTATACAGTTCAAATCAGAAGAACTACTTGGCCTGGTACCAGCAGAAACCAGGGCAGTCTCCTAAACTGCTGATCTACTGGGCATCCACTAGGGAATCTGGTGTCCCTGATCGCTTCACAGGCAGTGGATCTGGGACAGATTTTACTCTTACCATCAGCAGTGTACAAGCTGAAGACCTGGCAGTTTATTACTGTCATCAATACCTCTCCTCG
IGKV8-27*01_NOR	NOR/LTJ	IGKV	MUSMUS IGKV8-27*01 F	100	AACATTATGATGACACAGTCGCCATCATCTCTGGCTGTGTCTGCAGGAGAAAAGGTCACTATGAGCTGTAAGTCCAGTCAAAGTGTTTTATACAGTTCAAATCAGAAGAACTACTTGGCCTGGTACCAGCAGAAACCAGGGCAGTCTCCTAAACTGCTGATCTACTGGGCATCCACTAGGGAATCTGGTGTCCCTGATCGCTTCACAGGCAGTGGATCTGGGACAGATTTTACTCTTACCATCAGCAGTGTACAAGCTGAAGACCTGGCAGTTTATTACTGTCATCAATACCTCTCCTCG
IGKV8-27*01_NZB	NZB/BLNJ	IGKV	MUSMUS IGKV8-27*01 F	100	AACATTATGATGACACAGTCGCCATCATCTCTGGCTGTGTCTGCAGGAGAAAAGGTCACTATGAGCTGTAAGTCCAGTCAAAGTGTTTTATACAGTTCAAATCAGAAGAACTACTTGGCCTGGTACCAGCAGAAACCAGGGCAGTCTCCTAAACTGCTGATCTACTGGGCATCCACTAGGGAATCTGGTGTCCCTGATCGCTTCACAGGCAGTGGATCTGGGACAGATTTTACTCTTACCATCAGCAGTGTACAAGCTGAAGACCTGGCAGTTTATTACTGTCATCAATACCTCTCCTCG

IGKV8-27*01_PWD	PWD/PHJ	IGKV	MUSMUS IGKV8-27*01 F	100	AACATTATGATGACACAGTCGCCATCATCTCTGGCTGTGTCTGCAGGAGAAAAGGTCACTATGAGCTGTAAGTCCAGTCAAAGTGTTTTATACAGTTCAAATCAGAAGAACTACTTGGCCTGGTACCAGCAGAAACCAGGGCAGTCTCCTAAACTGCTGATCTACTGGGCATCCACTAGGGAATCTGGTGTCCCTGATCGCTTCACAGGCAGTGGATCTGGGACAGATTTTACTCTTACCATCAGCAGTGTACAAGCTGAAGACCTGGCAGTTTATTACTGTCATCAATACCTCTCCTCG
IGKV8-27*01_SJL	SJL/J	IGKV	MUSMUS IGKV8-27*01 F	100	AACATTATGATGACACAGTCGCCATCATCTCTGGCTGTGTCTGCAGGAGAAAAGGTCACTATGAGCTGTAAGTCCAGTCAAAGTGTTTTATACAGTTCAAATCAGAAGAACTACTTGGCCTGGTACCAGCAGAAACCAGGGCAGTCTCCTAAACTGCTGATCTACTGGGCATCCACTAGGGAATCTGGTGTCCCTGATCGCTTCACAGGCAGTGGATCTGGGACAGATTTTACTCTTACCATCAGCAGTGTACAAGCTGAAGACCTGGCAGTTTATTACTGTCATCAATACCTCTCCTCG
IGKV8-28*01_AJ	A/J	IGKV	MUSMUS IGKV8-28*01 F	100	GACATTGTGATGACACAGTCTCCATCCTCCCTGAGTGTGTCAGCAGGAGAGAAGGTCACTATGAGCTGCAAGTCCAGTCAGAGTCTGTTAAACAGTGGAAATCAAAAGAACTACTTGGCCTGGTACCAGCAGAAACCAGGGCAGCCTCCTAAACTGTTGATCTACGGGGCATCCACTAGGGAATCTGGGGTCCCTGATCGCTTCACAGGCAGTGGATCTGGAACCGATTTCACTCTTACCATCAGCAGTGTGCAGGCTGAAGACCTGGCAGTTTATTACTGTCAGAATGATCATAGTTATCCTCC
IGKV8-28*01_B6	C57BL/6J	IGKV	MUSMUS IGKV8-28*01 F	100	GACATTGTGATGACACAGTCTCCATCCTCCCTGAGTGTGTCAGCAGGAGAGAAGGTCACTATGAGCTGCAAGTCCAGTCAGAGTCTGTTAAACAGTGGAAATCAAAAGAACTACTTGGCCTGGTACCAGCAGAAACCAGGGCAGCCTCCTAAACTGTTGATCTACGGGGCATCCACTAGGGAATCTGGGGTCCCTGATCGCTTCACAGGCAGTGGATCTGGAACCGATTTCACTCTTACCATCAGCAGTGTGCAGGCTGAAGACCTGGCAGTTTATTACTGTCAGAATGATCATAGTTATCCTCC

IGKV8-28*01_BALB	BALB/CBYJ	IGKV	MUSMUS IGKV8-28*01 F	100	GACATTGTGATGACACAGTCTCCATCCTCCCTGAGTGTGTCAGCAGGAGAGAAGGTCACTATGAGCTGCAAGTCCAGTCAGAGTCTGTTAAACAGTGGAAATCAAAAGAACTACTTGGCCTGGTACCAGCAGAAACCAGGGCAGCCTCCTAAACTGTTGATCTACGGGGCATCCACTAGGGAATCTGGGGTCCCTGATCGCTTCACAGGCAGTGGATCTGGAACCGATTTCACTCTTACCATCAGCAGTGTGCAGGCTGAAGACCTGGCAGTTTATTACTGTCAGAATGATCATAGTTATCCTCC
IGKV8-28*01_C3H	C3H/HEJ	IGKV	MUSMUS IGKV8-28*01 F	100	GACATTGTGATGACACAGTCTCCATCCTCCCTGAGTGTGTCAGCAGGAGAGAAGGTCACTATGAGCTGCAAGTCCAGTCAGAGTCTGTTAAACAGTGGAAATCAAAAGAACTACTTGGCCTGGTACCAGCAGAAACCAGGGCAGCCTCCTAAACTGTTGATCTACGGGGCATCCACTAGGGAATCTGGGGTCCCTGATCGCTTCACAGGCAGTGGATCTGGAACCGATTTCACTCTTACCATCAGCAGTGTGCAGGCTGAAGACCTGGCAGTTTATTACTGTCAGAATGATCATAGTTATCCTCC
IGKV8-28*01_CBA	CBA/J	IGKV	MUSMUS IGKV8-28*01 F	100	GACATTGTGATGACACAGTCTCCATCCTCCCTGAGTGTGTCAGCAGGAGAGAAGGTCACTATGAGCTGCAAGTCCAGTCAGAGTCTGTTAAACAGTGGAAATCAAAAGAACTACTTGGCCTGGTACCAGCAGAAACCAGGGCAGCCTCCTAAACTGTTGATCTACGGGGCATCCACTAGGGAATCTGGGGTCCCTGATCGCTTCACAGGCAGTGGATCTGGAACCGATTTCACTCTTACCATCAGCAGTGTGCAGGCTGAAGACCTGGCAGTTTATTACTGTCAGAATGATCATAGTTATCCTCC
IGKV8-28*01_DBA1	DBA/1J	IGKV	MUSMUS IGKV8-28*01 F	100	GACATTGTGATGACACAGTCTCCATCCTCCCTGAGTGTGTCAGCAGGAGAGAAGGTCACTATGAGCTGCAAGTCCAGTCAGAGTCTGTTAAACAGTGGAAATCAAAAGAACTACTTGGCCTGGTACCAGCAGAAACCAGGGCAGCCTCCTAAACTGTTGATCTACGGGGCATCCACTAGGGAATCTGGGGTCCCTGATCGCTTCACAGGCAGTGGATCTGGAACCGATTTCACTCTTACCATCAGCAGTGTGCAGGCTGAAGACCTGGCAGTTTATTACTGTCAGAATGATCATAGTTATCCTCC

IGKV8-28*01_DBA2	DBA/2J	IGKV	MUSMUS IGKV8-28*01 F	100	GACATTGTGATGACACAGTCTCCATCCTCCCTGAGTGTGTCAGCAGGAGAGAAGGTCACTATGAGCTGCAAGTCCAGTCAGAGTCTGTTAAACAGTGGAAATCAAAAGAACTACTTGGCCTGGTACCAGCAGAAACCAGGGCAGCCTCCTAAACTGTTGATCTACGGGGCATCCACTAGGGAATCTGGGGTCCCTGATCGCTTCACAGGCAGTGGATCTGGAACCGATTTCACTCTTACCATCAGCAGTGTGCAGGCTGAAGACCTGGCAGTTTATTACTGTCAGAATGATCATAGTTATCCTCC
IGKV8-28*01_LEWES	LEWES/EIJ	IGKV	MUSMUS IGKV8-28*01 F	100	GACATTGTGATGACACAGTCTCCATCCTCCCTGAGTGTGTCAGCAGGAGAGAAGGTCACTATGAGCTGCAAGTCCAGTCAGAGTCTGTTAAACAGTGGAAATCAAAAGAACTACTTGGCCTGGTACCAGCAGAAACCAGGGCAGCCTCCTAAACTGTTGATCTACGGGGCATCCACTAGGGAATCTGGGGTCCCTGATCGCTTCACAGGCAGTGGATCTGGAACCGATTTCACTCTTACCATCAGCAGTGTGCAGGCTGAAGACCTGGCAGTTTATTACTGTCAGAATGATCATAGTTATCCTCC
IGKV8-28*01_NZB	NZB/BLNJ	IGKV	MUSMUS IGKV8-28*01 F	100	GACATTGTGATGACACAGTCTCCATCCTCCCTGAGTGTGTCAGCAGGAGAGAAGGTCACTATGAGCTGCAAGTCCAGTCAGAGTCTGTTAAACAGTGGAAATCAAAAGAACTACTTGGCCTGGTACCAGCAGAAACCAGGGCAGCCTCCTAAACTGTTGATCTACGGGGCATCCACTAGGGAATCTGGGGTCCCTGATCGCTTCACAGGCAGTGGATCTGGAACCGATTTCACTCTTACCATCAGCAGTGTGCAGGCTGAAGACCTGGCAGTTTATTACTGTCAGAATGATCATAGTTATCCTCC
IGKV8-28*01_S2077_PWD	PWD/PHJ	IGKV	MUSMUS IGKV8-28*01 F	99.33	GACATTGTGATGACACAGTCTCCATCCTCCCTGAGTGTGTCAGCAGGAGAGAAGGTCACTATGAGCTGCAAGTCCAGTCAGAGTCTGTTAAACAGTGGAAACCAAAAGAACTACTTGGCCTGGTACCAGCAGAAACCAGGGCAGCCTCCTAAACTGTTGATCTACGGGGCATCCACTAGGGAATCTGGGGTCCCTGATCGCTTCACAGGCAGTGGATCTGGAACCGATTTCACTCTTACCATCAGCAGTGTGCAGGCTGAAGACCTGGCAGTTTATTACTGTCAGAATGATTATAGTTATCCTCC

IGKV8-28*01_S7054_129	129S1/SVIMJ	IGKV	MUSMUS IGKV8-28*01 F	99.66	GACATTGTGATGACACAGTCTCCATCCTCCCTGAGTGTGTCAGCAGGAGAGAAGGTCACTATGAGCTGCAAGTCCAGTCAGAGTCTGTTAAACAGTGGAAATCAAAAGAACTACTTGGCCTGGTACCAGCAGAAACCAGGGCAGCCTCCTAAACTGTTGATCTACGGGGCATCCACTAGGGAATCTGGGGTCCCTGATCGCTTCACAGGCAGTGGATCTGGAACCGATTTCACTCTTACCATCAGCAGTGTGCAGGCTGAAGACCTGGCAGTTTATTACTGTCTGAATGATCATAGTTATCCTCC
IGKV8-28*01_SJL	SJL/J	IGKV	MUSMUS IGKV8-28*01 F	100	GACATTGTGATGACACAGTCTCCATCCTCCCTGAGTGTGTCAGCAGGAGAGAAGGTCACTATGAGCTGCAAGTCCAGTCAGAGTCTGTTAAACAGTGGAAATCAAAAGAACTACTTGGCCTGGTACCAGCAGAAACCAGGGCAGCCTCCTAAACTGTTGATCTACGGGGCATCCACTAGGGAATCTGGGGTCCCTGATCGCTTCACAGGCAGTGGATCTGGAACCGATTTCACTCTTACCATCAGCAGTGTGCAGGCTGAAGACCTGGCAGTTTATTACTGTCAGAATGATCATAGTTATCCTCC
IGKV8-28*02_AKR	AKR/J	IGKV	MUSMUS IGKV8-28*02 F	100	GACATTGTGATGACACAGTCTCCATCCTCCCTGAGTGTGTCAGCAGGAGATAAGGTCACTATGAGCTGCAAGTCCAGTCAGAGTCTGTTAAACAGTAGAAACCAAAAGAACTACTTGGCCTGGTACCAGCAGAAACCATGGCAGCCTCCTAAACTGCTGATCTACGGGGCATCCACTAGGGAATCTGGGGTCCCTGATCGCTTCACAGGCAGTGGATCTGGAACAGATTTCACTCTCACCATCAGCAGTGTGCAGGCTGAAGACCTGGCAGTTTATTACTGTCAGAATGATTATAGTTATCC
IGKV8-28*02_CAST	CAST/EIJ	IGKV	MUSMUS IGKV8-28*02 F	100	GACATTGTGATGACACAGTCTCCATCCTCCCTGAGTGTGTCAGCAGGAGATAAGGTCACTATGAGCTGCAAGTCCAGTCAGAGTCTGTTAAACAGTAGAAACCAAAAGAACTACTTGGCCTGGTACCAGCAGAAACCATGGCAGCCTCCTAAACTGCTGATCTACGGGGCATCCACTAGGGAATCTGGGGTCCCTGATCGCTTCACAGGCAGTGGATCTGGAACAGATTTCACTCTCACCATCAGCAGTGTGCAGGCTGAAGACCTGGCAGTTTATTACTGTCAGAATGATTATAGTTATCC

IGKV8-28*02_MRL	MRL/MPJ	IGKV	MUSMUS IGKV8-28*02 F	100	GACATTGTGATGACACAGTCTCCATCCTCCCTGAGTGTGTCAGCAGGAGATAAGGTCACTATGAGCTGCAAGTCCAGTCAGAGTCTGTTAAACAGTAGAAACCAAAAGAACTACTTGGCCTGGTACCAGCAGAAACCATGGCAGCCTCCTAAACTGCTGATCTACGGGGCATCCACTAGGGAATCTGGGGTCCCTGATCGCTTCACAGGCAGTGGATCTGGAACAGATTTCACTCTCACCATCAGCAGTGTGCAGGCTGAAGACCTGGCAGTTTATTACTGTCAGAATGATTATAGTTATCC
IGKV8-28*02_NOD	NOD/SHILTJ	IGKV	MUSMUS IGKV8-28*02 F	100	GACATTGTGATGACACAGTCTCCATCCTCCCTGAGTGTGTCAGCAGGAGATAAGGTCACTATGAGCTGCAAGTCCAGTCAGAGTCTGTTAAACAGTAGAAACCAAAAGAACTACTTGGCCTGGTACCAGCAGAAACCATGGCAGCCTCCTAAACTGCTGATCTACGGGGCATCCACTAGGGAATCTGGGGTCCCTGATCGCTTCACAGGCAGTGGATCTGGAACAGATTTCACTCTCACCATCAGCAGTGTGCAGGCTGAAGACCTGGCAGTTTATTACTGTCAGAATGATTATAGTTATCC
IGKV8-28*02_NOR	NOR/LTJ	IGKV	MUSMUS IGKV8-28*02 F	100	GACATTGTGATGACACAGTCTCCATCCTCCCTGAGTGTGTCAGCAGGAGATAAGGTCACTATGAGCTGCAAGTCCAGTCAGAGTCTGTTAAACAGTAGAAACCAAAAGAACTACTTGGCCTGGTACCAGCAGAAACCATGGCAGCCTCCTAAACTGCTGATCTACGGGGCATCCACTAGGGAATCTGGGGTCCCTGATCGCTTCACAGGCAGTGGATCTGGAACAGATTTCACTCTCACCATCAGCAGTGTGCAGGCTGAAGACCTGGCAGTTTATTACTGTCAGAATGATTATAGTTATCC
IGKV8-30*01_129	129S1/SVIMJ	IGKV	MUSMUS IGKV8-30*01 F	100	GACATTGTGATGTCACAGTCTCCATCCTCCCTAGCTGTGTCAGTTGGAGAGAAGGTTACTATGAGCTGCAAGTCCAGTCAGAGCCTTTTATATAGTAGCAATCAAAAGAACTACTTGGCCTGGTACCAGCAGAAACCAGGGCAGTCTCCTAAACTGCTGATTTACTGGGCATCCACTAGGGAATCTGGGGTCCCTGATCGCTTCACAGGCAGTGGATCTGGGACAGATTTCACTCTCACCATCAGCAGTGTGAAGGCTGAAGACCTGGCAGTTTATTACTGTCAGCAATATTATAGCTATCCTCC

IGKV8-30*01_AJ	A/J	IGKV	MUSMUS IGKV8-30*01 F	100	GACATTGTGATGTCACAGTCTCCATCCTCCCTAGCTGTGTCAGTTGGAGAGAAGGTTACTATGAGCTGCAAGTCCAGTCAGAGCCTTTTATATAGTAGCAATCAAAAGAACTACTTGGCCTGGTACCAGCAGAAACCAGGGCAGTCTCCTAAACTGCTGATTTACTGGGCATCCACTAGGGAATCTGGGGTCCCTGATCGCTTCACAGGCAGTGGATCTGGGACAGATTTCACTCTCACCATCAGCAGTGTGAAGGCTGAAGACCTGGCAGTTTATTACTGTCAGCAATATTATAGCTATCCTCC
IGKV8-30*01_B6	C57BL/6J	IGKV	MUSMUS IGKV8-30*01 F	100	GACATTGTGATGTCACAGTCTCCATCCTCCCTAGCTGTGTCAGTTGGAGAGAAGGTTACTATGAGCTGCAAGTCCAGTCAGAGCCTTTTATATAGTAGCAATCAAAAGAACTACTTGGCCTGGTACCAGCAGAAACCAGGGCAGTCTCCTAAACTGCTGATTTACTGGGCATCCACTAGGGAATCTGGGGTCCCTGATCGCTTCACAGGCAGTGGATCTGGGACAGATTTCACTCTCACCATCAGCAGTGTGAAGGCTGAAGACCTGGCAGTTTATTACTGTCAGCAATATTATAGCTATCCTCC
IGKV8-30*01_BALB	BALB/CBYJ	IGKV	MUSMUS IGKV8-30*01 F	100	GACATTGTGATGTCACAGTCTCCATCCTCCCTAGCTGTGTCAGTTGGAGAGAAGGTTACTATGAGCTGCAAGTCCAGTCAGAGCCTTTTATATAGTAGCAATCAAAAGAACTACTTGGCCTGGTACCAGCAGAAACCAGGGCAGTCTCCTAAACTGCTGATTTACTGGGCATCCACTAGGGAATCTGGGGTCCCTGATCGCTTCACAGGCAGTGGATCTGGGACAGATTTCACTCTCACCATCAGCAGTGTGAAGGCTGAAGACCTGGCAGTTTATTACTGTCAGCAATATTATAGCTATCCTCC
IGKV8-30*01_C3H	C3H/HEJ	IGKV	MUSMUS IGKV8-30*01 F	100	GACATTGTGATGTCACAGTCTCCATCCTCCCTAGCTGTGTCAGTTGGAGAGAAGGTTACTATGAGCTGCAAGTCCAGTCAGAGCCTTTTATATAGTAGCAATCAAAAGAACTACTTGGCCTGGTACCAGCAGAAACCAGGGCAGTCTCCTAAACTGCTGATTTACTGGGCATCCACTAGGGAATCTGGGGTCCCTGATCGCTTCACAGGCAGTGGATCTGGGACAGATTTCACTCTCACCATCAGCAGTGTGAAGGCTGAAGACCTGGCAGTTTATTACTGTCAGCAATATTATAGCTATCCTCC

IGKV8-30*01_CBA	CBA/J	IGKV	MUSMUS IGKV8-30*01 F	100	GACATTGTGATGTCACAGTCTCCATCCTCCCTAGCTGTGTCAGTTGGAGAGAAGGTTACTATGAGCTGCAAGTCCAGTCAGAGCCTTTTATATAGTAGCAATCAAAAGAACTACTTGGCCTGGTACCAGCAGAAACCAGGGCAGTCTCCTAAACTGCTGATTTACTGGGCATCCACTAGGGAATCTGGGGTCCCTGATCGCTTCACAGGCAGTGGATCTGGGACAGATTTCACTCTCACCATCAGCAGTGTGAAGGCTGAAGACCTGGCAGTTTATTACTGTCAGCAATATTATAGCTATCCTCC
IGKV8-30*01_DBA1	DBA/1J	IGKV	MUSMUS IGKV8-30*01 F	100	GACATTGTGATGTCACAGTCTCCATCCTCCCTAGCTGTGTCAGTTGGAGAGAAGGTTACTATGAGCTGCAAGTCCAGTCAGAGCCTTTTATATAGTAGCAATCAAAAGAACTACTTGGCCTGGTACCAGCAGAAACCAGGGCAGTCTCCTAAACTGCTGATTTACTGGGCATCCACTAGGGAATCTGGGGTCCCTGATCGCTTCACAGGCAGTGGATCTGGGACAGATTTCACTCTCACCATCAGCAGTGTGAAGGCTGAAGACCTGGCAGTTTATTACTGTCAGCAATATTATAGCTATCCTCC
IGKV8-30*01_DBA2	DBA/2J	IGKV	MUSMUS IGKV8-30*01 F	100	GACATTGTGATGTCACAGTCTCCATCCTCCCTAGCTGTGTCAGTTGGAGAGAAGGTTACTATGAGCTGCAAGTCCAGTCAGAGCCTTTTATATAGTAGCAATCAAAAGAACTACTTGGCCTGGTACCAGCAGAAACCAGGGCAGTCTCCTAAACTGCTGATTTACTGGGCATCCACTAGGGAATCTGGGGTCCCTGATCGCTTCACAGGCAGTGGATCTGGGACAGATTTCACTCTCACCATCAGCAGTGTGAAGGCTGAAGACCTGGCAGTTTATTACTGTCAGCAATATTATAGCTATCCTCC
IGKV8-30*01_LEWES	LEWES/EIJ	IGKV	MUSMUS IGKV8-30*01 F	100	GACATTGTGATGTCACAGTCTCCATCCTCCCTAGCTGTGTCAGTTGGAGAGAAGGTTACTATGAGCTGCAAGTCCAGTCAGAGCCTTTTATATAGTAGCAATCAAAAGAACTACTTGGCCTGGTACCAGCAGAAACCAGGGCAGTCTCCTAAACTGCTGATTTACTGGGCATCCACTAGGGAATCTGGGGTCCCTGATCGCTTCACAGGCAGTGGATCTGGGACAGATTTCACTCTCACCATCAGCAGTGTGAAGGCTGAAGACCTGGCAGTTTATTACTGTCAGCAATATTATAGCTATCCTCC

IGKV8-30*01_NOR	NOR/LTJ	IGKV	MUSMUS IGKV8-30*01 F	100	GACATTGTGATGTCACAGTCTCCATCCTCCCTAGCTGTGTCAGTTGGAGAGAAGGTTACTATGAGCTGCAAGTCCAGTCAGAGCCTTTTATATAGTAGCAATCAAAAGAACTACTTGGCCTGGTACCAGCAGAAACCAGGGCAGTCTCCTAAACTGCTGATTTACTGGGCATCCACTAGGGAATCTGGGGTCCCTGATCGCTTCACAGGCAGTGGATCTGGGACAGATTTCACTCTCACCATCAGCAGTGTGAAGGCTGAAGACCTGGCAGTTTATTACTGTCAGCAATATTATAGCTATCCTCC
IGKV8-30*01_NZB	NZB/BLNJ	IGKV	MUSMUS IGKV8-30*01 F	100	GACATTGTGATGTCACAGTCTCCATCCTCCCTAGCTGTGTCAGTTGGAGAGAAGGTTACTATGAGCTGCAAGTCCAGTCAGAGCCTTTTATATAGTAGCAATCAAAAGAACTACTTGGCCTGGTACCAGCAGAAACCAGGGCAGTCTCCTAAACTGCTGATTTACTGGGCATCCACTAGGGAATCTGGGGTCCCTGATCGCTTCACAGGCAGTGGATCTGGGACAGATTTCACTCTCACCATCAGCAGTGTGAAGGCTGAAGACCTGGCAGTTTATTACTGTCAGCAATATTATAGCTATCCTCC
IGKV8-30*01_S0907_AKR	AKR/J	IGKV	MUSMUS IGKV8-30*01 F	95.62	GACATTGTGATGTCACAGTCTCCATCCGCCCTAGCTGTGTCAGTTGGAGAGAAGGTCACTATGAGCTGCAAGTCCAGTCAGAGCCTTTTATATAGTAGCAATCAAAAGAACTACTTGGCCTGGTACCAGCAGAAACCAGGGCAGTCTCCTAAACTGTTAATCTACTGGGCATCCACTAGGGAATCTGGGGTCCCTGACCGCTTCACAGGCAGTAGATCAGGGACAGATTTCACTCTCACCATCAGCAGTGTGCAGGCTGAAGACCTGGCCGTTTATTACTGCAAGCAATCTTATAGCTATCCTC
IGKV8-30*01_S1609_CAST	CAST/EIJ	IGKV	MUSMUS IGKV8-30*01 F	95.61	GACATTGTGATGTCACAGTCTCCATCCGCCCTAGCTGTGTCAGTTGGAGAGAAGGTCACTATGAGCTGCAAGTCCAGTCAGAGCCTTTTATATAGTAGCAATCAAAAGAACTACTTGGCCTGGTACCAGCAGAAACCAGGGCAGTCTCCTAAACTGTTAATCTACTGGGCATCCACTAGGGAATCTGGGGTCCCTGACCGCTTCACAGGCAGTAGATCAGGGACAGATTTCACTCTCACCATCAGCAGTGTGCAGGCTGAAGACCTGGCCGTTTATTACTGCAAGCAATCTTATAG

IGKV8-30*01_S3861_MRL	MRL/MPJ	IGKV	MUSMUS IGKV8-30*01 F	95.62	GACATTGTGATGTCACAGTCTCCATCCGCCCTAGCTGTGTCAGTTGGAGAGAAGGTCACTATGAGCTGCAAGTCCAGTCAGAGCCTTTTATATAGTAGCAATCAAAAGAACTACTTGGCCTGGTACCAGCAGAAACCAGGGCAGTCTCCTAAACTGTTAATCTACTGGGCATCCACTAGGGAATCTGGGGTCCCTGACCGCTTCACAGGCAGTAGATCAGGGACAGATTTCACTCTCACCATCAGCAGTGTGCAGGCTGAAGACCTGGCCGTTTATTACTGCAAGCAATCTTATAGCTATCC
IGKV8-30*01_S3861_NOR	NOR/LTJ	IGKV	MUSMUS IGKV8-30*01 F	95.62	GACATTGTGATGTCACAGTCTCCATCCGCCCTAGCTGTGTCAGTTGGAGAGAAGGTCACTATGAGCTGCAAGTCCAGTCAGAGCCTTTTATATAGTAGCAATCAAAAGAACTACTTGGCCTGGTACCAGCAGAAACCAGGGCAGTCTCCTAAACTGTTAATCTACTGGGCATCCACTAGGGAATCTGGGGTCCCTGACCGCTTCACAGGCAGTAGATCAGGGACAGATTTCACTCTCACCATCAGCAGTGTGCAGGCTGAAGACCTGGCCGTTTATTACTGCAAGCAATCTTATAGCTATCC
IGKV8-30*01_S4505_MSM	MSM/MSJ	IGKV	MUSMUS IGKV8-30*01 F	97.31	GACATTGTTATGTCACAGTCTCCATCCTCCCTGGCTGTGTCAGTTGGAGAGAAGGTTACTATGAGCTGCAAGTCCAGTCAGAGCCTTTTATATAGTAGCAATCAAAAGAACTACTTGGCTTGGTACCAGCAGAAACCTGGGCAGTCTCCTAAACTGTTGATCTACTGGGCATCCACTAGGGAATCTTGGGTCCCTGATCGCTTCACAGGCAGTGGATCTGGGACAGATTTCACTCTCACCATCAGCAGTGTGAAGGCTGAAGACCTGGCCGTTTATTACTGTCAGCAATATTATAGCGATCCTCC
IGKV8-30*01_S6795A_NOD	NOD/SHILTJ	IGKV	MUSMUS IGKV8-30*01 F	95.59	GACATTGTGATGTCACAGTCTCCATCCGCCCTAGCTGTGTCAGTTGGAGAGAAGGTCACTATGAGCTGCAAGTCCAGTCAGAGCCTTTTATATAGTAGCAATCAAAAGAACTACTTGGCCTGGTACCAGCAGAAACCAGGGCAGTCTCCTAAACTGTTAATCTACTGGGCATCCACTAGGGAATCTGGGGTCCCTGACCGCTTCACAGGCAGTAGATCAGGGACAGATTTCACTCTCACCATCAGCAGTGTGCAGGCTGAAGACCTGGCCGTTTATTACTGCAAGCAATCTTATA

IGKV8-30*01_S8873_PWD	PWD/PHJ	IGKV	MUSMUS IGKV8-30*01 F	96.61	GACATTGTGATGTCACAGTCTCCATCCTCCCTGGCTGTGTCAGTTGGAGAGAAGGTTACTATGAGCTGCAAATCCAGTCAGAGCCTTTTATATAGTAGCAATCAAAAGAACTACTTGGCCTGGTACCAGCAGAAACCTGGGCAGTCTCCTAAACTGTTGATCTACTGGGCATCCACTAGGGAATCTGGGGTCCCTGATCGCTTCACAGGCAGTGGATCTGGGACAGATTTCACTCTCACCATCAGCAGTGTGAAGGCTGAAGACCTGGCCGTTTATTACTGCAAGCAATCTTATG
IGKV8-30*01_SJL	SJL/J	IGKV	MUSMUS IGKV8-30*01 F	100	GACATTGTGATGTCACAGTCTCCATCCTCCCTAGCTGTGTCAGTTGGAGAGAAGGTTACTATGAGCTGCAAGTCCAGTCAGAGCCTTTTATATAGTAGCAATCAAAAGAACTACTTGGCCTGGTACCAGCAGAAACCAGGGCAGTCTCCTAAACTGCTGATTTACTGGGCATCCACTAGGGAATCTGGGGTCCCTGATCGCTTCACAGGCAGTGGATCTGGGACAGATTTCACTCTCACCATCAGCAGTGTGAAGGCTGAAGACCTGGCAGTTTATTACTGTCAGCAATATTATAGCTATCCTCC
IGKV8-34*01_129	129S1/SVIMJ	IGKV	MUSMUS IGKV8-34*01 F	100	GACATTTTGATGACCCAGTCTCCATCCTCCCTGACTGTGTCAGCAGGAGAGAAGGTCACTATGAGCTGCAAGTCCAGTCAGAGTCTTTTAGCTAGTGGCAACCAAAATAACTACTTGGCCTGGCACCAGCAGAAACCAGGACGATCTCCTAAAATGCTGATAATTTGGGCATCCACTAGGGTATCTGGAGTCCCTGATCGCTTCATAGGCAGTGGATCTGGGACGGATTTCACTCTGACCATCAACAGTGTGCAGGCTGAAGATCTGGCTGTTTATTACTGTCAGCAGTCCTACAGCGCTCCTAC
IGKV8-34*01_AJ	A/J	IGKV	MUSMUS IGKV8-34*01 F	100	GACATTTTGATGACCCAGTCTCCATCCTCCCTGACTGTGTCAGCAGGAGAGAAGGTCACTATGAGCTGCAAGTCCAGTCAGAGTCTTTTAGCTAGTGGCAACCAAAATAACTACTTGGCCTGGCACCAGCAGAAACCAGGACGATCTCCTAAAATGCTGATAATTTGGGCATCCACTAGGGTATCTGGAGTCCCTGATCGCTTCATAGGCAGTGGATCTGGGACGGATTTCACTCTGACCATCAACAGTGTGCAGGCTGAAGATCTGGCTGTTTATTACTGTCAGCAGTCCTACAGCGCTCCTAC

IGKV8-34*01_B6	C57BL/6J	IGKV	MUSMUS IGKV8-34*01 F	100	GACATTTTGATGACCCAGTCTCCATCCTCCCTGACTGTGTCAGCAGGAGAGAAGGTCACTATGAGCTGCAAGTCCAGTCAGAGTCTTTTAGCTAGTGGCAACCAAAATAACTACTTGGCCTGGCACCAGCAGAAACCAGGACGATCTCCTAAAATGCTGATAATTTGGGCATCCACTAGGGTATCTGGAGTCCCTGATCGCTTCATAGGCAGTGGATCTGGGACGGATTTCACTCTGACCATCAACAGTGTGCAGGCTGAAGATCTGGCTGTTTATTACTGTCAGCAGTCCTACAGCGCTCCTAC
IGKV8-34*01_BALB	BALB/CBYJ	IGKV	MUSMUS IGKV8-34*01 F	100	GACATTTTGATGACCCAGTCTCCATCCTCCCTGACTGTGTCAGCAGGAGAGAAGGTCACTATGAGCTGCAAGTCCAGTCAGAGTCTTTTAGCTAGTGGCAACCAAAATAACTACTTGGCCTGGCACCAGCAGAAACCAGGACGATCTCCTAAAATGCTGATAATTTGGGCATCCACTAGGGTATCTGGAGTCCCTGATCGCTTCATAGGCAGTGGATCTGGGACGGATTTCACTCTGACCATCAACAGTGTGCAGGCTGAAGATCTGGCTGTTTATTACTGTCAGCAGTCCTACAGCGCTCCTAC
IGKV8-34*01_C3H	C3H/HEJ	IGKV	MUSMUS IGKV8-34*01 F	100	GACATTTTGATGACCCAGTCTCCATCCTCCCTGACTGTGTCAGCAGGAGAGAAGGTCACTATGAGCTGCAAGTCCAGTCAGAGTCTTTTAGCTAGTGGCAACCAAAATAACTACTTGGCCTGGCACCAGCAGAAACCAGGACGATCTCCTAAAATGCTGATAATTTGGGCATCCACTAGGGTATCTGGAGTCCCTGATCGCTTCATAGGCAGTGGATCTGGGACGGATTTCACTCTGACCATCAACAGTGTGCAGGCTGAAGATCTGGCTGTTTATTACTGTCAGCAGTCCTACAGCGCTCCTAC
IGKV8-34*01_CBA	CBA/J	IGKV	MUSMUS IGKV8-34*01 F	100	GACATTTTGATGACCCAGTCTCCATCCTCCCTGACTGTGTCAGCAGGAGAGAAGGTCACTATGAGCTGCAAGTCCAGTCAGAGTCTTTTAGCTAGTGGCAACCAAAATAACTACTTGGCCTGGCACCAGCAGAAACCAGGACGATCTCCTAAAATGCTGATAATTTGGGCATCCACTAGGGTATCTGGAGTCCCTGATCGCTTCATAGGCAGTGGATCTGGGACGGATTTCACTCTGACCATCAACAGTGTGCAGGCTGAAGATCTGGCTGTTTATTACTGTCAGCAGTCCTACAGCGCTCCTAC

IGKV8-34*01_DBA1	DBA/1J	IGKV	MUSMUS IGKV8-34*01 F	100	GACATTTTGATGACCCAGTCTCCATCCTCCCTGACTGTGTCAGCAGGAGAGAAGGTCACTATGAGCTGCAAGTCCAGTCAGAGTCTTTTAGCTAGTGGCAACCAAAATAACTACTTGGCCTGGCACCAGCAGAAACCAGGACGATCTCCTAAAATGCTGATAATTTGGGCATCCACTAGGGTATCTGGAGTCCCTGATCGCTTCATAGGCAGTGGATCTGGGACGGATTTCACTCTGACCATCAACAGTGTGCAGGCTGAAGATCTGGCTGTTTATTACTGTCAGCAGTCCTACAGCGCTCCTAC
IGKV8-34*01_DBA2	DBA/2J	IGKV	MUSMUS IGKV8-34*01 F	100	GACATTTTGATGACCCAGTCTCCATCCTCCCTGACTGTGTCAGCAGGAGAGAAGGTCACTATGAGCTGCAAGTCCAGTCAGAGTCTTTTAGCTAGTGGCAACCAAAATAACTACTTGGCCTGGCACCAGCAGAAACCAGGACGATCTCCTAAAATGCTGATAATTTGGGCATCCACTAGGGTATCTGGAGTCCCTGATCGCTTCATAGGCAGTGGATCTGGGACGGATTTCACTCTGACCATCAACAGTGTGCAGGCTGAAGATCTGGCTGTTTATTACTGTCAGCAGTCCTACAGCGCTCCTAC
IGKV8-34*01_LEWES	LEWES/EIJ	IGKV	MUSMUS IGKV8-34*01 F	100	GACATTTTGATGACCCAGTCTCCATCCTCCCTGACTGTGTCAGCAGGAGAGAAGGTCACTATGAGCTGCAAGTCCAGTCAGAGTCTTTTAGCTAGTGGCAACCAAAATAACTACTTGGCCTGGCACCAGCAGAAACCAGGACGATCTCCTAAAATGCTGATAATTTGGGCATCCACTAGGGTATCTGGAGTCCCTGATCGCTTCATAGGCAGTGGATCTGGGACGGATTTCACTCTGACCATCAACAGTGTGCAGGCTGAAGATCTGGCTGTTTATTACTGTCAGCAGTCCTACAGCGCTCCTAC
IGKV8-34*01_NZB	NZB/BLNJ	IGKV	MUSMUS IGKV8-34*01 F	100	GACATTTTGATGACCCAGTCTCCATCCTCCCTGACTGTGTCAGCAGGAGAGAAGGTCACTATGAGCTGCAAGTCCAGTCAGAGTCTTTTAGCTAGTGGCAACCAAAATAACTACTTGGCCTGGCACCAGCAGAAACCAGGACGATCTCCTAAAATGCTGATAATTTGGGCATCCACTAGGGTATCTGGAGTCCCTGATCGCTTCATAGGCAGTGGATCTGGGACGGATTTCACTCTGACCATCAACAGTGTGCAGGCTGAAGATCTGGCTGTTTATTACTGTCAGCAGTCCTACAGCGCTCCTAC

IGKV8-34*01_S0627_MSM	MSM/MSJ	IGKV	MUSMUS IGKV8-34*01 F	98.32	GACATTTTGATGACTCAGTCTCC<br>ATCCTCCCTGGCTGTGTCAGCA<br>GGAGAGAAGGTCACTATGAGCT<br>GCAAGTCCAGTCAGAGTCTTTTA<br>GCTAGTGCCAACCAAAATAACT<br>ACTTGGCCTGGCACCAGCAGA<br>AACCAGGACGATCTCCTAAAAT<br>GCTGATAATTTGGGCATCCACTA<br>GGGTATCTGGAGTCCCTGATCG<br>CTTCATAGGCAGTGGATCTGGA<br>ACGGATTTCACTCTGACCATCAA<br>CAGTGTGCAGGCTGAAGATCTG<br>GCTGTTTATTACTGTCAGCAGTC<br>CTATAGCGCTCCTAC
IGKV8-34*01_S3615_AKR	AKR/J	IGKV	MUSMUS IGKV8-34*01 F	99.33	GACATTTTGATGACTCAGTCTCC<br>ATCCTCCCTGACTGTGTCAGCA<br>GGAGAGAAGGTCACTATGAGCT<br>GCAAGTCCAGTCAGAGTCTTTTA<br>GCTAGTGCCAACCAAAATAACT<br>ACTTGGCCTGGCACCAGCAGA<br>AACCAGGACGATCTCCTAAAAT<br>GCTGATAATTTGGGCATCCACTA<br>GGGTATCTGGAGTCCCTGATCG<br>CTTCATAGGCAGTGGATCTGGG<br>ACGGATTTCACTCTGACCATCAA<br>CAGTGTGCAGGCTGAAGATCTG<br>GCTGTTTATTACTGTCAGCAGTC<br>CTACAGCGCTCCTAC
IGKV8-34*01_S3615_CAST	CAST/EIJ	IGKV	MUSMUS IGKV8-34*01 F	99.33	GACATTTTGATGACTCAGTCTCC<br>ATCCTCCCTGACTGTGTCAGCA<br>GGAGAGAAGGTCACTATGAGCT<br>GCAAGTCCAGTCAGAGTCTTTTA<br>GCTAGTGCCAACCAAAATAACT<br>ACTTGGCCTGGCACCAGCAGA<br>AACCAGGACGATCTCCTAAAAT<br>GCTGATAATTTGGGCATCCACTA<br>GGGTATCTGGAGTCCCTGATCG<br>CTTCATAGGCAGTGGATCTGGG<br>ACGGATTTCACTCTGACCATCAA<br>CAGTGTGCAGGCTGAAGATCTG<br>GCTGTTTATTACTGTCAGCAGTC<br>CTACAGCGCTCCTAC
IGKV8-34*01_S3615_MRL	MRL/MPJ	IGKV	MUSMUS IGKV8-34*01 F	99.33	GACATTTTGATGACTCAGTCTCC<br>ATCCTCCCTGACTGTGTCAGCA<br>GGAGAGAAGGTCACTATGAGCT<br>GCAAGTCCAGTCAGAGTCTTTTA<br>GCTAGTGCCAACCAAAATAACT<br>ACTTGGCCTGGCACCAGCAGA<br>AACCAGGACGATCTCCTAAAAT<br>GCTGATAATTTGGGCATCCACTA<br>GGGTATCTGGAGTCCCTGATCG<br>CTTCATAGGCAGTGGATCTGGG<br>ACGGATTTCACTCTGACCATCAA<br>CAGTGTGCAGGCTGAAGATCTG<br>GCTGTTTATTACTGTCAGCAGTC<br>CTACAGCGCTCCTAC

IGKV8-34*01_S3615_NOD	NOD/SHILTJ	IGKV	MUSMUS IGKV8-34*01 F	99.33	GACATTTTGATGACTCAGTCTCC<br>ATCCTCCCTGACTGTGTCAGCA<br>GGAGAGAAGGTCACTATGAGCT<br>GCAAGTCCAGTCAGAGTCTTTTA<br>GCTAGTGCCAACCAAAATAACT<br>ACTTGGCCTGGCACCAGCAGA<br>AACCAGGACGATCTCCTAAAAT<br>GCTGATAATTTGGGCATCCACTA<br>GGGTATCTGGAGTCCCTGATCG<br>CTTCATAGGCAGTGGATCTGGG<br>ACGGATTTCACTCTGACCATCAA<br>CAGTGTGCAGGCTGAAGATCTG<br>GCTGTTTATTACTGTCAGCAGTC<br>CTACAGCGCTCCTAC
IGKV8-34*01_S3615_NOR	NOR/LTJ	IGKV	MUSMUS IGKV8-34*01 F	99.33	GACATTTTGATGACTCAGTCTCC<br>ATCCTCCCTGACTGTGTCAGCA<br>GGAGAGAAGGTCACTATGAGCT<br>GCAAGTCCAGTCAGAGTCTTTTA<br>GCTAGTGCCAACCAAAATAACT<br>ACTTGGCCTGGCACCAGCAGA<br>AACCAGGACGATCTCCTAAAAT<br>GCTGATAATTTGGGCATCCACTA<br>GGGTATCTGGAGTCCCTGATCG<br>CTTCATAGGCAGTGGATCTGGG<br>ACGGATTTCACTCTGACCATCAA<br>CAGTGTGCAGGCTGAAGATCTG<br>GCTGTTTATTACTGTCAGCAGTC<br>CTACAGCGCTCCTAC
IGKV8-34*01_SJL	SJL/J	IGKV	MUSMUS IGKV8-34*01 F	100	GACATTTTGATGACCCAGTCTCC<br>ATCCTCCCTGACTGTGTCAGCA<br>GGAGAGAAGGTCACTATGAGCT<br>GCAAGTCCAGTCAGAGTCTTTTA<br>GCTAGTGGCAACCAAAATAACT<br>ACTTGGCCTGGCACCAGCAGA<br>AACCAGGACGATCTCCTAAAAT<br>GCTGATAATTTGGGCATCCACTA<br>GGGTATCTGGAGTCCCTGATCG<br>CTTCATAGGCAGTGGATCTGGG<br>ACGGATTTCACTCTGACCATCAA<br>CAGTGTGCAGGCTGAAGATCTG<br>GCTGTTTATTACTGTCAGCAGTC<br>CTACAGCGCTCCTAC
IGKV9-120*01_S0946_PWD	PWD/PHJ	IGKV	MUSMUS IGKV9-120*02 F	97.13	GACATCCAGATGACCCAGTCTC<br>CATCCTCCTTATCTGCCTCTCTG<br>GGAGAAAGAATCAGTCTCACTT<br>GCCGGGCAAGTCAGGACATTG<br>GTGGTAGCTTAAACTGGTTTCAG<br>CAGAAACCAGATGGAACTTTTAA<br>ACGCCTGATCTACGCCACATCC<br>AGTTTAGATTCTGGTGTCCCCAA<br>AAGGTTCAGTGGCAGTAGGTCT<br>GGGTCAGATTATTCTCTCACCAT<br>CAGCAGCCTTGAGTCTGAAGAT<br>TTTGCAGACTATTACTGTTTACAA<br>TATGCTAGTTATCCT

IGKV9-120*01_S2575_LEWES	LEWES/EIJ	IGKV	MUSMUS IGKV9-120*02 F	94.98	GACATCCAGATGACACAGTCTCCATCCTCCTTATCTGCCTCTCTGGGAGACAGAATCAGTCTCACTTGCCGGGCAAGTCAGGACATTGGTGGGAGCTTAAACTGGTTTCAGCAGAAACCAGATGAAACTTTTAAACGCCTGATATACGCCACATCCAATTTAGATTCTGGTGTCCCAAAAAGGTTCAGTGGCAGTAGGTCTGGGTCAGATTATTCTCTCACCATCAGCAGCCTTGAGTCTGAAGATTTTGCAGACTATTACTGTCTACAATATGCTAGTTATCCT
IGKV9-120*01_S2628_129	129S1/SVIMJ	IGKV	MUSMUS IGKV9-120*02 F	100	GACATCCAGATGACCCAGTCTCCATCCTCCTTATCTGCCTCTCTGGGAGAAAGAGTCAGTCTCACTTGTCGGGCAAGTCAGGACATTGGTAGTAGCTTAAACTGGCTTCAGCAGGAACCAGATGGAACTATTAAACGCCTGATCTACGCCACATCCAGTTTAGATTCTGGTGTCCCCAAAAGGTTCAGTGGCAGTAGGTCTGGGTCAGATTATTCTCTCACCATCAGCAGCCTTGAGTCTGAAGATTTTGTAGACTATTACTGTCTACAATATGCTAGTTCTCCT
IGKV9-120*01_S2628_AJ	A/J	IGKV	MUSMUS IGKV9-120*02 F	100	GACATCCAGATGACCCAGTCTCCATCCTCCTTATCTGCCTCTCTGGGAGAAAGAGTCAGTCTCACTTGTCGGGCAAGTCAGGACATTGGTAGTAGCTTAAACTGGCTTCAGCAGGAACCAGATGGAACTATTAAACGCCTGATCTACGCCACATCCAGTTTAGATTCTGGTGTCCCCAAAAGGTTCAGTGGCAGTAGGTCTGGGTCAGATTATTCTCTCACCATCAGCAGCCTTGAGTCTGAAGATTTTGTAGACTATTACTGTCTACAATATGCTAGTTCTCCT
IGKV9-120*01_S2628_AKR	AKR/J	IGKV	MUSMUS IGKV9-120*02 F	100	GACATCCAGATGACCCAGTCTCCATCCTCCTTATCTGCCTCTCTGGGAGAAAGAGTCAGTCTCACTTGTCGGGCAAGTCAGGACATTGGTAGTAGCTTAAACTGGCTTCAGCAGGAACCAGATGGAACTATTAAACGCCTGATCTACGCCACATCCAGTTTAGATTCTGGTGTCCCCAAAAGGTTCAGTGGCAGTAGGTCTGGGTCAGATTATTCTCTCACCATCAGCAGCCTTGAGTCTGAAGATTTTGTAGACTATTACTGTCTACAATATGCTAGTTCTCCT

IGKV9-120*01_S2628_B6	C57BL/6J	IGKV	MUSMUS IGKV9-120*02 F	100	GACATCCAGATGACCCAGTCTCCATCCTCCTTATCTGCCTCTCTGGGAGAAAGAGTCAGTCTCACTTGTCGGGCAAGTCAGGACATTGGTAGTAGCTTAAACTGGCTTCAGCAGGAACCAGATGGAACTATTAAACGCCTGATCTACGCCACATCCAGTTTAGATTCTGGTGTCCCCAAAAGGTTCAGTGGCAGTAGGTCTGGGTCAGATTATTCTCTCACCATCAGCAGCCTTGAGTCTGAAGATTTTGTAGACTATTACTGTCTACAATATGCTAGTTCTCCT
IGKV9-120*01_S2628_BALB	BALB/CBYJ	IGKV	MUSMUS IGKV9-120*02 F	100	GACATCCAGATGACCCAGTCTCCATCCTCCTTATCTGCCTCTCTGGGAGAAAGAGTCAGTCTCACTTGTCGGGCAAGTCAGGACATTGGTAGTAGCTTAAACTGGCTTCAGCAGGAACCAGATGGAACTATTAAACGCCTGATCTACGCCACATCCAGTTTAGATTCTGGTGTCCCCAAAAGGTTCAGTGGCAGTAGGTCTGGGTCAGATTATTCTCTCACCATCAGCAGCCTTGAGTCTGAAGATTTTGTAGACTATTACTGTCTACAATATGCTAGTTCTCCT
IGKV9-120*01_S2628_C3H	C3H/HEJ	IGKV	MUSMUS IGKV9-120*02 F	100	GACATCCAGATGACCCAGTCTCCATCCTCCTTATCTGCCTCTCTGGGAGAAAGAGTCAGTCTCACTTGTCGGGCAAGTCAGGACATTGGTAGTAGCTTAAACTGGCTTCAGCAGGAACCAGATGGAACTATTAAACGCCTGATCTACGCCACATCCAGTTTAGATTCTGGTGTCCCCAAAAGGTTCAGTGGCAGTAGGTCTGGGTCAGATTATTCTCTCACCATCAGCAGCCTTGAGTCTGAAGATTTTGTAGACTATTACTGTCTACAATATGCTAGTTCTCCT
IGKV9-120*01_S2628_CBA	CBA/J	IGKV	MUSMUS IGKV9-120*02 F	100	GACATCCAGATGACCCAGTCTCCATCCTCCTTATCTGCCTCTCTGGGAGAAAGAGTCAGTCTCACTTGTCGGGCAAGTCAGGACATTGGTAGTAGCTTAAACTGGCTTCAGCAGGAACCAGATGGAACTATTAAACGCCTGATCTACGCCACATCCAGTTTAGATTCTGGTGTCCCCAAAAGGTTCAGTGGCAGTAGGTCTGGGTCAGATTATTCTCTCACCATCAGCAGCCTTGAGTCTGAAGATTTTGTAGACTATTACTGTCTACAATATGCTAGTTCTCCT

IGKV9-120*01_S2628_DBA1	DBA/1J	IGKV	MUSMUS IGKV9-120*02 F	100	GACATCCAGATGACCCAGTCTCCATCCTCCTTATCTGCCTCTCTGGGAGAAAGAGTCAGTCTCACTTGTCGGGCAAGTCAGGACATTGGTAGTAGCTTAAACTGGCTTCAGCAGGAACCAGATGGAACTATTAAACGCCTGATCTACGCCACATCCAGTTTAGATTCTGGTGTCCCCAAAAGGTTCAGTGGCAGTAGGTCTGGGTCAGATTATTCTCTCACCATCAGCAGCCTTGAGTCTGAAGATTTTGTAGACTATTACTGTCTACAATATGCTAGTTCTCCT
IGKV9-120*01_S2628_DBA2	DBA/2J	IGKV	MUSMUS IGKV9-120*02 F	100	GACATCCAGATGACCCAGTCTCCATCCTCCTTATCTGCCTCTCTGGGAGAAAGAGTCAGTCTCACTTGTCGGGCAAGTCAGGACATTGGTAGTAGCTTAAACTGGCTTCAGCAGGAACCAGATGGAACTATTAAACGCCTGATCTACGCCACATCCAGTTTAGATTCTGGTGTCCCCAAAAGGTTCAGTGGCAGTAGGTCTGGGTCAGATTATTCTCTCACCATCAGCAGCCTTGAGTCTGAAGATTTTGTAGACTATTACTGTCTACAATATGCTAGTTCTCCT
IGKV9-120*01_S2628_MRL	MRL/MPJ	IGKV	MUSMUS IGKV9-120*02 F	100	GACATCCAGATGACCCAGTCTCCATCCTCCTTATCTGCCTCTCTGGGAGAAAGAGTCAGTCTCACTTGTCGGGCAAGTCAGGACATTGGTAGTAGCTTAAACTGGCTTCAGCAGGAACCAGATGGAACTATTAAACGCCTGATCTACGCCACATCCAGTTTAGATTCTGGTGTCCCCAAAAGGTTCAGTGGCAGTAGGTCTGGGTCAGATTATTCTCTCACCATCAGCAGCCTTGAGTCTGAAGATTTTGTAGACTATTACTGTCTACAATATGCTAGTTCTCCT
IGKV9-120*01_S2628_SJL	SJL/J	IGKV	MUSMUS IGKV9-120*02 F	100	GACATCCAGATGACCCAGTCTCCATCCTCCTTATCTGCCTCTCTGGGAGAAAGAGTCAGTCTCACTTGTCGGGCAAGTCAGGACATTGGTAGTAGCTTAAACTGGCTTCAGCAGGAACCAGATGGAACTATTAAACGCCTGATCTACGCCACATCCAGTTTAGATTCTGGTGTCCCCAAAAGGTTCAGTGGCAGTAGGTCTGGGTCAGATTATTCTCTCACCATCAGCAGCCTTGAGTCTGAAGATTTTGTAGACTATTACTGTCTACAATATGCTAGTTCTCCT

IGKV9-120*01_S5626_LEWES	LEWES/EIJ	IGKV	MUSMUS IGKV9-120*02 F	97.49	GACATCCAGATGACCCAGTCTCCATCCTCCTTATCTGCCTCTCTGGGAGAAAGAGTCAGTCTCACTTGCCGGGCAAGTCAGGACATTGGTGGTAGCTTAAACTGGTTTCAGCAGAAACCAGATGGAACTATTAAACGCCTGATCTACGGCACATCCAGTTTAGATTCTGGTGTCCCCAAAAGGTTCAGTGGCAGTAGGTCTGGGTCAGATTATTCTCTCACCATCAGCAGCCTAGAGTCTGAAGATTTTGCAGACTATTACTGTCTACAATATGCTAGTTCTCCT
IGKV9-120*01_S5725_MSM	MSM/MSJ	IGKV	MUSMUS IGKV9-120*02 F	96.42	GACATCCAGATGACCCAGTCTCCATCCTCCTTATCTGCCTCTCTGGGAGAAAGAATCAGTCTCACTTGCCGGGCAAGTCAGGACATTGGTGGTAGCTTAAACTGGTTCCAGCAGAAACCAGATGGAACTATTAAACGCCTGATCTACGGCACATCCAGTTTAGATTCTGGTGTCCCCAAAAGGTTCAGTGGCAGTAGGTCTGGGTCAGATTATTCTCTCACCATCAGCAGCCTTGAGTCTGAAGACTTTGCAGACTATTACTGTTTACAATATGCTAGTTATCCT
IGKV9-120*01_S7607_CAST	CAST/EIJ	IGKV	MUSMUS IGKV9-120*02 F	96.42	GACATCCAGATGACCCAGTCTCCATCCTCCTTATCTGCCTCTCTGGGAGAAAGAATCAGTCTCACTTGCCGGGCAAGTCAGGACATTTATGGTAGCTTAAACTGGTTTCAGCAGAAACCAGATGGAACTATTAAACTCCTGATCTACGGCACATCCAGTTTAGATTCTGGTGTCCCCAAAAGGTTCAGTGGCAGTAGGTCTGGGTCAGATTATTCTCTCACCATCAGCAGCCTTGAGTCTGAAGATTTTGCAGACTATTACTGTCTACAATATGCTAGTTCTCCT
IGKV9-120*01_S7607_NOR	NOR/LTJ	IGKV	MUSMUS IGKV9-120*02 F	96.42	GACATCCAGATGACCCAGTCTCCATCCTCCTTATCTGCCTCTCTGGGAGAAAGAATCAGTCTCACTTGCCGGGCAAGTCAGGACATTTATGGTAGCTTAAACTGGTTTCAGCAGAAACCAGATGGAACTATTAAACTCCTGATCTACGGCACATCCAGTTTAGATTCTGGTGTCCCCAAAAGGTTCAGTGGCAGTAGGTCTGGGTCAGATTATTCTCTCACCATCAGCAGCCTTGAGTCTGAAGATTTTGCAGACTATTACTGTCTACAATATGCTAGTTCTCCT

IGKV9-120*01_S7607_NZB	NZB/BLNJ	IGKV	MUSMUS IGKV9-120*02 F	96.42	GACATCCAGATGACCCAGTCTCCATCCTCCTTATCTGCCTCTCTGGGAGAAAGAATCAGTCTCACTTGCCGGGCAAGTCAGGACATTTATGGTAGCTTAAACTGGTTTCAGCAGAAACCAGATGGAACTATTAAACTCCTGATCTACGGCACATCCAGTTTAGATTCTGGTGTCCCCAAAAGGTTCAGTGGCAGTAGGTCTGGGTCAGATTATTCTCTCACCATCAGCAGCCTTGAGTCTGAAGATTTTGCAGACTATTACTGTCTACAATATGCTAGTTCTCCT
IGKV9-120*01_S9262_PWD	PWD/PHJ	IGKV	MUSMUS IGKV9-120*02 F	96.42	GACATCCAGATGACCCAGTCTCCATCCTCCTTATCTGCCTCTCTGGGAGAAAGAATCAGTCTCACTTGCCGGGCAAGTCAGGACATTGGTGGTAGCTTAAACTGGTTTCAGCAGAAACCAGATGGAACTTTTAAACGCCTGATCTACGCCATATCCAGTTTAGATTCTGGTGTCCCCAAAAGGTTCAGTGGCAGTAGGTCTGGGTCAGATTATTCTCTCACCATCAGCAGCCTTGAGTCTGAAGATTTTGCAGACTATTACTGTTTACAATATGCAAGTTATCC
IGKV9-123*01_129	129S1/SVIMJ	IGKV	MUSMUS IGKV9-123*01 F	100	GACATCCAGATGATTCAGTCTCCATCGTCCATGTTTGCCTCTCTGGGAGACAGAGTCAGTCTCTCTTGTCGGGCTAGTCAGGGCATTAGAGGTAATTTAGACTGGTATCAGCAGAAACCAGGTGGAACTATTAAACTCCTGATCTACTCCACATCCAATTTAAATTCTGGTGTCCCATCAAGGTTCAGTGGCAGTGGGTCTGGGTCAGATTATTCTCTCACCATCAGCAGCCTAGAGTCTGAAGATTTTGCAGACTATTACTGTCTACAGCGTAATGCGTATCCT
IGKV9-123*01_AJ	A/J	IGKV	MUSMUS IGKV9-123*01 F	100	GACATCCAGATGATTCAGTCTCCATCGTCCATGTTTGCCTCTCTGGGAGACAGAGTCAGTCTCTCTTGTCGGGCTAGTCAGGGCATTAGAGGTAATTTAGACTGGTATCAGCAGAAACCAGGTGGAACTATTAAACTCCTGATCTACTCCACATCCAATTTAAATTCTGGTGTCCCATCAAGGTTCAGTGGCAGTGGGTCTGGGTCAGATTATTCTCTCACCATCAGCAGCCTAGAGTCTGAAGATTTTGCAGACTATTACTGTCTACAGCGTAATGCGTATCCT

IGKV9-123*01_AKR	AKR/J	IGKV	MUSMUS IGKV9-123*01 F	100	GACATCCAGATGATTCAGTCTCCATCGTCCATGTTTGCCTCTCTGGGAGACAGAGTCAGTCTCTCTTGTCGGGCTAGTCAGGGCATTAGAGGTAATTTAGACTGGTATCAGCAGAAACCAGGTGGAACTATTAAACTCCTGATCTACTCCACATCCAATTTAAATTCTGGTGTCCCATCAAGGTTCAGTGGCAGTGGGTCTGGGTCAGATTATTCTCTCACCATCAGCAGCCTAGAGTCTGAAGATTTTGCAGACTATTACTGTCTACAGCGTAATGCGTATCCT
IGKV9-123*01_B6	C57BL/6J	IGKV	MUSMUS IGKV9-123*01 F	100	GACATCCAGATGATTCAGTCTCCATCGTCCATGTTTGCCTCTCTGGGAGACAGAGTCAGTCTCTCTTGTCGGGCTAGTCAGGGCATTAGAGGTAATTTAGACTGGTATCAGCAGAAACCAGGTGGAACTATTAAACTCCTGATCTACTCCACATCCAATTTAAATTCTGGTGTCCCATCAAGGTTCAGTGGCAGTGGGTCTGGGTCAGATTATTCTCTCACCATCAGCAGCCTAGAGTCTGAAGATTTTGCAGACTATTACTGTCTACAGCGTAATGCGTATCCT
IGKV9-123*01_BALB	BALB/CBYJ	IGKV	MUSMUS IGKV9-123*01 F	100	GACATCCAGATGATTCAGTCTCCATCGTCCATGTTTGCCTCTCTGGGAGACAGAGTCAGTCTCTCTTGTCGGGCTAGTCAGGGCATTAGAGGTAATTTAGACTGGTATCAGCAGAAACCAGGTGGAACTATTAAACTCCTGATCTACTCCACATCCAATTTAAATTCTGGTGTCCCATCAAGGTTCAGTGGCAGTGGGTCTGGGTCAGATTATTCTCTCACCATCAGCAGCCTAGAGTCTGAAGATTTTGCAGACTATTACTGTCTACAGCGTAATGCGTATCCT
IGKV9-123*01_C3H	C3H/HEJ	IGKV	MUSMUS IGKV9-123*01 F	100	GACATCCAGATGATTCAGTCTCCATCGTCCATGTTTGCCTCTCTGGGAGACAGAGTCAGTCTCTCTTGTCGGGCTAGTCAGGGCATTAGAGGTAATTTAGACTGGTATCAGCAGAAACCAGGTGGAACTATTAAACTCCTGATCTACTCCACATCCAATTTAAATTCTGGTGTCCCATCAAGGTTCAGTGGCAGTGGGTCTGGGTCAGATTATTCTCTCACCATCAGCAGCCTAGAGTCTGAAGATTTTGCAGACTATTACTGTCTACAGCGTAATGCGTATCCT

IGKV9-123*01_CBA	CBA/J	IGKV	MUSMUS IGKV9-123*01 F	100	GACATCCAGATGATTCAGTCTCCATCGTCCATGTTTGCCTCTCTGGGAGACAGAGTCAGTCTCTCTTGTCGGGCTAGTCAGGGCATTAGAGGTAATTTAGACTGGTATCAGCAGAAACCAGGTGGAACTATTAAACTCCTGATCTACTCCACATCCAATTTAAATTCTGGTGTCCCATCAAGGTTCAGTGGCAGTGGGTCTGGGTCAGATTATTCTCTCACCATCAGCAGCCTAGAGTCTGAAGATTTTGCAGACTATTACTGTCTACAGCGTAATGCGTATCCT
IGKV9-123*01_DBA1	DBA/1J	IGKV	MUSMUS IGKV9-123*01 F	100	GACATCCAGATGATTCAGTCTCCATCGTCCATGTTTGCCTCTCTGGGAGACAGAGTCAGTCTCTCTTGTCGGGCTAGTCAGGGCATTAGAGGTAATTTAGACTGGTATCAGCAGAAACCAGGTGGAACTATTAAACTCCTGATCTACTCCACATCCAATTTAAATTCTGGTGTCCCATCAAGGTTCAGTGGCAGTGGGTCTGGGTCAGATTATTCTCTCACCATCAGCAGCCTAGAGTCTGAAGATTTTGCAGACTATTACTGTCTACAGCGTAATGCGTATCCT
IGKV9-123*01_DBA2	DBA/2J	IGKV	MUSMUS IGKV9-123*01 F	100	GACATCCAGATGATTCAGTCTCCATCGTCCATGTTTGCCTCTCTGGGAGACAGAGTCAGTCTCTCTTGTCGGGCTAGTCAGGGCATTAGAGGTAATTTAGACTGGTATCAGCAGAAACCAGGTGGAACTATTAAACTCCTGATCTACTCCACATCCAATTTAAATTCTGGTGTCCCATCAAGGTTCAGTGGCAGTGGGTCTGGGTCAGATTATTCTCTCACCATCAGCAGCCTAGAGTCTGAAGATTTTGCAGACTATTACTGTCTACAGCGTAATGCGTATCCT
IGKV9-123*01_S4103_DBA1	DBA/1J	IGKV	MUSMUS IGKV9-123*01 F	100	GACATCCAGATGATTCAGTCTCCATCGTCCATGTTTGCCTCTCTGGGAGACAGAGTCAGTCTCTCTTGTCGGGCTAGTCAGGGCATTAGAGGTAATTTAGACTGGTATCAGCAGAAACCAGGTGGAACTATTAAACTCCTGATCTACTCCACATCCAATTTAAATTCTGGTGTCCCATCAAGGTTCAGTGGCAGTGGGTCTGGGTCAGATTATTCTCTCACCATCAGCAGCCTAGAGTCTGAAGATTTTGCAGACTATTACTGTCTACAGCGTAATGCGTTTCCT

IGKV9-123*01_S4220_CAST	CAST/EIJ	IGKV	MUSMUS IGKV9-123*01 F	99.28	GACATCCAGATGATTCAGTCTCCATCGTCCATGTTTGGCTCTCTGGGAGACAGAGTCAGTCTCTCTTGCCGGGCTAGTCAGGGCATTAGAGGTAATTTAGACTGGTATCAGCAGAAACCAGGTGGAACTATTAAACTCCTGATCTACTCCACATCCAATTTAAATTCTGGTGTCCCATCAAGGTTCAGTGGCAGTGGGTCTGGGTCAGATTATTCTCTCACCATCAGCAGCCTAGAGTCTGAAGATTTTGCAGACTATTACTGTCTACAGCGTAATGCGTATCCT
IGKV9-123*01_S4220_NOR	NOR/LTJ	IGKV	MUSMUS IGKV9-123*01 F	99.28	GACATCCAGATGATTCAGTCTCCATCGTCCATGTTTGGCTCTCTGGGAGACAGAGTCAGTCTCTCTTGCCGGGCTAGTCAGGGCATTAGAGGTAATTTAGACTGGTATCAGCAGAAACCAGGTGGAACTATTAAACTCCTGATCTACTCCACATCCAATTTAAATTCTGGTGTCCCATCAAGGTTCAGTGGCAGTGGGTCTGGGTCAGATTATTCTCTCACCATCAGCAGCCTAGAGTCTGAAGATTTTGCAGACTATTACTGTCTACAGCGTAATGCGTATCCT
IGKV9-123*01_S4220_NZB	NZB/BLNJ	IGKV	MUSMUS IGKV9-123*01 F	99.28	GACATCCAGATGATTCAGTCTCCATCGTCCATGTTTGGCTCTCTGGGAGACAGAGTCAGTCTCTCTTGCCGGGCTAGTCAGGGCATTAGAGGTAATTTAGACTGGTATCAGCAGAAACCAGGTGGAACTATTAAACTCCTGATCTACTCCACATCCAATTTAAATTCTGGTGTCCCATCAAGGTTCAGTGGCAGTGGGTCTGGGTCAGATTATTCTCTCACCATCAGCAGCCTAGAGTCTGAAGATTTTGCAGACTATTACTGTCTACAGCGTAATGCGTATCCT
IGKV9-123*01_S4220_PWD	PWD/PHJ	IGKV	MUSMUS IGKV9-123*01 F	99.28	GACATCCAGATGATTCAGTCTCCATCGTCCATGTTTGGCTCTCTGGGAGACAGAGTCAGTCTCTCTTGCCGGGCTAGTCAGGGCATTAGAGGTAATTTAGACTGGTATCAGCAGAAACCAGGTGGAACTATTAAACTCCTGATCTACTCCACATCCAATTTAAATTCTGGTGTCCCATCAAGGTTCAGTGGCAGTGGGTCTGGGTCAGATTATTCTCTCACCATCAGCAGCCTAGAGTCTGAAGATTTTGCAGACTATTACTGTCTACAGCGTAATGCGTATCCT

IGKV9-123*01_S4862_NZB	NZB/BLNJ	IGKV	MUSMUS IGKV9-123*01 F	89.61	GACATCCAGATGACCCAGTCTCCATCCTCCATGTCTGCCTCTCTGGGAGAAAGAGTCAGTCTCACTTGCCGGGCCAGTCAGGGCATTAACGGTAATTTACACTGGTTTCAGCAGAAGTCAGGTGGAACTCTTAAACGCCTGATCTACTCCACGTCCAATTTAGATTCTGGTGTTCCATCAAGGTTCAGTGGCAGCGGGTCTGGGTCAGATTATTCTCTCACCATCAGCAGCCTGGAGTCTGAAGATTTTGCAATCTATTACTATCTACAGTATGATGAACATCCT
IGKV9-123*01_S5635_LEWES	LEWES/EIJ	IGKV	MUSMUS IGKV9-123*01 F	98.57	GACATCCAGATGATTCAGTCTCCATCGTCCATGTTTGTCTCTCTGGGAGACAGAGTCAGTCTCTCTTGCCGGGCTAGTCAGGGCATTAGAGGTAATTTAGACTGGTATCAGCAGAAACCAGGTGGAACTATTAAACTCCTGATCTACTCCACATCCAATTTAAATTCTGGTGTCCCATCAAGGTTCAGTGGCAGTGGGTCTGGGTCAGATTATTCTCTCACCATCAGCAGCCTAGAGTCTGAAGATTTTGCAGACTATTACTGCCTACAGCGTAATGCATATCCT
IGKV9-123*01_S7058_MSM	MSM/MSJ	IGKV	MUSMUS IGKV9-123*01 F	99.28	GACATCCAGATGATTCAGTCTCCATCGTCCATGTTTGCCTCTCTGGGAGACAGAGTCAGTCTCTCTTGCCGGGCTAGTCAGGGCATTAGAGGTAATTTAGACTGGTATCAGCAGAAACCAGGTGGAACTATTAAACTCCTGATCTACTCCACATCTAATTTAAATTCTGGTGTCCCATCAAGGTTCAGTGGCAGTGGGTCTGGGTCAGATTATTCTCTCACCATCAGCAGCCTAGAGTCTGAAGATTTTGCAGACTATTACTGTCTACAGCGTAATGCGTATCCT
IGKV9-124*01_129	129S1/SVIMJ	IGKV	MUSMUS IGKV9-124*01 F	100	GACATCCAGATGACCCAGTCTCCATCCTCCTTATCTGCCTCTCTGGGAGAAAGAGTCAGTCTCACTTGTCGGGCAAGTCAGGAAATTAGTGGTTACTTAAGCTGGCTTCAGCAGAAACCAGATGGAACTATTAAACGCCTGATCTACGCCGCATCCACTTTAGATTCTGGTGTCCCAAAAAGGTTCAGTGGCAGTAGGTCTGGGTCAGATTATTCTCTCACCATCAGCAGCCTTGAGTCTGAAGATTTTGCAGACTATTACTGTCTACAATATGCTAGTTATCCT

IGKV9-124*01_AJ	A/J	IGKV	MUSMUS IGKV9-124*01 F	100	GACATCCAGATGACCCAGTCTCCATCCTCCTTATCTGCCTCTCTGGGAGAAAGAGTCAGTCTCACTTGTCGGGCAAGTCAGGAAATTAGTGGTTACTTAAGCTGGCTTCAGCAGAAACCAGATGGAACTATTAAACGCCTGATCTACGCCGCATCCACTTTAGATTCTGGTGTCCCAAAAAGGTTCAGTGGCAGTAGGTCTGGGTCAGATTATTCTCTCACCATCAGCAGCCTTGAGTCTGAAGATTTTGCAGACTATTACTGTCTACAATATGCTAGTTATCCT
IGKV9-124*01_AKR	AKR/J	IGKV	MUSMUS IGKV9-124*01 F	100	GACATCCAGATGACCCAGTCTCCATCCTCCTTATCTGCCTCTCTGGGAGAAAGAGTCAGTCTCACTTGTCGGGCAAGTCAGGAAATTAGTGGTTACTTAAGCTGGCTTCAGCAGAAACCAGATGGAACTATTAAACGCCTGATCTACGCCGCATCCACTTTAGATTCTGGTGTCCCAAAAAGGTTCAGTGGCAGTAGGTCTGGGTCAGATTATTCTCTCACCATCAGCAGCCTTGAGTCTGAAGATTTTGCAGACTATTACTGTCTACAATATGCTAGTTATCCT
IGKV9-124*01_B6	C57BL/6J	IGKV	MUSMUS IGKV9-124*01 F	100	GACATCCAGATGACCCAGTCTCCATCCTCCTTATCTGCCTCTCTGGGAGAAAGAGTCAGTCTCACTTGTCGGGCAAGTCAGGAAATTAGTGGTTACTTAAGCTGGCTTCAGCAGAAACCAGATGGAACTATTAAACGCCTGATCTACGCCGCATCCACTTTAGATTCTGGTGTCCCAAAAAGGTTCAGTGGCAGTAGGTCTGGGTCAGATTATTCTCTCACCATCAGCAGCCTTGAGTCTGAAGATTTTGCAGACTATTACTGTCTACAATATGCTAGTTATCCT
IGKV9-124*01_BALB	BALB/CBYJ	IGKV	MUSMUS IGKV9-124*01 F	100	GACATCCAGATGACCCAGTCTCCATCCTCCTTATCTGCCTCTCTGGGAGAAAGAGTCAGTCTCACTTGTCGGGCAAGTCAGGAAATTAGTGGTTACTTAAGCTGGCTTCAGCAGAAACCAGATGGAACTATTAAACGCCTGATCTACGCCGCATCCACTTTAGATTCTGGTGTCCCAAAAAGGTTCAGTGGCAGTAGGTCTGGGTCAGATTATTCTCTCACCATCAGCAGCCTTGAGTCTGAAGATTTTGCAGACTATTACTGTCTACAATATGCTAGTTATCCT

IGKV9-124*01_C3H	C3H/HEJ	IGKV	MUSMUS IGKV9-124*01 F	100	GACATCCAGATGACCCAGTCTCCATCCTCCTTATCTGCCTCTCTGGGAGAAAGAGTCAGTCTCACTTGTCGGGCAAGTCAGGAAATTAGTGGTTACTTAAGCTGGCTTCAGCAGAAACCAGATGGAACTATTAAACGCCTGATCTACGCCGCATCCACTTTAGATTCTGGTGTCCCAAAAAGGTTCAGTGGCAGTAGGTCTGGGTCAGATTATTCTCTCACCATCAGCAGCCTTGAGTCTGAAGATTTTGCAGACTATTACTGTCTACAATATGCTAGTTATCCT
IGKV9-124*01_CBA	CBA/J	IGKV	MUSMUS IGKV9-124*01 F	100	GACATCCAGATGACCCAGTCTCCATCCTCCTTATCTGCCTCTCTGGGAGAAAGAGTCAGTCTCACTTGTCGGGCAAGTCAGGAAATTAGTGGTTACTTAAGCTGGCTTCAGCAGAAACCAGATGGAACTATTAAACGCCTGATCTACGCCGCATCCACTTTAGATTCTGGTGTCCCAAAAAGGTTCAGTGGCAGTAGGTCTGGGTCAGATTATTCTCTCACCATCAGCAGCCTTGAGTCTGAAGATTTTGCAGACTATTACTGTCTACAATATGCTAGTTATCCT
IGKV9-124*01_DBA1	DBA/1J	IGKV	MUSMUS IGKV9-124*01 F	100	GACATCCAGATGACCCAGTCTCCATCCTCCTTATCTGCCTCTCTGGGAGAAAGAGTCAGTCTCACTTGTCGGGCAAGTCAGGAAATTAGTGGTTACTTAAGCTGGCTTCAGCAGAAACCAGATGGAACTATTAAACGCCTGATCTACGCCGCATCCACTTTAGATTCTGGTGTCCCAAAAAGGTTCAGTGGCAGTAGGTCTGGGTCAGATTATTCTCTCACCATCAGCAGCCTTGAGTCTGAAGATTTTGCAGACTATTACTGTCTACAATATGCTAGTTATCCT
IGKV9-124*01_DBA2	DBA/2J	IGKV	MUSMUS IGKV9-124*01 F	100	GACATCCAGATGACCCAGTCTCCATCCTCCTTATCTGCCTCTCTGGGAGAAAGAGTCAGTCTCACTTGTCGGGCAAGTCAGGAAATTAGTGGTTACTTAAGCTGGCTTCAGCAGAAACCAGATGGAACTATTAAACGCCTGATCTACGCCGCATCCACTTTAGATTCTGGTGTCCCAAAAAGGTTCAGTGGCAGTAGGTCTGGGTCAGATTATTCTCTCACCATCAGCAGCCTTGAGTCTGAAGATTTTGCAGACTATTACTGTCTACAATATGCTAGTTATCCT

IGKV9-124*01_MRL	MRL/MPJ	IGKV	MUSMUS IGKV9-124*01 F	100	GACATCCAGATGACCCAGTCTCCATCCTCCTTATCTGCCTCTCTGGGAGAAAGAGTCAGTCTCACTTGTCGGGCAAGTCAGGAAATTAGTGGTTACTTAAGCTGGCTTCAGCAGAAACCAGATGGAACTATTAAACGCCTGATCTACGCCGCATCCACTTTAGATTCTGGTGTCCCAAAAAGGTTCAGTGGCAGTAGGTCTGGGTCAGATTATTCTCTCACCATCAGCAGCCTTGAGTCTGAAGATTTTGCAGACTATTACTGTCTACAATATGCTAGTTATCCT
IGKV9-124*01_S2023_CAST	CAST/EIJ	IGKV	MUSMUS IGKV9-124*01 F	98.92	GACATCCAGATGACCCAGTCTCCATCCTCCTTATCTGCCTCTCTGGGAGAAAGAGTCAGTCTCACTTGTCGGGCAAGTCAGGAAATTAGTGGTTACTTAAGCTGGCTTCAGCAGAAACCAGATGGAACTATTAAACGCCTGATCTACAGCACATCCACTTTAGATTCTGGTGTCCCAAAAAGGTTCAGTGGCAGTAGGTCTGGGTCAGATTATTCTCTCACCATCAGCAGCCTTGAGTCTGAAGATTTTGCAGACTATTACTGTCTACAATATGCTAGTTCTCCT
IGKV9-124*01_S2023_NOD	NOD/SHILTJ	IGKV	MUSMUS IGKV9-124*01 F	98.92	GACATCCAGATGACCCAGTCTCCATCCTCCTTATCTGCCTCTCTGGGAGAAAGAGTCAGTCTCACTTGTCGGGCAAGTCAGGAAATTAGTGGTTACTTAAGCTGGCTTCAGCAGAAACCAGATGGAACTATTAAACGCCTGATCTACAGCACATCCACTTTAGATTCTGGTGTCCCAAAAAGGTTCAGTGGCAGTAGGTCTGGGTCAGATTATTCTCTCACCATCAGCAGCCTTGAGTCTGAAGATTTTGCAGACTATTACTGTCTACAATATGCTAGTTCTCCT
IGKV9-124*01_S2023_NOR	NOR/LTJ	IGKV	MUSMUS IGKV9-124*01 F	98.92	GACATCCAGATGACCCAGTCTCCATCCTCCTTATCTGCCTCTCTGGGAGAAAGAGTCAGTCTCACTTGTCGGGCAAGTCAGGAAATTAGTGGTTACTTAAGCTGGCTTCAGCAGAAACCAGATGGAACTATTAAACGCCTGATCTACAGCACATCCACTTTAGATTCTGGTGTCCCAAAAAGGTTCAGTGGCAGTAGGTCTGGGTCAGATTATTCTCTCACCATCAGCAGCCTTGAGTCTGAAGATTTTGCAGACTATTACTGTCTACAATATGCTAGTTCTCCT

IGKV9-124*01_S2023_NZB	NZB/BLNJ	IGKV	MUSMUS IGKV9-124*01 F	98.92	GACATCCAGATGACCCAGTCTCCATCCTCCTTATCTGCCTCTCTGGGAGAAAGAGTCAGTCTCACTTGTCGGGCAAGTCAGGAAATTAGTGGTTACTTAAGCTGGCTTCAGCAGAAACCAGATGGAACTATTAAACGCCTGATCTACAGCACATCCACTTTAGATTCTGGTGTCCCAAAAAGGTTCAGTGGCAGTAGGTCTGGGTCAGATTATTCTCTCACCATCAGCAGCCTTGAGTCTGAAGATTTTGCAGACTATTACTGTCTACAATATGCTAGTTCTCCT
IGKV9-124*01_S3726_MSM	MSM/MSJ	IGKV	MUSMUS IGKV9-124*01 F	98.57	GACATCCAGATGACCCAGTCTCCATCCTCCTTATCTGTCTCTCTGGGAGAAAGAGTCAGTCTCACTTGTCGGGCAAGTCAGGAAATTAGTGGTTACTTAAGCTGGCTTCAGCAGAAACCAGATGGAACTATTAAACGCCTGATCTACAGCACATCCACTTTAGATTCTGGTGTCCCAAAAAGGTTCAGTGGCAGTAGGTCTGGGTCAGATTATTCTCTCACCATCAGCAGCCTTGAGTCTGAAGATTTTGCAGACTATTACTGTCTACAATATGCTAGTTCTCCT
IGKV9-124*01_S5062_PWD	PWD/PHJ	IGKV	MUSMUS IGKV9-124*01 F	99.64	GACATCCAGATGACCCAGTCTCCATCCTCCTTATCTGCCTCTCTGGGAGAAAGAGTCAGTCTCACTTGTCGGGCAAGTCAGGAAATTAGTGGTTACTTAAGCTGGCTTCAGCAGAAACCAGATGGAACTATTAAACGCCTGATCTACGCTGCATCCACTTTAGATTCTGGTGTCCCAAAAAGGTTCAGTGGCAGTAGGTCTGGGTCAGATTATTCTCTCACCATCAGCAGCCTTGAGTCTGAAGATTTTGCAGACTATTACTGTCTACAATATGCTAGTTATCCT
IGKV9-124*01_S5726_LEWES	LEWES/EIJ	IGKV	MUSMUS IGKV9-124*01 F	97.49	GACATCCAAATGACCCAGTCTCCATCTTCCTTATCTGCCTCTCTGGGAGAAAGAGTCAGTCTCACTTGTCGGGCAAGTCAGGATATTAGTGGTTACTTAAGCTGGCTTCAGCAGAAACCAGATGGAACTATTAAACGCCTGATCTACAGCACATCCACTTTAGATTCTGGTGTCCCAAAAAGGTTCAGTGGCAGTAGGTCTGGGTCAGATTATTCTCTCACCATCAGCAGCCTAGAGTCTGAAGATTTTGCAGACTATTACTGTCTACAATATGCTAGTTCTCCT

IGKV9-129*01_AJ	A/J	IGKV	MUSMUS IGKV9-129*01 ORF	100	GACATCCAGATGACCCAGTCTC<br>CATCCTCCTTATCTGCCTCTCTG<br>GGAGAAAGAGTCAGTCTCACTT<br>GCCGGGCAAGTCAGGACATTC<br>ATGGTTATTTAAACTTGTTTCAGC<br>AGAAACCAGGTGAAACTATTAAA<br>CACCTGATCTATGAAACATCCAA<br>TTTAGATTCTGGTGTCCCAAAAA<br>GGTTCAGTGGCAGTAGGTCTGG<br>GTCAGATTATTCTCTCATTATCG<br>GCAGCCTTGAGTCTGAAGATTTT<br>GCAGACTATTACTGTCTACAATA<br>TGCTAGTTCTCCTCC
IGKV9-129*01_AKR	AKR/J	IGKV	MUSMUS IGKV9-129*01 ORF	100	GACATCCAGATGACCCAGTCTC<br>CATCCTCCTTATCTGCCTCTCTG<br>GGAGAAAGAGTCAGTCTCACTT<br>GCCGGGCAAGTCAGGACATTC<br>ATGGTTATTTAAACTTGTTTCAGC<br>AGAAACCAGGTGAAACTATTAAA<br>CACCTGATCTATGAAACATCCAA<br>TTTAGATTCTGGTGTCCCAAAAA<br>GGTTCAGTGGCAGTAGGTCTGG<br>GTCAGATTATTCTCTCATTATCG<br>GCAGCCTTGAGTCTGAAGATTTT<br>GCAGACTATTACTGTCTACAATA<br>TGCTAGTTCTCCTCC
IGKV9-129*01_B6	C57BL/6J	IGKV	MUSMUS IGKV9-129*01 ORF	100	GACATCCAGATGACCCAGTCTC<br>CATCCTCCTTATCTGCCTCTCTG<br>GGAGAAAGAGTCAGTCTCACTT<br>GCCGGGCAAGTCAGGACATTC<br>ATGGTTATTTAAACTTGTTTCAGC<br>AGAAACCAGGTGAAACTATTAAA<br>CACCTGATCTATGAAACATCCAA<br>TTTAGATTCTGGTGTCCCAAAAA<br>GGTTCAGTGGCAGTAGGTCTGG<br>GTCAGATTATTCTCTCATTATCG<br>GCAGCCTTGAGTCTGAAGATTTT<br>GCAGACTATTACTGTCTACAATA<br>TGCTAGTTCTCCTCC
IGKV9-129*01_BALB	BALB/CBYJ	IGKV	MUSMUS IGKV9-129*01 ORF	100	GACATCCAGATGACCCAGTCTC<br>CATCCTCCTTATCTGCCTCTCTG<br>GGAGAAAGAGTCAGTCTCACTT<br>GCCGGGCAAGTCAGGACATTC<br>ATGGTTATTTAAACTTGTTTCAGC<br>AGAAACCAGGTGAAACTATTAAA<br>CACCTGATCTATGAAACATCCAA<br>TTTAGATTCTGGTGTCCCAAAAA<br>GGTTCAGTGGCAGTAGGTCTGG<br>GTCAGATTATTCTCTCATTATCG<br>GCAGCCTTGAGTCTGAAGATTTT<br>GCAGACTATTACTGTCTACAATA<br>TGCTAGTTCTCCTCC

IGKV9-129*01_C3H	C3H/HEJ	IGKV	MUSMUS IGKV9-129*01 ORF	100	GACATCCAGATGACCCAGTCTCCATCCTCCTTATCTGCCTCTCTGGGAGAAAGAGTCAGTCTCACTTGCCGGGCAAGTCAGGACATTCATGGTTATTTAAACTTGTTTCAGCAGAAACCAGGTGAAACTATTAAACACCTGATCTATGAAACATCCAATTTAGATTCTGGTGTCCCAAAAAGGTTCAGTGGCAGTAGGTCTGGGTCAGATTATTCTCTCATTATCGGCAGCCTTGAGTCTGAAGATTTTGCAGACTATTACTGTCTACAATATGCTAGTTCTCCTCC
IGKV9-129*01_DBA1	DBA/1J	IGKV	MUSMUS IGKV9-129*01 ORF	100	GACATCCAGATGACCCAGTCTCCATCCTCCTTATCTGCCTCTCTGGGAGAAAGAGTCAGTCTCACTTGCCGGGCAAGTCAGGACATTCATGGTTATTTAAACTTGTTTCAGCAGAAACCAGGTGAAACTATTAAACACCTGATCTATGAAACATCCAATTTAGATTCTGGTGTCCCAAAAAGGTTCAGTGGCAGTAGGTCTGGGTCAGATTATTCTCTCATTATCGGCAGCCTTGAGTCTGAAGATTTTGCAGACTATTACTGTCTACAATATGCTAGTTCTCCTCC
IGKV9-129*01_DBA2	DBA/2J	IGKV	MUSMUS IGKV9-129*01 ORF	100	GACATCCAGATGACCCAGTCTCCATCCTCCTTATCTGCCTCTCTGGGAGAAAGAGTCAGTCTCACTTGCCGGGCAAGTCAGGACATTCATGGTTATTTAAACTTGTTTCAGCAGAAACCAGGTGAAACTATTAAACACCTGATCTATGAAACATCCAATTTAGATTCTGGTGTCCCAAAAAGGTTCAGTGGCAGTAGGTCTGGGTCAGATTATTCTCTCATTATCGGCAGCCTTGAGTCTGAAGATTTTGCAGACTATTACTGTCTACAATATGCTAGTTCTCCTCC
IGLV1*01_129S6	129S1/SVLMJ	IGLV	MUSMUS IGLV1*01 F	100	CAGGCTGTTGTGACTCAGGAATCTGCACTCACCACATCACCTGGTGAAACAGTCACACTCACTTGTCGCTCAAGTACTGGGGCTGTTACAACTAGTAACTATGCCAACTGGGTCCAAGAAAAACCAGATCATTTATTCACTGGTCTAATAGGTGGTACCAACAACCGAGCTCCAGGTGTTCCTGCCAGATTCTCAGGCTCCCTGATTGGAGACAAGGCTGCCCTCACCATCACAGGGGCACAGACTGAGGATGAGGCAATATATTTCTGTGCTCTATGGTACAGCAACCATTTC

IGLV1*01_AJ	A/J	IGLV	MUSMUS IGLV1*01 F	100	CAGGCTGTTGTGACTCAGGAATCTGCACTCACCACATCACCTGGTGAAACAGTCACACTCACTTGTCGCTCAAGTACTGGGGCTGTTACAACTAGTAACTATGCCAACTGGGTCCAAGAAAAACCAGATCATTTATTCACTGGTCTAATAGGTGGTACCAACAACCGAGCTCCAGGTGTTCCTGCCAGATTCTCAGGCTCCCTGATTGGAGACAAGGCTGCCCTCACCATCACAGGGGCACAGACTGAGGATGAGGCAATATATTTCTGTGCTCTATGGTACAGCAACCATTTC
IGLV1*01_AKR	AKR/J	IGLV	MUSMUS IGLV1*01 F	100	CAGGCTGTTGTGACTCAGGAATCTGCACTCACCACATCACCTGGTGAAACAGTCACACTCACTTGTCGCTCAAGTACTGGGGCTGTTACAACTAGTAACTATGCCAACTGGGTCCAAGAAAAACCAGATCATTTATTCACTGGTCTAATAGGTGGTACCAACAACCGAGCTCCAGGTGTTCCTGCCAGATTCTCAGGCTCCCTGATTGGAGACAAGGCTGCCCTCACCATCACAGGGGCACAGACTGAGGATGAGGCAATATATTTCTGTGCTCTATGGTACAGCAACCATTTC
IGLV1*01_B6	C57BL/6J	IGLV	MUSMUS IGLV1*01 F	100	CAGGCTGTTGTGACTCAGGAATCTGCACTCACCACATCACCTGGTGAAACAGTCACACTCACTTGTCGCTCAAGTACTGGGGCTGTTACAACTAGTAACTATGCCAACTGGGTCCAAGAAAAACCAGATCATTTATTCACTGGTCTAATAGGTGGTACCAACAACCGAGCTCCAGGTGTTCCTGCCAGATTCTCAGGCTCCCTGATTGGAGACAAGGCTGCCCTCACCATCACAGGGGCACAGACTGAGGATGAGGCAATATATTTCTGTGCTCTATGGTACAGCAACCATTTC
IGLV1*01_BALB	BALB/CBYJ	IGLV	MUSMUS IGLV1*01 F	100	CAGGCTGTTGTGACTCAGGAATCTGCACTCACCACATCACCTGGTGAAACAGTCACACTCACTTGTCGCTCAAGTACTGGGGCTGTTACAACTAGTAACTATGCCAACTGGGTCCAAGAAAAACCAGATCATTTATTCACTGGTCTAATAGGTGGTACCAACAACCGAGCTCCAGGTGTTCCTGCCAGATTCTCAGGCTCCCTGATTGGAGACAAGGCTGCCCTCACCATCACAGGGGCACAGACTGAGGATGAGGCAATATATTTCTGTGCTCTATGGTACAGCAACCATTTC

IGLV1*01_C3H	C3H/HEJ	IGLV	MUSMUS IGLV1*01 F	100	CAGGCTGTTGTGACTCAGGAATCTGCACTCACCACATCACCTGGTGAAACAGTCACACTCACTTGTCGCTCAAGTACTGGGGCTGTTACAACTAGTAACTATGCCAACTGGGTCCAAGAAAAACCAGATCATTTATTCACTGGTCTAATAGGTGGTACCAACAACCGAGCTCCAGGTGTTCCTGCCAGATTCTCAGGCTCCCTGATTGGAGACAAGGCTGCCCTCACCATCACAGGGGCACAGACTGAGGATGAGGCAATATATTTCTGTGCTCTATGGTACAGCAACCATTTC
IGLV1*01_CAST	CAST/EIJ	IGLV	MUSMUS IGLV1*01 F	97.92	CAGGCTGTCGTGACTCAGGAATCTGCACTCACCACATCACCTGGTGAAACAGTCACACTCACTTGTCGCTCAAGTACTGGGGCTGTTACAACTAGTAACTATGCCAACTGGGTCCAAGAAAAAACAGACCATTTATTCACTGGTCTAATAGGTGGTACCAACAACCGAGCTCCAGGTGTTCCTGTCAGATTCTCAGGCTCCCTGATTGGAGACAAGGCTGCCCTCACCATCACAGGGGCACAGACTGAGGATGATGCAATGTATTTCTGTGCTCTATGGTACAGCAACCATTTC
IGLV1*01_CAST_2	CAST/EIJ	IGLV	MUSSPR IGLV2*01 F	97.57	CAGGCTGTTGTGACTCAGGAGTCTGTTCTCACCACATCACCTGGTGGTACAGTCATAATCACTTGTCGCTCAAGTACTGGGGCTGTTACAACTAGTAACTATGCCATTTGGGTCCAAGAAAAAGCAGATCATTTATTCGCTGGTGTAATAGGTGATACAAGCAACCGAGCTCCAGGTGTTCCTGCCAGATTCTCAGGCTCCTTGATTGGAGACAAGGCTGCCCTCACCATCACAGGGGCACAGACTGAGGATGAGGCAATATATTTCTGTGCTCTATGGTACAGCAACCATTTC
IGLV1*01_CBA	CBA/J	IGLV	MUSMUS IGLV1*01 F	100	CAGGCTGTTGTGACTCAGGAATCTGCACTCACCACATCACCTGGTGAAACAGTCACACTCACTTGTCGCTCAAGTACTGGGGCTGTTACAACTAGTAACTATGCCAACTGGGTCCAAGAAAAACCAGATCATTTATTCACTGGTCTAATAGGTGGTACCAACAACCGAGCTCCAGGTGTTCCTGCCAGATTCTCAGGCTCCCTGATTGGAGACAAGGCTGCCCTCACCATCACAGGGGCACAGACTGAGGATGAGGCAATATATTTCTGTGCTCTATGGTACAGCAACCATTTC

IGLV1*01_DBA1	DBA/1J	IGLV	MUSMUS IGLV1*01 F	100	CAGGCTGTTGTGACTCAGGAATCTGCACTCACCACATCACCTGGTGAAACAGTCACACTCACTTGTCGCTCAAGTACTGGGGCTGTTACAACTAGTAACTATGCCAACTGGGTCCAAGAAAAACCAGATCATTTATTCACTGGTCTAATAGGTGGTACCAACAACCGAGCTCCAGGTGTTCCTGCCAGATTCTCAGGCTCCCTGATTGGAGACAAGGCTGCCCTCACCATCACAGGGGCACAGACTGAGGATGAGGCAATATATTTCTGTGCTCTATGGTACAGCAACCATTTC
IGLV1*01_DBA2	DBA/2J	IGLV	MUSMUS IGLV1*01 F	100	CAGGCTGTTGTGACTCAGGAATCTGCACTCACCACATCACCTGGTGAAACAGTCACACTCACTTGTCGCTCAAGTACTGGGGCTGTTACAACTAGTAACTATGCCAACTGGGTCCAAGAAAAACCAGATCATTTATTCACTGGTCTAATAGGTGGTACCAACAACCGAGCTCCAGGTGTTCCTGCCAGATTCTCAGGCTCCCTGATTGGAGACAAGGCTGCCCTCACCATCACAGGGGCACAGACTGAGGATGAGGCAATATATTTCTGTGCTCTATGGTACAGCAACCATTTC
IGLV1*01_LEWES	LEWES/EIJ	IGLV	MUSMUS IGLV1*01 F	99.65	CAGGCTGTTGTGACTCAGGAATCTGCACTCACCACATCACCTGGTGAAACAGTCACACTCACTTGTCGCTCAAGTACTGGGGCTGTTACAACTAGTAACTATGCCAACTGGGTCCAAGAAAAACCAGATCATTTATTCACTGGTCTAATAGGTGGTACCAACAACCGAGCTCCAGGTGTTCCTGCCAGATTCTCAGGCTCCCTGATTGGAGACAAGGCTGCCCTCACCATCACAGGGGCACAGACTGAGGATGAAGCAATATATTTCTGTGCTCTATGGTACAGCAACCATTTC
IGLV1*01_LEWES_2	LEWES/EIJ	IGLV	MUSSPR IGLV2*01 F	97.92	CAGGCTGTTGTGACTCAGGAGTCTGTACTCACCACATCACCTGGTGGTACAGTCATAATCACTTGTCGCTCAAGTACTGGGGCTGTTACAACTAGTAACTATGCCATTTGGGTCCAAGAAAAAGCAGATCATTTATTCGCTGGTGTAATAGGTGATACAAGCAACCGAGCTCCAGGTGTTCCTGCCAGATTCTCAGGCTCCTTGATTGGAGACAAGGCTGCCCTCACCATCACAGGGGCACAGACTGAGGATGAGGCAATATATTTCTGTGCTCTATGGTACAGCAACCATTTC

IGLV1*01_MRL	MRL/MPJ	IGLV	MUSMUS IGLV1*01 F	100	CAGGCTGTTGTGACTCAGGAATCTGCACTCACCACATCACCTGGTGAAACAGTCACACTCACTTGTCGCTCAAGTACTGGGGCTGTTACAACTAGTAACTATGCCAACTGGGTCCAAGAAAAACCAGATCATTTATTCACTGGTCTAATAGGTGGTACCAACAACCGAGCTCCAGGTGTTCCTGCCAGATTCTCAGGCTCCCTGATTGGAGACAAGGCTGCCCTCACCATCACAGGGGCACAGACTGAGGATGAGGCAATATATTTCTGTGCTCTATGGTACAGCAACCATTTC
IGLV1*01_MSM	MSM/MSJ	IGLV	MUSMUS IGLV1*01 F	100	CAGGCTGTTGTGACTCAGGAATCTGCACTCACCACATCACCTGGTGAAACAGTCACACTCACTTGTCGCTCAAGTACTGGGGCTGTTACAACTAGTAACTATGCCAACTGGGTCCAAGAAAAACCAGATCATTTATTCACTGGTCTAATAGGTGGTACCAACAACCGAGCTCCAGGTGTTCCTGCCAGATTCTCAGGCTCCCTGATTGGAGACAAGGCTGCCCTCACCATCACAGGGGCACAGACTGAGGATGAGGCAATATATTTCTGTGCTCTATGGTACAGCAACCATTTC
IGLV1*01_NOD	NOD/SHILTJ	IGLV	MUSMUS IGLV1*01 F	100	CAGGCTGTTGTGACTCAGGAATCTGCACTCACCACATCACCTGGTGAAACAGTCACACTCACTTGTCGCTCAAGTACTGGGGCTGTTACAACTAGTAACTATGCCAACTGGGTCCAAGAAAAACCAGATCATTTATTCACTGGTCTAATAGGTGGTACCAACAACCGAGCTCCAGGTGTTCCTGCCAGATTCTCAGGCTCCCTGATTGGAGACAAGGCTGCCCTCACCATCACAGGGGCACAGACTGAGGATGAGGCAATATATTTCTGTGCTCTATGGTACAGCAACCATTTC
IGLV1*01_NOR	NOR/LTJ	IGLV	MUSMUS IGLV1*01 F	100	CAGGCTGTTGTGACTCAGGAATCTGCACTCACCACATCACCTGGTGAAACAGTCACACTCACTTGTCGCTCAAGTACTGGGGCTGTTACAACTAGTAACTATGCCAACTGGGTCCAAGAAAAACCAGATCATTTATTCACTGGTCTAATAGGTGGTACCAACAACCGAGCTCCAGGTGTTCCTGCCAGATTCTCAGGCTCCCTGATTGGAGACAAGGCTGCCCTCACCATCACAGGGGCACAGACTGAGGATGAGGCAATATATTTCTGTGCTCTATGGTACAGCAACCATTTC

IGLV1*01_NZB	NZB/BLNJ	IGLV	MUSMUS IGLV1*01 F	100	CAGGCTGTTGTGACTCAGGAATCTGCACTCACCACATCACCTGGTGAAACAGTCACACTCACTTGTCGCTCAAGTACTGGGGCTGTTACAACTAGTAACTATGCCAACTGGGTCCAAGAAAAACCAGATCATTTATTCACTGGTCTAATAGGTGGTACCAACAACCGAGCTCCAGGTGTTCCTGCCAGATTCTCAGGCTCCCTGATTGGAGACAAGGCTGCCCTCACCATCACAGGGGCACAGACTGAGGATGAGGCAATATATTTCTGTGCTCTATGGTACAGCAACCATTTC
IGLV1*01_PWD	PWD/PHJ	IGLV	MUSMUS IGLV1*01 F	100	CAGGCTGTTGTGACTCAGGAATCTGCACTCACCACATCACCTGGTGAAACAGTCACACTCACTTGTCGCTCAAGTACTGGGGCTGTTACAACTAGTAACTATGCCAACTGGGTCCAAGAAAAACCAGATCATTTATTCACTGGTCTAATAGGTGGTACCAACAACCGAGCTCCAGGTGTTCCTGCCAGATTCTCAGGCTCCCTGATTGGAGACAAGGCTGCCCTCACCATCACAGGGGCACAGACTGAGGATGAGGCAATATATTTCTGTGCTCTATGGTACAGCAACCATTTC
IGLV1*01_SJL	SJL/J	IGLV	MUSMUS IGLV1*01 F	100	CAGGCTGTTGTGACTCAGGAATCTGCACTCACCACATCACCTGGTGAAACAGTCACACTCACTTGTCGCTCAAGTACTGGGGCTGTTACAACTAGTAACTATGCCAACTGGGTCCAAGAAAAACCAGATCATTTATTCACTGGTCTAATAGGTGGTACCAACAACCGAGCTCCAGGTGTTCCTGCCAGATTCTCAGGCTCCCTGATTGGAGACAAGGCTGCCCTCACCATCACAGGGGCACAGACTGAGGATGAGGCAATATATTTCTGTGCTCTATGGTACAGCAACCATTTC
IGLV2*02_129S6	129S1/SVLMJ	IGLV	MUSMUS IGLV2*02 F	100	CAGGCTGTTGTGACTCAGGAATCTGCACTCACCACATCACCTGGTGGAACAGTCATACTCACTTGTCGCTCAAGTACTGGGGCTGTTACAACTAGTAACTATGCCAACTGGGTCCAAGAAAAACCAGATCATTTATTCACTGGTCTAATAGGTGGTACCAGCAACCGAGCTCCAGGTGTTCCTGTCAGATTCTCAGGCTCCCTGATTGGAGACAAGGCTGCCCTCACCATCACAGGGGCACAGACTGAGGATGATGCAATGTATTTCTGTGCTCTATGGTACAGCACCCATTTC

IGLV2*02_AJ	A/J	IGLV	MUSMUS IGLV2*02 F	100	CAGGCTGTTGTGACTCAGGAATCTGCACTCACCACATCACCTGGTGGAACAGTCATACTCACTTGTCGCTCAAGTACTGGGGCTGTTACAACTAGTAACTATGCCAACTGGGTCCAAGAAAAACCAGATCATTTATTCACTGGTCTAATAGGTGGTACCAGCAACCGAGCTCCAGGTGTTCCTGTCAGATTCTCAGGCTCCCTGATTGGAGACAAGGCTGCCCTCACCATCACAGGGGCACAGACTGAGGATGATGCAATGTATTTCTGTGCTCTATGGTACAGCACCCATTTC
IGLV2*02_AKR	AKR/J	IGLV	MUSMUS IGLV2*02 F	100	CAGGCTGTTGTGACTCAGGAATCTGCACTCACCACATCACCTGGTGGAACAGTCATACTCACTTGTCGCTCAAGTACTGGGGCTGTTACAACTAGTAACTATGCCAACTGGGTCCAAGAAAAACCAGATCATTTATTCACTGGTCTAATAGGTGGTACCAGCAACCGAGCTCCAGGTGTTCCTGTCAGATTCTCAGGCTCCCTGATTGGAGACAAGGCTGCCCTCACCATCACAGGGGCACAGACTGAGGATGATGCAATGTATTTCTGTGCTCTATGGTACAGCACCCATTTC
IGLV2*02_B6	C57BL/6J	IGLV	MUSMUS IGLV2*02 F	100	CAGGCTGTTGTGACTCAGGAATCTGCACTCACCACATCACCTGGTGGAACAGTCATACTCACTTGTCGCTCAAGTACTGGGGCTGTTACAACTAGTAACTATGCCAACTGGGTCCAAGAAAAACCAGATCATTTATTCACTGGTCTAATAGGTGGTACCAGCAACCGAGCTCCAGGTGTTCCTGTCAGATTCTCAGGCTCCCTGATTGGAGACAAGGCTGCCCTCACCATCACAGGGGCACAGACTGAGGATGATGCAATGTATTTCTGTGCTCTATGGTACAGCACCCATTTC
IGLV2*02_BALB	BALB/CBYJ	IGLV	MUSMUS IGLV2*02 F	100	CAGGCTGTTGTGACTCAGGAATCTGCACTCACCACATCACCTGGTGGAACAGTCATACTCACTTGTCGCTCAAGTACTGGGGCTGTTACAACTAGTAACTATGCCAACTGGGTCCAAGAAAAACCAGATCATTTATTCACTGGTCTAATAGGTGGTACCAGCAACCGAGCTCCAGGTGTTCCTGTCAGATTCTCAGGCTCCCTGATTGGAGACAAGGCTGCCCTCACCATCACAGGGGCACAGACTGAGGATGATGCAATGTATTTCTGTGCTCTATGGTACAGCACCCATTTC

IGLV2*02_C3H	C3H/HEJ	IGLV	MUSMUS IGLV2*02 F	100	CAGGCTGTTGTGACTCAGGAATCTGCACTCACCACATCACCTGGTGGAACAGTCATACTCACTTGTCGCTCAAGTACTGGGGCTGTTACAACTAGTAACTATGCCAACTGGGTCCAAGAAAAACCAGATCATTTATTCACTGGTCTAATAGGTGGTACCAGCAACCGAGCTCCAGGTGTTCCTGTCAGATTCTCAGGCTCCCTGATTGGAGACAAGGCTGCCCTCACCATCACAGGGGCACAGACTGAGGATGATGCAATGTATTTCTGTGCTCTATGGTACAGCACCCATTTC
IGLV2*02_CAST	CAST/EIJ	IGLV	MUSMUS IGLV2*02 F	99.65	CAGGCTGTTGTGACTCAGGAATCTGCACTCACCACATCACCTGGTGGAACAGTCATACTCACTTGTCGCTCAAGTACTGGGGCTGTTACAACTAGTAACTATGCCAACTGGGTCCAAGAAAAACCAGATCATTTATTCACTGGTCTAATAGGTGGTACCAGCAACCGAGCTCCAGGTGTTCCAGTCAGATTCTCAGGCTCCCTGATTGGAGACAAGGCTGCCCTCACCATCACAGGGGCACAGACTGAGGATGATGCAATGTATTTCTGTGCTCTATGGTACAGCACCCATTTC
IGLV2*02_CBA	CBA/J	IGLV	MUSMUS IGLV2*02 F	100	CAGGCTGTTGTGACTCAGGAATCTGCACTCACCACATCACCTGGTGGAACAGTCATACTCACTTGTCGCTCAAGTACTGGGGCTGTTACAACTAGTAACTATGCCAACTGGGTCCAAGAAAAACCAGATCATTTATTCACTGGTCTAATAGGTGGTACCAGCAACCGAGCTCCAGGTGTTCCTGTCAGATTCTCAGGCTCCCTGATTGGAGACAAGGCTGCCCTCACCATCACAGGGGCACAGACTGAGGATGATGCAATGTATTTCTGTGCTCTATGGTACAGCACCCATTTC
IGLV2*02_DBA1	DBA/1J	IGLV	MUSMUS IGLV2*02 F	100	CAGGCTGTTGTGACTCAGGAATCTGCACTCACCACATCACCTGGTGGAACAGTCATACTCACTTGTCGCTCAAGTACTGGGGCTGTTACAACTAGTAACTATGCCAACTGGGTCCAAGAAAAACCAGATCATTTATTCACTGGTCTAATAGGTGGTACCAGCAACCGAGCTCCAGGTGTTCCTGTCAGATTCTCAGGCTCCCTGATTGGAGACAAGGCTGCCCTCACCATCACAGGGGCACAGACTGAGGATGATGCAATGTATTTCTGTGCTCTATGGTACAGCACCCATTTC

IGLV2*02_DBA2	DBA/2J	IGLV	MUSMUS IGLV2*02 F	100	CAGGCTGTTGTGACTCAGGAATCTGCACTCACCACATCACCTGGTGGAACAGTCATACTCACTTGTCGCTCAAGTACTGGGGCTGTTACAACTAGTAACTATGCCAACTGGGTCCAAGAAAAACCAGATCATTTATTCACTGGTCTAATAGGTGGTACCAGCAACCGAGCTCCAGGTGTTCCTGTCAGATTCTCAGGCTCCCTGATTGGAGACAAGGCTGCCCTCACCATCACAGGGGCACAGACTGAGGATGATGCAATGTATTTCTGTGCTCTATGGTACAGCACCCATTTC
IGLV2*02_LEWES	LEWES/EIJ	IGLV	MUSMUS IGLV2*02 F	100	CAGGCTGTTGTGACTCAGGAATCTGCACTCACCACATCACCTGGTGGAACAGTCATACTCACTTGTCGCTCAAGTACTGGGGCTGTTACAACTAGTAACTATGCCAACTGGGTCCAAGAAAAACCAGATCATTTATTCACTGGTCTAATAGGTGGTACCAGCAACCGAGCTCCAGGTGTTCCTGTCAGATTCTCAGGCTCCCTGATTGGAGACAAGGCTGCCCTCACCATCACAGGGGCACAGACTGAGGATGATGCAATGTATTTCTGTGCTCTATGGTACAGCACCCATTTC
IGLV2*02_MRL	MRL/MPJ	IGLV	MUSMUS IGLV2*02 F	100	CAGGCTGTTGTGACTCAGGAATCTGCACTCACCACATCACCTGGTGGAACAGTCATACTCACTTGTCGCTCAAGTACTGGGGCTGTTACAACTAGTAACTATGCCAACTGGGTCCAAGAAAAACCAGATCATTTATTCACTGGTCTAATAGGTGGTACCAGCAACCGAGCTCCAGGTGTTCCTGTCAGATTCTCAGGCTCCCTGATTGGAGACAAGGCTGCCCTCACCATCACAGGGGCACAGACTGAGGATGATGCAATGTATTTCTGTGCTCTATGGTACAGCACCCATTTC
IGLV2*02_MSM	MSM/MSJ	IGLV	MUSMUS IGLV2*02 F	100	CAGGCTGTTGTGACTCAGGAATCTGCACTCACCACATCACCTGGTGGAACAGTCATACTCACTTGTCGCTCAAGTACTGGGGCTGTTACAACTAGTAACTATGCCAACTGGGTCCAAGAAAAACCAGATCATTTATTCACTGGTCTAATAGGTGGTACCAGCAACCGAGCTCCAGGTGTTCCTGTCAGATTCTCAGGCTCCCTGATTGGAGACAAGGCTGCCCTCACCATCACAGGGGCACAGACTGAGGATGATGCAATGTATTTCTGTGCTCTATGGTACAGCACCCATTTC

IGLV2*02_NOD	NOD/SHILTJ	IGLV	MUSMUS IGLV2*02 F	100	CAGGCTGTTGTGACTCAGGAATCTGCACTCACCACATCACCTGGTGGAACAGTCATACTCACTTGTCGCTCAAGTACTGGGGCTGTTACAACTAGTAACTATGCCAACTGGGTCCAAGAAAAACCAGATCATTTATTCACTGGTCTAATAGGTGGTACCAGCAACCGAGCTCCAGGTGTTCCTGTCAGATTCTCAGGCTCCCTGATTGGAGACAAGGCTGCCCTCACCATCACAGGGGCACAGACTGAGGATGATGCAATGTATTTCTGTGCTCTATGGTACAGCACCCATTTC
IGLV2*02_NOR	NOR/LTJ	IGLV	MUSMUS IGLV2*02 F	100	CAGGCTGTTGTGACTCAGGAATCTGCACTCACCACATCACCTGGTGGAACAGTCATACTCACTTGTCGCTCAAGTACTGGGGCTGTTACAACTAGTAACTATGCCAACTGGGTCCAAGAAAAACCAGATCATTTATTCACTGGTCTAATAGGTGGTACCAGCAACCGAGCTCCAGGTGTTCCTGTCAGATTCTCAGGCTCCCTGATTGGAGACAAGGCTGCCCTCACCATCACAGGGGCACAGACTGAGGATGATGCAATGTATTTCTGTGCTCTATGGTACAGCACCCATTTC
IGLV2*02_NZB	NZB/BLNJ	IGLV	MUSMUS IGLV2*02 F	100	CAGGCTGTTGTGACTCAGGAATCTGCACTCACCACATCACCTGGTGGAACAGTCATACTCACTTGTCGCTCAAGTACTGGGGCTGTTACAACTAGTAACTATGCCAACTGGGTCCAAGAAAAACCAGATCATTTATTCACTGGTCTAATAGGTGGTACCAGCAACCGAGCTCCAGGTGTTCCTGTCAGATTCTCAGGCTCCCTGATTGGAGACAAGGCTGCCCTCACCATCACAGGGGCACAGACTGAGGATGATGCAATGTATTTCTGTGCTCTATGGTACAGCACCCATTTC
IGLV2*02_PWD	PWD/PHJ	IGLV	MUSMUS IGLV2*02 F	99.65	CAGGCTGTTGTGACTCAGGAATCTGCACTCACCACATCACCTGGTGGAACAGTCATACTCACTTGTCGCTCAAGTACTGGGGCTGTTACAACTAGTAACTATGCCAACTGGGTCCAAGAAAAACCAGATCATTTATTCACTGGTCTAATAGGTGGTACCAGCAACCGAGCTCCAGGTGTTCCAGTCAGATTCTCAGGCTCCCTGATTGGAGACAAGGCTGCCCTCACCATCACAGGGGCACAGACTGAGGATGATGCAATGTATTTCTGTGCTCTATGGTACAGCACCCATTTC

IGLV2*02_SJL	SJL/J	IGLV	MUSMUS IGLV2*02 F	100	CAGGCTGTTGTGACTCAGGAATCTGCACTCACCACATCACCTGGTGGAACAGTCATACTCACTTGTCGCTCAAGTACTGGGGCTGTTACAACTAGTAACTATGCCAACTGGGTCCAAGAAAAACCAGATCATTTATTCACTGGTCTAATAGGTGGTACCAGCAACCGAGCTCCAGGTGTTCCTGTCAGATTCTCAGGCTCCCTGATTGGAGACAAGGCTGCCCTCACCATCACAGGGGCACAGACTGAGGATGATGCAATGTATTTCTGTGCTCTATGGTACAGCACCCATTTC
IGLV3*01_129S6	129S1/SVLMJ	IGLV	MUSMUS IGLV3*01 F	100	CAACTTGTGCTCACTCAGTCATCTTCAGCCTCTTTCTCCCTGGGAGCCTCAGCAAAACTCACGTGCACCTTGAGTAGTCAGCACAGTACGTACACCATTGAATGGTATCAGCAACAGCCACTCAAGCCTCCTAAGTATGTGATGGAGCTTAAGAAAGATGGAAGCCACAGCACAGGTGATGGGATTCCTGATCGCTTCTCTGGATCCAGCTCTGGTGCTGATCGCTACCTTAGCATTTCCAACATCCAGCCTGAAGATGAAGCAATATACATCTGTGGTGTGGGTGATACAATTAAGGAACAATTTGTGTAAC
IGLV3*01_AJ	A/J	IGLV	MUSMUS IGLV3*01 F	100	CAACTTGTGCTCACTCAGTCATCTTCAGCCTCTTTCTCCCTGGGAGCCTCAGCAAAACTCACGTGCACCTTGAGTAGTCAGCACAGTACGTACACCATTGAATGGTATCAGCAACAGCCACTCAAGCCTCCTAAGTATGTGATGGAGCTTAAGAAAGATGGAAGCCACAGCACAGGTGATGGGATTCCTGATCGCTTCTCTGGATCCAGCTCTGGTGCTGATCGCTACCTTAGCATTTCCAACATCCAGCCTGAAGATGAAGCAATATACATCTGTGGTGTGGGTGATACAATTAAGGAACAATTTGTGTAAC
IGLV3*01_AKR	AKR/J	IGLV	MUSMUS IGLV3*01 F	100	CAACTTGTGCTCACTCAGTCATCTTCAGCCTCTTTCTCCCTGGGAGCCTCAGCAAAACTCACGTGCACCTTGAGTAGTCAGCACAGTACGTACACCATTGAATGGTATCAGCAACAGCCACTCAAGCCTCCTAAGTATGTGATGGAGCTTAAGAAAGATGGAAGCCACAGCACAGGTGATGGGATTCCTGATCGCTTCTCTGGATCCAGCTCTGGTGCTGATCGCTACCTTAGCATTTCCAACATCCAGCCTGAAGATGAAGCAATATACATCTGTGGTGTGGGTGATACAATTAAGGAACAATTTGTGTAAC

IGLV3*01_B6	C57BL/6J	IGLV	MUSMUS IGLV3*01 F	100	CAACTTGTGCTCACTCAGTCATCTTCAGCCTCTTTCTCCCTGGGAGCCTCAGCAAAACTCACGTGCACCTTGAGTAGTCAGCACAGTACGTACACCATTGAATGGTATCAGCAACAGCCACTCAAGCCTCCTAAGTATGTGATGGAGCTTAAGAAAGATGGAAGCCACAGCACAGGTGATGGGATTCCTGATCGCTTCTCTGGATCCAGCTCTGGTGCTGATCGCTACCTTAGCATTTCCAACATCCAGCCTGAAGATGAAGCAATATACATCTGTGGTGTGGGTGATACAATTAAGGAACAATTTGTGTAAC
IGLV3*01_BALB	BALB/CBYJ	IGLV	MUSMUS IGLV3*01 F	100	CAACTTGTGCTCACTCAGTCATCTTCAGCCTCTTTCTCCCTGGGAGCCTCAGCAAAACTCACGTGCACCTTGAGTAGTCAGCACAGTACGTACACCATTGAATGGTATCAGCAACAGCCACTCAAGCCTCCTAAGTATGTGATGGAGCTTAAGAAAGATGGAAGCCACAGCACAGGTGATGGGATTCCTGATCGCTTCTCTGGATCCAGCTCTGGTGCTGATCGCTACCTTAGCATTTCCAACATCCAGCCTGAAGATGAAGCAATATACATCTGTGGTGTGGGTGATACAATTAAGGAACAATTTGTGTAAC
IGLV3*01_C3H	C3H/HEJ	IGLV	MUSMUS IGLV3*01 F	100	CAACTTGTGCTCACTCAGTCATCTTCAGCCTCTTTCTCCCTGGGAGCCTCAGCAAAACTCACGTGCACCTTGAGTAGTCAGCACAGTACGTACACCATTGAATGGTATCAGCAACAGCCACTCAAGCCTCCTAAGTATGTGATGGAGCTTAAGAAAGATGGAAGCCACAGCACAGGTGATGGGATTCCTGATCGCTTCTCTGGATCCAGCTCTGGTGCTGATCGCTACCTTAGCATTTCCAACATCCAGCCTGAAGATGAAGCAATATACATCTGTGGTGTGGGTGATACAATTAAGGAACAATTTGTGTAAC
IGLV3*01_CAST	CAST/EIJ	IGLV	MUSSPR IGLV3*01 F	96.26	CAACCTGTGCTCACTCAGTCATCTTCAGCCTCTTTCTCCCTGGGAGCCTCAGCAAAACTCACATGCACCTTGAGTAGTGAGCACAGTACGTACATCATTGAATGGTACCAGCAACAGCCACTCAAGCCTCCTAAGTATGTGATGCAGCTTAAGAAAGATGGAAGCCACAGCAAGGGTGATGGAATCCCTGATCGCTTCTCTGGATCCAGCTCTGGTGCTGACCGCTACCTTAGCATCTCCAACATCCAGCCTGAAGATGAAGCAATATACATCTGTGGTGTGAGTGGAACAATTAAGGAACAATTCGTGTAAC

IGLV3*01_CBA	CBA/J	IGLV	MUSMUS IGLV3*01 F	100	CAACTTGTGCTCACTCAGTCATCTTCAGCCTCTTTCTCCCTGGGAGCCTCAGCAAAACTCACGTGCACCTTGAGTAGTCAGCACAGTACGTACACCATTGAATGGTATCAGCAACAGCCACTCAAGCCTCCTAAGTATGTGATGGAGCTTAAGAAAGATGGAAGCCACAGCACAGGTGATGGGATTCCTGATCGCTTCTCTGGATCCAGCTCTGGTGCTGATCGCTACCTTAGCATTTCCAACATCCAGCCTGAAGATGAAGCAATATACATCTGTGGTGTGGGTGATACAATTAAGGAACAATTTGTGTAAC
IGLV3*01_DBA1	DBA/1J	IGLV	MUSMUS IGLV3*01 F	100	CAACTTGTGCTCACTCAGTCATCTTCAGCCTCTTTCTCCCTGGGAGCCTCAGCAAAACTCACGTGCACCTTGAGTAGTCAGCACAGTACGTACACCATTGAATGGTATCAGCAACAGCCACTCAAGCCTCCTAAGTATGTGATGGAGCTTAAGAAAGATGGAAGCCACAGCACAGGTGATGGGATTCCTGATCGCTTCTCTGGATCCAGCTCTGGTGCTGATCGCTACCTTAGCATTTCCAACATCCAGCCTGAAGATGAAGCAATATACATCTGTGGTGTGGGTGATACAATTAAGGAACAATTTGTGTAAC
IGLV3*01_DBA2	DBA/2J	IGLV	MUSMUS IGLV3*01 F	100	CAACTTGTGCTCACTCAGTCATCTTCAGCCTCTTTCTCCCTGGGAGCCTCAGCAAAACTCACGTGCACCTTGAGTAGTCAGCACAGTACGTACACCATTGAATGGTATCAGCAACAGCCACTCAAGCCTCCTAAGTATGTGATGGAGCTTAAGAAAGATGGAAGCCACAGCACAGGTGATGGGATTCCTGATCGCTTCTCTGGATCCAGCTCTGGTGCTGATCGCTACCTTAGCATTTCCAACATCCAGCCTGAAGATGAAGCAATATACATCTGTGGTGTGGGTGATACAATTAAGGAACAATTTGTGTAAC
IGLV3*01_LEWES	LEWES/EIJ	IGLV	MUSSPR IGLV3*01 F	97.28	CAACCTGTGCTCACTCAGTCATCTTCAGCCTCTTTCTCCCTGGGAGCCTCAGCAAAACTCACATGCACCTTGAGTAGTGAGCACAGTACGTACTTCATTGAATGGTACCAGCAACAGCCACTCAAGCCTCCTAAGTATGTGATGCAGCTTAAGAAAGATGGAAGCCACAGCAAGGGTGATGGGATCCCTGATCGCTTCTCTGGATCCAGCTCTGGTGCTGATCGCTACCTTAGCATCTCCAACATCCAGCCTGAAGATGAAGCAATATACATCTGTGGTGTGGGTGAAACAATTAAGGAACGATACGTGTAAC

IGLV3*01_MRL	MRL/MPJ	IGLV	MUSMUS IGLV3*01 F	100	CAACTTGTGCTCACTCAGTCATC<br>TTCAGCCTCTTTCTCCCTGGGA<br>GCCTCAGCAAAACTCACGTGCA<br>CCTTGAGTAGTCAGCACAGTAC<br>GTACACCATTGAATGGTATCAGC<br>AACAGCCACTCAAGCCTCCTAA<br>GTATGTGATGGAGCTTAAGAAAG<br>ATGGAAGCCACAGCACAGGTG<br>ATGGGATTCCTGATCGCTTCTCT<br>GGATCCAGCTCTGGTGCTGATC<br>GCTACCTTAGCATTTCCAACATC<br>CAGCCTGAAGATGAAGCAATAT<br>ACATCTGTGGTGTGGGTGATAC<br>AATTAAGGAACAATTTGTGTAAC
IGLV3*01_MSM	MSM/MSJ	IGLV	MUSMUS IGLV3*01 F	99.66	CAACTTGTGCTCACTCAGTCATC<br>TTCAGCCTCTTTCTCCCTGGGA<br>GCCTCAGCAAAACTCACGTGCA<br>CCTTGAGTAGTCAGCACAGTAC<br>GTACACCATTGAATGGTATCAGC<br>AACAGCCACTCAAGCCTCCTAA<br>GTATGTGATGGAGCTTAAGAAAG<br>ATGGAAGCCACAGCACAGGTG<br>ATGGGATCCCTGATCGCTTCTC<br>TGGATCCAGCTCTGGTGCTGAT<br>CGCTACCTTAGCATTTCCAACAT<br>CCAGCCTGAAGATGAAGCAATA<br>TACATCTGTGGTGTGGGTGATAC<br>AATTAAGGAACAATTTGTGTAAC
IGLV3*01_NOD	NOD/SHILTJ	IGLV	MUSMUS IGLV3*01 F	100	CAACTTGTGCTCACTCAGTCATC<br>TTCAGCCTCTTTCTCCCTGGGA<br>GCCTCAGCAAAACTCACGTGCA<br>CCTTGAGTAGTCAGCACAGTAC<br>GTACACCATTGAATGGTATCAGC<br>AACAGCCACTCAAGCCTCCTAA<br>GTATGTGATGGAGCTTAAGAAAG<br>ATGGAAGCCACAGCACAGGTG<br>ATGGGATTCCTGATCGCTTCTCT<br>GGATCCAGCTCTGGTGCTGATC<br>GCTACCTTAGCATTTCCAACATC<br>CAGCCTGAAGATGAAGCAATAT<br>ACATCTGTGGTGTGGGTGATAC<br>AATTAAGGAACAATTTGTGTAAC
IGLV3*01_NOR	NOR/LTJ	IGLV	MUSMUS IGLV3*01 F	100	CAACTTGTGCTCACTCAGTCATC<br>TTCAGCCTCTTTCTCCCTGGGA<br>GCCTCAGCAAAACTCACGTGCA<br>CCTTGAGTAGTCAGCACAGTAC<br>GTACACCATTGAATGGTATCAGC<br>AACAGCCACTCAAGCCTCCTAA<br>GTATGTGATGGAGCTTAAGAAAG<br>ATGGAAGCCACAGCACAGGTG<br>ATGGGATTCCTGATCGCTTCTCT<br>GGATCCAGCTCTGGTGCTGATC<br>GCTACCTTAGCATTTCCAACATC<br>CAGCCTGAAGATGAAGCAATAT<br>ACATCTGTGGTGTGGGTGATAC<br>AATTAAGGAACAATTTGTGTAAC

IGLV3*01_NZB	NZB/BLNJ	IGLV	MUSMUS IGLV3*01 F	100	CAACTTGTGCTCACTCAGTCATCTTCAGCCTCTTTCTCCCTGGGAGCCTCAGCAAAACTCACGTGCACCTTGAGTAGTCAGCACAGTACGTACACCATTGAATGGTATCAGCAACAGCCACTCAAGCCTCCTAAGTATGTGATGGAGCTTAAGAAAGATGGAAGCCACAGCACAGGTGATGGGATTCCTGATCGCTTCTCTGGATCCAGCTCTGGTGCTGATCGCTACCTTAGCATTTCCAACATCCAGCCTGAAGATGAAGCAATATACATCTGTGGTGTGGGTGATACAATTAAGGAACAATTTGTGTAAC
IGLV3*01_PWD	PWD/PHJ	IGLV	MUSSPR IGLV3*01 F	95.92	CAACCTGTGCTCACTCAGTCATCTTCAGCCTCTTTCTCCCTGGGAGCCTCAGCAAAACTCACATGCACCTTGAGTAGTGAGCACAGTACGTACATCATTGAATGGTACCAGCAACAGCCACTCAAGCCTCCTAAGTATGTGATGCAGCTTAAGAAAGATGGAAGCCACAGCAAGGGTGATGGAATCCCTGATCGCTTCTCTGGATCCAGCTCTGGTGCTGACCGCTACCTTAACATCTCCAACATCCAGCCTGAAGATGAAGCAATATACATCTGTGGTGTGAGTGGAACAATTAAGGAACAATTCGTGTAAC
IGLV3*01_SJL	SJL/J	IGLV	MUSMUS IGLV3*01 F	100	CAACTTGTGCTCACTCAGTCATCTTCAGCCTCTTTCTCCCTGGGAGCCTCAGCAAAACTCACGTGCACCTTGAGTAGTCAGCACAGTACGTACACCATTGAATGGTATCAGCAACAGCCACTCAAGCCTCCTAAGTATGTGATGGAGCTTAAGAAAGATGGAAGCCACAGCACAGGTGATGGGATTCCTGATCGCTTCTCTGGATCCAGCTCTGGTGCTGATCGCTACCTTAGCATTTCCAACATCCAGCCTGAAGATGAAGCAATATACATCTGTGGTGTGGGTGATACAATTAAGGAACAATTTGTGTAAC
IGLV4*01_CAST	CAST/EIJ	IGLV	MUSMUS IGLV4*01 [F]	100	TTACTCAGCCAAGCTCTGTGTCTACGTCTCTAGGAAGCACAGTCAAACTGTCTTGCAAGCGCAGCACTGGTAACATTGGAAACAACTATGTGCACTGGTACCAGCAGTACATGGGAAGATCTCCCACCAATATGATCTATGATGATAATAAGCGACCATCTGGAGTTTCTGATAGGTTCTCTGGCTCCATTGACAGCTCTTCCAACTCAGCCTTCCTGACAATCAATAATGTGCAGGCTGAGGAT

IGLV5*01_CAST	CAST/EIJ	IGLV	MUSMUS IGLV5*01 [F]	93.88	TTACTCAGCCAAGCTCTGTGTCTACGTCTCTAGGAAGCACAGCCAAACTGCCTTGCAAGGCCAGCACTGGTAACATTGGAGACAGCTATGTGAACTGGTACCAGCAGTACATGGGAAGATCTCCCACTAATATAATCTATGGAGATGATCTCCGACCATCTGGAGTTTCTGATAGGTTCTCTGGCTCCATTGACAGCTCTTCCAACTCAGCCTTCCTGACAATCCAAAATGTGCAGGCTGATGAT
IGLV5*01_MSM	MSM/MSJ	IGLV	MUSMUS IGLV5*01 [F]	93.88	TTACTCAGCCAAGCTCTGTGTCTACGTCTCTAGGAAGCACAGCCAAACTGCCTTGCAAGGCCAGCACTGGTAACATTGGAGACAGCTATGTGAACTGGTACCAGCAGTACATGGGAAGATCTCCCACTAATATGATCTATGGAGATGATCTCCGACCATCTGGAGTTTCTGATAGGTTCTCTGGCTCCATTCACAGCTCTTCCAACTCAGCCTTCCTGACAATCCAAAATGTGCAGGCTGATGAT
IGLV6*01_CAST	CAST/EIJ	IGLV	MUSMUS IGLV6*01 [F]	98.78	TTACTCAGCCAAGCTCTGTGTCTACATCTCTAGGAAGCACAGTCAAACTGCCTTGCAAGCGCAGCACTGATAATATTGGAAGCTACTATGTGCATTGGTACCAGCAACATATGGGAAGATCTCCTACCAATATGATCCATAGTGATGATCAGCGACCATCTGGAGTTTCTGATAGGTTCTCTGGCTCCATTGACAGCTCTTCCAACTCAGCCTTCCTGACAATCAATAATGTTCAGGCTGAGGAT
IGLV6*03_MSM	MSM/MSJ	IGLV	MUSMUS IGLV6*03 [F]	99.59	TTACTCAGCCAAGCTCTGTGTCTACATCTCTAGGAAGCACAGTCAAACTGCCTTGCAAGCGCAGCACTGGAAATATTGGAAGCTACTATGTGCATTGGTACCAGCAACATATGGGAAGATCTCCCACCAATATGATCCATAGTGATGATCAGCGACCATCTGGAGTTTCTGATAGGTTCTCTGGCTCCATTGACAGCTCTTCCAACTCAGCCTTCCTGACAATCAATAATGTTCAGGCTGAGGAT
IGLV7*01_CAST	CAST/EIJ	IGLV	MUSMUS IGLV7*01 [F]	100	TTACTCAGCCAAGCTCTGTGTCTACGTCTCTAGGAAGCACAGTCAAACTGTCTTGCAAGCCCAGCACTGGTAAAATTGGAAATTACTTTATGAGCTGGTACCAGCAACACATGGGAAGATCTCCCACAAATATGATCTATAGAGATGATCTCCGACCATCTGGAGTTTCTGATAGGTTCTCTGGCTCCATTGACAGCTCTTCCAACTCAGCCTTCCTGACAATCAATAATGTGCAGGCTGAGGAT

IGLV8*01_CAST	CAST/EIJ	IGLV	MUSMUS IGLV8*01 [F]	100	TTACTCAGCCAAGCTCTGTGTCTACGTCTCTAGGAAGCACAGTCAAACTGCCTTGCAAGCGCAGCACTGGTAACATTGGAAACGACTATGTGCACTGGTACCAGCAACACATGGGAAGATCTCCCACCAATATGATCTATAGAGATGATCAGCGACCATCTGGAGTTTCTGATAGGTTCTCTGGCTCCATTGACAGCTCTTCCAACTCAGCCTTCCTGACAATCAATAATGTGCAGGCTGAGGAT
